# Effect of Birth Interval on Foetal and Postnatal Child Growth

**DOI:** 10.1155/2021/6624184

**Published:** 2021-08-20

**Authors:** Mahama Saaka, Benjamin Aggrey

**Affiliations:** ^1^University for Development Studies, School of Allied Health Sciences, P.O. Box 1883, Tamale, Ghana; ^2^Ghana Health Service, P.O. Box 1883, Navrongo, Ghana

## Abstract

**Background:**

Although available evidence suggests short birth intervals are associated with adverse perinatal outcomes, little is known about the extent to which birth spacing affects postnatal child growth. The present study assessed the independent association of birth interval with birth weight and subsequent postnatal growth indices.

**Methods:**

This retrospective cohort study carried out in the rural areas of Kassena-Nankana district of Ghana compared postnatal growth across different categories of birth intervals. Birth intervals were calculated as month difference between consecutive births of a woman. The study population comprised 530 postpartum women who had delivered a live baby in the past 24 months prior to the study.

**Results:**

Using the analysis of covariance (ANCOVA) that adjusted for age of the child, gender of the child, weight-for-length *z*-score (WLZ), birth weight, adequacy of antenatal care (ANC) attendance, and dietary diversity of the child, the mean length-for-age *z*-score (LAZ) among children of short preceding birth interval (<24 months) was significantly higher than among children of long birth interval (that is, at least 24 months) ((0.51 versus −0.04) (95% CI: 0.24–0.87), *p* = 0.001). The adjusted mean birth weight of children born to mothers of longer birth interval was 74.0 g more than children born to mothers of shorter birth interval (CI: 5.89–142.0, *p*< 0.03).

**Conclusions:**

The results suggest that a short birth interval is associated positively with an increased risk of low birth weight (an indicator of foetal growth), but birth spacing is associated negatively with the LAZ (an indicator of postnatal growth).

## 1. Introduction

Childhood undernutrition remains global public health concern especially in low- and middle-income countries because as many as 165 million children are estimated to be stunted and it is an underlying cause of 3.1 million child deaths annually [1]. In 2016, 45% of the 5.6 million children under the age of 5 years who died were attributed to undernutrition [2]. Stunting, which is defined as low height for age, also contributes to the global burden of childhood diseases, 80% of which is affecting children in developing countries [3].

In sub-Saharan Africa, it has been reported that 34% of children under 5 years are stunted [4]. Global assessment of stunting in low- and middle-income countries revealed that the causes of stunting are multifaceted including poor nutrition, infectious diseases, and household environment [5] and that growth restriction in utero and lack of access to sanitation are the main drivers of stunting [6].

Although a multitude of risk factors have been documented, these vary from place to place and are therefore context specific. Furthermore, a full understanding of these factors and their relative effects is key to help the priority setting in designing policies and interventions to improve childhood growth.

One factor that has not received full investigation in the search for solutions to child undernutrition is the relationship between birth interval and child growth. It is well documented that child nutritional status is a direct outcome of the maternal environment during gestation and that the mother's system needs a reasonable amount of time for the restoration of nutrient reserves to provide optimum nutrition during a new gestation [7, 8]. Well-nourished women will require less time for maternal nutrient repletion than in poorly nourished women. When there is inadequate time between pregnancies for the mother's reproductive system to return to optimum biological functioning, there is a likelihood that inadequate provision of nutrients and/or mechanical inability to carry pregnancy to the full term will prevail.

Stunting begins early in utero and continues for at least the first 2 years of postnatal life; the period from conception to a child's second birthday has, therefore, been identified as the most crucial window of opportunity for intervention because this is found to be one of the most critical periods for linear growth. It is time stunting that becomes prevalent and peaks in developing countries due to the high demand for nutrients along with limited quality and quantity of complementary foods [9]. The complex interaction between maternal nutritional status, endocrine and metabolic signals, and placental development has a significant influence on foetal growth [10].

Evidence from past and recent studies have consistently shown that children born following shorter birth intervals have substantially higher rates of adverse pregnancy outcomes such as stillbirth, neonatal, postneonatal, and childhood mortality than those born after longer intervals [7, 11–14]. However, there is little information available on the extent to which birth spacing affects postnatal child growth especially in most low- and middle-income countries (LMICs) including Ghana, where poor child growth is a common occurrence. A review of evidence from research findings suggests the relationship between birth interval, and nutritional status is inconclusive as conflicting results have emerged and are also reported to have important methodological limitations and residual confounding (that is, factors such as breastfeeding and maternal height not included in the analysis) [15].

This study investigated whether the length of birth interval preceding the index child is associated with the risk of low birth weight and postnatal child growth while controlling for potentially confounding variables in the Upper East Region of Ghana.

## 2. Materials and Methods

### 2.1. Study Setting

The study was conducted in the Kassena-Nankana municipality (KNM) in the Upper East Region (UER) of Ghana. The estimated population of the KNM is 116,668 with a population density of 102 persons per square kilometre and a growth rate of 1.2%. Women in fertile age (WIFA) are estimated to be 28,008 with 4,668 expected deliveries while children aged 0–59 months constitute 21,590 [16]. The municipality consists of 99 communities which are predominantly rural; only 13% of the population live in towns. The settlement pattern of the population is dispersed. This settlement pattern has a profound effect on the distribution of social amenities and public goods, especially water and sanitation facilities [16]. Both curative and preventive health services are rendered by health facilities. There is 1 government hospital (War Memorial Hospital), 2 health centres (HC), and 25 community health planning and services (CHPS) facilities.

### 2.2. Study Design, Population, and Sampling Procedure

A retrospective population-based cohort of women who delivered live babies for the past 24 months were used to compare postnatal growth across different categories of birth intervals.

The specific inclusion and exclusion criteria for study participants were women resident in the study area and were attending the child welfare clinic (CWC). Additionally, eligible participants should have delivered at least two children including the current index child whose birth weights were adequately recorded by the health personnel in the child health record booklet. Excluded were twin or triplets.

Study participants were selected using a multistage sampling technique. All 28 health facilities rendering CWC services in the KNM constituted the sampling universe, out of which the sampling frame of health facilities rendering family planning and contraceptive services were constituted. In each facility, mother-child pairs who met the criteria for participating in the research were chosen using systematic random sampling.

### 2.3. Sample Size Determination

The sample size was determined based on two sample populations at 95% confidence level and an effect size of 8% reduction in low birth weight (LBW). The sample size for this study was calculated using the following formula:

(1) $$\begin{array}{c} n=\frac{D\left[{\left(Z_{\alpha }\;+\;Z_{\beta }\right)}^{2}\;\times \left(P_{1}\;\left(1\;-\;P_{1}\right)+\;P_{2}\;\left(1\;-\;P_{2}\right)\right)\right]\;}{{\left(P_{2}\;-\;P_{1}\right)}^{2}}, \end{array}$$

where *n* represents the required minimum sample size per study group, and *D* represents the design effect (assumed in the following equations to be the default value of 1.

The population proportion was derived from a study in the Kassena-Nankana district which reported a prevalence of 13.8% in LBW [17]. *P*_1_ is the prevalence of LBW in the nonexposed group (that is, women with a birth interval of at least 2 years). *P*_2_ is the prevalence of LBW in the exposed group (that is, women with birth interval of less than 2 years), such that the quantity (*P*_2_ − *P*_1_) is the size of the magnitude of change it is desired to be able to detect. When *Z*_*α*_ = 1.96 and *Z*_*β*_ = 0.842, assuming *P*_1_ = 5.8%, the prevalence of LBW in the control group (women with birth interval of at least 2 years) and *P*_2_ is estimated to be 13.8%, the expected prevalence of LBW in the exposed group (that is, women with birth interval of less than 2 years). Going by these assumptions, the estimated minimum sample size was 241 for each group, making a total sample size of 482, and making provision of 10% contingency, the sample size was adjusted to 530 (that is, 265 for each study group).

### 2.4. Data Collection Methods

The data were collected from the mothers/caregivers using a structured questionnaire, which was administered through a face-to-face interview at the household level. The data included sociodemographic and economic characteristics of the participants, parity, marital status, maternal educational level, age of mother and child, occupation, antenatal care attendance, household wealth index, child illness in the past two weeks, birth spacing, and child anthropometric measures.

### 2.5. Independent and Dependent Variables

The main dependent outcomes were the birth weight (a proxy measure of foetal growth) and length-for-age *z*-score (LAZ), which is a measure of postnatal child growth.

The main independent variable is the length of the preceding birth-to-birth interval, measured as the number of months between the date of birth of the child under study (index child) and the date of birth of the immediately preceding child to the mother. The exposed group, therefore, comprised mothers with short birth intervals and the nonexposed group were mothers with long birth intervals. A brief description of how the study variables were measured is described as follows.

### 2.6. Assessment of Birth Interval

Birth interval was defined as the length of time between two successive live births, rather than the interconception interval. The birth interval was dichotomized as short (<24 months) versus normal (≥24 months) based on the World Health Organization (WHO) recommended birth spacing (that is, birth-to-pregnancy intervals of at least 24 months or about three years between births [18].

### 2.7. Assessment of Postnatal Growth of Children

The child's growth rate (g/month) was calculated based on the following formula: growth rate = current weight (g)–birthweight (g)/age (months).

Child growth indicators including the length-for-age *z*-score (LAZ), weight-for-age *z*-score (WAZ), and weight-for-length *z*-score (WLZ) were calculated as recommended by the World Health Organization [19]. The LAZ index is an indicator of linear growth retardation and cumulative growth deficits. Children whose LAZ-scores were below minus two standard deviations (−2 SD) from the median of the reference population were considered short for their age (stunted) or chronically malnourished.

The WLZ index measures body mass in relation to body height or length and describes current nutritional status. Children whose *z*-scores were below −2 SD from the median of the reference population were considered thin (wasted) or acutely malnourished. Wasting represents the failure to receive adequate nutrition in the period immediately preceding the survey. It may result from inadequate food intake or a recent episode of illness causing a loss of weight and the onset of malnutrition. WAZ is a composite index of LAZ and WLZ. Children whose WAZ were below −2 SD from the median of the reference population were classified as underweight.

### 2.8. Data Analysis

Data cleaning and analysis were carried out using statistical weighted analysis in the SPSS complex samples module for windows 22.0 (SSPS Inc., Chicago, IL, USA). This was carried out to make statistically valid population inferences and compute standard errors from the sample data obtained from cluster sampling. The nutritional indicators of the child were computed using the Anthro Plus which converted anthropometric measures into *z*-scores. The various *z*-scores were transferred to the SPSS software for further analysis.

Bivariate and multivariable regression analyses were performed to identify to assess the degree of association between explanatory and selected dependent variables. Only variables that showed a significant association (*p* < 0.05) with each dependent variable in the bivariate analysis were selected and adjusted for in the multivariable regression analysis. Multicollinearity among the predictor variables was checked before their inclusion in the final regression model. The level of statistical significance was set at *p* less than 5% with 95% confidence intervals (CI).

### 2.9. Ethics Consideration

The study protocol was approved by the Ethics Committee, Institutional Review Board of Navrongo Health Research Centre (NHRC), Ghana. Informed consent was also obtained after needed information and explanation. In situations where the respondent could not write or read, verbal informed consent was sought from all study participants before the commencement of any interview.

## 3. Results

### 3.1. Sociodemographic Characteristics

In all, a total of 542 study participants were interviewed, but due to incomplete data and nonresponses, the data were trimmed to 530 valid responses constituting 97.8% response rate. The mean age of the children was 10.0 ± 6.4 months.

The two groups of women that were studied were those who delivered with birth interval less than two years and those with an interval two years and above.  Table 1 provides that the study groups were similar in sociodemographic characteristics except with regard to parity. Mothers who were secundiparous were significantly higher among women who had a birth interval less than 24 months.

### 3.2. Comparison of Child Growth Indicators according to Birth Interval of Mothers

The child's growth rate (g/month) was calculated based on the following formula: growth rate = current weight (g)–birth weight (g)/age (months). The mean child's growth rate/velocity was 0.669.0 ± 0.32 (g/month) among children aged 0–24 months.

Table 2 provides unadjusted differences in mean child growth indices according to birth interval using analysis of variance (ANOVA). The unadjusted values of the mean length-for-age *z*-score (LAZ), weight-for-age *z*-score (WAZ), and postnatal growth rate of children were negatively associated with birth interval, but weight-for-length *z*-score (WLZ) was not associated. Children with shorter birth intervals (<24 months) had better postnatal growth indicators, compared with their counterparts whose birth intervals were longer (that is, at least 24 months). Birth weight, however, associated positively with birth interval.

The unadjusted mean difference in LAZ was significantly higher among children of short preceding birth interval than among children of long birth interval (0.75 versus −0.29) (95% CI: 0.73–1.34), *p* < 0.001. Using the analysis of covariance (ANCOVA) that adjusted for age of the child, gender of the child, WLZ, birth weight, adequacy of ANC attendance, and dietary diversity of the child, there was still a significant difference in mean LAZ between the study groups (*F* (1, 529) = 11.79, *p* = 0.001), although the difference reduced to 0.55 (0.51 versus −0.04) (95% CI: 0.24–0.87), *p* = 0.001.

After controlling for age of the child, gender of the child, WLZ, and dietary diversity of the child, no significant difference in growth velocity (weight gained/month) was observed between the study groups.

In terms of birth weight, the long preceding birth interval was positively associated with birth weight. The adjusted mean birth weight of children born to mothers of longer birth interval was 74.0 g more than children born to mothers of shorter birth interval (CI: 5.89–142.0, *p* < 0.03).

### 3.3. Predictors of Length-for-Age *Z*-Score (LAZ)

To control for confounding, the following variables were included in the multivariable regression analysis: age and sex of the child, minimum dietary diversity, mother's educational level, mother's age at birth, birth interval, timing of first prenatal care visit, frequency of ANC attendance, birth weight, occupation of mother, maternal age, breastfeeding status, timing of first ANC attendance, household wealth index, and adequacy of ANC attendance and parity that were modelled to determine which factors predicted mean LAZ.

The results showed that the birth interval was a significant determinant of length-for-age *z*-score in this study population. The independent predictors of mean LAZ are given in Table 3. The consistent independent predictors of LAZ for children aged 0–24 months were birth interval, age of child, sex of child, weight-for-length *z*-score (WLZ), dietary diversity score (DDS), adequacy of antenatal care (ANC) attendance, and birth weight.

The adjusted mean difference in LAZ was significantly lower (beta = −0.12, *p* = 0.004), among children of long preceding birth interval than among children of the short birth interval. A unit increase in the age of the child leads to a reduction in LAZ by 0.33 standard units. Low birth weight born children had a lower LAZ compared to children born with birth weight of at least 2.5 kg. Mothers who had adequate antenatal care (ANC) attendance (that is, started in the first trimester and attended at least 4 times) had a higher LAZ (beta = 0.09, *p* = 0.02), compared to mothers who had inadequate ANC. Female children had a higher LAZ compared to their male counterparts (beta = 0.08, *p* = 0.03). A unit increase in the child dietary diversity score (DDS) was associated with an increase of 0.11 standard units in LAZ ((beta = 0.10, *p* = 0.04), while a unit increase in WLZ leads to a reduction in HAZ by 0.29 standard units.

## 4. Discussion

The aim of the present study was to assess the independent association of birth interval with birth weight and subsequent postnatal growth indices. We hypothesized that a short birth interval will produce children of low birth weight (<2.5 kg) and may influence postnatal growth. The findings of the current study showed that after controlling for potential confounders, a short birth interval is associated positively with an increased risk of low birth weight (an indicator of foetal growth), but birth spacing is associated negatively with LAZ (an indicator of postnatal growth).

### 4.1. The Association of Birth Intervals with Birth Weight

The World Health Organization (WHO) has defined birth interval as the period from live birth to a successive pregnancy, and the recommended period is at least 24 months (2 years) [20]. In this study, the long preceding birth interval was positively associated with birth weight. This may be partly explained by the fact that adequate birth spacing might allow women to recover and be healthy for their next pregnancy. Therefore, if a pregnancy occurs too soon after the previous birth, the mother may not have recovered her nutritional status, a situation that leads to what is described as “maternal depletion syndrome” [15]. With an adequate birth interval, the mother will be able to replenish her nutrient stores which are essential for foetal growth [13, 21]. The positive relationship between long birth interval and birth weight found in the present study is congruent with the findings of earlier studies [22–27].

The growth of children in different stages of life has been reported to be related in one way or the other. For example, it has been reported that gains in the *z*-score during the first 8 weeks of life are strongly and positively associated with attained *z*-scores at 12 months [28]. This may partly explain the negative association of LBW with postnatal growth.

### 4.2. The Association between Birth Interval and Postnatal Growth Indices

One of the main findings of this study is that a short birth interval was associated with a decreased risk of poor postnatal child growth as measured by length-for-age *z*-score (LAZ), but birth spacing was unrelated to growth velocity as measured by weight gained/month and weight-for-length *z*-score (WLZ).

Although long birth intervals have been positively linked to postnatal child growth outcomes in previous studies, this study observed a significant negative association after controlling for key confounders. It is not clear why children with short birth intervals in our sample significantly had high mean length-for-age *z*-score (LAZ) values, compared with their counterparts whose birth intervals were at least 24 months?

Some researchers do suggest that birth spacing might influence childhood undernutrition through its association with preterm birth and low birth weight (LBW). The mechanism is considered related to the “maternal depletion syndrome” which postulates that the woman may not have fully recuperated from one pregnancy before supporting the next one [13, 15]. The understanding here is that short birth spacing leads to poor perinatal outcomes including LBW, which then exposes children to childhood stunting. We observed in our study population that a short birth interval is not only associated positively with LBW babies but also associated with the decreased risk of childhood stunting. Therefore, this hypothesized mechanism does not appear to hold wholly in our study population. The implication could be that good postnatal socioeconomic conditions and other biological factors may interact, such that short interval children who are likely to have foetal growth restriction have a postnatal growth advantage over long interval children. Most infants who are born small for gestational age (SGA) achieve a normal height following a period of catch-up growth before the age of 2 years [29], and this accelerated growth can lead to a weight or height within 2 standard deviations for age. For example, one study has reported that after a period of intrauterine growth restriction, postnatal growth can take two forms, accelerated growth (catch-up) or growth at a normal postnatal rate (no catch-up) [30]. Accelerated catch-up linear growth reduces, or possibly even erases, a child's early-life height deficit. This type of catch-up pattern is described to be a transient increase in growth velocity after cessation of the growth restriction, followed by normal a growth velocity when the original growth curve is achieved [31].

Catch-up growth represents an interaction of the child's biological growth potential with environmental factors that enhance growth, among which optimal nutrition and low morbidity are the most important. Stunted children at birth with improvements in socioeconomic conditions and diet in later childhood show catch-up in linear growth to more closely approximate their growth potential [32].

There is currently an ongoing debate in the literature around the persistence of early childhood growth faltering and the potential for later-life catch-up growth. While some researchers argue that complete catch-up growth, even in the presence of later-life compensatory investments or behavior, is unlikely [33–36], others have found evidence of complete catch-up growth among children who experienced early-life stunting and nutritional deficits [32, 37].

Postnatal child growth failure is controlled by a multitude of biological and environmental factors including exposure of the foetus and/or young child to nutritional deficiencies and infectious diseases. It is worthy to note that postnatal growth consists of three distinct phases: infancy, childhood, and puberty [38], which are influenced by different growth factors. Infant growth is largely a continuation of intrauterine growth that is influenced predominantly by maternal nutrition and is largely growth hormone independent [39]. Childhood growth, on the other hand, is largely dependent on growth hormone-dependent [40] such as the insulin-like growth factor-1.

Apart from the foregoing explanations for what might account for the observed association between short birth spacing and postnatal child growth outcomes in this study, it could also be driven by a wide range of confounding factors that were not measured.

The relationship between birth interval and postnatal growth appears inconclusive because there are inconsistent findings regarding this relationship. While past studies have reported that longer birth intervals have great potential in reducing malnutrition in children, others have been negative, and some showed no effect at all [15]. Earlier studies in Kenya and Nigeria did not find a significant relationship between preceding birth interval and growth of the child [41, 42].

In one earlier systematic review that used data from 17 Demographic and Health Surveys (DHS), it was concluded that the effects of short birth intervals on nutritional status were rather moderate, and there is a weak relationship with lower attendance at prenatal care services [43]. Another study carried out in El Salvador concluded that birth spacing plays an important role in nutritional status among children under 5 years of age, with shorter birth intervals increasing the risk of both stunting and underweight [44]. Analysis from the survey shows that a child born after an interval of less than 24 months is 1.52 times more likely to be stunted, compared with an interval of 36–59 months.

In four low- and middle-income countries including Brazil, Guatemala, Philippines, and South Africa, it found increased absolute height and relative HAZ scores and lower stunting odds among children who were more widely spaced compared to children who were more narrowly spaced (<2years) [45]. A systematic review of the available evidence from 52 studies confirms that birth intervals can affect nutritional outcomes, although the degree to which the relationship holds varies substantially [15]. In that review, half of the studies found that a previous birth interval of at least 36 months was associated with a 10–50% reduction in childhood stunting, whereas the remaining studies found no association or were inconclusive. In another review involving 153 Demographic and Health Surveys (DHS) across 61 countries, it was found that birth intervals of less than 12 months and between 12 and 23 months were associated with higher risk for stunting as compared to a 24–35-month interpregnancy interval [46].

### 4.3. Determinants of LAZ

Stunting prevalence (that is, low length/height for age) is a key indicator to evaluate a country's progress towards United Nations' Sustainable Development Goal 2, which is to end hunger and achieve improved nutrition by 2030. Therefore, a better understanding of the factors that contribute to early linear growth faltering is necessary to accelerate progress towards reducing the prevalence of stunting. The consistent independent predictors of LAZ for children aged 0–24 months were birth interval, age of child, sex of the child, weight-for-height *z*-score (WHZ), dietary diversity score (DDS), antenatal care (ANC) attendance, and birth weight.

Low birth weight, which is an outcome of foetal growth failure, was an independent determinant of postnatal growth as measured by height-for-age *z*-score, since LBW children had a significantly lower mean LAZ compared to children born with an appropriate birth weight of at least 2.5 kg. Put in another way, it means birth weight was positively related to postnatal linear growth during childhood. The strong and positive association of the child's birth weight with LAZ is in agreement with previous studies [30, 47]. This association is supported by the fact that infant growth is largely a continuation of in utero growth, which is largely influenced by maternal nutrition and growth hormones [39]. In Guatemala, similar findings were reported, in which poorer growth and development were observed among stunted LBW newborns [48]. However, in Sweden, an excellent catch-up of stunted newborns has been reported [49]. This has been interpreted to imply that good postnatal socioeconomic conditions may overcome foetal insults.

Consistent with other studies, more older children had lower mean LAZ (that is, stunted) than younger ones in our study sample [44, 50, 51]. Female children had a higher LAZ compared to their male counterparts and this which concurs very well with the findings from sixteen Demographic and Health Surveys from ten sub-Saharan countries [52]. The hypothesized mechanism that explains why boys are more vulnerable to stunting than girls is that it occurs already during pregnancy, with sex differences in foetal growth [53, 54]. The sex of the child is reported to play a crucial role in postnatal growth, whereby boys and girls differ in the rate of physical and structural growth and development [30].

Adequate prenatal care utilization was positively associated with length-for-age in this study sample. Mothers who had adequate antenatal care (ANC) attendance had a higher LAZ, compared to mothers who had inadequate ANC. This finding is in consonance with studies conducted in El Salvador, four low- and middle-income countries (Brazil, Guatemala, Philippines, and South Africa) [44, 45].

## 5. Conclusions

The results suggest that a short birth interval is associated positively with an increased risk of low birth weight (an indicator of foetal growth), but birth spacing is associated negatively with LAZ (an indicator of postnatal growth). Birth spacing was unrelated to growth velocity as measured by weight gained/month and weight-for-length *z*-score (WLZ). The causal relationship between low birth spacing is likely to be context specific and therefore requires further research.

## Figures and Tables

**Table 1:** Comparison of sociodemographic characteristics by study groups (*N* = 530).

Variable	*N*	Study groups		Test statistic chi-squared (χ^2^)
		Birth interval <24 months	Birth interval ≥24 months	
		*n* (%)	*n* (%)	
Age of mother (years)				
Under 25	120	63(52.5)	57 (47.5)	χ^2^ = 0.39, *p* = 0.82
25–34	297	146 (49.2)	151 (50.8)	
35+	113	56 (49.6)	58 (50.4)	

Maternal educational level				
None	175	84 (48.0)	91 (52.0)	χ^*2*^ = 0.92, *p* = 0.63
Low (primary and JHS)	271	141 (52.0)	130 (48.0)	
High (at least SHS)	84	40 (47.6)	44 (52.4)	

Marital status				
Married	509	251 (49.3)	258 (50.7)	χ^*2*^ = 2.43, *p* = 0.12
Single	21	14 (66.7)	7 (33.3)	

Religion				
Islam	78	41 (52.6)	37 (47.4)	χ^*2*^ = 0.24, *p* = 0.62
Christianity	452	224 (49.6)	228 (50.4)	

Trimester of first ANC visit				
First	455	225 (49.5)	230 (50.5)	χ^*2*^ = 0.39, *p* = 0.53
Second	75	40 (53.3)	35 (46.7)	

Frequency of ANC visits				
Less than 4	40	23 (57.5)	17 (42.5)	χ^*2*^ = 0.97, *p*= 0.32
At least 4	490	242 (49.4)	248 (50.6)	

Classification of parity				
Primiparous	12	7 (58.3)	4 (41.7)	χ^*2*^ = 7.02, *p* = 0.03
Secundiparous	206	117 (56.8)	89 (43.2)	
Multiparous	312	141 (54.2)	171 (54.8)	

Classification of wealth index				
Low	327	177 (54.1)	150 (45.9)	χ^*2*^ = 0.29, *p*= 0.59
High	203	105 (51.7)	98 (48.3)	

**Table 2:** Comparison of child growth indices according to the birth interval (unadjusted differences).

Indicator	Birth interval	*N*	Mean	95% confidence interval for mean			Test statistics
				Std. deviation	Lower bound	Upper bound	
HAZ	<24	265	0.75	1.85	0.53	0.97	*F* (1, 529) = 45.37, *p* < 0.001
	At least 24	265	−0.29	1.69	−0.49	−0.08	

WHZ	<24	265	−0.63	1.64	−0.83	−0.43	*F* (1, 529) = 0.98, *p* = 0.32
	At least 24	265	−0.49	1.54	−0.68	−0.31	

WAZ	<24	265	−0.14	1.31	−0.30	0.02	*F* (1, 529) = 12.68, *p* < 0.001
	At least 24	265	−0.53	1.19	−0.67	−0.38	

Birthweight (g)	<24	265	2919.7	380.6	2873.7	2965.7	*F* (1, 529) = 4.56, *p* = 0.03
	At least 24	265	2993.7	416.7	2943.3	3044.1	

Postnatal growth rate of child in grams/month	<24	265	765.5	307.3	728.3	802.6	*F* (1, 529) = 53.08, *p* ≤ 0.001
	At least 24	265	572.7	301.9	536.2	609.2	

**Table 3:** Predictors of the length-for-age *z*-score (multivariable regression analysis).

Model	Standardized coefficients	*t*	Sig.	95.0% confidence interval for B		Collinearity statistics	
	Beta			Lower bound	Upper bound	Tolerance	VIF
Constant		0.708	0.480	−0.38	0.80		
Female child	0.08	2.14	0.033	0.024	0.58	0.983	1.017
Age of index child	−0.33	−6.66	<0.001	−0.12	−0.07	0.600	1.667
Dietary diversity score	0.10	2.10	0.04	0.005	0.16	0.702	1.425
Low birth weight	−0.12	−3.13	0.002	−1.31	−0.30	0.988	1.013
Adequate ANC attendance	0.09	2.38	0.02	0.08	0.80	0.990	1.010
WHZ	−0.29	−7.46	<0.001	−0.42	−0.25	0.981	1.019
Birth interval ≥24 months	−0.12	−2.91	0.004	−0.76	−0.15	0.798	1.253

## Data Availability

The quantitative data presented in the form of SPSS used to support the findings of this study are included within the supplementary information file.

## Additional Points

Recall bias could not be ruled out as the study design was a retrospective study; women had to remember before they could respond to questions. Infant growth has been shown to be highly heterogeneous due to several variables that were not all measured in the present study. Nevertheless, the study contributes to the body of knowledge as it has shed some light on the effect birth intervals can have on foetal and postnatal growth.

## Supplementary Materials

Supplementary Materials The dataset on the effect of birth interval on foetal and postnatal child growth is provided.
