# Supplementary material for: Effect of Birth Interval on Foetal and Postnatal Child Growth
Source: Scientifica (Cairo). 2021 Aug 20;2021:6624184. doi: 10.1155/2021/6624184 (PMC8405331; doi:10.1155/2021/6624184)
Supplement: Supplementary Materials — The dataset on the effect of birth interval on foetal and postnatal child growth is provided. [file 6624184.f1.pdf]

Group	Group2	SubMunici	Community	Faciltiy	Interviewe	Date	ID	Duration
2.00	1.00	7	vunania	vunania ch	7	8-Nov-17	1	26.00
2.00	0.00	4	pungu	pungu nort	4	8-Nov-17	2	30.00
1.00	0.00	7	vunania vunania chc		8	8-Nov-17	3	12.00
1.00	0.00	4	biwiu	pungu nort	1	8-Nov-17	4	14.00
2.00	1.00	4	pungu	pungu s. CH	3	8-Nov-17	5	24.00
2.00	1.00	1	Akurugu Da	KN CHC	4	9-Nov-17	6	24.00
1.00	0.00	1	navrongo	W. memori	4	8-Nov-17	6	15.00
1.00	0.00	1	navrongo	War memo	4	8-Nov-17	7	11.00
2.00	1.00	4	pungu	pungu sout	4	8-Nov-17	8	24.00
1.00	1.00	7	vunania	vunani CHC	7	8-Nov-17	9	24.00
1.00	0.00	7	pungu	pungu sout	4	8-Nov-17	10	10.00
2.00	1.00	4	Telania	pungu S. CH	3	8-Nov-17	11	24.00
1.00	0.00	4	pungu	pungu nort	3	8-Nov-17	12	15.00
1.00	0.00	4	pungu	pungu nort	4	8-Nov-17	13	24.00
2.00	1.00	4	pungu	pungu nort	4	8-Nov-17	14	24.00
1.00	0.00	4	pungu	pungu nort	4	8-Nov-17	15	11.00
2.00	1.00	4	pungu	pungu nort	4	8-Nov-17	16	24.00
1.00	1.00	4	pungu	pungu nort	3	8-Nov-17	17	12.00
1.00	0.00	4	pungu	pungu	4	9-Nov-17	18	16.00
2.00	1.00	4	pungu	pungu noet	3	8-Nov-17	19	24.00
2.00	1.00	4	pungu	pungu nort	3	8-Nov-17	20	26.00
1.00	0.00	4	pungu	pungu nort	3	8-Nov-17	21	8.00
2.00	1.00	4	pungu	pungu nort	3	8-Nov-17	22	27.00
1.00	0.00	1	navrongo	WMH	6	8-Nov-17	23	13.00
2.00	1.00	1	vavrongo	WMH	12	9-Nov-17	24	14.00
2.00	1.00	1	akurugu da	KN CHC	4	9-Nov-17	25	25.00
2.00	1.00	1	akurugu da	KN CHC	4	9-Nov-17	27	24.00
1.00	1.00	2	akurud dab	akurug dab	3	9-Nov-17	28	10.00
2.00	1.00	2	Akurugu da	KN CHc	2	9-Nov-17	29	36.00
1.00	1.00	2	Ak dabo	KNCHC	3	9-Nov-17	30	24.00
1.00	0.00	2	Ak dabo	KN Chc	9	9-Nov-17	31	12.00
2.00	0.00	2	Ak bado	KN CHC	9	9-Nov-19	32	15.00
2.00	1.00	2	Ak Dabo	KN CHC	9	9-Nov-17	33	27.00
2.00	0.00	2	Ak dabo	KNE CHC	3	9-Nov-17	34	25.00
1.00	0.00	2	Ak dabo	KNE cHC	3	9-Nov-17	35	13.00
1.00	0.00	7	vunania	vunania CH	8	9-Nov-17	36	8.00
2.00	0.00	7	vunania	vunania CH	8	9-Nov-17	37	29.00
1.00	0.00	7	vunania	vunania CH	8	9-Nov-17	38	14.00
1.00	0.00	7	vunania	vunania CH	8	9-Nov-17	39	11.00
1.00	0.00	7	vunania	vunania CH	8	9-Nov-17	40	12.00
2.00	1.00	7	vunanai	vunania CH	8	9-Nov-17	41	26.00
1.00	0.00	7	vunania	vunania CH	8	9-Nov-17	42	9.00
2.00	0.00	7	vunania	vunania ch	8	9-Nov-17	43	12.00
2.00	1.00	2	AK Dabo	KN CHC	4	9-Nov-17	44	24.00
2.00	1.00	2	AK dabo	KN CHC	9	9-Nov-17	45	30.00
2.00	1.00	2	AK dabo	KN CHC	4	9-Nov-17	46	24.00

2.00	0.00	2	Akrugu Dar	Akrugu Dal	9	9-Nov-17	46	30.00
1.00	1.00	2	Ak dabo	KN CHC	4	9-Nov-17	47	13.00
2.00	1.00	2	Ah Dabo	KN CHC	4	9-Nov-17	48	24.00
2.00	1.00	2	Ak dabo	KNC CHC	4	9-Nov-17	49	24.00
1.00	0.00	2	Ak Dabo	KN CHC	9	9-Nov-17	50	11.00
2.00	1.00	2	Akrugu Dal	Akrugu Dal	3	9-Nov-17	53	24.00
1.00	0.00	3	gumongo	gumongo c	3	10-Nov-17	54	10.00
2.00	0.00	2	natugnia	natugnia ch	4	10-Nov-17	81	12.00
2.00	1.00	3	sabisi	natugnia	4	10-Nov-17	82	25.00
1.00	0.00	3	natugnia	natugnia	4	10-Nov-17	83	13.00
1.00	0.00	3	sabisi	natugnia	4	10-Nov-17	84	14.00
2.00	0.00	3	sabisi	natugnia	3	10-Nov-17	85	13.00
2.00	1.00	3	sabisi	natugnia	4	10-Nov-17	86	26.00
2.00	0.00	3	sabisi	natugnia	2	10-Nov-17	87	30.00
1.00	0.00	3	sabisi	natugnia	3	10-Nov-17	88	7.00
2.00	0.00	3	sabisi	natugnia	3	10-Nov-17	89	27.00
1.00	0.00	3	sabisi	natugnia	3	10-Nov-17	90	11.00
2.00	0.00	3	sabisi	natugnia	3	10-Nov-17	91	24.00
2.00	1.00	3	sabisi	natugnia	3	10-Nov-17	92	26.00
1.00	0.00	3	sabisi	natugnia	3	10-Nov-17	93	15.00
2.00	0.00	3	sabisi	natugnia	6	10-Nov-17	94	26.00
2.00	0.00	3	sabisi	natugnia	6	10-Nov-17	95	24.00
1.00	0.00	3	sabisi	natugnia	6	10-Nov-17	96	12.00
2.00	1.00	3	sabisi	natugnia	6	10-Nov-17	97	24.00
1.00	0.00	3	sabisi	natugnia	6	10-Nov-17	98	10.00
1.00	0.00	2	natugnia	natugnia	4	10-Nov-17	99	14.00
2.00	0.00	2	natugnia	natugnia ch	4	10-Nov-17	100	24.00
2.00	0.00	2	natugnia	natugnia ch	4	10-Nov-17	101	21.00
1.00	0.00	2	natugnia	natugnia ch	4	10-Nov-17	102	13.00
2.00	1.00	7	gani	vunania	7	10-Nov-17	103	18.00
1.00	0.00	7	gani	vunania	7	10-Nov-17	104	12.00
2.00	0.00	7	gani	vunania	7	10-Nov-17	105	26.00
1.00	0.00	7	gani	vunania	7	10-Nov-17	106	13.00
2.00	1.00	7	gani	vunania	7	10-Nov-17	107	28.00
2.00	1.00	7	gani	vunania	7	10-Nov-17	108	30.00
1.00	0.00	7	gani	vunania	7	10-Nov-17	109	10.00
2.00	0.00	7	gani	vunania	7	10-Nov-17	110	24.00
2.00	1.00	7	vunania	vunania ch	7	10-Nov-17	111	24.00
1.00	0.00	2	natugnia	natiugnia c	4	10-Nov-17	112	12.00
2.00	0.00	2	natugnia na	natugnia ch	4	10-Nov-17	113	24.00
2.00	1.00	2	natugnia	natugnia ch	4	10-Nov-17	114	24.00
1.00	0.00	2	natugnia	natugnia ch	4	10-Nov-17	115	13.00
2.00	1.00	2	natugnia	natugnia ch	4	10-Nov-17	116	24.00
1.00	0.00	2	natugnia	natugnia ch	4	10-Nov-17	117	9.00
2.00	1.00	2	natugnia	natugnia ch	4	10-Nov-17	118	24.00
1.00	0.00	2	natugnia	natugnia ch	4	10-Nov-17	119	31.00
1.00	0.00	3	natugnia	natugnia ch	5	10-Nov-17	120	14.00

2.00	1.00	3	natugnia	natugnia ch	5	10-Nov-17	121	20.00
1.00	0.00	3	natugnia	natugnia ch	5	#NULL!	122	7.00
2.00	1.00	3	Natugnia	Natugnia C	5	10-Nov-17	123	25.00
1.00	0.00	3	Sabisi	Natugnia	4	10-Nov-17	124	11.00
2.00	0.00	3	Sabisi	Natugnia	4	10-Nov-17	124	24.00
1.00	0.00	3	sabisi	Natugnia	4	10-Nov-17	125	12.00
2.00	1.00	3	Sabisi	Natugnia C	4	10-Nov-17	126	30.00
1.00	0.00	3	Sabisi	Natugnia C	4	10-Nov-17	127	7.00
2.00	1.00	3	Sabisi	Natugnia C	4	10-Nov-17	128	24.00
1.00	0.00	3	Sabisi	Natugnia C	4	10-Nov-17	129	24.00
2.00	1.00	3	Sabisi	Natugnia C	4	10-Nov-17	130	30.00
1.00	0.00	3	Sabisi	Natugnia C	4	10-Nov-17	131	11.00
2.00	1.00	3	Sabisi	Natugnia C	4	10-Nov-17	132	30.00
2.00	1.00	3	Sabisi	Natugnia C	4	10-Nov-17	133	28.00
2.00	1.00	3	Gumongo	Gumongo (	5	10-Nov-17	134	26.00
1.00	0.00	3	Gumongo	Gumongo (	5	10-Nov-17	135	10.00
1.00	0.00	3	Gumongo	Gumongo (	3	10-Nov-17	136	8.00
1.00	0.00	3	Sabisi	Natugnia C	5	10-Nov-17	137	13.00
2.00	1.00	3	Sabisi	Natugnia C	5	10-Nov-17	138	24.00
1.00	0.00	3	Sabisi	Natugnia C	5	10-Nov-17	139	10.00
2.00	0.00	3	Gumongo	Gumongo (	2	10-Nov-17	140	24.00
2.00	1.00	3	Gumongo	Gumongo (	3	10-Nov-17	141	24.00
1.00	0.00	3	Gumongo	Gumongo (	3	10-Nov-17	142	12.00
2.00	1.00	3	Gumongo	Gumongo (	2	10-Nov-17	143	25.00
2.00	1.00	3	Natugnia	Natugnia C	5	10-Nov-17	144	34.00
1.00	0.00	3	Sabisi	Natugnia C	5	10-Nov-17	145	11.00
2.00	0.00	3	Sabisi	Natugnia C	5	10-Nov-17	146	17.00
2.00	0.00	3	Sabisi	Natugnia C	5	10-Nov-17	147	30.00
1.00	0.00	3	Sabisi	Natugnia C	5	10-Nov-17	148	14.00
2.00	1.00	3	Sabisi	Natugnia C	5	10-Nov-17	149	24.00
1.00	0.00	3	Sabisi	Natugnia C	5	10-Nov-17	150	9.00
1.00	0.00	3	Natugnia	Natugnia C	5	10-Nov-17	151	14.00
2.00	1.00	3	Sabisi	NatugniaCh	5	10-Nov-17	152	30.00
1.00	0.00	3	Sabisi	Natugnia C	5	10-Nov-17	153	12.00
2.00	1.00	3	Natugnia	Natugnia C	4	10-Nov-17	154	24.00
1.00	0.00	3	Natugnia	Natugnia C	5	10-Nov-17	155	27.00
1.00	0.00	3	Natugnia	Natugnia C	5	10-Nov-17	156	12.00
2.00	1.00	3	Natugnia	Natugnia C	5	10-Nov-17	157	24.00
1.00	0.00	3	Natugnia	Natugnia C	5	10-Nov-17	158	13.00
2.00	0.00	3	Natugnia	Natugnia C	5	10-Nov-17	159	24.00
1.00	0.00	3	Natugnia	Natugnia C	5	10-Nov-17	160	7.00
2.00	1.00	3	Natugnia	Natugnia C	5	10-Nov-17	161	24.00
1.00	0.00	3	Natugnia	Natugnia C	4	10-Nov-17	162	16.00
1.00	0.00	3	Natugnia	Natugnia C	4	10-Nov-17	163	14.00
2.00	0.00	3	Natugniia	Natugnia C	4	10-Nov-17	164	24.00
1.00	0.00	3	Natugnia	Natugnia C	4	10-Nov-17	165	12.00
1.00	0.00	3	Natugnia	Natugnia C	4	10-Nov-17	166	10.00

2.00	1.00	3	Natugnia	Natugnia C	4	10-Nov-17	167	24.00
1.00	0.00	1	Namolo	NHC	11	10-Nov-17	168	11.00
2.00	0.00	1	namolo	NHC	11	10-Nov-17	169	25.00
2.00	0.00	1	namolo	NHC	11	10-Nov-17	170	18.00
1.00	1.00	1	namolo	NHC	11	10-Nov-17	171	12.00
1.00	0.00	1	namolo	NHC	11	10-Nov-17	172	15.00
1.00	0.00	1	namolo	NHC	11	10-Nov-17	173	16.00
1.00	1.00	1	namolo	NHC	10	10-Nov-17	174	12.00
1.00	0.00	1	namolo	NHC	10	10-Nov-17	175	6.00
2.00	0.00	1	namolo	NHC	10	10-Nov-17	176	24.00
1.00	0.00	1	namolo	NHC	10	10-Nov-17	177	15.00
2.00	1.00	1	namolo	NHC	10	10-Nov-17	178	24.00
2.00	0.00	1	namolo	NHC	10	10-Nov-17	179	20.00
1.00	0.00	7	vunania	Vunania CH	7	10-Nov-17	180	9.00
2.00	1.00	7	gaani	Vunania CI	7	10-Nov-17	181	29.00
1.00	0.00	7	vunania	Vunania CH	7	10-Nov-17	182	24.00
2.00	0.00	7	gaani	Vunania CH	7	10-Nov-17	183	26.00
1.00	0.00	7	gaani	Vunania CH	7	10-Nov-17	184	12.00
2.00	1.00	7	Gaani	Vunania CH	7	10-Nov-17	185	18.00
1.00	0.00	7	Gaani	Vunania CH	7	10-Nov-17	186	11.00
2.00	0.00	7	Gaani	Vunania CH	7	10-Nov-17	187	24.00
1.00	0.00	7	Gaani	Vunania	7	10-Nov-17	188	10.00
1.00	0.00	7	Gaani	Vunania CH	7	10-Nov-17	189	14.00
2.00	1.00	7	Gaani	Vunania CH	7	10-Nov-17	190	26.00
2.00	1.00	7	Gaani	Vunania CH	7	10-Nov-17	191	36.00
1.00	0.00	7	Gaani	Vunania CH	7	10-Nov-17	192	13.00
2.00	1.00	7	Gaani	Vunania CH	7	10-Nov-17	193	27.00
1.00	0.00	7	Gaani	Vunania CH	7	10-Nov-17	194	14.00
2.00	1.00	7	Gaani	Vunania CH	7	10-Nov-17	195	24.00
1.00	0.00	3	Gumongo	Gumongo (	2	10-Nov-17	196	11.00
2.00	1.00	3	Gumongo	Gumongo (	3	10-Nov-17	197	28.00
1.00	0.00	3	Gumongo	Gumongo (	3	10-Nov-17	198	12.00
2.00	1.00	3	Gumongo	Gumongo (	3	10-Nov-17	199	28.00
2.00	1.00	3	Gumongo	Gumongo (	2	10-Nov-17	200	24.00
1.00	0.00	3	Gumongo	Gumongo (	3	10-Nov-17	201	14.00
1.00	0.00	3	Gumongo	Gumongo (	3	10-Nov-17	202	24.00
1.00	0.00	3	Gumongo	Gumongo (	3	10-Nov-17	203	9.00
2.00	0.00	3	Gumongo	Gumongo (	3	10-Nov-17	204	18.00
1.00	0.00	3	Gumongo	Gumongo (	3	10-Nov-17	206	15.00
2.00	1.00	3	Gumongo	Gumongo (	2	10-Nov-17	207	24.00
1.00	0.00	3	Gumongo	Gumongo (	2	10-Nov-17	208	14.00
2.00	0.00	3	Gumongo	Gumongo (	2	10-Nov-17	209	24.00
1.00	0.00	3	Gumongo	Gumongo (	2	10-Nov-17	210	8.00
1.00	0.00	3	Gumongo	Gumongo	2	10-Nov-17	211	11.00
2.00	1.00	#NULL!	Gumongo	Gumongo (	2	10-Nov-17	212	36.00
2.00	0.00	3	Gumongo	Gumongo (	3	10-Nov-17	213	28.00
1.00	0.00	3	Gumongo	Gumongo (	3	10-Nov-17	214	11.00

2.00	0.00	3	Gumongo	Gumongo (	4	10-Nov-17	215	24.00
1.00	0.00	7	Janania	Vunania CI	4	13-Nov-17	215	13.00
1.00	0.00	7	Janania	Vunania CH	4	13-Nov-17	216	10.00
1.00	0.00	7	Janania	Vunania CH	4	13-Nov-17	217	9.00
2.00	1.00	7	Janania	Vunania	4	13-Nov-17	218	24.00
2.00	0.00	7	Janania	Vunania	4	13-Nov-17	219	18.00
1.00	0.00	3	Benyim	Manyoro C	3	13-Nov-17	221	13.00
2.00	1.00	3	Benyim	Manyoro C	3	13-Nov-17	222	26.00
2.00	1.00	3	Benyim	Manyoro C	3	13-Nov-17	223	27.00
1.00	0.00	3	Benyim	Manyoro C	3	13-Nov-17	224	10.00
2.00	1.00	3	Benyim	Manyoro C	3	13-Nov-17	225	17.00
1.00	0.00	3	Benyim	Manyoro	3	13-Nov-17	226	12.00
2.00	1.00	3	Benyim	Manyoro C	3	13-Nov-17	227	24.00
1.00	0.00	3	Benyim	Manyoro C	3	13-Nov-17	228	12.00
2.00	0.00	3	Benyim	Manyoro C	3	13-Nov-17	229	24.00
2.00	1.00	3	Benyim	ManyoroCH	3	13-Nov-17	230	20.00
1.00	1.00	3	manyoro	Manyoro	3	13-Nov-17	231	13.00
2.00	1.00	5	gia	gia chc	9	14-Nov-17	232	24.00
1.00	1.00	3	Kapalla	MANYORO	2	13-Nov-17	244	12.00
2.00	1.00	7	Janania	Vunania ch	6	13-Nov-17	245	26.00
2.00	1.00	3	Sakonia	Manyoro C	1	13-Nov-17	246	24.00
1.00	1.00	3	Manyoro	Manyoro C	3	13-Nov-17	247	15.00
2.00	1.00	3	Sakonia	Manyoro C	3	13-Nov-17	248	26.00
1.00	0.00	3	Kupella	Manyoro C	2	13-Nov-17	249	11.00
2.00	0.00	1	Navrongo	Navrongo H	10	13-Nov-17	250	18.00
1.00	0.00	1	Navrongo	Navrongo H	10	13-Nov-17	251	12.00
1.00	0.00	1	Navrongo	Navrongo H	10	13-Nov-17	252	10.00
2.00	1.00	1	Navrongo	Navrongo H	11	13-Nov-17	253	24.00
1.00	0.00	1	Navrongo	Navrongo H	11	13-Nov-17	254	11.00
2.00	1.00	1	Navrongo	Navrongo H	11	13-Nov-17	255	26.00
1.00	0.00	1	Navrongo	Navrongo H	10	16-Nov-17	256	14.00
2.00	1.00	1	Navrongo	Navrongo H	11	13-Nov-17	257	19.00
1.00	0.00	7	Janania	Vunania CH	9	13-Nov-17	258	26.00
1.00	0.00	7	Jaanania	Vunania CH	9	13-Nov-17	259	10.00
2.00	1.00	7	Jaanania	Vunania CH	5	13-Nov-17	260	24.00
1.00	0.00	7	Jaanania	Vunania CH	5	13-Nov-17	261	13.00
2.00	1.00	7	Jaanania	Vunania CH	4	13-Nov-17	262	16.00
2.00	1.00	3	Dambisi	Manyoro cl	1	13-Nov-17	263	24.00
1.00	0.00	3	Dambisi	Manyoro C	3	13-Nov-17	264	12.00
1.00	0.00	3	Dambisi	Manyoro C	3	13-Nov-17	265	10.00
2.00	1.00	6	zoongo	zoongo chc	6	16-Nov-17	266	29.00
1.00	0.00	3	Dambisi	Manyoro C	3	13-Nov-17	266	28.00
1.00	0.00	3	Dambisi	Manyoro C	3	13-Nov-17	267	14.00
2.00	1.00	3	Dambisi	Manyoro C	3	13-Nov-17	268	24.00
1.00	0.00	3	Dambisi	Manyoro C	3	13-Nov-17	269	11.00
2.00	1.00	3	Gwari	Manyoro C	1	13-Nov-17	270	16.00
2.00	1.00	3	Wanjania	Manyoro C	1	13-Nov-17	271	37.00

1.00	0.00	3	Kupella	Manyoro C	1	13-Nov-17	272	10.00
1.00	0.00	3	Dambisi	Manyoro C	1	13-Nov-17	273	13.00
1.00	0.00	3	Wanjania	Manyoro cl	1	13-Nov-17	274	24.00
2.00	1.00	3	Manyoro	Manyoro C	3	13-Nov-17	275	25.00
1.00	0.00	3	Dambisi	Manyoro C	1	13-Nov-17	276	12.00
2.00	1.00	3	Wanjania	Manyoro cl	1	13-Nov-17	277	25.00
2.00	1.00	3	Kupela	Manyoro	1	13-Nov-17	278	30.00
1.00	0.00	3	Benyim	Manyoro C	1	13-Nov-17	279	12.00
2.00	1.00	3	Sakonia	Manyoro (	3	13-Nov-17	280	26.00
1.00	0.00	1	Navrongo	Navrongo H	10	13-Nov-17	281	12.00
2.00	1.00	1	Navrongo	Navrongo H	10	13-Nov-17	282	24.00
1.00	0.00	1	Navrongo	Navrongo H	10	13-Nov-17	283	9.00
2.00	1.00	1	N avrongo`	Navrongo H	10	13-Nov-17	284	19.00
1.00	0.00	3	Dambisi	Manyoro C	1	13-Nov-17	285	10.00
1.00	0.00	3	Benyim	Manyoro C	1	13-Nov-17	286	8.00
2.00	1.00	3	Wanjania	Manyoro C	1	13-Nov-17	287	24.00
1.00	0.00	3	Benyim Ma	Manyoro C	1	13-Nov-17	288	14.00
2.00	1.00	3	Kupela	Manyoro C	1	13-Nov-17	289	36.00
1.00	0.00	3	Kupela	Manyoro C	3	13-Nov-17	290	14.00
2.00	1.00	3	Kupela	Manyoro C	3	13-Nov-17	291	36.00
1.00	0.00	3	Kupela	Manyoro C	3	13-Nov-17	292	12.00
1.00	0.00	3	Wanjagnia	ManyoroCH	2	13-Nov-17	293	7.00
1.00	0.00	3	Kupela	Manyoro C	2	13-Nov-17	294	24.00
2.00	1.00	3	Kupella	Manyoro C	2	13-Nov-17	295	36.00
1.00	0.00	3	Dambisi	Manyoro C	2	13-Nov-17	296	26.00
1.00	0.00	3	Wanjangnia	Manyoro C	2	13-Nov-17	297	12.00
2.00	0.00	3	Wanjagnia	Manyoro C	2	13-Nov-17	298	24.00
1.00	0.00	3	Dambisi	Manyoro C	2	13-Nov-17	299	6.00
2.00	1.00	6	zuo	zuo chc	4	16-Nov-17	304	24.00
1.00	0.00	2	Doba	Nayagnia C	2	14-Nov-17	305	16.00
2.00	0.00	3	sabisi	natugnia	6	10-Nov-17	306	24.00
1.00	0.00	2	natugnia	natugnia ch	4	10-Nov-17	307	10.00
2.00	1.00	5	pindaa	pindaa chc	8	16-Nov-17	308	24.00
1.00	1.00	5	Gia	Gia CHC	4	14-Nov-17	309	30.00
1.00	0.00	3	Dambisi	Manyoro C	1	14-Nov-17	311	12.00
1.00	0.00	2	Nayagnia	Nayagnia C	2	14-Nov-17	313	13.00
2.00	1.00	3	Gumongo	Gumongo (	3	14-Nov-17	314	30.00
1.00	0.00	5	Gia	Gia CHC	5	14-Nov-17	315	2.00
1.00	0.00	5	Gia	Gia CHC	4	14-Nov-17	316	8.00
2.00	1.00	2	Nayagnia	Nayagnia C	3	14-Nov-17	317	24.00
2.00	0.00	1	Navrongo	Navrongo H	10	14-Nov-17	318	22.00
1.00	0.00	1	Navrongo	Navrongo H	11	14-Nov-17	319	9.00
2.00	1.00	2	Doba	Nayagnia C	2	14-Nov-17	320	20.00
2.00	1.00	2	Nayagnia	Nayagnia C	3	14-Nov-17	321	24.00
1.00	0.00	4	Punyoro	Punyoro CH	6	14-Nov-17	322	11.00
2.00	1.00	2	Nayagnia	Nayagnia C	3	14-Nov-17	322	24.00
2.00	0.00	2	Nayagnia	Nayagnia C	3	14-Nov-17	323	18.00

1.00	0.00	2	Nayagnia	Nayagnia C	3	14-Nov-17	324	18.00
2.00	1.00	2	Nayagnia	Nayagnia C	3	14-Nov-17	325	24.00
1.00	0.00	2	Nayagnia	Nayagnia C	3	14-Nov-17	326	13.00
2.00	0.00	2	Nayagnia	Nayagnia C	1	14-Nov-17	327	24.00
2.00	1.00	4	Punyoro	Punyoro Cł	6	14-Nov-17	328	24.00
1.00	0.00	4	Punyoro	Punyoro Cł	6	14-Nov-17	329	12.00
1.00	0.00	4	Punyoro	Punyoro Cł	6	14-Nov-17	330	13.00
2.00	1.00	4	Punyoro	Punyoro Cł	6	14-Nov-17	331	24.00
2.00	0.00	4	Punyoro	Punyoro Cł	6	14-Nov-17	332	24.00
2.00	1.00	4	Punyoro	Punyoro Cł	6	14-Nov-17	333	24.00
1.00	0.00	4	punyoro	Punyoro Cł	6	14-Nov-17	334	13.00
2.00	1.00	4	Punyoro	Punyoro Cł	6	14-Nov-17	335	24.00
1.00	1.00	4	Punyoro	Punyoro Cł	7	14-Nov-17	336	10.00
2.00	1.00	4	Punyoro	Punyoro Cł	7	14-Nov-17	337	24.00
1.00	0.00	4	Punyoro	Punyoro Cł	7	14-Nov-17	338	11.00
2.00	1.00	4	Punyoro	Punyoro Cł	7	14-Nov-17	339	24.00
2.00	1.00	4	Punyoro	Punyoro Cł	7	14-Nov-17	340	16.00
1.00	0.00	4	Punyoro	Punyoro Cł	8	14-Nov-17	341	14.00
2.00	1.00	4	Punyoro	Punyoro Cł	8	14-Nov-17	342	24.00
2.00	1.00	4	Punyoro	Punyoro Cł	8	14-Nov-17	343	24.00
1.00	0.00	4	Punyoro	Punyoro Cł	8	14-Nov-17	344	7.00
2.00	1.00	4	Punyoro	PunyoroCH	8	14-Nov-17	345	24.00
2.00	0.00	4	Punyoro	Punyoro Cł	8	14-Nov-17	346	18.00
1.00	0.00	4	Punyoro	Punyoro Cł	8	14-Nov-17	347	26.00
1.00	0.00	5	Gia	Gia CHC	4	14-Nov-17	348	14.00
2.00	0.00	5	Gia	Gia CHC	4	14-Nov-17	349	18.00
1.00	1.00	5	Gia	Gia CHC	8	14-Nov-17	350	27.00
2.00	1.00	5	Gia	Gia CHC	4	14-Nov-17	351	27.00
2.00	0.00	1	Navrongo	Navrongo ł	10	14-Nov-17	352	12.00
2.00	0.00	1	Navrongo	Navrongo (	10	14-Nov-17	353	24.00
1.00	0.00	1	Navrongo	Navrongo (	10	14-Nov-17	354	14.00
1.00	0.00	1	nogecenia	nhc	11	14-Nov-17	355	8.00
2.00	0.00	1	nogecenia	nhc	10	14-Nov-17	356	18.00
2.00	1.00	1	nogecenia	nhc	11	14-Nov-17	357	24.00
1.00	0.00	1	nogecenia	nhc	10	14-Nov-17	358	12.00
2.00	1.00	2	nayagnia	nayagnia cł	3	14-Nov-17	359	25.00
1.00	1.00	2	nayagnia	nayagnia cł	1	14-Nov-17	360	25.00
1.00	1.00	2	nayagnia	nayagniach	1	14-Nov-17	361	13.00
1.00	0.00	2	tankuna	nayagnia cł	2	14-Nov-17	362	18.00
2.00	1.00	2	nayagnia	nayagnia cł	3	14-Nov-17	363	24.00
2.00	1.00	2	barania	nayagnia cł	2	14-Nov-17	364	24.00
1.00	0.00	2	nayagnia	nayagnia cł	3	14-Nov-17	365	9.00
2.00	1.00	2	tankuna	nayagnia cł	2	14-Nov-17	366	24.00
1.00	1.00	5	gia	gia chc	4	14-Nov-17	367	16.00
2.00	1.00	5	gia	gia chc	5	14-Nov-17	368	12.00
1.00	0.00	5	gia	gia chc	9	14-Nov-17	369	15.00
1.00	0.00	5	gia	gia chc	5	14-Nov-17	370	12.00

2.00	1.00	5	gia	gia chc	5	14-Nov-17	371	24.00
1.00	0.00	5	gia	gia chc	5	14-Nov-17	372	24.00
2.00	0.00	5	gia	gia chc	4	14-Nov-17	373	24.00
2.00	1.00	5	gia	gia chc	9	14-Nov-17	374	18.00
1.00	0.00	5	gia	gia chc	4	14-Nov-17	375	12.00
2.00	1.00	5	gia	gia chc	4	14-Nov-17	376	24.00
2.00	0.00	5	gia	gia chc	9	14-Nov-17	377	24.00
1.00	0.00	5	gia	gia chc	4	14-Nov-17	378	10.00
2.00	1.00	5	gia	gia chc	4	14-Nov-17	379	24.00
1.00	1.00	5	gia	gia chc	4	4-Nov-17	380	24.00
1.00	0.00	5	gia	gia chc	4	14-Nov-17	381	12.00
2.00	1.00	5	gia	gia chc	5	14-Nov-17	382	24.00
1.00	0.00	5	gia	gia chc	9	14-Nov-17	383	15.00
1.00	0.00	5	gia	gia chc	9	14-Nov-17	384	9.00
1.00	1.00	5	gia	gia chc	5	14-Nov-17	385	24.00
2.00	0.00	5	gia	gia chc	4	14-Nov-17	386	27.00
2.00	1.00	5	gia	gia chc	4	14-Nov-17	387	26.00
1.00	1.00	5	gia	gia chc	5	14-Nov-17	388	9.00
2.00	1.00	5	gia	gia chc	5	14-Nov-17	389	24.00
2.00	1.00	5	gia	gia chc	4	14-Nov-17	390	24.00
1.00	1.00	5	gia	gia chc	4	14-Nov-17	391	11.00
2.00	0.00	1	Navrongo	Navrongo (	10	15-Nov-17	392	18.00
2.00	1.00	1	Navrongo	Navrongo (	11	15-Nov-17	393	24.00
1.00	0.00	1	Navrongo	Navrongo	11	15-Nov-17	394	24.00
1.00	1.00	1	Navrongo	Navrongo H	11	15-Nov-17	395	12.00
2.00	1.00	1	Navrongo	Navrongo H	11	15-Nov-17	396	24.00
2.00	1.00	1	Navrongo	Navrongo H	10	15-Nov-17	397	22.00
1.00	0.00	1	Navrongo	Navrongo H	10	15-Nov-17	398	8.00
2.00	1.00	1	Navrongo	Navrovgo H	10	15-Nov-17	399	19.00
1.00	0.00	1	Navrongo	Navrongo H	11	15-Nov-17	400	7.00
1.00	1.00	1	Navrongo	Navrongo H	10	15-Nov-17	401	8.00
1.00	1.00	1	Navrongo	Navrongo H	10	15-Nov-17	402	9.00
2.00	0.00	1	Navrongo	Navrongo H	10	15-Nov-17	403	24.00
2.00	0.00	1	Navrongo	Navrongo H	10	15-Nov-70	404	17.00
2.00	1.00	1	Navrongo	Navrongo H	11	15-Nov-17	405	24.00
1.00	1.00	1	Navrongo	Navrongo H	10	15-Nov-17	406	12.00
1.00	0.00	1	Navrongo	Navrongo H	10	15-Nov-17	407	11.00
1.00	0.00	1	Navrongo	Navrongo H	11	15-Nov-17	408	12.00
1.00	0.00	1	Navrongoa	Navrongo H	10	15-Nov-17	409	9.00
1.00	1.00	1	Navrongo	Navrongo H	10	14-Nov-17	410	10.00
2.00	1.00	1	Navrongo	Navrongo H	7	15-Nov-17	411	28.00
1.00	0.00	1	Navrongo	Navrongo H	10	15-Nov-17	412	8.00
2.00	1.00	1	Navrongo	Navrongo H	10	16-Nov-17	413	21.00
2.00	1.00	1	Navrongo	Navrongo H	10	16-Nov-17	414	24.00
1.00	0.00	1	Navrongo	Navrongon	10	16-Nov-17	415	10.00
1.00	1.00	1	Navrongo	Navrongo H	11	16-Nov-17	416	14.00
1.00	1.00	1	Navrongo	Navrongo H	11	16-Nov-17	417	11.00

2.00	1.00	1	Navrongo	Navrongo H	11	16-Nov-17	418	24.00
2.00	0.00	1	Navrongo	Navrongo H	11	16-Nov-17	419	18.00
1.00	0.00	1	Navrongo	navrongo H	11	16-Nov-17	420	14.00
2.00	0.00	1	Navrongo	Navrongo H	11	16-Nov-17	421	18.00
1.00	0.00	1	Navrongo	Navrongo H	10	16-Nov-17	422	13.00
2.00	1.00	1	Navrongo	Navrongo H	11	16-Nov-17	423	2.00
1.00	0.00	5	Pindia	Pindia CHPS	8	16-Nov-17	424	11.00
2.00	1.00	5	Pindia	Pindia CHPS	8	16-Nov-17	425	27.00
1.00	0.00	5	pindaa	pindaa chc	8	16-Nov-17	426	36.00
2.00	1.00	5	pindaa	pindaa chc	8	16-Nov-17	427	24.00
1.00	0.00	5	pindaa	pindaa chc	8	16-Nov-17	428	8.00
2.00	1.00	5	pindaa	pindaa chc	7	16-Nov-17	429	30.00
1.00	1.00	5	pindaa	pindaa chc	9	16-Nov-17	430	16.00
2.00	0.00	6	zuo	zuo chc	9	16-Nov-17	431	20.00
1.00	0.00	2	doba	doba chc	3	16-Nov-17	432	13.00
2.00	0.00	2	doba	doba chc	3	16-Nov-17	433	30.00
1.00	1.00	2	doba	doba chc	3	16-Nov-17	434	12.00
1.00	1.00	2	doba	doba chc	3	16-Nov-17	435	14.00
1.00	1.00	2	doba	doba chc	3	16-Nov-17	436	9.00
2.00	0.00	2	doba	doba chc	3	16-Nov-17	437	18.00
1.00	0.00	2	doba	doba chc	3	16-Nov-17	438	12.00
2.00	1.00	2	doba	doba chc	3	16-Nov-17	439	22.00
1.00	1.00	2	doba	doba chc	3	16-Nov-17	440	12.00
1.00	0.00	2	doba	doba chc	3	14-Nov-17	441	24.00
2.00	1.00	2	doba	doba chc	3	16-Nov-17	442	25.00
1.00	0.00	2	doba	doba chc	3	16-Nov-17	443	16.00
1.00	0.00	2	doba	doba chc	3	16-Nov-17	444	10.00
1.00	0.00	2	doba	doba chc	3	16-Nov-17	445	12.00
2.00	1.00	2	doba	doba chc	3	16-Nov-17	446	18.00
2.00	1.00	2	doba	doba chc	3	16-Nov-17	447	24.00
1.00	0.00	2	doba	doba chc	3	16-Nov-17	448	36.00
1.00	1.00	2	doba	doba chc	3	16-Nov-17	449	11.00
2.00	1.00	2	doba	doba chc	3	16-Nov-17	450	18.00
2.00	0.00	2	doba	doba chc	3	16-Nov-17	451	24.00
1.00	0.00	6	zuo	zuo chc	6	16-Nov-17	452	12.00
1.00	0.00	6	zuo	zuo chc	4	16-Nov-17	453	12.00
2.00	1.00	6	zuo	zuo chc	9	16-Nov-17	454	24.00
1.00	0.00	6	zuo	zuo chc	4	16-Nov-17	455	9.00
2.00	1.00	6	zuo	zuo chc	4	16-Nov-17	456	24.00
1.00	0.00	6	zuo	zuo chc	4	16-Nov-17	457	12.00
2.00	1.00	6	zuo	zuo chc	4	16-Nov-17	458	18.00
1.00	0.00	6	zuo	zuo chc	4	16-Nov-17	459	20.00
1.00	1.00	6	zuo	zuo chc	4	16-Nov-17	460	11.00
2.00	1.00	6	zuo	zuo chc	6	16-Nov-17	461	24.00
2.00	1.00	6	zoongo	zoongo chc	5	16-Nov-17	462	24.00
1.00	0.00	6	zoongo	zoongo chc	5	16-Nov-17	463	12.00
2.00	1.00	6	zoongo	zoongo chc	6	16-Nov-17	464	28.00

1.00	1.00	6	zoongo	zoongo chc	6	16-Nov-17	465	27.00
2.00	0.00	6	zoongo	zoongo chc	6	16-Nov-17	467	24.00
1.00	1.00	6	zoongo	zoongo chc	6	16-Nov-17	468	10.00
2.00	0.00	6	zoongo	zoongo chc	6	16-Nov-17	469	24.00
2.00	1.00	6	zoogo	zoongo chc	9	16-Nov-17	470	31.00
1.00	0.00	6	zoongo	zoongo chc	6	16-Nov-17	471	7.00
1.00	0.00	6	zoongo	zoongo chc	9	16-Nov-17	472	11.00
2.00	1.00	6	zoongo	zoongo chc	9	16-Nov-17	473	36.00
1.00	0.00	6	zoongo	zoongo chc	6	16-Nov-17	474	13.00
1.00	0.00	6	zoongo	zoongo chc	6	16-Nov-17	475	14.00
2.00	0.00	6	zoongo	zoongo chc	5	16-Nov-17	476	24.00
2.00	0.00	6	zoongo	zoongo chc	5	16-Nov-17	477	24.00
1.00	0.00	6	zoongo	zoongo chc	6	16-Nov-17	478	12.00
1.00	0.00	6	zoongo	zoongo chc	4	16-Nov-17	479	14.00
2.00	1.00	6	zoongo	zoongo chc	4	16-Nov-17	480	24.00
2.00	1.00	6	zuo	zuo chc	4	16-Nov-17	481	20.00
2.00	1.00	6	zuo	zuo chc	9	16-Nov-17	482	30.00
1.00	1.00	6	zuo chc	zuo chc	9	16-Nov-17	483	10.00
1.00	0.00	6	zuo	zuo chc	9	16-Nov-17	484	24.00
2.00	1.00	6	zuo	zuo chc	9	16-Nov-17	485	29.00
1.00	0.00	6	zuo	zuo chc	9	16-Nov-17	486	12.00
2.00	1.00	6	zuo	zuo chc	5	16-Nov-17	487	24.00
2.00	0.00	6	zuo	zuo chc	6	16-Nov-17	488	36.00
1.00	0.00	6	zuo	zuo chc	6	16-Nov-17	489	11.00
2.00	0.00	6	zuo	zuo chc	6	16-Nov-17	490	25.00
2.00	0.00	6	zuo	zuo chc	9	16-Nov-17	491	24.00
1.00	1.00	6	zuo	zuo chc	4	16-Nov-17	492	10.00
2.00	1.00	6	zuo	zuo chc	4	14-Nov-17	493	24.00
2.00	1.00	6	zuo	zuo chc	4	16-Nov-17	494	24.00
1.00	0.00	6	zuo	zuo chc	4	16-Nov-17	495	12.00
1.00	0.00	6	zuo	zuo chc	4	16-Nov-17	496	9.00
2.00	1.00	6	zuo	zuo chc	4	16-Nov-17	497	18.00
2.00	1.00	6	zuo	zuo chc	4	16-Nov-17	498	24.00
1.00	0.00	6	zuo	zuo chc	4	16-Nov-17	499	15.00
2.00	1.00	6	zuo	zuo chc	4	16-Nov-17	500	24.00
2.00	1.00	6	zuo	zuo chc	6	16-Nov-17	501	20.00
1.00	0.00	6	zuo	zuo chc	6	16-Nov-17	502	25.00
2.00	1.00	6	zuo	zuo chc	6	16-Nov-17	503	24.00
1.00	0.00	6	zuo	zuo chc	6	16-Nov-17	504	11.00
2.00	1.00	6	zuo	zuo chc	6	16-Nov-17	505	27.00
1.00	0.00	6	zuo	zuo chc	6	16-Nov-17	506	24.00
1.00	0.00	6	zuo	zuo chc	6	16-Nov-17	507	8.00
2.00	1.00	6	zuo	zuo chc	9	16-Nov-17	508	24.00
1.00	0.00	6	zuo	zuo chc	9	16-Nov-17	509	24.00
2.00	1.00	6	zuo	zuo chc	9	16-Nov-17	510	24.00
1.00	0.00	6	zuo	zuo chc	9	16-Nov-17	511	10.00
2.00	1.00	6	zuo	zuo chc	9	16-Nov-17	512	30.00

2.00	1.00	6	zuo	zuo chc	9	16-Nov-17	513	26.00
1.00	0.00	6	zuo	zuo chc	9	16-Nov-17	514	12.00
1.00	0.00	6	zuo	zuo chc	9	16-Nov-17	515	11.00
2.00	0.00	6	zuo	zuo chc	9	16-Nov-17	516	15.00
2.00	1.00	6	zuo	zuo chc	9	16-Nov-17	517	36.00
1.00	0.00	6	zuo	zuo chc	9	16-Nov-17	518	36.00
2.00	1.00	6	zuo	zuo chc	9	16-Nov-17	519	25.00
1.00	0.00	6	zuo	zuo chc	9	16-Nov-17	520	14.00
2.00	1.00	6	zuo	zuo chc	9	16-Nov-17	521	24.00
1.00	1.00	6	zuo	zuo chc	9	16-Nov-17	521	12.00
2.00	1.00	6	zuo	zuo chc	9	16-Nov-17	523	48.00
2.00	0.00	6	zuo	zuo chc	9	16-Nov-17	524	28.00
1.00	1.00	6	zuo	zuo chc	9	16-Nov-17	525	13.00
2.00	1.00	6	zuo	zuo chc	9	16-Nov-17	526	24.00
1.00	0.00	6	zuo	zuo chc	4	16-Nov-17	527	13.00
2.00	1.00	6	zuo	zuo chc	4	16-Nov-17	528	24.00
1.00	0.00	6	zuo	zuo chc	4	16-Nov-17	529	11.00
2.00	1.00	6	zuo	zuo chc	4	16-Nov-17	530	27.00
2.00	1.00	6	Zuo	Zuo CHPS	4	16-Nov-17	531	24.00
1.00	1.00	6	Zuo	Zuo CHPS	9	16-Nov-17	532	12.00
1.00	0.00	6	Zuo	Zuo CHPS	9	16-Nov-17	533	13.00
2.00	1.00	6	ZUO	ZUO CHPS	9	16-Nov-17	534	24.00
2.00	1.00	6	Zuo	Zuo CHPS	4	16-Nov-17	535	20.00
1.00	0.00	6	Zuo	Zuo CHPS	4	16-Nov-17	536	18.00
2.00	1.00	6	Zuo	Zuo CHPS	4	16-Nov-17	537	24.00
1.00	0.00	1	Gongnia	Gongnia H/	4	16-Nov-17	538	10.00
2.00	0.00	1	Gongnia	Gongnia CI	9	17-Nov-17	539	24.00
1.00	0.00	1	Gongnia	Gongian CI	9	17-Nov-17	540	11.00
1.00	1.00	1	Gongnia	Gongnia CI	4	17-Nov-70	541	16.00
2.00	0.00	1	Nogsenia	Hospital	7	17-Nov-17	542	22.00
2.00	0.00	1	Nosegnia	Hospital	7	17-Nov-17	543	23.00
1.00	1.00	1	Nogsinia	Hospital	7	17-Nov-17	544	13.00
2.00	1.00	1	hospital	wmh	7	17-Nov-17	545	18.00
1.00	1.00	1	hospital	hospital	7	17-Nov-17	546	18.00
1.00	0.00	1	hospital	hospital	7	17-Nov-17	547	14.00
2.00	1.00	1	namolo	nhc	3	17-Nov-17	548	24.00
2.00	1.00	1	namolo	nhc	3	17-Nov-17	549	18.00
1.00	0.00	1	namolo	nhc	2	17-Nov-17	550	12.00
2.00	1.00	1	namolo	nch	3	17-Nov-17	551	24.00
1.00	0.00	1	namolo	nch	2	17-Nov-17	552	7.00
2.00	1.00	1	namolo	nch	3	17-Nov-17	553	16.00
1.00	0.00	1	namolo	hospital	8	17-Nov-17	554	19.00
1.00	0.00	1	nogecenia	wmh	8	17-Nov-17	555	10.00
1.00	1.00	1	nogecenia	wmh	8	17-Nov-17	556	10.00
1.00	0.00	1	nogecenia	wmh	5	17-Nov-17	557	13.00
1.00	0.00	1	nogecenia	wmh	8	17-Nov-17	558	11.00
2.00	1.00	1	nogecenia	wmh	8	17-Nov-17	559	24.00

1.00	0.00	1	nogecenia	wmh	8	17-Nov-17	560	13.00
2.00	0.00	1	nogesenia\	WMH	8	20-Nov-17	561	12.00
1.00	1.00	1	nogesenia	WMH	8	20-Nov-17	562	11.00
2.00	1.00	1	nogesenia	WMH	8	20-Nov-17	563	21.00
1.00	1.00	1	nogesenia	WMH	8	20-Nov-17	564	14.00
2.00	1.00	1	nogesenia	WMH	8	20-Nov-17	565	24.00
1.00	0.00	1	nogesenia	WMH	7	20-Nov-17	566	12.00
2.00	1.00	1	nogesenia	WMH	7	20-Nov-17	567	24.00
1.00	1.00	1	nogesenia	WMH	7	20-Nov-17	568	9.00
2.00	1.00	1	nogesenia	WMH	7	20-Nov-17	569	18.00
2.00	1.00	1	nogesenia	WMH	8	20-Nov-17	570	24.00
1.00	0.00	1	nogesenia	WMH	8	20-Nov-17	571	13.00
1.00	0.00	1	nogesenia	WMH	8	20-Nov-17	26	11.00
2.00	1.00	3	Benyim	Manyoro C	3	13-Nov-17	223	27.00

Duration2	Sex	Birthwt	Age1	Age2	Alive	WEIGHTCU	HEIGHTCUI	Estimateda
2.00	2	3.50	21	68	1	12.90	88.0	68
2.00	2	2.90	11	42	1	9.10	72.2	42
1.00	2	3.60	13	21	1	10.40	86.5	21
1.00	1	2.90	8	23	1	7.50	70.3	23
2.00	1	3.20	2	63	1	5.20	59.6	63
2.00	1	3.00	6	52	1	8.90	70.0	52
1.00	2	3.50	12	20	1	8.90	78.9	20
1.00	2	3.30	13	23	1	8.20	74.0	23
2.00	1	3.50	4	76	1	7.40	65.5	76
2.00	1	3.40	7	22	1	7.20	68.0	22
1.00	1	2.40	4	20	1	7.10	61.5	20
2.00	1	3.60	4	42	1	8.30	68.2	42
1.00	1	2.60	5	24	1	7.40	64.2	24
2.00	1	2.50	8	23	1	6.90	67.9	23
2.00	1	2.10	3	98	1	4.10	54.9	98
1.00	2	2.50	5	21	1	6.20	64.1	21
2.00	1	2.60	6	43	1	8.20	64.6	43
1.00	2	4.00	11	23	1	6.60	62.8	23
1.00	1	2.70	3	24	1	6.70	59.2	24
2.00	1	3.00	3	87	1	6.70	61.2	87
2.00	2	2.70	5	81	1	7.30	66.0	81
1.00	2	2.80	8	22	1	7.50	66.0	22
2.00	1	2.80	8	72	1	9.70	73.3	72
1.00	1	2.90	12	22	1	8.90	83.7	22
1.00	2	2.50	19	72	1	9.30	75.0	72
2.00	2	3.00	7	87	1	7.30	62.2	87
2.00	2	3.30	4	48	1	7.30	65.3	48
1.00	1	3.20	9	24	1	9.00	76.4	24
2.00	1	3.50	13	96	1	10.50	79.9	96
2.00	2	3.10	8	20	1	9.70	71.7	20
1.00	1	2.90	8	21	1	7.70	69.4	21
1.00	1	2.70	7	48	1	8.20	67.9	24
2.00	1	2.70	11	72	1	9.40	74.8	72
2.00	1	2.40	11	39	1	8.00	65.4	39
1.00	2	2.90	11	23	1	9.30	68.3	23
1.00	1	2.80	6	19	1	9.20	70.4	19
2.00	1	2.60	20	45	1	13.50	78.0	45
1.00	2	2.50	18	39	1	10.90	78.0	39
1.00	1	3.00	11	21	1	7.50	73.0	21
1.00	1	2.60	10	23	1	8.00	76.0	23
2.00	1	2.90	24	71	1	12.00	87.0	71
1.00	1	2.30	12	19	1	9.50	74.2	19
1.00	1	1.70	12	42	1	9.30	75.0	42
2.00	2	3.20	23	72	1	9.90	75.9	72
2.00	1	2.80	24	64	1	12.00	84.3	64
2.00	1	3.10	13	48	1	12.00	84.3	48

2.00	1	2.80	24	45	1	13.10	75.6	45
1.00	1	3.40	7	22	1	7.40	68.2	22
2.00	1	2.60	5	56	1	5.50	69.3	56
2.00	2	2.90	6	72	1	6.90	69.3	72
1.00	2	2.80	6	21	1	6.20	66.4	21
2.00	2	3.00	3	63	1	6.80	65.7	63
1.00	2	2.60	11	23	1	7.00	67.5	23
1.00	1	2.70	19	31	1	10.00	81.0	31
2.00	1	3.15	24	72	1	10.00	86.0	72
1.00	2	3.40	5	24	1	9.50	69.0	24
1.00	1	2.70	10	23	1	9.50	69.0	23
1.00	1	3.90	14	43	0	8.50	70.9	24
2.00	1	2.60	2	48	1	4.50	60.0	48
2.00	2	3.10	20	49	1	10.30	79.0	49
1.00	2	2.30	4	19	1	6.50	65.0	19
2.00	1	2.80	24	53	1	12.00	76.0	53
1.00	2	2.90	2	20	1	5.50	59.6	20
2.00	2	2.70	13	36	1	9.00	68.0	36
2.00	2	2.80	21	66	1	10.00	83.3	66
1.00	1	2.40	6	22	1	6.50	70.2	22
2.00	1	3.70	5	35	1	6.00	68.0	35
2.00	1	2.70	22	52	1	10.60	87.6	52
1.00	2	2.50	10	23	1	10.20	82.6	23
2.00	1	3.00	5	40	1	6.60	70.9	40
1.00	1	3.20	12	20	1	9.00	81.3	20
1.00	1	3.10	7	23	1	7.40	68.0	23
2.00	1	3.30	8	35	1	7.00	69.9	35
1.00	2	2.60	11	31	1	7.40	74.3	31
1.00	2	3.00	4	24	1	7.10	66.0	24
1.00	2	2.60	15	48	1	10.70	81.0	48
1.00	2	3.00	6	21	1	10.10	76.0	21
2.00	2	3.30	18	36	1	8.60	77.0	36
1.00	1	2.70	3	21	1	6.00	67.2	21
2.00	2	3.10	11	68	1	8.80	74.0	68
2.00	2	2.70	17	72	1	9.50	78.0	72
1.00	1	2.80	11	20	1	8.30	73.0	20
2.00	1	3.00	10	49	1	9.30	75.0	20
2.00	1	2.90	15	61	1	8.40	69.0	61
1.00	2	2.40	7	23	0	8.00	70.7	23
2.00	1	3.10	24	49	1	13.00	78.0	49
2.00	2	3.20	19	55	1	8.00	70.0	55
1.00	2	2.40	14	24	0	7.30	70.1	24
2.00	1	3.40	5	38	1	7.70	67.2	38
1.00	2	2.90	12	22	1	8.00	80.1	22
2.00	2	2.60	11	72	1	8.20	72.1	72
2.00	1	3.10	3	24	1	5.10	63.6	24
1.00	1	2.90	5	23	1	6.40	70.2	23

1.00	1	2.90	23	57	1	9.00	81.3	57
1.00	2	3.60	8	22	1	8.00	74.2	22
2.00	1	2.90	5	48	1	5.90	69.0	48
1.00	1	2.50	3	19	1	6.70	65.6	19
2.00	1	2.50	17	48	1	8.70	69.6	48
1.00	2	2.80	6	23	1	6.00	64.1	23
2.00	2	2.80	12	60	1	8.30	73.0	60
1.00	1	2.60	4	19	1	8.30	72.5	19
2.00	1	3.30	10	67	1	7.50	66.2	67
2.00	2	2.60	12	23	1	8.20	78.9	23
2.00	1	3.00	9	48	1	7.80	71.3	48
1.00	1	3.00	3	20	1	7.80	71.2	20
2.00	1	3.30	19	72	1	9.60	85.5	72
2.00	2	3.00	8	46	1	8.40	73.0	46
2.00	2	3.10	24	72	1	14.00	90.3	72
1.00	2	2.90	4	19	1	6.90	66.3	19
1.00	2	2.90	4	24	1	5.50	60.0	24
1.00	2	3.30	4	22	1	6.80	68.0	22
2.00	1	2.70	16	60	1	9.00	81.2	60
1.00	2	2.70	3	18	1	5.30	55.9	18
2.00	1	1.90	20	38	1	11.40	76.8	38
2.00	2	2.60	24	62	1	9.30	70.3	62
1.00	1	3.20	3	22	1	5.20	63.4	22
2.00	2	2.90	23	59	1	12.10	88.5	59
2.00	2	2.60	23	65	1	10.00	85.1	65
1.00	2	3.10	5	24	1	6.40	65.4	24
1.00	2	2.30	5	34	1	6.10	66.1	34
2.00	2	3.70	11	36	1	9.70	77.6	36
1.00	1	3.30	4	22	1	6.10	60.8	22
2.00	1	2.50	19	60	1	9.50	74.2	60
1.00	2	2.60	7	23	1	9.00	70.0	23
1.00	2	3.00	3	23	1	6.00	61.2	23
2.00	2	3.00	2	40	1	4.80	59.2	40
1.00	1	3.30	5	24	1	7.80	73.0	24
2.00	1	3.30	19	87	1	10.10	76.1	87
2.00	1	3.00	6	21	1	6.80	68.6	21
1.00	1	3.20	2	23	0	6.00	61.5	23
2.00	1	2.40	17	57	1	9.40	80.0	57
1.00	2	3.00	8	21	1	6.90	69.6	21
2.00	2	2.70	16	48	1	8.00	78.0	48
1.00	1	3.20	6	22	1	7.20	72.0	22
2.00	2	3.00	10	48	1	10.00	75.3	48
1.00	1	3.40	3	24	1	7.90	58.8	24
1.00	2	2.90	11	23	1	7.50	72.1	23
2.00	2	2.80	7	39	1	7.00	70.4	39
1.00	2	2.60	8	23	1	6.40	65.0	23
1.00	1	2.70	6	18	1	5.40	65.5	18

2.00	2	3.20	22	83	1	11.60	76.0	83
1.00	1	2.40	6	21	1	7.40	72.2	21
2.00	1	3.30	19	45	1	10.30	83.0	45
1.00	2	3.00	4	36	1	6.00	65.0	36
1.00	1	2.90	2	48	1	5.20	56.0	48
1.00	1	3.00	8	23	1	8.40	79.0	23
1.00	1	3.50	5	24	1	7.00	68.0	24
1.00	1	3.00	7	48	1	8.70	70.5	48
1.00	1	2.90	9	24	1	9.00	68.8	24
2.00	1	2.60	14	132	1	8.70	75.4	132
1.00	1	3.00	5	24	1	8.00	75.0	24
2.00	2	3.40	2	48	1	4.60	55.0	48
1.00	1	3.30	20	49	1	10.90	87.4	49
1.00	1	3.60	10	24	1	10.00	79.0	24
2.00	2	2.80	17	66	1	9.30	77.0	66
2.00	2	3.00	9	23	1	9.00	68.0	23
2.00	2	2.60	24	36	1	8.90	81.0	36
1.00	1	3.00	9	22	1	9.60	75.0	22
1.00	1	2.60	17	96	1	8.30	77.0	96
1.00	1	4.00	7	23	1	9.00	74.0	23
2.00	2	3.00	16	48	1	8.90	80.0	48
1.00	2	2.70	4	19	1	7.80	57.7	19
1.00	2	3.40	7	23	1	6.40	69.0	23
2.00	1	2.80	23	60	1	9.10	80.5	60
2.00	1	3.50	24	72	1	10.00	85.0	72
1.00	1	3.40	9	24	1	7.20	69.0	24
2.00	1	2.80	11	81	1	14.50	79.5	81
1.00	1	2.90	8	23	1	8.50	60.0	23
2.00	2	3.00	6	51	1	7.60	69.0	51
1.00	2	2.90	7	20	1	7.90	69.7	20
2.00	1	4.20	19	54	1	10.20	78.2	54
1.00	2	2.50	4	24	1	6.00	65.0	24
2.00	2	2.50	13	49	1	8.40	74.2	49
2.00	2	2.90	23	77	1	10.50	81.8	77
1.00	1	3.20	7	23	1	8.00	76.5	23
2.00	1	3.00	2	21	1	5.00	57.2	21
1.00	2	2.80	3	18	1	8.80	68.5	18
1.00	1	2.50	17	36	1	8.60	79.3	36
1.00	2	2.50	9	24	1	6.20	67.3	24
2.00	2	3.00	9	45	1	8.40	72.3	45
1.00	1	2.60	6	23	1	7.50	63.2	23
2.00	1	3.80	13	40	1	9.60	77.2	40
1.00	1	2.90	8	21	1	8.90	78.5	21
1.00	1	2.80	6	24	1	7.00	64.3	24
2.00	1	3.10	23	84	1	11.20	83.5	84
2.00	1	2.70	9	38	1	8.50	80.5	38
1.00	2	3.40	7	24	1	8.50	60.1	24

2.00	2	2.90	23	48	1	9.00	75.6	48
1.00	1	3.00	6	22	1	7.90	66.6	22
1.00	1	2.50	11	22	1	10.60	76.8	22
1.00	2	3.10	9	24	1	8.60	71.2	24
2.00	1	2.10	9	55	1	7.50	71.0	55
1.00	2	2.90	8	31	1	7.50	69.1	31
1.00	2	2.90	11	21	1	7.90	74.4	21
2.00	1	3.40	8	48	1	8.40	72.8	48
2.00	1	3.40	5	51	1	7.60	66.9	51
1.00	2	2.54	8	22	1	6.30	70.4	22
1.00	2	2.30	15	69	1	8.30	70.0	69
1.00	2	2.30	8	21	1	7.01	67.8	21
2.00	1	2.50	12	53	1	7.40	72.1	53
1.00	2	2.90	8	20	1	7.80	79.8	20
2.00	2	2.50	10	38	1	8.50	67.0	38
1.00	1	3.30	11	52	1	10.60	76.8	52
1.00	2	3.00	6	23	1	10.40	68.0	23
2.00	1	3.90	4	168	1	5.00	60.0	40
1.00	1	3.25	5	22	0	6.00	67.5	22
2.00	2	2.90	24	59	1	12.70	89.1	59
2.00	1	3.20	24	83	1	10.50	80.6	83
1.00	1	3.20	7	24	1	8.00	76.6	24
2.00	2	3.40	24	72	1	12.50	88.3	72
1.00	1	3.12	3	23	1	5.00	60.0	23
1.00	1	2.90	7	35	1	8.70	73.8	35
1.00	2	3.00	6	19	1	6.80	68.1	19
1.00	2	2.00	4	23	1	5.40	63.0	23
2.00	1	3.20	3	41	1	5.60	62.0	41
1.00	2	2.80	8	19	1	7.80	67.1	19
2.00	1	3.40	24	84	1	10.20	80.5	84
1.00	2	3.00	9	22	1	8.70	72.7	22
1.00	2	3.50	2	54	1	4.80	60.4	54
2.00	1	2.80	4	19	1	5.40	65.0	19
1.00	2	2.90	4	19	1	9.00	65.0	19
2.00	1	3.00	20	84	1	8.80	82.3	84
1.00	2	2.30	4	23	1	5.70	67.1	23
1.00	1	2.60	2	70	1	6.10	60.2	70
2.00	2	3.10	7	99	1	7.80	73.0	99
1.00	2	3.80	6	24	1	8.00	70.0	24
1.00	1	2.40	12	22	1	8.50	77.2	22
2.00	1	2.50	14	53	1	10.60	78.5	53
2.00	1	3.20	7	21	1	5.60	62.5	21
1.00	1	3.20	10	23	1	10.00	72.6	23
2.00	1	2.80	24	59	1	12.30	87.0	59
1.00	2	2.70	13	24	1	8.20	71.0	24
1.00	2	2.70	3	51	1	6.80	69.0	51
2.00	1	3.20	5	59	1	7.00	65.9	59

1.00	2	2.70	5	24	1	6.90	63.8	24
1.00	1	3.10	5	23	1	6.90	66.8	23
2.00	2	2.90	6	19	1	6.80	67.2	19
2.00	1	2.00	20	96	1	8.40	80.5	96
1.00	2	2.80	5	21	1	6.80	65.0	21
2.00	2	3.10	4	42	1	7.11	63.8	42
2.00	2	2.90	9	50	1	9.10	73.2	50
1.00	1	2.30	10	23	1	7.60	73.6	23
2.00	2	2.90	12	48	1	7.50	72.7	48
1.00	2	3.00	12	23	1	9.50	77.6	23
2.00	1	2.90	15	84	1	8.80	77.5	84
1.00	2	3.30	10	23	1	9.40	73.0	23
1.00	2	2.60	3	54	1	5.00	62.0	54
1.00	1	3.20	9	24	1	9.00	77.4	24
1.00	1	3.30	13	23	1	9.20	78.6	23
2.00	1	3.40	2	48	1	6.20	64.0	48
1.00	2	3.00	9	24	1	10.60	72.0	24
2.00	1	2.40	21	72	1	8.90	81.9	72
1.00	2	3.10	11	23	1	7.70	73.6	23
2.00	2	3.00	13	48	1	10.50	79.5	48
1.00	2	2.90	6	24	1	8.20	70.0	24
1.00	2	3.50	7	23	0	10.10	68.3	23
2.00	1	2.40	15	36	1	7.90	78.4	36
2.00	1	3.00	8	60	1	6.70	67.8	60
2.00	2	2.20	8	22	1	7.51	71.6	22
1.00	2	3.00	11	24	1	7.50	71.1	24
2.00	1	2.80	8	36	1	7.90	70.7	36
1.00	1	2.10	11	22	1	7.90	69.2	22
2.00	1	2.50	4	79	1	6.70	61.5	79
1.00	2	3.40	6	23	1	7.70	68.2	23
2.00	1	3.00	22	52	1	10.60	87.6	52
1.00	2	2.40	14	23	0	7.30	70.1	23
2.00	1	2.60	5	49	1	5.10	61.0	49
2.00	2	2.60	24	58	1	9.30	77.2	58
1.00	2	2.61	7	24	1	8.90	76.9	24
1.00	1	3.00	5	23	1	7.00	72.7	23
2.00	2	2.68	24	65	1	11.00	83.7	65
1.00	1	3.10	9	23	1	10.40	70.1	23
1.00	2	2.80	3	24	1	6.50	65.0	24
2.00	1	2.80	24	72	1	11.10	85.3	72
1.00	2	3.00	24	48	1	10.00	86.0	48
1.00	1	2.70	5	22	1	8.50	66.1	22
1.00	2	3.40	6	45	1	7.70	68.2	45
2.00	1	3.10	6	72	1	8.00	68.4	72
1.00	2	2.50	9	24	1	7.90	70.0	24
2.00	1	2.70	13	49	1	8.60	81.3	49
1.00	2	3.30	24	48	1	9.70	80.7	48

1.00	2	3.70	3	21	1	6.30	62.2	21
2.00	1	3.70	5	77	1	8.30	67.0	77
1.00	1	3.40	3	23	1	5.10	59.5	23
2.00	2	3.30	6	32	1	6.00	66.4	32
2.00	1	2.50	14	52	1	8.30	83.7	52
1.00	2	2.50	9	23	1	8.60	74.5	23
1.00	1	2.00	6	23	1	9.20	73.0	23
2.00	1	2.90	5	48	1	6.90	65.0	48
2.00	2	3.10	12	24	1	8.50	74.0	24
2.00	1	3.00	3	44	1	7.00	56.0	44
1.00	2	2.90	11	24	1	8.80	70.0	24
2.00	2	2.60	18	71	1	8.50	79.7	71
1.00	2	3.40	7	23	1	8.70	72.0	23
2.00	2	2.90	20	53	1	8.60	84.0	53
1.00	1	2.70	12	24	1	7.90	70.0	24
2.00	1	2.90	23	99	1	13.90	89.0	99
1.00	1	3.50	11	76	1	8.80	74.0	76
1.00	2	3.00	3	24	1	8.20	64.0	24
2.00	1	3.50	2	46	1	7.00	59.4	46
2.00	2	2.80	11	49	1	8.70	70.0	49
1.00	1	3.20	10	19	1	9.60	76.5	19
2.00	1	3.10	13	99	1	8.60	77.0	99
1.00	1	3.60	8	37	1	7.40	73.3	37
2.00	1	2.70	4	20	1	5.40	64.3	20
1.00	1	2.60	2	24	1	5.70	60.1	24
1.00	2	2.70	5	31	1	7.00	64.1	31
2.00	1	3.00	11	22	1	8.80	74.1	22
2.00	2	3.30	11	62	1	7.70	72.8	62
1.00	1	2.80	5	35	1	6.20	68.0	35
2.00	2	2.60	23	42	1	9.50	81.1	42
1.00	2	2.90	2	23	1	5.50	57.0	23
1.00	2	3.20	5	24	1	7.50	72.0	24
1.00	2	3.40	10	36	1	7.20	68.3	36
2.00	2	4.50	16	60	1	9.50	79.1	60
1.00	1	2.00	8	22	1	6.50	64.6	22
2.00	1	3.80	9	55	1	10.20	72.4	55
2.00	1	2.90	13	21	1	9.60	79.1	21
1.00	1	4.40	11	24	1	10.80	80.1	24
1.00	1	2.60	6	23	1	7.70	65.9	23
2.00	2	3.20	18	62	1	11.10	79.0	62
2.00	2	3.40	11	63	1	8.10	71.0	63
1.00	1	2.60	6	21	1	6.10	65.0	21
2.00	1	3.00	11	56	1	7.90	70.0	56
1.00	1	2.50	11	62	1	9.40	73.5	62
1.00	2	3.70	8	43	1	7.20	71.0	43
1.00	1	3.90	4	24	1	6.80	60.0	24
1.00	2	2.90	8	20	1	8.80	77.0	20

2.00	1	3.10	16	66	1	9.10	76.0	66
2.00	2	3.10	5	20	1	8.90	67.0	20
2.00	2	3.10	6	38	1	8.20	65.4	38
1.00	2	3.70	6	42	1	6.20	66.0	42
1.00	2	3.50	13	24	1	8.40	75.0	24
2.00	1	3.00	19	67	1	10.60	80.0	67
2.00	1	2.90	13	35	1	8.60	74.9	35
1.00	1	3.30	3	24	1	7.50	64.0	24
2.00	2	2.50	16	52	1	9.00	76.5	52
2.00	1	3.20	8	23	1	7.60	68.2	23
1.00	1	3.50	7	21	1	9.40	78.0	21
2.00	2	3.10	5	50	1	7.90	68.0	50
1.00	2	2.60	10	22	1	7.00	72.0	22
1.00	2	2.70	7	19	1	9.00	73.0	19
2.00	2	2.90	13	22	1	8.90	75.1	22
2.00	1	3.10	17	40	1	9.70	80.0	40
2.00	1	3.50	5	72	1	7.10	68.0	72
1.00	1	3.10	3	19	1	5.50	56.0	19
2.00	1	3.20	9	90	1	8.30	70.9	90
2.00	2	2.70	18	51	1	7.50	74.2	51
1.00	1	3.40	11	23	1	10.80	80.0	23
1.00	1	2.90	24	49	1	10.40	86.0	49
2.00	1	2.70	24	37	1	9.60	76.0	37
2.00	1	3.60	2	23	1	4.20	57.0	23
1.00	1	3.10	9	21	1	8.90	71.5	21
2.00	2	3.90	8	55	1	8.90	70.0	55
1.00	1	3.00	2	48	1	5.50	60.3	48
1.00	2	2.80	4	22	1	4.10	58.0	22
1.00	1	3.40	2	54	1	5.00	62.0	54
1.00	2	2.30	4	17	1	7.50	68.0	17
1.00	2	3.00	12	23	1	9.50	78.1	23
1.00	1	2.90	7	20	1	11.50	70.0	20
2.00	2	4.10	17	38	1	6.50	65.2	38
1.00	2	3.70	4	30	0	5.60	60.0	30
2.00	2	3.10	5	57	1	7.00	69.2	57
1.00	2	3.00	14	24	1	9.50	79.0	24
1.00	1	2.60	5	19	1	7.70	67.0	19
1.00	1	3.00	5	24	1	6.00	69.0	24
1.00	1	2.30	4	23	1	6.80	66.0	23
1.00	2	3.40	4	20	1	6.00	64.0	20
2.00	1	3.00	23	72	1	12.30	86.2	72
1.00	2	3.40	8	24	1	7.30	68.2	24
1.00	1	3.60	11	96	1	9.20	76.0	96
2.00	2	3.00	9	67	1	8.70	72.7	67
1.00	1	3.00	3	23	1	5.50	60.4	23
1.00	2	3.20	9	23	1	10.70	77.7	23
1.00	2	2.80	8	21	1	8.00	71.2	21

2.00	2	3.10	15	60	1	8.50	78.0	60
1.00	2	3.00	12	36	1	8.00	62.0	36
1.00	1	3.30	6	22	1	10.00	73.5	22
1.00	2	2.70	18	49	1	7.30	76.0	49
1.00	1	2.80	2	24	1	5.50	60.5	24
1.00	2	2.80	23	96	1	10.00	88.0	96
1.00	2	2.70	7	21	1	7.40	69.0	21
2.00	1	3.80	13	49	1	9.00	79.0	49
2.00	2	2.40	9	21	1	8.26	67.0	21
2.00	1	3.90	5	49	1	5.10	61.0	49
1.00	1	2.70	5	19	1	7.30	73.4	19
2.00	2	3.10	2	47	1	5.90	57.5	47
1.00	1	3.30	7	23	1	9.80	78.0	23
1.00	2	2.20	11	35	1	7.10	72.0	35
1.00	1	3.00	2	24	1	6.80	62.3	24
2.00	1	3.20	12	36	1	7.90	75.1	36
1.00	1	3.80	9	24	1	11.90	76.5	24
1.00	1	2.70	8	23	1	8.00	71.3	23
1.00	2	2.90	6	22	1	10.50	70.0	22
1.00	1	2.50	6	35	1	6.20	57.5	35
1.00	1	3.10	8	22	1	7.50	67.0	22
1.00	2	2.40	13	48	1	9.20	78.2	48
1.00	2	3.40	10	21	1	8.00	62.5	21
2.00	2	2.40	3	24	1	5.00	65.5	24
2.00	1	3.40	18	53	1	10.30	81.5	53
1.00	2	2.90	6	24	1	6.90	68.3	24
1.00	2	2.70	9	21	1	8.50	77.3	21
1.00	1	3.40	2	24	1	5.20	62.1	24
1.00	1	3.40	5	52	1	7.30	65.5	52
2.00	1	3.10	21	64	1	9.90	81.5	64
2.00	1	2.70	7	23	1	9.90	77.6	23
1.00	1	3.00	15	24	1	9.90	76.7	24
1.00	1	3.30	2	48	1	6.80	60.2	48
2.00	1	3.20	22	49	1	10.10	78.5	49
1.00	1	2.70	4	22	1	6.90	68.4	22
1.00	2	3.00	4	23	1	6.90	61.0	23
2.00	1	3.10	24	60	1	12.40	78.4	60
1.00	2	2.70	2	19	1	6.00	62.5	19
2.00	1	2.50	4	79	1	6.70	61.5	79
1.00	1	2.90	2	23	1	4.20	56.0	23
1.00	2	3.00	24	92	1	12.10	89.0	92
1.00	2	2.80	8	19	1	8.60	64.8	19
1.00	1	3.40	8	22	1	7.70	73.3	22
2.00	2	2.10	21	65	1	9.20	74.5	65
2.00	2	2.60	7	58	1	6.40	64.5	58
1.00	2	2.00	13	22	1	7.10	69.5	22
2.00	2	2.50	8	55	1	7.10	67.3	55

2.00	1	3.20	10	20	1	10.00	79.0	20
2.00	2	2.70	13	38	1	8.00	76.4	38
1.00	2	3.20	11	21	1	9.60	85.0	21
2.00	1	3.50	11	40	1	9.80	72.2	40
2.00	2	2.90	13	51	1	7.20	75.0	51
1.00	2	2.50	3	23	1	5.10	61.0	23
1.00	2	2.80	9	19	1	7.50	71.0	19
2.00	2	3.30	14	84	1	9.00	74.5	84
1.00	1	2.60	5	24	1	7.00	69.5	24
1.00	1	3.30	7	39	1	9.00	70.4	39
2.00	1	3.20	13	41	1	10.20	77.1	41
2.00	2	3.20	10	36	1	8.20	71.0	36
1.00	2	3.10	10	21	1	9.20	76.0	21
1.00	1	2.50	9	22	1	10.50	83.0	22
2.00	1	2.50	3	39	1	6.60	67.0	39
1.00	1	3.30	7	50	1	8.20	67.0	50
2.00	2	3.30	5	66	1	7.10	66.0	66
1.00	1	3.20	11	20	1	9.30	73.0	20
2.00	2	3.00	5	24	1	6.60	63.2	24
2.00	1	3.40	3	73	1	7.40	61.0	73
1.00	1	2.90	9	24	1	10.60	75.9	24
2.00	2	3.00	20	65	1	7.40	82.6	65
2.00	2	3.30	18	48	1	9.50	77.7	48
1.00	2	3.10	2	24	1	6.10	61.1	24
2.00	1	3.30	4	35	1	5.50	55.0	35
2.00	2	2.00	4	22	1	8.50	68.5	22
1.00	2	3.40	9	21	1	7.70	68.6	21
2.00	2	3.20	3	64	1	5.50	60.3	64
2.00	1	3.00	24	86	1	11.30	84.2	86
1.00	2	3.00	2	22	1	6.00	59.5	22
1.00	1	2.80	7	24	1	9.10	82.1	24
1.00	1	2.30	24	79	1	10.10	86.0	79
2.00	1	3.00	24	73	1	11.20	84.0	73
1.00	2	2.80	11	24	1	10.10	74.9	24
2.00	1	3.00	24	60	1	13.20	88.0	60
1.00	1	3.10	12	53	1	9.50	78.1	53
2.00	2	2.60	9	24	1	7.10	69.3	24
2.00	1	3.10	13	66	1	7.70	71.5	66
1.00	1	2.90	3	23	1	4.70	64.2	23
2.00	2	2.90	21	92	1	12.90	74.5	92
2.00	2	2.80	9	22	1	11.30	76.5	22
1.00	2	2.80	6	20	1	8.40	70.5	20
2.00	2	3.40	23	84	1	11.50	91.0	84
2.00	2	2.50	4	24	1	5.80	69.0	24
2.00	1	3.20	2	72	1	4.50	60.2	72
1.00	1	2.50	5	19	1	6.40	69.0	19
2.00	1	2.80	22	72	1	13.50	91.0	72

2.00	2	3.20	24	83	1	10.50	82.0	83
1.00	1	2.70	8	23	1	9.80	78.0	23
1.00	2	3.10	7	21	1	7.50	68.0	21
1.00	1	2.80	4	42	1	8.90	69.5	22
2.00	2	2.50	24	96	1	12.40	95.6	96
2.00	1	2.70	11	23	1	8.50	71.4	23
2.00	1	2.70	14	82	1	9.80	80.0	82
1.00	2	2.70	10	23	1	8.20	73.0	23
2.00	2	3.00	20	74	1	9.10	80.0	74
1.00	2	3.00	8	21	1	7.10	80.0	21
2.00	2	3.00	23	71	1	13.50	80.0	71
2.00	2	2.80	5	23	1	6.70	69.5	23
1.00	2	3.50	9	22	1	9.10	70.5	22
2.00	1	2.60	5	80	1	8.20	69.3	80
1.00	1	3.40	3	24	1	5.90	64.9	24
2.00	2	3.10	24	62	1	10.50	83.5	62
1.00	1	2.50	11	21	1	10.10	78.2	21
2.00	1	3.00	2	37	1	4.80	54.6	37
2.00	2	3.20	6	46	1	7.00	69.2	46
1.00	2	3.00	9	18	1	12.00	78.5	18
1.00	1	3.20	4	23	1	7.70	66.9	23
2.00	1	4.00	24	72	1	11.80	84.7	72
1.00	1	2.60	23	88	1	10.00	82.5	88
1.00	2	2.50	6	21	1	8.00	67.5	21
2.00	2	2.80	21	62	1	12.50	82.5	62
1.00	2	2.30	12	22	1	8.60	70.0	22
2.00	2	2.90	12	40	1	8.00	72.1	40
1.00	2	2.50	11	21	1	7.40	74.1	21
1.00	1	3.50	7	24	1	8.80	70.3	24
1.00	1	3.00	21	44	1	15.40	90.8	44
1.00	2	2.70	13	44	1	8.40	74.0	44
1.00	1	3.00	8	22	1	8.50	70.6	22
1.00	1	4.00	18	61	1	11.70	84.3	61
1.00	2	3.20	9	20	1	8.50	74.5	20
1.00	2	2.80	4	24	1	6.00	68.8	24
2.00	2	2.90	3	47	1	6.70	62.1	47
1.00	2	2.40	3	43	1	6.20	59.8	43
1.00	2	2.20	12	23	1	9.00	80.0	23
2.00	1	3.00	18	72	1	12.60	77.1	72
1.00	2	2.80	4	22	1	5.80	59.6	22
1.00	1	3.50	2	49	1	5.30	59.2	49
1.00	2	2.70	6	24	1	6.40	64.9	24
1.00	1	2.80	3	19	1	7.10	63.1	19
1.00	2	3.00	7	20	1	7.00	73.5	20
1.00	2	2.70	3	23	1	5.60	55.0	23
1.00	2	3.00	3	22	1	6.70	62.0	22
2.00	2	3.20	8	62	1	6.40	68.5	62

1.00	1	3.30	4	22	1	6.80	68.0	22
1.00	1	2.40	4	36	1	6.70	54.5	36
1.00	2	3.10	5	22	1	4.50	60.5	22
1.00	2	3.30	3	53	1	4.60	54.6	53
1.00	1	3.30	12	24	1	7.80	74.1	24
2.00	2	3.00	18	54	1	9.00	81.3	54
1.00	1	2.10	9	19	1	7.60	71.2	19
2.00	1	2.70	9	55	1	7.30	69.0	55
1.00	2	3.00	5	22	1	9.20	70.5	22
1.00	1	2.50	8	62	1	8.90	68.5	62
2.00	1	3.20	2	63	1	5.30	60.3	63
1.00	1	2.80	3	23	1	6.50	59.5	23
1.00	1	3.00	3	24	1	6.30	69.2	24
2.00	1	3.40	5	51	1	7.60	68.5	51

B1IDofmot	Age	B4EDUCAT	B5MaritalS	C1motherC	C2Typeofo	C2aOtherO	C2Didthepı	Agedifferer
1	34	2	1	1	3		1	5
2	25	2	1	1	1		1	5
3	30	3	1	1	1		1	3
4	30	2	1	2	1		1	4
5	33	2	1	1	4		1	6
26	25	2	1	1	3	1	1	6
6	27	4	1	1	4		1	4
7	36	4	1	1	3		1	4
8	29	4	1	1	1		1	6
9	27	3	1	1	3		1	4
10	34	1	1	1	1		1	3
11	21	2	1	1	3		1	6
12	24	1	1	1	1		1	4
13	26	2	1	2	1		1	4
14	29	2	1	1	1		1	6
15	26	2	1	1	1		1	3
16	21	2	1	2	1		1	5
17	24	2	1	1	1		1	4
18	39	3	1	1	4		1	4
19	26	2	1	1	3		1	6
20	23	2	1	1	1		1	6
21	22	2	1	1	1		1	3
22	38	2	1	1	1		1	6
23	42	3	2	1	1		1	3
24	29	2	1	1	5		1	6
25	33	1	1	1	3		1	5
27	40	2	1	1	3		1	5
28	25	2	1	1	1		1	4
29	29	1	1	1	3		1	6
30	30	2	1	1	3		1	4
31	24	1	2	2	3		1	3
32	27	1	2	1	1		1	5
33	30	1	1	1	1		1	6
34	26	1	1	1	3		1	6
35	35	1	1	1	3		1	4
36	30	4	1	1	6	unemploye	1	3
37	28	3	1	1	1		1	6
38	24	2	1	1	6	apprentice	1	3
39	22	2	1	1	5		1	3
40	35	1	1	1	1		1	4
41	32	2	1	1	5		1	5
42	25	2	1	2	1		1	3
43	25	2	1	2	1		1	5
44	35	2	1	1	3		1	6
45	25	1	1	2	3		1	6
46	35	1	1	1	3		1	5

46	25	2	1	2	1		1	5
47	23	2	2	2	1		1	3
48	25	2	1	1	1		1	6
49	24	2	1	1	5		1	6
50	30	1	1	2	1		1	3
53	21	2	1	1	5		1	6
54	42	1	1	1	3		1	4
81	24	2	1	1	5		1	5
82	22	1	1	1	3		1	6
83	37	1	1	1	3		1	4
84	19	2	2	2	6		1	4
85	23	2	2	2	6		1	5
86	41	2	1	1	5		1	6
87	20	1	1	2	1		1	5
88	21	2	1	1	5		1	3
89	36	1	1	1	3		1	5
90	21	2	1	1	5		1	3
91	30	1	1	1	3		1	5
92	26	1	1	1	3		1	5
93	38	1	1	1	1		1	3
94	38	1	1	1	3		1	5
95	40	1	1	1	3		1	6
96	21	1	2	2	1		1	4
97	27	1	1	1	3		1	5
98	30	2	1	1	3		1	3
99	40	2	1	1	3		1	4
100	31	2	1	1	3		1	5
101	24	3	1	1	1		1	5
102	19	2	1	1	3		1	4
103	37	1	1	1	2		1	5
104	29	2	1	1	3		1	3
105	38	1	1	1	3		1	5
106	32	1	1	1	3		1	3
107	38	2	2	1	3		1	6
108	37	1	2	1	2		1	6
109	42	1	1	1	3		1	3
110	42	2	1	1	3		1	5
111	33	3	1	1	4		1	6
112	35	1	1	1	1		1	4
113	31	3	1	1	4		1	5
114	32	2	1	1	2		1	5
115	25	2	1	2	5		1	4
116	26	2	1	1	2		1	5
117	20	2	1	1	3		1	3
118	30	1	1	1	3		1	6
119	28	2	1	1	5		1	4
120	40	4	1	1	5		1	4

121	40	1	1	1	3	1	6
122	25	1	1	2	1	1	3
123	24	3	1	2	1	1	5
124	30	2	1	2	1	1	3
124	30	2	1	2	1	1	5
125	27	1	1	2	6	1	4
126	30	1	1	2	6	1	6
127	20	2	1	1	5	1	3
128	30	1	1	1	3	1	6
129	29	2	1	2	6	1	4
130	22	1	1	1	5	1	5
131	36	1	1	1	3	1	3
132	35	1	1	1	1	1	6
133	37	1	1	1	3	1	5
134	28	1	1	1	3	1	6
135	27	2	1	1	3	1	3
136	40	1	1	1	3	1	4
137	38	1	1	1	3	1	3
138	23	1	1	1	3	1	5
139	19	2	1	2	1	1	3
140	20	2	1	1	3	1	5
141	22	1	1	1	3	1	5
142	36	1	1	1	3	1	3
143	25	1	1	1	1	1	5
144	27	2	1	1	3	1	6
145	39	1	1	1	3	1	4
146	20	2	1	1	1	1	5
147	41	1	1	1	1	1	5
148	29	2	1	1	3	1	3
149	23	2	1	1	1	1	5
150	28	2	1	1	1	1	4
151	26	1	1	1	5	1	4
152	25	2	1	1	1	1	5
153	31	1	1	2	6	1	4
154	30	4	1	1	1	1	6
155	22	1	1	2	1	1	4
156	22	2	1	2	1	1	4
157	35	1	1	2	1	1	5
158	31	2	1	2	1	1	3
159	28	3	1	1	1	1	5
160	28	1	1	1	3	1	3
161	32	1	1	1	3	1	5
162	27	1	1	1	3	1	4
163	26	2	1	1	3	1	4
164	20	2	1	1	5	1	5
165	30	1	1	1	3	1	4
166	30	1	1	1	3	1	3

167	32	2	1	1	5	1	6
168	30	2	1	1	3	1	3
169	30	2	1	1	1	1	5
170	20	2	1	2	6	1	5
171	32	1	1	1	1	1	4
172	32	2	1	1	1	1	4
173	39	2	1	1	1	1	4
174	24	3	2	1	1	1	3
175	26	2	1	2	5	1	4
176	32	3	1	2	1	1	6
177	35	3	1	1	1	1	4
178	25	3	1	2	6	1	5
179	28	3	1	1	6	1	5
180	34	1	1	1	3	1	4
181	40	1	1	1	1	1	6
182	26	2	1	1	3	1	4
183	39	1	1	1	3	1	5
184	36	2	1	1	1	1	3
185	28	1	1	1	1	1	6
186	39	1	1	1	3	1	4
187	34	3	1	1	1	1	5
188	34	2	1	1	3	1	3
189	43	1	1	1	3	1	4
190	22	2	1	1	3	1	6
191	41	1	1	1	1	1	6
192	20	2	1	1	3	1	4
193	39	2	1	1	3	1	6
194	21	2	1	1	3	1	4
195	24	1	1	1	3	1	5
196	28	2	1	1	1	1	3
197	30	1	1	1	3	1	5
198	22	2	2	1	3	1	4
199	30	2	1	1	3	1	5
200	23	2	1	1	3	1	6
201	20	2	1	1	3	1	4
202	25	1	1	1	3	1	4
203	23	2	1	1	3	1	3
204	29	1	1	1	3	1	5
206	26	1	1	1	3	1	4
207	32	1	1	2	3	1	5
208	35	1	1	1	1	1	3
209	23	1	1	2	1	1	5
210	23	1	1	1	3	1	3
22	31	1	1	2	1	1	4
212	35	1	1	2	1	1	6
213	28	1	1	1	3	1	5
25	27	1	1	1	3	1	4

215	32	2	1	2	1		1	5
215	30	2	2	1	1		1	3
216	20	2	1	1	1		1	3
217	35	2	1	1	4		1	4
218	26	2	1	1	5		1	5
219	22	2	1	1	3		1	5
221	36	1	1	1	3		1	3
222	23	2	1	1	3		1	5
223	30	2	1	1	1		1	6
224	45	1	1	1	3		1	3
225	30	1	1	1	3		1	6
226	26	2	1	1	3		1	3
227	23	2	1	1	5		1	5
228	21	2	1	1	3		1	3
229	30	2	1	1	1		1	5
230	27	2	1	1	3		1	5
231	35	1	1	1	3		1	4
369	32	2	1	2	4		1	6
244	40	1	1	1	3		1	3
245	35	1	1	2	1		1	6
246	26	1	1	1	3		1	6
247	30	2	1	1	3		1	4
248	30	2	1	1	1		1	6
249	26	1	1	1	3		1	4
250	32	4	1	2	1		1	5
251	31	3	1	1	1		1	3
252	28	3	1	2	1		1	4
253	20	1	1	1	1		1	5
254	31	4	1	1	4		1	3
255	30	2	1	1	1		1	6
414	24	3	1	1	1		1	3
257	25	2	1	1	1		1	6
258	27	2	1	1	5		1	3
259	27	3	1	2	6		1	3
260	29	2	1	1	3		1	6
261	28	2	1	1	3		1	4
262	26	2	1	1	5		1	6
263	27	3	1	1	1		1	6
264	25	2	1	1	6	Dressmake	1	4
265	35	2	1	1	3		1	3
466	20	2	1	1	3		1	5
266	28	2	1	1	3		1	4
267	21	3	1	1	3		1	4
268	35	1	1	1	3		1	5
269	23	2	1	1	3		1	4
270	24	3	1	1	3		1	6
271	24	2	1	1	3		1	6

272	21	2	1	2	1		1	4
273	26	2	1	1	3		1	4
274	23	2	1	1	3		1	3
275	35	2	1	1	3		1	6
276	24	2	1	1	3		1	3
277	20	2	1	1	6	Senstress	1	5
278	25	2	1	1	3		1	5
279	23	1	1	1	3		1	4
280	35	2	1	1	1		1	5
281	37	3	1	2	1		1	4
282	25	3	1	1	1		1	6
283	37	2	1	1	1		1	4
284	27	3	1	1	1		1	6
285	23	2	1	1	1		1	4
286	25	2	1	1	3		1	4
287	26	2	1	1	1		1	5
288	23	2	1	1	3		1	3
289	28	1	1	1	3		1	6
290	37	1	1	1	3		1	3
291	42	1	1	1	3		1	5
292	45	2	1	1	3		1	4
293	31	1	1	2	3		1	3
294	40	1	1	1	3		1	4
295	30	2	1	1	3		1	6
296	25	1	1	1	3		1	4
297	26	2	1	1	3		1	3
298	20	1	1	1	3		1	5
299	29	2	1	1	3		1	3
456	38	1	1	1	3		1	6
320	32	2	2	2	1		1	4
95	40	1	1	1	1		1	6
115	32	2	1	2	3		1	4
427	38	1	1	2	3		1	5
309	28	2	1	1	3		1	4
311	21	2	1	1	3		1	4
313	32	2	1	1	1		1	4
314	32	1	1	1	3		1	5
315	29	2	1	1	5		1	4
316	20	2	1	1	1		1	4
317	32	1	1	1	1		1	6
318	22	3	1	1	1		1	5
319	24	3	1	1	1		1	3
320	32	2	2	2	3		1	5
321	25	2	1	1	1		1	6
329	34	2	1	1	1		1	4
322	33	2	1	1	1		1	5
323	30	2	1	1	3		1	5

324	34	1	1	1	6	Smestrees	1	3
325	29	2	1	1	3		1	6
326	31	2	1	1	6	Hairdresser	1	4
327	32	4	1	1	6	Caterer	1	5
328	31	3	1	1	1		1	6
329	34	2	1	1	1		1	4
330	27	2	1	1	2		1	4
331	24	1	1	1	1		1	5
332	38	2	1	1	1		1	6
333	23	2	1	1	1		1	5
334	24	2	1	2	3		1	4
335	27	2	1	1	1		1	6
336	23	2	1	1	1		1	4
337	24	2	1	1	1		1	5
338	24	2	1	1	3		1	4
339	34	1	1	1	1		1	6
340	35	4	1	1	4		1	6
341	25	2	1	1	1		1	4
342	32	2	1	1	4		1	5
343	23	2	1	1	1		1	5
344	25	2	1	1	1		1	3
345	32	2	1	1	1		1	6
346	29	4	1	1	4		1	5
347	35	2	1	1	1		1	3
348	22	2	1	1	1		1	4
349	27	2	1	1	1		1	5
350	24	2	1	1	3		1	3
351	27	2	1	2	1		1	6
352	41	4	1	2	6		1	5
353	24	2	1	1	1		1	5
354	34	2	1	2	3		1	4
355	32	3	1	2	3		1	4
356	30	4	1	2	3		1	5
357	35	1	1	1	1		1	6
358	31	2	1	1	1		1	3
359	28	2	1	1	1		1	6
360	23	2	1	1	1		1	3
361	27	2	1	1	1		1	4
362	30	1	1	2	3		1	4
363	25	2	1	1	6	semestress	1	6
364	31	2	1	1	1		1	6
365	32	2	1	1	3		1	3
366	26	2	1	2	3		1	5
367	35	1	1	1	3		1	4
368	27	3	1	1	1		1	5
369	32	2	1	2	3		1	4
370	36	1	1	2	3		1	3

371	27	3	1	2	6		1	6
372	27	2	1	1	1		1	3
373	32	2	1	1	3		1	5
374	22	2	1	1	1		1	5
375	23	1	1	1	1		1	4
376	29	2	1	1	1		1	6
377	26	3	1	1	4		1	5
378	30	2	1	1	6	hairdresser	1	4
379	30	2	1	1	5		1	5
380	22	3	1	2	6		1	4
381	26	2	1	1	4		1	3
382	26	2	1	1	1		1	5
383	26	2	1	1	1		1	3
384	26	2	1	1	5		1	3
385	35	2	1	1	5		1	4
386	27	2	1	1	5		1	5
387	33	3	1	1	5		1	6
388	28	2	1	1	5		1	3
389	28	2	1	1	5		1	6
390	33	1	1	1	3		1	5
391	25	3	1	1	3		1	4
392	27	3	1	1	4		1	5
393	26	2	1	2	4		1	5
394	29	2	1	1	1		1	4
395	28	2	1	1	1		1	3
396	35	2	1	1	1		1	6
397	29	2	1	1	1		1	5
398	41	1	1	1	1		1	3
399	25	2	1	1	1		1	6
400	26	2	1	1	1		1	2
401	24	1	1	1	1		1	4
402	26	3	1	1	4		1	3
403	28	1	1	1	1		1	5
404	28	2	1	2	3		1	5
405	27	2	1	2	3		1	5
406	33	4	1	1	1		1	4
407	28	4	1	2	3		1	3
408	27	4	1	1	4		1	4
409	34	3	1	1	4		1	4
410	30	2	1	1	1		1	3
411	41	1	1	2	3		1	6
412	26	4	1	1	4		1	4
413	28	3	1	2	3		1	6
414	24	3	1	1	1		1	6
415	20	2	1	1	1		1	4
416	43	1	1	1	1		1	4
417	31	4	1	1	4		1	3

418	29	4	1	1	4		1	6
419	32	4	1	1	4		1	5
420	24	4	1	1	4		1	3
421	31	1	1	1	1		1	5
422	20	2	1	2	3		1	4
423	23	4	1	1	4		1	6
424	25	2	1	1	1		1	3
425	40	1	1	1	1		1	5
426	25	2	1	2	3		1	3
427	38	1	1	2	3		1	5
428	42	1	1	1	1		1	3
429	40	1	1	1	1	subsistence	1	5
430	36	1	1	1	3		1	4
431	36	1	1	1	3		1	5
432	25	1	1	1	3		1	4
433	30	2	1	2	3		1	5
434	35	2	1	1	3		1	4
435	29	4	1	1	4		1	4
436	35	2	1	1	3		1	3
437	27	2	1	1	3		1	5
438	30	3	2	1	3		1	3
439	31	3	2	1	6	semestress	1	5
440	20	2	1	1	6	hairdresser	1	3
441	25	2	1	1	6	hairdresser	1	4
442	32	3	1	1	1		1	5
443	21	2	1	2	1		1	4
444	33	2	1	1	3		1	3
445	25	2	1	1	6	semestress	1	4
446	22	2	1	2	6		1	5
447	32	3	2	1	1		1	6
448	26	2	1	1	3		1	4
449	34	3	1	1	3		1	4
450	22	2	1	1	3		1	5
451	21	2	1	1	3		1	5
452	41	1	1	1	3		1	3
453	20	2	1	1	3		1	4
454	22	2	1	1	1		1	6
455	29	3	2	1	3		1	3
456	38	1	1	1	3		1	6
457	22	2	1	1	3		1	4
458	34	2	1	1	3		1	6
259	28	1	1	1	3		1	3
460	20	2	1	1	3		1	3
461	38	1	1	1	3		1	6
462	22	1	1	1	1		1	6
463	34	1	1	1	3		1	3
464	31	2	1	1	1		1	5

465	28	1	1	1	3		1	3
467	37	2	1	1	3		1	5
468	34	1	1	1	3		1	3
469	30	2	1	1	3		1	5
470	31	3	1	1	3		1	5
471	32	1	1	1	3		1	4
472	35	1	1	1	3		1	3
473	39	2	1	1	3		1	6
474	26	4	1	2	3		1	4
475	32	1	1	1	3		1	3
476	24	1	1	1	3		1	5
477	30	2	1	1	3		1	5
478	26	2	1	1	3		1	3
479	43	1	1	1	1		1	3
480	24	1	1	1	1		1	5
481	22	2	1	1	3		1	5
482	28	1	1	1	3		1	6
483	33	1	1	1	3		1	3
484	22	2	1	1	3		1	4
485	20	2	1	1	3		1	6
486	29	1	1	1	3		1	4
487	36	2	1	1	5		1	6
488	35	2	1	1	3		1	5
489	23	2	1	1	3		1	4
490	32	1	1	1	3		1	5
491	33	1	1	1	3		1	6
492	26	2	1	1	3		1	3
493	24	2	1	1	3		1	6
494	26	2	1	1	3		1	6
495	24	2	1	1	3		1	3
496	34	1	1	1	3		1	4
497	29	2	1	1	3		1	6
498	37	4	1	1	1		1	6
499	30	1	1	1	1		1	4
500	21	2	1	1	1		1	6
501	23	2	1	1	3		1	6
502	40	1	1	1	1		1	3
503	23	2	1	1	1		1	6
504	30	1	1	1	1		1	4
505	35	1	1	1	1		1	6
506	38	1	1	1	3		1	4
507	41	1	1	1	3		1	3
508	32	1	1	1	3		1	6
509	27	3	2	1	3		1	4
510	28	1	1	2	3		1	6
511	35	1	1	1	3		1	3
512	25	2	1	1	3		1	6

513	27	2	1	1	3		1	6
514	35	1	1	1	1		1	4
515	27	1	1	2	4		1	4
516	30	1	1	2	3		1	5
517	30	2	1	1	3		1	6
518	23	2	1	1	4		1	4
519	31	1	1	1	1		1	6
520	26	2	1	1	3		1	4
521	43	2	1	1	3		1	6
521	22	2	1	1	3		1	3
523	39	1	1	1	3		1	6
524	30	2	1	1	1		1	6
525	33	1	1	1	1		1	3
526	45	1	1	1	5		1	6
527	32	2	1	1	5		1	4
528	30	2	1	1	3		1	6
529	32	2	1	1	1		1	3
530	32	1	1	1	3		1	5
531	24	1	1	1	1		1	5
532	38	1	1	1	1		1	3
533	23	2	1	1	3		1	4
534	40	1	1	1	3		1	6
535	35	1	1	1	3		1	6
536	21	2	1	1	3		1	4
537	40	1	1	1	3		1	6
538	30	2	1	1	1		1	3
539	40	2	1	1	3		1	5
540	30	1	1	1	1		1	3
541	43	1	1	1	1		1	4
542	31	1	1	1	1		1	5
543	33	4	1	1	4		1	5
544	25	3	1	1	1		1	3
145	21	1	1	1	1		1	6
146	30	4	1	1	4		1	3
147	28	2	1	1	5		1	4
548	23	1	1	1	3		1	5
149	32	4	1	1	4		1	5
550	26	1	2	1	1		1	4
551	38	2	1	1	3		1	6
552	25	2	2	1	5		1	3
553	30	2	1	1	5		1	5
554	34	4	1	1	4		1	4
555	28	2	1	1	1		1	3
556	27	2	1	1	1		1	3
557	35	2	1	1	5		1	4
558	29	2	1	1	1		1	3
559	31	2	1	1	1		1	6

560	28	2	1	1	1		1	3
561	28	2	1	1	5		1	5
562	24	1	1	1	4		1	3
563	30	1	1	1	5		1	5
564	26	2	1	1	1		1	4
565	28	4	1	1	4		1	5
566	25	2	1	2	3		1	3
567	25	2	1	1	3		1	6
568	39	2	1	1	1		1	3
569	30	4	1	1	4		1	6
570	33	4	1	1	4		1	6
571	34	3	1	1	4		1	4
572	25	4	1	1	1		1	4
223	30	2	1	1	1		1	5

D1Whatistl	Gender	D3Dateofb	D5twhatsta	D6BREASTF	Contracept	E2IfNOwhy	E3Ifyeswha	E4Iftraditoi
rhanner	2	2-Mar-12	#NULL!	26	1		2	
techiga joa	2	11-Apr-14	#NULL!	30	1		2	
noah akany	1	11-Feb-16	#NULL!	12	1		2	
kelvin enoc	1	17-Dec-16	#NULL!	14	2	due to fina	#NULL!	
maduna sa	2	12-Aug-12	#NULL!	24	1		2	
kofi	1	########	#NULL!	24	1		2	
kudus inusa	1	21-Apr-16	#NULL!	15	2		#NULL!	
	1	28-Dec-16	#NULL!	11	2	because I lo	#NULL!	
godgon lag	1	19-Jul-11	#NULL!	24	1		2	
adagwine v	2	1-Jan-16	#NULL!	24	1		2	
melody aba	2	1-Mar-16	#NULL!	10	1		2	
delfiria asa	2	########	#NULL!	24	2	no reason g	#NULL!	
sunday kwo	1	3-Nov-15	#NULL!	15	2		#NULL!	
emma koliy	1	8-Nov-15	#NULL!	24	2	no reason g	#NULL!	
eunie wew	2	18-Aug-05	#NULL!	24	1		2	
mepiah we	2	12-Feb-16	#NULL!	11	1		2	
wepiah ayil	2	29-Aug-14	#NULL!	24	2		#NULL!	
Adyei emm	1	7-Dec-16	#NULL!	12	1		2	
Richard bas	1	19-Nov-15	#NULL!	16	2		#NULL!	
sabastian	1	5-Aug-10	#NULL!	24	1		2	
safia womo	2	24-Feb-11	#NULL!	26	1		2	
theodora a	2	20-Feb-16	#NULL!	8	1		2	
geofrey	1	########	#NULL!	27	2		#NULL!	
cyndy adeb	2	2-Feb-16	#NULL!	13	1		2	
donald adiy	1	28-Jan-11	#NULL!	14	1		2	
dorothy asa	2	7-Jul-10	#NULL!	25	2		#NULL!	
angel asaw	2	9-Nov-13	#NULL!	24	2	i don't wan	#NULL!	
dorothy	2	20-Nov-15	#NULL!	10	1		2	
sabina abiir	2	10-Nov-09	#NULL!	36	1		2	
prince	1	20-Apr-16	#NULL!	24	1		2	
macel ason	2	2-Mar-16	#NULL!	12	2		#NULL!	
amintenga	1	26-Nov-13	#NULL!	15	1		2	
foster atan	1	1-Apr-11	#NULL!	27	1		2	
patience	2	20-Jun-13	#NULL!	25	2		#NULL!	
Issabella	2	3-Nov-15	#NULL!	13	2		#NULL!	
wilfred	1	12-Apr-16	#NULL!	8	1		2	
noah kumu	1	26-Jan-14	#NULL!	29	1		2	
1shmael ac	1	20-Jun-13	#NULL!	14	1	2	2	
Ida awepor	2	2-Apr-16	#NULL!	11	2		#NULL!	
eugiene ma	1	4-Nov-15	#NULL!	12	1		2	
modicai aw	1	25-Nov-11	#NULL!	26	1		2	
prince adol	1	16-Apr-16	#NULL!	9	1		2	
prince adol	1	16-Jul-14	#NULL!	12	1		2	
atap0re A	1	9-Jan-11	#NULL!	24	1		2	
Mavis awal	2	22-Jul-12	#NULL!	30	1		2	
jeremiah	1	1-Apr-16	#NULL!	24	2		#NULL!	

Mavis Awa	2	22-Jul-12	#NULL!	30	1		2	
osman ali	1	17-Feb-16	#NULL!	13	1		2	
Daniel akol	1	8-Feb-13	#NULL!	24	2		#NULL!	
Frank atore	1	5-Feb-16	#NULL!	24	2		#NULL!	
Jeremia Azı	1	2-Dec-16	#NULL!	11	2		#NULL!	
Morris	1	24-Aug-12	#NULL!	24	2		#NULL!	
solomon ap	1	11-Dec-15	#NULL!	10	1		2	
ibrahim	1	28-Apr-15	#NULL!	12	2		#NULL!	
foster yaro	1	6-Feb-11	#NULL!	25	2		#NULL!	
sandra	2	28-Nov-15	#NULL!	13	1		2	
nana asakir	1	4-Nov-15	#NULL!	14	2		#NULL!	
	1	30-Nov-14	1	13	2		#NULL!	
doris ayisib	2	5-Oct-13	#NULL!	26	2		#NULL!	
patrick sum	1	25-Dec-12	#NULL!	30	1		2	
gilbert ano	1	5-Jul-14	#NULL!	7	1		2	
roger	1	########	#NULL!	27	2		#NULL!	
fidams awi	2	7-Apr-16	#NULL!	11	1		2	
priscila	2	10-Oct-14	#NULL!	24	2		#NULL!	
rita anagbi	2	16-Apr-12	#NULL!	26	2	he travelec	#NULL!	
paba queer	2	22-Feb-16	#NULL!	15	2	husband in	#NULL!	
atingane lir	1	26-Nov-14	#NULL!	26	2		#NULL!	
akentoa	1	12-Mar-13	#NULL!	24	2		#NULL!	
nancy	2	30-Dec-15	#NULL!	12	1		1	breasfeedir
suzana aka	2	1-Jun-14	#NULL!	24	2		#NULL!	
elisha asilal	1	9-Mar-16	#NULL!	10	2	wasnt living	#NULL!	
sophia	2	28-Dec-15	#NULL!	14	2		#NULL!	
victor	1	13-Nov-14	#NULL!	24	2	it causes si	#NULL!	
atobakew a	2	########	#NULL!	21	2		#NULL!	
anutua aye	2	10-Nov-15	#NULL!	13	2	causes infe	#NULL!	
portia	2	15-Oct-13	#NULL!	18	2	nothig	#NULL!	
benedicta	2	10-Apr-16	#NULL!	12	1		2	
1	1	20-Nov-14	#NULL!	26	2	nothing	#NULL!	
joyce	2	22-Apr-16	#NULL!	13	1	2	2	
bansford	1	2-Mar-11	#NULL!	28	1	2	2	
gideon	1	########	#NULL!	30	2	he wont ag	2	
martha	2	11-Mar-16	#NULL!	10	1		2	
evans	1	2-Mar-13	#NULL!	24	2		#NULL!	
akampati	2	19-Sep-12	#NULL!	24	1		2	
maxwel	1	28-Nov-15	1	12	2		#NULL!	
asaah godv	1	10-Jan-13	#NULL!	24	2	i dont want	#NULL!	
demian asa	1	21-Apr-13	#NULL!	24	1		2	
elen	2	15-Nov-15	1	13	1		2	
lovia azii	2	8-Aug-14	#NULL!	24	1		2	
abena mya	2	19-Jan-16	#NULL!	9	2		#NULL!	
prince agar	1	31-Oct-11	#NULL!	24	2	i dontwant	#NULL!	
atinga ateiv	2	10-Nov-15	#NULL!	31	1		2	
joshua awu	1	15-Dec-15	#NULL!	14	2		#NULL!	

james atazı	1	22-Feb-13	#NULL!	20	2		#NULL!
felicia abug	2	18-Feb-16	#NULL!	7	2		#NULL!
Azure chris	2	17-Oct-13	#NULL!	25	1		2
Janet Akan	2	########	#NULL!	11	2		#NULL!
janet	2	20-Jun-13	#NULL!	24	2		#NULL!
Asumbono	1	1-Dec-15	#NULL!	12	2		#NULL!
maxwell	1	2-Dec-12	#NULL!	30	2		#NULL!
Joyce	2	23-Apr-16	#NULL!	7	2		#NULL!
Blessing	2	13-Apr-12	#NULL!	24	2	Husband liv	#NULL!
Benedicta	2	26-Dec-15	#NULL!	24	1		2
Ismael	1	2-Jun-13	#NULL!	30	1		2
Adombire	1	12-Mar-15	#NULL!	11	1		2
Anuse	2	3-May-11	#NULL!	30	2	Wasn't livir	#NULL!
Christabel	2	2-Jan-14	#NULL!	28	2	living separ	#NULL!
matilda	2	10-Jun-11	#NULL!	26	2		#NULL!
Dorothy	2	26-Apr-16	#NULL!	10	1		2
issabela	2	20-Nov-15	#NULL!	8	1		2
	1	27-Jan-15	#NULL!	13	2		#NULL!
SAviour	1	29-Aug-12	#NULL!	24	2		#NULL!
sampson	1	########	#NULL!	10	2		#NULL!
Roland	1	20-Jul-13	#NULL!	24	2		#NULL!
Jeremiah	1	6-Sep-12	#NULL!	24	2		#NULL!
Achaawine	1	28-Jan-15	#NULL!	12	2		#NULL!
Awinpoka	2	5-Nov-12	#NULL!	25	2	not interes	#NULL!
Nicholas	1	6-Apr-12	#NULL!	34	2	Nothing	#NULL!
Zidina	2	24-Nov-15	#NULL!	11	2		2
Rukaya	2	10-Jan-15	#NULL!	17	2	pregnancy	#NULL!
Edmund	1	9-Jan-14	#NULL!	30	2	nothing	#NULL!
Samuela	2	26-Feb-16	#NULL!	14	1		2
Abigail	2	25-Oct-12	#NULL!	24	1		2
Adinam	1	22-Dec-15	#NULL!	9	1		2
Derick	1	16-Dec-15	#NULL!	14	1		2
Evans	1	7-Oct-13	#NULL!	30	1		2
Angela	2	29-Nov-15	#NULL!	12	2	nothing	#NULL!
Nillu	2	2-Jul-10	#NULL!	24	1		2
Ayamba	1	9-Aug-13	#NULL!	27	2		#NULL!
Lovia	2	6-Dec-16	2	12	2		#NULL!
Dominic	1	9-Feb-13	#NULL!	24	1		2
Stephen	2	1-Mar-16	#NULL!	13	2		#NULL!
christian	1	9-Nov-13	#NULL!	24	1		2
Calvin	1	1-Mar-16	#NULL!	7	1		2
Mabel	2	7-Mar-14	#NULL!	24	1		2
Bright	2	21-Nov-15	#NULL!	16	1		2
Mary	2	24-Dec-15	#NULL!	14	1		2
Asobila	2	30-Jul-14	#NULL!	24	1		2
Agoah	1	10-Dec-16	#NULL!	12	2		#NULL!
Martin	1	########	#NULL!	10	1		2

Anaba	1	11-Dec-10	#NULL!	24	2	i dont want	#NULL!	
litucia	2	24-Mar-15	#NULL!	11	2	not willing	#NULL!	
Blessing	2	27-Feb-14	#NULL!	25	1		2	
imoro	1	3-Jun-14	#NULL!	18	1		2	
kofi	1	1-Mar-14	#NULL!	12	2		#NULL!	
Douglas	1	6-Dec-16	#NULL!	15	1		2	
Daniela	2	15-Nov-15	#NULL!	16	1		2	
samuel	1	7-Mar-15	#NULL!	12	1		2	
paschal	1	21-Nov-15	#NULL!	6	1		1	calender m
Joshua	1	3-Jan-05	#NULL!	24	1		2	
Haruna	1	25-Nov-15	#NULL!	15	1		2	
sadia	2	1-Jan-13	#NULL!	24	2	nothing	#NULL!	
Alberta	2	15-Oct-13	#NULL!	20	2	side effects	#NULL!	
Jerry	1	11-Nov-15	#NULL!	9	1		2	
Abagna	1	29-Mar-12	#NULL!	29	2	nothing	#NULL!	
macurius	1	17-Jan-16	#NULL!	24	2	nothing	#NULL!	
Nchoyure	1	29-Mar-12	#NULL!	26	2	nothing	#NULL!	
	2	27-Feb-16	#NULL!	12	1		2	
richard	1	25-Sep-09	#NULL!	18	2	nothing	#NULL!	
ruth	2	12-Dec-15	#NULL!	11	1		2	
Slyvester	1	19-Aug-14	#NULL!	24	1		2	
Helen	2	20-Apr-16	#NULL!	10	2	nothing	#NULL!	
joshua	1	21-Dec-15	#NULL!	14	1		2	
mauricia	2	14-Oct-12	#NULL!	26	2	nothing	#NULL!	
Bright	1	6-Aug-10	#NULL!	36	1		2	
	2	20-Dec-15	#NULL!	13	2		#NULL!	
Jennifer	2	20-Mar-10	#NULL!	27	1		2	
kingley	1	29-Nov-15	#NULL!	14	2		#NULL!	
nancy	2	31-Aug-13	#NULL!	24	1		2	
saviour	1	9-Mar-16	#NULL!	11	1		2	
Gideon	1	########	#NULL!	28	1		2	
theophilus	1	8-Nov-15	#NULL!	12	2		#NULL!	
Blessing	2	12-Oct-17	#NULL!	28	1		2	
Prince	1	1-Jun-11	#NULL!	24	2	nothing	#NULL!	
Godfred	1	20-Dec-15	#NULL!	14	2	nothing	#NULL!	
justice	1	20-Feb-16	#NULL!	24	1		2	
Stephen	1	########	#NULL!	9	2		#NULL!	
Hannah	2	8-Nov-14	#NULL!	18	2		#NULL!	
lovia	2	14-Nov-15	#NULL!	15	2		#NULL!	
willifred	1	24-Feb-14	#NULL!	24	2	was divorc	#NULL!	
millicent	2	9-Dec-15	#NULL!	14	1		2	
portia	2	11-Jul-14	#NULL!	24	2		#NULL!	
maxwell	1	11-Feb-16	#NULL!	8	1		2	
philomina	2	27-Nov-15	#NULL!	11	2	didnt have	#NULL!	
evelyn	2	20-Feb-10	#NULL!	36	2	ever had a	#NULL!	
Elijah	1	5-Sep-14	#NULL!	28	1		2	
Gabriel	1	9-Dec-15	#NULL!	11	2		#NULL!	

magdalene	2	2-Nov-13	#NULL!	24	2		#NULL!	
Romina Ata	2	11-Jun-16	#NULL!	13	1		2	
Fancy Akay	1	9-Jan-12	#NULL!	10	2		#NULL!	
Barthy Aka	2	9-Dec-15	#NULL!	9	1		2	
Blesseng Ar	2	12-Apr-13	#NULL!	24	1		2	
Emmanuell	2	25-Dec-14	#NULL!	18	1		2	
Bugawe	1	11-Feb-16	#NULL!	13	1		2	
Welaga Sal	2	########	#NULL!	26	2		#NULL!	
Anuboom	2	30-Aug-13	#NULL!	27	1		2	
Asagiba W	1	30-Jan-16	#NULL!	10	1		2	
Kumangtio	1	20-Feb-12	#NULL!	17	2		#NULL!	
Kuyibanyim	2	25-Feb-16	#NULL!	12	2		#NULL!	
Abongidew	2	4-Jun-13	#NULL!	24	1		2	
Asubataane	2	26-Mar-16	#NULL!	12	2		#NULL!	
0phelia Baj	2	31-Aug-14	#NULL!	24	1		2	
Ernest	1	21-Jul-13	#NULL!	20	2		#NULL!	
Wese	1	30-Nov-15	#NULL!	13	1		2	
sivea	2	25-Oct-03	#NULL!	24	1		1	
Kaduah Kw	2	21-Feb-16	2	12	2		#NULL!	
Regiaald Ac	1	11-Nov-12	#NULL!	26	1		2	
Bapinwo	1	12-Dec-10	#NULL!	24	1		2	
Philomina I	2	20-Nov-15	#NULL!	15	1		2	
Benedecta	2	20-Nov-11	#NULL!	26	2		#NULL!	
Matilda Aje	2	11-Dec-15	#NULL!	11	1		2	
Shennel	2	29-Dec-14	#NULL!	18	1		1	Menstrual
juliet amey	2	4-Apr-16	#NULL!	12	2	I wanted tc	#NULL!	
	2	18-Dec-15	#NULL!	10	2		#NULL!	
Salam Mun	1	15-Jun-13	#NULL!	24	1		2	
Lordlugard	1	4-Apr-16	#NULL!	11	1		2	
Justice Wei	1	17-Dec-10	#NULL!	26	1		2	
Stanley	1	28-Feb-16	#NULL!	14	1		2	
Emmanuel	1	########	#NULL!	19	1		2	
Jessica Amo	2	10-Apr-16	#NULL!	26	1		1	menstrual
Elenor Akar	2	27-Mar-16	#NULL!	10	1		2	
Hager Awir	2	2-Jul-10	#NULL!	24	1		2	
Theophelus	1	30-Dec-15	#NULL!	13	1		2	
Jonas Wen	1	13-Jan-12	#NULL!	16	1		2	
Alberta	2	17-Oct-08	#NULL!	24	2	Nothing	#NULL!	
Joel	1	21-Dec-15	#NULL!	12	1		2	
Apana	2	30-Jan-16	#NULL!	10	2		#NULL!	
ayidana ack	1	5-Jun-13	#NULL!	29	1		2	
Tibiru Adur	1	18-Sep-16	#NULL!	28	2		#NULL!	
LIONCY WE	2	18-Dec-15	#NULL!	14	2		#NULL!	
Enestina K	1	28-Dec-12	#NULL!	24	2		#NULL!	
Gabriel Aya	1	10-Nov-15	#NULL!	11	1		2	
Joseph Aku	1	28-Jul-13	#NULL!	16	1		2	
Bismark	1	########	#NULL!	37	2	My husban	#NULL!	

Olivia	2	28-Nov-15	#NULL!	10	2	I do not kn	#NULL!
Michael	1	10-Dec-15	#NULL!	13	2		#NULL!
Naomi	2	26-Jan-16	#NULL!	24	2	Iwas not m	#NULL!
Wepaare	1	30-Nov-09	#NULL!	25	2		#NULL!
Balolugu	2	30-Apr-16	#NULL!	12	1		2
Rebecca	2	########	#NULL!	25	1		2
Burawuven	2	23-Aug-13	#NULL!	30	2	Nothing	#NULL!
rahinatu	2	20-Dec-15	#NULL!	12	2	Nothing	#NULL!
Wedadeba	1	30-Nov-13	#NULL!	26	2		#NULL!
	1	1-Dec-15	#NULL!	12	2	IShe did no	#NULL!
Lilibert	2	8-May-10	#NULL!	24	1		2
Gilbert	1	8-Dec-15	#NULL!	9	2	Nothing	#NULL!
Osbert	1	########	#NULL!	19	1		2
Kolalam	2	1-Mar-15	#NULL!	10	2	It did not k	#NULL!
Wenawom	1	8-Dec-15	#NULL!	8	1		2
Christiana	2	13-Nov-13	#NULL!	24	2	My husban	#NULL!
Kadua Edm	2	1-Nov-15	#NULL!	14	2	Nothing	#NULL!
Afia	2	26-Jun-11	#NULL!	36	2	Nothing	#NULL!
Clinton Kw	1	12-Dec-15	#NULL!	14	2		#NULL!
Monday Ak	1	30-Nov-13	#NULL!	36	2		#NULL!
Tigase	1	30-Nov-15	#NULL!	12	1		2
Bebelanwe	2	20-Dec-15	1	7	2	Husbad has	#NULL!
Batachem I	2	10-Nov-14	#NULL!	24	2	No reason	#NULL!
Yorose We	1	20-Jun-12	#NULL!	36	2	Husband tr	#NULL!
Webalora Y	1	11-Feb-16	#NULL!	26	1		2
Songose W	1	1-Nov-15	#NULL!	12	2	No reason	#NULL!
Kwodama I	1	10-Nov-14	#NULL!	24	2	No reason	#NULL!
Judth Seizu	2	22-Jan-16	#NULL!	6	1		2
aziseri agar	1	26-Jun-11	#NULL!	24	1		2
Wisdom Ak	1	20-Dec-16	#NULL!	16	2	Nothing	#NULL!
akentoa	1	12-Mar-13	#NULL!	24	2		#NULL!
elen	2	13-Dec-15	2	10	1		2
babalewe a	1	11-Oct-13	#NULL!	24	1		2
Webalaibal	1	1-Jan-13	#NULL!	30	1		2
Claudia	2	1-Nov-15	#NULL!	12	2	Nothing	#NULL!
Gerald Juju	1	4-Dec-15	#NULL!	13	2	Nothing	#NULL!
Asenawine	1	20-Jun-12	#NULL!	30	1		2
Edmond Ak	1	20-Dec-15	#NULL!	2	1		2
Wetagea D	1	10-Nov-15	#NULL!	8	2	Nothing	#NULL!
Israel Agye	1	30-Oct-11	#NULL!	24	2		#NULL!
Abigail	2	30-Oct-13	#NULL!	22	2	No reason	#NULL!
Augustine	2	30-Jan-16	#NULL!	9	2	I got pregn	#NULL!
Wisdom Ak	1	12-Feb-14	#NULL!	20	2	Nothing	#NULL!
Emmanuel	1	3-Nov-11	#NULL!	24	1		2
Amina	2	27-Nov-15	#NULL!	11	2	I had spont	#NULL!
Rashmas Cl	1	3-Sep-13	#NULL!	24	2		#NULL!
Peter	1	30-Oct-13	#NULL!	18	1		2

Saviour	1	30-Apr-16	#NULL!	18	2		#NULL!	
Paulina Ana	2	########	#NULL!	24	1		1	menstrual
Rhoda Wer	2	30-Dec-15	#NULL!	13	2		#NULL!	
Ephrainm	1	26-Feb-14	#NULL!	24	2	Nothing	#NULL!	
Rachael An	2	24-Jun-13	#NULL!	24	1		2	
Rosemond	1	29-Dec-15	#NULL!	12	2	I had spont	#NULL!	
Daniel Ach	1	17-Dec-15	#NULL!	13	2	My husban	#NULL!	
Celicia Gwa	1	18-Oct-13	#NULL!	24	2	Nothing	#NULL!	
Denis	1	30-Dec-15	#NULL!	24	2	I could not	#NULL!	
Kinsgstey	1	6-Aug-13	#NULL!	24	1		2	
Abraham A	1	18-Oct-15	#NULL!	13	1		2	
Slyvia Aliya	2	9-Dec-11	#NULL!	24	1		2	
Jennifer	2	5-Dec-15	#NULL!	10	1		2	
Honey Nya	1	15-Jun-14	#NULL!	24	1		2	
	2	12-Nov-15	#NULL!	11	1		2	
Christain Ti	1	22-Jan-10	#NULL!	24	1		2	
Ajegewe Av	1	25-Aug-10	#NULL!	16	1		2	
Bernice Abı	2	18-Nov-15	#NULL!	14	2	I did not se	#NULL!	
Nicholas W	2	23-Dec-13	#NULL!	24	1		2	
Clara Kabor	2	10-Sep-13	#NULL!	24	1		2	
Listowel To	1	15-Apr-16	#NULL!	7	1		2	
Juliana Lat	2	24-Oct-08	#NULL!	24	1		2	
Ane Anewe	2	27-Sep-14	#NULL!	18	1		1	Calender m
Ezekiel Kwa	1	17-Oct-14	#NULL!	26	1		2	
	2	1-Nov-15	#NULL!	14	2	Nothing	#NULL!	
Afogajong A	1	9-Apr-15	#NULL!	18	1		2	
Gladys Bab	2	4-Feb-16	#NULL!	27	1		2	
Abena	2	10-Aug-12	#NULL!	27	1		2	
Jaydem	1	16-Nov-14	#NULL!	12	2	Husband n	#NULL!	
Esther	2	30-Jan-14	#NULL!	24	2	Nothing	1	Calender m
	1	11-Dec-15	#NULL!	14	2		#NULL!	
	1	12-Nov-15	#NULL!	8	1		2	
dariana	2	11-Nov-14	#NULL!	18	2	nothing	#NULL!	
emmanuel	2	15-Oct-12	#NULL!	24	1		2	
cathrine kw	2	30-Jan-16	#NULL!	12	1		2	
eugenia yiz	2	30-Apr-13	#NULL!	25	2		#NULL!	
eliana	2	24-Feb-16	#NULL!	25	2		#NULL!	
daniella	2	21-Nov-15	#NULL!	13	1		2	
philip awur	1	11-Dec-15	#NULL!	18	2	nothing	#NULL!	
jacob naba	1	18-Aug-12	#NULL!	24	2		#NULL!	
awedana ta	2	20-Aug-12	#NULL!	24	2	nothing	#NULL!	
philip siwe	1	30-Jan-16	#NULL!	9	1		2	
michael kur	1	24-Feb-13	#NULL!	24	1		2	
weriyirirwo	2	11-Aug-12	#NULL!	16	1		2	
babera	2	23-Apr-14	#NULL!	12	1		2	
sivea	2	25-Nov-15	#NULL!	15	1		1	
Silvian	2	12-Apr-16	#NULL!	12	1		2	

davina anvl	2	########	#NULL!	24	1		2	
nathaniel	1	28-Mar-16	#NULL!	24	1		2	
deborah w	2	18-Sep-14	#NULL!	24	2		#NULL!	
david akun	1	########	#NULL!	18	1		2	
gladys kaba	2	25-Nov-15	#NULL!	12	1		2	
bakiweyen	1	22-Apr-12	#NULL!	24	1		2	
felix kaba	1	10-Oct-14	#NULL!	24	2	i do not wa	#NULL!	
mumuni ka	1	11-Nov-15	#NULL!	10	2	i do not wa	#NULL!	
brian achar	1	28-Aug-13	#NULL!	24	1		2	
adamoah n	2	17-Dec-15	#NULL!	24	1		2	
kudamo jar	2	10-Oct-15	#NULL!	12	2	i do not wa	#NULL!	
webasiah b	1	8-Jul-13	#NULL!	24	1		2	
florence w	2	9-Jan-16	#NULL!	15	2		#NULL!	
issabella pa	2	11-Mar-16	#NULL!	9	1		2	
wenawote	1	3-Feb-16	#NULL!	24	1		2	
kennedy sc	1	22-Jul-10	#NULL!	27	2		#NULL!	
jubene wep	2	24-Sep-11	#NULL!	26	1		2	
erica asoliv	2	18-Apr-16	#NULL!	9	1		2	
cliford abu	1	13-Feb-11	#NULL!	24	2		#NULL!	
clinton aba	1	15-Jul-13	#NULL!	24	2		#NULL!	
slyvia balar	2	14-Dec-15	#NULL!	11	1		2	
Beringa Kar	2	10-Oct-10	#NULL!	18	1		2	
Emma Wep	1	7-Oct-14	#NULL!	24	1		2	
Wisdom Ja	1	17-Dec-15	#NULL!	24	1		2	
Issah Issftu	1	24-Feb-16	#NULL!	12	1		2	
Shafoua La	2	14-Jun-13	#NULL!	24	1		1	I uses the c
Vicentia Ac	2	15-Nov-13	#NULL!	22	1		2	
Baba Salifu	1	10-Feb-16	#NULL!	8	1		2	
Modesta	2	6-May-13	#NULL!	19	1	Husband tr	#NULL!	
Blessing An	1	1-May-16	#NULL!	7	1		2	
	1	10-Oct-15	#NULL!	8	1		#NULL!	
	2	26-Feb-16	#NULL!	9	2		#NULL!	
Awelana	2	9-Apr-14	#NULL!	24	1		2	
David	1	28-Apr-13	1	17	1		2	
Fatima Osn	2	21-Feb-13	#NULL!	24	2		#NULL!	
Orsborn	1	14-Oct-15	#NULL!	12	1		2	
	2	19-Mar-16	#NULL!	11	2		#NULL!	
Isahk Suraj	1	25-Nov-15	#NULL!	12	2	Husband re	#NULL!	
Belvelnad	1	3-Dec-15	#NULL!	9	1		2	
	2	25-Feb-16	#NULL!	10	2		#NULL!	
Abdulirn	1	16-Nov-11	#NULL!	28	1		2	
Shadrack	1	2-Nov-12	#NULL!	8	1		2	
Douglas	1	23-Mar-09	#NULL!	21	1		1	Calender m
Stanley	1	28-Apr-12	#NULL!	24	1		2	
Stephanie	2	18-Dec-15	#NULL!	10	2	No money	#NULL!	
Amadu	1	23-Dec-15	#NULL!	14	2	Wants preg	#NULL!	
	2	18-Feb-16	#NULL!	11	1		#NULL!	

Newton	1	3-Nov-12	#NULL!	24	1		2	
Blessing	2	7-Nov-14	#NULL!	18	1		2	
	1	17-Dec-15	#NULL!	14	1		2	
Fauziya	2	3-Oct-11	#NULL!	18	1		2	
	2	30-Nov-15	#NULL!	13	2		#NULL!	
Noreen	2	30-Sep-09	#NULL!	2	1		2	
Ezaethel Ba	1	10-Feb-16	#NULL!	11	2	Has no rea:	#NULL!	
Weja Apuri	1	16-Oct-13	#NULL!	27	1		2	
julius apan	1	24-Mar-16	#NULL!	36	1		2	
babalewe a	1	11-Oct-13	#NULL!	24	1		2	
safia	2	6-Mar-16	#NULL!	8	1		2	
christina w	2	25-Nov-13	#NULL!	30	1		2	
songojori	1	9-Dec-15	#NULL!	16	1		2	0
agebuno ol	2	28-Dec-14	#NULL!	20	2		#NULL!	
justice ado	1	30-Nov-15	#NULL!	13	2		#NULL!	
christabel a	2	20-Oct-14	#NULL!	30	2		#NULL!	
elisa akulga	2	30-Nov-15	#NULL!	12	1		2	
danwonno	1	19-Dec-15	#NULL!	14	2		#NULL!	
christabel	2	6-Dec-15	#NULL!	9	2		#NULL!	
alhassan fa	1	9-Dec-14	#NULL!	18	2		#NULL!	
johnson	1	30-Dec-15	#NULL!	12	2		#NULL!	
leonard ala	1	21-Nov-13	#NULL!	22	1		2	
titus alaaru	1	30-Jan-16	#NULL!	12	1		2	
martina ab	2	27-Dec-16	#NULL!	24	2		#NULL!	
theresa atu	2	5-Jun-13	#NULL!	25	1		2	
lilian atodic	2	5-Dec-15	#NULL!	16	1		2	
daniel nyaa	1	30-Mar-16	#NULL!	10	2		#NULL!	
pius tijaani	1	9-Nov-15	#NULL!	12	2		#NULL!	
zilam adon	2	4-Jul-13	#NULL!	18	1		2	
aiden akum	1	14-Jul-12	#NULL!	24	1		2	
moses ador	1	30-Dec-16	#NULL!	36	1		2	
nancy azur	2	8-Dec-15	#NULL!	11	1		2	
samu adam	1	20-Dec-13	#NULL!	18	1		2	
elizabeth	2	10-Oct-13	#NULL!	24	1		2	
cynthia nbi	2	11-Jan-16	#NULL!	12	1		2	
wisdom an	1	20-Dec-15	#NULL!	12	2	i don't wan	#NULL!	
ayorebono	1	10-Nov-12	#NULL!	24	2	i haven't de	#NULL!	
	1	14-Apr-16	#NULL!	9	1		2	
aziseri agar	1	26-Jun-11	#NULL!	24	1		2	
mathias aw	1	23-Dec-15	#NULL!	12	2		#NULL!	
	2	9-Mar-10	#NULL!	18	1		2	
abelewine	1	19-Apr-16	#NULL!	20	1		2	0
	2	12-Dec-15	#NULL!	11	2		#NULL!	
adesira abe	1	3-Jun-12	#NULL!	24	1		2	
clinton ben	1	1-Jan-13	#NULL!	24	1		1	abstinance
anabuno ar	1	21-Feb-16	#NULL!	12	1		2	
philipa azik	2	1-Apr-13	#NULL!	28	1		2	

felicia ayab	2	16-Mar-16	#NULL!	27	1		2	
justina agai	2	4-May-14	#NULL!	24	2		#NULL!	
akelignera	2	6-Feb-16	#NULL!	10	1		2	
clemencia a	2	3-Jul-14	#NULL!	24	1		1	abstainanc
calvin ason	1	28-Jul-13	#NULL!	31	1		1	abstainces
awinmata a	2	2-Dec-15	#NULL!	7	2	was not livi	#NULL!	
philip kofi	1	12-Apr-16	#NULL!	11	2		#NULL!	
faiza alhass	2	17-Oct-10	#NULL!	36	1		2	
josiah akuv	1	11-Nov-15	#NULL!	13	2		#NULL!	
nchor lucy	2	30-Jul-14	#NULL!	14	1		2	
jennifer am	2	10-Apr-14	#NULL!	24	2		#NULL!	
shalot akul	2	14-Oct-14	#NULL!	24	2		#NULL!	
joyce asanc	2	18-Feb-16	#NULL!	12	2		#NULL!	
helen asaki	2	5-Jan-15	#NULL!	14	1		2	
shedrack av	1	20-Jul-14	#NULL!	24	1		2	
asakiya	1	2-Sep-13	#NULL!	20	1		2	
avoliya awi	2	4-May-12	#NULL!	30	1		2	
atiborum o	2	16-Feb-16	#NULL!	10	1		2	
adagwine a	2	6-Dec-15	#NULL!	24	1		2	
gladys awir	2	20-Oct-11	#NULL!	29	2		#NULL!	
rahel anafc	2	22-Nov-15	#NULL!	12	1		2	
gideon asu:	1	19-Jun-12	#NULL!	24	2		#NULL!	
adugbire nı	2	2-Nov-13	#NULL!	36	1		2	
gabriel asu	1	11-Nov-15	#NULL!	11	2		#NULL!	
joseph bab	1	11-Dec-14	#NULL!	25	1		2	
atibila anyi	1	14-Feb-16	#NULL!	24	1		2	
akagji kami	1	3-Dec-15	#NULL!	10	1		2	
daniel akug	1	28-Aug-12	#NULL!	24	1		2	
	2	11-Aug-10	#NULL!	24	1		2	
christabel a	2	22-Jan-16	#NULL!	12	2	nothing	#NULL!	
ponganga	2	10-Nov-15	#NULL!	9	2	nothing	#NULL!	
anabil	2	3-Jun-11	#NULL!	18	1		2	
cynthia apc	2	1-Sep-10	#NULL!	24	1		2	
akanbuke a	2	10-Nov-15	#NULL!	15	1		2	
atowine ba	1	10-Nov-10	#NULL!	24	1		2	
macillina aı	2	########	#NULL!	20	1		2	
deliwuni av	1	5-Nov-15	#NULL!	25	2	not living w	#NULL!	
augustine a	1	22-Apr-12	#NULL!	24	1		2	
gideon ayir	1	19-Dec-15	#NULL!	11	1		2	
ajusiba apa	2	11-Mar-14	#NULL!	27	1		1	abstaince
ibrahim ma	1	21-Jan-16	#NULL!	24	2	nothing	#NULL!	
felix atinga	1	3-Apr-16	#NULL!	8	1	0	2	0
ajesiba nya	1	26-Mar-10	#NULL!	24	1		2	
ayidana bal	1	30-Nov-16	#NULL!	24	1		2	
adoliwine a	1	30-Sep-11	#NULL!	24	1		2	
Modesta	2	22-Mar-16	#NULL!	10	2		#NULL!	
richmond r	1	15-Nov-09	#NULL!	30	2		#NULL!	

awinsugiya	1	10-Dec-10	#NULL!	26	1		2
awine abiga	2	18-Dec-15	#NULL!	12	1		2
mma abena	2	3-Feb-16	#NULL!	11	2		#NULL!
	2	10-Jan-14	#NULL!	15	1		2
elisha aber	1	25-Jul-09	#NULL!	36	1		2
awinboya a	1	7-Dec-16	#NULL!	36	1		2
akafabili ak	2	24-Feb-10	#NULL!	25	1		2
akanbeliba	2	31-Dec-15	#NULL!	14	1		2
anyidina ak	1	6-Sep-11	#NULL!	24	1		2
anyidina ak	1	6-Sep-11	#NULL!	12	1		2
amosuno a	2	18-Nov-11	#NULL!	48	2		#NULL!
awinimong	2	9-Nov-16	#NULL!	28	1		2
modesta al	2	26-Feb-16	#NULL!	13	2		#NULL!
onespjilous	1	23-Apr-11	#NULL!	24	1		2
ataree devi	1	1-Oct-15	#NULL!	13	1		2
akafari ama	1	26-Sep-12	#NULL!	24	2	fears the n	#NULL!
atasire	1	4-Mar-16	#NULL!	11	1		2
agabisa kw	1	3-Sep-14	#NULL!	27	1		2
Atoyire Ago	1	18-Jan-13	#NULL!	24	2	i do not wa	#NULL!
Awinebisa A	1	10-Mar-16	#NULL!	12	2	Nothing	#NULL!
Achelwine	2	12-Dec-15	#NULL!	13	1		2
Adijiboba A	2	17-Mar-11	#NULL!	24	2	I do not wa	#NULL!
Akinbe Abo	1	23-Jun-10	#NULL!	20	1		2
Atamiya Av	2	12-Feb-16	#NULL!	18	1		2
Aporeyala I	1	9-Aug-12	#NULL!	24	2	I do not wa	#NULL!
Williams Je	1	15-Jan-16	#NULL!	10	2	I do not hav	#NULL!
Gloria Ncho	2	19-Apr-14	#NULL!	24	2		#NULL!
Prince Agos	1	27-Mar-16	#NULL!	11	1		2
Jane Atiro	2	19-Nov-15	#NULL!	16	1		2
Nancy Wez	2	2-Apr-13	#NULL!	22	2		2
Alban Wed	1	2-Mar-14	#NULL!	23	1		2
Nathasia	2	11-Jan-15	#NULL!	13	1		2
logodabam	1	16-Oct-12	#NULL!	18	1		2
malvin aba	1	15-Mar-16	#NULL!	18	1		2
shadrack	1	8-Nov-15	#NULL!	14	2		#NULL!
elijahil	1	12-Dec-13	#NULL!	24	2		#NULL!
madiba asi	1	17-Apr-14	#NULL!	18	2		#NULL!
issah sulem	1	1-Dec-16	#NULL!	12	1		2
dorcas	2	20-Dec-11	#NULL!	24	1	2	2
Atiah roger	1	10-Feb-16	#NULL!	7	1		2
emmanuell	2	27-Sep-13	#NULL!	16	2		#NULL!
micheal	1	22-Nov-15	#NULL!	19	1		2
nashed har	2	12-Jan-13	#NULL!	10	1		2
Sadick	1	15-Feb-16	#NULL!	10	1		#NULL!
moses abal	1	10-Dec-15	#NULL!	13	1		2
masilatu	2	24-Jan-16	#NULL!	11	1	2	2
amelana nu	2	3-Sep-12	#NULL!	24	1		2

hannah kur	2	14-Jan-16	#NULL!	13	2	#NULL!	
valiham	2	23-Nov-14	#NULL!	12	1	2	
jason chris	1	30-Jan-16	#NULL!	11	1	1	study mens
elikem	1	8-Jun-13	#NULL!	21	1	2	
Justice	1	9-Nov-15	#NULL!	14	1	2	
bervelim	1	4-Jul-13	#NULL!	24	1	2	
blessing	2	12-Apr-16	#NULL!	12	1	2	
blessing	2	12-Apr-13	#NULL!	24	1	2	
ocash	1	10-Jan-16	#NULL!	9	1	2	
leonard	1	2-Aug-12	#NULL!	18	1	2	
madunna	2	12-Aug-12	#NULL!	24	1	2	
lordina	2	27-Dec-15	#NULL!	13	1	2	
Alex	1	13-Nov-15	#NULL!	11	1	2	
Anuboom	2	30-Aug-13	#NULL!	27	1	2	

E5bDepoPr	E5cImplanc	E5eMicrog	E4aNorigyr	E5dJadelle	E6Whydidy	E7Wheredi	E7aothersc	F1Wasyour
1	#NULL!	#NULL!	#NULL!	#NULL!	it can be st	1		1
1	#NULL!	#NULL!	#NULL!	#NULL!	suitable for	1		2
1	#NULL!	#NULL!	#NULL!	#NULL!	it is good a	1		1
#NULL!	#NULL!	#NULL!	#NULL!	#NULL!		#NULL!		#NULL!
1	#NULL!	#NULL!	#NULL!	#NULL!	because yo	1		2
#NULL!	1	#NULL!	#NULL!	#NULL!	that was th	1		1
#NULL!	#NULL!	#NULL!	#NULL!	#NULL!		#NULL!		#NULL!
#NULL!	#NULL!	#NULL!	#NULL!	#NULL!		#NULL!		#NULL!
1	#NULL!	#NULL!	#NULL!	#NULL!	i chose bas	1		1
1	#NULL!	#NULL!	#NULL!	#NULL!	one can sto	1		1
#NULL!	1	#NULL!	#NULL!	#NULL!	i wanted to	1		1
#NULL!	#NULL!	#NULL!	#NULL!	#NULL!		#NULL!		#NULL!
#NULL!	#NULL!	#NULL!	#NULL!	#NULL!		#NULL!		#NULL!
#NULL!	#NULL!	#NULL!	#NULL!	#NULL!		#NULL!		#NULL!
1	#NULL!	#NULL!	#NULL!	#NULL!		1		1
#NULL!	1	#NULL!	#NULL!	#NULL!		1	1	1
#NULL!	#NULL!	#NULL!	#NULL!	#NULL!		#NULL!		#NULL!
1	#NULL!	#NULL!	#NULL!	#NULL!	good for m	1		1
#NULL!	#NULL!	#NULL!	#NULL!	#NULL!		#NULL!		#NULL!
#NULL!	1	#NULL!	#NULL!	#NULL!	because it :	1		1
1	#NULL!	#NULL!	#NULL!	1	they are go	1		1
1	#NULL!	#NULL!	#NULL!	#NULL!	because it's	1		1
#NULL!	#NULL!	#NULL!	#NULL!	#NULL!		#NULL!		#NULL!
#NULL!	#NULL!	1	#NULL!	1		1		1
1	#NULL!	#NULL!	#NULL!	#NULL!	i was comfo	1		2
#NULL!	#NULL!	#NULL!	#NULL!	#NULL!		#NULL!		#NULL!
#NULL!	#NULL!	#NULL!	#NULL!	#NULL!		#NULL!		#NULL!
1	#NULL!	#NULL!	#NULL!	#NULL!	it is good fo	1		1
1	#NULL!	#NULL!	#NULL!	#NULL!	that suits m	1		1
1	#NULL!	#NULL!	#NULL!	#NULL!	it was good	1		1
#NULL!	#NULL!	#NULL!	#NULL!	#NULL!		#NULL!		#NULL!
1	#NULL!	#NULL!	#NULL!	#NULL!		1		1
1	#NULL!	#NULL!	#NULL!	#NULL!	that is wha	1		1
#NULL!	#NULL!	#NULL!	#NULL!	#NULL!		#NULL!		#NULL!
#NULL!	#NULL!	#NULL!	#NULL!	#NULL!		#NULL!		#NULL!
1	#NULL!	#NULL!	#NULL!	#NULL!		#NULL!		1
1	#NULL!	#NULL!	#NULL!	#NULL!	trial	1		1
1	#NULL!	#NULL!	#NULL!	#NULL!	good for m	1		1
#NULL!	#NULL!	#NULL!	#NULL!	#NULL!		#NULL!		#NULL!
1	#NULL!	#NULL!	#NULL!	#NULL!	it is good fo	1		1
1	1	#NULL!	#NULL!	#NULL!	tried depo	1		1
1	#NULL!	#NULL!	#NULL!	#NULL!	trial, but ha	1		2
1	#NULL!	#NULL!	#NULL!	#NULL!	chose to tr	1		2
#NULL!	#NULL!	#NULL!	#NULL!	1		1		1
1	1	#NULL!	#NULL!	#NULL!	beacause o	1		1
#NULL!	#NULL!	#NULL!	#NULL!	#NULL!		#NULL!		#NULL!

1	#NULL!	#NULL!	#NULL!	#NULL!	Because of	1		1
#NULL!	1	#NULL!	#NULL!	#NULL!		1		1
#NULL!	#NULL!	#NULL!	#NULL!	#NULL!		#NULL!		#NULL!
#NULL!	#NULL!	#NULL!	#NULL!	#NULL!		#NULL!		#NULL!
#NULL!	#NULL!	#NULL!	#NULL!	#NULL!		#NULL!		#NULL!
#NULL!	#NULL!	#NULL!	#NULL!	#NULL!		#NULL!		#NULL!
0	0	0	0	1	ito prevent	1	2	2
#NULL!	#NULL!	#NULL!	#NULL!	#NULL!		#NULL!		#NULL!
#NULL!	#NULL!	#NULL!	#NULL!	#NULL!		#NULL!		#NULL!
1	0	0	0	0	resting for	1	1	1
#NULL!	#NULL!	#NULL!	#NULL!	#NULL!		#NULL!		#NULL!
#NULL!	#NULL!	#NULL!	#NULL!	#NULL!		#NULL!		#NULL!
#NULL!	#NULL!	#NULL!	#NULL!	#NULL!		#NULL!		#NULL!
1	0	0	0	0		1		1
1	0	0	0	0		1		1
#NULL!	#NULL!	#NULL!	#NULL!	#NULL!		#NULL!		#NULL!
1	0	0	0	0		1		1
#NULL!	#NULL!	#NULL!	#NULL!	#NULL!		#NULL!		#NULL!
#NULL!	#NULL!	#NULL!	#NULL!	#NULL!		#NULL!		#NULL!
#NULL!	#NULL!	#NULL!	#NULL!	#NULL!		#NULL!		#NULL!
#NULL!	#NULL!	#NULL!	#NULL!	#NULL!		#NULL!		#NULL!
#NULL!	#NULL!	#NULL!	#NULL!	#NULL!		#NULL!		#NULL!
#NULL!	#NULL!	#NULL!	#NULL!	#NULL!	safe and ch	#NULL!		#NULL!
#NULL!	#NULL!	#NULL!	#NULL!	#NULL!		#NULL!		#NULL!
#NULL!	#NULL!	#NULL!	#NULL!	#NULL!		#NULL!		#NULL!
#NULL!	#NULL!	#NULL!	#NULL!	#NULL!		#NULL!		#NULL!
#NULL!	#NULL!	#NULL!	#NULL!	#NULL!		1	3	#NULL!
#NULL!	#NULL!	#NULL!	#NULL!	#NULL!		#NULL!		#NULL!
#NULL!	#NULL!	#NULL!	#NULL!	#NULL!		#NULL!		#NULL!
#NULL!	#NULL!	#NULL!	#NULL!	#NULL!		#NULL!		#NULL!
0	1	0	0	0	just space r	1		1
#NULL!	#NULL!	#NULL!	#NULL!	#NULL!		#NULL!		#NULL!
1	0	0	0	0	it is goog fc	1		1
1	0	0	0	0	just want it	1		1
0	0	0	0	1	I needed sc	1		2
1	0	0	0	0		1		1
#NULL!	#NULL!	#NULL!	#NULL!	#NULL!		#NULL!		#NULL!
1	0	0	0	0	fofr a trick	1		1
#NULL!	#NULL!	#NULL!	#NULL!	#NULL!		#NULL!		#NULL!
#NULL!	#NULL!	#NULL!	#NULL!	#NULL!		#NULL!		#NULL!
0	0	0	0	1	i like tyhat	1		#NULL!
1	0	0	0	0	.i like it	1		1
1	0	0	0	0		1		1
#NULL!	#NULL!	#NULL!	#NULL!	#NULL!		#NULL!		#NULL!
#NULL!	#NULL!	#NULL!	#NULL!	#NULL!		#NULL!		#NULL!
1	0	0	0	0		1		1
#NULL!	#NULL!	#NULL!	#NULL!	#NULL!		#NULL!		#NULL!

#NULL!	#NULL!	#NULL!	#NULL!	#NULL!		#NULL!	#NULL!
#NULL!	#NULL!	#NULL!	#NULL!	#NULL!		#NULL!	#NULL!
1	0	0	0	0	it is good fo	1	1
#NULL!	#NULL!	#NULL!	#NULL!	#NULL!		#NULL!	#NULL!
#NULL!	#NULL!	#NULL!	#NULL!	#NULL!		#NULL!	#NULL!
#NULL!	#NULL!	#NULL!	#NULL!	#NULL!		#NULL!	#NULL!
#NULL!	#NULL!	#NULL!	#NULL!	#NULL!		#NULL!	#NULL!
#NULL!	#NULL!	#NULL!	#NULL!	#NULL!		#NULL!	#NULL!
#NULL!	#NULL!	#NULL!	#NULL!	#NULL!		#NULL!	#NULL!
0	0	0	1	0	nothing	1	1
1	0	0	0	0	nothing	3	1
1	0	0	0	0	nothing	1	1
#NULL!	#NULL!	#NULL!	#NULL!	#NULL!		#NULL!	#NULL!
#NULL!	#NULL!	#NULL!	#NULL!	#NULL!		#NULL!	#NULL!
#NULL!	#NULL!	#NULL!	#NULL!	#NULL!		#NULL!	#NULL!
1	0	0	0	0	it is conven	1	1
1	0	0	0	0	it is good fo	1	1
#NULL!	#NULL!	#NULL!	#NULL!	#NULL!		#NULL!	#NULL!
#NULL!	#NULL!	#NULL!	#NULL!	#NULL!		#NULL!	#NULL!
#NULL!	#NULL!	#NULL!	#NULL!	#NULL!		#NULL!	#NULL!
#NULL!	#NULL!	#NULL!	#NULL!	#NULL!		#NULL!	#NULL!
#NULL!	#NULL!	#NULL!	#NULL!	#NULL!		#NULL!	#NULL!
#NULL!	#NULL!	#NULL!	#NULL!	#NULL!		#NULL!	#NULL!
#NULL!	#NULL!	#NULL!	#NULL!	#NULL!		#NULL!	#NULL!
#NULL!	#NULL!	#NULL!	#NULL!	#NULL!		#NULL!	#NULL!
1	0	0	0	0		#NULL!	1
#NULL!	#NULL!	#NULL!	#NULL!	#NULL!		#NULL!	#NULL!
#NULL!	#NULL!	#NULL!	#NULL!	#NULL!		#NULL!	#NULL!
0	0	1	0	0	no side effe	1	1
0	0	1	0	0	minimal sic	1	1
1	0	0	0	0	nothing	1	2
1	0	0	0	0	nothing	1	1
1	0	0	0	0	nothing	1	1
#NULL!	#NULL!	#NULL!	#NULL!	#NULL!		#NULL!	#NULL!
1	0	0	0	0		1	1
#NULL!	#NULL!	#NULL!	#NULL!	#NULL!		#NULL!	#NULL!
#NULL!	#NULL!	#NULL!	#NULL!	#NULL!		#NULL!	#NULL!
1	0	0	0	0	tried to see	1	1
#NULL!	#NULL!	#NULL!	#NULL!	#NULL!		#NULL!	#NULL!
1	0	0	0	0		#NULL!	1
1	0	0	0	0	nothing	1	1
1	0	0	0	0		1	1
0	0	1	0	0		3	1
0	0	0	1	0		1	1
1	0	0	0	0		1	1
#NULL!	#NULL!	#NULL!	#NULL!	#NULL!		#NULL!	#NULL!
0	0	0	1	0		1	2

#NULL!	#NULL!	#NULL!	#NULL!	#NULL!		#NULL!		#NULL!
#NULL!	#NULL!	#NULL!	#NULL!	#NULL!		#NULL!		#NULL!
1	0	1	0	0	it is good fc	1		1
0	0	1	0	0		3		1
#NULL!	#NULL!	#NULL!	#NULL!	#NULL!		#NULL!		#NULL!
1	0	0	0	0	affects my	1		1
1	0	0	0	0		1		2
1	0	0	0	0	good to use	1		2
#NULL!	#NULL!	#NULL!	#NULL!	#NULL!		4	work place	1
1	0	0	0	0	early returı	1		1
1	0	0	0	0	good to use	1		1
#NULL!	#NULL!	#NULL!	#NULL!	#NULL!		#NULL!		#NULL!
#NULL!	#NULL!	#NULL!	#NULL!	#NULL!		#NULL!		#NULL!
1	0	0	0	0	for spacing	1		1
#NULL!	#NULL!	#NULL!	#NULL!	#NULL!		#NULL!		#NULL!
#NULL!	#NULL!	#NULL!	#NULL!	#NULL!		#NULL!		#NULL!
#NULL!	#NULL!	#NULL!	#NULL!	#NULL!		#NULL!		#NULL!
1	0	0	0	0	it is good fc	1		1
#NULL!	#NULL!	#NULL!	#NULL!	#NULL!		#NULL!		#NULL!
0	0	0	0	1	want to spa	1		1
1	0	0	0	0	it is good fc	1		1
#NULL!	#NULL!	#NULL!	#NULL!	#NULL!		#NULL!		#NULL!
1	0	0	0	0	to rest	1		1
#NULL!	#NULL!	#NULL!	#NULL!	#NULL!		#NULL!		#NULL!
1	0	0	0	0	easy to sto	1		1
#NULL!	#NULL!	#NULL!	#NULL!	#NULL!		#NULL!		#NULL!
0	0	0	0	1	to rest for s	1		1
#NULL!	#NULL!	#NULL!	#NULL!	#NULL!		#NULL!		#NULL!
1	0	0	0	0	wants child	1		1
1	0	0	0	0	nothing	1		1
1	0	0	0	0	it is nice foı	1		1
#NULL!	#NULL!	#NULL!	#NULL!	#NULL!		#NULL!		#NULL!
0	0	0	0	1	wanted a lc	1		2
#NULL!	#NULL!	#NULL!	#NULL!	#NULL!		#NULL!		#NULL!
#NULL!	#NULL!	#NULL!	#NULL!	#NULL!		#NULL!		#NULL!
1	0	0	0	0	i like it	1		2
#NULL!	#NULL!	#NULL!	#NULL!	#NULL!		#NULL!		#NULL!
#NULL!	#NULL!	#NULL!	#NULL!	#NULL!		#NULL!		#NULL!
#NULL!	#NULL!	#NULL!	#NULL!	#NULL!		#NULL!		#NULL!
#NULL!	#NULL!	#NULL!	#NULL!	#NULL!		#NULL!		#NULL!
1	0	0	0	0		1		1
#NULL!	#NULL!	#NULL!	#NULL!	#NULL!		#NULL!		#NULL!
1	0	0	0	0	dont know	1		1
#NULL!	#NULL!	#NULL!	#NULL!	#NULL!		#NULL!		#NULL!
#NULL!	#NULL!	#NULL!	#NULL!	#NULL!		#NULL!		#NULL!
0	0	0	0	1	for my chilc	1		1
#NULL!	#NULL!	#NULL!	#NULL!	#NULL!		#NULL!		#NULL!

#NULL!	#NULL!	#NULL!	#NULL!	#NULL!		#NULL!	#NULL!
1	0	0	0	0	it is good fo	1	1
#NULL!	#NULL!	#NULL!	#NULL!	#NULL!		#NULL!	#NULL!
1	#NULL!	#NULL!	#NULL!	#NULL!	i am comfo	1	1
1	#NULL!	#NULL!	#NULL!	#NULL!		#NULL!	1
#NULL!	#NULL!	#NULL!	#NULL!	1		1	1
1	#NULL!	1	#NULL!	#NULL!	It is good fo	1	1
#NULL!	#NULL!	#NULL!	#NULL!	#NULL!		#NULL!	#NULL!
1	#NULL!	#NULL!	#NULL!	#NULL!	My husban	1	1
1	#NULL!	#NULL!	#NULL!	#NULL!	It is good fo	1	1
#NULL!	#NULL!	#NULL!	#NULL!	#NULL!		#NULL!	#NULL!
#NULL!	#NULL!	#NULL!	#NULL!	#NULL!		#NULL!	#NULL!
1	#NULL!	#NULL!	#NULL!	#NULL!	Just wante	1	1
#NULL!	#NULL!	#NULL!	#NULL!	#NULL!		#NULL!	#NULL!
1	#NULL!	#NULL!	#NULL!	#NULL!	That is wha	1	1
#NULL!	#NULL!	#NULL!	#NULL!	#NULL!		#NULL!	#NULL!
1	#NULL!	#NULL!	#NULL!	#NULL!	It was good	1	1
1	0	0	0	1	some one r	1	1
#NULL!	#NULL!	#NULL!	#NULL!	#NULL!		#NULL!	#NULL!
1	#NULL!	#NULL!	#NULL!	#NULL!		1	1
1	#NULL!	#NULL!	#NULL!	#NULL!	Nothing	1	1
1	#NULL!	#NULL!	#NULL!	#NULL!	It is good fo	1	1
#NULL!	#NULL!	#NULL!	#NULL!	#NULL!		#NULL!	#NULL!
1	#NULL!	#NULL!	#NULL!	#NULL!	That was w	1	2
#NULL!	#NULL!	#NULL!	#NULL!	#NULL!		#NULL!	1
#NULL!	#NULL!	#NULL!	#NULL!	#NULL!		#NULL!	#NULL!
#NULL!	#NULL!	#NULL!	#NULL!	#NULL!		#NULL!	#NULL!
1	#NULL!	#NULL!	#NULL!	#NULL!		1	1
#NULL!	#NULL!	1	#NULL!	#NULL!	To give spa	3	1
1	#NULL!	#NULL!	#NULL!	#NULL!	To give spa	1	1
1	#NULL!	#NULL!	#NULL!	#NULL!	It was good	1	1
1	#NULL!	#NULL!	#NULL!	#NULL!		1	1
#NULL!	#NULL!	#NULL!	#NULL!	#NULL!	That is the	#NULL!	#NULL!
1	#NULL!	#NULL!	#NULL!	#NULL!		1	1
1	#NULL!	#NULL!	#NULL!	#NULL!		1	1
1	#NULL!	#NULL!	#NULL!	#NULL!		1	1
1	#NULL!	#NULL!	#NULL!	#NULL!		1	1
#NULL!	#NULL!	#NULL!	#NULL!	#NULL!		#NULL!	#NULL!
#NULL!	#NULL!	#NULL!	1	#NULL!	Good for m	1	1
#NULL!	#NULL!	#NULL!	#NULL!	#NULL!		#NULL!	#NULL!
0	1	0	0	0	nothing	1	1
#NULL!	#NULL!	#NULL!	#NULL!	#NULL!		#NULL!	#NULL!
#NULL!	#NULL!	#NULL!	#NULL!	#NULL!		#NULL!	#NULL!
#NULL!	#NULL!	#NULL!	#NULL!	#NULL!		#NULL!	#NULL!
#NULL!	#NULL!	#NULL!	#NULL!	1	To prevent	1	1
1	#NULL!	#NULL!	#NULL!	#NULL!	My blood v	1	1
#NULL!	#NULL!	#NULL!	#NULL!	#NULL!		#NULL!	#NULL!

#NULL!	#NULL!	#NULL!	#NULL!	#NULL!		#NULL!		#NULL!
#NULL!	#NULL!	#NULL!	#NULL!	#NULL!		#NULL!		#NULL!
#NULL!	#NULL!	#NULL!	#NULL!	#NULL!		#NULL!		#NULL!
#NULL!	#NULL!	#NULL!	#NULL!	#NULL!		#NULL!		#NULL!
#NULL!	#NULL!	1	#NULL!	#NULL!	Because of	1		1
#NULL!	1	#NULL!	#NULL!	#NULL!	My body is	1		1
#NULL!	#NULL!	#NULL!	#NULL!	#NULL!		#NULL!		#NULL!
#NULL!	#NULL!	#NULL!	#NULL!	#NULL!		#NULL!		#NULL!
#NULL!	#NULL!	#NULL!	#NULL!	#NULL!		#NULL!		#NULL!
#NULL!	#NULL!	#NULL!	#NULL!	#NULL!		#NULL!		#NULL!
1	#NULL!	#NULL!	#NULL!	#NULL!		1		1
#NULL!	#NULL!	#NULL!	#NULL!	#NULL!		#NULL!		#NULL!
1	#NULL!	#NULL!	#NULL!	#NULL!	Prefers tha	1		1
#NULL!	#NULL!	#NULL!	#NULL!	#NULL!		#NULL!		#NULL!
#NULL!	#NULL!	#NULL!	1	#NULL!	To avoid cc	1		1
#NULL!	#NULL!	#NULL!	#NULL!	#NULL!		#NULL!		#NULL!
#NULL!	#NULL!	#NULL!	#NULL!	#NULL!		#NULL!		#NULL!
#NULL!	#NULL!	#NULL!	#NULL!	#NULL!		#NULL!		#NULL!
#NULL!	#NULL!	#NULL!	#NULL!	#NULL!		#NULL!		#NULL!
#NULL!	#NULL!	#NULL!	#NULL!	#NULL!		#NULL!		#NULL!
1	#NULL!	#NULL!	#NULL!	#NULL!	It is good fc	1		1
#NULL!	#NULL!	#NULL!	#NULL!	#NULL!		#NULL!		#NULL!
#NULL!	#NULL!	#NULL!	#NULL!	#NULL!		#NULL!		#NULL!
#NULL!	#NULL!	#NULL!	#NULL!	#NULL!		#NULL!		#NULL!
1	#NULL!	#NULL!	#NULL!	#NULL!	Was introd	1		1
#NULL!	#NULL!	#NULL!	#NULL!	#NULL!		#NULL!		#NULL!
#NULL!	#NULL!	#NULL!	#NULL!	#NULL!		#NULL!		#NULL!
1	#NULL!	#NULL!	#NULL!	#NULL!	She has use	1		1
1	0	0	0	0	for birth sp	1	0	1
#NULL!	#NULL!	#NULL!	#NULL!	#NULL!		#NULL!		#NULL!
#NULL!	#NULL!	#NULL!	#NULL!	#NULL!		#NULL!		#NULL!
1	0	0	0	0	.i like it	1		1
1	0	0	0	0	good for he	1	0	1
#NULL!	#NULL!	#NULL!	#NULL!	1	For long sp	1		1
#NULL!	#NULL!	#NULL!	#NULL!	#NULL!		#NULL!		#NULL!
#NULL!	#NULL!	#NULL!	#NULL!	#NULL!		#NULL!		#NULL!
#NULL!	#NULL!	1	#NULL!	#NULL!	I am comfo	1		1
1	#NULL!	#NULL!	#NULL!	#NULL!		1		1
#NULL!	#NULL!	#NULL!	#NULL!	#NULL!		#NULL!		#NULL!
#NULL!	#NULL!	#NULL!	#NULL!	#NULL!		#NULL!		#NULL!
#NULL!	#NULL!	#NULL!	#NULL!	#NULL!		#NULL!		#NULL!
#NULL!	#NULL!	#NULL!	#NULL!	#NULL!		#NULL!		#NULL!
#NULL!	#NULL!	#NULL!	#NULL!	#NULL!		#NULL!		#NULL!
1	#NULL!	#NULL!	1	#NULL!	To prevent	1		1
#NULL!	#NULL!	#NULL!	#NULL!	#NULL!		#NULL!		#NULL!
#NULL!	#NULL!	#NULL!	#NULL!	#NULL!		#NULL!		#NULL!
#NULL!	#NULL!	#NULL!	#NULL!	1	To prevent	1		1

#NULL!	#NULL!	#NULL!	#NULL!	#NULL!		#NULL!		#NULL!
#NULL!	#NULL!	#NULL!	#NULL!	#NULL!	I want the r	#NULL!	I am aware	#NULL!
#NULL!	#NULL!	#NULL!	#NULL!	#NULL!		#NULL!		#NULL!
#NULL!	#NULL!	#NULL!	#NULL!	#NULL!		#NULL!		#NULL!
1	#NULL!	#NULL!	#NULL!	#NULL!	That one is	1		1
#NULL!	#NULL!	#NULL!	#NULL!	#NULL!		#NULL!		#NULL!
#NULL!	#NULL!	#NULL!	#NULL!	#NULL!		#NULL!		#NULL!
#NULL!	#NULL!	#NULL!	#NULL!	#NULL!		#NULL!		#NULL!
#NULL!	#NULL!	#NULL!	#NULL!	#NULL!		#NULL!		#NULL!
1	#NULL!	#NULL!	#NULL!	#NULL!	nothing	1		1
#NULL!	#NULL!	1	#NULL!	#NULL!	Friend advi	1		1
#NULL!	1	#NULL!	#NULL!	#NULL!	I wanted m	1		1
1	#NULL!	#NULL!	#NULL!	#NULL!	Health staf	1		1
1	#NULL!	#NULL!	#NULL!	#NULL!	To try and s	1		1
#NULL!	1	#NULL!	#NULL!	#NULL!		1		1
1	#NULL!	#NULL!	1	#NULL!	It can be st	1		1
#NULL!	#NULL!	1	#NULL!	#NULL!	To correct r	3		1
#NULL!	#NULL!	#NULL!	#NULL!	#NULL!		#NULL!		#NULL!
1	#NULL!	#NULL!	#NULL!	#NULL!	It is good fo	1		1
1	#NULL!	#NULL!	#NULL!	1	I was bleed	1		1
1	#NULL!	#NULL!	#NULL!	#NULL!	is safe and	1		1
1	#NULL!	#NULL!	#NULL!	#NULL!	It is good a	1		1
#NULL!	#NULL!	#NULL!	#NULL!	#NULL!	i am not co	4		#NULL!
1	#NULL!	#NULL!	#NULL!	#NULL!	Is good	1		1
#NULL!	#NULL!	#NULL!	#NULL!	#NULL!		#NULL!		#NULL!
1	#NULL!	#NULL!	#NULL!	#NULL!	Suitable for	1		1
1	#NULL!	#NULL!	#NULL!	#NULL!	Because of	1		1
1	#NULL!	#NULL!	#NULL!	#NULL!	Depo was h	1		1
#NULL!	#NULL!	#NULL!	#NULL!	#NULL!		#NULL!		#NULL!
#NULL!	#NULL!	#NULL!	#NULL!	#NULL!		#NULL!		#NULL!
#NULL!	#NULL!	#NULL!	#NULL!	#NULL!		#NULL!		#NULL!
1	0	0	0	0	to prrevent	1		1
0	0	0	0	0		#NULL!		#NULL!
1	0	0	0	0		1	0	1
0	1	0	0	0	wanted a lo	1	0	1
0	0	0	0	0		#NULL!		#NULL!
#NULL!	#NULL!	#NULL!	#NULL!	#NULL!		#NULL!		#NULL!
1	0	0	0	0	nothing	1	0	1
#NULL!	#NULL!	#NULL!	#NULL!	#NULL!		#NULL!		#NULL!
#NULL!	#NULL!	#NULL!	#NULL!	#NULL!		#NULL!		#NULL!
#NULL!	#NULL!	#NULL!	#NULL!	#NULL!		#NULL!		#NULL!
1	0	0	0	0	is good for	1	0	1
0	0	0	0	1	i did not wa	1	0	1
1	0	1	0	0	it is helpful	1	0	1
1	0	0	0	0	method wa	1	0	1
1	0	0	0	1	some one r	1		1
1	0	0	0	0	method is g	1	0	1

0	1	0	0	0		1	0	1
1	0	0	0	0	method wa	1	0	1
#NULL!	#NULL!	#NULL!	#NULL!	#NULL!		#NULL!		#NULL!
1	0	0	0	0	she likes it	1	0	1
1	0	0	0	0	suitable for	1		1
1	0	0	0	0	i like it	1		1
#NULL!	#NULL!	#NULL!	#NULL!	#NULL!		#NULL!		#NULL!
#NULL!	#NULL!	#NULL!	#NULL!	#NULL!		#NULL!		#NULL!
1	0	0	0	0	i like it	1	0	1
0	0	0	0	1	that was av	1	0	1
#NULL!	#NULL!	#NULL!	#NULL!	#NULL!		#NULL!		#NULL!
1	0	0	0	0		1	0	1
#NULL!	#NULL!	#NULL!	#NULL!	#NULL!		#NULL!		#NULL!
0	0	0	0	1		1	0	1
1	0	0	0	0		1	0	1
#NULL!	#NULL!	#NULL!	#NULL!	#NULL!		#NULL!		#NULL!
0	0	0	0	1	long space	1	0	1
1	0	0	0	0		1	0	1
#NULL!	#NULL!	#NULL!	#NULL!	#NULL!		#NULL!		#NULL!
#NULL!	#NULL!	#NULL!	#NULL!	#NULL!		#NULL!		#NULL!
1	0	0	0	0		1		1
1	#NULL!	#NULL!	#NULL!	#NULL!	That one is	1		1
1	#NULL!	#NULL!	#NULL!	#NULL!	It is good fo	1		1
1	#NULL!	#NULL!	#NULL!	#NULL!	I am comfo	1		1
1	#NULL!	#NULL!	#NULL!	#NULL!		1		1
#NULL!	#NULL!	#NULL!	#NULL!	#NULL!	I like the na	4	i already kr	#NULL!
#NULL!	#NULL!	#NULL!	#NULL!	1		1		1
1	#NULL!	#NULL!	#NULL!	#NULL!		1		1
#NULL!	#NULL!	#NULL!	#NULL!	#NULL!		1		#NULL!
1	#NULL!	#NULL!	#NULL!	#NULL!	Tha others	1		1
#NULL!	#NULL!	#NULL!	#NULL!	#NULL!		#NULL!		#NULL!
#NULL!	#NULL!	#NULL!	#NULL!	#NULL!		#NULL!		#NULL!
1	#NULL!	#NULL!	#NULL!	#NULL!	To prevent	1		1
1	#NULL!	#NULL!	#NULL!	1		1		1
#NULL!	#NULL!	#NULL!	#NULL!	#NULL!		#NULL!		#NULL!
1	#NULL!	#NULL!	#NULL!	#NULL!		1		1
#NULL!	#NULL!	#NULL!	#NULL!	#NULL!		#NULL!		#NULL!
#NULL!	#NULL!	#NULL!	#NULL!	#NULL!		#NULL!		#NULL!
#NULL!	#NULL!	1	#NULL!	#NULL!		1		1
#NULL!	#NULL!	#NULL!	#NULL!	#NULL!		#NULL!		#NULL!
1	#NULL!	#NULL!	#NULL!	#NULL!	To enable s	1		1
1	#NULL!	#NULL!	#NULL!	#NULL!	It was avail	1		1
#NULL!	#NULL!	#NULL!	#NULL!	#NULL!		#NULL!		#NULL!
1	#NULL!	#NULL!	#NULL!	#NULL!	It was gooc	1		1
#NULL!	#NULL!	#NULL!	#NULL!	#NULL!		#NULL!		#NULL!
#NULL!	#NULL!	#NULL!	#NULL!	#NULL!		#NULL!		#NULL!
#NULL!	#NULL!	#NULL!	#NULL!	#NULL!		#NULL!		#NULL!

1	#NULL!	#NULL!	#NULL!	1		1		1
1	#NULL!	#NULL!	#NULL!	#NULL!		1		1
1	#NULL!	#NULL!	#NULL!	#NULL!		1		1
1	#NULL!	#NULL!	#NULL!	#NULL!		1		1
#NULL!	#NULL!	#NULL!	#NULL!	#NULL!		#NULL!		#NULL!
1	#NULL!	#NULL!	#NULL!	#NULL!	Is part of tł	1		1
#NULL!	#NULL!	#NULL!	#NULL!	#NULL!		#NULL!		#NULL!
1	#NULL!	#NULL!	#NULL!	#NULL!	Depo gives	1		1
1	0	0	0	0	did not wai	1	0	1
1	0	0	0	0	good for he	1	0	1
1	0	0	0	0	is good for	1	0	1
1	0	0	0	0	depo is goc	1	0	1
1	0	0	0	0	just to try	1	0	1
#NULL!	#NULL!	#NULL!	#NULL!	#NULL!		#NULL!		#NULL!
#NULL!	#NULL!	#NULL!	#NULL!	#NULL!		#NULL!		#NULL!
#NULL!	#NULL!	#NULL!	#NULL!	#NULL!		#NULL!		#NULL!
0	0	0	0	1	to be able t	1	0	1
#NULL!	#NULL!	#NULL!	#NULL!	#NULL!		#NULL!		#NULL!
#NULL!	#NULL!	#NULL!	#NULL!	#NULL!		#NULL!		#NULL!
#NULL!	#NULL!	#NULL!	#NULL!	#NULL!		#NULL!		#NULL!
#NULL!	#NULL!	#NULL!	#NULL!	#NULL!		#NULL!		#NULL!
1	0	0	0	0	IS A METHC	1	0	1
1	0	0	0	0	i justb like i	1	0	1
#NULL!	#NULL!	#NULL!	#NULL!	#NULL!		#NULL!		#NULL!
0	0	1	0	0	to prevent	1	0	1
0	0	0	0	1	it is good fc	1		1
#NULL!	#NULL!	#NULL!	#NULL!	#NULL!		#NULL!		#NULL!
#NULL!	#NULL!	#NULL!	#NULL!	#NULL!		#NULL!		#NULL!
0	0	0	0	1	to be able t	2	0	1
1	0	0	1	1	is good for	1	0	1
0	0	0	0	1		1	0	1
0	0	0	0	1	it was the c	1	0	1
0	0	0	0	1	TO PREVEN	1	0	1
0	0	0	0	1	good for m	1	0	1
1	0	0	0	0	nothing	1		2
#NULL!	#NULL!	#NULL!	#NULL!	#NULL!		#NULL!		#NULL!
#NULL!	#NULL!	#NULL!	#NULL!	#NULL!		#NULL!		#NULL!
1	0	0	0	0		#NULL!		1
1	0	0	0	0	for birth sp	1	0	1
#NULL!	#NULL!	#NULL!	#NULL!	#NULL!		#NULL!		#NULL!
1	0	0	0	0	for spacing	1	0	1
0	0	0	0	1	for long spa	1		1
#NULL!	#NULL!	#NULL!	#NULL!	#NULL!		#NULL!		#NULL!
1	0	0	0	0	less side ef	1	0	1
#NULL!	#NULL!	#NULL!	#NULL!	#NULL!	no side effe	#NULL!		1
1	0	0	0	0	no side effe	1	0	1
0	1	0	0	0	wanted to :	1		1

0	1	0	0	0	want my fi	1	0	1
#NULL!	#NULL!	#NULL!	#NULL!	#NULL!		#NULL!		#NULL!
1	0	0	0	0	less expens	1		1
#NULL!	#NULL!	#NULL!	#NULL!	#NULL!	no side effe	4	natural	1
#NULL!	#NULL!	#NULL!	#NULL!	#NULL!	to avoid pr	4	natural	1
#NULL!	#NULL!	#NULL!	#NULL!	#NULL!		#NULL!		#NULL!
#NULL!	#NULL!	#NULL!	#NULL!	#NULL!		#NULL!		#NULL!
1	0	0	0	0		1		1
#NULL!	#NULL!	#NULL!	#NULL!	#NULL!		#NULL!		#NULL!
0	1	0	0	0	nothing	1		1
#NULL!	#NULL!	#NULL!	#NULL!	#NULL!		#NULL!		#NULL!
#NULL!	#NULL!	#NULL!	#NULL!	#NULL!		#NULL!		#NULL!
#NULL!	#NULL!	#NULL!	#NULL!	#NULL!		#NULL!		#NULL!
1	0	0	0	0		#NULL!		1
1	0	0	0	0		1		1
0	1	0	0	0		1		1
1	0	0	0	0		1		1
1	0	0	0	0	it suits her	1		1
1	0	0	0	0		1		1
#NULL!	#NULL!	#NULL!	#NULL!	#NULL!		#NULL!		#NULL!
1	0	0	0	0	it does not	1	0	1
#NULL!	#NULL!	#NULL!	#NULL!	#NULL!		#NULL!		#NULL!
1	0	0	0	0	less side ef	1		1
#NULL!	#NULL!	#NULL!	#NULL!	#NULL!		#NULL!		#NULL!
0	0	1	0	0	no side effe	1		1
1	0	0	0	0	for child sp	1		1
1	0	0	0	0		1		1
1	0	0	0	1		1		2
1	0	0	0	0	is good for	1		1
#NULL!	#NULL!	#NULL!	#NULL!	#NULL!		#NULL!		#NULL!
#NULL!	#NULL!	#NULL!	#NULL!	#NULL!		#NULL!		#NULL!
0	0	0	0	1	want a long	1		1
1	0	0	0	0	good for m	1		1
1	0	0	0	0	suitable for	1	0	1
0	0	1	0	0		1	0	1
1	0	0	0	0	nothing	1		1
#NULL!	#NULL!	#NULL!	#NULL!	#NULL!		#NULL!		#NULL!
1	0	0	0	0	nothing	1	0	1
0	1	0	0	0	wanted to	1	0	1
#NULL!	#NULL!	#NULL!	#NULL!	#NULL!	no side effe	#NULL!	natural	1
#NULL!	#NULL!	#NULL!	#NULL!	#NULL!		#NULL!		#NULL!
0	0	0	1	0		1	0	1
1	0	0	1	0		1	0	1
1	0	0	0	0	0	#NULL!		1
1	0	0	0	0		1	0	1
#NULL!	#NULL!	#NULL!	#NULL!	#NULL!		#NULL!		#NULL!
#NULL!	#NULL!	#NULL!	#NULL!	#NULL!		#NULL!		#NULL!

1	0	0	1	0		1	0	1
1	0	0	0	0	less expens	1	0	1
#NULL!	#NULL!	#NULL!	#NULL!	#NULL!		#NULL!		#NULL!
1	0	0	0	0	it suits her	1	0	1
1	0	0	0	0	nothing	1	0	1
1	0	0	0	0	is good for	1	0	1
1	0	0	0	0	to space m	1	0	1
1	0	0	0	0	is the best	1	0	1
1	0	0	0	0	is good for	1	0	1
1	0	0	0	0	is good for	1	0	1
#NULL!	#NULL!	#NULL!	#NULL!	#NULL!		#NULL!		#NULL!
0	0	1	0	0	the others	1	0	1
#NULL!	#NULL!	#NULL!	#NULL!	#NULL!		#NULL!		#NULL!
1	0	0	0	0	is good for	1	0	1
0	0	1	0	0	the pills is g	1	0	1
#NULL!	#NULL!	#NULL!	#NULL!	#NULL!		#NULL!		#NULL!
1	0	0	0	0	am comfor	1	0	1
0	0	0	1	0	nothing	1	0	1
#NULL!	#NULL!	#NULL!	#NULL!	#NULL!		#NULL!		#NULL!
#NULL!	#NULL!	#NULL!	#NULL!	#NULL!		#NULL!		#NULL!
#NULL!	#NULL!	#NULL!	#NULL!	1		1		1
#NULL!	#NULL!	#NULL!	#NULL!	#NULL!		#NULL!		#NULL!
#NULL!	#NULL!	#NULL!	#NULL!	1		1		1
#NULL!	#NULL!	#NULL!	#NULL!	1	Suitable for	1		1
#NULL!	#NULL!	#NULL!	#NULL!	#NULL!		#NULL!		#NULL!
#NULL!	#NULL!	#NULL!	#NULL!	#NULL!		#NULL!		#NULL!
#NULL!	#NULL!	#NULL!	#NULL!	#NULL!		#NULL!		#NULL!
1	#NULL!	#NULL!	#NULL!	#NULL!		1		1
1	#NULL!	#NULL!	#NULL!	#NULL!		1		1
#NULL!	1	#NULL!	#NULL!	#NULL!	I will not vi	1		1
#NULL!	#NULL!	#NULL!	#NULL!	1	It is safe fo	1		1
1	#NULL!	#NULL!	#NULL!	#NULL!	Wants to tr	1		1
0	1	0	0	0	to rest a wl	1		1
0	0	0	1	0	it is good fc	1		1
#NULL!	#NULL!	#NULL!	#NULL!	#NULL!		#NULL!		#NULL!
#NULL!	#NULL!	#NULL!	#NULL!	#NULL!		#NULL!		#NULL!
#NULL!	#NULL!	#NULL!	#NULL!	#NULL!		#NULL!		#NULL!
1	0	0	0	0	i just that c	1		1
1	0	0	0	0	it's ggod fo	1		1
1	0	0	0	0	a friend rec	1		1
#NULL!	#NULL!	#NULL!	#NULL!	#NULL!		#NULL!		#NULL!
#NULL!	#NULL!	#NULL!	#NULL!	#NULL!	not comfor	3		1
1	0	0	0	0	kit was goc	1		1
#NULL!	#NULL!	#NULL!	#NULL!	#NULL!		#NULL!		#NULL!
1	0	0	0	0	triasl	1		1
1	0	0	0	0	i needed a	1	1	1
1	0	0	0	0	trial	1		1

#NULL!	#NULL!	#NULL!	#NULL!	#NULL!		#NULL!	#NULL!
1	0	0	0	1	the depo w	1	1
#NULL!	#NULL!	#NULL!	#NULL!	#NULL!	not sure of	#NULL!	1
1	0	0	0	0	trial	#NULL!	1
1	0	0	0	0	wanted sho	1	1
1	0	0	0	0	wantted to	1	1
1	0	0	0	0	it is good fo	1	1
1	0	0	0	0	nothing	1	1
1	0	0	0	0	to space m	1	1
#NULL!	#NULL!	1	#NULL!	#NULL!		3	1
1	0	0	0	0	was conver	1	1
1	0	1	0	0	i still have r	1	1
1	0	1	0	0		1	1
1	#NULL!	#NULL!	#NULL!	#NULL!	My husban	1	1

F2Ifyesdoe	F3IFNOwhy	E4Whodec	Parity0	Gender3	sons	Daughters	E6children	HHealthPro
1		3	4	2	1	3	5	2
#NULL!	if i tell him,	1	2	2	#NULL!	2	5	2
1		1	2	1	1	1	3	1
#NULL!		3	3	1	3	0	4	2
#NULL!	because he	1	3	2	1	2	6	1
1		3	2	1	2	#NULL!	4	2
#NULL!		#NULL!	2	1	1	1	3	2
#NULL!		3	1	1	#NULL!	1	3	1
1		1	2	1	2	#NULL!	3	2
1		3	2	2	1	1	4	1
1		#NULL!	4	1	3	1	4	2
#NULL!		#NULL!	2	2	1	1	3	2
#NULL!		#NULL!	2	1	2	#NULL!	5	1
#NULL!		3	2	1	2	#NULL!	2	2
1		3	2	2	#NULL!	2	4	2
1		#NULL!	2	2	#NULL!	2	4	2
#NULL!		3	2	1	2	#NULL!	5	2
1		#NULL!	3	1	2	1	3	2
#NULL!		3	4	1	4	#NULL!	4	2
1		1	3	2	2	1	4	2
1		1	2	2	#NULL!	2	5	2
1		#NULL!	2	2	#NULL!	2	4	2
#NULL!		1	4	1	3	1	4	2
1		1	2	2	1	1	2	2
#NULL!	I don't stay	#NULL!	3	1	1	1	3	2
#NULL!		#NULL!	4	2	1	3	6	2
#NULL!		1	5	2	1	4	5	2
1		3	2	2	1	1	5	2
1		3	3	2	2	#NULL!	5	2
1		#NULL!	4	2	2	2	5	2
#NULL!		3	2	2	1	1	5	2
1		3	2	1	2	#NULL!	5	2
1		#NULL!	2	1	2	#NULL!	5	2
#NULL!		#NULL!	3	2	2	1	4	2
#NULL!		1	4	2	2	2	4	2
1		1	2	1	1	1	4	2
1		1	2	1	2	#NULL!	4	2
2		#NULL!	2	1	1	1	4	2
#NULL!		1	2	2	1	1	3	2
1		1	5	2	3	1	4	2
1		#NULL!	3	1	2	#NULL!	4	2
#NULL!	not marrie	1	3	1	3	#NULL!	3	2
#NULL!	not marrie	3	3	1	3	#NULL!	3	2
1		3	5	1	4	1	6	2
1		#NULL!	3	2	1	2	5	2
#NULL!		3	4	1	3	1	5	2

1		#NULL!	3	2	1	2	5	2
1		#NULL!	2	1	2	#NULL!	4	2
#NULL!		#NULL!	2	1	2	#NULL!	4	2
#NULL!		#NULL!	1	1	1	1	5	2
#NULL!		1	2	1	1	1	5	2
#NULL!		#NULL!	2	1	1	1	4	2
2	dead	#NULL!	5	1	4	3	7	2
#NULL!		3	1	2	1	1	#NULL!	2
#NULL!		#NULL!	4	1	3	1	4	2
1		#NULL!	6	2	1	1	1	2
#NULL!		#NULL!	2	1	2	0	5	2
#NULL!		3	2	1	1	0	4	2
#NULL!		3	3	1	1	2	4	2
1		#NULL!	2	2	2	0	4	2
1		3	2	1	1	11	4	2
#NULL!		#NULL!	5	2	2	3	5	2
1		#NULL!	2	2	1	1	4	2
#NULL!		#NULL!	4	2	0	3	10	2
#NULL!		#NULL!	3	2	0	3	5	2
#NULL!		#NULL!	4	1	2	2	10	2
#NULL!		#NULL!	6	2	2	4	7	2
#NULL!		1	8	1	3	5	10	2
#NULL!		#NULL!	2	2	0	2	4	2
#NULL!		#NULL!	4	1	2	1	10	2
#NULL!		#NULL!	6	1	2	2	10	2
#NULL!		#NULL!	3	2	1	2	4	2
#NULL!		#NULL!	5	1	3	2	7	2
#NULL!		#NULL!	3	2	0	3	3	2
#NULL!		2	2	2	0	2	3	2
#NULL!		#NULL!	5	2	0	5	5	1
1		2	2	2	0	2	5	2
#NULL!		2	4	1	4	2	6	2
1		1	4	2	2	1	5	2
1		3	3	2	2	1	4	2
2	he will not	#NULL!	4	1	3	1	4	2
1		3	6	1	3	3	6	1
#NULL!		#NULL!	5	2	5	1	6	2
1		#NULL!	3	2	1	2	3	2
#NULL!		#NULL!	6	2	1	5	7	2
#NULL!		3	2	1	2	0	3	2
#NULL!		3	3	1	2	1	5	2
1		#NULL!	2	1	1	1	3	2
1		#NULL!	3	2	1	2	4	2
#NULL!		3	2	2	0	2	2	2
#NULL!		#NULL!	3	2	1	2	4	2
1		#NULL!	2	1	2	#NULL!	2	2
#NULL!		#NULL!	3	2	2	1	10	2

#NULL!		3	5	1	3	2	11	2
#NULL!		#NULL!	2	2	2	2	5	2
1		#NULL!	1	2	1	1	3	2
#NULL!		#NULL!	5	2	1	4	10	2
#NULL!		#NULL!	5	2	1	4	10	2
#NULL!		#NULL!	4	1	2	2	10	2
#NULL!		3	3	1	2	1	5	2
#NULL!		3	2	2	1	1	4	2
#NULL!		1	4	2	2	2	6	2
1		#NULL!	3	2	0	3	7	2
1		#NULL!	2	1	2	0	6	2
1		#NULL!	4	1	3	0	10	2
#NULL!		3	1	1	2	2	6	2
#NULL!		3	6	1	1	5	6	2
#NULL!		#NULL!	2	2	0	2	4	2
1		#NULL!	3	2	1	2	5	2
1		#NULL!	6	1	1	5	6	2
#NULL!		#NULL!	5	2	1	3	6	2
#NULL!		#NULL!	3	1	2	1	6	2
#NULL!		#NULL!	3	1	2	1	6	2
#NULL!		#NULL!	6	1	3	3	6	2
#NULL!		#NULL!	2	1	1	1	4	2
#NULL!		3	3	1	3	0	4	2
#NULL!		#NULL!	3	1	1	2	3	2
#NULL!		#NULL!	3	2	2	1	4	2
1		3	2	2	0	2	5	2
#NULL!		3	2	2	0	2	4	2
#NULL!		1	6	2	2	4	6	2
1		1	2	2	1	1	4	2
1		3	3	2	1	2	5	2
#NULL!	was not ma	#NULL!	3	2	1	2	5	2
1		3	2	1	1	1	5	2
1		#NULL!	4	1	2	1	7	2
#NULL!		#NULL!	6	2	3	3	6	2
1		3	2	2	1	1	5	2
#NULL!		#NULL!	2	1	2	0	4	2
#NULL!		3	2	2	1	0	7	2
1		3	3	2	2	1	4	2
#NULL!		3	3	1	1	2	10	2
1		3	2	1	1	1	4	2
1		3	4	2	3	1	6	2
1		3	7	1	3	2	7	2
1		#NULL!	4	1	3	1	6	2
1		1	4	1	0	3	10	2
1		#NULL!	2	2	1	1	4	2
#NULL!		#NULL!	4	1	2	2	8	2
2	i didnt tell I	1	4	2	2	2	4	2

#NULL!		3	4	2	2	2	4	2
#NULL!		#NULL!	2	2	1	1	3	2
1		3	4	1	2	2	4	2
1		1	2	1	1	1	4	2
#NULL!		1	3	1	2	1	4	2
2	he didnt as	3	2	1	2	0	2	2
#NULL!	border not	2	5	2	2	2	5	2
#NULL!	he wouldnt	3	2	1	2	0	3	2
1		#NULL!	2	1	2	0	4	2
2	he was not	2	2	1	2	0	3	1
1		3	4	2	3	1	4	2
#NULL!		#NULL!	3	2	0	3	10	2
#NULL!		#NULL!	5	2	2	3	5	2
1		3	4	1	4	0	7	2
#NULL!		#NULL!	4	1	3	1	5	2
#NULL!		#NULL!	2	1	1	1	4	2
#NULL!		#NULL!	6	2	2	3	5	2
1		2	5	2	3	1	5	2
#NULL!		#NULL!	2	1	2	0	4	2
1		3	5	2	2	3	6	2
1		3	2	2	1	1	2	2
#NULL!		#NULL!	4	1	2	2	4	2
1		1	6	1	3	3	6	2
#NULL!		#NULL!	2	2	1	1	4	2
1		1	4	2	2	2	5	2
#NULL!		#NULL!	2	2	1	0	3	2
1		1	5	1	1	3	5	2
#NULL!		#NULL!	2	1	2	0	4	2
1		3	2	2	0	2	3	2
1		2	4	1	2	2	6	2
1		3	5	1	4	1	5	2
#NULL!		#NULL!	2	1	1	1	4	2
#NULL!	he wasnt ir	1	6	1	3	3	6	2
#NULL!		#NULL!	2	1	1	1	5	2
#NULL!		#NULL!	2	1	2	0	5	2
#NULL!	he wont ag	1	2	1	2	0	5	2
#NULL!		#NULL!	3	2	1	2	4	2
#NULL!		#NULL!	3	1	2	1	3	2
#NULL!		#NULL!	2	2	0	2	4	2
#NULL!		#NULL!	5	1	4	1	5	2
1		2	6	1	3	3	6	2
#NULL!		#NULL!	2	2	1	1	6	2
1		1	3	2	2	1	5	2
#NULL!		#NULL!	4	1	2	2	5	2
#NULL!		#NULL!	5	1	1	4	7	2
1		3	2	1	2	0	5	2
#NULL!		#NULL!	4	1	3	1	4	2

#NULL!		#NULL!	2	2	0	1	4	2
1		3	3	1	2	1	3	2
#NULL!		#NULL!	2	2	1	1	2	2
1		3	4	1	1	3	4	2
1		3	3	2	2	1	3	2
1		3	2	2	#NULL!	2	4	2
1		1	4	2	2	2	#NULL!	2
#NULL!		#NULL!	2	2	1	1	4	2
1		2	3	2	1	2	6	2
1		3	5	1	3	2	6	2
#NULL!		#NULL!	2	2	1	1	#NULL!	2
#NULL!		#NULL!	2	2	0	2	10	2
1		2	3	1	2	1	10	2
#NULL!		#NULL!	2	2	0	2	8	2
1		3	3	2	0	3	6	2
#NULL!		#NULL!	4	1	3	1	4	2
1		3	5	2	3	2	5	2
1		3	3	2	1	2	4	2
#NULL!		#NULL!	2	2	1	0	5	2
1		3	8	1	6	2	8	2
1		1	3	2	2	1	5	2
1		3	4	2	1	3	4	2
#NULL!		#NULL!	2	2	0	2	2	2
#NULL!	He would r	1	5	1	4	1	7	2
1		3	2	2	1	1	3	2
#NULL!		#NULL!	2	1	1	1	4	2
#NULL!		#NULL!	1	2	0	1	3	2
1		3	3	1	3	0	4	2
1		3	3	1	2	1	3	2
1		3	3	2	2	1	4	2
1		3	2	1	1	1	3	2
1		3	3	2	1	2	3	2
#NULL!		1	6	2	2	4	6	2
1		3	3	2	0	3	4	2
1		3	3	1	2	1	4	2
1		3	3	2	1	2	3	2
1		3	2	1	2	0	2	1
#NULL!		#NULL!	2	2	1	1	4	2
1		3	2	1	1	1	4	2
#NULL!		#NULL!	4	1	2	2	4	2
1		1	2	1	2	0	3	2
#NULL!		#NULL!	2	1	1	1	4	2
#NULL!		#NULL!	2	1	1	1	5	2
#NULL!		#NULL!	2	1	1	1	3	2
1		3	2	1	1	1	6	2
1		3	2	1	1	1	5	2
#NULL!		#NULL!	2	1	2	0	6	2

#NULL!	#NULL!	2	2	0	2	4	2
#NULL!	#NULL!	2	1	2	#NULL!	6	2
#NULL!	#NULL!	3	2	0	3	6	2
#NULL!	#NULL!	4	1	4	#NULL!	4	2
1	1	5	2	1	4	5	2
2	3	2	2	0	2	5	2
#NULL!	#NULL!	2	2	0	2	5	2
#NULL!	#NULL!	2	2	1	1	6	2
#NULL!	#NULL!	6	2	3	3	6	2
#NULL!	#NULL!	3	2	0	3	4	2
1	3	3	2	1	2	#NULL!	2
#NULL!	#NULL!	2	1	1	1	5	2
1	3	2	1	1	1	3	2
#NULL!	#NULL!	2	1	1	1	6	2
1	1	3	2	2	1	6	2
#NULL!	#NULL!	3	1	1	2	6	2
#NULL!	#NULL!	2	2	#NULL!	2	6	2
#NULL!	#NULL!	5	1	2	3	6	2
#NULL!	#NULL!	6	2	4	2	#NULL!	2
#NULL!	#NULL!	4	2	1	3	5	2
1	3	5	2	1	4	5	2
#NULL!	#NULL!	3	2	1	1	9	2
#NULL!	#NULL!	2	2	1	1	#NULL!	2
#NULL!	#NULL!	5	1	#NULL!	5	10	2
1	1	2	1	1	1	#NULL!	2
#NULL!	#NULL!	4	1	2	2	6	2
#NULL!	#NULL!	2	1	2	#NULL!	5	2
1	3	3	2	1	2	4	2
1	3	6	2	4	2	6	2
#NULL!	#NULL!	3	2	1	2	3	2
#NULL!	#NULL!	8	1	3	5	10	2
1	3	2	1	1	1	3	2
1	1	5	2	3	2	6	2
1	3	2	1	1	1	4	2
#NULL!	#NULL!	2	2	0	2	5	2
#NULL!	#NULL!	3	2	2	1	3	2
1	3	3	2	1	2	5	2
1	3	3	1	3	0	#NULL!	2
#NULL!	#NULL!	2	2	1	1	2	2
#NULL!	#NULL!	3	1	2	1	4	2
#NULL!	#NULL!	3	2	0	3	4	2
#NULL!	#NULL!	2	1	2	0	4	2
#NULL!	#NULL!	3	2	1	2	3	2
1	3	3	2	2	1	4	2
#NULL!	#NULL!	2	1	1	1	4	2
#NULL!	#NULL!	3	1	3	0	4	2
1	3	2	1	1	1	3	2

#NULL!		#NULL!	2	1	1	1	4	2
#NULL!		1	3	1	2	1	3	2
#NULL!		#NULL!	3	1	2	1	5	1
#NULL!		#NULL!	2	1	1	1	3	2
2	He did not	3	2	2	1	1	4	2
#NULL!		#NULL!	2	1	1	1	4	2
#NULL!		#NULL!	3	1	3	0	3	2
#NULL!		#NULL!	2	1	1	1	4	2
#NULL!		#NULL!	2	1	1	1	2	2
1		3	2	1	2	0	3	2
1		3	2	1	1	1	4	2
2	I did it outs	1	2	2	0	2	4	2
1		2	2	2	0	2	4	2
1		3	2	2	0	2	3	2
1		3	5	1	0	3	3	2
1		3	4	1	2	2	4	2
1		3	3	1	3	0	3	2
#NULL!		#NULL!	2	2	0	2	2	2
1		1	4	2	2	2	4	2
1		1	2	2	0	2	5	2
1		1	2	1	2	0	4	2
1		1	4	1	2	2	4	2
1		1	2	2	1	1	3	2
1		1	3	1	3	0	5	2
#NULL!		#NULL!	3	1	2	2	4	2
1		1	3	2	1	2	5	2
1		3	2	2	1	1	4	2
1		3	3	2	0	3	4	2
#NULL!		#NULL!	3	1	3	0	3	2
#NULL!		#NULL!	3	2	0	3	4	2
#NULL!		#NULL!	3	2	2	1	4	2
1		3	2	1	1	1	3	2
#NULL!		#NULL!	2	2	0	2	4	2
1		3	3	1	1	2	3	2
1	0	2	3	1	2	1	4	2
#NULL!		#NULL!	4	1	3	1	5	2
#NULL!		#NULL!	3	1	2	1	6	2
1		1	2	2	1	1	4	2
#NULL!		#NULL!	5	2	3	2	5	2
#NULL!		#NULL!	2	1	1	1	3	2
#NULL!		#NULL!	3	2	0	3	5	2
1		3	2	1	2	0	3	2
1		3	4	2	2	2	5	2
1		3	4	1	3	1	5	2
1		3	3	2	0	3	4	2
1		3	3	2	1	2	4	2
1		2	5	2	2	1	5	2

1		3	2	2	1	1	3	2
1		3	3	1	2	1	4	2
#NULL!		#NULL!	4	1	2	3	5	2
1		3	2	1	1	1	5	2
1		1	2	2	0	2	10	2
1		3	2	1	2	0	4	2
#NULL!		#NULL!	2	1	2	0	3	2
#NULL!		#NULL!	2	1	2	0	3	2
1		5	2	1	1	1	4	2
1		3	2	2	1	1	4	2
#NULL!		#NULL!	4	2	2	2	5	2
1		3	3	2	1	2	4	2
#NULL!		#NULL!	2	2	0	2	4	2
1		3	4	2	1	3	4	2
1		3	6	2	3	3	6	2
#NULL!		#NULL!	2	1	2	0	4	2
1		3	5	2	2	3	5	2
1	0	3	3	2	1	2	5	2
#NULL!		#NULL!	3	1	3	0	4	2
#NULL!		#NULL!	4	1	2	1	4	2
1		3	2	2	1	1	4	2
1		3	2	2	1	1	4	2
1		3	2	1	2	0	3	2
1		3	3	2	1	2	3	2
1		3	2	1	2	0	3	2
#NULL!		#NULL!	4	2	3	1	4	2
1		3	4	1	3	1	#NULL!	2
1		3	5	2	3	2	5	2
#NULL!		#NULL!	2	1	1	1	5	2
1		3	2	1	1	1	3	2
#NULL!		#NULL!	2	1	1	1	4	2
#NULL!		#NULL!	1	2	0	1	3	2
2	I did not in	1	5	2	1	4	5	2
1		3	5	2	3	2	5	2
#NULL!		#NULL!	5	1	2	2	#NULL!	2
1		3	3	2	1	2	3	2
#NULL!		#NULL!	1	2	0	1	3	2
#NULL!		#NULL!	2	1	1	1	4	2
1		3	4	1	4	0	4	2
#NULL!		#NULL!	1	1	1	0	3	2
1		1	4	2	3	1	5	1
1		3	2	1	1	1	4	2
#NULL!		#NULL!	2	1	2	0	3	2
1		3	2	1	1	1	3	2
#NULL!		#NULL!	2	2	1	1	2	2
#NULL!		#NULL!	2	1	1	1	2	2
#NULL!		#NULL!	2	1	1	1	3	2

1		3	2	1	1	1	3	2
1		3	2	2	0	2	2	2
1		3	2	1	1	1	3	2
1		3	3	2	0	3	#NULL!	2
#NULL!		#NULL!	1	1	1	0	2	2
1		3	3	2	0	3	5	2
#NULL!		#NULL!	3	1	2	1	4	2
1		1	5	2	3	2	5	2
1		1	4	1	3	1	4	2
1		1	5	2	3	2	6	2
1		1	6	2	3	3	6	2
1		2	4	1	2	2	4	2
1		3	3	1	3	0	5	2
#NULL!		#NULL!	3	1	1	2	5	2
#NULL!		#NULL!	3	2	2	1	3	2
#NULL!		#NULL!	1	2	1	1	3	2
1		3	5	1	3	2	6	2
#NULL!		#NULL!	2	1	2	#NULL!	2	2
#NULL!		#NULL!	4	1	1	3	4	2
#NULL!		#NULL!	2	1	1	1	4	2
#NULL!		#NULL!	3	2	2	1	3	2
1		3	3	2	1	2	4	2
1		1	2	1	1	1	3	2
#NULL!		#NULL!	2	2	#NULL!	2	2	2
1		1	5	2	2	3	5	2
1		3	2	2	#NULL!	2	6	2
#NULL!		#NULL!	3	2	1	2	4	2
#NULL!		#NULL!	2	1	2	#NULL!	2	2
1		1	2	1	1	1	4	2
1		3	5	1	4	1	5	2
1		3	2	1	2	0	2	2
1		1	2	2	1	1	5	2
1		3	2	1	2	#NULL!	3	2
1		3	3	1	2	1	3	2
#NULL!	he would n	1	7	1	4	3	7	2
#NULL!		#NULL!	2	1	1	1	6	2
#NULL!		#NULL!	2	1	2	#NULL!	5	2
1		3	2	2	1	1	#NULL!	2
1		3	6	2	4	2	6	2
#NULL!		#NULL!	2	1	2	#NULL!	9	2
1		3	3	2	0	3	4	2
1		3	4	2	2	2	4	2
#NULL!		#NULL!	2	1	1	1	4	2
1		3	5	1	3	2	10	2
1		3	2	1	1	1	4	2
1		3	6	2	2	4	7	2
1		3	4	1	2	2	4	2

1	1	2	2	1	1	5	2
#NULL!	#NULL!	2	2	0	2	5	2
1	3	4	2	0	4	15	2
1	3	4	2	2	2	6	2
1	3	2	1	1	1	10	2
#NULL!	#NULL!	4	1	1	3	20	2
#NULL!	#NULL!	6	1	4	2	7	2
1	3	6	2	2	4	6	2
#NULL!	#NULL!	2	1	1	1	3	2
1	3	4	1	3	1	4	2
#NULL!	#NULL!	3	2	1	2	6	2
#NULL!	#NULL!	3	2	0	3	10	2
#NULL!	#NULL!	3	1	1	2	5	2
1	3	6	1	2	3	6	2
1	3	3	1	3	0	10	2
1	3	2	1	2	0	4	2
1	2	4	2	1	3	5	2
1	3	4	2	1	3	4	2
1	3	2	2	0	2	4	2
#NULL!	#NULL!	2	2	1	1	4	2
1	3	2	2	1	1	5	2
#NULL!	#NULL!	3	2	0	2	4	2
1	3	4	2	1	3	4	2
#NULL!	#NULL!	2	1	1	1	6	2
1	3	4	1	3	2	5	2
1	3	4	1	3	1	5	2
1	3	2	1	1	1	6	2
#NULL!	he will not	1	3	1	2	1	4	2
1	3	2	2	1	1	4	2
#NULL!	#NULL!	3	1	1	2	5	2
#NULL!	#NULL!	1	2	3	3	6	2
1	3	2	2	1	1	4	2
1	3	4	1	2	2	5	2
1	3	5	2	1	4	6	2
1	3	5	1	2	0	4	2
1	3	3	1	2	1	5	2
#NULL!	#NULL!	7	1	3	4	7	2
1	3	2	1	2	0	4	2
1	3	3	2	2	1	6	2
1	3	4	1	2	2	4	2
#NULL!	#NULL!	6	2	2	4	10	2
1	3	5	2	2	3	5	2
1	3	5	1	2	3	10	2
1	3	2	1	1	1	4	2
1	3	4	1	3	1	6	2
#NULL!	#NULL!	10	2	2	5	10	2
#NULL!	#NULL!	3	1	2	1	5	2

1		3	4	2	1	3	5	2
1		3	4	2	2	2	5	2
#NULL!		#NULL!	4	1	2	2	6	2
1		3	3	2	2	1	4	2
1		3	2	1	1	1	4	2
1		4	3	2	2	1	4	2
1		3	4	1	2	2	4	2
1		3	3	2	0	3	6	2
1		1	3	1	2	1	6	2
1		1	3	1	2	1	6	2
#NULL!		#NULL!	5	1	1	5	5	2
1		3	3	1	1	2	6	2
#NULL!		#NULL!	3	1	1	2	4	2
2		1	3	1	3	0	4	2
1		3	3	1	3	0	4	2
#NULL!		#NULL!	6	1	5	1	20	2
1		3	3	2	2	1	4	2
1		3	5	1	4	1	6	2
#NULL!		#NULL!	4	1	3	1	5	2
#NULL!		#NULL!	5	2	3	1	4	2
1		3	3	1	2	1	5	2
#NULL!		#NULL!	7	2	1	6	7	2
1		3	5	2	2	3	7	2
1		3	2	2	1	2	10	2
#NULL!		#NULL!	6	2	1	5	6	2
#NULL!		#NULL!	2	1	1	1	4	2
#NULL!		#NULL!	9	2	1	8	9	2
1		3	3	1	2	1	3	2
1		3	5	1	3	2	5	2
1		1	3	2	1	2	4	2
1		3	4	1	2	2	4	2
1		1	3	2	1	2	4	2
1		1	2	1	0	4	4	2
1		1	4	2	1	2	5	2
#NULL!		#NULL!	2	1	1	1	4	2
#NULL!		#NULL!	3	2	1	2	6	2
#NULL!		#NULL!	3	2	1	2	4	2
1		3	2	2	1	1	5	2
1		1	5	1	2	3	5	2
2	nothing	1	2	2	2	2	3	2
#NULL!		#NULL!	3	2	1	2	6	2
1		3	2	2	1	1	2	2
1	1	1	3	2	2	1	4	2
#NULL!		#NULL!	2	2	0	1	3	2
1		1	5	1	2	2	4	2
1		1	3	2	0	3	5	2
1		1	6	1	2	2	4	2

#NULL!		#NULL!	2	1	1	1	4	2
1		1	2	2	1	1	4	2
1		1	2	1	1	1	3	2
1		1	2	1	1	1	3	2
1		1	4	2	1	1	3	2
2	he will not	#NULL!	3	1	1	2	4	2
1		1	3	2	2	1	3	2
1		1	3	2	2	1	3	2
1		1	6	2	1	5	6	2
1		2	2	1	2	0	3	2
1		3	3	2	1	2	6	2
1		1	3	2	1	2	4	2
1		1	4	2	2	2	3	2
1		2	3	2	1	2	6	2

H1aNorigy	H1bDepop	H1cImplan	H1dJadelle	H1eMicrog	H2Andwhy	H3Inyouro	Gestation	Mode
#NULL!	#NULL!	#NULL!	#NULL!	#NULL!			1	1
#NULL!	#NULL!	#NULL!	#NULL!	#NULL!			1	1
#NULL!	1	#NULL!	#NULL!	#NULL!	the see it t	it helps the	1	1
#NULL!	#NULL!	#NULL!	#NULL!	#NULL!			1	1
5	1	2	3	4	they are co	to allow ch	1	1
#NULL!	#NULL!	#NULL!	#NULL!	#NULL!			1	1
#NULL!	#NULL!	#NULL!	#NULL!	#NULL!			1	2
#NULL!	1	2	4	3	depo is not	they do to	1	1
#NULL!	#NULL!	#NULL!	#NULL!	#NULL!			1	1
#NULL!	1	#NULL!	#NULL!	#NULL!	it has mino	it helps to	1	1
#NULL!	#NULL!	#NULL!	#NULL!	#NULL!			1	1
#NULL!	#NULL!	#NULL!	#NULL!	#NULL!			1	1
5	1	2	3	4	it si simple	mothers wa	1	1
#NULL!	#NULL!	#NULL!	#NULL!	#NULL!			1	1
#NULL!	#NULL!	#NULL!	#NULL!	#NULL!			1	1
#NULL!	#NULL!	#NULL!	#NULL!	#NULL!			1	1
#NULL!	#NULL!	#NULL!	#NULL!	#NULL!			1	1
#NULL!	#NULL!	#NULL!	#NULL!	#NULL!			1	1
#NULL!	#NULL!	#NULL!	#NULL!	#NULL!			1	1
#NULL!	#NULL!	#NULL!	#NULL!	#NULL!			1	1
#NULL!	#NULL!	#NULL!	#NULL!	#NULL!			1	1
#NULL!	#NULL!	#NULL!	#NULL!	#NULL!			1	1
#NULL!	#NULL!	#NULL!	#NULL!	#NULL!			1	1
#NULL!	#NULL!	#NULL!	#NULL!	#NULL!			1	1
#NULL!	#NULL!	#NULL!	#NULL!	#NULL!			1	1
#NULL!	#NULL!	#NULL!	#NULL!	#NULL!			1	1
#NULL!	#NULL!	#NULL!	#NULL!	#NULL!			1	2
#NULL!	#NULL!	#NULL!	#NULL!	#NULL!			1	1
#NULL!	#NULL!	#NULL!	#NULL!	#NULL!			1	1
#NULL!	#NULL!	#NULL!	#NULL!	#NULL!			1	1
#NULL!	#NULL!	#NULL!	#NULL!	#NULL!			1	1
#NULL!	#NULL!	#NULL!	#NULL!	#NULL!			1	1
#NULL!	#NULL!	#NULL!	#NULL!	#NULL!			1	1
#NULL!	#NULL!	#NULL!	#NULL!	#NULL!			1	1
#NULL!	#NULL!	#NULL!	#NULL!	#NULL!			1	1
#NULL!	#NULL!	#NULL!	#NULL!	#NULL!			1	1
#NULL!	#NULL!	#NULL!	#NULL!	#NULL!			1	1
#NULL!	#NULL!	#NULL!	#NULL!	#NULL!			1	1
#NULL!	#NULL!	#NULL!	#NULL!	#NULL!			1	1
#NULL!	#NULL!	#NULL!	#NULL!	#NULL!			1	1
#NULL!	#NULL!	#NULL!	#NULL!	#NULL!			1	1
#NULL!	#NULL!	#NULL!	#NULL!	#NULL!			1	1
#NULL!	#NULL!	#NULL!	#NULL!	#NULL!			1	1
#NULL!	#NULL!	#NULL!	#NULL!	#NULL!			1	1
#NULL!	#NULL!	#NULL!	#NULL!	#NULL!			1	1
#NULL!	#NULL!	#NULL!	#NULL!	#NULL!			1	1

#NULL!	#NULL!	#NULL!	#NULL!	#NULL!	1	1
#NULL!	#NULL!	#NULL!	#NULL!	#NULL!	1	1
#NULL!	#NULL!	#NULL!	#NULL!	#NULL!	1	1
#NULL!	#NULL!	#NULL!	#NULL!	#NULL!	1	1
#NULL!	#NULL!	#NULL!	#NULL!	#NULL!	1	1
#NULL!	#NULL!	#NULL!	#NULL!	#NULL!	1	1
#NULL!	#NULL!	#NULL!	#NULL!	#NULL!	1	1
#NULL!	#NULL!	#NULL!	#NULL!	#NULL!	1	1
#NULL!	#NULL!	#NULL!	#NULL!	#NULL!	1	1
#NULL!	#NULL!	#NULL!	#NULL!	#NULL!	1	1
#NULL!	#NULL!	#NULL!	#NULL!	#NULL!	1	1
#NULL!	#NULL!	#NULL!	#NULL!	#NULL!	1	1
#NULL!	#NULL!	#NULL!	#NULL!	#NULL!	1	1
#NULL!	#NULL!	#NULL!	#NULL!	#NULL!	1	1
#NULL!	#NULL!	#NULL!	#NULL!	#NULL!	1	1
#NULL!	#NULL!	#NULL!	#NULL!	#NULL!	1	1
#NULL!	#NULL!	#NULL!	#NULL!	#NULL!	1	1
#NULL!	#NULL!	#NULL!	#NULL!	#NULL!	1	1
#NULL!	#NULL!	#NULL!	#NULL!	#NULL!	1	1
#NULL!	#NULL!	#NULL!	#NULL!	#NULL!	1	1
#NULL!	#NULL!	#NULL!	#NULL!	#NULL!	1	1
#NULL!	#NULL!	#NULL!	#NULL!	#NULL!	1	1
#NULL!	#NULL!	#NULL!	#NULL!	#NULL!	1	1
#NULL!	#NULL!	#NULL!	#NULL!	#NULL!	1	1
#NULL!	#NULL!	#NULL!	#NULL!	#NULL!	1	1
#NULL!	#NULL!	#NULL!	#NULL!	#NULL!	1	1
#NULL!	#NULL!	#NULL!	#NULL!	#NULL!	1	1
#NULL!	#NULL!	#NULL!	#NULL!	#NULL!	1	2
#NULL!	#NULL!	#NULL!	#NULL!	#NULL!	1	1
#NULL!	#NULL!	#NULL!	#NULL!	#NULL!	1	1
#NULL!	#NULL!	#NULL!	#NULL!	#NULL!	1	1
#NULL!	#NULL!	#NULL!	#NULL!	#NULL!	1	1
#NULL!	#NULL!	#NULL!	#NULL!	#NULL!	1	1
#NULL!	#NULL!	#NULL!	#NULL!	#NULL!	1	1
#NULL!	#NULL!	#NULL!	#NULL!	#NULL!	1	1
#NULL!	#NULL!	#NULL!	#NULL!	#NULL!	1	1
#NULL!	#NULL!	#NULL!	#NULL!	#NULL!	1	1
#NULL!	#NULL!	#NULL!	#NULL!	#NULL!	1	2
#NULL!	#NULL!	#NULL!	#NULL!	#NULL!	1	1
#NULL!	#NULL!	#NULL!	#NULL!	#NULL!	1	1
#NULL!	#NULL!	#NULL!	#NULL!	#NULL!	1	1
#NULL!	#NULL!	#NULL!	#NULL!	#NULL!	1	1
#NULL!	#NULL!	#NULL!	#NULL!	#NULL!	1	1
#NULL!	#NULL!	#NULL!	#NULL!	#NULL!	1	1
#NULL!	#NULL!	#NULL!	#NULL!	#NULL!	1	2
#NULL!	#NULL!	#NULL!	#NULL!	#NULL!	1	1
#NULL!	#NULL!	#NULL!	#NULL!	#NULL!	1	1

#NULL!	#NULL!	#NULL!	#NULL!	#NULL!	1	1
#NULL!	#NULL!	#NULL!	#NULL!	#NULL!	1	1
#NULL!	#NULL!	#NULL!	#NULL!	#NULL!	1	1
#NULL!	#NULL!	#NULL!	#NULL!	#NULL!	1	1
#NULL!	#NULL!	#NULL!	#NULL!	#NULL!	1	1
#NULL!	#NULL!	#NULL!	#NULL!	#NULL!	1	1
#NULL!	#NULL!	#NULL!	#NULL!	#NULL!	1	1
#NULL!	#NULL!	#NULL!	#NULL!	#NULL!	1	1
#NULL!	#NULL!	#NULL!	#NULL!	#NULL!	1	1
#NULL!	#NULL!	#NULL!	#NULL!	#NULL!	1	1
#NULL!	#NULL!	#NULL!	#NULL!	#NULL!	1	1
#NULL!	#NULL!	#NULL!	#NULL!	#NULL!	1	1
#NULL!	#NULL!	#NULL!	#NULL!	#NULL!	1	1
#NULL!	#NULL!	#NULL!	#NULL!	#NULL!	1	1
#NULL!	#NULL!	#NULL!	#NULL!	#NULL!	1	1
#NULL!	#NULL!	#NULL!	#NULL!	#NULL!	1	1
#NULL!	#NULL!	#NULL!	#NULL!	#NULL!	1	1
#NULL!	#NULL!	#NULL!	#NULL!	#NULL!	1	1
#NULL!	#NULL!	#NULL!	#NULL!	#NULL!	1	1
#NULL!	#NULL!	#NULL!	#NULL!	#NULL!	1	1
#NULL!	#NULL!	#NULL!	#NULL!	#NULL!	1	1
#NULL!	#NULL!	#NULL!	#NULL!	#NULL!	1	1
#NULL!	#NULL!	#NULL!	#NULL!	#NULL!	1	1
#NULL!	#NULL!	#NULL!	#NULL!	#NULL!	1	1
#NULL!	#NULL!	#NULL!	#NULL!	#NULL!	1	1
#NULL!	#NULL!	#NULL!	#NULL!	#NULL!	1	1
#NULL!	#NULL!	#NULL!	#NULL!	#NULL!	1	1
#NULL!	#NULL!	#NULL!	#NULL!	#NULL!	1	1
#NULL!	#NULL!	#NULL!	#NULL!	#NULL!	1	1
#NULL!	#NULL!	#NULL!	#NULL!	#NULL!	1	1
#NULL!	#NULL!	#NULL!	#NULL!	#NULL!	1	1
#NULL!	#NULL!	#NULL!	#NULL!	#NULL!	1	1
#NULL!	#NULL!	#NULL!	#NULL!	#NULL!	1	1
#NULL!	#NULL!	#NULL!	#NULL!	#NULL!	1	1
#NULL!	#NULL!	#NULL!	#NULL!	#NULL!	1	1
#NULL!	#NULL!	#NULL!	#NULL!	#NULL!	1	1
#NULL!	#NULL!	#NULL!	#NULL!	#NULL!	1	1
#NULL!	#NULL!	#NULL!	#NULL!	#NULL!	1	1
#NULL!	#NULL!	#NULL!	#NULL!	#NULL!	1	1
#NULL!	#NULL!	#NULL!	#NULL!	#NULL!	1	1
#NULL!	#NULL!	#NULL!	#NULL!	#NULL!	1	1
#NULL!	#NULL!	#NULL!	#NULL!	#NULL!	1	1
#NULL!	#NULL!	#NULL!	#NULL!	#NULL!	1	1
#NULL!	#NULL!	#NULL!	#NULL!	#NULL!	1	1
#NULL!	#NULL!	#NULL!	#NULL!	#NULL!	1	1
#NULL!	#NULL!	#NULL!	#NULL!	#NULL!	1	1
#NULL!	#NULL!	#NULL!	#NULL!	#NULL!	1	1

#NULL!	#NULL!	#NULL!	#NULL!	#NULL!			1	1
#NULL!	#NULL!	#NULL!	#NULL!	#NULL!			1	1
#NULL!	#NULL!	#NULL!	#NULL!	#NULL!			1	1
#NULL!	#NULL!	#NULL!	#NULL!	#NULL!			1	1
#NULL!	#NULL!	#NULL!	#NULL!	#NULL!			1	1
#NULL!	#NULL!	#NULL!	#NULL!	#NULL!			1	1
#NULL!	#NULL!	#NULL!	#NULL!	#NULL!			1	1
#NULL!	#NULL!	#NULL!	#NULL!	#NULL!			1	1
#NULL!	#NULL!	#NULL!	#NULL!	#NULL!			1	1
3	1	4	2	#NULL!	easily acce	to prepare	1	1
#NULL!	#NULL!	#NULL!	#NULL!	#NULL!			1	1
#NULL!	#NULL!	#NULL!	#NULL!	#NULL!			1	1
#NULL!	#NULL!	#NULL!	#NULL!	#NULL!			1	1
#NULL!	#NULL!	#NULL!	#NULL!	#NULL!			1	1
#NULL!	#NULL!	#NULL!	#NULL!	#NULL!			1	1
#NULL!	#NULL!	#NULL!	#NULL!	#NULL!			1	1
#NULL!	#NULL!	#NULL!	#NULL!	#NULL!			1	1
#NULL!	#NULL!	#NULL!	#NULL!	#NULL!			1	2
#NULL!	#NULL!	#NULL!	#NULL!	#NULL!			1	1
#NULL!	#NULL!	#NULL!	#NULL!	#NULL!			1	1
#NULL!	#NULL!	#NULL!	#NULL!	#NULL!			1	1
#NULL!	#NULL!	#NULL!	#NULL!	#NULL!			1	1
#NULL!	#NULL!	#NULL!	#NULL!	#NULL!			1	1
#NULL!	#NULL!	#NULL!	#NULL!	#NULL!			1	1
#NULL!	#NULL!	#NULL!	#NULL!	#NULL!			1	1
#NULL!	#NULL!	#NULL!	#NULL!	#NULL!			1	1
#NULL!	#NULL!	#NULL!	#NULL!	#NULL!			1	1
#NULL!	#NULL!	#NULL!	#NULL!	#NULL!			1	1
#NULL!	#NULL!	#NULL!	#NULL!	#NULL!			1	1
#NULL!	#NULL!	#NULL!	#NULL!	#NULL!			1	1
#NULL!	#NULL!	#NULL!	#NULL!	#NULL!			1	1
#NULL!	#NULL!	#NULL!	#NULL!	#NULL!			1	1
#NULL!	#NULL!	#NULL!	#NULL!	#NULL!			1	1
#NULL!	#NULL!	#NULL!	#NULL!	#NULL!			1	1
#NULL!	#NULL!	#NULL!	#NULL!	#NULL!			1	2
#NULL!	#NULL!	#NULL!	#NULL!	#NULL!			1	1
#NULL!	#NULL!	#NULL!	#NULL!	#NULL!			1	1
#NULL!	#NULL!	#NULL!	#NULL!	#NULL!			1	1
#NULL!	#NULL!	#NULL!	#NULL!	#NULL!			1	1
#NULL!	#NULL!	#NULL!	#NULL!	#NULL!			1	1
#NULL!	#NULL!	#NULL!	#NULL!	#NULL!			1	1
#NULL!	#NULL!	#NULL!	#NULL!	#NULL!			1	1
#NULL!	#NULL!	#NULL!	#NULL!	#NULL!			1	1
#NULL!	#NULL!	#NULL!	#NULL!	#NULL!			1	1
#NULL!	#NULL!	#NULL!	#NULL!	#NULL!			1	1
#NULL!	#NULL!	#NULL!	#NULL!	#NULL!			1	1

#NULL!	#NULL!	#NULL!	#NULL!	#NULL!		1	1
#NULL!	#NULL!	#NULL!	#NULL!	#NULL!		1	1
#NULL!	#NULL!	#NULL!	#NULL!	#NULL!		1	1
#NULL!	#NULL!	#NULL!	#NULL!	#NULL!		1	1
#NULL!	#NULL!	#NULL!	#NULL!	#NULL!		1	2
#NULL!	#NULL!	#NULL!	#NULL!	#NULL!		1	1
#NULL!	#NULL!	#NULL!	#NULL!	#NULL!		1	1
#NULL!	#NULL!	#NULL!	#NULL!	#NULL!		1	1
#NULL!	#NULL!	#NULL!	#NULL!	#NULL!		1	1
#NULL!	#NULL!	#NULL!	#NULL!	#NULL!		1	1
#NULL!	#NULL!	#NULL!	#NULL!	#NULL!		1	1
#NULL!	#NULL!	#NULL!	#NULL!	#NULL!		1	1
#NULL!	#NULL!	#NULL!	#NULL!	#NULL!		1	1
#NULL!	#NULL!	#NULL!	#NULL!	#NULL!		1	1
#NULL!	#NULL!	#NULL!	#NULL!	#NULL!		1	1
#NULL!	#NULL!	#NULL!	#NULL!	#NULL!		1	1
#NULL!	#NULL!	#NULL!	#NULL!	#NULL!		1	1
#NULL!	#NULL!	#NULL!	#NULL!	#NULL!		1	1
#NULL!	#NULL!	#NULL!	#NULL!	#NULL!		1	1
#NULL!	#NULL!	#NULL!	#NULL!	#NULL!		1	1
#NULL!	#NULL!	#NULL!	#NULL!	#NULL!		1	1
#NULL!	#NULL!	#NULL!	#NULL!	#NULL!		1	1
#NULL!	#NULL!	#NULL!	#NULL!	#NULL!		1	1
#NULL!	#NULL!	#NULL!	#NULL!	#NULL!		1	1
#NULL!	#NULL!	#NULL!	#NULL!	#NULL!		1	1
#NULL!	#NULL!	#NULL!	#NULL!	#NULL!		1	1
#NULL!	#NULL!	#NULL!	#NULL!	#NULL!		1	1
#NULL!	#NULL!	#NULL!	#NULL!	#NULL!		1	1
#NULL!	#NULL!	#NULL!	#NULL!	#NULL!		1	1
#NULL!	#NULL!	#NULL!	#NULL!	#NULL!		1	1
#NULL!	#NULL!	#NULL!	#NULL!	#NULL!		1	1
#NULL!	#NULL!	#NULL!	#NULL!	#NULL!		1	1
#NULL!	#NULL!	#NULL!	#NULL!	#NULL!		1	1
#NULL!	#NULL!	#NULL!	#NULL!	#NULL!		1	1
#NULL!	#NULL!	#NULL!	#NULL!	#NULL!		1	1
#NULL!	#NULL!	#NULL!	#NULL!	#NULL!		1	1
#NULL!	1	2	3	4	Child spaci	1	1
#NULL!	#NULL!	#NULL!	#NULL!	#NULL!		1	1
#NULL!	#NULL!	#NULL!	#NULL!	#NULL!		1	1
#NULL!	#NULL!	#NULL!	#NULL!	#NULL!		1	1
#NULL!	#NULL!	#NULL!	#NULL!	#NULL!		1	1
#NULL!	#NULL!	#NULL!	#NULL!	#NULL!		1	1
#NULL!	#NULL!	#NULL!	#NULL!	#NULL!		1	1
#NULL!	#NULL!	#NULL!	#NULL!	#NULL!		1	1
#NULL!	#NULL!	#NULL!	#NULL!	#NULL!		1	1
#NULL!	#NULL!	#NULL!	#NULL!	#NULL!		1	1
#NULL!	#NULL!	#NULL!	#NULL!	#NULL!		1	1

#NULL!	#NULL!	#NULL!	#NULL!	#NULL!			1	1
#NULL!	#NULL!	#NULL!	#NULL!	#NULL!			1	1
#NULL!	#NULL!	#NULL!	#NULL!	#NULL!			1	1
#NULL!	#NULL!	#NULL!	#NULL!	#NULL!			1	1
#NULL!	#NULL!	#NULL!	#NULL!	#NULL!			1	1
#NULL!	#NULL!	#NULL!	#NULL!	#NULL!			1	1
#NULL!	#NULL!	#NULL!	#NULL!	#NULL!			1	1
#NULL!	#NULL!	#NULL!	#NULL!	#NULL!			1	1
#NULL!	#NULL!	#NULL!	#NULL!	#NULL!			1	1
#NULL!	#NULL!	#NULL!	#NULL!	#NULL!			1	1
#NULL!	#NULL!	#NULL!	#NULL!	#NULL!			1	1
#NULL!	#NULL!	#NULL!	#NULL!	#NULL!			1	1
#NULL!	#NULL!	#NULL!	#NULL!	#NULL!			1	1
#NULL!	#NULL!	#NULL!	#NULL!	#NULL!			1	1
#NULL!	#NULL!	#NULL!	#NULL!	#NULL!			1	1
#NULL!	#NULL!	#NULL!	#NULL!	#NULL!			1	1
#NULL!	#NULL!	#NULL!	#NULL!	#NULL!			1	1
#NULL!	#NULL!	#NULL!	#NULL!	#NULL!			1	1
#NULL!	#NULL!	#NULL!	#NULL!	#NULL!			1	1
#NULL!	#NULL!	#NULL!	#NULL!	#NULL!			1	1
#NULL!	#NULL!	#NULL!	#NULL!	#NULL!			1	1
#NULL!	#NULL!	#NULL!	#NULL!	#NULL!			1	1
#NULL!	#NULL!	#NULL!	#NULL!	#NULL!			1	1
#NULL!	#NULL!	#NULL!	#NULL!	#NULL!			1	1
#NULL!	#NULL!	#NULL!	#NULL!	#NULL!			1	1
#NULL!	#NULL!	#NULL!	#NULL!	#NULL!			1	1
#NULL!	#NULL!	#NULL!	#NULL!	#NULL!			1	1
#NULL!	#NULL!	#NULL!	#NULL!	#NULL!			1	1
#NULL!	#NULL!	#NULL!	#NULL!	#NULL!			1	1
#NULL!	#NULL!	#NULL!	#NULL!	#NULL!			1	1
#NULL!	#NULL!	#NULL!	#NULL!	#NULL!			1	1
#NULL!	#NULL!	#NULL!	#NULL!	#NULL!			1	1
#NULL!	#NULL!	#NULL!	#NULL!	#NULL!			1	1
#NULL!	#NULL!	#NULL!	#NULL!	#NULL!			1	1
#NULL!	#NULL!	#NULL!	#NULL!	#NULL!			1	1
#NULL!	#NULL!	#NULL!	#NULL!	#NULL!			1	1
#NULL!	#NULL!	#NULL!	#NULL!	#NULL!			1	1
#NULL!	#NULL!	#NULL!	#NULL!	#NULL!			1	1
#NULL!	#NULL!	#NULL!	#NULL!	#NULL!			1	1
#NULL!	#NULL!	#NULL!	#NULL!	#NULL!			1	1
#NULL!	#NULL!	#NULL!	#NULL!	#NULL!			1	1
#NULL!	#NULL!	#NULL!	#NULL!	#NULL!			1	1
#NULL!	#NULL!	#NULL!	#NULL!	#NULL!			1	1
#NULL!	#NULL!	#NULL!	#NULL!	#NULL!			1	1
#NULL!	#NULL!	#NULL!	#NULL!	#NULL!			1	1
#NULL!	#NULL!	#NULL!	#NULL!	#NULL!			1	1
#NULL!	#NULL!	#NULL!	#NULL!	#NULL!			1	1

#NULL!	#NULL!	#NULL!	#NULL!	#NULL!			1	1
#NULL!	#NULL!	#NULL!	#NULL!	#NULL!			1	1
3	1	#NULL!	2	4	Easily rever	Having mar	1	1
#NULL!	#NULL!	#NULL!	#NULL!	#NULL!			1	1
#NULL!	#NULL!	#NULL!	#NULL!	#NULL!			1	1
#NULL!	#NULL!	#NULL!	#NULL!	#NULL!			1	1
#NULL!	#NULL!	#NULL!	#NULL!	#NULL!			1	1
#NULL!	#NULL!	#NULL!	#NULL!	#NULL!			1	1
#NULL!	#NULL!	#NULL!	#NULL!	#NULL!			1	1
#NULL!	#NULL!	#NULL!	#NULL!	#NULL!			1	1
#NULL!	#NULL!	#NULL!	#NULL!	#NULL!			1	1
#NULL!	#NULL!	#NULL!	#NULL!	#NULL!			1	1
#NULL!	#NULL!	#NULL!	#NULL!	#NULL!			1	1
#NULL!	#NULL!	#NULL!	#NULL!	#NULL!			1	1
#NULL!	#NULL!	#NULL!	#NULL!	#NULL!			1	1
#NULL!	#NULL!	#NULL!	#NULL!	#NULL!			1	1
#NULL!	#NULL!	#NULL!	#NULL!	#NULL!			1	1
#NULL!	#NULL!	#NULL!	#NULL!	#NULL!			1	1
#NULL!	#NULL!	#NULL!	#NULL!	#NULL!			1	1
#NULL!	#NULL!	#NULL!	#NULL!	#NULL!			1	1
#NULL!	#NULL!	#NULL!	#NULL!	#NULL!			1	1
#NULL!	#NULL!	#NULL!	#NULL!	#NULL!			1	1
#NULL!	#NULL!	#NULL!	#NULL!	#NULL!			1	1
#NULL!	#NULL!	#NULL!	#NULL!	#NULL!			1	1
#NULL!	#NULL!	#NULL!	#NULL!	#NULL!			1	1
#NULL!	#NULL!	#NULL!	#NULL!	#NULL!			1	1
#NULL!	#NULL!	#NULL!	#NULL!	#NULL!			1	1
#NULL!	#NULL!	#NULL!	#NULL!	#NULL!			1	1
#NULL!	#NULL!	#NULL!	#NULL!	#NULL!			1	1
#NULL!	#NULL!	#NULL!	#NULL!	#NULL!			1	1
#NULL!	#NULL!	#NULL!	#NULL!	#NULL!			1	1
#NULL!	#NULL!	#NULL!	#NULL!	#NULL!			1	1
#NULL!	#NULL!	#NULL!	#NULL!	#NULL!			1	2
#NULL!	#NULL!	#NULL!	#NULL!	#NULL!			1	1
#NULL!	#NULL!	#NULL!	#NULL!	#NULL!			1	1
#NULL!	#NULL!	#NULL!	#NULL!	#NULL!			1	1
#NULL!	#NULL!	#NULL!	#NULL!	#NULL!			1	1
#NULL!	#NULL!	#NULL!	#NULL!	#NULL!			1	1
#NULL!	#NULL!	#NULL!	#NULL!	#NULL!			1	1
#NULL!	#NULL!	#NULL!	#NULL!	#NULL!			1	1
#NULL!	#NULL!	#NULL!	#NULL!	#NULL!			1	1
#NULL!	#NULL!	#NULL!	#NULL!	#NULL!			1	1
#NULL!	#NULL!	#NULL!	#NULL!	#NULL!			1	1
#NULL!	#NULL!	#NULL!	#NULL!	#NULL!			1	1
#NULL!	#NULL!	#NULL!	#NULL!	#NULL!			1	1
#NULL!	#NULL!	#NULL!	#NULL!	#NULL!			1	1
#NULL!	#NULL!	#NULL!	#NULL!	#NULL!			1	1

#NULL!	#NULL!	#NULL!	#NULL!	#NULL!			1	1
#NULL!	#NULL!	#NULL!	#NULL!	#NULL!			1	1
#NULL!	#NULL!	#NULL!	#NULL!	#NULL!			1	1
#NULL!	#NULL!	#NULL!	#NULL!	#NULL!			1	1
#NULL!	#NULL!	#NULL!	#NULL!	#NULL!			1	1
#NULL!	#NULL!	#NULL!	#NULL!	#NULL!			1	1
#NULL!	#NULL!	#NULL!	#NULL!	#NULL!			1	1
#NULL!	#NULL!	#NULL!	#NULL!	#NULL!			1	1
#NULL!	#NULL!	#NULL!	#NULL!	#NULL!			1	1
#NULL!	#NULL!	#NULL!	#NULL!	#NULL!			1	1
#NULL!	#NULL!	#NULL!	#NULL!	#NULL!			1	1
#NULL!	#NULL!	#NULL!	#NULL!	#NULL!			1	1
#NULL!	#NULL!	#NULL!	#NULL!	#NULL!			1	1
#NULL!	#NULL!	#NULL!	#NULL!	#NULL!			1	1
#NULL!	#NULL!	#NULL!	#NULL!	#NULL!			1	1
#NULL!	#NULL!	#NULL!	#NULL!	#NULL!			1	1
#NULL!	#NULL!	#NULL!	#NULL!	#NULL!			1	1
#NULL!	#NULL!	#NULL!	#NULL!	#NULL!			1	1
#NULL!	#NULL!	#NULL!	#NULL!	#NULL!			1	1
#NULL!	#NULL!	#NULL!	#NULL!	#NULL!			1	1
#NULL!	#NULL!	#NULL!	#NULL!	#NULL!			1	1
#NULL!	#NULL!	#NULL!	#NULL!	#NULL!			1	1
#NULL!	#NULL!	#NULL!	#NULL!	#NULL!			1	1
#NULL!	#NULL!	#NULL!	#NULL!	#NULL!			1	1
#NULL!	#NULL!	#NULL!	#NULL!	#NULL!			1	1
#NULL!	#NULL!	#NULL!	#NULL!	#NULL!			1	1
#NULL!	#NULL!	#NULL!	#NULL!	#NULL!			1	1
#NULL!	#NULL!	#NULL!	#NULL!	#NULL!			1	1
#NULL!	#NULL!	#NULL!	#NULL!	#NULL!			1	1
#NULL!	#NULL!	#NULL!	#NULL!	#NULL!			1	1
#NULL!	#NULL!	#NULL!	#NULL!	#NULL!			1	1
#NULL!	#NULL!	#NULL!	#NULL!	#NULL!			1	2
#NULL!	#NULL!	#NULL!	#NULL!	#NULL!			1	1
#NULL!	#NULL!	#NULL!	#NULL!	#NULL!			1	1
#NULL!	#NULL!	#NULL!	#NULL!	#NULL!			1	1
#NULL!	#NULL!	#NULL!	#NULL!	#NULL!			1	1
#NULL!	#NULL!	#NULL!	#NULL!	#NULL!			1	1
#NULL!	#NULL!	#NULL!	#NULL!	#NULL!			1	1
#NULL!	#NULL!	#NULL!	#NULL!	#NULL!			1	1
#NULL!	#NULL!	#NULL!	#NULL!	#NULL!			1	1
2	1	#NULL!	#NULL!	#NULL!	It can be st	Based on si	1	1
#NULL!	#NULL!	#NULL!	#NULL!	#NULL!			1	2
#NULL!	#NULL!	#NULL!	#NULL!	#NULL!			1	1
#NULL!	#NULL!	#NULL!	#NULL!	#NULL!			1	1
#NULL!	#NULL!	#NULL!	#NULL!	#NULL!			1	1
#NULL!	#NULL!	#NULL!	#NULL!	#NULL!			1	1
#NULL!	#NULL!	#NULL!	#NULL!	#NULL!			1	1

#NULL!	#NULL!	#NULL!	#NULL!	#NULL!	1	1
#NULL!	#NULL!	#NULL!	#NULL!	#NULL!	1	1
#NULL!	#NULL!	#NULL!	#NULL!	#NULL!	1	1
#NULL!	#NULL!	#NULL!	#NULL!	#NULL!	1	1
#NULL!	#NULL!	#NULL!	#NULL!	#NULL!	1	1
#NULL!	#NULL!	#NULL!	#NULL!	#NULL!	1	1
#NULL!	#NULL!	#NULL!	#NULL!	#NULL!	1	1
#NULL!	#NULL!	#NULL!	#NULL!	#NULL!	1	1
#NULL!	#NULL!	#NULL!	#NULL!	#NULL!	1	1
#NULL!	#NULL!	#NULL!	#NULL!	#NULL!	1	1
#NULL!	#NULL!	#NULL!	#NULL!	#NULL!	1	1
#NULL!	#NULL!	#NULL!	#NULL!	#NULL!	1	1
#NULL!	#NULL!	#NULL!	#NULL!	#NULL!	1	1
#NULL!	#NULL!	#NULL!	#NULL!	#NULL!	1	1
#NULL!	#NULL!	#NULL!	#NULL!	#NULL!	1	1
#NULL!	#NULL!	#NULL!	#NULL!	#NULL!	1	1
#NULL!	#NULL!	#NULL!	#NULL!	#NULL!	1	1
#NULL!	#NULL!	#NULL!	#NULL!	#NULL!	1	1
#NULL!	#NULL!	#NULL!	#NULL!	#NULL!	1	1
#NULL!	#NULL!	#NULL!	#NULL!	#NULL!	1	1
#NULL!	#NULL!	#NULL!	#NULL!	#NULL!	1	1
#NULL!	#NULL!	#NULL!	#NULL!	#NULL!	1	1
#NULL!	#NULL!	#NULL!	#NULL!	#NULL!	1	1
#NULL!	#NULL!	#NULL!	#NULL!	#NULL!	1	1
#NULL!	#NULL!	#NULL!	#NULL!	#NULL!	1	1
#NULL!	#NULL!	#NULL!	#NULL!	#NULL!	1	1
#NULL!	#NULL!	#NULL!	#NULL!	#NULL!	1	1
#NULL!	#NULL!	#NULL!	#NULL!	#NULL!	1	1
#NULL!	#NULL!	#NULL!	#NULL!	#NULL!	1	1
#NULL!	#NULL!	#NULL!	#NULL!	#NULL!	1	1
#NULL!	#NULL!	#NULL!	#NULL!	#NULL!	1	1
#NULL!	#NULL!	#NULL!	#NULL!	#NULL!	1	1
#NULL!	#NULL!	#NULL!	#NULL!	#NULL!	1	1
#NULL!	#NULL!	#NULL!	#NULL!	#NULL!	1	1
#NULL!	#NULL!	#NULL!	#NULL!	#NULL!	1	1
#NULL!	#NULL!	#NULL!	#NULL!	#NULL!	1	1
#NULL!	#NULL!	#NULL!	#NULL!	#NULL!	1	1
#NULL!	#NULL!	#NULL!	#NULL!	#NULL!	1	1
#NULL!	#NULL!	#NULL!	#NULL!	#NULL!	1	1
#NULL!	#NULL!	#NULL!	#NULL!	#NULL!	1	1
#NULL!	#NULL!	#NULL!	#NULL!	#NULL!	1	1
#NULL!	#NULL!	#NULL!	#NULL!	#NULL!	1	1
#NULL!	#NULL!	#NULL!	#NULL!	#NULL!	1	1
#NULL!	#NULL!	#NULL!	#NULL!	#NULL!	1	1
#NULL!	#NULL!	#NULL!	#NULL!	#NULL!	1	1
#NULL!	#NULL!	#NULL!	#NULL!	#NULL!	1	1
#NULL!	#NULL!	#NULL!	#NULL!	#NULL!	1	1

#NULL!	#NULL!	#NULL!	#NULL!	#NULL!			1	1
#NULL!	#NULL!	#NULL!	#NULL!	#NULL!			1	1
#NULL!	#NULL!	#NULL!	#NULL!	#NULL!			1	1
#NULL!	#NULL!	#NULL!	#NULL!	#NULL!			1	1
#NULL!	#NULL!	#NULL!	#NULL!	#NULL!			1	1
#NULL!	#NULL!	#NULL!	#NULL!	#NULL!			1	1
#NULL!	#NULL!	#NULL!	#NULL!	#NULL!			1	1
#NULL!	#NULL!	#NULL!	#NULL!	#NULL!			1	1
#NULL!	#NULL!	#NULL!	#NULL!	#NULL!			1	1
#NULL!	#NULL!	#NULL!	#NULL!	#NULL!			1	1
#NULL!	#NULL!	#NULL!	#NULL!	#NULL!			1	1
#NULL!	#NULL!	#NULL!	#NULL!	#NULL!			1	1
#NULL!	#NULL!	#NULL!	#NULL!	#NULL!			1	1
#NULL!	#NULL!	#NULL!	#NULL!	#NULL!			1	1
#NULL!	#NULL!	#NULL!	#NULL!	#NULL!			1	1
#NULL!	#NULL!	#NULL!	#NULL!	#NULL!			1	1
#NULL!	#NULL!	#NULL!	#NULL!	#NULL!			1	1
#NULL!	#NULL!	#NULL!	#NULL!	#NULL!			1	1
#NULL!	#NULL!	#NULL!	#NULL!	#NULL!			1	1
#NULL!	#NULL!	#NULL!	#NULL!	#NULL!			1	1
#NULL!	#NULL!	#NULL!	#NULL!	#NULL!			1	1
#NULL!	#NULL!	#NULL!	#NULL!	#NULL!			1	1
#NULL!	#NULL!	#NULL!	#NULL!	#NULL!			1	1
#NULL!	#NULL!	#NULL!	#NULL!	#NULL!			1	1
#NULL!	#NULL!	#NULL!	#NULL!	#NULL!			1	1
#NULL!	#NULL!	#NULL!	#NULL!	#NULL!			1	1
#NULL!	#NULL!	#NULL!	#NULL!	#NULL!			1	1
#NULL!	#NULL!	#NULL!	#NULL!	#NULL!			1	1
#NULL!	#NULL!	#NULL!	#NULL!	#NULL!			1	1
#NULL!	#NULL!	#NULL!	#NULL!	#NULL!			1	1
#NULL!	#NULL!	#NULL!	#NULL!	#NULL!			1	1
#NULL!	#NULL!	#NULL!	#NULL!	#NULL!			1	1
#NULL!	#NULL!	#NULL!	#NULL!	#NULL!			1	1
#NULL!	#NULL!	#NULL!	#NULL!	#NULL!			1	1
#NULL!	#NULL!	#NULL!	#NULL!	#NULL!			1	1
#NULL!	#NULL!	#NULL!	#NULL!	#NULL!			1	1
#NULL!	#NULL!	#NULL!	#NULL!	#NULL!			1	1
#NULL!	#NULL!	#NULL!	#NULL!	#NULL!			1	1
#NULL!	#NULL!	#NULL!	#NULL!	#NULL!			1	1
#NULL!	#NULL!	#NULL!	#NULL!	#NULL!			1	1
#NULL!	#NULL!	#NULL!	#NULL!	#NULL!			1	1
#NULL!	#NULL!	#NULL!	#NULL!	#NULL!			1	1
#NULL!	#NULL!	#NULL!	#NULL!	#NULL!			1	1
#NULL!	#NULL!	#NULL!	#NULL!	#NULL!			1	1
#NULL!	#NULL!	#NULL!	#NULL!	#NULL!			1	1
#NULL!	#NULL!	#NULL!	#NULL!	#NULL!			1	1
#NULL!	#NULL!	#NULL!	#NULL!	#NULL!			1	1

#NULL!	#NULL!	#NULL!	#NULL!	#NULL!	1	1
#NULL!	#NULL!	#NULL!	#NULL!	#NULL!	1	1
#NULL!	#NULL!	#NULL!	#NULL!	#NULL!	1	1
#NULL!	#NULL!	#NULL!	#NULL!	#NULL!	1	1
#NULL!	#NULL!	#NULL!	#NULL!	#NULL!	1	1
#NULL!	#NULL!	#NULL!	#NULL!	#NULL!	1	1
#NULL!	#NULL!	#NULL!	#NULL!	#NULL!	1	1
#NULL!	#NULL!	#NULL!	#NULL!	#NULL!	1	1
#NULL!	#NULL!	#NULL!	#NULL!	#NULL!	1	1
#NULL!	#NULL!	#NULL!	#NULL!	#NULL!	1	1
#NULL!	#NULL!	#NULL!	#NULL!	#NULL!	1	1
#NULL!	#NULL!	#NULL!	#NULL!	#NULL!	1	1
#NULL!	#NULL!	#NULL!	#NULL!	#NULL!	1	1
#NULL!	#NULL!	#NULL!	#NULL!	#NULL!	1	1
#NULL!	#NULL!	#NULL!	#NULL!	#NULL!	1	1
#NULL!	#NULL!	#NULL!	#NULL!	#NULL!	1	1
#NULL!	#NULL!	#NULL!	#NULL!	#NULL!	1	1
#NULL!	#NULL!	#NULL!	#NULL!	#NULL!	1	1
#NULL!	#NULL!	#NULL!	#NULL!	#NULL!	1	1
#NULL!	#NULL!	#NULL!	#NULL!	#NULL!	1	1
#NULL!	#NULL!	#NULL!	#NULL!	#NULL!	1	1
#NULL!	#NULL!	#NULL!	#NULL!	#NULL!	1	1
#NULL!	#NULL!	#NULL!	#NULL!	#NULL!	1	1
#NULL!	#NULL!	#NULL!	#NULL!	#NULL!	1	2
#NULL!	#NULL!	#NULL!	#NULL!	#NULL!	1	1
#NULL!	#NULL!	#NULL!	#NULL!	#NULL!	1	1
#NULL!	#NULL!	#NULL!	#NULL!	#NULL!	1	1
#NULL!	#NULL!	#NULL!	#NULL!	#NULL!	1	1
#NULL!	#NULL!	#NULL!	#NULL!	#NULL!	1	1
#NULL!	#NULL!	#NULL!	#NULL!	#NULL!	1	1
#NULL!	#NULL!	#NULL!	#NULL!	#NULL!	1	1
#NULL!	#NULL!	#NULL!	#NULL!	#NULL!	1	1
#NULL!	#NULL!	#NULL!	#NULL!	#NULL!	1	1
#NULL!	#NULL!	#NULL!	#NULL!	#NULL!	1	2
#NULL!	#NULL!	#NULL!	#NULL!	#NULL!	1	1
#NULL!	#NULL!	#NULL!	#NULL!	#NULL!	1	1
#NULL!	#NULL!	#NULL!	#NULL!	#NULL!	1	1
#NULL!	#NULL!	#NULL!	#NULL!	#NULL!	1	2
#NULL!	#NULL!	#NULL!	#NULL!	#NULL!	1	1
#NULL!	#NULL!	#NULL!	#NULL!	#NULL!	1	1
#NULL!	#NULL!	#NULL!	#NULL!	#NULL!	1	1
#NULL!	#NULL!	#NULL!	#NULL!	#NULL!	1	1
#NULL!	#NULL!	#NULL!	#NULL!	#NULL!	1	1
#NULL!	#NULL!	#NULL!	#NULL!	#NULL!	1	2
#NULL!	#NULL!	#NULL!	#NULL!	#NULL!	1	1
#NULL!	#NULL!	#NULL!	#NULL!	#NULL!	1	2
#NULL!	#NULL!	#NULL!	#NULL!	#NULL!	1	1

#NULL!	#NULL!	#NULL!	#NULL!	#NULL!	1	1
#NULL!	#NULL!	#NULL!	#NULL!	#NULL!	1	1
#NULL!	#NULL!	#NULL!	#NULL!	#NULL!	1	1
#NULL!	#NULL!	#NULL!	#NULL!	#NULL!	1	1
#NULL!	#NULL!	#NULL!	#NULL!	#NULL!	1	1
#NULL!	#NULL!	#NULL!	#NULL!	#NULL!	1	1
#NULL!	#NULL!	#NULL!	#NULL!	#NULL!	1	2
#NULL!	#NULL!	#NULL!	#NULL!	#NULL!	1	2
#NULL!	#NULL!	#NULL!	#NULL!	#NULL!	1	2
#NULL!	#NULL!	#NULL!	#NULL!	#NULL!	1	2
#NULL!	#NULL!	#NULL!	#NULL!	#NULL!	1	1
#NULL!	#NULL!	#NULL!	#NULL!	#NULL!	1	1
#NULL!	#NULL!	#NULL!	#NULL!	#NULL!	1	1
#NULL!	#NULL!	#NULL!	#NULL!	#NULL!	1	1

I3ANCdurir	I4WhichM(	I5HowMAN	I6TIMESpo:	J1HowOBT.	J2aNorigyn	J2bDepopr	J2cImplanc	J2dJadelle5
1	1	5	4	x	0	0	1	1
1	1	5	4		0	0	0	0
1	1	5	4	from regior	1	1	1	1
1	2	4	3		0	0	0	0
1	1	5	4	sub-munici	0	1	1	1
1	2	4	4		0	0	0	0
1	1	5	4		0	0	0	0
1	1	5	4	request fro	1	1	1	1
1	1	5	4		0	0	0	0
1	1	5	4	regional mo	0	0	1	1
1	1	5	4		0	0	0	0
1	1	5	4		0	0	0	0
1	1	5	4	receive fror	1	1	0	1
1	1	5	4		0	0	0	0
1	1	5	4		0	0	0	0
1	1	5	4		0	0	0	0
1	1	5	4		0	0	0	0
1	1	5	4		0	0	0	0
1	2	4	4		0	0	0	0
1	1	5	4		0	0	0	0
1	1	5	4		0	0	0	0
1	1	5	4		0	0	0	0
1	1	4	4		0	0	0	0
1	1	5	4		0	0	0	0
1	1	5	4		0	0	0	0
1	1	5	4		0	0	0	0
1	1	5	4		0	0	0	0
1	1	5	4		0	0	0	0
1	1	5	4		0	0	0	0
1	1	5	4		0	0	0	0
1	1	5	4		0	0	0	0
1	1	5	3		0	0	0	0
1	1	5	4		0	0	0	0
1	1	5	4		0	0	0	0
1	1	5	4		0	0	0	0
1	2	5	4		0	0	0	0
1	2	5	4		0	0	0	0
1	1	5	4		0	0	0	0
1	2	5	4		0	0	0	0
1	1	5	4		0	0	0	0
1	1	5	4		0	0	0	0
1	1	5	4		0	0	0	0
1	1	5	4		0	0	0	0
1	1	5	4		0	0	0	0
1	1	5	4		0	0	0	0
1	1	5	4		0	0	0	0

1	1	5	4		0	0	0	0
1	1	5	4		0	0	0	0
1	1	5	4		0	0	0	0
1	1	5	4		0	0	0	0
1	1	5	4		0	0	0	0
1	1	5	4		0	0	0	0
1	1	5	4		0	0	0	0
1	2	5	4		0	0	0	0
1	1	5	4		0	0	0	0
1	1	5	4		0	0	0	0
1	2	4	4		0	0	0	0
1	1	4	4		0	0	0	0
1	1	4	4		0	0	0	0
1	2	4	4		0	0	0	0
1	2	4	4		0	0	0	0
1	1	5	4		0	0	0	0
1	1	4	4		0	0	0	0
1	2	5	4		0	0	0	0
1	1	5	4		0	0	0	0
1	1	5	4		0	0	0	0
2	2	5	4	we get fror	0	1	1	1
1	1	5	4		0	0	0	0
1	1	5	4		0	0	0	0
1	1	5	1		0	0	0	0
1	1	5	4		0	0	0	0
1	1	5	4		0	0	0	0
1	1	5	4		0	0	0	0
1	1	5	4		0	0	0	0
1	1	5	4		0	0	0	0
1	1	5	4		0	0	0	0
1	1	4	3		0	0	0	0
1	1	5	4		0	0	0	0
1	2	5	4		0	0	0	0
1	1	5	4		0	0	0	0
1	1	5	4		0	0	0	0
1	2	5	4		0	0	0	0
1	2	5	4		0	0	0	0
1	1	5	4		0	0	0	0
1	2	4	4		0	0	0	0
1	1	5	4		0	0	0	0
1	1	5	4		0	0	0	0
1	1	5	4		0	0	0	0
1	1	5	4		0	0	0	0
1	1	4	4		0	0	0	0
1	1	5	4		0	0	0	0
1	1	4	4		0	0	0	0
1	1	5	4		0	0	0	0

1	1	4	4	0	0	0	0
1	1	5	4	0	0	0	0
1	1	5	4	0	0	0	0
1	1	5	4	0	0	0	0
1	1	5	4	0	0	0	0
1	1	4	4	0	0	0	0
1	1	4	3	0	0	0	0
1	1	5	3	0	0	0	0
1	1	5	4	0	0	0	0
1	1	4	4	0	0	0	0
1	1	5	4	0	0	0	0
1	1	5	4	0	0	0	0
1	1	5	4	0	0	0	0
1	1	5	4	0	0	0	0
1	1	5	4	0	0	0	0
1	1	5	4	0	0	0	0
1	1	5	4	0	0	0	0
1	1	5	4	0	0	0	0
1	1	5	4	0	0	0	0
1	1	5	5	0	0	0	0
1	1	5	4	0	0	0	0
1	2	5	4	0	0	0	0
1	1	5	4	0	0	0	0
1	1	5	5	0	0	0	0
1	1	5	4	0	0	0	0
1	1	5	4	0	0	0	0
1	2	5	4	0	0	0	0
1	2	5	4	0	0	0	0
1	1	5	4	0	0	0	0
1	1	5	4	0	0	0	0
1	1	5	3	0	0	0	0
1	1	5	4	0	0	0	0
1	1	5	4	0	0	0	0
1	1	5	4	0	0	0	0
1	1	5	4	0	0	0	0
1	1	5	4	0	0	0	0
1	1	5	4	0	0	0	0
1	1	5	4	0	0	0	0
1	2	5	4	0	0	0	0
1	1	5	4	0	0	0	0
1	1	5	4	0	0	0	0
1	1	4	3	0	0	0	0
1	1	5	4	0	0	0	0
1	1	5	4	0	0	0	0
1	1	5	4	0	0	0	0
1	2	4	4	0	0	0	0
1	1	5	4	0	0	0	0

1	1	5	4		0	0	0	0
1	1	5	4		0	0	0	0
1	1	5	4		0	0	0	0
1	2	4	4		0	0	0	0
1	1	5	4		0	0	0	0
1	1	5	4		0	0	0	0
1	1	5	4		0	0	0	0
1	1	5	4		0	0	0	0
1	2	5	4		0	0	0	0
2	2	3	3	from regior	1	1	0	1
1	1	5	4		0	0	0	0
1	1	5	4		0	0	0	0
1	2	4	4		0	0	0	0
1	3	5	4		0	0	0	0
1	3	5	4		0	0	0	0
1	1	5	4		0	0	0	0
1	2	5	4		0	0	0	0
1	1	5	4		0	0	0	0
1	1	5	4		0	0	0	0
1	3	5	4		0	0	0	0
1	3	5	4		0	0	0	0
1	3	4	4		0	0	0	0
1	2	4	4		0	0	0	0
1	3	5	4		0	0	0	0
1	2	5	4		0	0	0	0
1	1	5	4		0	0	0	0
1	2	4	4		0	0	0	0
1	1	4	4		0	0	0	0
1	2	5	4		0	0	0	0
1	1	5	4		0	0	0	0
1	1	5	4		0	0	0	0
1	1	5	4		0	0	0	0
1	1	5	4		0	0	0	0
1	1	5	5		0	0	0	0
1	1	5	4		0	0	0	0
1	1	5	4		0	0	0	0
1	1	5	4		0	0	0	0
1	1	5	4		0	0	0	0
1	2	5	4		0	0	0	0
1	1	5	5		0	0	0	0
1	1	5	5		0	0	0	0
1	1	5	5		0	0	0	0
1	1	5	4		0	0	0	0
1	2	4	4		0	0	0	0
1	1	5	4		0	0	0	0
1	1	5	4		0	0	0	0
1	1	5	4		0	0	0	0

1	3	5	2		0	0	0	0
1	1	5	4		0	0	0	0
1	2	4	4		0	0	0	0
1	2	5	4		0	0	0	0
1	1	5	4		0	0	0	0
1	1	5	4		0	0	0	0
1	1	5	4		0	0	0	0
1	1	5	4		0	0	0	0
1	1	5	4		0	0	0	0
1	2	5	4		0	0	0	0
1	1	5	4		0	0	0	0
1	1	5	4		0	0	0	0
1	2	5	4		0	0	0	0
1	1	5	4		0	0	0	0
1	1	5	4		0	0	0	0
1	1	5	4		0	0	0	0
1	1	5	4		0	0	0	0
1	1	5	4		0	0	0	0
1	1	5	4		0	0	0	0
1	1	5	4		0	0	0	0
1	1	5	4		0	0	0	0
1	1	5	4		0	0	0	0
1	1	5	4		0	0	0	0
1	1	5	4		0	0	0	0
1	1	5	4		0	0	0	0
1	2	5	4		0	0	0	0
1	2	4	3		0	0	0	0
1	1	5	4		0	0	0	0
1	1	5	4		0	0	0	0
1	1	5	4		0	0	0	0
1	1	5	4		0	0	0	0
1	1	5	4		0	0	0	0
1	2	5	4		0	0	0	0
1	1	5	4		0	0	0	0
1	1	5	4		0	0	0	0
1	1	5	4		0	0	0	0
1	1	5	4	Requst fror	0	1	1	1
1	1	5	4		0	0	0	0
1	1	5	4		0	0	0	0
1	1	5	4		0	0	0	0
1	1	5	4		0	0	0	0
1	1	5	4		0	0	0	0
1	1	5	4		0	0	0	0
1	1	5	4		0	0	0	0
1	1	5	4		0	0	0	0
1	1	5	4		0	0	0	0
1	1	5	4		0	0	0	0

1	1	5	4	0	0	0	0
1	1	5	4	0	0	0	0
1	1	5	4	0	0	0	0
1	1	5	4	0	0	0	0
1	1	5	4	0	0	0	0
1	1	5	4	0	0	0	0
1	1	5	4	0	0	0	0
1	1	5	4	0	0	0	0
1	1	5	4	0	0	0	0
1	1	5	4	0	0	0	0
1	1	5	4	0	0	0	0
1	1	5	4	0	0	0	0
1	2	5	4	0	0	0	0
1	1	5	4	0	0	0	0
1	1	5	4	0	0	0	0
1	1	5	4	0	0	0	0
1	1	5	4	0	0	0	0
1	1	5	4	0	0	0	0
1	1	5	4	0	0	0	0
1	1	5	4	0	0	0	0
1	1	5	4	0	0	0	0
1	2	5	4	0	0	0	0
1	2	5	4	0	0	0	0
1	1	5	4	0	0	0	0
1	1	5	4	0	0	0	0
1	2	5	4	0	0	0	0
1	1	4	4	0	0	0	0
1	2	5	3	0	0	0	0
1	1	5	4	0	0	0	0
1	2	3	4	0	0	0	0
1	1	5	4	0	0	0	0
1	1	5	4	0	0	0	0
1	1	5	4	0	0	0	0
1	1	5	4	0	0	0	0
1	1	5	4	0	0	0	0
1	1	5	4	0	0	0	0
1	1	5	4	0	0	0	0
1	1	5	4	0	0	0	0
1	1	5	4	0	0	0	0
1	1	5	4	0	0	0	0
1	1	5	4	0	0	0	0
1	1	5	4	0	0	0	0
1	2	3	4	0	0	0	0
1	1	5	4	0	0	0	0
1	1	5	4	0	0	0	0
1	1	5	4	0	0	0	0
1	2	5	4	0	0	0	0

1	1	5	4		0	0	0	0
1	1	5	4		0	0	0	0
1	1	5	4	Requistion	1	1	0	0
1	1	5	4		0	0	0	0
1	1	5	4		0	0	0	0
1	1	5	4		0	0	0	0
1	2	5	4		0	0	0	0
1	1	5	4		0	0	0	0
1	2	5	4		0	0	0	0
1	1	5	4		0	0	0	0
1	1	5	4		0	0	0	0
1	1	5	4		0	0	0	0
1	1	5	4		0	0	0	0
1	1	5	4	Order from	1	1	0	1
1	2	5	4		0	0	0	0
1	1	5	4		0	0	0	0
1	2	5	4		0	0	0	0
1	1	5	4		0	0	0	0
1	1	5	4		0	0	0	0
1	1	5	4		0	0	0	0
1	1	5	4		0	0	0	0
1	1	5	4		0	0	0	0
1	1	5	4		0	0	0	0
1	1	5	4		0	0	0	0
1	1	5	4		0	0	0	0
1	2	5	4		0	0	0	0
1	1	5	4		0	0	0	0
1	1	5	4		0	0	0	0
1	1	5	4		0	0	0	0
1	2	4	4		0	0	0	0
1	1	5	4		0	0	0	0
1	1	5	4		0	0	0	0
1	1	5	4		0	0	0	0
1	1	5	4		0	0	0	0
1	1	5	4		0	0	0	0
1	1	5	4		0	0	0	0
1	1	5	4		0	0	0	0
1	1	5	4		0	0	0	0
1	1	5	4		0	0	0	0
1	1	5	4		0	0	0	0
1	1	5	4		0	0	0	0
1	1	5	4		0	0	0	0
1	1	5	4		0	0	0	0
1	1	5	4		0	0	0	0
1	1	5	4		0	0	0	0
1	1	5	4		0	0	0	0
1	1	5	4		0	0	0	0

1	1	5	4		0	0	0	0
1	1	5	4		0	0	0	0
1	1	5	4		0	0	0	0
1	1	5	4		0	0	0	0
1	1	5	4		0	0	0	0
1	1	5	4		0	0	0	0
1	1	5	4		0	0	0	0
1	1	5	4		0	0	0	0
1	1	5	4		0	0	0	0
1	1	5	4		0	0	0	0
1	1	5	4		0	0	0	0
1	1	5	4		0	0	0	0
1	1	5	4		0	0	0	0
1	1	5	4		0	0	0	0
1	1	5	4		0	0	0	0
1	1	5	4		0	0	0	0
1	1	6	4		0	0	0	0
1	1	5	4		0	0	0	0
1	1	5	5		0	0	0	0
1	1	5	5		0	0	0	0
1	1	5	5		0	0	0	0
1	1	5	4		0	0	0	0
1	1	5	4		0	0	0	0
1	1	5	4		0	0	0	0
1	1	5	4		0	0	0	0
1	1	5	4		0	0	0	0
1	1	5	4		0	0	0	0
1	1	5	4		0	0	0	0
1	1	5	4		0	0	0	0
1	1	5	4		0	0	0	0
1	1	5	4		0	0	0	0
1	2	5	4		0	0	0	0
1	1	3	2		0	0	0	0
1	2	5	4		0	0	0	0
1	1	5	4		0	0	0	0
1	1	5	4		0	0	0	0
1	1	5	4		0	0	0	0
1	1	5	4		0	0	0	0
1	1	5	4		0	0	0	0
1	1	5	4		0	0	0	0
1	1	5	4	Sub-munici	1	1	0	1
1	2	5	4		0	0	0	0
1	1	5	4		0	0	0	0
1	1	5	4		0	0	0	0
1	2	5	4		0	0	0	0
1	1	5	4		0	0	0	0
1	1	5	4		0	0	0	0

1	1	5	4	0	0	0	0
1	1	5	4	0	0	0	0
1	1	5	4	0	0	0	0
1	1	5	4	0	0	0	0
1	1	5	4	0	0	0	0
1	1	5	4	0	0	0	0
1	1	5	4	0	0	0	0
1	1	5	4	0	0	0	0
1	1	5	4	0	0	0	0
1	1	5	4	0	0	0	0
1	1	5	5	0	0	0	0
1	1	5	4	0	0	0	0
1	1	5	4	0	0	0	0
1	1	5	4	0	0	0	0
1	1	5	4	0	0	0	0
1	1	5	4	0	0	0	0
1	1	5	4	0	0	0	0
1	1	5	4	0	0	0	0
1	1	5	4	0	0	0	0
1	1	5	4	0	0	0	0
1	1	5	4	0	0	0	0
1	1	5	4	0	0	0	0
1	1	5	4	0	0	0	0
1	1	5	4	0	0	0	0
1	1	5	4	0	0	0	0
1	1	5	4	0	0	0	0
1	1	5	4	0	0	0	0
1	1	5	4	0	0	0	0
1	1	5	4	0	0	0	0
1	1	5	4	0	0	0	0
1	1	5	4	0	0	0	0
1	1	5	4	0	0	0	0
1	1	5	4	0	0	0	0
1	1	5	4	0	0	0	0
1	1	5	4	0	0	0	0
1	1	5	4	0	0	0	0
1	1	5	4	0	0	0	0
1	1	5	4	0	0	0	0
1	1	5	4	0	0	0	0
1	1	5	4	0	0	0	0
1	1	5	4	0	0	0	0
1	1	5	4	0	0	0	0
1	1	5	4	0	0	0	0
1	1	5	4	0	0	0	0
1	1	5	4	0	0	0	0
1	1	5	4	0	0	0	0
1	1	5	4	0	0	0	0

1	1	5	4	0	0	0	0
1	1	5	4	0	0	0	0
1	1	5	4	0	0	0	0
1	1	5	4	0	0	0	0
1	1	5	4	0	0	0	0
1	1	5	4	0	0	0	0
1	1	5	4	0	0	0	0
1	1	5	4	0	0	0	0
1	1	5	4	0	0	0	0
1	1	5	4	0	0	0	0
1	1	5	4	0	0	0	0
1	1	5	4	0	0	0	0
1	1	5	4	0	0	0	0
1	1	5	4	0	0	0	0
1	1	5	4	0	0	0	0
1	1	5	4	0	0	0	0
1	1	5	4	0	0	0	0
1	1	5	4	0	0	0	0
1	1	5	4	0	0	0	0
1	1	5	4	0	0	0	0
1	1	6	5	0	0	0	0
1	1	5	4	0	0	0	0
1	1	5	4	0	0	0	0
1	1	5	4	0	0	0	0
1	1	5	4	0	0	0	0
1	1	5	4	0	0	0	0
1	1	5	5	0	0	0	0
1	1	5	4	0	0	0	0
1	1	5	4	0	0	0	0
1	1	5	4	0	0	0	0
1	1	5	5	0	0	0	0
1	1	5	4	0	0	0	0
1	1	5	4	0	0	0	0
1	1	5	4	0	0	0	0
1	1	5	4	0	0	0	0
1	1	5	4	0	0	0	0
1	1	5	4	0	0	0	0
1	1	5	4	0	0	0	0
1	1	5	4	0	0	0	0
1	1	5	4	0	0	0	0
1	1	5	4	0	0	0	0
1	1	5	4	0	0	0	0
1	1	5	4	0	0	0	0
1	1	5	4	0	0	0	0
1	1	5	5	0	0	0	0
1	1	5	4	0	0	0	0
1	1	5	4	0	0	0	0

1	1	5	4	0	0	0	0
1	1	5	4	0	0	0	0
1	1	5	4	0	0	0	0
1	1	5	5	0	0	0	0
1	1	5	4	0	0	0	0
1	1	5	4	0	0	0	0
1	1	5	4	0	0	0	0
1	1	1	1	0	0	0	0
1	1	5	4	0	0	0	0
1	1	5	4	0	0	0	0
1	1	5	4	0	0	0	0
1	1	5	4	0	0	0	0
1	1	4	5	0	0	0	0
1	1	5	5	0	0	0	0
1	1	5	4	0	0	0	0
1	1	5	4	0	0	0	0
1	1	5	4	0	0	0	0
1	1	5	4	0	0	0	0
1	1	5	4	0	0	0	0
1	1	5	4	0	0	0	0
1	1	5	4	0	0	0	0
1	1	5	4	0	0	0	0
1	1	5	4	0	0	0	0
1	1	5	4	0	0	0	0
1	1	5	4	0	0	0	0
1	1	5	4	0	0	0	0
1	2	4	4	0	0	0	0
1	2	4	3	0	0	0	0
1	2	5	4	0	0	0	0
1	1	5	4	0	0	0	0
1	1	5	4	0	0	0	0
1	1	5	4	0	0	0	0
1	1	5	4	0	0	0	0
1	2	5	4	0	0	0	0
1	1	5	4	0	0	0	0
1	1	5	4	0	0	0	0
2	2	5	4	0	0	0	0
1	1	5	1	0	0	0	0
1	1	5	4	0	0	0	0
1	1	5	4	0	0	0	0
1	1	5	4	0	0	0	0
1	2	5	4	0	0	0	0
1	2	5	4	0	0	0	0
1	1	5	4	0	0	0	0
1	1	5	4	0	0	0	0
1	1	5	4	0	0	0	0
1	1	5	4	0	0	0	0

1	1	5	4	0	0	0	0
1	1	5	4	0	0	0	0
1	1	5	4	0	0	0	0
1	2	5	4	0	0	0	0
1	1	5	4	0	0	0	0
1	1	5	4	0	0	0	0
1	1	5	4	0	0	0	0
1	2	5	4	0	0	0	0
1	2	5	4	0	0	0	0
1	2	5	4	0	0	0	0
1	1	5	4	0	0	0	0
1	1	5	4	0	0	0	0
1	1	4	4	0	0	0	0
1	1	5	4	0	0	0	0

J2eMicrogy	J7Whichoft	J7aOthercc	J8aNorigyn	J8bDepopr	J8cImplanc	J8dJadelle5	Microgynoi	J9Haveyou
0		Jadele, Imp	1	0	2	3	4	according t
0			#NULL!	#NULL!	#NULL!	#NULL!	#NULL!	
1	3. injectables-Depo		1	0	2	3	4	safe and cc
0			#NULL!	#NULL!	#NULL!	#NULL!	#NULL!	
0			4	0	1	2	3	the order is
0			#NULL!	#NULL!	#NULL!	#NULL!	#NULL!	
0			#NULL!	#NULL!	#NULL!	#NULL!	#NULL!	
0			3	0	2	4	1	it is accordi
0			#NULL!	#NULL!	#NULL!	#NULL!	#NULL!	
0	6. Other (sp	jadelle, imp	1	0	2	3	4	the supply i
0			#NULL!	#NULL!	#NULL!	#NULL!	#NULL!	
0			#NULL!	#NULL!	#NULL!	#NULL!	#NULL!	
1		all in the qu	4	0	1	2	3	depends or
0			#NULL!	#NULL!	#NULL!	#NULL!	#NULL!	
0			#NULL!	#NULL!	#NULL!	#NULL!	#NULL!	
0			#NULL!	#NULL!	#NULL!	#NULL!	#NULL!	
0			#NULL!	#NULL!	#NULL!	#NULL!	#NULL!	
0			#NULL!	#NULL!	#NULL!	#NULL!	#NULL!	
0			#NULL!	#NULL!	#NULL!	#NULL!	#NULL!	
0			#NULL!	#NULL!	#NULL!	#NULL!	#NULL!	
0			#NULL!	#NULL!	#NULL!	#NULL!	#NULL!	
0			#NULL!	#NULL!	#NULL!	#NULL!	#NULL!	
0			#NULL!	#NULL!	#NULL!	#NULL!	#NULL!	
0			#NULL!	#NULL!	#NULL!	#NULL!	#NULL!	
0			#NULL!	#NULL!	#NULL!	#NULL!	#NULL!	
0			#NULL!	#NULL!	#NULL!	#NULL!	#NULL!	
0			#NULL!	#NULL!	#NULL!	#NULL!	#NULL!	
0			#NULL!	#NULL!	#NULL!	#NULL!	#NULL!	
0			#NULL!	#NULL!	#NULL!	#NULL!	#NULL!	
0			#NULL!	#NULL!	#NULL!	#NULL!	#NULL!	
0			#NULL!	#NULL!	#NULL!	#NULL!	#NULL!	
0			#NULL!	#NULL!	#NULL!	#NULL!	#NULL!	
0			#NULL!	#NULL!	#NULL!	#NULL!	#NULL!	
0			#NULL!	#NULL!	#NULL!	#NULL!	#NULL!	
0			#NULL!	#NULL!	#NULL!	#NULL!	#NULL!	
0			#NULL!	#NULL!	#NULL!	#NULL!	#NULL!	
0			#NULL!	#NULL!	#NULL!	#NULL!	#NULL!	
0			#NULL!	#NULL!	#NULL!	#NULL!	#NULL!	
0			#NULL!	#NULL!	#NULL!	#NULL!	#NULL!	
0			#NULL!	#NULL!	#NULL!	#NULL!	#NULL!	
0			#NULL!	#NULL!	#NULL!	#NULL!	#NULL!	
0			#NULL!	#NULL!	#NULL!	#NULL!	#NULL!	
0			#NULL!	#NULL!	#NULL!	#NULL!	#NULL!	
0			#NULL!	#NULL!	#NULL!	#NULL!	#NULL!	
0			#NULL!	#NULL!	#NULL!	#NULL!	#NULL!	
0			#NULL!	#NULL!	#NULL!	#NULL!	#NULL!	

0			#NULL!	#NULL!	#NULL!	#NULL!	#NULL!	
0			#NULL!	#NULL!	#NULL!	#NULL!	#NULL!	
0			#NULL!	#NULL!	#NULL!	#NULL!	#NULL!	
0			#NULL!	#NULL!	#NULL!	#NULL!	#NULL!	
0			#NULL!	#NULL!	#NULL!	#NULL!	#NULL!	
0			#NULL!	#NULL!	#NULL!	#NULL!	#NULL!	
0			#NULL!	#NULL!	#NULL!	#NULL!	#NULL!	
0			#NULL!	#NULL!	#NULL!	#NULL!	#NULL!	
0			#NULL!	#NULL!	#NULL!	#NULL!	#NULL!	
0			#NULL!	#NULL!	#NULL!	#NULL!	#NULL!	
0			#NULL!	#NULL!	#NULL!	#NULL!	#NULL!	
0			#NULL!	#NULL!	#NULL!	#NULL!	#NULL!	
0			#NULL!	#NULL!	#NULL!	#NULL!	#NULL!	
0			#NULL!	#NULL!	#NULL!	#NULL!	#NULL!	1
0			#NULL!	#NULL!	#NULL!	#NULL!	#NULL!	
0			#NULL!	#NULL!	#NULL!	#NULL!	#NULL!	
0			#NULL!	#NULL!	#NULL!	#NULL!	#NULL!	
0			#NULL!	#NULL!	#NULL!	#NULL!	#NULL!	
0			#NULL!	#NULL!	#NULL!	#NULL!	#NULL!	
0			#NULL!	#NULL!	#NULL!	#NULL!	#NULL!	
0	1. Jadelle	inplanon, d	#NULL!	0	2	3	#NULL!	2
0			#NULL!	#NULL!	#NULL!	#NULL!	#NULL!	
0			#NULL!	#NULL!	#NULL!	#NULL!	#NULL!	
0			#NULL!	#NULL!	#NULL!	#NULL!	#NULL!	
0			#NULL!	#NULL!	#NULL!	#NULL!	#NULL!	
0			#NULL!	#NULL!	#NULL!	#NULL!	#NULL!	
0			#NULL!	#NULL!	#NULL!	#NULL!	#NULL!	
0			#NULL!	#NULL!	#NULL!	#NULL!	#NULL!	
0			#NULL!	#NULL!	#NULL!	#NULL!	#NULL!	
0			#NULL!	#NULL!	#NULL!	#NULL!	#NULL!	
0			#NULL!	#NULL!	#NULL!	#NULL!	#NULL!	
0			#NULL!	#NULL!	#NULL!	#NULL!	#NULL!	
0			#NULL!	#NULL!	#NULL!	#NULL!	#NULL!	
0			#NULL!	#NULL!	#NULL!	#NULL!	#NULL!	
0			#NULL!	#NULL!	#NULL!	#NULL!	#NULL!	
0			#NULL!	#NULL!	#NULL!	#NULL!	#NULL!	
0			#NULL!	#NULL!	#NULL!	#NULL!	#NULL!	
0			#NULL!	#NULL!	#NULL!	#NULL!	#NULL!	
0			#NULL!	#NULL!	#NULL!	#NULL!	#NULL!	
0			#NULL!	#NULL!	#NULL!	#NULL!	#NULL!	
0			#NULL!	#NULL!	#NULL!	#NULL!	#NULL!	
0			#NULL!	#NULL!	#NULL!	#NULL!	#NULL!	
0			#NULL!	#NULL!	#NULL!	#NULL!	#NULL!	
0			#NULL!	#NULL!	#NULL!	#NULL!	#NULL!	
0			#NULL!	#NULL!	#NULL!	#NULL!	#NULL!	
0			#NULL!	#NULL!	#NULL!	#NULL!	#NULL!	
0			#NULL!	#NULL!	#NULL!	#NULL!	#NULL!	

0	#NULL!	#NULL!	#NULL!	#NULL!	#NULL!
0	#NULL!	#NULL!	#NULL!	#NULL!	#NULL!
0	#NULL!	#NULL!	#NULL!	#NULL!	#NULL!
0	#NULL!	#NULL!	#NULL!	#NULL!	#NULL!
0	#NULL!	#NULL!	#NULL!	#NULL!	#NULL!
0	#NULL!	#NULL!	#NULL!	#NULL!	#NULL!
0	#NULL!	#NULL!	#NULL!	#NULL!	#NULL!
0	#NULL!	#NULL!	#NULL!	#NULL!	#NULL!
0	#NULL!	#NULL!	#NULL!	#NULL!	#NULL!
0	#NULL!	#NULL!	#NULL!	#NULL!	#NULL!
0	#NULL!	#NULL!	#NULL!	#NULL!	#NULL!
0	#NULL!	#NULL!	#NULL!	#NULL!	#NULL!
0	#NULL!	#NULL!	#NULL!	#NULL!	#NULL!
0	#NULL!	#NULL!	#NULL!	#NULL!	#NULL!
0	#NULL!	#NULL!	#NULL!	#NULL!	#NULL!
0	#NULL!	#NULL!	#NULL!	#NULL!	#NULL!
0	#NULL!	#NULL!	#NULL!	#NULL!	#NULL!
0	#NULL!	#NULL!	#NULL!	#NULL!	#NULL!
0	#NULL!	#NULL!	#NULL!	#NULL!	#NULL!
0	#NULL!	#NULL!	#NULL!	#NULL!	#NULL!
0	#NULL!	#NULL!	#NULL!	#NULL!	#NULL!
0	#NULL!	#NULL!	#NULL!	#NULL!	#NULL!
0	#NULL!	#NULL!	#NULL!	#NULL!	#NULL!
0	#NULL!	#NULL!	#NULL!	#NULL!	#NULL!
0	#NULL!	#NULL!	#NULL!	#NULL!	#NULL!
0	#NULL!	#NULL!	#NULL!	#NULL!	#NULL!
0	#NULL!	#NULL!	#NULL!	#NULL!	#NULL!
0	#NULL!	#NULL!	#NULL!	#NULL!	#NULL!
0	#NULL!	#NULL!	#NULL!	#NULL!	#NULL!
0	#NULL!	#NULL!	#NULL!	#NULL!	#NULL!
0	#NULL!	#NULL!	#NULL!	#NULL!	#NULL!
0	#NULL!	#NULL!	#NULL!	#NULL!	#NULL!
0	#NULL!	#NULL!	#NULL!	#NULL!	#NULL!
0	#NULL!	#NULL!	#NULL!	#NULL!	#NULL!
0	#NULL!	#NULL!	#NULL!	#NULL!	#NULL!
0	#NULL!	#NULL!	#NULL!	#NULL!	#NULL!
0	#NULL!	#NULL!	#NULL!	#NULL!	#NULL!
0	#NULL!	#NULL!	#NULL!	#NULL!	#NULL!
0	#NULL!	#NULL!	#NULL!	#NULL!	#NULL!
0	#NULL!	#NULL!	#NULL!	#NULL!	#NULL!
0	#NULL!	#NULL!	#NULL!	#NULL!	#NULL!
0	#NULL!	#NULL!	#NULL!	#NULL!	#NULL!
0	#NULL!	#NULL!	#NULL!	#NULL!	#NULL!
0	#NULL!	#NULL!	#NULL!	#NULL!	#NULL!
0	#NULL!	#NULL!	#NULL!	#NULL!	#NULL!
0	#NULL!	#NULL!	#NULL!	#NULL!	#NULL!
0	#NULL!	#NULL!	#NULL!	#NULL!	#NULL!

0		#NULL!	#NULL!	#NULL!	#NULL!	#NULL!
0		#NULL!	#NULL!	#NULL!	#NULL!	#NULL!
0		#NULL!	#NULL!	#NULL!	#NULL!	#NULL!
0		#NULL!	#NULL!	#NULL!	#NULL!	#NULL!
0		#NULL!	#NULL!	#NULL!	#NULL!	#NULL!
0		#NULL!	#NULL!	#NULL!	#NULL!	#NULL!
0		#NULL!	#NULL!	#NULL!	#NULL!	#NULL!
0		#NULL!	#NULL!	#NULL!	#NULL!	#NULL!
0		#NULL!	#NULL!	#NULL!	#NULL!	#NULL!
0	1. Jadelle	2	1	0	3	0
0		#NULL!	#NULL!	#NULL!	#NULL!	#NULL!
0		#NULL!	#NULL!	#NULL!	#NULL!	#NULL!
0		#NULL!	#NULL!	#NULL!	#NULL!	#NULL!
0		#NULL!	#NULL!	#NULL!	#NULL!	#NULL!
0		#NULL!	#NULL!	#NULL!	#NULL!	#NULL!
0		#NULL!	#NULL!	#NULL!	#NULL!	#NULL!
0		#NULL!	#NULL!	#NULL!	#NULL!	#NULL!
0		#NULL!	#NULL!	#NULL!	#NULL!	#NULL!
0		#NULL!	#NULL!	#NULL!	#NULL!	#NULL!
0		#NULL!	#NULL!	#NULL!	#NULL!	#NULL!
0		#NULL!	#NULL!	#NULL!	#NULL!	#NULL!
0		#NULL!	#NULL!	#NULL!	#NULL!	#NULL!
0		#NULL!	#NULL!	#NULL!	#NULL!	#NULL!
0		#NULL!	#NULL!	#NULL!	#NULL!	#NULL!
0		#NULL!	#NULL!	#NULL!	#NULL!	#NULL!
0		#NULL!	#NULL!	#NULL!	#NULL!	#NULL!
0		#NULL!	#NULL!	#NULL!	#NULL!	#NULL!
0		#NULL!	#NULL!	#NULL!	#NULL!	#NULL!
0		#NULL!	#NULL!	#NULL!	#NULL!	#NULL!
0		#NULL!	#NULL!	#NULL!	#NULL!	#NULL!
0		#NULL!	#NULL!	#NULL!	#NULL!	#NULL!
0		#NULL!	#NULL!	#NULL!	#NULL!	#NULL!
0		#NULL!	#NULL!	#NULL!	#NULL!	#NULL!
0		#NULL!	#NULL!	#NULL!	#NULL!	#NULL!
0		#NULL!	#NULL!	#NULL!	#NULL!	#NULL!
0		#NULL!	#NULL!	#NULL!	#NULL!	#NULL!
0		#NULL!	#NULL!	#NULL!	#NULL!	#NULL!
0		#NULL!	#NULL!	#NULL!	#NULL!	#NULL!
0		#NULL!	#NULL!	#NULL!	#NULL!	#NULL!
0		#NULL!	#NULL!	#NULL!	#NULL!	#NULL!
0		#NULL!	#NULL!	#NULL!	#NULL!	#NULL!
0		#NULL!	#NULL!	#NULL!	#NULL!	#NULL!
0		#NULL!	#NULL!	#NULL!	#NULL!	#NULL!
0		#NULL!	#NULL!	#NULL!	#NULL!	#NULL!
0		#NULL!	#NULL!	#NULL!	#NULL!	#NULL!
0		#NULL!	#NULL!	#NULL!	#NULL!	#NULL!
0		#NULL!	#NULL!	#NULL!	#NULL!	#NULL!

0		#NULL!	#NULL!	#NULL!	#NULL!	#NULL!	
0		#NULL!	#NULL!	#NULL!	#NULL!	#NULL!	
0		#NULL!	#NULL!	#NULL!	#NULL!	#NULL!	
0		#NULL!	#NULL!	#NULL!	#NULL!	#NULL!	
0		#NULL!	#NULL!	#NULL!	#NULL!	#NULL!	
0		#NULL!	#NULL!	#NULL!	#NULL!	#NULL!	
0		#NULL!	#NULL!	#NULL!	#NULL!	#NULL!	
0		#NULL!	#NULL!	#NULL!	#NULL!	#NULL!	
0		#NULL!	#NULL!	#NULL!	#NULL!	#NULL!	
0		#NULL!	#NULL!	#NULL!	#NULL!	#NULL!	
0		#NULL!	#NULL!	#NULL!	#NULL!	#NULL!	
0		#NULL!	#NULL!	#NULL!	#NULL!	#NULL!	
0		#NULL!	#NULL!	#NULL!	#NULL!	#NULL!	
0		#NULL!	#NULL!	#NULL!	#NULL!	#NULL!	
0		#NULL!	#NULL!	#NULL!	#NULL!	#NULL!	
0		#NULL!	#NULL!	#NULL!	#NULL!	#NULL!	
0		#NULL!	#NULL!	#NULL!	#NULL!	#NULL!	
0		#NULL!	#NULL!	#NULL!	#NULL!	#NULL!	
0		#NULL!	#NULL!	#NULL!	#NULL!	#NULL!	
0		#NULL!	#NULL!	#NULL!	#NULL!	#NULL!	
0		#NULL!	#NULL!	#NULL!	#NULL!	#NULL!	
0		#NULL!	#NULL!	#NULL!	#NULL!	#NULL!	
0		#NULL!	#NULL!	#NULL!	#NULL!	#NULL!	
0		#NULL!	#NULL!	#NULL!	#NULL!	#NULL!	
0		#NULL!	#NULL!	#NULL!	#NULL!	#NULL!	
0		#NULL!	#NULL!	#NULL!	#NULL!	#NULL!	
0		#NULL!	#NULL!	#NULL!	#NULL!	#NULL!	
0		#NULL!	#NULL!	#NULL!	#NULL!	#NULL!	
0		#NULL!	#NULL!	#NULL!	#NULL!	#NULL!	
0		#NULL!	#NULL!	#NULL!	#NULL!	#NULL!	
0		#NULL!	#NULL!	#NULL!	#NULL!	#NULL!	
0		#NULL!	#NULL!	#NULL!	#NULL!	#NULL!	
0		#NULL!	#NULL!	#NULL!	#NULL!	#NULL!	
0		#NULL!	#NULL!	#NULL!	#NULL!	#NULL!	
0		#NULL!	#NULL!	#NULL!	#NULL!	#NULL!	
0		#NULL!	#NULL!	#NULL!	#NULL!	#NULL!	
1	3. injectables-Depo	2	1	4	#NULL!	3	Always ava
0		#NULL!	#NULL!	#NULL!	#NULL!	#NULL!	
0		#NULL!	#NULL!	#NULL!	#NULL!	#NULL!	
0		#NULL!	#NULL!	#NULL!	#NULL!	#NULL!	
0		#NULL!	#NULL!	#NULL!	#NULL!	#NULL!	
0		#NULL!	#NULL!	#NULL!	#NULL!	#NULL!	
0		#NULL!	#NULL!	#NULL!	#NULL!	#NULL!	
0		#NULL!	#NULL!	#NULL!	#NULL!	#NULL!	
0		#NULL!	#NULL!	#NULL!	#NULL!	#NULL!	
0		#NULL!	#NULL!	#NULL!	#NULL!	#NULL!	
0		#NULL!	#NULL!	#NULL!	#NULL!	#NULL!	

0	#NULL!	#NULL!	#NULL!	#NULL!	#NULL!
0	#NULL!	#NULL!	#NULL!	#NULL!	#NULL!
0	#NULL!	#NULL!	#NULL!	#NULL!	#NULL!
0	#NULL!	#NULL!	#NULL!	#NULL!	#NULL!
0	#NULL!	#NULL!	#NULL!	#NULL!	#NULL!
0	#NULL!	#NULL!	#NULL!	#NULL!	#NULL!
0	#NULL!	#NULL!	#NULL!	#NULL!	#NULL!
0	#NULL!	#NULL!	#NULL!	#NULL!	#NULL!
0	#NULL!	#NULL!	#NULL!	#NULL!	#NULL!
0	#NULL!	#NULL!	#NULL!	#NULL!	#NULL!
0	#NULL!	#NULL!	#NULL!	#NULL!	#NULL!
0	#NULL!	#NULL!	#NULL!	#NULL!	#NULL!
0	#NULL!	#NULL!	#NULL!	#NULL!	#NULL!
0	#NULL!	#NULL!	#NULL!	#NULL!	#NULL!
0	#NULL!	#NULL!	#NULL!	#NULL!	#NULL!
0	#NULL!	#NULL!	#NULL!	#NULL!	#NULL!
0	#NULL!	#NULL!	#NULL!	#NULL!	#NULL!
0	#NULL!	#NULL!	#NULL!	#NULL!	#NULL!
0	#NULL!	#NULL!	#NULL!	#NULL!	#NULL!
0	#NULL!	#NULL!	#NULL!	#NULL!	#NULL!
0	#NULL!	#NULL!	#NULL!	#NULL!	#NULL!
0	#NULL!	#NULL!	#NULL!	#NULL!	#NULL!
0	#NULL!	#NULL!	#NULL!	#NULL!	#NULL!
0	#NULL!	#NULL!	#NULL!	#NULL!	#NULL!
0	#NULL!	#NULL!	#NULL!	#NULL!	#NULL!
0	#NULL!	#NULL!	#NULL!	#NULL!	#NULL!
0	#NULL!	#NULL!	#NULL!	#NULL!	#NULL!
0	#NULL!	#NULL!	#NULL!	#NULL!	#NULL!
0	#NULL!	#NULL!	#NULL!	#NULL!	#NULL!
0	#NULL!	#NULL!	#NULL!	#NULL!	#NULL!
0	#NULL!	#NULL!	#NULL!	#NULL!	#NULL!
0	#NULL!	#NULL!	#NULL!	#NULL!	#NULL!
0	#NULL!	#NULL!	#NULL!	#NULL!	#NULL!
0	#NULL!	#NULL!	#NULL!	#NULL!	#NULL!
0	#NULL!	#NULL!	#NULL!	#NULL!	#NULL!
0	#NULL!	#NULL!	#NULL!	#NULL!	#NULL!
0	#NULL!	#NULL!	#NULL!	#NULL!	#NULL!
0	#NULL!	#NULL!	#NULL!	#NULL!	#NULL!
0	#NULL!	#NULL!	#NULL!	#NULL!	#NULL!
0	#NULL!	#NULL!	#NULL!	#NULL!	#NULL!
0	#NULL!	#NULL!	#NULL!	#NULL!	#NULL!
0	#NULL!	#NULL!	#NULL!	#NULL!	#NULL!
0	#NULL!	#NULL!	#NULL!	#NULL!	#NULL!
0	#NULL!	#NULL!	#NULL!	#NULL!	#NULL!
0	#NULL!	#NULL!	#NULL!	#NULL!	#NULL!
0	#NULL!	#NULL!	#NULL!	#NULL!	#NULL!
0	#NULL!	#NULL!	#NULL!	#NULL!	#NULL!

0			#NULL!	#NULL!	#NULL!	#NULL!	#NULL!	
0			#NULL!	#NULL!	#NULL!	#NULL!	#NULL!	
0	3. injectabl	Norgynon	2	1	4	#NULL!	3	They are m
0			#NULL!	#NULL!	#NULL!	#NULL!	#NULL!	
0			#NULL!	#NULL!	#NULL!	#NULL!	#NULL!	
0			#NULL!	#NULL!	#NULL!	#NULL!	#NULL!	
0			#NULL!	#NULL!	#NULL!	#NULL!	#NULL!	
0			#NULL!	#NULL!	#NULL!	#NULL!	#NULL!	
0			#NULL!	#NULL!	#NULL!	#NULL!	#NULL!	
0			#NULL!	#NULL!	#NULL!	#NULL!	#NULL!	
0			#NULL!	#NULL!	#NULL!	#NULL!	#NULL!	
0			#NULL!	#NULL!	#NULL!	#NULL!	#NULL!	
0			#NULL!	#NULL!	#NULL!	#NULL!	#NULL!	
0	1. Jadelle	Injectable	3	1	#NULL!	2	#NULL!	Most client
0			#NULL!	#NULL!	#NULL!	#NULL!	#NULL!	
0			#NULL!	#NULL!	#NULL!	#NULL!	#NULL!	
0			#NULL!	#NULL!	#NULL!	#NULL!	#NULL!	
0			#NULL!	#NULL!	#NULL!	#NULL!	#NULL!	
0			#NULL!	#NULL!	#NULL!	#NULL!	#NULL!	
0			#NULL!	#NULL!	#NULL!	#NULL!	#NULL!	
0			#NULL!	#NULL!	#NULL!	#NULL!	#NULL!	
0			#NULL!	#NULL!	#NULL!	#NULL!	#NULL!	
0			#NULL!	#NULL!	#NULL!	#NULL!	#NULL!	
0			#NULL!	#NULL!	#NULL!	#NULL!	#NULL!	
0			#NULL!	#NULL!	#NULL!	#NULL!	#NULL!	
0			#NULL!	#NULL!	#NULL!	#NULL!	#NULL!	
0			#NULL!	#NULL!	#NULL!	#NULL!	#NULL!	
0			#NULL!	#NULL!	#NULL!	#NULL!	#NULL!	
0			#NULL!	#NULL!	#NULL!	#NULL!	#NULL!	
0			#NULL!	#NULL!	#NULL!	#NULL!	#NULL!	
0			#NULL!	#NULL!	#NULL!	#NULL!	#NULL!	
0			#NULL!	#NULL!	#NULL!	#NULL!	#NULL!	
0			#NULL!	#NULL!	#NULL!	#NULL!	#NULL!	
0			#NULL!	#NULL!	#NULL!	#NULL!	#NULL!	
0			#NULL!	#NULL!	#NULL!	#NULL!	#NULL!	
0			#NULL!	#NULL!	#NULL!	#NULL!	#NULL!	
0			#NULL!	#NULL!	#NULL!	#NULL!	#NULL!	
0			#NULL!	#NULL!	#NULL!	#NULL!	#NULL!	
0			#NULL!	#NULL!	#NULL!	#NULL!	#NULL!	
0			#NULL!	#NULL!	#NULL!	#NULL!	#NULL!	
0			#NULL!	#NULL!	#NULL!	#NULL!	#NULL!	
0			#NULL!	#NULL!	#NULL!	#NULL!	#NULL!	
0			#NULL!	#NULL!	#NULL!	#NULL!	#NULL!	
0			#NULL!	#NULL!	#NULL!	#NULL!	#NULL!	
0			#NULL!	#NULL!	#NULL!	#NULL!	#NULL!	
0			#NULL!	#NULL!	#NULL!	#NULL!	#NULL!	
0			#NULL!	#NULL!	#NULL!	#NULL!	#NULL!	

0			#NULL!	#NULL!	#NULL!	#NULL!	#NULL!	
0			#NULL!	#NULL!	#NULL!	#NULL!	#NULL!	
0			#NULL!	#NULL!	#NULL!	#NULL!	#NULL!	
0			#NULL!	#NULL!	#NULL!	#NULL!	#NULL!	
0			#NULL!	#NULL!	#NULL!	#NULL!	#NULL!	
0			#NULL!	#NULL!	#NULL!	#NULL!	#NULL!	
0			#NULL!	#NULL!	#NULL!	#NULL!	#NULL!	
0			#NULL!	#NULL!	#NULL!	#NULL!	#NULL!	
0			#NULL!	#NULL!	#NULL!	#NULL!	#NULL!	
0			#NULL!	#NULL!	#NULL!	#NULL!	#NULL!	
0			#NULL!	#NULL!	#NULL!	#NULL!	#NULL!	
0			#NULL!	#NULL!	#NULL!	#NULL!	#NULL!	
0			#NULL!	#NULL!	#NULL!	#NULL!	#NULL!	
0			#NULL!	#NULL!	#NULL!	#NULL!	#NULL!	
0			#NULL!	#NULL!	#NULL!	#NULL!	#NULL!	
0			#NULL!	#NULL!	#NULL!	#NULL!	#NULL!	
0			#NULL!	#NULL!	#NULL!	#NULL!	#NULL!	
0			#NULL!	#NULL!	#NULL!	#NULL!	#NULL!	
0			#NULL!	#NULL!	#NULL!	#NULL!	#NULL!	
0			#NULL!	#NULL!	#NULL!	#NULL!	#NULL!	
0			#NULL!	#NULL!	#NULL!	#NULL!	#NULL!	
0			#NULL!	#NULL!	#NULL!	#NULL!	#NULL!	
0			#NULL!	#NULL!	#NULL!	#NULL!	#NULL!	
0			#NULL!	#NULL!	#NULL!	#NULL!	#NULL!	
0			#NULL!	#NULL!	#NULL!	#NULL!	#NULL!	
0			#NULL!	#NULL!	#NULL!	#NULL!	#NULL!	
0			#NULL!	#NULL!	#NULL!	#NULL!	#NULL!	
0			#NULL!	#NULL!	#NULL!	#NULL!	#NULL!	
0			#NULL!	#NULL!	#NULL!	#NULL!	#NULL!	
0			#NULL!	#NULL!	#NULL!	#NULL!	#NULL!	
0			#NULL!	#NULL!	#NULL!	#NULL!	#NULL!	
0			#NULL!	#NULL!	#NULL!	#NULL!	#NULL!	
0			#NULL!	#NULL!	#NULL!	#NULL!	#NULL!	
0			#NULL!	#NULL!	#NULL!	#NULL!	#NULL!	
0			#NULL!	#NULL!	#NULL!	#NULL!	#NULL!	
0			#NULL!	#NULL!	#NULL!	#NULL!	#NULL!	
0			#NULL!	#NULL!	#NULL!	#NULL!	#NULL!	
0			#NULL!	#NULL!	#NULL!	#NULL!	#NULL!	
0			#NULL!	#NULL!	#NULL!	#NULL!	#NULL!	
0			#NULL!	#NULL!	#NULL!	#NULL!	#NULL!	
0	1. Jadelle	condom	3	1	#NULL!	2	#NULL!	These ones
0			#NULL!	#NULL!	#NULL!	#NULL!	#NULL!	
0			#NULL!	#NULL!	#NULL!	#NULL!	#NULL!	
0			#NULL!	#NULL!	#NULL!	#NULL!	#NULL!	
0			#NULL!	#NULL!	#NULL!	#NULL!	#NULL!	
0			#NULL!	#NULL!	#NULL!	#NULL!	#NULL!	
0			#NULL!	#NULL!	#NULL!	#NULL!	#NULL!	

0	#NULL!	#NULL!	#NULL!	#NULL!	#NULL!
0	#NULL!	#NULL!	#NULL!	#NULL!	#NULL!
0	#NULL!	#NULL!	#NULL!	#NULL!	#NULL!
0	#NULL!	#NULL!	#NULL!	#NULL!	#NULL!
0	#NULL!	#NULL!	#NULL!	#NULL!	#NULL!
0	#NULL!	#NULL!	#NULL!	#NULL!	#NULL!
0	#NULL!	#NULL!	#NULL!	#NULL!	#NULL!
0	#NULL!	#NULL!	#NULL!	#NULL!	#NULL!
0	#NULL!	#NULL!	#NULL!	#NULL!	#NULL!
0	#NULL!	#NULL!	#NULL!	#NULL!	#NULL!
0	#NULL!	#NULL!	#NULL!	#NULL!	#NULL!
0	#NULL!	#NULL!	#NULL!	#NULL!	#NULL!
0	#NULL!	#NULL!	#NULL!	#NULL!	#NULL!
0	#NULL!	#NULL!	#NULL!	#NULL!	#NULL!
0	#NULL!	#NULL!	#NULL!	#NULL!	#NULL!
0	#NULL!	#NULL!	#NULL!	#NULL!	#NULL!
0	#NULL!	#NULL!	#NULL!	#NULL!	#NULL!
0	#NULL!	#NULL!	#NULL!	#NULL!	#NULL!
0	#NULL!	#NULL!	#NULL!	#NULL!	#NULL!
0	#NULL!	#NULL!	#NULL!	#NULL!	#NULL!
0	#NULL!	#NULL!	#NULL!	#NULL!	#NULL!
0	#NULL!	#NULL!	#NULL!	#NULL!	#NULL!
0	#NULL!	#NULL!	#NULL!	#NULL!	#NULL!
0	#NULL!	#NULL!	#NULL!	#NULL!	#NULL!
0	#NULL!	#NULL!	#NULL!	#NULL!	#NULL!
0	#NULL!	#NULL!	#NULL!	#NULL!	#NULL!
0	#NULL!	#NULL!	#NULL!	#NULL!	#NULL!
0	#NULL!	#NULL!	#NULL!	#NULL!	#NULL!
0	#NULL!	#NULL!	#NULL!	#NULL!	#NULL!
0	#NULL!	#NULL!	#NULL!	#NULL!	#NULL!
0	#NULL!	#NULL!	#NULL!	#NULL!	#NULL!
0	#NULL!	#NULL!	#NULL!	#NULL!	#NULL!
0	#NULL!	#NULL!	#NULL!	#NULL!	#NULL!
0	#NULL!	#NULL!	#NULL!	#NULL!	#NULL!
0	#NULL!	#NULL!	#NULL!	#NULL!	#NULL!
0	#NULL!	#NULL!	#NULL!	#NULL!	#NULL!
0	#NULL!	#NULL!	#NULL!	#NULL!	#NULL!
0	#NULL!	#NULL!	#NULL!	#NULL!	#NULL!
0	#NULL!	#NULL!	#NULL!	#NULL!	#NULL!
0	#NULL!	#NULL!	#NULL!	#NULL!	#NULL!
0	#NULL!	#NULL!	#NULL!	#NULL!	#NULL!
0	#NULL!	#NULL!	#NULL!	#NULL!	#NULL!
0	#NULL!	#NULL!	#NULL!	#NULL!	#NULL!
0	#NULL!	#NULL!	#NULL!	#NULL!	#NULL!
0	#NULL!	#NULL!	#NULL!	#NULL!	#NULL!
0	#NULL!	#NULL!	#NULL!	#NULL!	#NULL!
0	#NULL!	#NULL!	#NULL!	#NULL!	#NULL!

0	#NULL!	#NULL!	#NULL!	#NULL!	#NULL!
0	#NULL!	#NULL!	#NULL!	#NULL!	#NULL!
0	#NULL!	#NULL!	#NULL!	#NULL!	#NULL!
0	#NULL!	#NULL!	#NULL!	#NULL!	#NULL!
0	#NULL!	#NULL!	#NULL!	#NULL!	#NULL!
0	#NULL!	#NULL!	#NULL!	#NULL!	#NULL!
0	#NULL!	#NULL!	#NULL!	#NULL!	#NULL!
0	#NULL!	#NULL!	#NULL!	#NULL!	#NULL!
0	#NULL!	#NULL!	#NULL!	#NULL!	#NULL!
0	#NULL!	#NULL!	#NULL!	#NULL!	#NULL!
0	#NULL!	#NULL!	#NULL!	#NULL!	#NULL!
0	#NULL!	#NULL!	#NULL!	#NULL!	#NULL!
0	#NULL!	#NULL!	#NULL!	#NULL!	#NULL!
0	#NULL!	#NULL!	#NULL!	#NULL!	#NULL!
0	#NULL!	#NULL!	#NULL!	#NULL!	#NULL!
0	#NULL!	#NULL!	#NULL!	#NULL!	#NULL!
0	#NULL!	#NULL!	#NULL!	#NULL!	#NULL!
0	#NULL!	#NULL!	#NULL!	#NULL!	#NULL!
0	#NULL!	#NULL!	#NULL!	#NULL!	#NULL!
0	#NULL!	#NULL!	#NULL!	#NULL!	#NULL!
0	#NULL!	#NULL!	#NULL!	#NULL!	#NULL!
0	#NULL!	#NULL!	#NULL!	#NULL!	#NULL!
0	#NULL!	#NULL!	#NULL!	#NULL!	#NULL!
0	#NULL!	#NULL!	#NULL!	#NULL!	#NULL!
0	#NULL!	#NULL!	#NULL!	#NULL!	#NULL!
0	#NULL!	#NULL!	#NULL!	#NULL!	#NULL!
0	#NULL!	#NULL!	#NULL!	#NULL!	#NULL!
0	#NULL!	#NULL!	#NULL!	#NULL!	#NULL!
0	#NULL!	#NULL!	#NULL!	#NULL!	#NULL!
0	#NULL!	#NULL!	#NULL!	#NULL!	#NULL!
0	#NULL!	#NULL!	#NULL!	#NULL!	#NULL!
0	#NULL!	#NULL!	#NULL!	#NULL!	#NULL!
0	#NULL!	#NULL!	#NULL!	#NULL!	#NULL!
0	#NULL!	#NULL!	#NULL!	#NULL!	#NULL!
0	#NULL!	#NULL!	#NULL!	#NULL!	#NULL!
0	#NULL!	#NULL!	#NULL!	#NULL!	#NULL!
0	#NULL!	#NULL!	#NULL!	#NULL!	#NULL!
0	#NULL!	#NULL!	#NULL!	#NULL!	#NULL!
0	#NULL!	#NULL!	#NULL!	#NULL!	#NULL!
0	#NULL!	#NULL!	#NULL!	#NULL!	#NULL!
0	#NULL!	#NULL!	#NULL!	#NULL!	#NULL!
0	#NULL!	#NULL!	#NULL!	#NULL!	#NULL!
0	#NULL!	#NULL!	#NULL!	#NULL!	#NULL!
0	#NULL!	#NULL!	#NULL!	#NULL!	#NULL!
0	#NULL!	#NULL!	#NULL!	#NULL!	#NULL!
0	#NULL!	#NULL!	#NULL!	#NULL!	#NULL!
0	#NULL!	#NULL!	#NULL!	#NULL!	#NULL!

0	#NULL!	#NULL!	#NULL!	#NULL!	#NULL!
0	#NULL!	#NULL!	#NULL!	#NULL!	#NULL!
0	#NULL!	#NULL!	#NULL!	#NULL!	#NULL!
0	#NULL!	#NULL!	#NULL!	#NULL!	#NULL!
0	#NULL!	#NULL!	#NULL!	#NULL!	#NULL!
0	#NULL!	#NULL!	#NULL!	#NULL!	#NULL!
0	#NULL!	#NULL!	#NULL!	#NULL!	#NULL!
0	#NULL!	#NULL!	#NULL!	#NULL!	#NULL!
0	#NULL!	#NULL!	#NULL!	#NULL!	#NULL!
0	#NULL!	#NULL!	#NULL!	#NULL!	#NULL!
0	#NULL!	#NULL!	#NULL!	#NULL!	#NULL!
0	#NULL!	#NULL!	#NULL!	#NULL!	#NULL!
0	#NULL!	#NULL!	#NULL!	#NULL!	#NULL!
0	#NULL!	#NULL!	#NULL!	#NULL!	#NULL!
0	#NULL!	#NULL!	#NULL!	#NULL!	#NULL!
0	#NULL!	#NULL!	#NULL!	#NULL!	#NULL!
0	#NULL!	#NULL!	#NULL!	#NULL!	#NULL!
0	#NULL!	#NULL!	#NULL!	#NULL!	#NULL!
0	#NULL!	#NULL!	#NULL!	#NULL!	#NULL!
0	#NULL!	#NULL!	#NULL!	#NULL!	#NULL!
0	#NULL!	#NULL!	#NULL!	#NULL!	#NULL!
0	#NULL!	#NULL!	#NULL!	#NULL!	#NULL!
0	#NULL!	#NULL!	#NULL!	#NULL!	#NULL!
0	#NULL!	#NULL!	#NULL!	#NULL!	#NULL!
0	#NULL!	#NULL!	#NULL!	#NULL!	#NULL!
0	#NULL!	#NULL!	#NULL!	#NULL!	#NULL!
0	#NULL!	#NULL!	#NULL!	#NULL!	#NULL!
0	#NULL!	#NULL!	#NULL!	#NULL!	#NULL!
0	#NULL!	#NULL!	#NULL!	#NULL!	#NULL!
0	#NULL!	#NULL!	#NULL!	#NULL!	#NULL!
0	#NULL!	#NULL!	#NULL!	#NULL!	#NULL!
0	#NULL!	#NULL!	#NULL!	#NULL!	#NULL!
0	#NULL!	#NULL!	#NULL!	#NULL!	#NULL!
0	#NULL!	#NULL!	#NULL!	#NULL!	#NULL!
0	#NULL!	#NULL!	#NULL!	#NULL!	#NULL!
0	#NULL!	#NULL!	#NULL!	#NULL!	#NULL!
0	#NULL!	#NULL!	#NULL!	#NULL!	#NULL!
0	#NULL!	#NULL!	#NULL!	#NULL!	#NULL!
0	#NULL!	#NULL!	#NULL!	#NULL!	#NULL!
0	#NULL!	#NULL!	#NULL!	#NULL!	#NULL!
0	#NULL!	#NULL!	#NULL!	#NULL!	#NULL!
0	#NULL!	#NULL!	#NULL!	#NULL!	#NULL!
0	#NULL!	#NULL!	#NULL!	#NULL!	#NULL!
0	#NULL!	#NULL!	#NULL!	#NULL!	#NULL!
0	#NULL!	#NULL!	#NULL!	#NULL!	#NULL!
0	#NULL!	#NULL!	#NULL!	#NULL!	#NULL!
0	#NULL!	#NULL!	#NULL!	#NULL!	#NULL!

0	#NULL!	#NULL!	#NULL!	#NULL!	#NULL!
0	#NULL!	#NULL!	#NULL!	#NULL!	#NULL!
0	#NULL!	#NULL!	#NULL!	#NULL!	#NULL!
0	#NULL!	#NULL!	#NULL!	#NULL!	#NULL!
0	#NULL!	#NULL!	#NULL!	#NULL!	#NULL!
0	#NULL!	#NULL!	#NULL!	#NULL!	#NULL!
0	#NULL!	#NULL!	#NULL!	#NULL!	#NULL!
0	#NULL!	#NULL!	#NULL!	#NULL!	#NULL!
0	#NULL!	#NULL!	#NULL!	#NULL!	#NULL!
0	#NULL!	#NULL!	#NULL!	#NULL!	#NULL!
0	#NULL!	#NULL!	#NULL!	#NULL!	#NULL!
0	#NULL!	#NULL!	#NULL!	#NULL!	#NULL!
0	#NULL!	#NULL!	#NULL!	#NULL!	#NULL!
0	#NULL!	#NULL!	#NULL!	#NULL!	#NULL!

J10Doyous	J11Howoft	J12Howlon	J13Ifyesiny	J14Whatro	J15Inyourv	K1Haveyou	K2Yesterda	K3Howmar
1. Yes	1	3. one mor	doed not m	Does not e	Supply or a	1	1	2
	#NULL!					1	1	3
2. No	#NULL!	2. 1-2 weeks				1	1	1
	#NULL!					1	1	2
1. Yes	4		health wor	late reporti	early repor	2	2	#NULL!
	#NULL!					1	1	2
	#NULL!					1	1	1
2. No	#NULL!					1	2	2
	#NULL!					2	2	#NULL!
is according	#NULL!					1	1	3
	#NULL!					2	2	#NULL!
	#NULL!					2	2	#NULL!
1. Yes	3	4. More th	delay in rep	do not act	notify all re	2	2	#NULL!
	#NULL!					1	1	1
	#NULL!					2	2	#NULL!
	#NULL!					2	2	#NULL!
	#NULL!					1	1	2
	#NULL!					1	1	2
	#NULL!					2	2	#NULL!
	#NULL!					2	2	#NULL!
	#NULL!					2	2	#NULL!
	#NULL!					1	1	4
	#NULL!					1	1	4
	#NULL!					1	1	3
	#NULL!					1	1	1
	#NULL!					1	1	2
	#NULL!					2	2	#NULL!
	#NULL!					1	1	3
	#NULL!					1	1	4
	#NULL!					1	1	3
	#NULL!					1	1	3
	#NULL!					1	1	3
	#NULL!					1	1	2
	#NULL!					1	1	3
	#NULL!					1	1	3
	#NULL!					1	1	2
	#NULL!					1	1	3
	#NULL!					1	1	2
	#NULL!					1	1	2
	#NULL!					1	1	4
	#NULL!					1	1	1
	#NULL!					1	1	3
	#NULL!					1	1	3
	#NULL!					1	1	3
	#NULL!					1	1	3
	#NULL!					1	1	2

	#NULL!				1	1	3
	#NULL!				1	1	2
	#NULL!				2	2	#NULL!
	#NULL!				1	1	3
	#NULL!				1	1	1
	#NULL!				2	2	#NULL!
	#NULL!				1	1	3
	#NULL!				1	1	3
	#NULL!				1	1	3
	#NULL!				1	1	3
	#NULL!				1	1	3
	#NULL!				1	1	1
	#NULL!				2	2	#NULL!
	#NULL!				1	1	3
	#NULL!				1	1	3
	#NULL!				1	1	3
	#NULL!				2	2	#NULL!
	#NULL!				1	1	3
	#NULL!				1	1	3
	#NULL!				1	1	4
1. Yes	2	4. More than 1 month	they dont s	the contrac	2	2	#NULL!
	#NULL!				1	1	2
	#NULL!				1	1	3
	#NULL!				1	1	4
	#NULL!				1	1	3
	#NULL!				1	1	3
	#NULL!				1	1	1
	#NULL!				1	1	2
	#NULL!				2	2	#NULL!
	#NULL!				1	1	2
	#NULL!				1	2	4
	#NULL!				1	1	2
	#NULL!				1	1	3
	#NULL!				1	1	3
	#NULL!				1	1	4
	#NULL!				1	1	3
	#NULL!				1	1	4
	#NULL!				1	1	3
	#NULL!				1	1	2
	#NULL!				1	1	2
	#NULL!				1	1	3
	#NULL!				1	1	1
	#NULL!				2	2	#NULL!
	#NULL!				1	1	1
	#NULL!				1	1	1
	#NULL!				1	1	3
	#NULL!				1	1	3

#NULL!	1	1	3
#NULL!	1	1	2
#NULL!	2	2	#NULL!
#NULL!	2	2	#NULL!
#NULL!	1	1	4
#NULL!	1	2	3
#NULL!	1	1	3
#NULL!	2	1	#NULL!
#NULL!	1	1	3
#NULL!	1	1	4
#NULL!	1	1	2
#NULL!	1	1	4
#NULL!	1	1	3
#NULL!	1	1	3
#NULL!	1	1	4
#NULL!	1	2	3
#NULL!	2	2	#NULL!
#NULL!	1	2	3
#NULL!	1	1	3
#NULL!	1	2	3
#NULL!	1	1	3
#NULL!	1	1	3
#NULL!	1	2	3
#NULL!	1	1	1
#NULL!	1	1	4
#NULL!	2	2	#NULL!
#NULL!	2	2	#NULL!
#NULL!	1	1	3
#NULL!	1	2	1
#NULL!	1	1	3
#NULL!	1	1	1
#NULL!	2	2	#NULL!
#NULL!	2	2	#NULL!
#NULL!	2	2	#NULL!
#NULL!	1	1	3
#NULL!	1	1	2
#NULL!	2	2	#NULL!
#NULL!	1	1	3
#NULL!	1	1	4
#NULL!	1	1	3
#NULL!	1	1	3
#NULL!	1	1	3
#NULL!	2	2	#NULL!
#NULL!	1	1	2
#NULL!	1	1	2
#NULL!	1	1	3
#NULL!	1	2	4

#NULL!	1	2	4
#NULL!	1	1	4
#NULL!	1	1	4
#NULL!	2	2	#NULL!
#NULL!	2	2	#NULL!
#NULL!	1	1	3
#NULL!	2	2	#NULL!
#NULL!	1	1	2
#NULL!	1	1	1
#NULL!	1	1	2
#NULL!	1	2	2
#NULL!	2	2	#NULL!
#NULL!	1	1	3
#NULL!	1	1	3
#NULL!	1	1	3
#NULL!	1	1	3
#NULL!	1	1	2
#NULL!	1	1	4
#NULL!	1	1	4
#NULL!	1	1	1
#NULL!	1	1	4
#NULL!	1	2	4
#NULL!	1	1	3
#NULL!	1	1	3
#NULL!	1	1	3
#NULL!	1	1	2
#NULL!	1	1	3
#NULL!	1	1	3
#NULL!	1	1	2
#NULL!	1	1	2
#NULL!	1	1	3
#NULL!	1	2	3
#NULL!	1	1	3
#NULL!	1	1	3
#NULL!	1	1	3
#NULL!	2	2	#NULL!
#NULL!	1	2	3
#NULL!	1	1	3
#NULL!	1	1	4
#NULL!	1	1	3
#NULL!	1	1	3
#NULL!	1	1	2
#NULL!	1	1	2
#NULL!	1	1	2
#NULL!	1	1	2
#NULL!	1	1	3
#NULL!	1	1	4

	#NULL!		1	1	3
	#NULL!		1	1	4
	#NULL!		1	1	3
	#NULL!		1	1	3
	#NULL!		1	1	2
	#NULL!		1	1	4
	#NULL!		1	1	3
	#NULL!		1	1	3
	#NULL!		1	2	4
	#NULL!		1	1	3
	#NULL!		1	1	3
	#NULL!		1	1	3
	#NULL!		1	1	3
	#NULL!		1	1	3
	#NULL!		1	1	3
	#NULL!		1	1	4
	#NULL!		1	1	3
	#NULL!		2	2	#NULL!
	#NULL!		2	2	#NULL!
	#NULL!		1	1	3
	#NULL!		1	1	3
	#NULL!		1	1	3
	#NULL!		1	1	3
	#NULL!		1	2	3
	#NULL!		1	1	3
	#NULL!		1	1	2
	#NULL!		2	2	#NULL!
	#NULL!		2	2	#NULL!
	#NULL!		1	1	4
	#NULL!		1	1	3
	#NULL!		1	1	3
	#NULL!		2	2	#NULL!
	#NULL!		2	2	#NULL!
	#NULL!		1	2	3
	#NULL!		1	1	3
	#NULL!		2	2	#NULL!
2. No	#NULL!	1. Less than a week	2	2	#NULL!
	#NULL!		1	1	3
	#NULL!		1	1	3
	#NULL!		1	1	3
	#NULL!		1	1	3
	#NULL!		1	1	4
	#NULL!		1	1	4
	#NULL!		1	1	3
	#NULL!		1	1	3
	#NULL!		2	2	#NULL!
	#NULL!		1	2	3

#NULL!	2	2	#NULL!
#NULL!	2	2	#NULL!
#NULL!	1	2	1
#NULL!	1	1	3
#NULL!	2	2	#NULL!
#NULL!	2	2	#NULL!
#NULL!	1	1	3
#NULL!	1	1	3
#NULL!	1	1	4
#NULL!	1	1	4
#NULL!	1	1	2
#NULL!	1	1	3
#NULL!	2	2	#NULL!
#NULL!	1	1	2
#NULL!	1	1	3
#NULL!	2	2	#NULL!
#NULL!	1	1	3
#NULL!	1	1	3
#NULL!	1	1	3
#NULL!	1	1	4
#NULL!	1	1	3
#NULL!	1	1	2
#NULL!	1	1	3
#NULL!	1	1	4
#NULL!	1	1	3
#NULL!	1	1	3
#NULL!	1	1	3
#NULL!	1	1	3
#NULL!	2	2	#NULL!
#NULL!	1	1	3
#NULL!	1	1	2
#NULL!	1	1	1
#NULL!	2	2	#NULL!
#NULL!	1	1	3
#NULL!	1	1	3
#NULL!	1	1	3
#NULL!	1	1	3
#NULL!	1	1	4
#NULL!	2	2	#NULL!
#NULL!	1	1	3
#NULL!	1	1	4
#NULL!	2	2	#NULL!
#NULL!	1	1	3
#NULL!	1	2	3
#NULL!	1	1	3
#NULL!	1	1	3
#NULL!	1	1	2

	#NULL!					2	2	#NULL!
	#NULL!					1	2	3
1. Yes	#NULL!		The shorta	Goverment	Free supply	2	2	#NULL!
	#NULL!					1	1	3
	#NULL!					1	1	2
	#NULL!					1	1	3
	#NULL!					1	1	3
	#NULL!					2	2	#NULL!
	#NULL!					1	1	4
	#NULL!					2	2	#NULL!
	#NULL!					1	1	4
	#NULL!					1	1	3
	#NULL!					1	1	4
1. Yes	#NULL!	3. one mor	Inadequate	Delay in su	Requstion	1	1	3
	#NULL!					1	1	2
	#NULL!					1	1	3
	#NULL!					1	1	3
	#NULL!					2	2	#NULL!
	#NULL!					2	2	#NULL!
	#NULL!					1	1	2
	#NULL!					1	1	1
	#NULL!					1	1	3
	#NULL!					1	1	3
	#NULL!					1	2	4
	#NULL!					2	2	#NULL!
	#NULL!					1	2	4
	#NULL!					1	1	3
	#NULL!					1	1	3
	#NULL!					2	2	#NULL!
	#NULL!					1	1	4
	#NULL!					2	2	#NULL!
	#NULL!					1	2	4
	#NULL!					1	1	4
	#NULL!					1	1	4
	#NULL!					1	1	3
	#NULL!					1	1	2
	#NULL!					1	1	3
	#NULL!					1	1	3
	#NULL!					1	1	3
	#NULL!					1	1	3
	#NULL!					1	1	2
	#NULL!					2	2	#NULL!
	#NULL!					1	1	1
	#NULL!					1	1	4
1. Yes	#NULL!					1	1	3
	#NULL!					2	2	#NULL!
	#NULL!					1	1	3

	#NULL!					1	1	4
	#NULL!					1	2	2
	#NULL!					1	2	2
	#NULL!					1	1	1
	#NULL!					1	1	3
	#NULL!					1	1	2
	#NULL!					1	1	1
	#NULL!					1	2	#NULL!
	#NULL!					1	1	1
	#NULL!					1	1	1
	#NULL!					1	1	2
	#NULL!					2	2	#NULL!
	#NULL!					1	1	3
	#NULL!					1	1	3
	#NULL!					1	1	3
	#NULL!					1	1	3
	#NULL!					2	2	#NULL!
	#NULL!					1	2	4
	#NULL!					1	1	4
	#NULL!					1	1	3
	#NULL!					1	1	3
	#NULL!					1	1	4
	#NULL!					1	1	4
	#NULL!					2	2	#NULL!
	#NULL!					1	2	4
	#NULL!					1	1	3
	#NULL!					2	2	#NULL!
	#NULL!					2	2	#NULL!
	#NULL!					2	2	#NULL!
	#NULL!					1	2	4
	#NULL!					1	1	4
	#NULL!					1	1	4
	#NULL!					1	1	3
	#NULL!					2	2	#NULL!
	#NULL!					2	2	#NULL!
	#NULL!					1	1	4
	#NULL!					2	2	#NULL!
	#NULL!					2	2	#NULL!
	#NULL!					1	2	4
	#NULL!					2	2	#NULL!
1. Yes	1	3. one mor	Mostly use	can not exp	Timely pay	1	1	3
	#NULL!					1	1	3
	#NULL!					1	1	2
	#NULL!					1	1	3
	#NULL!					2	2	#NULL!
	#NULL!					1	1	4
	#NULL!					1	1	4

#NULL!	1	1	3
#NULL!	1	1	4
#NULL!	1	1	3
#NULL!	1	1	3
#NULL!	2	2	#NULL!
#NULL!	1	1	4
#NULL!	1	1	3
#NULL!	1	1	4
#NULL!	1	1	3
#NULL!	2	2	#NULL!
#NULL!	1	2	1
#NULL!	2	2	#NULL!
#NULL!	1	1	3
#NULL!	1	1	3
#NULL!	2	2	#NULL!
#NULL!	1	1	3
#NULL!	1	1	3
#NULL!	1	1	3
#NULL!	1	1	3
#NULL!	2	2	#NULL!
#NULL!	1	1	3
#NULL!	1	1	3
#NULL!	1	2	3
#NULL!	2	2	#NULL!
#NULL!	1	1	3
#NULL!	1	2	3
#NULL!	1	1	3
#NULL!	2	2	#NULL!
#NULL!	2	2	#NULL!
#NULL!	1	1	3
#NULL!	1	1	3
#NULL!	1	1	3
#NULL!	2	2	#NULL!
#NULL!	1	2	3
#NULL!	1	2	1
#NULL!	2	2	#NULL!
#NULL!	1	1	2
#NULL!	2	2	#NULL!
#NULL!	2	2	#NULL!
#NULL!	2	2	#NULL!
#NULL!	1	1	3
#NULL!	1	1	3
#NULL!	1	1	3
#NULL!	1	1	3
#NULL!	1	1	4
#NULL!	1	2	3
#NULL!	1	1	3

#NULL!	1	2	3
#NULL!	1	1	3
#NULL!	1	1	2
#NULL!	1	1	3
#NULL!	1	1	3
#NULL!	2	2	#NULL!
#NULL!	1	1	3
#NULL!	1	1	4
#NULL!	1	1	3
#NULL!	1	1	3
#NULL!	1	1	4
#NULL!	1	1	3
#NULL!	1	1	3
#NULL!	1	1	3
#NULL!	2	2	#NULL!
#NULL!	1	1	4
#NULL!	2	2	#NULL!
#NULL!	1	1	3
#NULL!	2	2	#NULL!
#NULL!	2	2	#NULL!
#NULL!	1	1	3
#NULL!	1	1	4
#NULL!	1	1	3
#NULL!	2	2	#NULL!
#NULL!	2	2	#NULL!
#NULL!	2	2	#NULL!
#NULL!	1	1	3
#NULL!	2	2	#NULL!
#NULL!	1	1	3
#NULL!	2	2	#NULL!
#NULL!	1	1	3
#NULL!	1	1	3
#NULL!	1	1	3
#NULL!	1	1	3
#NULL!	1	1	3
#NULL!	1	1	3
#NULL!	1	1	4
#NULL!	1	1	2
#NULL!	2	2	#NULL!
#NULL!	1	1	2
#NULL!	1	1	2
#NULL!	1	2	3
#NULL!	1	1	3
#NULL!	2	2	#NULL!
#NULL!	2	2	#NULL!
#NULL!	1	2	3
#NULL!	1	1	3

#NULL!	1	1	3
#NULL!	1	1	3
#NULL!	1	1	3
#NULL!	1	1	3
#NULL!	1	1	3
#NULL!	1	1	2
#NULL!	1	1	2
#NULL!	1	1	3
#NULL!	1	1	3
#NULL!	1	1	3
#NULL!	1	1	4
#NULL!	1	1	4
#NULL!	1	1	3
#NULL!	2	2	#NULL!
#NULL!	2	2	#NULL!
#NULL!	1	1	2
#NULL!	1	1	2
#NULL!	2	2	#NULL!
#NULL!	1	1	1
#NULL!	1	1	3
#NULL!	2	2	#NULL!
#NULL!	1	1	2
#NULL!	1	1	3
#NULL!	1	1	2
#NULL!	1	1	3
#NULL!	1	1	3
#NULL!	1	1	3
#NULL!	1	1	3
#NULL!	1	1	3
#NULL!	1	1	2
#NULL!	1	1	4
#NULL!	1	1	3
#NULL!	1	1	4
#NULL!	1	1	4
#NULL!	2	2	#NULL!
#NULL!	2	2	#NULL!
#NULL!	2	2	#NULL!
#NULL!	1	1	2
#NULL!	1	1	3
#NULL!	2	2	#NULL!
#NULL!	2	2	#NULL!
#NULL!	2	2	#NULL!
#NULL!	2	2	#NULL!
#NULL!	1	1	3
#NULL!	2	2	#NULL!
#NULL!	2	2	#NULL!
#NULL!	1	1	3

#NULL!	2	2	#NULL!
#NULL!	2	2	#NULL!
#NULL!	2	2	#NULL!
#NULL!	2	2	#NULL!
#NULL!	1	1	3
#NULL!	1	1	3
#NULL!	1	1	3
#NULL!	1	1	3
#NULL!	2	2	#NULL!
#NULL!	1	1	1
#NULL!	2	2	#NULL!
#NULL!	2	2	#NULL!
#NULL!	2	2	#NULL!
#NULL!	2	2	#NULL!

Grainsroots	Dairyprodu	Fleshfoods	Eggs	Legumes	VitaminAri	Otherfruits	L1TYPEofH	L2TOILETfa
1	0	1	0	1	0	0	2	5
1	0	1	0	1	0	0	3	5
1	0	1	0	1	0	0	2	5
1	0	0	0	0	0	0	3	5
0	0	0	0	0	0	0	2	5
1	0	1	0	1	1	0	1	4
1	0	1	0	0	0	0	1	1
1	0	0	0	0	0	0	1	1
0	0	0	0	0	0	0	3	5
1	1	0	1	1	0	0	2	5
0	0	0	0	0	0	0	3	5
0	0	0	0	0	0	0	2	5
0	0	0	0	0	0	0	2	5
1	0	0	0	0	0	0	1	5
0	0	0	0	0	0	0	3	5
0	0	0	0	0	0	0	3	5
0	0	1	1	0	1	1	3	5
1	0	1	0	0	0	1	1	5
0	0	0	0	0	0	0	2	5
0	0	0	0	0	0	0	1	4
0	0	0	0	0	0	0	2	5
1	1	1	0	1	1	0	2	5
1	1	1	0	1	1	0	2	5
1	1	1	0	1	1	0	1	1
1	0	1	0	0	0	0	1	5
1	1	0	1	0	0	1	2	5
0	0	0	0	0	0	0	3	3
1	0	1	0	1	1	0	2	5
0	1	1	1	1	0	0	2	5
1	1	1	0	1	1	1	1	5
1	0	0	1	1	1	0	2	5
1	0	1	0	0	1	0	2	5
1	0	0	0	0	1	1	1	5
1	0	1	0	1	1	0	2	5
1	0	1	0	1	1	0	2	5
1	1	1	0	1	0	0	2	5
1	0	0	1	0	1	0	2	5
1	1	0	0	1	0	0	1	5
1	0	1	0	1	1	1	2	5
1	0	1	0	1	1	0	2	5
1	0	1	0	1	1	0	2	5
1	0	1	0	1	1	0	2	5
1	0	1	0	1	1	0	2	5
1	1	1	0	1	1	1	2	5
1	0	1	0	0	1	0	2	5
1	0	1	0	1	1	1	1	5

1	0	1	0	0	1	0	2	5
1	0	0	0	0	0	0	3	5
0	0	0	0	0	0	0	3	5
1	0	0	0	0	0	0	3	5
1	0	0	0	0	1	1	2	5
0	0	0	0	0	0	0	1	3
1	0	1	0	1	1	0	1	5
1	1	0	0	1	1	1	3	3
1	0	0	0	1	0	1	3	5
1	0	1	0	1	0	1	3	5
1	1	1	0	0	0	0	2	1
1	0	0	0	1	0	0	2	5
0	0	0	0	0	0	0	2	5
1	0	1	0	1	1	1	1	3
1	0	1	0	0	1	1	2	5
1	1	1	0	1	0	0	2	2
0	0	0	0	0	0	0	2	5
1	0	0	0	1	0	0	3	5
1	0	0	0	1	1	0	3	5
1	0	0	1	1	0	1	3	5
0	0	0	0	0	0	0	3	5
1	0	1	1	0	0	1	2	5
1	0	0	0	0	0	0	3	5
1	0	0	0	1	0	0	2	5
1	0	1	0	1	1	1	3	5
1	0	0	0	0	0	0	3	5
1	0	0	0	0	0	0	1	5
1	1	1	0	0	1	0	3	3
0	0	0	0	0	0	0	3	5
1	0	0	0	1	1	0	2	5
1	1	0	0	0	0	0	3	5
1	0	1	0	1	1	1	2	5
0	0	0	0	0	0	0	2	5
1	1	0	0	0	0	1	2	5
1	0	1	0	1	0	1	3	5
1	0	1	0	0	1	0	1	5
1	1	0	1	1	1	0	2	1
1	1	1	0	0	0	0	1	3
1	0	1	0	1	1	0	3	5
1	0	0	0	0	1	0	3	5
1	1	1	0	1	1	0	3	5
1	1	0	0	0	0	0	3	5
0	0	0	0	0	0	0	3	5
1	1	0	0	0	0	0	3	5
1	0	0	0	0	0	0	3	5
1	1	1	0	1	1	1	3	5
1	1	1	1	1	1	1	3	5

1	0	1	0	1	1	1	3	5
1	1	1	1	1	1	1	3	5
0	0	0	0	0	0	0	3	5
0	0	0	0	0	0	0	2	5
1	0	0	0	1	0	0	2	5
1	0	0	0	1	0	0	2	5
1	0	1	0	1	0	1	2	5
1	0	1	0	0	1	0	2	5
1	0	0	0	0	1	0	3	5
1	0	1	0	1	1	1	3	5
1	0	0	0	0	0	0	2	5
0	0	1	0	1	1	1	2	5
1	1	0	0	1	0	0	3	5
1	1	0	0	1	0	0	3	5
1	0	1	0	1	1	0	2	5
0	0	0	0	0	1	0	2	5
0	0	0	0	0	0	0	2	5
0	0	0	0	0	0	1	2	5
1	0	1	0	0	0	0	2	5
1	0	0	0	1	0	0	3	5
1	0	1	0	1	1	0	2	5
1	0	1	0	1	0	0	2	5
1	0	1	0	1	1	0	2	5
1	0	1	0	1	0	1	3	5
1	1	0	0	0	0	0	3	5
0	0	0	0	0	0	0	2	5
0	0	0	0	0	0	0	3	5
1	0	0	0	1	1	0	3	5
1	0	0	0	0	0	0	3	5
1	1	1	0	1	0	0	3	5
1	0	0	0	0	0	0	2	5
0	0	0	0	0	0	0	2	5
0	0	0	0	0	0	0	2	5
0	0	0	0	0	0	0	2	5
1	1	1	0	1	1	1	3	5
1	1	1	0	1	1	1	3	5
0	0	0	0	0	0	0	3	5
1	1	0	0	1	0	1	3	5
1	1	1	0	1	1	1	1	5
1	0	1	0	1	1	1	3	5
1	1	0	0	1	0	0	1	5
1	0	1	0	0	1	1	2	5
0	0	0	0	0	0	0	1	5
1	0	1	0	1	1	0	1	5
1	0	0	0	0	0	0	1	5
1	0	1	0	1	1	1	3	3
1	1	0	0	0	0	0	3	5

1	1	1	0	1	0	1	3	5
1	0	1	0	0	1	1	2	5
1	0	0	0	0	1	1	2	3
0	0	0	0	0	0	0	1	3
0	0	0	0	0	0	0	1	1
1	0	1	0	0	1	1	1	5
0	0	0	0	0	0	0	1	5
1	1	0	0	0	0	0	1	2
1	1	1	0	0	1	0	1	2
0	1	0	0	1	1	0	1	5
1	0	0	0	1	1	0	1	5
0	0	0	0	0	0	0	1	2
1	1	1	1	1	0	1	1	5
1	0	1	0	0	0	0	2	5
1	1	1	0	1	1	0	2	5
1	0	1	0	0	1	0	2	5
1	0	1	0	1	1	1	2	5
1	0	1	0	1	0	1	2	5
1	1	0	0	1	0	1	2	5
0	0	0	0	1	0	0	2	5
1	0	1	0	1	0	1	2	5
1	0	1	0	1	1	1	2	5
1	0	0	0	1	0	0	2	5
1	0	1	0	1	1	1	2	5
1	0	1	0	1	0	1	1	5
1	0	0	0	0	0	0	2	5
1	0	1	0	1	0	1	2	5
1	1	0	0	0	1	0	2	5
1	1	0	0	1	0	1	2	5
1	1	0	0	1	0	0	3	5
1	0	1	0	1	1	0	3	5
1	0	1	1	1	1	0	1	5
1	0	1	0	1	1	0	3	5
1	0	0	0	1	1	0	3	5
1	0	1	0	1	1	0	3	5
0	0	0	0	0	0	0	2	5
1	1	1	0	1	1	0	2	5
1	0	1	0	1	1	0	1	5
1	1	1	1	1	1	1	2	5
1	0	0	0	0	0	0	1	5
1	0	1	0	0	0	0	3	5
1	0	1	0	0	0	1	3	5
1	0	0	0	1	1	1	3	5
1	0	0	0	1	1	0	3	5
1	0	1	0	1	1	1	3	5
1	0	1	0	1	1	0	3	5
1	1	0	1	1	1	0	2	5

1	0	0	0	1	0	0	1	5
1	1	0	0	0	0	0	3	5
1	0	1	0	1	1	1	3	5
1	0	1	0	1	1	0	3	5
1	1	0	0	0	0	0	3	5
1	1	0	0	1	1	1	3	5
1	0	1	1	0	0	0	2	5
1	0	1	0	1	1	0	2	5
1	0	0	0	1	0	0	2	5
1	0	1	0	1	1	1	2	5
1	0	1	0	1	1	1	2	5
1	0	1	0	1	1	0	2	5
1	0	1	0	1	1	1	2	5
1	0	1	0	1	0	1	2	5
1	1	0	0	1	0	0	2	5
1	0	1	0	1	0	0	2	5
1	0	1	0	1	1	1	2	5
0	0	0	0	0	0	0	3	5
0	0	0	0	0	0	0	3	5
1	1	1	1	1	1	1	3	5
1	0	1	0	1	1	0	3	5
1	0	1	0	1	1	0	2	5
1	0	1	0	1	1	1	2	5
1	0	0	0	0	1	1	3	5
1	1	0	0	0	0	0	1	5
1	1	0	0	0	0	0	1	5
0	0	0	0	0	0	0	1	4
0	0	0	0	0	0	0	1	5
1	1	1	1	1	1	1	2	3
1	1	1	0	1	1	1	1	3
1	0	1	0	1	1	0	3	5
0	0	0	0	0	0	0	1	5
0	0	0	0	0	0	0	3	5
1	1	0	0	0	0	1	1	1
1	1	1	1	1	1	1	3	5
0	0	0	0	0	0	0	3	5
0	0	0	0	0	0	0	3	5
1	0	1	0	1	1	0	3	5
1	0	0	0	1	0	0	3	5
1	0	1	0	1	1	1	2	5
1	1	1	0	0	0	0	3	5
1	1	0	1	1	1	1	2	5
1	0	1	0	1	1	0	2	3
1	0	1	0	1	1	0	2	5
1	0	1	0	1	1	0	2	5
0	0	0	0	0	0	0	3	5
1	1	0	0	0	0	0	3	5

0	0	0	0	0	0	0	1	3
0	0	0	0	0	0	0	3	5
1	0	0	0	0	0	0	3	5
1	0	1	0	1	1	1	2	5
0	0	0	0	0	0	0	3	5
0	0	0	0	0	0	0	3	5
1	0	0	0	0	0	0	3	5
1	0	1	0	1	0	0	3	5
1	0	1	0	1	0	1	2	5
1	1	1	1	1	1	1	1	5
1	1	1	0	1	0	0	1	3
1	0	1	0	0	1	0	1	5
0	0	0	0	0	0	0	1	2
1	0	1	0	0	1	0	3	5
1	0	0	0	0	1	0	3	5
0	0	0	0	0	0	0	3	3
1	0	1	0	1	0	1	3	5
1	0	1	0	1	0	0	1	5
1	0	1	0	1	1	1	2	5
1	1	0	0	0	1	1	2	5
1	1	0	0	1	1	0	2	5
1	0	0	0	1	0	0	3	5
1	0	0	0	1	1	1	3	5
1	1	1	0	1	1	1	3	5
1	0	0	0	0	0	0	3	3
1	0	0	0	0	1	1	3	5
1	0	0	0	0	0	0	3	5
1	0	0	0	0	1	1	3	5
0	0	0	0	0	0	0	3	5
1	0	0	0	0	0	0	3	5
1	0	1	1	0	0	1	2	5
1	1	0	0	0	0	0	3	5
0	0	0	0	0	0	0	2	5
1	0	1	0	1	1	1	3	5
1	0	1	0	1	0	0	3	5
1	0	1	0	1	1	0	1	5
0	1	1	0	1	1	0	2	5
1	1	1	1	1	1	1	3	5
0	0	0	0	0	0	0	3	5
1	1	1	0	1	1	0	2	5
1	0	0	1	1	1	0	3	4
0	0	0	0	0	0	0	1	3
1	0	0	0	0	0	0	3	5
1	1	0	1	0	0	0	3	4
1	0	0	0	1	0	1	3	4
1	1	1	0	1	1	0	2	5
1	0	1	0	0	0	0	2	5

0	0	0	0	0	0	0	1	1
1	0	0	0	0	0	0	2	5
0	0	0	0	0	0	0	1	5
1	1	1	0	1	0	0	1	1
1	0	1	0	1	1	0	3	5
1	0	0	0	1	0	1	3	4
1	0	0	0	0	1	1	3	5
0	0	0	0	0	0	0	3	5
1	1	1	0	1	0	1	1	5
0	0	0	0	0	0	0	3	5
1	0	0	0	0	1	1	1	5
1	0	1	0	1	0	1	3	4
1	1	1	0	0	1	0	2	5
1	1	1	0	1	1	1	1	1
1	0	1	0	0	0	0	2	5
1	0	0	1	1	0	1	1	1
1	1	1	0	0	1	0	2	3
0	0	0	0	0	0	0	2	5
0	0	0	0	0	0	0	2	4
1	0	1	0	1	1	0	2	5
1	1	0	0	0	0	0	2	5
1	1	1	0	0	0	1	3	4
1	1	1	0	1	1	1	1	3
1	0	1	0	1	1	0	2	5
0	0	0	0	0	0	0	3	5
1	0	1	0	0	1	0	1	5
1	1	1	0	1	1	1	3	5
1	0	1	1	1	1	1	3	5
0	0	0	0	0	0	0	1	2
1	1	1	1	1	1	0	1	5
0	0	0	0	0	0	0	1	2
1	1	1	0	1	1	1	1	5
1	0	1	0	1	1	0	1	1
1	0	1	0	1	0	0	1	5
1	1	0	0	1	1	0	3	5
1	0	1	0	1	1	1	2	5
1	0	1	0	1	1	1	1	5
1	0	1	0	1	1	0	3	5
1	0	0	0	0	0	0	3	5
1	0	1	0	1	1	0	2	5
1	0	0	0	1	1	0	3	5
0	0	0	0	0	0	0	2	5
1	0	0	0	1	1	0	1	5
1	0	1	0	1	1	0	3	5
1	0	0	0	0	1	1	3	3
0	0	0	0	0	0	0	3	5
1	0	0	0	1	1	0	3	5

1	1	0	0	1	1	0	3	5
1	0	0	0	1	1	0	3	5
1	0	0	0	0	0	0	1	5
1	1	0	0	0	0	1	3	5
1	0	0	1	1	0	1	3	5
1	1	1	1	1	1	0	3	5
1	1	0	0	0	0	0	1	3
1	0	1	0	0	0	1	3	5
1	0	1	0	1	1	1	3	5
1	1	1	0	1	0	0	3	5
1	1	1	0	1	1	1	3	5
0	0	0	0	0	0	0	3	5
1	1	1	1	1	1	1	3	5
1	1	1	1	1	1	1	3	5
1	1	1	1	1	1	1	3	5
1	1	1	1	1	1	1	3	5
0	0	0	0	0	0	0	1	5
1	1	1	1	1	1	1	3	5
1	1	1	1	1	1	1	3	5
1	1	1	1	1	1	1	3	5
1	1	0	1	1	1	1	3	5
1	1	0	0	1	1	0	2	5
1	1	1	0	1	1	0	1	5
0	0	0	0	0	0	0	1	5
1	0	0	1	1	0	0	1	5
1	0	1	1	0	1	1	1	2
0	0	0	0	0	0	0	2	5
0	0	0	0	0	0	0	1	4
0	0	0	0	0	0	0	1	5
1	0	1	0	0	1	0	1	1
1	1	1	0	0	0	0	1	2
1	1	0	0	0	1	0	1	1
1	1	1	1	0	1	1	3	5
0	0	0	0	0	0	0	1	5
0	0	0	0	0	0	0	1	3
1	1	0	0	1	1	0	1	1
0	0	0	0	0	0	0	1	2
0	0	0	0	0	0	0	1	4
1	1	0	0	1	0	0	1	2
0	0	0	0	0	0	0	1	1
1	0	1	0	1	0	1	2	3
1	1	1	0	1	0	1	3	5
1	1	0	0	0	1	0	1	5
1	0	1	0	1	1	0	3	5
0	0	0	0	0	0	0	3	5
1	0	1	1	1	0	1	1	3
1	1	0	0	0	0	0	1	2

1	1	1	1	1	1	0	1	3
1	1	1	1	0	1	1	2	2
1	1	1	0	1	0	1	1	3
1	1	1	0	0	1	1	2	5
0	0	0	0	0	0	0	1	4
1	1	1	1	0	1	1	1	1
1	0	0	0	0	0	0	2	5
1	0	1	0	1	1	0	2	3
1	1	0	1	0	0	1	2	4
0	0	0	0	0	0	0	2	5
1	0	1	0	1	1	0	2	4
0	0	0	0	0	0	0	2	5
1	0	1	0	1	0	1	2	3
1	1	1	0	1	1	1	3	5
0	0	0	0	0	0	0	2	5
1	0	1	0	1	1	1	2	5
1	0	1	0	1	1	1	2	5
1	1	1	0	1	1	0	2	5
1	1	1	0	1	1	0	2	5
0	0	0	0	0	0	0	2	5
1	0	1	0	1	1	0	2	5
1	1	1	0	1	1	1	2	5
1	1	1	0	1	1	1	2	5
0	0	0	0	0	0	0	1	5
1	0	1	0	1	1	0	1	5
1	1	1	0	1	1	0	2	5
1	0	1	0	1	1	0	2	5
0	0	0	0	0	0	0	3	5
0	0	0	0	0	0	0	1	5
1	1	1	1	1	1	0	1	5
1	0	1	0	1	0	1	2	5
1	0	1	0	1	0	1	2	5
0	0	0	0	0	0	0	2	5
1	0	1	0	1	0	1	1	5
1	0	0	0	1	0	1	3	5
0	0	0	0	0	0	0	3	5
1	0	1	0	1	1	0	3	5
0	0	0	0	0	0	0	3	5
0	0	0	0	0	0	0	3	5
0	0	0	0	0	0	0	3	5
1	1	1	0	1	1	0	3	5
1	1	1	0	1	1	0	3	5
1	0	1	0	1	0	1	3	5
1	0	1	1	0	0	1	3	5
1	1	1	1	1	1	1	3	5
1	1	1	0	1	0	0	3	5
1	1	1	0	1	1	1	3	5

1	0	0	1	0	0	0	3	5
1	0	0	0	1	1	1	3	5
1	0	0	0	1	0	1	3	5
1	0	0	0	1	1	1	3	5
1	0	0	0	0	0	0	3	3
0	0	0	0	0	0	0	3	5
1	1	1	0	1	1	1	3	5
1	1	1	1	1	1	1	1	5
1	1	1	1	1	1	1	3	5
1	1	1	0	1	1	1	3	5
1	1	1	1	1	1	1	3	5
1	1	1	1	1	1	1	3	5
1	1	1	1	1	1	1	1	5
1	1	1	1	1	1	1	3	5
0	0	0	0	0	0	0	3	5
1	1	1	1	1	1	1	3	5
0	0	0	0	0	0	0	3	5
1	1	1	1	1	1	1	3	5
0	0	0	0	0	0	0	3	5
0	0	0	0	0	0	0	3	5
1	1	1	1	1	1	1	3	5
1	1	1	1	1	1	1	3	5
1	0	0	0	1	0	1	3	5
0	0	0	0	0	0	0	3	5
0	0	0	0	0	0	0	3	5
0	0	0	0	0	0	0	3	5
1	0	1	0	1	1	0	3	5
0	0	0	0	0	0	0	3	5
1	0	1	1	1	0	0	3	5
0	0	0	0	0	0	0	3	5
1	0	1	0	1	1	0	3	5
1	1	1	0	1	1	0	3	5
1	0	1	0	1	1	0	3	3
1	0	1	1	1	1	0	3	5
1	1	1	0	1	1	0	3	5
1	0	0	0	0	0	0	3	5
1	1	1	1	0	1	1	1	5
1	1	1	0	0	1	0	3	5
0	0	0	0	0	0	0	1	5
1	1	1	0	1	1	1	3	5
1	0	0	0	1	0	0	3	5
1	1	0	0	0	0	0	3	5
1	0	1	0	1	1	1	3	5
0	0	0	0	0	0	0	2	5
0	0	0	0	0	0	0	3	5
1	0	1	0	1	1	1	3	5
1	0	1	0	1	1	1	3	5

1	0	1	0	1	1	1	2	5
1	0	1	0	1	1	1	2	5
1	0	1	0	1	1	1	2	5
1	0	1	0	1	1	1	2	5
1	0	0	0	1	0	0	2	5
1	1	0	0	0	0	0	3	5
1	1	0	0	1	0	0	2	5
1	1	1	0	0	0	1	3	5
1	1	1	0	0	1	1	2	5
1	1	1	0	0	1	1	2	5
1	0	1	0	1	1	0	2	5
1	0	1	0	1	1	1	1	5
1	0	1	0	1	1	1	3	5
0	0	0	0	0	0	0	3	5
0	0	0	0	0	0	0	3	5
1	0	1	0	1	1	1	3	5
1	0	1	1	1	1	1	1	5
0	0	0	0	0	0	0	3	5
1	0	1	0	0	1	0	3	5
1	1	1	0	1	1	0	3	5
0	0	0	0	0	0	0	3	5
1	1	1	0	1	1	0	3	5
1	1	0	0	1	1	0	3	5
1	0	1	0	0	1	0	3	5
1	0	0	0	0	1	0	3	5
1	0	1	0	1	1	0	3	5
1	1	1	1	1	1	1	3	5
1	1	1	1	1	1	1	3	5
1	1	1	0	0	0	0	3	5
0	0	0	0	0	0	0	1	5
1	0	1	0	0	1	1	1	1
1	0	1	0	1	1	1	1	5
1	0	1	0	0	1	0	1	3
1	1	1	0	1	1	1	2	3
0	0	0	0	0	0	0	1	1
0	0	0	0	0	0	0	2	3
0	0	0	0	0	0	0	1	5
1	0	0	0	0	1	0	1	3
1	0	1	0	1	1	0	2	5
0	0	0	0	0	0	0	3	5
0	0	0	0	0	0	0	1	5
0	0	0	0	0	0	0	1	1
0	0	0	0	0	0	0	2	5
1	1	0	0	1	1	0	1	1
0	0	0	0	0	0	0	2	5
0	0	0	0	0	0	0	1	5
1	1	0	0	0	1	0	1	2

0	0	0	0	0	0	0	1	1
0	0	0	0	0	0	0	1	5
0	0	0	0	0	0	0	1	5
0	0	0	0	0	0	0	2	4
1	1	1	0	0	1	0	2	5
1	0	1	0	0	1	0	2	5
1	0	0	0	0	0	0	2	5
1	0	0	0	0	0	0	1	4
0	0	0	0	0	0	0	2	3
1	0	1	0	0	1	0	1	3
0	0	0	0	0	0	0	2	5
0	0	0	0	0	0	0	2	5
0	0	0	0	0	0	0	1	3
0	0	0	0	0	0	0	2	5

L3SOURCE(	L3aOtherlig	L4TypeofFu	L4aotherFu	Radio	Computer	ColorBlack	Electricfan	Satellitedis
3		5		0	0	0	0	0
1		5		0	0	1	0	1
1		5		0	0	0	0	0
4		5		0	0	0	0	0
1		3		1	1	0	1	0
4		3		1	0	1	0	1
1	1	1		0	0	1	1	1
1		2	charcoal	1	0	1	1	1
1		3		1	0	1	0	1
1		5		1	0	1	0	1
1		3		1	0	1	1	1
1		5		1	0	0	0	0
3	flash light;	5		1	0	0	0	0
1	Maches	3		0	0	0	0	0
1		5		1	0	1	1	1
1		5		1	0	1	0	1
4		5		1	0	0	0	0
4		3	firewood	1	1	0	1	0
1		5		0	0	1	0	1
1		1		0	0	1	0	1
4		5		0	0	0	0	0
1		5		1	0	1	1	1
1		3	firewood	1	0	1	1	1
1		2	charcoal	1	1	1	1	1
1		3	0	0	0	0	0	0
1		5		1	1	1	1	1
1		5		0	0	1	1	1
1		5		0	0	0	1	0
1		5		1	0	1	0	1
2		5		1	0	0	0	0
4		5		1	0	0	0	0
3		5		0	0	0	0	0
1		5		1	0	0	0	0
4		5		0	0	0	0	0
4		5		0	0	0	0	0
4		5	1	1	0	1	0	1
4		5		0	0	0	0	0
4		3		0	0	0	0	0
4		5		1	0	1	0	1
4		5		0	0	0	0	0
4		5		1	0	0	0	0
4		3	firewood	0	0	1	1	1
1		5		0	0	1	1	1
1		5		0	0	1	1	1
1		5		0	0	0	0	0
1		5		0	0	0	0	0

1	5		0	0	0	0	0
1	5		0	0	0	0	0
1	5		0	0	0	0	0
4	5		0	0	0	0	0
3	5		0	0	0	0	0
1	3		1	1	1	1	1
4	5		0	0	0	0	0
4	5		1	0	0	0	0
5	4		1	0	1	1	1
4	5		0	0	0	0	0
4	5		1	0	0	0	0
4	5		0	0	0	0	0
4	5		0	0	0	0	0
3	1		1	0	1	1	1
4	5		0	0	0	0	0
4	5		0	0	0	0	0
4	5	0	0	0	0	0	0
1	5		0	0	0	1	0
4	5		1	0	0	0	0
4	5		1	0	0	0	0
4	5		1	0	0	0	0
4	5		0	0	0	0	0
4	5		1	0	0	0	0
4	5		0	0	0	0	0
4	5	0	0	0	0	0	0
4	5		0	0	0	0	0
4	6	corn stocks	0	0	0	0	0
1	5		1	0	0	1	0
1	5		0	0	1	0	1
4	2		0	0	0	0	0
4	4		0	0	0	0	0
4	5		1	0	0	0	0
4	5		1	0	0	0	0
4	5		0	0	0	0	0
4	5		0	0	0	0	0
4	5		1	0	0	0	0
2	5	0	0	0	0	0	0
1	2		1	0	1	1	1
4	5	1	1	0	0	0	0
1	3		1	0	1	0	1
4	5		1	0	0	0	0
4	5		0	0	0	0	0
1	5		1	0	0	0	0
4	5		1	0	0	0	0
4	5		1	0	0	0	0
4	5		0	0	0	0	0
3	5		1	0	0	0	0

1		4	0	0	0	0	0
1		5	1	0	0	0	0
1		5	0	0	1	0	1
1	Torch Light	5	0	0	0	0	0
4		5	1	0	1	1	0
4		5	0	0	0	0	0
4		5	1	0	0	1	1
4		5	0	0	0	0	0
4		5	0	0	0	0	0
4		5	0	0	0	0	0
4		5	1	0	0	0	0
4		5	0	0	0	0	0
4		5	1	0	0	0	0
4		5	0	0	0	0	0
4		5	1	0	0	0	0
4		5	1	0	0	0	0
4		5	1	0	0	0	0
4		5	0	0	0	0	0
4		5	0	0	0	0	0
4		5	1	0	0	0	0
4		5	0	0	0	0	0
4		5	1	0	0	0	0
4		5	0	0	0	0	0
4		5	1	0	0	0	0
4		5	1	0	0	0	0
4		5	1	0	0	0	0
4		5	1	0	1	0	1
1		5	0	0	0	0	0
1		5	0	0	1	0	1
4		5	1	0	0	0	0
4		5	0	0	0	0	0
4		5	1	0	0	0	0
1		5	1	0	1	1	1
4		5	0	0	0	0	0
3		5	1	0	1	0	0
1		5	1	0	1	0	1
4		5	0	0	0	0	0
3		5	0	0	0	0	0
4		5	1	0	1	0	1
4		5	1	0	0	0	0
4		5	0	0	0	0	0
4		5	1	0	0	0	0
4		5	1	0	0	0	0
4		5	1	0	0	0	0
4		5	1	0	0	0	0
4		5	0	0	0	0	0
4		3	1	0	0	0	0

1	5	0	0	1	0	1
1	5	1	0	1	0	1
1	5	1	0	0	0	0
1	3	1	0	1	1	1
1	3	1	1	1	1	1
1	1	1	0	1	1	1
4	3	0	0	0	0	0
4	5	1	0	1	1	1
1	3	0	0	1	1	1
1	3	1	0	1	1	1
1	5	1	0	1	1	1
1	5	1	0	1	1	1
4	5	1	0	0	0	0
4	5	0	0	0	0	0
4	5	0	0	0	0	0
4	5	1	0	0	0	0
4	5	0	0	0	0	0
1	5	1	0	1	1	1
4	5	0	0	0	0	0
4	5	1	0	0	0	0
4	1 0	0	0	0	0	0
1	4	1	0	0	0	0
4	5	0	0	0	0	0
4	5	0	0	1	0	1
1	5	0	0	1	0	1
4	5	0	0	0	0	0
4	5	1	0	0	0	0
4	5	1	0	0	0	0
4	5	0	0	0	0	0
4	5	0	0	0	0	0
4	5	1	0	0	0	0
4	5	0	0	0	0	0
4	5	0	0	0	0	0
4	5	0	0	0	0	0
4	5	1	0	0	0	0
4	5	1	0	0	0	0
4	5	0	0	0	0	0
4	5	1	0	0	0	0
4	5	0	0	0	0	0
2	5	1	0	0	0	0
4	5	1	0	0	0	0
4	5	1	0	1	0	1
4	5	1	0	0	0	0
4	5	0	0	0	0	0
4	5	1	0	0	0	0
4	5	1	0	0	0	0
4	5	1	0	0	0	0

1		5		0	0	1	0	1
1		3		0	0	1	0	1
4	Flash light	5		1	0	0	0	0
1		3		1	0	1	1	1
1		5		1	0	1	0	1
4	Lamp	5		0	0	0	0	0
5	dry cell tou	5		1	0	0	0	0
1		5		1	0	1	1	1
4		5		1	0	0	0	0
4		5		1	0	0	0	0
4		5		0	0	0	0	0
4		5		1	0	0	0	0
4		5		1	0	0	0	0
4		5		0	0	0	0	0
4		5		1	0	0	0	0
4		5		0	0	0	0	0
4		5		1	0	0	0	0
1	0	3	firewood	1	0	0	0	0
4		5		1	0	0	0	0
4		5		0	0	0	0	0
1		5		0	0	0	0	0
4		5		1	0	0	0	0
4		5		0	0	0	0	0
4		5		0	0	0	0	0
1		3	Firewood	0	1	1	1	1
1		2		1	1	1	1	1
1		3		1	0	1	1	1
1		3		1	0	1	1	1
1		2	charcoal	1	1	1	1	1
1		3		1	1	1	0	1
1		3		0	0	1	1	1
1		2		1	0	1	1	1
4		5		1	0	0	0	0
1		3		1	0	1	1	1
4		5		0	0	0	0	0
4		5		0	0	0	0	0
1		5		1	0	1	0	1
4		5		0	0	0	0	0
4		5		0	0	0	0	0
4		5		0	0	0	0	0
4	0	5	0	1	0	0	0	0
4		5		1	0	0	0	0
1		5		1	0	0	0	0
4		5		0	0	0	0	0
4		5		0	0	0	0	0
4		1	millet stalk	0	0	0	0	0
4		5		0	0	0	0	0

4		5		0	0	0	0	0
4		5		1	0	0	0	0
4		5		0	0	0	0	0
4		5		1	0	0	0	0
4		6	millet stalk	1	0	0	0	0
4		5		0	0	0	0	0
4		5		1	0	0	0	0
4		5		0	0	0	0	0
1		3		0	0	1	1	1
1		2	charcoal	1	0	1	1	1
1		3		1	0	1	1	1
4		4		0	0	1	0	1
1		2		1	0	1	0	1
1		5		0	0	1	1	1
4		5		0	0	0	0	0
1		3		1	1	1	1	1
4		5		0	0	0	0	0
4		3		1	0	0	0	0
4		5		1	0	0	0	0
1		5		1	0	1	0	1
4		5		0	0	0	0	0
4		5		0	0	0	0	0
4		5		0	0	0	0	0
4		5		0	0	0	0	0
4		5		0	0	0	0	0
4		5		1	0	0	0	0
4		5		1	0	0	0	0
4		5		0	0	0	0	0
4	0	5	0	0	0	0	0	0
4		5		0	0	0	0	0
4		5		0	0	0	0	0
4		5		0	0	0	0	0
4	0	3	firewood	0	0	0	0	0
4		5		0	0	0	0	0
4		5		1	0	0	1	1
5	lamp	3		1	0	1	0	1
4		5		0	0	0	0	0
1		3		1	0	1	1	0
1		3	1	1	1	1	1	1
3		3		1	0	0	0	0
1		3		1	0	1	1	1
1		2		1	0	1	1	1
4		5		0	0	0	0	0
1		3		1	0	1	1	1
3		3		0	0	0	0	0
4		5		1	0	0	0	0
4		5		1	0	0	0	0

1		3		1	1	1	0	1
4		5		0	0	0	0	0
1		3		1	0	1	1	1
1		3		1	0	1	1	1
4		3		1	0	0	0	0
3		3		0	0	0	0	0
1		5		1	0	1	0	1
1		3		1	0	0	0	0
1		3		1	0	1	1	1
4		5		1	0	0	0	0
1		5		1	0	1	1	1
4		5		0	0	0	0	0
4		5		1	0	0	0	0
4		3	Firewood	1	0	0	0	0
1	lamp	5		0	0	0	0	0
1		3	firewood	1	0	1	1	1
1	Lamp	2		0	0	1	1	1
4	Lamp	5		1	0	0	0	0
1		5		1	0	1	1	0
4		3		1	0	0	0	0
4		5		1	0	0	0	0
1		3		1	1	1	1	1
1		3		1	1	1	1	1
4		3	firewood	1	0	1	0	1
4		5		1	0	0	0	0
4		5		0	0	0	0	0
4		5		1	0	0	0	0
4		5		1	0	0	0	0
1		2	Charcoal	1	0	1	1	1
1		3		0	1	0	0	0
1		3		1	0	1	1	1
1		3		1	0	1	1	1
1	0	2	0	1	1	1	1	1
1	0	3	0	1	0	1	1	1
4		5	0	1	0	0	0	0
4		5		0	0	0	0	0
4	0	5	0	1	0	0	0	0
4		5	0	1	0	0	0	0
4	0	5	0	1	0	0	0	0
4	0	5	0	1	0	0	0	0
3		5	0	1	0	0	0	0
1	0	3	0	1	0	1	1	1
1	0	3	0	0	0	1	1	1
4	0	5	0	1	0	0	0	0
5	0	5		1	0	1	0	1
1	0	3	firewood	1	0	0	0	0
3	0	5		1	0	0	0	0

3		5		0	0	0	0	0
4		5		1	0	0	0	0
1		5		1	0	1	1	1
4		3	0	1	0	0	0	0
1		3		0	0	0	0	0
1		5		0	0	0	0	0
1	0	2	0	1	1	1	1	1
1	0	3	0	1	1	1	1	1
4	0	5	0	1	0	0	0	0
1	0	5	0	1	0	0	0	0
1	0	3	0	1	0	0	1	0
1	0	5	0	1	1	1	1	1
4	0	5	0	0	0	0	0	0
2	torch light	3	firewood	1	0	0	0	0
4	0	5	0	1	0	0	0	0
4	0	5	0	1	0	1	0	1
1	0	3	firewood	1	0	1	0	0
1	0	5	0	1	0	1	1	1
4	0	5	0	1	0	0	0	0
4		5		0	0	0	0	0
4	0	5	0	1	0	0	0	0
1		3		1	1	1	0	1
1		3		1	0	1	1	1
1		3		0	0	1	1	1
1		3		1	0	1	0	1
1		2	Charcoal	1	1	1	1	1
4	Lamp	3	firewood	1	0	0	0	0
1		5		1	0	1	1	1
1		3		1	0	1	1	1
1		2		1	1	1	1	1
1		3		1	0	1	1	1
1		2	Charcoal	1	0	1	1	1
4		5		1	1	0	1	1
5		3	firewood	1	0	0	0	0
1		2	Charcoal	1	0	1	1	1
1		2	Charcoal	1	0	1	0	1
1		2		1	1	1	1	1
1		2	charcoal	1	1	1	1	1
1		3		1	1	1	1	1
1		2	CHARCOAL	1	0	1	1	1
1	Tough ligh	5		0	0	0	0	0
1		2	charcoal	1	1	1	1	1
1		2	charcoal	1	0	1	1	1
1		3		0	0	1	1	1
1		5		0	0	0	0	0
1		2	charcoal	1	0	1	1	1
1		2	Charcoal	1	1	1	1	1

1		2	charcoal	1	0	1	1	1
1		3		1	0	0	1	0
1		2	Charcoal	1	0	1	1	1
4		3		0	0	0	0	0
1		2	Charcoal	1	0	1	1	1
1		2	charcoal	1	1	1	1	1
4		5		0	0	0	0	0
1	Touch light	5		1	0	0	0	0
1		3		1	0	0	0	0
4	0	3	firewood	0	0	0	0	0
1	0	5	0	1	0	0	1	0
1	torch light	5	0	1	0	1	1	1
4	0	5		1	0	0	0	0
5	0	5	0	0	0	0	0	0
1	0	5	0	0	1	0	0	0
1	0	5	0	1	0	1	0	1
4	0	5	0	0	0	0	0	0
4	0	5		1	0	0	0	0
5	0	5	0	1	1	1	1	1
4	0	5	0	0	0	0	0	0
4	0	5	0	1	0	0	0	0
4	0	5	0	1	0	0	0	0
4	0	5	0	0	0	0	0	0
1	0	5	0	1	0	1	0	1
1	0	3	0	1	1	1	1	0
1	0	5	0	0	0	1	1	1
4	0	5	0	1	0	1	0	1
1	0	3	0	1	0	1	1	1
4	0	5	0	0	0	0	0	0
1	0	2	0	1	1	1	1	1
1	0	5	0	1	0	1	0	1
4	0	5	0	0	0	0	0	0
4	0	5	0	0	0	0	0	0
4	0	5	0	1	0	0	0	0
3	torch light	5	0	0	0	1	0	0
4	0	5	0	0	0	0	0	0
4	0	3	0	0	0	0	0	0
4	0	5	0	0	0	0	1	0
4	0	5	0	0	0	0	0	0
4		5		1	0	0	0	0
4	0	5	0	1	0	0	0	0
4	0	5		0	0	0	0	0
4	0	5	0	1	0	0	0	0
4	0	5	0	0	0	0	0	0
4	0	5	0	0	0	0	0	0
4	0	3	0	1	0	0	0	0
4		5		1	0	0	0	0

4		5		0	0	0	0	0
4	0	5	0	0	0	0	0	0
4		5	0	0	0	0	0	0
4		5	0	1	0	0	0	0
2	0	3	firewood	1	0	1	1	1
4		5		0	0	0	0	0
4		5		1	0	0	0	0
4		5		1	0	0	0	0
4		5		1	0	0	0	0
4	0	5	chacoal	1	0	0	0	0
4		5		0	0	0	0	0
4	0	5	0	1	0	0	0	0
1		5		1	0	1	1	1
4	0	5	0	0	0	1	0	1
1		5		0	0	1	0	1
4		5		0	0	0	0	0
4		5		1	1	1	1	0
4		5		1	0	0	0	0
4		5		1	0	0	0	0
4		5		0	0	0	0	0
4		5		0	0	0	0	0
4		5		0	0	0	0	0
4	0	5	0	0	0	0	0	0
4		5		0	0	0	0	0
4		5		1	0	0	0	0
4		5		1	0	1	0	1
4	0	5	0	1	0	0	0	0
4		5		0	0	1	0	1
4		5		1	0	0	0	0
4		5		1	0	0	1	0
4	0	5	0	0	0	0	0	0
4	0	5	0	1	0	0	0	0
4	0	5	0	1	0	0	0	0
4	0	5	0	0	0	0	0	0
4	0	5	0	0	0	1	0	1
1	0	5	0	0	0	0	0	0
1	0	5	0	0	0	0	0	0
1	0	5	0	0	0	0	0	0
4	0	5	0	0	0	0	0	0
4	0	5	0	0	0	0	0	0
4	0	5	0	0	0	0	0	0
4	0	5	0	0	0	0	0	0
4	0	5	0	0	0	0	0	0
4	5	5		1	0	0	0	0
4	0	5	0	0	0	1	0	0
4	0	5	0	0	0	0	0	0
4	0	5	0	0	0	0	0	0

4	0	5	0	1	0	0	0	0
4	0	5	0	1	0	0	0	0
4	0	5	0	0	0	0	0	0
4	0	5	0	1	0	0	0	0
4	0	5	0	1	0	0	0	0
4	0	5	0	0	0	0	0	0
4	0	5	0	0	0	0	0	0
4	0	5	0	0	0	0	0	0
4	0	5	0	1	0	0	0	0
4	0	5	0	1	0	0	0	0
4	0	5	0	1	0	1	0	1
4	0	5	0	1	1	0	1	0
4	0	5	0	1	0	0	0	0
4	0	5	0	1	0	0	0	0
4	0	5	0	1	0	1	0	1
4	0	5	0	1	0	0	0	0
4	0	5	0	1	0	0	1	0
4	0	5	0	0	0	0	0	0
4		5	0	0	0	0	0	0
4		6	Corn stock	0	0	1	0	1
4		5		1	0	0	0	0
4		5		0	0	0	0	0
4		5		1	0	0	0	0
4		5		1	0	0	0	0
4		5		0	0	0	1	0
1		5		1	0	1	1	1
4	Lamp	5		1	0	0	0	0
4		5		1	0	0	0	0
1		5		1	0	1	0	1
1		2	Charcoal	1	0	1	1	1
1	Tough light	2	charcoal	0	1	1	1	1
1	lamp	3		0	0	1	1	1
1		5		0	0	1	0	1
1		2		0	0	1	1	1
4		2		1	0	0	0	0
4		5		1	0	0	0	0
1		2	1	1	1	1	1	1
4		2		1	1	1	1	1
1		5		0	0	0	0	0
1		1		1	0	1	1	1
1		2		1	0	1	1	1
1	solar	2		1	1	1	1	1
1		5		0	1	1	1	1
1		3		1	1	1	1	1
4		5		0	0	0	0	0
4		3		1	0	0	0	0
1		5		1	0	1	0	1

1 solar	2	1	1	1	1	1
4	2	1	1	1	1	1
4	2	1	1	1	1	1
4	1	0	1	1	1	1
1	2	1	0	1	1	1
1	2	1	1	1	1	1
1	5	1	0	1	0	1
1	3	1	0	1	0	0
1	2	1	1	1	1	1
1	1	0	0	1	1	1
4	5	0	1	1	1	1
1	2	1	1	1	1	1
1	2	0	0	1	1	1
4	5	1	0	0	0	0

Mobilepho	SewingMa	Bicycle	Mattress	Motrocycle	Refrigerato	Animaldrav	DVDVCD	Car
0	0	1	0	0	0	0	0	0
1	0	1	0	1	0	0	0	0
1	0	1	1	1	0	0	0	0
1	0	0	0	0	0	0	0	1
1	0	0	1	0	0	0	0	0
1	1	1	1	0	0	0	0	0
1	0	1	1	1	1	0	0	0
1	0	1	1	1	1	0	1	0
0	0	0	1	0	1	0	1	0
1	0	1	1	0	0	0	0	0
1	0	1	1	1	1	0	0	0
1	0	1	1	1	0	1	0	0
0	0	1	1	0	0	0	0	0
1	0	0	1	0	0	0	0	0
1	0	1	1	1	1	1	1	1
1	0	1	1	1	1	1	1	1
0	0	0	0	0	0	1	0	0
1	1	1	1	1	0	1	0	0
1	0	0	1	0	0	0	0	0
1	1	1	0	1	0	0	0	0
0	0	1	0	0	0	0	0	0
1	0	1	1	0	0	0	0	0
1	1	1	1	0	0	0	0	0
1	1	1	1	1	1	1	1	0
1	1	1	1	0	0	0	0	0
1	1	1	0	0	1	0	1	0
1	0	1	1	1	0	0	0	0
1	0	0	0	0	0	0	0	0
1	0	1	1	1	0	0	0	0
1	1	1	1	1	0	0	0	0
0	0	1	0	0	0	0	0	0
1	0	1	0	1	0	1	0	0
0	0	1	0	0	0	1	0	0
1	0	1	0	0	0	0	0	0
1	0	0	0	0	0	0	0	0
1	0	1	1	1	0	0	0	0
1	1	1	1	0	0	0	0	0
1	1	1	1	1	0	0	0	0
1	1	1	0	0	0	1	1	0
1	0	1	1	0	0	1	0	0
1	1	1	1	0	0	0	0	0
1	0	1	0	0	1	0	0	0
1	0	1	0	0	1	0	0	0
1	0	1	0	0	1	0	0	0
0	0	1	0	0	0	1	0	0
1	0	1	0	1	0	1	0	0

0	0	1	0	0	0	1	0	0
1	0	1	0	0	0	0	0	0
1	0	1	0	0	0	0	0	0
1	0	1	0	0	0	0	0	0
0	0	1	0	0	0	0	0	0
1	1	1	1	1	1	1	1	1
1	0	1	0	0	0	0	0	0
1	0	1	0	0	0	0	0	0
0	0	0	1	0	0	0	0	0
0	0	1	0	0	0	0	0	0
0	0	1	1	0	0	0	0	0
1	0	1	1	0	0	0	0	0
1	0	1	0	0	0	0	0	0
1	0	1	0	1	1	1	1	0
1	0	1	1	0	0	0	0	0
0	0	1	0	0	0	1	0	0
0	1	0	1	0	0	1	0	0
0	0	1	1	0	0	0	0	0
1	0	0	0	0	0	0	0	0
0	0	1	1	0	0	0	1	0
0	0	1	1	0	0	1	0	0
0	0	1	1	0	0	0	0	0
1	0	1	1	0	0	0	0	0
0	0	1	0	0	0	1	0	0
0	0	1	1	1	0	0	0	0
1	1	1	1	0	0	0	0	0
1	0	1	0	0	0	0	0	0
1	0	1	1	0	0	0	0	0
1	0	1	1	0	0	0	0	0
0	0	1	1	0	0	0	0	0
1	0	1	0	0	0	0	0	0
0	0	1	1	1	0	1	0	0
1	0	1	1	1	0	0	0	0
0	0	0	1	0	0	0	0	0
0	0	1	0	0	0	0	0	0
0	0	1	0	0	0	0	0	0
1	0	1	1	1	0	0	0	0
1	0	0	1	1	1	0	0	0
1	0	1	1	0	0	0	0	0
1	0	1	0	1	0	0	0	0
1	0	1	1	0	0	0	0	0
1	1	1	1	1	0	0	0	0
1	0	1	1	0	0	0	0	0
0	0	1	1	0	0	1	0	0
1	0	1	0	0	0	0	0	0
1	0	1	0	0	0	0	0	0
1	0	0	0	1	0	0	0	1

1	0	1	0	1	0	1	0	0
1	0	1	0	0	0	1	0	0
1	0	1	1	0	0	0	0	0
0	0	0	0	0	0	0	0	0
0	0	1	1	0	1	0	0	0
0	0	1	0	0	0	0	0	0
1	1	1	1	0	0	0	0	0
0	0	1	0	0	0	0	0	0
1	0	0	0	0	0	0	0	0
1	0	1	0	0	0	0	0	0
0	0	1	1	1	0	0	0	0
1	0	0	0	0	0	0	0	0
0	0	1	0	0	0	1	0	0
1	0	1	0	0	0	0	0	0
1	1	1	1	0	0	0	0	0
1	0	1	1	1	0	0	0	0
0	0	1	0	0	0	0	0	0
1	0	1	1	0	0	0	0	0
1	0	1	1	0	0	0	0	0
0	0	1	1	0	0	0	0	0
1	0	0	0	0	0	0	0	0
0	0	1	1	0	0	0	0	0
1	0	1	0	1	0	1	0	0
0	0	1	1	0	1	0	0	0
1	1	1	1	1	0	0	0	1
0	0	1	0	0	0	0	0	0
1	0	1	1	0	1	0	0	0
1	0	0	0	0	0	0	0	0
1	0	0	1	0	0	0	0	0
1	0	1	1	0	0	1	0	0
1	0	0	0	0	0	0	0	0
1	0	1	1	0	0	0	0	0
1	0	1	1	1	0	0	1	0
1	0	1	0	0	0	0	0	0
1	0	0	1	0	0	0	0	0
1	0	1	1	1	1	0	0	1
1	0	0	0	0	0	0	0	0
1	0	0	1	1	0	0	0	0
1	1	1	1	1	0	1	0	0
1	0	1	1	0	0	0	0	0
1	0	1	0	0	0	0	0	0
1	0	1	0	0	0	0	0	0
1	0	1	1	0	0	1	0	0
1	0	1	1	1	0	0	0	0
1	0	1	1	0	0	1	0	0
1	0	1	0	0	0	0	0	0
1	0	1	1	0	0	0	0	0

1	0	1	1	0	0	0	0	0
0	0	0	1	0	1	1	1	0
1	0	0	0	0	0	0	0	0
1	1	0	1	0	1	0	1	0
1	1	0	1	1	1	0	0	0
1	0	1	1	1	1	0	1	0
1	0	1	1	0	0	0	0	0
1	0	1	1	1	0	0	0	0
1	0	0	1	0	0	0	0	0
1	0	0	1	1	1	0	1	0
1	0	1	1	1	1	0	1	0
1	0	0	1	1	1	1	1	0
1	0	1	0	1	0	0	0	0
1	0	1	0	0	0	1	0	0
1	0	1	0	0	0	1	0	0
1	0	0	0	0	0	0	0	0
0	0	0	0	0	0	1	0	0
1	0	1	0	1	1	1	0	0
1	0	1	1	0	0	0	0	0
0	0	1	0	0	0	0	0	0
1	0	1	0	0	0	0	0	0
0	0	1	1	0	0	0	0	0
1	0	1	0	0	0	0	0	0
1	0	1	0	1	0	1	0	0
0	0	1	0	0	0	1	0	0
1	0	1	0	0	0	0	0	0
1	0	1	1	1	0	1	0	0
1	0	1	0	0	0	0	0	1
1	0	0	0	0	0	0	0	0
1	0	1	0	0	0	0	0	0
1	0	1	0	0	0	0	0	0
1	0	1	1	0	0	0	0	0
0	1	0	1	0	0	0	0	0
1	0	0	1	0	0	0	0	0
0	0	0	0	0	0	0	0	0
0	0	1	1	0	0	0	0	0
1	0	0	1	0	0	0	0	0
0	1	0	1	0	0	0	0	0
1	0	1	1	1	0	0	0	0
1	1	1	1	0	0	0	0	0
1	0	1	0	0	0	0	0	0
0	0	1	1	0	0	0	0	0
0	0	0	0	0	0	0	0	0
0	1	0	0	0	0	0	0	0
1	1	1	0	1	0	1	0	0
1	0	1	0	0	0	0	0	0
0	0	1	0	0	0	0	0	0

1	0	1	1	0	0	0	0	0
1	0	1	1	0	0	0	0	0
0	0	1	0	0	0	0	0	0
1	1	1	1	1	1	1	1	0
1	0	1	1	0	0	0	0	0
0	0	0	1	0	0	0	0	0
1	0	1	0	0	0	0	0	0
1	0	1	0	0	0	0	0	0
1	0	1	1	1	0	1	0	0
1	0	1	0	0	0	0	0	0
1	0	1	0	0	0	0	0	0
1	0	1	0	1	0	1	0	0
1	1	1	1	0	0	0	0	0
1	0	1	0	0	0	0	0	0
1	0	1	0	0	0	0	0	0
0	0	1	0	0	0	0	0	0
1	0	1	0	0	0	1	0	0
1	1	1	1	0	0	0	0	0
1	0	1	0	1	0	1	0	0
1	0	1	0	0	0	0	0	0
1	1	0	0	0	0	0	0	0
0	0	1	1	1	0	0	0	0
1	0	1	0	1	0	0	0	0
1	0	1	0	0	0	1	0	0
1	0	0	1	1	1	0	0	0
1	0	0	0	1	1	1	1	0
1	0	1	1	0	0	0	0	0
1	0	1	1	0	0	0	0	0
1	0	1	1	1	1	0	1	0
1	1	1	1	1	0	1	1	0
1	0	1	1	0	0	0	0	0
1	0	1	1	0	1	0	1	0
1	0	0	0	0	0	0	0	0
1	0	1	1	1	1	1	1	0
1	0	1	1	1	0	0	0	0
1	0	1	0	0	0	0	0	0
1	0	1	0	0	1	0	0	0
1	0	0	0	0	0	0	0	0
1	0	0	0	0	0	0	0	0
0	0	1	0	0	0	0	0	0
1	0	1	0	1	0	0	0	0
0	0	1	0	0	0	0	0	0
1	1	0	1	0	0	0	0	0
1	0	1	0	1	0	0	0	0
1	0	0	0	0	0	0	0	0
1	0	0	0	0	0	0	0	0
1	0	0	0	0	0	0	0	0

1	1	0	1	0	0	0	0	0
1	0	1	1	0	0	0	0	1
1	0	1	0	0	0	0	0	0
1	1	1	0	1	0	1	0	0
0	0	0	0	0	0	0	0	0
1	1	1	0	1	0	0	0	0
1	0	0	0	0	0	0	0	0
1	0	0	0	0	0	0	0	0
1	0	0	1	1	1	1	1	0
1	0	1	1	1	1	0	0	0
1	0	1	1	0	1	0	0	0
0	0	1	1	1	1	0	0	0
1	1	1	1	1	1	0	1	1
1	0	0	1	0	0	0	0	0
1	1	1	1	1	0	0	0	0
1	0	1	1	1	1	0	1	0
0	0	1	0	0	0	0	0	0
1	0	1	0	1	0	1	0	0
1	0	1	1	1	0	1	0	0
1	0	1	1	0	0	0	0	0
1	0	1	0	0	0	1	0	0
1	0	0	0	0	0	0	0	0
1	0	1	0	1	0	1	0	0
1	0	0	0	0	0	0	0	0
1	0	1	0	0	0	0	0	0
0	0	1	1	0	0	0	0	0
0	0	1	1	0	0	0	0	0
1	1	1	0	1	0	1	0	0
1	0	0	0	0	0	0	0	0
1	0	1	0	0	0	0	0	0
0	0	1	0	0	0	0	0	0
1	1	1	1	1	0	0	0	0
0	0	1	0	0	0	0	0	0
1	0	1	0	0	0	1	0	0
0	0	1	1	0	1	0	0	0
1	0	1	0	1	0	0	0	0
1	0	1	0	0	0	0	0	0
1	1	1	1	1	0	0	0	0
1	0	1	1	1	1	1	1	0
1	0	1	0	1	0	1	0	0
1	0	1	1	0	0	0	0	1
1	0	1	1	0	1	1	1	0
1	0	1	0	0	0	0	0	0
1	0	1	1	1	1	0	1	0
1	0	1	0	1	0	0	0	0
1	0	1	1	0	0	0	0	0
0	0	1	0	0	0	0	0	0

1	1	1	1	0	1	1	1	0
0	0	0	0	0	0	0	0	0
1	0	1	1	1	1	0	0	0
1	1	1	1	1	1	1	1	0
1	0	1	1	1	0	1	0	0
1	0	1	0	1	0	0	0	0
1	0	1	0	1	0	1	0	0
1	0	1	0	0	0	1	0	0
1	0	1	1	1	1	1	1	0
1	0	1	0	1	0	1	0	0
1	0	1	1	1	1	1	0	0
1	0	1	1	1	0	0	0	0
1	0	1	1	0	0	0	0	0
1	0	1	1	1	0	0	0	0
1	0	1	1	0	0	0	0	0
1	0	1	1	0	0	0	0	0
1	1	1	1	1	1	0	1	0
1	1	1	1	0	0	0	0	0
1	1	1	0	1	0	0	1	0
1	0	1	1	0	0	0	0	0
1	1	1	1	1	0	0	0	1
1	1	1	1	1	0	1	1	0
1	0	0	1	1	1	0	1	0
1	0	1	1	1	0	1	1	0
0	0	1	1	0	0	0	0	0
1	0	1	0	1	0	0	0	0
1	0	0	0	0	0	0	0	0
0	0	1	1	0	0	0	0	0
1	0	1	1	1	1	0	1	0
0	0	0	1	1	1	0	0	1
1	0	1	1	0	1	0	0	1
1	0	1	1	0	1	0	1	0
1	0	0	1	0	1	0	1	1
1	0	1	1	1	0	0	0	0
1	0	1	1	1	0	0	0	0
1	0	1	0	0	0	0	0	0
1	1	1	1	1	0	0	0	0
0	0	1	1	0	0	0	0	0
0	0	1	1	0	0	0	0	0
1	1	1	0	1	0	0	0	0
1	0	1	0	0	0	0	0	0
1	0	1	1	0	1	0	0	0
1	0	1	1	0	0	0	1	0
1	0	1	0	1	0	0	0	0
0	0	1	1	0	1	1	0	0
1	1	1	1	0	0	0	0	0
1	0	1	0	0	0	1	0	0

1	0	0	0	0	0	0	0	0
0	0	1	0	0	0	1	0	0
1	0	1	1	1	1	0	1	0
1	0	1	1	1	0	0	0	0
1	0	1	0	1	0	1	0	0
0	0	1	1	0	0	0	0	0
1	0	1	1	1	1	0	1	0
1	1	1	1	1	1	0	1	0
1	1	1	0	0	0	1	0	0
1	1	0	0	1	0	1	0	0
1	0	1	1	0	0	1	1	0
1	1	0	1	0	1	0	0	0
1	0	0	0	0	0	0	0	0
1	0	1	1	1	0	1	0	0
1	0	1	0	0	0	1	0	0
1	0	1	1	1	0	0	0	0
1	1	1	1	1	0	0	0	0
1	1	1	1	1	0	0	1	0
1	0	1	1	0	0	0	0	0
1	1	0	0	1	0	1	0	0
1	0	1	0	1	0	0	0	0
1	1	1	0	1	1	0	1	0
1	0	1	1	1	1	0	0	0
1	0	1	1	1	1	0	1	1
1	0	1	1	1	0	0	0	0
1	1	0	1	1	1	0	1	0
1	1	1	1	1	0	0	0	0
1	1	1	1	1	1	0	1	0
1	0	0	1	1	1	0	1	0
1	1	0	1	1	1	0	1	0
1	0	1	1	1	1	0	0	0
1	0	0	1	1	1	0	1	0
1	0	0	0	1	0	0	0	1
1	0	1	0	0	0	0	0	0
1	0	0	1	1	1	0	1	0
1	0	1	1	1	1	0	1	0
1	0	0	1	1	1	0	1	0
1	0	0	1	1	1	0	1	0
1	1	1	1	1	1	0	0	1
1	0	1	1	1	1	0	1	1
1	0	1	0	0	0	1	0	0
1	1	1	1	1	1	0	0	0
1	0	1	1	1	1	0	1	0
1	0	1	1	0	0	0	0	0
0	0	1	0	0	0	0	0	0
1	0	0	1	1	1	0	1	0
1	0	0	1	1	1	0	1	0

1	0	0	1	1	1	0	1	0
1	1	0	1	1	0	0	0	0
1	1	1	1	1	1	0	1	0
0	0	1	1	0	0	0	0	0
1	0	1	1	1	1	0	0	0
1	0	1	1	1	1	0	1	1
0	0	1	0	0	0	0	0	0
1	0	1	0	1	0	1	0	0
1	0	0	0	0	0	0	0	0
0	0	1	0	0	0	0	0	0
1	0	1	0	0	0	1	0	0
1	0	1	1	1	1	1	1	0
1	0	1	0	1	0	1	0	0
1	0	0	0	0	0	0	0	0
1	0	1	1	0	0	0	0	0
1	0	0	0	0	0	0	0	0
0	0	0	1	0	0	0	0	0
1	1	1	1	1	0	0	0	0
1	1	1	1	1	0	0	1	0
1	0	0	1	0	0	0	0	0
0	0	1	1	0	0	1	0	0
1	1	1	1	1	0	0	0	0
1	0	0	0	0	0	0	0	0
1	0	0	0	0	0	0	0	0
1	1	1	1	1	0	1	1	0
1	0	1	1	1	1	1	0	1
0	0	1	1	0	0	0	0	0
0	1	1	0	0	0	0	0	0
1	0	1	0	0	0	0	0	0
1	1	1	1	1	1	0	1	0
1	0	1	1	0	1	0	1	1
1	0	1	0	0	0	0	0	0
1	0	0	0	0	0	0	0	0
1	0	1	0	0	0	1	0	0
1	0	1	1	0	0	0	0	0
0	0	0	1	0	0	0	0	0
1	0	0	0	0	0	0	0	0
0	0	0	0	0	0	0	0	0
1	0	0	0	0	0	0	0	0
1	0	1	0	0	0	0	0	0
1	0	0	0	0	0	0	0	0
0	0	0	0	0	0	0	0	0
1	0	0	0	0	0	0	0	0
1	0	1	0	1	0	1	0	0
0	0	0	1	0	0	0	0	0
1	0	1	0	0	0	0	0	0
1	0	1	1	1	0	0	0	0

1	0	1	0	0	0	0	0	1
1	0	0	0	0	0	0	0	0
1	0	1	0	0	0	0	0	0
1	0	1	0	0	0	0	0	0
1	0	1	1	0	0	0	1	0
0	0	1	0	0	0	0	0	0
1	0	1	1	0	0	0	0	0
1	1	1	1	1	0	1	0	0
1	0	1	1	1	0	0	0	0
1	0	1	0	0	0	0	0	0
0	0	1	0	0	0	0	0	0
1	0	1	0	0	0	0	0	0
1	0	0	1	0	1	1	0	0
0	0	1	1	0	0	0	1	0
1	0	1	0	1	0	0	0	0
1	0	1	0	0	0	0	0	0
1	0	1	1	0	0	0	0	0
0	0	1	1	0	1	0	0	0
1	1	1	1	0	0	0	0	0
1	0	0	0	0	0	0	0	0
1	0	1	0	0	0	0	0	0
1	1	0	0	0	0	0	0	0
1	0	1	0	0	0	0	0	0
1	0	1	0	0	0	0	0	0
1	0	1	0	0	0	0	0	0
1	1	0	1	0	1	0	0	0
1	0	1	0	0	0	0	0	0
1	0	1	0	1	0	1	0	0
0	0	1	0	0	0	0	0	0
1	0	1	0	0	0	0	0	0
1	0	0	0	0	0	0	0	0
1	0	1	0	0	0	0	0	0
1	0	1	1	1	0	0	0	0
0	0	0	1	0	0	0	0	0
1	0	1	0	0	0	0	0	0
1	0	1	0	1	0	1	0	0
1	0	1	0	0	0	0	0	0
1	0	0	1	1	1	0	0	0
1	1	1	1	0	0	0	0	0
1	0	1	0	0	0	0	0	0
1	0	0	0	0	0	0	0	0
1	0	1	1	1	0	1	0	0
0	0	1	0	0	0	0	0	0
1	0	1	1	0	0	0	0	0
1	1	1	1	0	0	0	0	0
1	0	1	0	1	0	0	0	0
1	0	1	0	0	0	0	0	0

1	0	1	0	0	0	0	0	0
1	0	1	0	0	0	0	0	0
0	0	1	1	0	0	0	0	0
1	0	1	0	0	0	0	0	0
1	1	1	0	0	0	0	0	0
1	0	1	1	1	0	0	0	0
0	0	1	0	0	0	0	0	0
1	0	1	0	0	0	0	0	0
1	0	1	0	0	0	1	0	0
1	0	1	0	0	0	1	0	0
1	0	1	1	0	0	1	0	0
1	0	1	1	1	1	1	1	0
1	1	1	1	0	0	1	0	0
1	1	1	1	1	0	1	0	0
1	0	1	1	1	0	0	1	0
0	0	1	1	0	0	0	0	0
1	0	1	1	1	0	1	0	0
1	0	1	0	0	0	0	0	0
1	0	1	0	0	0	1	0	0
1	1	0	0	1	0	0	0	0
1	0	1	0	0	0	0	0	0
0	0	1	0	0	0	0	0	0
1	0	1	0	1	0	0	0	0
0	0	0	0	0	0	0	0	0
0	0	1	0	0	0	0	0	0
1	0	1	0	1	1	0	1	0
1	0	1	0	0	0	0	0	0
1	0	1	1	1	0	0	0	0
1	0	1	0	0	0	0	0	0
1	1	1	1	1	1	0	1	0
1	1	1	1	1	1	0	1	0
1	0	1	1	0	0	0	0	0
1	0	1	1	0	0	0	1	0
1	0	1	1	1	1	0	1	0
1	1	1	1	1	1	0	0	0
1	0	1	0	0	0	0	0	0
1	1	1	1	1	1	1	1	1
1	1	1	1	1	1	0	1	0
1	0	1	0	0	0	0	0	0
1	1	1	1	1	1	0	1	1
1	0	1	1	1	1	1	0	0
1	0	1	1	1	1	0	1	1
1	0	0	0	0	0	0	0	0
1	1	1	1	1	1	0	1	0
0	0	1	1	0	0	0	0	0
1	0	1	1	1	0	0	0	0
0	0	1	1	0	1	0	1	0

1	1	1	1	1	1	1	1	1
1	1	1	1	1	1	0	1	0
1	0	0	1	1	1	0	1	0
1	1	1	1	1	1	0	1	0
1	0	1	1	1	0	0	1	1
1	0	1	1	1	1	0	0	0
1	1	1	0	0	0	0	0	0
1	1	1	0	0	0	0	0	0
1	1	1	1	1	1	0	1	0
1	1	1	1	1	1	0	1	0
1	0	0	1	1	0	0	1	0
1	1	1	1	1	1	0	1	0
1	1	1	1	1	1	1	1	0
1	0	1	1	1	0	1	0	0

UniqueKey	RECSTATUS	C1DELIVER	ESTIAGEOF	AGEMONTI	BIRTHWEIG	BFDURATIC	MOTERNAI	ANCATTEN
297	1	3	2.00	3.00	2.00	3.00	2.00	2.00
298	1	1	2.00	2.00	2.00	3.00	2.00	2.00
299	1	1	1.00	3.00	2.00	2.00	2.00	2.00
300	1	2	1.00	2.00	2.00	2.00	2.00	1.00
301	1	2	2.00	1.00	2.00	2.00	2.00	2.00
322	1	1	2.00	2.00	2.00	2.00	2.00	1.00
302	1	1	1.00	3.00	2.00	2.00	2.00	2.00
303	1	1	1.00	3.00	2.00	2.00	2.00	2.00
304	1	1	2.00	1.00	2.00	2.00	2.00	2.00
305	1	1	1.00	2.00	2.00	2.00	2.00	2.00
306	1	3	1.00	1.00	1.00	2.00	2.00	2.00
307	1	1	2.00	1.00	2.00	2.00	1.00	2.00
308	1	1	1.00	1.00	2.00	2.00	1.00	2.00
309	1	1	1.00	2.00	2.00	2.00	2.00	2.00
310	1	2	2.00	1.00	1.00	2.00	2.00	2.00
311	1	1	1.00	1.00	2.00	2.00	2.00	2.00
312	1	2	2.00	2.00	2.00	2.00	1.00	2.00
313	1	2	1.00	2.00	2.00	2.00	1.00	2.00
314	1	3	1.00	1.00	2.00	2.00	2.00	1.00
315	1	2	2.00	1.00	2.00	2.00	2.00	2.00
316	1	1	2.00	1.00	2.00	3.00	1.00	2.00
317	1	1	1.00	2.00	2.00	2.00	1.00	2.00
318	1	3	2.00	2.00	2.00	3.00	2.00	1.00
319	1	1	1.00	3.00	2.00	2.00	3.00	2.00
320	1	2	2.00	3.00	2.00	2.00	2.00	2.00
321	1	3	2.00	2.00	2.00	3.00	2.00	2.00
323	1	4	2.00	1.00	2.00	2.00	3.00	2.00
324	1	1	1.00	2.00	2.00	2.00	2.00	2.00
325	1	1	2.00	3.00	2.00	2.00	2.00	2.00
326	1	1	1.00	2.00	2.00	2.00	2.00	2.00
327	1	1	1.00	2.00	2.00	2.00	1.00	2.00
328	1	1	1.00	2.00	2.00	2.00	2.00	2.00
329	1	1	2.00	2.00	2.00	3.00	2.00	2.00
330	1	1	2.00	2.00	1.00	3.00	2.00	2.00
331	1	3	1.00	2.00	2.00	2.00	2.00	2.00
332	1	1	1.00	2.00	2.00	2.00	2.00	2.00
333	1	1	2.00	3.00	2.00	3.00	2.00	2.00
334	1	1	2.00	3.00	2.00	2.00	1.00	2.00
335	1	1	1.00	2.00	2.00	2.00	1.00	2.00
336	1	4	1.00	2.00	2.00	2.00	2.00	2.00
337	1	2	2.00	3.00	2.00	3.00	2.00	2.00
338	1	1	1.00	3.00	1.00	2.00	2.00	2.00
339	1	1	2.00	3.00	1.00	2.00	2.00	2.00
340	1	4	2.00	3.00	2.00	2.00	2.00	2.00
341	1	2	2.00	3.00	2.00	3.00	2.00	2.00
342	1	3	2.00	3.00	2.00	2.00	2.00	2.00

282	1	2	2.00	3.00	2.00	3.00	2.00	2.00
343	1	1	1.00	2.00	2.00	2.00	1.00	2.00
344	1	3	2.00	1.00	2.00	2.00	2.00	2.00
345	1	1	2.00	2.00	2.00	2.00	1.00	2.00
346	1	1	1.00	2.00	2.00	2.00	2.00	2.00
281	1	1	2.00	1.00	2.00	2.00	1.00	2.00
347	1	3	1.00	2.00	2.00	2.00	3.00	2.00
373	1	2	2.00	3.00	2.00	2.00	1.00	2.00
374	1	3	2.00	3.00	2.00	3.00	1.00	2.00
375	1	4	1.00	1.00	2.00	2.00	2.00	2.00
376	1	1	1.00	2.00	2.00	2.00	1.00	1.00
377	1	1	1.00	3.00	2.00	2.00	1.00	1.00
378	1	2	2.00	1.00	2.00	3.00	3.00	1.00
379	1	1	2.00	3.00	2.00	3.00	1.00	1.00
380	1	1	1.00	1.00	1.00	2.00	1.00	1.00
381	1	4	2.00	3.00	2.00	3.00	2.00	2.00
382	1	1	1.00	1.00	2.00	2.00	1.00	1.00
383	1	3	2.00	3.00	2.00	2.00	2.00	2.00
384	1	2	2.00	3.00	2.00	3.00	2.00	2.00
385	1	3	1.00	2.00	1.00	2.00	2.00	2.00
386	1	4	2.00	1.00	2.00	3.00	2.00	2.00
387	1	4	2.00	3.00	2.00	2.00	3.00	2.00
388	1	2	1.00	2.00	2.00	2.00	1.00	2.00
389	1	3	2.00	1.00	2.00	2.00	2.00	2.00
390	1	4	1.00	3.00	2.00	2.00	2.00	2.00
391	1	2	1.00	2.00	2.00	2.00	3.00	2.00
392	1	4	2.00	2.00	2.00	2.00	2.00	2.00
393	1	2	2.00	2.00	2.00	2.00	1.00	2.00
394	1	1	1.00	1.00	2.00	2.00	1.00	2.00
395	1	4	2.00	3.00	2.00	2.00	2.00	2.00
396	1	1	1.00	2.00	2.00	2.00	2.00	1.00
397	1	3	2.00	3.00	2.00	3.00	2.00	2.00
398	1	3	1.00	1.00	2.00	2.00	2.00	2.00
399	1	2	2.00	2.00	2.00	3.00	2.00	2.00
400	1	3	2.00	3.00	2.00	3.00	2.00	2.00
401	1	4	1.00	2.00	2.00	2.00	3.00	2.00
402	1	4	1.00	2.00	2.00	2.00	3.00	2.00
403	1	3	2.00	3.00	2.00	2.00	2.00	2.00
404	1	4	1.00	2.00	1.00	2.00	2.00	1.00
405	1	2	2.00	3.00	2.00	2.00	2.00	2.00
406	1	4	2.00	3.00	2.00	2.00	2.00	2.00
407	1	1	1.00	3.00	1.00	2.00	2.00	2.00
408	1	2	2.00	1.00	2.00	2.00	2.00	2.00
409	1	1	1.00	3.00	2.00	2.00	1.00	1.00
410	1	2	2.00	2.00	2.00	2.00	2.00	2.00
411	1	1	1.00	1.00	2.00	2.00	2.00	1.00
412	1	1	1.00	1.00	2.00	2.00	3.00	2.00

413	1	4	2.00	3.00	2.00	2.00	3.00	1.00
414	1	4	1.00	2.00	2.00	2.00	2.00	2.00
1	1	1	2.00	1.00	2.00	3.00	1.00	2.00
106	1	4	1.00	1.00	2.00	2.00	2.00	2.00
2	1	4	2.00	3.00	2.00	2.00	2.00	2.00
3	1	3	1.00	2.00	2.00	2.00	2.00	1.00
4	1	2	2.00	3.00	2.00	3.00	2.00	1.00
5	1	1	1.00	1.00	2.00	2.00	1.00	2.00
6	1	3	2.00	2.00	2.00	2.00	2.00	2.00
7	1	2	1.00	3.00	2.00	2.00	2.00	1.00
8	1	1	2.00	2.00	2.00	3.00	1.00	2.00
9	1	3	1.00	1.00	2.00	2.00	2.00	2.00
10	1	4	2.00	3.00	2.00	3.00	2.00	2.00
11	1	4	2.00	2.00	2.00	3.00	2.00	2.00
12	1	1	2.00	3.00	2.00	3.00	2.00	2.00
13	1	2	1.00	1.00	2.00	2.00	2.00	2.00
14	1	4	1.00	1.00	2.00	2.00	3.00	2.00
15	1	4	1.00	1.00	2.00	2.00	2.00	2.00
16	1	2	2.00	3.00	2.00	2.00	1.00	2.00
17	1	2	1.00	1.00	2.00	2.00	1.00	2.00
18	1	4	2.00	3.00	1.00	2.00	1.00	2.00
19	1	1	2.00	3.00	2.00	2.00	1.00	2.00
20	1	2	1.00	1.00	2.00	2.00	2.00	2.00
21	1	2	2.00	3.00	2.00	3.00	2.00	2.00
22	1	2	2.00	3.00	2.00	2.00	2.00	2.00
23	1	1	1.00	1.00	2.00	2.00	2.00	2.00
24	1	1	2.00	1.00	1.00	2.00	1.00	2.00
25	1	4	2.00	2.00	2.00	3.00	3.00	2.00
26	1	1	1.00	1.00	2.00	2.00	2.00	2.00
27	1	2	2.00	3.00	2.00	2.00	1.00	2.00
28	1	2	1.00	2.00	2.00	2.00	2.00	2.00
29	1	1	1.00	1.00	2.00	2.00	2.00	2.00
30	1	2	2.00	1.00	2.00	3.00	2.00	2.00
31	1	4	1.00	1.00	2.00	2.00	2.00	2.00
32	1	1	2.00	3.00	2.00	2.00	2.00	2.00
33	1	1	1.00	2.00	2.00	3.00	1.00	2.00
34	1	1	1.00	1.00	2.00	2.00	1.00	2.00
35	1	2	2.00	3.00	1.00	2.00	2.00	2.00
36	1	2	1.00	2.00	2.00	2.00	2.00	2.00
37	1	1	2.00	3.00	2.00	2.00	2.00	2.00
38	1	3	1.00	2.00	2.00	2.00	2.00	2.00
39	1	4	2.00	2.00	2.00	2.00	2.00	1.00
40	1	3	1.00	1.00	2.00	2.00	2.00	2.00
41	1	3	1.00	2.00	2.00	2.00	2.00	2.00
42	1	1	2.00	2.00	2.00	2.00	1.00	2.00
43	1	3	1.00	2.00	2.00	2.00	2.00	1.00
44	1	4	1.00	2.00	2.00	2.00	2.00	2.00

45	1	3	2.00	3.00	2.00	2.00	2.00	2.00
46	1	1	1.00	2.00	1.00	2.00	2.00	2.00
47	1	3	2.00	3.00	2.00	3.00	2.00	2.00
48	1	1	2.00	1.00	2.00	2.00	1.00	1.00
49	1	4	2.00	1.00	2.00	2.00	2.00	2.00
50	1	1	1.00	2.00	2.00	2.00	2.00	2.00
51	1	4	1.00	1.00	2.00	2.00	2.00	2.00
52	1	1	2.00	2.00	2.00	2.00	1.00	2.00
53	1	1	1.00	2.00	2.00	1.00	2.00	2.00
54	1	1	2.00	3.00	2.00	2.00	2.00	1.00
55	1	1	1.00	1.00	2.00	2.00	2.00	2.00
56	1	2	2.00	1.00	2.00	2.00	2.00	2.00
57	1	4	2.00	3.00	2.00	2.00	2.00	1.00
58	1	3	1.00	2.00	2.00	2.00	2.00	2.00
59	1	3	2.00	3.00	2.00	3.00	3.00	2.00
60	1	1	1.00	2.00	2.00	2.00	2.00	2.00
61	1	4	2.00	3.00	2.00	3.00	2.00	2.00
62	1	4	1.00	2.00	2.00	2.00	2.00	2.00
63	1	1	2.00	3.00	2.00	2.00	2.00	2.00
64	1	4	1.00	2.00	2.00	2.00	2.00	2.00
65	1	1	2.00	3.00	2.00	2.00	2.00	2.00
66	1	1	1.00	1.00	2.00	2.00	2.00	1.00
67	1	4	1.00	2.00	2.00	2.00	3.00	1.00
68	1	1	2.00	3.00	2.00	3.00	1.00	2.00
69	1	3	2.00	3.00	2.00	#NULL!	3.00	2.00
70	1	1	1.00	2.00	2.00	2.00	1.00	2.00
71	1	4	2.00	2.00	2.00	3.00	2.00	1.00
72	1	1	1.00	2.00	2.00	2.00	1.00	1.00
73	1	1	2.00	2.00	2.00	2.00	1.00	2.00
74	1	3	1.00	2.00	2.00	2.00	2.00	2.00
75	1	4	2.00	3.00	2.00	3.00	2.00	2.00
76	1	1	1.00	1.00	2.00	2.00	1.00	2.00
77	1	4	2.00	3.00	2.00	3.00	2.00	2.00
78	1	1	2.00	3.00	2.00	2.00	1.00	2.00
79	1	1	1.00	2.00	2.00	2.00	1.00	2.00
80	1	1	1.00	1.00	2.00	2.00	2.00	2.00
81	1	2	1.00	1.00	2.00	2.00	1.00	2.00
82	1	2	2.00	3.00	2.00	2.00	2.00	2.00
84	1	1	1.00	2.00	2.00	2.00	2.00	2.00
85	1	4	2.00	2.00	2.00	2.00	2.00	2.00
86	1	4	1.00	2.00	2.00	2.00	2.00	2.00
87	1	1	2.00	3.00	2.00	2.00	1.00	2.00
88	1	2	1.00	2.00	2.00	2.00	1.00	2.00
89	1	3	1.00	2.00	2.00	2.00	2.00	1.00
90	1	4	2.00	3.00	2.00	#NULL!	2.00	2.00
91	1	1	2.00	2.00	2.00	3.00	2.00	2.00
92	1	3	1.00	2.00	2.00	2.00	2.00	2.00

93	1	1	2.00	3.00	2.00	2.00	2.00	2.00
105	1	2	1.00	2.00	2.00	2.00	2.00	2.00
107	1	1	1.00	2.00	2.00	2.00	1.00	1.00
108	1	3	1.00	2.00	2.00	2.00	2.00	2.00
109	1	2	2.00	2.00	1.00	2.00	2.00	2.00
110	1	1	2.00	2.00	2.00	2.00	1.00	2.00
112	1	3	1.00	2.00	2.00	2.00	2.00	2.00
113	1	1	2.00	2.00	2.00	3.00	1.00	2.00
114	1	2	2.00	1.00	2.00	3.00	2.00	2.00
115	1	4	1.00	2.00	2.00	2.00	3.00	2.00
116	1	3	2.00	3.00	1.00	2.00	2.00	2.00
117	1	1	1.00	2.00	1.00	2.00	2.00	2.00
118	1	2	2.00	3.00	2.00	2.00	1.00	2.00
119	1	1	1.00	2.00	2.00	2.00	1.00	2.00
120	1	2	2.00	2.00	2.00	2.00	2.00	2.00
121	1	3	2.00	2.00	2.00	2.00	2.00	2.00
122	1	4	1.00	2.00	2.00	2.00	2.00	2.00
445	1	2	2.00	1.00	2.00	2.00	2.00	2.00
135	1	1	1.00	1.00	2.00	2.00	3.00	2.00
136	1	4	2.00	3.00	2.00	3.00	2.00	2.00
137	1	2	2.00	3.00	2.00	2.00	2.00	2.00
138	1	2	1.00	2.00	2.00	2.00	2.00	2.00
139	1	1	2.00	3.00	2.00	3.00	2.00	2.00
140	1	4	1.00	1.00	2.00	2.00	2.00	2.00
141	1	1	2.00	2.00	2.00	2.00	2.00	2.00
142	1	3	1.00	2.00	2.00	2.00	2.00	2.00
143	1	1	1.00	1.00	1.00	2.00	2.00	1.00
144	1	2	2.00	1.00	2.00	2.00	1.00	2.00
145	1	2	1.00	2.00	2.00	2.00	2.00	2.00
146	1	2	2.00	3.00	2.00	3.00	2.00	2.00
268	1	1	1.00	2.00	2.00	2.00	1.00	2.00
148	1	2	2.00	1.00	2.00	2.00	2.00	2.00
149	1	4	1.00	1.00	2.00	3.00	2.00	2.00
150	1	2	1.00	1.00	2.00	2.00	2.00	2.00
151	1	2	2.00	3.00	2.00	2.00	2.00	2.00
152	1	2	1.00	1.00	1.00	2.00	2.00	2.00
153	1	1	2.00	1.00	2.00	2.00	2.00	2.00
154	1	1	2.00	2.00	2.00	2.00	2.00	2.00
155	1	1	1.00	2.00	2.00	2.00	2.00	2.00
156	1	4	1.00	3.00	1.00	2.00	2.00	2.00
508	1	1	2.00	3.00	2.00	3.00	1.00	2.00
157	1	1	1.00	2.00	2.00	3.00	2.00	2.00
158	1	1	1.00	2.00	2.00	2.00	1.00	2.00
159	1	1	2.00	3.00	2.00	2.00	2.00	2.00
160	1	1	1.00	3.00	2.00	2.00	1.00	2.00
161	1	1	2.00	1.00	2.00	2.00	1.00	2.00
162	1	1	2.00	1.00	2.00	2.00	1.00	2.00

163	1	1	1.00	1.00	2.00	2.00	1.00	2.00
164	1	1	1.00	1.00	2.00	2.00	2.00	2.00
165	1	2	1.00	2.00	2.00	2.00	1.00	2.00
166	1	3	2.00	3.00	1.00	3.00	2.00	2.00
167	1	4	1.00	1.00	2.00	2.00	1.00	2.00
168	1	1	2.00	1.00	2.00	3.00	1.00	2.00
169	1	1	2.00	2.00	2.00	3.00	2.00	2.00
170	1	1	1.00	2.00	1.00	2.00	1.00	2.00
171	1	4	2.00	3.00	2.00	3.00	2.00	2.00
172	1	3	1.00	3.00	2.00	2.00	2.00	2.00
173	1	2	2.00	3.00	2.00	2.00	2.00	2.00
174	1	2	1.00	2.00	2.00	2.00	2.00	2.00
175	1	2	2.00	1.00	2.00	2.00	2.00	2.00
176	1	1	1.00	2.00	2.00	2.00	1.00	2.00
177	1	2	1.00	3.00	2.00	2.00	2.00	2.00
178	1	2	2.00	1.00	2.00	2.00	2.00	2.00
179	1	1	1.00	2.00	2.00	2.00	1.00	2.00
180	1	4	2.00	3.00	1.00	#NULL!	2.00	2.00
181	1	4	1.00	2.00	2.00	2.00	2.00	2.00
182	1	3	2.00	3.00	2.00	#NULL!	3.00	2.00
183	1	4	1.00	2.00	2.00	2.00	3.00	2.00
184	1	2	1.00	2.00	2.00	2.00	2.00	2.00
185	1	3	2.00	3.00	1.00	2.00	3.00	2.00
186	1	4	2.00	2.00	2.00	#NULL!	2.00	2.00
187	1	2	1.00	2.00	1.00	3.00	2.00	2.00
188	1	3	1.00	2.00	2.00	2.00	2.00	2.00
189	1	1	2.00	2.00	2.00	2.00	1.00	1.00
190	1	2	1.00	2.00	1.00	1.00	2.00	2.00
578	1	4	2.00	1.00	2.00	2.00	2.00	2.00
577	1	2	1.00	2.00	2.00	2.00	2.00	1.00
576	1	4	2.00	3.00	2.00	2.00	3.00	2.00
575	1	1	1.00	3.00	1.00	2.00	2.00	2.00
574	1	4	2.00	1.00	2.00	2.00	2.00	2.00
200	1	1	2.00	3.00	2.00	3.00	2.00	2.00
202	1	1	1.00	2.00	2.00	2.00	1.00	2.00
204	1	2	1.00	1.00	2.00	2.00	2.00	2.00
205	1	2	2.00	3.00	2.00	3.00	2.00	2.00
206	1	2	1.00	2.00	2.00	1.00	2.00	2.00
207	1	1	1.00	1.00	2.00	2.00	1.00	2.00
208	1	2	2.00	3.00	2.00	2.00	2.00	2.00
209	1	2	2.00	3.00	2.00	2.00	1.00	2.00
210	1	1	1.00	1.00	2.00	2.00	1.00	2.00
211	1	2	2.00	2.00	2.00	2.00	2.00	1.00
212	1	2	2.00	2.00	2.00	2.00	2.00	2.00
220	1	2	1.00	2.00	2.00	2.00	2.00	2.00
213	1	2	2.00	3.00	2.00	2.00	2.00	2.00
214	1	1	2.00	3.00	2.00	2.00	2.00	2.00

215	1	1	1.00	1.00	2.00	2.00	2.00	2.00
216	1	2	2.00	1.00	2.00	2.00	2.00	2.00
217	1	2	1.00	1.00	2.00	2.00	2.00	2.00
218	1	1	2.00	2.00	2.00	2.00	2.00	2.00
219	1	1	2.00	3.00	2.00	2.00	2.00	2.00
220	1	2	1.00	2.00	2.00	2.00	2.00	2.00
221	1	2	1.00	2.00	1.00	2.00	2.00	2.00
222	1	1	2.00	1.00	2.00	2.00	1.00	2.00
223	1	1	1.00	3.00	2.00	2.00	2.00	2.00
224	1	1	2.00	1.00	2.00	2.00	1.00	2.00
225	1	1	1.00	2.00	2.00	2.00	1.00	2.00
226	1	1	2.00	3.00	2.00	2.00	2.00	2.00
227	1	1	1.00	2.00	2.00	2.00	1.00	2.00
228	1	1	2.00	3.00	2.00	2.00	1.00	2.00
229	1	4	1.00	3.00	2.00	2.00	1.00	2.00
230	1	3	2.00	3.00	2.00	2.00	2.00	2.00
231	1	2	2.00	2.00	2.00	2.00	2.00	2.00
232	1	1	1.00	1.00	2.00	2.00	2.00	2.00
233	1	3	2.00	1.00	2.00	2.00	2.00	2.00
234	1	1	2.00	2.00	2.00	2.00	1.00	2.00
235	1	1	1.00	2.00	2.00	2.00	2.00	2.00
236	1	3	2.00	3.00	2.00	2.00	2.00	2.00
237	1	1	2.00	2.00	2.00	2.00	2.00	2.00
238	1	2	1.00	1.00	2.00	3.00	2.00	2.00
239	1	2	1.00	1.00	2.00	2.00	1.00	2.00
240	1	2	2.00	1.00	2.00	2.00	2.00	2.00
241	1	1	1.00	2.00	2.00	3.00	1.00	2.00
242	1	2	2.00	2.00	2.00	3.00	2.00	2.00
243	1	3	2.00	1.00	2.00	2.00	3.00	2.00
244	1	3	2.00	3.00	2.00	2.00	1.00	1.00
245	1	3	1.00	1.00	2.00	2.00	2.00	2.00
431	1	1	1.00	1.00	2.00	2.00	2.00	2.00
432	1	1	2.00	2.00	2.00	2.00	2.00	2.00
433	1	2	2.00	3.00	2.00	2.00	2.00	2.00
434	1	2	1.00	2.00	1.00	2.00	2.00	2.00
435	1	3	2.00	2.00	2.00	3.00	2.00	2.00
436	1	2	1.00	3.00	2.00	3.00	1.00	2.00
437	1	1	1.00	2.00	2.00	2.00	2.00	2.00
438	1	4	1.00	2.00	2.00	2.00	2.00	2.00
439	1	1	2.00	3.00	2.00	2.00	2.00	2.00
440	1	2	2.00	2.00	2.00	2.00	2.00	2.00
441	1	1	1.00	2.00	2.00	2.00	2.00	2.00
442	1	3	2.00	2.00	2.00	2.00	2.00	2.00
443	1	3	2.00	2.00	2.00	2.00	2.00	2.00
444	1	2	2.00	2.00	2.00	2.00	2.00	2.00
445	1	2	1.00	1.00	2.00	2.00	2.00	2.00
446	1	4	1.00	2.00	2.00	2.00	2.00	2.00

447	1	1	2.00	3.00	2.00	2.00	2.00	2.00
448	1	2	1.00	1.00	2.00	2.00	2.00	2.00
449	1	3	2.00	2.00	2.00	2.00	2.00	2.00
450	1	1	2.00	2.00	2.00	2.00	1.00	2.00
451	1	1	1.00	3.00	2.00	2.00	1.00	2.00
452	1	1	2.00	3.00	2.00	2.00	2.00	2.00
453	1	1	2.00	3.00	2.00	2.00	2.00	2.00
454	1	1	1.00	1.00	2.00	2.00	2.00	2.00
455	1	1	2.00	3.00	2.00	2.00	2.00	2.00
456	1	1	1.00	2.00	2.00	2.00	1.00	2.00
457	1	1	1.00	2.00	2.00	2.00	2.00	2.00
458	1	2	2.00	1.00	2.00	2.00	2.00	2.00
459	1	1	1.00	2.00	2.00	2.00	2.00	2.00
460	1	3	1.00	2.00	2.00	2.00	2.00	2.00
461	1	4	1.00	3.00	2.00	2.00	2.00	2.00
462	1	1	2.00	3.00	2.00	3.00	2.00	2.00
463	1	4	2.00	1.00	2.00	3.00	2.00	2.00
464	1	2	1.00	1.00	2.00	2.00	2.00	2.00
465	1	2	2.00	2.00	2.00	2.00	2.00	2.00
466	1	3	2.00	3.00	2.00	2.00	2.00	2.00
467	1	1	1.00	2.00	2.00	2.00	2.00	2.00
246	1	1	2.00	3.00	2.00	2.00	2.00	2.00
247	1	1	2.00	3.00	2.00	2.00	2.00	2.00
248	1	1	1.00	1.00	2.00	2.00	2.00	2.00
249	1	1	1.00	2.00	2.00	2.00	2.00	2.00
250	1	3	2.00	2.00	2.00	2.00	2.00	2.00
251	1	3	2.00	1.00	2.00	2.00	2.00	2.00
252	1	4	1.00	1.00	2.00	2.00	3.00	2.00
253	1	1	2.00	1.00	2.00	2.00	2.00	2.00
254	1	1	1.00	1.00	1.00	2.00	2.00	2.00
255	1	2	1.00	3.00	2.00	2.00	1.00	2.00
256	1	1	1.00	2.00	2.00	2.00	2.00	2.00
257	1	1	2.00	3.00	2.00	2.00	2.00	1.00
258	1	4	2.00	1.00	2.00	2.00	2.00	2.00
259	1	4	2.00	1.00	2.00	2.00	2.00	2.00
260	1	1	1.00	3.00	2.00	2.00	2.00	2.00
261	1	1	1.00	1.00	2.00	2.00	2.00	2.00
262	1	1	1.00	1.00	2.00	2.00	2.00	2.00
263	1	3	1.00	1.00	1.00	2.00	2.00	2.00
264	1	1	1.00	1.00	2.00	2.00	2.00	2.00
265	1	3	2.00	3.00	2.00	3.00	3.00	2.00
266	1	1	1.00	2.00	2.00	2.00	2.00	2.00
267	1	1	2.00	2.00	2.00	2.00	2.00	2.00
268	1	1	2.00	2.00	2.00	2.00	1.00	2.00
269	1	1	1.00	1.00	2.00	2.00	1.00	2.00
270	1	1	1.00	2.00	2.00	2.00	3.00	2.00
271	1	1	1.00	2.00	2.00	2.00	2.00	2.00

272	1	1	2.00	3.00	2.00	2.00	2.00	2.00
273	1	1	2.00	3.00	2.00	2.00	2.00	2.00
274	1	2	1.00	2.00	2.00	2.00	1.00	2.00
275	1	2	2.00	3.00	2.00	2.00	2.00	2.00
276	1	2	1.00	1.00	2.00	2.00	1.00	2.00
277	1	2	2.00	3.00	2.00	1.00	1.00	2.00
278	1	2	1.00	2.00	2.00	2.00	2.00	2.00
279	1	4	2.00	3.00	2.00	3.00	3.00	2.00
468	1	3	1.00	2.00	1.00	#NULL!	2.00	2.00
469	1	4	2.00	1.00	2.00	2.00	2.00	2.00
470	1	4	1.00	1.00	2.00	2.00	3.00	2.00
471	1	3	2.00	1.00	2.00	3.00	3.00	2.00
472	1	2	1.00	2.00	2.00	2.00	2.00	2.00
473	1	2	2.00	2.00	1.00	2.00	2.00	2.00
474	1	2	1.00	1.00	2.00	2.00	2.00	2.00
475	1	1	2.00	3.00	2.00	3.00	2.00	2.00
476	1	4	1.00	2.00	2.00	2.00	2.00	2.00
477	1	2	1.00	2.00	2.00	2.00	2.00	2.00
478	1	3	1.00	2.00	2.00	2.00	2.00	2.00
479	1	1	2.00	2.00	2.00	2.00	2.00	2.00
480	1	2	1.00	2.00	2.00	2.00	2.00	2.00
481	1	2	2.00	3.00	1.00	2.00	2.00	2.00
482	1	1	1.00	2.00	2.00	2.00	1.00	2.00
483	1	1	1.00	1.00	1.00	2.00	2.00	2.00
484	1	4	2.00	3.00	2.00	3.00	2.00	2.00
485	1	1	1.00	2.00	2.00	2.00	1.00	2.00
486	1	2	1.00	2.00	2.00	2.00	2.00	2.00
487	1	1	1.00	1.00	2.00	2.00	2.00	2.00
488	1	1	2.00	1.00	2.00	2.00	1.00	2.00
489	1	4	2.00	3.00	2.00	2.00	2.00	2.00
490	1	1	1.00	2.00	2.00	3.00	2.00	2.00
491	1	1	1.00	3.00	2.00	2.00	2.00	2.00
492	1	1	2.00	1.00	2.00	2.00	1.00	2.00
493	1	2	2.00	3.00	2.00	2.00	1.00	2.00
494	1	4	1.00	1.00	2.00	2.00	3.00	2.00
495	1	1	1.00	1.00	2.00	2.00	1.00	2.00
496	1	1	2.00	3.00	2.00	2.00	1.00	2.00
497	1	3	1.00	1.00	2.00	2.00	2.00	2.00
498	1	4	2.00	1.00	2.00	2.00	2.00	2.00
499	1	1	1.00	1.00	2.00	2.00	1.00	2.00
500	1	2	2.00	3.00	2.00	2.00	2.00	2.00
501	1	3	1.00	2.00	2.00	2.00	2.00	2.00
502	1	1	1.00	2.00	2.00	2.00	1.00	2.00
503	1	4	2.00	3.00	1.00	2.00	2.00	2.00
504	1	1	2.00	2.00	2.00	2.00	1.00	2.00
505	1	4	1.00	3.00	1.00	2.00	2.00	2.00
506	1	3	2.00	2.00	2.00	3.00	2.00	2.00

507	1	1	1.00	2.00	2.00	3.00	2.00	2.00
509	1	1	2.00	3.00	2.00	2.00	2.00	2.00
510	1	3	1.00	2.00	2.00	2.00	2.00	2.00
511	1	3	2.00	2.00	2.00	2.00	2.00	2.00
512	1	1	2.00	3.00	2.00	#NULL!	2.00	2.00
513	1	3	1.00	1.00	2.00	2.00	2.00	2.00
514	1	4	1.00	2.00	2.00	2.00	2.00	2.00
515	1	4	2.00	3.00	2.00	#NULL!	2.00	2.00
516	1	1	1.00	1.00	2.00	2.00	2.00	2.00
517	1	3	2.00	2.00	2.00	2.00	2.00	2.00
518	1	2	2.00	3.00	2.00	2.00	1.00	2.00
519	1	2	2.00	2.00	2.00	2.00	2.00	2.00
520	1	2	1.00	2.00	2.00	2.00	2.00	2.00
521	1	4	1.00	2.00	2.00	2.00	3.00	2.00
522	1	2	2.00	1.00	2.00	2.00	1.00	2.00
523	1	1	2.00	2.00	2.00	2.00	1.00	2.00
524	1	3	2.00	1.00	2.00	3.00	2.00	2.00
525	1	3	1.00	2.00	2.00	2.00	2.00	2.00
526	1	1	1.00	1.00	2.00	2.00	1.00	2.00
527	1	1	2.00	1.00	2.00	3.00	1.00	2.00
528	1	1	1.00	2.00	2.00	2.00	2.00	2.00
529	1	2	2.00	3.00	2.00	2.00	2.00	2.00
530	1	3	2.00	3.00	2.00	#NULL!	2.00	2.00
531	1	1	1.00	1.00	2.00	2.00	1.00	2.00
532	1	3	2.00	1.00	2.00	3.00	2.00	2.00
533	1	3	1.00	1.00	1.00	2.00	2.00	2.00
534	1	1	1.00	2.00	2.00	2.00	2.00	2.00
535	1	2	2.00	1.00	2.00	2.00	1.00	2.00
536	1	1	2.00	3.00	2.00	2.00	2.00	2.00
537	1	2	1.00	1.00	2.00	2.00	1.00	2.00
538	1	3	1.00	2.00	2.00	2.00	2.00	2.00
539	1	1	2.00	3.00	1.00	2.00	2.00	2.00
540	1	3	2.00	3.00	2.00	2.00	2.00	2.00
541	1	4	1.00	2.00	2.00	2.00	2.00	2.00
542	1	4	2.00	3.00	2.00	2.00	1.00	2.00
543	1	2	2.00	3.00	2.00	2.00	1.00	2.00
544	1	4	1.00	2.00	2.00	3.00	3.00	2.00
545	1	1	2.00	3.00	2.00	2.00	1.00	2.00
546	1	2	1.00	1.00	2.00	2.00	2.00	2.00
547	1	3	2.00	3.00	2.00	3.00	2.00	2.00
548	1	4	1.00	2.00	2.00	2.00	2.00	2.00
549	1	4	1.00	2.00	2.00	2.00	3.00	2.00
550	1	4	2.00	3.00	2.00	2.00	2.00	2.00
551	1	1	1.00	1.00	2.00	2.00	2.00	2.00
552	1	3	2.00	1.00	2.00	2.00	2.00	2.00
553	1	4	1.00	1.00	2.00	2.00	2.00	2.00
554	1	2	2.00	3.00	2.00	3.00	2.00	2.00

555	1	3	2.00	3.00	2.00	3.00	2.00	2.00
556	1	4	1.00	2.00	2.00	2.00	2.00	2.00
557	1	3	1.00	2.00	2.00	2.00	2.00	2.00
558	1	2	1.00	1.00	2.00	2.00	2.00	2.00
559	1	1	2.00	3.00	2.00	#NULL!	2.00	2.00
560	1	2	1.00	2.00	2.00	#NULL!	1.00	2.00
561	1	3	2.00	3.00	2.00	3.00	2.00	2.00
562	1	2	1.00	2.00	2.00	2.00	2.00	1.00
573	1	2	2.00	3.00	2.00	2.00	3.00	2.00
563	1	2	1.00	2.00	2.00	2.00	1.00	2.00
565	1	4	2.00	3.00	2.00	#NULL!	2.00	2.00
566	1	2	1.00	1.00	2.00	3.00	2.00	2.00
567	1	3	1.00	2.00	2.00	2.00	2.00	1.00
568	1	2	2.00	1.00	2.00	2.00	3.00	2.00
569	1	2	1.00	1.00	2.00	2.00	2.00	2.00
570	1	4	2.00	3.00	2.00	2.00	2.00	2.00
571	1	2	1.00	2.00	2.00	2.00	2.00	2.00
572	1	4	2.00	1.00	2.00	3.00	2.00	2.00
283	1	3	2.00	2.00	2.00	2.00	1.00	2.00
284	1	4	1.00	2.00	2.00	2.00	2.00	2.00
285	1	3	1.00	1.00	2.00	2.00	1.00	2.00
286	1	4	2.00	3.00	2.00	2.00	3.00	2.00
287	1	4	2.00	3.00	2.00	2.00	2.00	2.00
288	1	1	1.00	2.00	2.00	2.00	1.00	2.00
289	1	4	2.00	3.00	2.00	2.00	3.00	2.00
290	1	1	1.00	3.00	1.00	2.00	2.00	2.00
291	1	4	2.00	3.00	2.00	2.00	3.00	1.00
292	1	2	1.00	2.00	2.00	2.00	2.00	1.00
293	1	4	1.00	2.00	2.00	2.00	3.00	2.00
294	1	2	2.00	3.00	2.00	2.00	2.00	2.00
295	1	3	2.00	3.00	2.00	2.00	2.00	2.00
296	1	3	1.00	2.00	2.00	2.00	2.00	2.00
415	1	1	2.00	3.00	2.00	2.00	1.00	2.00
416	1	1	1.00	2.00	2.00	2.00	2.00	2.00
417	1	1	1.00	1.00	2.00	2.00	2.00	2.00
418	1	2	2.00	1.00	2.00	2.00	1.00	2.00
419	1	2	2.00	1.00	1.00	2.00	2.00	2.00
420	1	1	1.00	3.00	1.00	2.00	2.00	2.00
421	1	1	2.00	3.00	2.00	2.00	2.00	2.00
422	1	1	1.00	1.00	2.00	2.00	2.00	2.00
423	1	2	2.00	1.00	2.00	2.00	2.00	2.00
424	1	1	1.00	2.00	2.00	2.00	2.00	2.00
425	1	1	1.00	1.00	2.00	2.00	2.00	2.00
426	1	1	1.00	2.00	2.00	2.00	2.00	2.00
427	1	1	1.00	1.00	2.00	2.00	2.00	2.00
428	1	2	1.00	1.00	2.00	2.00	2.00	2.00
429	1	4	2.00	2.00	2.00	2.00	2.00	2.00

430	1	1	1.00	1.00	2.00	2.00	2.00	2.00
94	1	1	2.00	1.00	1.00	2.00	2.00	2.00
95	1	1	1.00	1.00	2.00	2.00	1.00	2.00
96	1	1	2.00	1.00	2.00	2.00	2.00	2.00
97	1	3	1.00	3.00	2.00	2.00	2.00	2.00
98	1	2	2.00	3.00	2.00	2.00	2.00	2.00
99	1	1	1.00	2.00	1.00	2.00	2.00	2.00
100	1	1	2.00	2.00	2.00	2.00	2.00	2.00
101	1	4	1.00	1.00	2.00	2.00	2.00	2.00
102	1	1	2.00	2.00	2.00	2.00	2.00	2.00
103	1	2	2.00	1.00	2.00	2.00	2.00	2.00
104	1	2	1.00	1.00	2.00	2.00	2.00	2.00
#NULL!	1	2	1.00	1.00	2.00	2.00	2.00	1.00
114	1	2	2.00	1.00	2.00	3.00	2.00	2.00

BIRTHSPAC	EDUCATIOI	MATERNAL	MATERNAL	MONTHS	WEIGHT	HEIGHT	WAZNCHS	HAZNCHS
2.00	1.00	3.00	2.00	21	12.90	88.0	1.2740	1.3180
2.00	1.00	2.00	1.00	11	9.10	72.2	-0.1390	-0.3080
1.00	2.00	3.00	2.00	21	10.40	86.5	-0.8040	0.8470
1.00	1.00	3.00	2.00	8	7.50	70.3	-1.3290	-0.2510
2.00	1.00	3.00	2.00	2	5.20	59.6	0.0110	0.5890
2.00	1.00	2.00	1.00	6	8.90	70.0	1.0850	0.8120
1.00	2.00	2.00	1.00	12	8.90	78.9	-0.6020	1.6230
1.00	2.00	3.00	2.00	13	8.20	74.0	-1.4840	-0.5220
2.00	2.00	2.00	1.00	4	7.40	65.5	0.7830	0.6740
1.00	2.00	2.00	1.00	7	7.20	68.0	-1.1780	-0.5560
1.00	1.00	3.00	2.00	4	7.10	61.5	0.4570	-0.8180
2.00	1.00	1.00	1.00	4	8.30	68.2	1.7630	1.6800
1.00	1.00	1.00	1.00	5	7.40	64.2	0.1090	-0.6350
1.00	1.00	2.00	1.00	8	6.90	67.9	-1.9510	-1.1560
2.00	1.00	2.00	1.00	3	4.10	54.9	-1.9780	-2.3480
1.00	1.00	2.00	1.00	5	6.20	64.1	-0.5430	0.0070
2.00	1.00	1.00	1.00	6	8.20	64.6	0.3650	-1.1990
1.00	1.00	1.00	1.00	11	6.60	62.8	-2.5840	-3.6680
1.00	2.00	3.00	2.00	3	6.70	59.2	0.8260	-0.7210
2.00	1.00	2.00	1.00	3	6.70	61.2	0.8260	0.0360
2.00	1.00	1.00	1.00	5	7.30	66.0	0.7280	0.7370
1.00	1.00	1.00	1.00	8	7.50	66.0	-0.7030	-1.1330
2.00	1.00	3.00	2.00	8	9.70	73.3	0.9030	0.8790
1.00	2.00	4.00	2.00	12	8.90	83.7	-1.2320	2.8240
2.00	1.00	2.00	1.00	19	9.30	75.0	-1.4430	-2.2240
2.00	1.00	3.00	2.00	7	7.30	62.2	-0.4520	-2.0100
2.00	1.00	4.00	2.00	4	7.30	65.3	1.4860	1.3050
1.00	1.00	2.00	1.00	9	9.00	76.4	-0.1870	1.5380
2.00	1.00	2.00	1.00	13	10.50	79.9	0.0770	0.9720
1.00	1.00	3.00	2.00	8	9.70	71.7	1.5900	0.9750
1.00	1.00	1.00	1.00	8	7.70	69.4	-1.1220	-0.5910
2.00	1.00	2.00	1.00	7	8.20	67.9	-0.1440	-0.5930
2.00	1.00	3.00	2.00	11	9.40	74.8	-0.4630	-0.0310
2.00	1.00	2.00	1.00	11	8.00	65.4	-1.8700	-3.5590
1.00	1.00	3.00	2.00	11	9.30	68.3	0.0560	-1.7020
1.00	2.00	3.00	2.00	6	9.20	70.4	1.3940	0.9610
2.00	2.00	2.00	1.00	20	13.50	78.0	1.3180	-1.9720
1.00	1.00	1.00	1.00	18	10.90	78.0	0.0660	-0.9530
1.00	1.00	1.00	1.00	11	7.50	73.0	-2.3720	-0.7060
1.00	1.00	3.00	2.00	10	8.00	76.0	-1.5710	0.8940
2.00	1.00	3.00	2.00	24	12.00	87.0	-0.3040	0.4420
1.00	1.00	2.00	1.00	12	9.50	74.2	-0.6410	-0.6990
2.00	1.00	2.00	1.00	12	9.30	75.0	-0.8380	-0.4020
2.00	1.00	3.00	2.00	23	9.90	75.9	-1.4900	-2.9890
2.00	1.00	2.00	1.00	24	12.00	84.3	-0.3040	-0.4060
2.00	1.00	3.00	2.00	13	12.00	84.3	1.4020	2.5790

2.00	1.00	2.00	1.00	24	13.10	75.6	0.4530	-3.1370
1.00	1.00	1.00	1.00	7	7.40	68.2	-0.9710	-0.4810
2.00	1.00	2.00	1.00	5	5.50	69.3	-1.8170	1.2580
2.00	1.00	1.00	1.00	6	6.90	69.3	-0.3540	1.2740
1.00	1.00	3.00	2.00	6	6.20	66.4	-1.1580	0.1770
2.00	1.00	1.00	1.00	3	6.80	65.7	1.8060	2.4760
1.00	1.00	4.00	2.00	11	7.00	67.5	-2.1920	-1.9880
2.00	1.00	1.00	1.00	19	10.00	81.0	-1.3750	-0.7540
2.00	1.00	1.00	1.00	24	10.00	86.0	-2.0830	0.1280
1.00	1.00	3.00	2.00	5	9.50	69.0	3.2090	1.8900
1.00	1.00	1.00	1.00	10	9.50	69.0	-0.0400	-1.7510
2.00	1.00	1.00	1.00	14	8.50	70.9	-2.0240	-2.6730
2.00	1.00	4.00	2.00	2	4.50	60.0	-0.8050	0.7450
2.00	1.00	1.00	1.00	20	10.30	79.0	-0.7440	-1.2310
1.00	1.00	1.00	1.00	4	6.50	65.0	0.5350	1.1870
2.00	1.00	3.00	2.00	24	12.00	76.0	-0.3040	-3.0110
1.00	1.00	1.00	1.00	2	5.50	59.6	1.1640	1.1840
2.00	1.00	3.00	2.00	13	9.00	68.0	-0.7380	-2.6120
2.00	1.00	2.00	1.00	21	10.00	83.3	-1.1350	-0.1590
1.00	1.00	3.00	2.00	6	6.50	70.2	-1.3790	0.8860
2.00	1.00	3.00	2.00	5	6.00	68.0	-1.3110	0.7760
2.00	1.00	4.00	2.00	22	10.60	87.6	-1.2630	0.4930
1.00	1.00	1.00	1.00	10	10.20	82.6	1.2690	3.9140
2.00	1.00	2.00	1.00	5	6.60	70.9	-0.7050	1.8520
1.00	1.00	3.00	2.00	12	9.00	81.3	-1.1340	1.9340
1.00	1.00	4.00	2.00	7	7.40	68.0	-0.9710	-0.5560
2.00	1.00	3.00	2.00	8	7.00	69.9	-1.8480	-0.4020
2.00	2.00	1.00	1.00	11	7.40	74.3	-1.8010	0.4420
1.00	1.00	1.00	1.00	4	7.10	66.0	1.2480	1.5790
2.00	1.00	3.00	2.00	15	10.70	81.0	0.4200	1.0940
1.00	1.00	2.00	1.00	6	10.10	76.0	3.1450	3.8090
2.00	1.00	3.00	2.00	18	8.60	77.0	-1.9020	-1.2790
1.00	1.00	3.00	2.00	3	6.00	67.2	0.0190	2.3040
2.00	1.00	3.00	2.00	11	8.80	74.0	-0.4330	0.3350
2.00	1.00	3.00	2.00	17	9.50	78.0	-0.9870	-0.6300
1.00	1.00	4.00	2.00	11	8.30	73.0	-1.5680	-0.7060
2.00	1.00	4.00	2.00	10	9.30	75.0	-0.2440	0.5160
2.00	2.00	3.00	2.00	15	8.40	69.0	-2.2670	-3.6640
1.00	1.00	3.00	2.00	7	8.00	70.7	0.3080	1.1680
2.00	2.00	3.00	2.00	24	13.00	78.0	0.3930	-2.3840
2.00	1.00	3.00	2.00	19	8.00	70.0	-2.5410	-3.8330
1.00	1.00	2.00	1.00	14	7.30	70.1	-2.4890	-2.2530
2.00	1.00	2.00	1.00	5	7.70	67.2	0.4250	0.4790
1.00	1.00	1.00	1.00	12	8.00	80.1	-1.4600	2.0460
2.00	1.00	3.00	2.00	11	8.20	72.1	-1.0190	-0.3440
1.00	1.00	2.00	1.00	3	5.10	63.6	-0.9280	0.9430
1.00	2.00	4.00	2.00	5	6.40	70.2	-0.9070	1.5930

2.00	1.00	4.00	2.00	23	9.00	81.3	-2.6040	-1.6600
1.00	1.00	2.00	1.00	8	8.00	74.2	-0.1680	1.8990
2.00	2.00	1.00	1.00	5	5.90	69.0	-1.4120	1.1470
1.00	1.00	3.00	2.00	3	6.70	65.6	0.8260	1.6990
2.00	1.00	3.00	2.00	17	8.70	69.6	-2.2420	-3.9880
1.00	1.00	2.00	1.00	6	6.00	64.1	-1.3880	-0.6940
2.00	1.00	3.00	2.00	12	8.30	73.0	-1.1740	-0.4590
1.00	1.00	1.00	1.00	4	8.30	72.5	1.7630	3.2830
2.00	1.00	3.00	2.00	10	7.50	66.2	-2.0810	-2.8090
1.00	1.00	2.00	1.00	12	8.20	78.9	-1.2700	1.6230
2.00	1.00	1.00	1.00	9	7.80	71.3	-1.4250	-0.3910
1.00	1.00	3.00	2.00	3	7.80	71.2	2.0930	3.8170
2.00	1.00	3.00	2.00	19	9.60	85.5	-1.7060	0.6960
2.00	1.00	3.00	2.00	8	8.40	73.0	0.2500	1.4560
2.00	1.00	2.00	1.00	24	14.00	90.3	1.5690	1.7950
1.00	1.00	2.00	1.00	4	6.90	66.3	1.0110	1.6970
1.00	1.00	4.00	2.00	4	5.50	60.0	-0.6910	-0.7740
1.00	1.00	3.00	2.00	4	6.80	68.0	0.8920	2.3630
2.00	1.00	1.00	1.00	16	9.00	81.2	-1.8570	0.2590
1.00	1.00	1.00	1.00	3	5.30	55.9	-0.1330	-1.4690
2.00	1.00	1.00	1.00	20	11.40	76.8	-0.3630	-2.3510
2.00	1.00	1.00	1.00	24	9.30	70.3	-2.1270	-4.4280
1.00	1.00	3.00	2.00	3	5.20	63.4	-0.8230	0.8680
2.00	1.00	2.00	1.00	23	12.10	88.5	0.2970	0.8910
2.00	1.00	2.00	1.00	23	10.00	85.1	-1.4090	-0.1550
1.00	1.00	3.00	2.00	5	6.40	65.4	-0.3040	0.5060
2.00	1.00	1.00	1.00	5	6.10	66.1	-0.6630	0.7750
2.00	1.00	4.00	2.00	11	9.70	77.6	0.4460	1.6220
1.00	1.00	2.00	1.00	4	6.10	60.8	-0.5870	-1.0790
2.00	1.00	1.00	1.00	19	9.50	74.2	-1.7890	-2.9460
1.00	1.00	2.00	1.00	7	9.00	70.0	1.3640	0.9060
1.00	1.00	2.00	1.00	3	6.00	61.2	0.7750	0.6650
2.00	1.00	2.00	1.00	2	4.80	59.2	0.1340	1.0180
1.00	1.00	3.00	2.00	5	7.80	73.0	0.5300	2.6320
2.00	2.00	3.00	2.00	19	10.10	76.1	-1.2920	-2.3330
1.00	1.00	1.00	1.00	6	6.80	68.6	-1.0720	0.2910
1.00	1.00	1.00	1.00	2	6.00	61.5	1.0260	1.3280
2.00	1.00	3.00	2.00	17	9.40	80.0	-1.6330	-0.4860
1.00	1.00	3.00	2.00	8	6.90	69.6	-1.3440	0.1990
2.00	2.00	2.00	1.00	16	8.00	78.0	-2.1530	-0.2870
1.00	1.00	2.00	1.00	6	7.20	72.0	-0.6620	1.5560
2.00	1.00	3.00	2.00	10	10.00	75.3	1.0710	1.2740
1.00	1.00	2.00	1.00	3	7.90	58.8	2.2080	-0.8720
1.00	1.00	2.00	1.00	11	7.50	72.1	-1.7040	-0.3440
2.00	1.00	1.00	1.00	7	7.00	70.4	-0.7840	1.0560
1.00	1.00	3.00	2.00	8	6.40	65.0	-1.8780	-1.5030
1.00	1.00	3.00	2.00	6	5.40	65.5	-2.5070	-0.8630

2.00	1.00	3.00	2.00	22	11.60	76.0	0.0420	-2.7110
1.00	1.00	3.00	2.00	6	7.40	72.2	-0.4570	1.6300
2.00	1.00	3.00	2.00	19	10.30	83.0	-1.1260	-0.1100
2.00	1.00	1.00	1.00	4	6.00	65.0	-0.0620	1.1870
1.00	1.00	3.00	2.00	2	5.20	56.0	0.0110	-0.8110
1.00	1.00	3.00	2.00	8	8.40	79.0	-0.3960	3.0270
1.00	1.00	3.00	2.00	5	7.00	68.0	-0.3000	0.7760
1.00	2.00	1.00	1.00	7	8.70	70.5	0.3640	0.3810
1.00	1.00	2.00	1.00	9	9.00	68.8	-0.1870	-1.3370
2.00	2.00	3.00	2.00	14	8.70	75.4	-1.8360	-1.0580
1.00	2.00	3.00	2.00	5	8.00	75.0	0.7410	3.3740
2.00	2.00	2.00	1.00	2	4.60	55.0	-0.1600	-0.7320
2.00	2.00	2.00	1.00	20	10.90	87.4	-0.7680	0.9960
1.00	1.00	3.00	2.00	10	10.00	79.0	0.4340	2.0260
2.00	1.00	4.00	2.00	17	9.30	77.0	-1.1600	-0.9600
1.00	1.00	2.00	1.00	9	9.00	68.0	0.4440	-0.8960
2.00	1.00	3.00	2.00	24	8.90	81.0	-2.4680	-1.0880
1.00	1.00	3.00	2.00	9	9.60	75.0	0.4030	1.0090
2.00	1.00	2.00	1.00	17	8.30	77.0	-2.5900	-1.4960
1.00	1.00	3.00	2.00	7	9.00	74.0	0.6660	1.6910
2.00	2.00	3.00	2.00	16	8.90	80.0	-1.3620	0.3820
1.00	1.00	3.00	2.00	4	7.80	57.7	2.0800	-1.6760
1.00	1.00	4.00	2.00	7	6.40	69.0	-1.4480	0.5330
2.00	1.00	1.00	1.00	23	9.10	80.5	-2.5270	-1.9000
2.00	1.00	4.00	2.00	24	10.00	85.0	-2.0830	-0.1860
1.00	1.00	1.00	1.00	9	7.20	69.0	-2.0440	-1.2610
2.00	1.00	3.00	2.00	11	14.50	79.5	4.2710	1.7310
1.00	1.00	1.00	1.00	8	8.50	60.0	-0.2930	-4.1360
2.00	1.00	1.00	1.00	6	7.60	69.0	0.4260	1.1610
1.00	1.00	2.00	1.00	7	7.90	69.7	0.2020	0.7940
2.00	1.00	3.00	2.00	19	10.20	78.2	-1.2090	-1.6560
1.00	1.00	1.00	1.00	4	6.00	65.0	-0.0620	1.1870
2.00	1.00	3.00	2.00	13	8.40	74.2	-1.2970	-0.4520
2.00	1.00	1.00	1.00	23	10.50	81.8	-1.0010	-1.1720
1.00	1.00	1.00	1.00	7	8.00	76.5	-0.3500	2.6270
1.00	1.00	2.00	1.00	2	5.00	57.2	-0.2230	-0.3440
1.00	1.00	1.00	1.00	3	8.80	68.5	4.3850	3.6040
2.00	1.00	2.00	1.00	17	8.60	79.3	-2.3290	-0.7220
1.00	1.00	2.00	1.00	9	6.20	67.3	-2.4430	-1.1520
2.00	1.00	3.00	2.00	9	8.40	72.3	-0.1650	0.6770
1.00	1.00	3.00	2.00	6	7.50	63.2	-0.3540	-1.7200
2.00	1.00	1.00	1.00	13	9.60	77.2	-0.7830	-0.0140
1.00	1.00	1.00	1.00	8	8.90	78.5	0.1160	2.8380
1.00	1.00	3.00	2.00	6	7.00	64.3	-0.8670	-1.3100
2.00	1.00	3.00	2.00	23	11.20	83.5	-0.9240	-0.9990
2.00	1.00	2.00	1.00	9	8.50	80.5	-0.7030	3.0890
1.00	1.00	2.00	1.00	7	8.50	60.1	0.8360	-2.7950

2.00	1.00	3.00	2.00	23	9.00	75.6	-2.2250	-3.0810
1.00	1.00	3.00	2.00	6	7.90	66.6	0.0560	-0.4540
1.00	1.00	1.00	1.00	11	10.60	76.8	0.6810	0.7190
1.00	1.00	3.00	2.00	9	8.60	71.2	0.0410	0.2750
2.00	1.00	2.00	1.00	9	7.50	71.0	-1.7340	-0.5040
2.00	1.00	1.00	1.00	8	7.50	69.1	-0.7030	0.0140
1.00	1.00	3.00	2.00	11	7.90	74.4	-1.3130	0.4780
2.00	1.00	1.00	1.00	8	8.40	72.8	-0.3960	0.6910
2.00	1.00	3.00	2.00	5	7.60	66.9	0.3190	0.3680
1.00	1.00	4.00	2.00	8	6.30	70.4	-1.9850	0.4950
2.00	1.00	3.00	2.00	15	8.30	70.0	-1.7420	-2.6360
1.00	1.00	2.00	1.00	8	7.01	67.8	-1.2260	-0.4670
2.00	1.00	1.00	1.00	12	7.40	72.1	-2.7110	-1.4780
1.00	1.00	1.00	1.00	8	7.80	79.8	-0.3820	3.9690
2.00	1.00	3.00	2.00	10	8.50	67.0	-0.4220	-1.7290
2.00	1.00	2.00	1.00	11	10.60	76.8	0.6810	0.7190
1.00	1.00	3.00	2.00	6	10.40	68.0	3.4710	0.7820
2.00	1.00	3.00	2.00	4	5.00	60.0	-1.7000	-1.3780
1.00	1.00	4.00	2.00	5	6.00	67.5	-1.3110	0.5900
2.00	1.00	3.00	2.00	24	12.70	89.1	0.6430	1.4240
2.00	1.00	2.00	1.00	24	10.50	80.6	-1.6380	-1.5670
1.00	1.00	3.00	2.00	7	8.00	76.6	-0.3500	2.6640
2.00	1.00	3.00	2.00	24	12.50	88.3	0.5010	1.1770
1.00	1.00	2.00	1.00	3	5.00	60.0	-1.0330	-0.4180
2.00	2.00	3.00	2.00	7	8.70	73.8	0.3640	1.6160
1.00	2.00	3.00	2.00	6	6.80	68.1	-0.4690	0.8200
1.00	2.00	2.00	1.00	4	5.40	63.0	-0.8160	0.4030
2.00	1.00	1.00	1.00	3	5.60	62.0	-0.4020	0.3380
1.00	2.00	3.00	2.00	8	7.80	67.1	-0.3820	-0.7260
2.00	1.00	3.00	2.00	24	10.20	80.5	-1.9050	-1.5990
1.00	2.00	1.00	1.00	9	8.70	72.7	0.1420	0.8240
2.00	1.00	2.00	1.00	2	4.80	60.4	0.1340	1.5180
1.00	1.00	2.00	1.00	4	5.40	65.0	-1.2960	0.4870
1.00	2.00	2.00	1.00	4	9.00	65.0	3.5060	1.1870
2.00	1.00	2.00	1.00	20	8.80	82.3	-2.4710	-0.6140
1.00	1.00	2.00	1.00	4	5.70	67.1	-0.4390	2.0100
2.00	1.00	2.00	1.00	2	6.10	60.2	1.1530	0.8230
2.00	2.00	2.00	1.00	7	7.80	73.0	0.0970	2.0270
1.00	1.00	2.00	1.00	6	8.00	70.0	0.8610	1.5390
1.00	1.00	3.00	2.00	12	8.50	77.2	-1.6270	0.4140
2.00	1.00	1.00	1.00	14	10.60	78.5	-0.0490	0.0550
1.00	1.00	2.00	1.00	7	5.60	62.5	-2.8340	-2.6160
1.00	2.00	1.00	1.00	10	10.00	72.6	0.4340	-0.3900
2.00	1.00	3.00	2.00	24	12.30	87.0	-0.0380	0.4420
1.00	1.00	1.00	1.00	13	8.20	71.0	-1.4840	-1.5670
2.00	2.00	1.00	1.00	3	6.80	69.0	1.8060	3.8050
2.00	1.00	1.00	1.00	5	7.00	65.9	-0.3000	-0.0030

1.00	1.00	1.00	1.00	5	6.90	63.8	0.2770	-0.1090
1.00	1.00	2.00	1.00	5	6.90	66.8	-0.4010	0.3310
1.00	1.00	1.00	1.00	6	6.80	67.2	-0.4690	0.4800
2.00	1.00	3.00	2.00	20	8.40	80.5	-2.7950	-1.1830
1.00	1.00	1.00	1.00	5	6.80	65.0	0.1650	0.3520
2.00	1.00	1.00	1.00	4	7.11	63.8	1.2600	0.7170
2.00	1.00	2.00	1.00	9	9.10	73.2	0.5450	1.0060
1.00	1.00	1.00	1.00	10	7.60	73.6	-1.9790	-0.0120
2.00	1.00	3.00	2.00	12	7.50	72.7	-1.9370	-0.5650
1.00	2.00	3.00	2.00	12	9.50	77.6	-0.0300	1.1640
2.00	2.00	2.00	1.00	15	8.80	77.5	-1.9000	-0.6740
1.00	1.00	3.00	2.00	10	9.40	73.0	0.4760	0.4420
2.00	2.00	2.00	1.00	3	5.00	62.0	-0.5360	0.9870
1.00	1.00	1.00	1.00	9	9.00	77.4	-0.1870	1.9170
1.00	1.00	2.00	1.00	13	9.20	78.6	-1.1690	0.4980
2.00	1.00	2.00	1.00	2	6.20	64.0	1.2790	2.3000
1.00	1.00	1.00	1.00	9	10.60	72.0	2.0590	0.5670
2.00	1.00	2.00	1.00	21	8.90	81.9	-2.4890	-1.0000
1.00	1.00	3.00	2.00	11	7.70	73.6	-1.5080	0.1920
2.00	1.00	4.00	2.00	13	10.50	79.5	0.6710	1.3940
1.00	1.00	4.00	2.00	6	8.20	70.0	1.0780	1.5390
1.00	1.00	3.00	2.00	7	10.10	68.3	2.5260	0.2710
1.00	1.00	4.00	2.00	15	7.90	78.4	-2.7250	-0.3580
2.00	1.00	3.00	2.00	8	6.70	67.8	-2.1590	-1.1940
1.00	1.00	2.00	1.00	8	7.51	71.6	-0.6920	0.9380
1.00	1.00	2.00	1.00	11	7.50	71.1	-1.7040	-0.7010
2.00	1.00	1.00	1.00	8	7.90	70.7	-0.9150	-0.1000
1.00	1.00	2.00	1.00	11	7.90	69.2	-1.9700	-2.1320
2.00	1.00	3.00	2.00	4	6.70	61.5	0.0210	-0.8180
1.00	1.00	3.00	2.00	6	7.70	68.2	0.5350	0.8580
2.00	1.00	4.00	2.00	22	10.60	87.6	-1.2630	0.4930
1.00	1.00	3.00	2.00	14	7.30	70.1	-2.4890	-2.2530
2.00	1.00	3.00	2.00	5	5.10	61.0	-2.2210	-1.8240
1.00	1.00	2.00	1.00	24	9.30	77.2	-2.1270	-2.2740
1.00	1.00	1.00	1.00	7	8.90	76.9	1.2590	3.4850
1.00	1.00	3.00	2.00	5	7.00	72.7	-0.3000	2.5200
2.00	1.00	3.00	2.00	24	11.00	83.7	-0.6780	-0.2460
1.00	1.00	2.00	1.00	9	10.40	70.1	1.1730	-0.8450
1.00	1.00	1.00	1.00	3	6.50	65.0	1.4200	2.1950
2.00	1.00	3.00	2.00	24	11.10	85.3	-1.1050	-0.0920
2.00	2.00	1.00	1.00	24	10.00	86.0	-1.5300	0.4670
1.00	2.00	1.00	1.00	5	8.50	66.1	1.2670	0.0710
2.00	1.00	3.00	2.00	6	7.70	68.2	0.5350	0.8580
2.00	1.00	2.00	1.00	6	8.00	68.4	0.1590	0.2160
1.00	1.00	3.00	2.00	9	7.90	70.0	-0.6830	-0.1640
2.00	1.00	3.00	2.00	13	8.60	81.3	-1.7470	1.4840
2.00	1.00	3.00	2.00	24	9.70	80.7	-1.7860	-1.1820

1.00	1.00	3.00	2.00	3	6.30	62.2	1.1620	1.0670
2.00	1.00	2.00	1.00	5	8.30	67.0	1.0560	0.4050
1.00	1.00	3.00	2.00	3	5.10	59.5	-0.9280	-0.6070
2.00	2.00	3.00	2.00	6	6.00	66.4	-1.3880	0.1770
2.00	2.00	3.00	2.00	14	8.30	83.7	-2.2120	1.9200
1.00	1.00	3.00	2.00	9	8.60	74.5	0.0410	1.4820
1.00	1.00	2.00	1.00	6	9.20	73.0	1.3940	1.9280
2.00	1.00	1.00	1.00	5	6.90	65.0	-0.4010	-0.3380
2.00	1.00	3.00	2.00	12	8.50	74.0	-0.9840	-0.1060
2.00	1.00	1.00	1.00	3	7.00	56.0	1.1710	-1.9320
1.00	1.00	1.00	1.00	11	8.80	70.0	-0.4330	-1.0940
2.00	1.00	2.00	1.00	18	8.50	79.7	-1.9870	-0.3990
1.00	1.00	1.00	1.00	7	8.70	72.0	1.0470	1.6540
2.00	1.00	1.00	1.00	20	8.60	84.0	-2.1650	0.3590
1.00	1.00	1.00	1.00	12	7.90	70.0	-2.2180	-2.2570
2.00	1.00	3.00	2.00	23	13.90	89.0	1.1640	0.6540
2.00	2.00	3.00	2.00	11	8.80	74.0	-1.0660	-0.3310
1.00	1.00	2.00	1.00	3	8.20	64.0	3.6110	1.7920
2.00	1.00	3.00	2.00	2	7.00	59.4	2.2940	0.5120
2.00	1.00	1.00	1.00	11	8.70	70.0	-0.5300	-1.0940
1.00	1.00	2.00	1.00	10	9.60	76.5	0.0580	1.0820
2.00	1.00	3.00	2.00	13	8.60	77.0	-1.7470	-0.0870
2.00	2.00	2.00	1.00	8	7.40	73.3	-1.4330	0.8790
1.00	1.00	3.00	2.00	4	5.40	64.3	-1.2960	0.2260
1.00	1.00	1.00	1.00	2	5.70	60.1	0.6450	0.7840
2.00	1.00	2.00	1.00	5	7.00	64.1	0.3900	0.0070
1.00	1.00	1.00	1.00	11	8.80	74.1	-1.0660	-0.2940
2.00	1.00	2.00	1.00	11	7.70	72.8	-1.5080	-0.0940
2.00	2.00	4.00	2.00	5	6.20	68.0	-1.1090	0.7760
2.00	1.00	1.00	1.00	23	9.50	81.1	-1.8170	-1.3870
1.00	1.00	3.00	2.00	2	5.50	57.0	1.1640	0.1010
1.00	2.00	3.00	2.00	5	7.50	72.0	0.9540	3.0420
2.00	2.00	3.00	2.00	10	7.20	68.3	-1.7280	-1.2580
2.00	1.00	3.00	2.00	16	9.50	79.1	-0.8340	0.0810
1.00	1.00	3.00	2.00	8	6.50	64.6	-2.3660	-2.4010
2.00	1.00	2.00	1.00	9	10.20	72.4	0.9810	0.0260
1.00	1.00	1.00	1.00	13	9.60	79.1	-0.7830	0.6800
1.00	1.00	2.00	1.00	11	10.80	80.1	0.8650	1.9560
1.00	1.00	3.00	2.00	6	7.70	65.9	-0.1490	-0.7140
2.00	1.00	2.00	1.00	18	11.10	79.0	0.2430	-0.6270
2.00	1.00	3.00	2.00	11	8.10	71.0	-1.1170	-0.7370
1.00	1.00	3.00	2.00	6	6.10	65.0	-1.7890	-1.0500
2.00	1.00	2.00	1.00	11	7.90	70.0	-1.9700	-1.8320
1.00	1.00	3.00	2.00	11	9.40	73.5	-0.4630	-0.5190
2.00	2.00	2.00	1.00	8	7.20	71.0	-1.0230	0.7160
1.00	1.00	3.00	2.00	4	6.80	60.0	0.1300	-1.3780
1.00	1.00	3.00	2.00	8	8.80	77.0	0.6620	2.9340

2.00	2.00	2.00	1.00	16	9.10	76.0	-1.7680	-1.5310
1.00	1.00	2.00	1.00	5	8.90	67.0	2.5320	1.1210
2.00	1.00	3.00	2.00	6	8.20	65.4	1.0780	-0.2020
2.00	1.00	1.00	1.00	6	6.20	66.0	-1.1580	0.0260
1.00	1.00	1.00	1.00	13	8.40	75.0	-1.2970	-0.1740
2.00	1.00	2.00	1.00	19	10.60	80.0	-0.8770	-1.0760
2.00	2.00	2.00	1.00	13	8.60	74.9	-1.7470	-0.8540
1.00	1.00	3.00	2.00	3	7.50	64.0	1.7470	1.0940
2.00	1.00	3.00	2.00	16	9.00	76.5	-1.2740	-0.7890
1.00	2.00	1.00	1.00	8	7.60	68.2	-1.2260	-1.0430
1.00	1.00	2.00	1.00	7	9.40	78.0	1.0680	3.1880
2.00	1.00	2.00	1.00	5	7.90	68.0	1.4050	1.5050
1.00	1.00	2.00	1.00	10	7.00	72.0	-1.9290	0.0800
1.00	1.00	2.00	1.00	7	9.00	73.0	1.3640	2.0270
1.00	1.00	3.00	2.00	13	8.90	75.1	-0.8320	-0.1390
2.00	1.00	2.00	1.00	17	9.70	80.0	-1.3720	-0.4860
2.00	2.00	3.00	2.00	5	7.10	68.0	-0.1990	0.7760
1.00	1.00	2.00	1.00	3	5.50	56.0	-0.5080	-1.9320
2.00	1.00	2.00	1.00	9	8.30	70.9	-0.9090	-0.5420
2.00	1.00	3.00	2.00	18	7.50	74.2	-2.8420	-2.1910
1.00	2.00	2.00	1.00	11	10.80	80.0	0.8650	1.9190
2.00	2.00	2.00	1.00	24	10.40	86.0	-1.7270	0.1280
2.00	1.00	2.00	1.00	24	9.60	76.0	-2.4380	-3.0110
1.00	1.00	2.00	1.00	2	4.20	57.0	-1.1550	-0.4220
1.00	1.00	2.00	1.00	9	8.90	71.5	-0.2900	-0.3150
2.00	1.00	3.00	2.00	8	8.90	70.0	0.7650	0.3470
2.00	1.00	2.00	1.00	2	5.50	60.3	0.3920	0.8620
1.00	1.00	4.00	2.00	4	4.10	58.0	-2.4510	-1.5590
2.00	1.00	2.00	1.00	2	5.00	62.0	-0.2230	1.5230
1.00	1.00	2.00	1.00	4	7.50	68.0	1.7240	2.3630
1.00	1.00	1.00	1.00	12	9.50	78.1	-0.0300	1.3410
1.00	2.00	2.00	1.00	7	11.50	70.0	3.1830	0.1940
2.00	1.00	2.00	1.00	17	6.50	65.2	-3.5860	-4.8550
2.00	1.00	2.00	1.00	4	5.60	60.0	-0.5650	-0.7740
2.00	1.00	2.00	1.00	5	7.00	69.2	0.3900	1.9660
1.00	2.00	3.00	2.00	14	9.50	79.0	-0.4830	0.8060
1.00	2.00	2.00	1.00	5	7.70	67.0	0.4250	0.4050
1.00	2.00	2.00	1.00	5	6.00	69.0	-1.3110	1.1470
1.00	2.00	3.00	2.00	4	6.80	66.0	0.1300	0.8600
1.00	1.00	3.00	2.00	4	6.00	64.0	-0.0620	0.7950
2.00	1.00	4.00	2.00	23	12.30	86.2	-0.0840	-0.1870
1.00	2.00	2.00	1.00	8	7.30	68.2	-0.9170	-0.3190
2.00	2.00	2.00	1.00	11	9.20	76.0	-0.6640	0.4190
2.00	2.00	1.00	1.00	9	8.70	72.7	0.1420	0.8240
1.00	1.00	1.00	1.00	3	5.50	60.4	-0.5080	-0.2670
1.00	1.00	4.00	2.00	9	10.70	77.7	2.1600	2.6530
1.00	2.00	3.00	2.00	8	8.00	71.2	-0.1680	0.7900

2.00	2.00	2.00	1.00	15	8.50	78.0	-1.5630	0.0760
2.00	2.00	3.00	2.00	12	8.00	62.0	-1.4600	-4.3410
1.00	2.00	1.00	1.00	6	10.00	73.5	2.2170	2.1140
2.00	1.00	3.00	2.00	18	7.30	76.0	-3.0120	-1.6050
1.00	1.00	1.00	1.00	2	5.50	60.5	0.3920	0.9390
2.00	2.00	1.00	1.00	23	10.00	88.0	-1.4090	0.7370
1.00	1.00	2.00	1.00	7	7.40	69.0	-0.3410	0.5330
2.00	1.00	4.00	2.00	13	9.00	79.0	-1.3610	0.6440
1.00	1.00	2.00	1.00	9	8.26	67.0	-0.3100	-1.2620
2.00	1.00	3.00	2.00	5	5.10	61.0	-2.2210	-1.8240
1.00	1.00	4.00	2.00	5	7.30	73.4	0.0030	2.7800
2.00	1.00	4.00	2.00	2	5.90	57.5	1.7520	0.3090
1.00	1.00	3.00	2.00	7	9.80	78.0	1.4710	3.1880
2.00	1.00	3.00	2.00	11	7.10	72.0	-2.0950	-0.3800
1.00	1.00	2.00	1.00	2	6.80	62.3	2.0400	1.6390
2.00	1.00	3.00	2.00	12	7.90	75.1	-2.2180	-0.3650
1.00	1.00	3.00	2.00	9	11.90	76.5	2.6170	1.5760
1.00	2.00	2.00	1.00	8	8.00	71.3	-0.8110	0.1260
1.00	1.00	3.00	2.00	6	10.50	70.0	3.5800	1.5390
2.00	1.00	2.00	1.00	6	6.20	57.5	-1.6870	-3.8430
1.00	2.00	3.00	2.00	8	7.50	67.0	-1.3290	-1.4960
2.00	2.00	3.00	2.00	13	9.20	78.2	-0.5520	0.9410
1.00	1.00	1.00	1.00	10	8.00	62.5	-0.9240	-3.3570
1.00	1.00	2.00	1.00	3	5.00	65.5	-0.5360	2.3960
2.00	2.00	3.00	2.00	18	10.30	81.5	-0.9920	-0.2990
1.00	1.00	1.00	1.00	6	6.90	68.3	-0.3540	0.8960
1.00	1.00	3.00	2.00	9	8.50	77.3	-0.0620	2.5060
1.00	1.00	2.00	1.00	2	5.20	62.1	0.0110	1.5620
2.00	1.00	1.00	1.00	5	7.30	65.5	0.0030	-0.1520
2.00	2.00	3.00	2.00	21	9.90	81.5	-1.6960	-1.1240
1.00	1.00	2.00	1.00	7	9.90	77.6	1.5720	3.0380
1.00	2.00	3.00	2.00	15	9.90	76.7	-0.8920	-0.9560
2.00	1.00	1.00	1.00	2	6.80	60.2	2.0400	0.8230
2.00	1.00	1.00	1.00	22	10.10	78.5	-1.6520	-2.2810
1.00	1.00	4.00	2.00	4	6.90	68.4	0.2390	1.7550
1.00	1.00	1.00	1.00	4	6.90	61.0	1.0110	-0.3820
2.00	1.00	1.00	1.00	24	12.40	78.4	0.0340	-2.2580
1.00	2.00	2.00	1.00	2	6.00	62.5	1.8990	2.3930
2.00	1.00	3.00	2.00	4	6.70	61.5	0.0210	-0.8180
1.00	1.00	1.00	1.00	2	4.20	56.0	-1.1550	-0.8110
2.00	1.00	3.00	2.00	24	12.10	89.0	0.2160	1.3930
1.00	1.00	2.00	1.00	8	8.60	64.8	0.4560	-1.5770
1.00	1.00	1.00	1.00	8	7.70	73.3	-1.1220	0.8790
2.00	1.00	3.00	2.00	21	9.20	74.5	-1.7980	-2.9250
2.00	1.00	1.00	1.00	7	6.40	64.5	-1.4480	-1.1500
1.00	1.00	3.00	2.00	13	7.10	69.5	-2.5080	-2.0890
2.00	1.00	3.00	2.00	8	7.10	67.3	-1.1300	-0.6520

1.00	1.00	2.00	1.00	10	10.00	79.0	0.4340	2.0260
2.00	1.00	3.00	2.00	13	8.00	76.4	-1.6700	0.3140
1.00	1.00	3.00	2.00	11	9.60	85.0	0.3490	4.2670
2.00	1.00	3.00	2.00	11	9.80	72.2	-0.0610	-1.0070
2.00	2.00	3.00	2.00	13	7.20	75.0	-2.4150	-0.1740
1.00	1.00	3.00	2.00	3	5.10	61.0	-0.4010	0.5840
1.00	1.00	3.00	2.00	9	7.50	71.0	-1.0970	0.2020
2.00	1.00	3.00	2.00	14	9.00	74.5	-0.9390	-0.7410
1.00	2.00	2.00	1.00	5	7.00	69.5	-0.3000	1.3330
1.00	1.00	3.00	2.00	7	9.00	70.4	0.6660	0.3430
2.00	1.00	1.00	1.00	13	10.20	77.1	-0.2050	-0.0500
2.00	1.00	3.00	2.00	10	8.20	71.0	-0.7230	-0.2820
1.00	1.00	2.00	1.00	10	9.20	76.0	0.2780	1.5270
1.00	1.00	4.00	2.00	9	10.50	83.0	1.2700	4.0340
2.00	1.00	1.00	1.00	3	6.60	67.0	0.7100	2.2290
2.00	1.00	1.00	1.00	7	8.20	67.0	-0.1440	-0.9300
2.00	1.00	2.00	1.00	5	7.10	66.0	0.5030	0.7370
1.00	1.00	3.00	2.00	11	9.30	73.0	-0.5630	-0.7060
1.00	1.00	1.00	1.00	5	6.60	63.2	-0.0650	-0.3400
2.00	1.00	1.00	1.00	3	7.40	61.0	1.6320	-0.0400
1.00	1.00	2.00	1.00	9	10.60	75.9	1.3660	1.3490
2.00	1.00	3.00	2.00	20	7.40	82.6	-3.1690	-0.0870
2.00	1.00	3.00	2.00	18	9.50	77.7	-1.1320	-1.0510
1.00	1.00	1.00	1.00	2	6.10	61.1	2.0460	1.8100
2.00	1.00	3.00	2.00	4	5.50	55.0	-1.1950	-3.2430
2.00	1.00	3.00	2.00	4	8.50	68.5	2.9120	2.5590
1.00	1.00	2.00	1.00	9	7.70	68.6	-0.8900	-0.6770
2.00	1.00	1.00	1.00	3	5.50	60.3	0.1300	0.3020
2.00	1.00	2.00	1.00	24	11.30	84.2	-0.9270	-0.4370
1.00	1.00	1.00	1.00	2	6.00	59.5	1.8990	1.1430
1.00	1.00	3.00	2.00	7	9.10	82.1	0.7660	4.7230
2.00	1.00	2.00	1.00	24	10.10	86.0	-1.9940	0.1280
2.00	2.00	3.00	2.00	24	11.20	84.0	-1.0160	-0.5000
1.00	1.00	3.00	2.00	11	10.10	74.9	0.8370	0.6570
2.00	1.00	1.00	1.00	24	13.20	88.0	0.5130	0.7570
2.00	1.00	1.00	1.00	12	9.50	78.1	-0.6410	0.7480
1.00	1.00	4.00	2.00	9	7.10	69.3	-1.5110	-0.4200
2.00	1.00	1.00	1.00	13	7.70	71.5	-2.6140	-2.0960
1.00	1.00	3.00	2.00	3	4.70	64.2	-1.3480	1.1700
2.00	1.00	3.00	2.00	21	12.90	74.5	1.2740	-2.9250
1.00	1.00	3.00	2.00	9	11.30	76.5	2.7660	2.2140
1.00	1.00	4.00	2.00	6	8.40	70.5	1.2960	1.7280
2.00	1.00	3.00	2.00	23	11.50	91.0	-0.1850	1.6610
1.00	2.00	2.00	1.00	4	5.80	69.0	-0.3130	2.7550
2.00	1.00	2.00	1.00	2	4.50	60.2	-0.8050	0.8230
1.00	1.00	3.00	2.00	5	6.40	69.0	-0.9070	1.1470
2.00	1.00	2.00	1.00	22	13.50	91.0	1.0040	1.5290

2.00	1.00	2.00	1.00	24	10.50	82.0	-1.1040	-0.7760
1.00	1.00	3.00	2.00	8	9.80	78.0	1.0020	2.6500
1.00	1.00	2.00	1.00	7	7.50	68.0	-0.2310	0.1590
2.00	1.00	3.00	2.00	4	8.90	69.5	2.4160	2.1650
2.00	1.00	3.00	2.00	24	12.40	95.6	0.4300	3.4310
1.00	1.00	1.00	1.00	11	8.50	71.4	-1.3670	-1.3070
2.00	1.00	3.00	2.00	14	9.80	80.0	-0.8010	0.5930
1.00	1.00	2.00	1.00	10	8.20	73.0	-0.7230	0.4420
2.00	1.00	4.00	2.00	20	9.10	80.0	-1.7470	-0.9130
1.00	1.00	1.00	1.00	8	7.10	80.0	-1.1300	4.0430
2.00	1.00	3.00	2.00	23	13.50	80.0	1.4120	-1.7260
2.00	1.00	3.00	2.00	5	6.70	69.5	0.0520	2.0820
1.00	1.00	3.00	2.00	9	9.10	70.5	0.5450	0.0190
2.00	1.00	4.00	2.00	5	8.20	69.3	0.9510	1.2580
1.00	1.00	3.00	2.00	3	5.90	64.9	-0.0870	1.4350
2.00	1.00	3.00	2.00	24	10.50	83.5	-1.1040	-0.3080
1.00	1.00	3.00	2.00	11	10.10	78.2	0.2210	1.2440
2.00	1.00	3.00	2.00	2	4.80	54.6	-0.4560	-1.3550
2.00	1.00	1.00	1.00	6	7.00	69.2	-0.2390	1.2360
1.00	1.00	3.00	2.00	9	12.00	78.5	3.4720	2.9450
1.00	1.00	1.00	1.00	4	7.70	66.9	1.1100	1.1950
2.00	1.00	4.00	2.00	24	11.80	84.7	-0.4820	-0.2800
2.00	1.00	3.00	2.00	23	10.00	82.5	-1.8400	-1.2990
1.00	1.00	1.00	1.00	6	8.00	67.5	0.8610	0.5930
2.00	1.00	4.00	2.00	21	12.50	82.5	0.9410	-0.4100
1.00	1.00	3.00	2.00	12	8.60	70.0	-0.8880	-1.5180
2.00	1.00	4.00	2.00	12	8.00	72.1	-1.4600	-0.7770
1.00	1.00	3.00	2.00	11	7.40	74.1	-1.8010	0.3710
1.00	1.00	4.00	2.00	7	8.80	70.3	0.4640	0.3060
2.00	1.00	3.00	2.00	21	15.40	90.8	2.6630	1.7590
2.00	2.00	3.00	2.00	13	8.40	74.0	-1.2970	-0.5220
1.00	2.00	2.00	1.00	8	8.50	70.6	-0.2930	-0.1380
2.00	1.00	1.00	1.00	18	11.70	84.3	0.1880	0.6230
1.00	2.00	3.00	2.00	9	8.50	74.5	-0.0620	1.4820
1.00	1.00	2.00	1.00	4	6.00	68.8	-0.0620	2.6770
2.00	1.00	1.00	1.00	3	6.70	62.1	1.6770	1.0270
2.00	2.00	3.00	2.00	3	6.20	59.8	1.0330	0.1010
1.00	1.00	2.00	1.00	12	9.00	80.0	-0.5070	2.0110
2.00	1.00	3.00	2.00	18	12.60	77.1	0.9210	-1.7480
1.00	1.00	2.00	1.00	4	5.80	59.6	-0.3130	-0.9310
2.00	1.00	3.00	2.00	2	5.30	59.2	0.1380	0.4340
1.00	2.00	3.00	2.00	6	6.40	64.9	-0.9280	-0.3910
1.00	1.00	2.00	1.00	3	7.10	63.1	1.2860	0.7540
1.00	1.00	2.00	1.00	7	7.00	73.5	-0.7840	2.2140
1.00	1.00	3.00	2.00	3	5.60	55.0	0.2590	-1.8320
1.00	1.00	2.00	1.00	3	6.70	62.0	1.6770	0.9870
2.00	1.00	3.00	2.00	8	6.40	68.5	-1.8780	-0.2080

1.00	1.00	2.00	1.00	4	6.80	68.0	0.1300	1.6050
2.00	1.00	2.00	1.00	4	6.70	54.5	0.0210	-3.4290
1.00	1.00	1.00	1.00	5	4.50	60.5	-2.5770	-1.3780
2.00	1.00	3.00	2.00	3	4.60	54.6	-1.0730	-1.9930
1.00	1.00	2.00	1.00	12	7.80	74.1	-2.3170	-0.7360
2.00	2.00	2.00	1.00	18	9.00	81.3	-1.5600	0.1220
1.00	1.00	2.00	1.00	9	7.60	71.2	-1.6310	-0.4280
2.00	1.00	2.00	1.00	9	7.30	69.0	-1.9400	-1.2610
1.00	1.00	3.00	2.00	5	9.20	70.5	2.8710	2.4660
2.00	2.00	3.00	2.00	8	8.90	68.5	0.1160	-0.9300
2.00	2.00	3.00	2.00	2	5.30	60.3	0.1380	0.8620
1.00	2.00	3.00	2.00	3	6.50	59.5	0.5950	-0.6070
1.00	2.00	2.00	1.00	3	6.30	69.2	0.3650	3.0610
2.00	1.00	3.00	2.00	5	7.60	68.5	0.3190	0.9620

WHZNCHS	WAZWHO	HAZWHO	WHZWHO	BMIZWHO	WAMNCHS	HAMNCHS	WHMNCHS	VAR00004
0.7770	1.3680	1.4160	0.8270	0.8020	113.50	105.00	106.20	26.66
0.1980	0.3460	-0.2300	0.6090	0.6420	98.46	98.82	101.70	10.56
-1.5750	-0.3470	0.9260	-1.2480	-1.3300	91.47	103.20	87.77	24.89
-1.4960	-1.2700	-0.1460	-1.5400	-1.6130	85.40	99.06	86.93	7.80
-0.5420	-0.5530	0.5810	-1.4990	-1.2480	100.20	102.60	93.78	2.38
0.4220	1.0500	1.1010	0.6630	0.5590	113.40	103.20	104.10	7.57
-1.7040	-0.0420	1.9010	-1.1810	-1.5690	93.37	106.20	85.61	16.28
-1.3880	-0.9230	-0.4680	-1.0000	-0.9330	83.74	98.01	87.51	11.98
0.1820	0.4850	0.7670	0.0240	0.0620	110.80	102.80	102.00	4.78
-1.0690	-1.2900	-0.5350	-1.2550	-1.3240	86.34	97.87	90.18	6.23
1.4050	0.1170	-1.1550	1.2380	1.0690	106.30	96.56	116.70	3.04
0.3180	1.5170	2.0640	0.4250	0.4670	124.20	107.10	103.20	6.36
0.6890	-0.1300	-0.8010	0.5430	0.4520	101.40	97.41	107.60	4.16
-1.4410	-2.0220	-1.2340	-1.7600	-1.7900	78.57	95.68	86.73	6.16
-0.3970	-3.5650	-3.1810	-1.1500	-2.5490	68.53	89.84	95.03	1.03
-0.7280	-0.8690	0.0370	-1.1530	-1.2110	93.18	100.00	92.43	5.04
1.5750	0.2960	-1.4220	1.5920	1.4990	104.50	95.25	117.20	4.32
0.3740	-2.3350	-3.9580	0.0550	0.1690	71.41	85.95	104.20	4.35
1.8440	0.4340	-1.0770	1.7490	1.4550	112.00	96.88	123.30	2.25
0.9760	0.4340	-0.0980	0.7030	0.6730	112.00	100.20	111.70	2.91
0.0560	0.4630	0.8940	-0.0110	-0.0520	109.70	103.00	100.60	6.16
0.3260	-0.4840	-1.1720	0.2840	0.2480	91.94	95.57	103.30	6.16
0.3310	1.0910	1.2140	0.6980	0.5470	110.40	103.30	103.10	10.00
-3.0610	-0.7350	3.3510	-2.9070	-3.6390	87.68	110.00	76.27	19.47
-0.3450	-0.9520	-2.2680	0.1810	0.6250	84.48	91.56	96.94	12.83
1.6060	-0.3760	-2.1960	1.3680	1.2120	94.70	92.05	118.50	4.06
0.3310	1.0250	1.4750	0.2270	0.2890	120.70	105.40	103.50	5.73
-1.3910	0.1010	1.9740	-1.0170	-1.3480	98.03	105.60	88.80	12.57
-0.4550	0.5590	1.2240	0.0810	-0.1710	100.80	103.40	96.41	15.72
1.1300	1.6090	1.2370	1.4140	1.2560	118.90	103.80	110.00	10.17
-0.9110	-1.0310	-0.5540	-0.9040	-0.9540	87.68	97.79	91.89	7.15
0.3020	-0.1070	-0.5810	0.3830	0.3170	98.34	97.72	103.10	6.16
-0.4610	-0.0130	0.1090	-0.0800	-0.0910	95.33	99.89	96.23	11.22
0.9930	-1.4830	-3.9260	0.9970	1.2210	81.13	87.34	110.70	4.75
1.8340	0.5220	-1.7770	1.8600	2.0850	100.60	93.48	117.50	7.67
0.6450	1.3580	1.2880	0.9300	0.8210	117.30	103.80	106.30	7.87
3.1760	1.5500	-2.2060	3.3630	3.9290	113.90	92.59	128.70	13.95
0.8110	0.5090	-0.9340	1.2710	1.4590	100.70	96.39	106.70	15.46
-2.3510	-2.0670	-0.6640	-1.4010	-2.3770	76.06	97.49	80.36	9.77
-2.5190	-1.2380	1.1970	-1.4040	-2.6730	83.87	103.20	79.64	12.21
-0.5110	-0.1120	-0.0430	-0.1690	-0.1330	97.23	101.60	95.46	23.08
-0.1590	-0.1400	-0.6480	0.2060	0.3320	93.59	97.53	98.69	10.73
-0.6430	-0.3340	-0.3110	-0.2620	-0.2000	91.62	98.58	94.75	11.38
0.1380	-1.0670	-3.0310	0.6690	1.2140	84.43	88.66	101.10	13.59
0.0260	-0.1120	-0.9260	0.5160	0.6660	97.23	98.49	100.30	20.09
0.2050	1.7880	3.0370	0.6880	0.1580	115.20	109.10	101.80	20.09

2.2140	0.6550	-3.7720	3.3940	4.3200	106.10	88.32	130.40	11.88
-0.8720	-1.0420	-0.4430	-0.9830	-1.0550	88.74	98.15	92.03	6.36
-3.8280	-2.7480	1.6150	-4.9680	-4.8330	75.37	105.10	65.86	7.08
-1.6370	-0.4650	1.5650	-1.7040	-1.8240	95.72	105.10	84.25	8.39
-1.5850	-1.3480	0.2860	-2.0050	-2.0670	86.01	100.70	84.08	6.42
-0.5110	1.2100	2.8150	-0.6970	-0.4030	125.90	110.30	94.82	6.00
-0.9060	-1.8320	-2.0940	-0.9740	-0.8090	75.74	92.39	91.05	7.15
-1.2840	-0.9670	-0.8100	-0.7510	-0.6710	85.77	97.19	89.94	16.77
-2.1290	-1.7100	-0.3700	-1.2280	-2.2960	81.02	100.50	81.01	21.96
1.8320	2.5920	2.2470	1.8890	1.8530	142.80	107.70	117.20	8.16
1.4920	0.3330	-1.8670	1.7510	1.8960	99.59	93.71	114.90	6.88
-0.3820	-1.5600	-2.8840	-0.1790	0.2650	79.80	90.49	96.70	8.23
-1.8000	-1.6890	0.7810	-3.5600	-3.0290	86.69	103.30	79.54	2.51
-0.1320	-0.2710	-1.2320	0.4490	0.6550	92.05	95.33	98.89	16.38
-0.6560	0.0900	1.3360	-0.9550	-0.8780	107.50	104.90	93.27	5.57
1.3480	-0.1120	-3.6410	2.3070	3.0920	97.23	88.79	118.40	12.21
0.0980	0.5350	1.2380	-0.5320	-0.1960	116.80	105.00	101.20	2.91
1.5520	-0.1550	-2.7490	1.6060	2.0000	91.91	90.07	115.00	7.47
-1.3700	-0.6710	-0.1170	-0.8880	-0.8860	87.95	99.40	89.01	20.62
-2.7900	-1.8020	1.1940	-3.3430	-3.3870	82.85	103.50	75.58	7.70
-2.7050	-1.9920	0.9990	-3.5800	-3.4940	82.23	103.20	75.15	6.23
-1.9630	-0.9130	0.5250	-1.7170	-1.8320	86.72	101.90	85.03	23.74
-1.0050	1.4900	4.5130	-0.4870	-1.2150	114.40	115.10	91.87	19.89
-2.8840	-1.1520	2.3730	-3.3780	-3.3580	90.45	107.60	75.09	8.23
-2.4910	-0.6330	2.3410	-1.1440	-2.7390	88.67	106.90	80.50	17.03
-0.7960	-1.0420	-0.5350	-0.9110	-0.9820	88.74	97.87	92.69	6.23
-2.0230	-1.8930	-0.3270	-1.2890	-2.3520	79.70	98.50	82.18	7.51
-2.4090	-1.3620	0.6030	-2.2780	-2.4290	80.07	101.70	78.40	12.24
-0.2160	0.8020	1.7980	-0.3180	-0.2470	117.40	106.50	97.82	6.16
-0.1120	0.8680	1.2680	0.4270	0.2170	104.40	104.10	99.08	18.28
0.3560	2.6260	4.5210	0.8640	0.3710	140.10	115.30	103.00	13.68
-1.6350	-1.4450	-1.2780	-1.1500	-0.9540	79.44	95.15	85.86	14.54
-2.4150	-0.5060	2.8370	-3.2980	-2.8250	100.30	110.00	77.38	5.73
-0.6770	0.0740	0.4840	-0.2010	-0.2910	95.21	101.30	93.91	11.98
-0.8230	-0.4360	-0.5810	-0.2350	-0.1440	89.29	97.61	92.97	15.46
-1.3250	-1.1490	-0.6640	-1.1130	-1.0490	84.17	97.49	88.93	9.77
-0.6430	0.1380	0.7590	-0.2620	-0.3840	97.50	101.90	94.75	11.38
0.1600	-1.8570	-4.0150	0.2990	0.8760	77.26	86.88	101.60	6.88
-0.6990	0.3750	1.4750	-0.4170	-0.6100	103.80	104.60	93.41	9.41
1.7210	0.5880	-2.9870	2.7840	3.4310	105.30	91.13	122.90	13.95
-0.4750	-2.2390	-3.9580	-0.2230	0.4830	72.67	85.46	95.48	8.89
-1.3840	-2.1040	-2.3390	-1.2880	-0.9460	72.79	91.45	86.84	8.95
-0.0740	0.2290	0.6200	-0.1310	-0.1690	105.50	102.00	99.31	5.73
-2.9710	-0.9220	2.3670	-2.7390	-3.2280	83.93	107.80	75.27	17.40
-0.8740	-0.5060	-0.2690	-0.5160	-0.5040	88.72	98.68	91.92	10.46
-2.3160	-1.8580	1.0760	-3.8360	-3.4150	85.24	104.10	76.12	3.86
-2.9220	-1.4250	2.0410	-3.5300	-3.4840	87.71	106.50	74.42	7.70

-2.4910	-2.4970	-1.8830	-2.1440	-1.9900	72.52	93.64	80.50	17.03
-1.6750	0.0480	2.2940	-1.3340	-1.6790	98.07	107.40	84.96	12.14
-3.1900	-2.1390	1.4730	-4.1050	-4.0060	80.86	104.70	71.36	6.88
-0.8260	0.4340	2.0540	-1.2460	-0.9620	112.00	107.40	91.94	4.85
0.3180	-1.8870	-4.4080	0.5210	1.2460	77.15	85.46	103.10	7.31
-1.0150	-1.6210	-0.7280	-1.5290	-1.6400	83.24	97.22	89.45	5.04
-1.0060	-0.6160	-0.3900	-0.6110	-0.5620	87.08	98.25	90.82	11.19
-1.1710	1.5170	4.1300	-0.9650	-0.9950	124.20	113.80	90.15	9.41
0.0470	-1.8230	-3.0930	-0.0820	0.0460	78.63	89.91	100.50	5.17
-2.5010	-0.7160	1.9010	-2.1480	-2.5620	86.03	106.20	78.87	16.28
-1.4370	-1.2070	-0.2990	-1.3720	-1.4110	84.96	98.57	87.68	8.49
-1.4040	1.7580	4.7940	-1.3400	-1.1010	130.40	116.50	87.94	8.43
-2.6280	-1.3320	0.8230	-2.4220	-2.7090	82.34	102.60	79.77	21.40
-0.8860	0.4470	1.7870	-0.4760	-0.7450	103.00	105.70	91.92	11.19
0.6930	1.5550	1.4160	1.0560	1.0400	118.70	106.90	107.70	29.52
-0.6020	0.5710	1.9370	-0.7400	-0.6580	114.10	107.00	93.94	6.36
-0.0700	-1.2580	-0.9730	-0.7300	-0.9540	90.92	96.82	99.23	3.07
-1.3410	0.4540	2.7220	-1.4680	-1.3710	112.40	109.70	86.85	7.47
-2.4710	-1.4120	0.3830	-2.1200	-2.3750	81.23	100.90	80.65	16.94
1.3340	-0.7660	-1.8430	1.0900	0.3950	98.16	93.87	117.90	1.63
1.2610	0.0410	-2.6320	1.7040	2.3020	96.22	91.16	111.50	12.90
0.4780	-1.7540	-4.7820	1.1450	2.0440	78.84	83.21	106.80	9.12
-2.0930	-1.6980	0.9780	-3.5200	-3.1320	86.91	103.80	78.30	3.79
-0.1660	0.5700	0.9400	0.0020	0.0080	103.20	103.40	98.74	27.25
-1.7300	-0.9820	-0.1320	-1.3480	-1.3470	85.28	99.41	86.38	22.55
-0.9480	-0.6090	0.6230	-1.2780	-1.3040	96.18	102.10	90.33	5.80
-1.6150	-1.0020	0.9390	-2.0890	-2.0760	91.67	103.10	83.71	6.23
-0.5040	0.8600	1.9120	0.0840	-0.2640	105.00	106.20	95.67	15.10
0.3020	-1.2120	-1.4910	-0.2150	-0.4670	91.31	95.46	103.70	2.78
-0.1590	-1.4260	-3.2780	0.2060	0.9000	81.48	89.03	98.69	10.73
0.8100	1.3320	1.1730	1.0570	0.9180	116.80	103.60	107.40	8.89
0.1770	0.2150	0.6760	-0.3200	-0.2230	111.10	102.80	102.10	3.60
-0.8850	-0.5120	1.0410	-1.8780	-1.4870	101.90	104.30	90.12	2.78
-1.9660	0.3460	3.3670	-1.8970	-2.0320	106.90	110.80	83.58	9.77
0.0350	-0.8780	-2.5890	0.4550	1.0300	86.63	91.31	100.30	12.30
-1.8340	-1.4000	0.4470	-2.2030	-2.2560	86.67	101.20	83.39	6.62
-0.1300	0.6030	1.5310	-0.7780	-0.3290	115.60	105.90	98.58	3.04
-1.7590	-1.1910	-0.4680	-1.2960	-1.3030	83.36	98.23	86.15	15.79
-1.7310	-1.1770	0.3490	-1.7950	-1.9100	84.58	100.80	83.42	8.59
-2.5420	-1.7030	-0.2170	-2.2330	-2.2850	76.57	98.91	78.29	15.46
-2.4420	-0.8860	2.0350	-1.6220	-2.7560	91.77	106.20	79.30	9.02
0.4210	1.3330	1.5540	0.9060	0.6640	112.10	104.90	103.50	13.06
3.7870	1.8710	-1.2730	3.8570	3.5950	132.00	96.23	148.40	2.12
-1.7240	-1.2490	-0.2690	-1.5380	-1.5460	81.15	98.68	84.07	10.46
-1.8490	-0.7230	1.3460	-1.8640	-2.0340	90.81	104.20	82.49	9.18
-0.7960	-1.8090	-1.5950	-1.1310	-1.1990	78.46	94.12	91.84	5.57
-2.6160	-3.4030	-1.0010	-3.9270	-3.9240	68.83	96.58	74.40	4.78

2.1900	0.3810	-2.7600	2.3190	2.8220	100.40	89.71	118.30	13.68
-2.2430	-0.6390	2.1280	-2.3290	-2.4790	94.32	106.50	81.05	9.18
-1.3690	-0.7030	-0.0840	-0.8530	-0.9240	88.35	99.59	89.36	18.74
-1.3550	-0.5560	1.3360	-1.8760	-1.7530	99.19	104.90	86.10	5.57
0.9700	-0.5530	-1.2190	0.8450	0.1810	100.20	96.41	113.40	1.33
-2.7170	-0.2370	3.7970	-2.4980	-3.1580	95.65	111.30	78.49	14.87
-1.3420	-0.6290	0.9990	-1.6120	-1.6170	95.93	103.20	87.68	6.23
0.0210	0.4370	0.6170	0.2310	0.1230	104.30	101.50	100.20	7.93
0.9580	0.1010	-1.4140	1.1860	1.2420	98.03	95.12	109.60	6.75
-1.5000	-1.3490	-1.0680	-1.1860	-1.0070	81.67	96.24	87.81	11.71
-2.2670	0.5760	4.3150	-2.1400	-2.3870	109.60	113.80	81.51	11.38
0.5310	-0.8370	-1.0210	0.1200	-0.3880	97.69	96.90	107.20	1.36
-1.6070	-0.3590	1.1340	-1.3040	-1.4880	92.00	103.70	87.73	23.51
-0.8280	0.8070	2.5100	-0.3160	-0.7790	104.80	107.30	93.45	14.87
-0.8270	-0.6110	-0.9320	-0.2580	-0.0920	87.41	96.36	92.85	14.54
1.5520	0.7320	-0.8880	1.6060	1.6600	105.10	96.52	115.00	7.47
-2.0120	-2.1340	-1.4660	-1.8540	-1.7930	75.45	95.87	80.93	18.28
-0.2680	0.6960	1.3500	0.1220	-0.0720	104.60	103.70	97.81	11.38
-2.3920	-2.3090	-1.6040	-2.1830	-1.9520	73.60	94.54	80.83	13.06
-0.7310	0.7500	2.2290	-0.3950	-0.6510	107.90	106.50	93.97	10.56
-1.9330	-0.8030	0.4990	-1.4370	-1.5910	85.18	101.40	83.89	17.33
4.4580	1.5590	-2.0360	4.1350	3.7570	128.90	93.11	158.10	2.22
-2.1800	-1.4690	0.7410	-1.5000	-2.6060	83.03	102.10	78.94	8.16
-2.2140	-2.4000	-2.1500	-1.8170	-1.5590	73.33	92.72	82.61	16.28
-1.9500	-1.7100	-0.6970	-1.9470	-1.9650	81.02	99.31	82.52	20.85
-1.4380	-1.9310	-1.3250	-1.6120	-1.5960	78.42	95.39	87.09	6.88
3.8160	4.1320	2.1260	3.9150	3.6310	147.10	106.20	134.20	15.33
4.0660	-0.1290	-4.8150	4.0670	3.7500	96.78	84.55	150.20	2.51
-0.6480	0.3280	1.4330	-0.5090	-0.6400	105.40	104.70	93.74	8.16
-0.5020	0.2730	1.0430	-0.2790	-0.4320	102.50	103.10	95.20	8.69
-0.3950	-0.7900	-1.8260	0.0950	0.4820	87.49	93.83	96.86	14.14
-1.3550	-0.5560	1.3360	-1.8760	-1.7530	99.19	104.90	86.10	5.57
-1.2020	-0.7230	-0.3920	-0.7730	-0.7140	85.78	98.28	89.21	12.14
-0.5050	-0.5770	-1.1720	0.0320	0.1900	89.54	95.56	95.88	19.07
-2.6390	-0.3330	3.3800	-2.5320	-2.9820	95.94	110.10	78.76	12.63
0.1650	-0.8650	-0.6190	-0.4300	-0.7570	96.32	98.48	102.20	1.63
1.0970	3.2980	4.1450	1.2290	1.4870	163.00	115.00	110.40	7.84
-2.5450	-1.9900	-0.7330	-2.2640	-2.2740	76.26	97.37	79.89	15.13
-1.8930	-2.3680	-1.1780	-2.3180	-2.3250	72.43	95.53	81.25	7.01
-0.6900	0.1710	0.8910	-0.2970	-0.4620	98.13	102.60	93.65	10.63
1.2300	-0.5170	-2.0760	1.1180	0.9580	95.60	93.19	113.90	3.70
-0.8640	-0.2600	0.1110	-0.4140	-0.4350	92.20	99.95	93.09	13.26
-2.0150	0.2940	3.5710	-1.6470	-2.2480	101.30	110.60	84.00	14.41
0.1240	-1.1400	-1.5620	-0.1700	-0.2920	89.22	94.81	101.40	4.19
-0.4740	-0.5710	-1.1480	0.0530	0.2230	90.25	96.17	96.32	19.27
-2.9070	-0.4240	3.8020	-2.6930	-3.4340	92.58	111.30	77.17	16.28
4.2850	0.8690	-3.1030	3.8240	3.6220	110.30	88.94	152.60	3.10

-0.8450	-1.8780	-3.1250	-0.3190	0.2300	76.75	88.31	92.56	13.32
0.4040	-0.0450	-0.4870	0.3990	0.3230	100.70	98.20	104.20	5.40
0.4030	1.0930	0.9670	0.8620	0.7390	107.50	102.60	103.70	12.90
-0.1150	0.3630	0.4360	0.2500	0.1490	100.50	101.10	98.92	9.77
-1.7320	-1.5630	-0.4330	-1.7720	-1.8060	81.69	98.16	85.07	8.30
-0.8090	-0.4840	0.1380	-0.6870	-0.7850	91.94	100.10	92.19	8.26
-1.8430	-0.8150	0.6430	-1.5300	-1.6750	85.48	101.80	83.50	12.30
-1.1350	-0.2370	0.9870	-0.9050	-1.0620	95.65	102.60	90.49	9.64
-0.0930	0.1100	0.4780	-0.1810	-0.2190	104.20	101.50	99.13	5.57
-2.7210	-1.9420	0.6880	-3.1190	-3.2360	77.23	101.90	74.24	9.18
-0.0990	-1.2070	-2.7470	0.1830	0.6450	81.00	90.00	99.06	8.89
-0.9980	-1.0450	-0.4110	-1.0540	-1.1220	85.93	98.17	90.19	7.34
-2.2120	-2.4060	-1.5320	-2.2980	-2.1400	72.90	94.76	81.28	9.08
-3.1380	-0.1600	4.6610	-2.9750	-3.6380	95.62	115.50	73.79	17.13
1.2760	0.0220	-1.8100	1.3030	1.4430	95.29	93.34	112.60	6.82
0.4030	1.0930	0.9670	0.8620	0.7390	107.50	102.60	103.70	12.90
3.4080	2.8600	0.9920	3.0710	3.1000	144.30	103.10	132.80	7.47
-1.0230	-2.9060	-1.8760	-2.2370	-2.5440	74.84	94.20	88.38	2.51
-2.5250	-1.9920	0.7620	-3.4060	-3.3240	82.23	102.40	76.53	5.93
-0.0250	0.8030	1.0440	0.2470	0.2300	107.70	105.50	99.78	28.01
-0.6210	-1.2820	-2.1370	-0.2210	0.1150	85.07	94.17	94.22	16.38
-2.6630	-0.3330	3.4260	-2.5570	-3.0150	95.94	110.20	78.58	12.73
-0.0450	0.6780	0.7960	0.2510	0.2550	106.00	104.50	99.60	27.02
-1.0230	-2.0200	-0.6860	-2.2370	-2.3040	83.57	98.19	88.38	2.51
-1.0530	0.4370	2.1370	-0.7560	-1.0050	104.30	106.20	91.30	10.40
-1.3740	-0.5850	1.0360	-1.4990	-1.5940	94.34	103.30	86.54	7.54
-1.4650	-1.4060	0.4120	-2.3100	-2.2220	89.27	101.70	84.51	4.45
-0.9410	-1.0850	0.2930	-1.8970	-1.7430	93.60	101.50	89.87	3.20
0.3020	-0.1600	-0.7070	0.3550	0.3170	95.62	97.16	103.00	6.88
-0.8900	-1.5360	-2.1690	-0.5520	-0.2270	82.64	94.05	91.71	16.28
-0.4420	0.4570	1.0570	-0.0090	-0.1900	101.60	103.20	95.95	10.96
-1.3740	-0.5120	1.6300	-2.5020	-1.9040	101.90	106.40	84.91	3.24
-2.4260	-2.2570	0.5270	-3.7350	-3.5170	80.83	102.10	75.94	4.52
2.7650	2.7120	1.3360	2.5120	2.6670	148.80	104.90	129.10	5.57
-2.9130	-2.2650	-0.6780	-2.6820	-2.8020	74.27	97.69	77.30	18.05
-2.4900	-0.9700	2.3060	-3.2440	-3.0110	94.23	108.30	75.26	6.88
0.5500	0.7390	0.8810	0.1060	0.3540	117.50	103.60	106.80	2.58
-1.6050	0.1680	2.4690	-1.3220	-1.6240	101.20	108.00	85.35	11.19
-0.4750	0.7460	1.8740	-0.2230	-0.3890	111.00	106.20	95.48	8.89
-2.1960	-1.1530	0.6150	-1.9270	-2.1150	83.74	101.50	82.42	13.26
0.0050	0.4460	0.1840	0.4890	0.4770	99.51	100.20	100.00	14.41
-1.1470	-3.4870	-3.0680	-2.1480	-2.3700	67.16	89.95	87.81	3.40
0.8770	0.8070	-0.2910	1.2510	1.3000	104.80	98.60	108.30	9.48
-0.2420	0.1040	-0.0430	0.1370	0.1840	99.66	101.60	97.85	23.08
-0.5450	-0.9230	-1.6080	-0.2230	0.0260	83.74	94.04	94.88	9.64
-1.6690	1.2100	4.3830	-1.7820	-1.4560	125.90	115.90	83.87	8.16
-0.5260	-0.6290	0.0040	-0.8170	-0.8510	95.93	99.99	94.91	5.01

0.3850	0.0050	-0.0990	0.1540	0.0740	103.70	99.56	104.20	4.88
-1.0210	-0.7570	0.4300	-1.3470	-1.3570	94.56	101.40	90.34	5.50
-1.0640	-0.5850	0.6390	-1.2070	-1.2940	94.34	101.90	89.44	6.95
-3.0230	-2.6790	-1.3170	-2.8470	-2.8350	70.90	95.55	76.26	16.28
-0.2360	-0.1140	0.4420	-0.4480	-0.4970	102.20	101.40	97.58	5.57
0.6750	0.8130	0.7820	0.4790	0.5080	117.50	102.90	107.40	4.88
-0.1030	0.8220	1.2640	0.3620	0.1620	106.30	103.90	99.07	11.35
-2.3940	-1.7030	0.1470	-2.4070	-2.5010	79.67	99.96	80.16	10.23
-1.8860	-1.4630	-0.5070	-1.7000	-1.6590	78.68	97.84	82.72	10.96
-0.7340	0.4870	1.3960	-0.1490	-0.4140	99.67	104.40	93.70	15.10
-1.9040	-1.4370	-0.6560	-1.5600	-1.4930	80.94	97.59	84.79	13.52
0.3320	0.8380	0.6220	0.7630	0.6660	105.40	101.70	102.90	11.19
-1.6840	-1.2260	1.0560	-2.7850	-2.4550	92.61	104.10	81.93	3.96
-1.6390	0.1010	2.4200	-1.2550	-1.6810	98.03	107.00	86.90	13.42
-1.6820	-0.6470	0.6880	-1.2540	-1.4510	88.35	101.80	86.66	14.51
-0.8880	0.8730	2.7800	-1.5520	-0.8660	119.40	110.20	90.95	4.06
2.1830	2.0390	0.7670	2.2600	2.1880	123.80	102.20	119.20	10.40
-2.7230	-2.3120	-1.1240	-2.4340	-2.4490	73.94	96.21	78.74	17.63
-1.8800	-1.0280	0.3260	-1.6280	-1.7230	83.31	100.70	82.98	11.65
-0.0150	1.0850	1.6240	0.5520	0.2650	107.20	105.30	99.88	16.84
-0.2250	0.9470	1.8740	0.0500	-0.1150	113.80	106.20	97.86	8.89
2.8920	2.2580	0.4390	2.6960	2.6920	131.00	101.10	127.60	7.67
-3.1830	-2.4090	-0.3010	-3.1480	-3.2760	72.66	98.72	74.71	14.31
-1.6770	-2.2860	-1.2790	-2.1010	-2.1300	76.29	95.54	84.52	6.09
-1.5710	-0.4730	1.1950	-1.3850	-1.5840	92.06	103.70	85.38	10.10
-1.4380	-1.2490	-0.6660	-1.2610	-1.2170	81.15	97.31	86.52	9.71
-1.1040	-0.7980	0.0350	-1.0180	-1.0980	89.95	99.63	90.43	8.07
-0.5690	-1.5970	-2.2950	-0.5180	-0.3190	80.12	92.41	94.91	7.01
0.8500	-0.3950	-1.1550	0.5570	0.3790	100.30	96.56	110.10	3.04
-0.2400	0.4340	1.0800	-0.1240	-0.2340	106.80	103.40	97.65	7.61
-1.9630	-0.9130	0.5250	-1.7170	-1.8320	86.72	101.90	85.03	23.74
-1.3840	-2.1040	-2.3390	-1.2880	-0.9460	72.79	91.45	86.84	8.95
-1.2810	-3.3700	-2.3170	-2.5650	-2.8460	69.89	92.55	85.82	2.84
-0.8820	-1.7540	-2.6430	-0.4530	-0.0630	78.84	91.37	91.09	14.74
-1.2660	1.2420	4.1530	-0.7340	-1.3060	115.50	113.80	89.03	14.44
-2.9070	-0.6290	3.2250	-3.1990	-3.2570	95.93	110.30	75.61	9.54
-0.5290	-0.3480	-0.6290	-0.0750	0.0100	93.25	99.07	95.16	21.04
2.1730	1.4400	-0.8340	2.4390	2.5000	113.30	96.91	121.30	7.64
-0.6560	0.8510	2.4820	-0.9550	-0.6580	120.40	109.20	93.27	5.57
-0.9960	-0.7950	-0.5990	-0.7000	-0.6370	89.93	99.66	91.09	21.17
-1.8960	-1.1380	0.0840	-1.7620	-1.8350	84.77	101.80	83.00	23.54
1.3550	1.1300	0.0990	1.4490	1.4090	116.50	100.30	114.30	5.11
-0.2400	0.4340	1.0800	-0.1240	-0.2340	106.80	103.40	97.65	7.61
-0.1330	0.0700	0.3530	-0.0890	-0.1720	102.00	100.90	98.79	6.49
-0.6010	-0.3320	-0.0610	-0.3630	-0.4250	92.29	99.36	94.28	8.89
-2.9480	-1.2600	1.8010	-2.7320	-3.2640	82.59	105.30	76.92	17.03
-1.1870	-1.3950	-1.5590	-0.7720	-0.6230	82.23	95.52	88.70	17.99

0.1970	0.6030	1.1510	-0.2110	-0.0460	116.70	104.50	102.30	4.06
0.7560	0.9120	0.5250	0.8450	0.8040	113.70	101.70	107.80	5.63
-0.6570	-1.8580	-0.9300	-1.6810	-1.8750	85.24	97.37	92.44	2.35
-1.8550	-1.6210	0.2860	-2.3930	-2.4410	83.24	100.70	81.37	6.42
-3.7240	-1.7750	2.2830	-3.7630	-4.3560	77.92	106.80	71.13	19.47
-1.0420	0.3630	1.8020	-0.5780	-0.8780	100.50	105.80	90.69	12.40
-0.1700	1.3580	2.5020	0.1500	-0.0550	117.30	107.60	98.58	9.77
-0.2990	-0.7570	-0.4220	-0.6350	-0.6910	94.56	98.62	97.03	4.52
-1.0320	-0.4190	-0.0020	-0.5910	-0.6020	89.17	99.59	90.71	11.98
3.8050	0.8120	-2.6420	4.2170	3.2970	117.00	91.64	152.70	1.33
0.5490	0.0740	-1.1020	0.8170	0.9550	95.21	95.81	105.00	8.89
-2.3250	-1.5440	-0.3490	-1.9090	-1.9440	78.52	98.49	80.56	17.03
-0.2380	1.0580	2.0370	0.1690	-0.0790	112.90	106.50	97.80	10.40
-3.0570	-1.7860	0.4280	-2.8730	-3.0490	76.85	101.40	75.66	21.34
-0.8590	-1.8160	-2.4160	-0.7870	-0.5180	77.83	92.00	92.45	7.57
1.0710	1.3280	0.6880	1.3110	1.3150	112.00	102.50	108.90	26.23
-0.9850	-0.6170	-0.2350	-0.6700	-0.6500	89.25	98.82	91.88	10.56
2.0930	2.7090	2.0070	1.9050	2.2000	151.90	107.50	122.80	4.98
2.1900	1.8860	0.4810	2.1330	2.2730	134.80	102.30	127.50	2.32
0.4190	-0.0190	-1.1020	0.6940	0.8290	94.13	95.81	103.80	8.89
-0.6820	0.4300	1.4160	-0.2500	-0.4820	100.60	103.90	94.51	12.63
-2.0270	-1.2600	0.0290	-1.7310	-1.8000	82.59	99.69	83.75	13.06
-2.5650	-1.3920	1.2140	-2.6650	-2.8650	84.26	103.30	78.66	10.00
-2.1560	-2.2570	0.1900	-3.4510	-3.2740	80.83	101.00	78.20	4.19
0.0200	0.1850	0.8310	-0.6580	-0.3890	109.80	103.50	100.30	2.55
0.4020	0.1220	0.0370	0.1990	0.1290	105.20	100.00	104.40	5.04
-1.0140	-0.6170	-0.1920	-0.6980	-0.6840	89.25	98.95	91.65	10.66
-1.6720	-1.0280	0.0080	-1.4190	-1.4630	83.31	99.64	84.70	11.02
-2.4320	-1.7040	0.9990	-3.1810	-3.1130	84.97	103.20	77.66	6.23
-1.4820	-1.4150	-1.3920	-0.9400	-0.7930	81.01	94.74	87.81	18.38
1.1790	0.5350	-0.0390	0.8500	0.7600	116.80	100.40	115.60	1.99
-1.6960	0.6830	3.6000	-1.5100	-1.6790	112.70	112.40	84.31	10.40
-0.9230	-1.3550	-1.2830	-0.9070	-0.8450	80.72	95.15	90.99	7.67
-1.0640	-0.2640	0.1770	-0.4770	-0.5370	90.92	100.30	91.04	16.48
-0.7040	-2.5570	-2.7300	-1.2150	-1.2820	74.01	91.03	92.94	4.32
1.1680	1.2590	0.1910	1.5380	1.5170	111.10	100.10	111.10	9.31
-1.3230	-0.2600	0.8940	-0.8370	-1.0610	92.20	102.40	89.53	14.97
-0.1540	1.2660	2.3840	0.3760	-0.0670	109.50	107.00	98.79	15.89
0.4140	-0.2780	-0.8140	0.3510	0.2680	98.14	97.17	104.40	5.01
0.8030	0.6540	-0.5900	1.2600	1.3800	102.50	97.62	106.60	16.38
-0.6690	-0.6070	-0.7060	-0.3600	-0.2930	87.64	97.18	93.72	9.64
-1.4340	-2.3650	-1.2350	-1.1950	-2.2660	77.75	95.84	85.78	4.52
-0.8590	-1.5970	-1.9520	-0.7870	-0.6090	80.12	93.48	92.45	7.57
-0.0720	-0.0130	-0.4490	0.2680	0.3440	95.33	98.15	99.41	10.17
-1.7780	-0.8220	0.9410	-1.7070	-1.8780	88.26	102.80	83.31	9.64
1.6350	-0.2640	-1.8760	1.4860	1.1420	101.80	94.20	120.20	2.51
-1.4040	0.8250	3.4770	-0.8850	-1.4330	107.90	111.50	87.85	14.54

-1.1640	-1.3120	-1.6300	-0.7820	-0.4600	82.13	94.47	90.59	12.21
1.8100	2.0670	1.3450	1.7810	1.7840	133.80	104.60	117.90	6.82
1.5120	0.9470	-0.1550	1.4320	1.3780	113.80	99.19	115.70	5.80
-1.4430	-1.3480	0.1100	-1.8620	-1.9300	86.01	100.10	85.42	6.16
-1.4010	-0.7230	-0.0870	-0.9640	-0.9650	85.78	99.34	87.56	12.83
-0.3630	-0.4460	-1.1730	0.1740	0.3940	90.92	95.99	97.15	15.79
-1.4900	-1.2600	-0.8370	-1.1990	-1.0720	82.59	96.97	87.83	11.29
0.9020	1.4130	1.2710	0.7840	0.9470	125.40	104.70	110.00	4.06
-1.0580	-0.7100	-0.7530	-0.5180	-0.3880	86.14	97.01	90.79	14.11
-0.6000	-1.1500	-1.0980	-0.6510	-0.6810	86.54	96.10	94.51	6.36
-1.3020	1.1540	4.0710	-0.8530	-1.4220	112.70	112.30	89.63	13.95
0.0940	1.1040	1.7960	0.2170	0.1600	118.70	106.10	100.90	7.47
-2.3030	-1.5920	0.2170	-2.3240	-2.4200	78.48	100.30	78.69	10.40
-0.1660	1.3320	2.4690	0.2900	-0.0090	116.80	108.00	98.48	11.19
-0.8390	-0.2460	-0.0490	-0.3310	-0.3220	90.88	99.47	92.56	12.90
-1.4100	-0.9090	-0.4680	-0.9050	-0.8870	86.02	98.23	88.90	15.79
-1.2050	-0.5020	0.9990	-1.4320	-1.4430	97.30	103.20	88.93	6.23
1.4430	-1.2350	-2.6420	1.4790	0.4400	91.92	91.64	120.00	1.33
-0.6450	-0.6410	-0.4780	-0.4700	-0.4830	90.40	98.02	94.43	8.23
-2.2670	-2.6300	-2.2420	-2.0920	-1.7210	69.28	91.69	79.65	12.14
-0.1300	1.2660	2.3410	0.3970	-0.0350	109.50	106.80	98.98	15.79
-1.7660	-1.3660	-0.3700	-1.7180	-1.7430	84.26	100.50	84.25	21.96
-0.5290	-2.0680	-3.6410	-0.2970	0.4680	77.78	88.79	94.74	12.21
-1.0870	-2.2200	-0.7190	-2.4330	-2.6540	80.91	98.13	86.78	1.59
-0.0640	-0.0020	-0.2100	0.1930	0.1730	96.94	98.85	99.45	8.66
0.6800	0.9160	0.5190	0.9380	0.8390	109.10	101.40	106.20	8.89
-0.3740	-0.1040	0.9310	-1.2120	-0.8740	106.00	103.80	95.78	2.61
-1.6040	-3.5690	-1.8970	-3.0660	-3.4010	67.78	93.59	81.82	2.32
-1.8340	-0.8650	1.7800	-3.3460	-2.5840	96.32	106.70	80.24	3.20
-0.4290	1.2430	2.7220	-0.3560	-0.3000	124.00	109.70	95.80	7.47
-0.8460	0.4870	1.5910	-0.2570	-0.5630	99.67	105.10	92.79	15.52
3.5190	3.0540	0.3860	3.6430	3.6270	137.90	100.70	134.60	7.57
-0.7330	-3.6360	-5.0750	-1.0280	-0.3890	61.09	81.59	92.50	5.67
0.0840	-1.1120	-0.9730	-0.5280	-0.7570	92.57	96.82	101.00	3.07
-1.4780	0.1220	2.3370	-1.5060	-1.5640	105.20	108.00	85.76	8.33
-1.0420	0.0980	0.9770	-0.4550	-0.6590	94.72	103.10	91.21	16.38
0.0050	0.2290	0.5250	-0.0580	-0.0970	105.50	101.70	100.10	5.63
-3.0550	-1.9920	1.4730	-3.9110	-3.8220	82.23	104.70	72.57	6.88
-0.8450	-0.2640	1.0070	-1.2190	-1.1330	101.80	103.60	91.83	5.04
-0.9760	-0.5560	0.8740	-1.4890	-1.4140	99.19	103.30	89.84	4.98
0.1180	0.2530	-0.2470	0.5130	0.6010	99.12	99.28	101.00	22.16
-0.7590	-0.7080	-0.2420	-0.7200	-0.7940	89.49	98.75	92.58	7.61
-1.0410	-0.2100	0.6240	-0.6470	-0.7630	93.30	101.50	91.59	12.21
-0.4420	0.4570	1.0570	-0.0090	-0.1900	101.60	103.20	95.95	10.96
-0.4170	-1.2350	-0.4900	-1.2680	-1.3400	91.92	98.85	95.31	2.64
0.6470	2.1130	3.1270	1.1340	0.6360	125.00	110.30	105.30	15.16
-0.8530	0.0480	1.0260	-0.5540	-0.7320	98.07	103.10	92.02	9.77

-1.9690	-1.0070	0.1730	-1.5130	-1.6040	82.96	100.30	83.18	15.46
2.6810	-0.9220	-4.6630	2.3820	2.6200	83.93	83.44	131.10	3.96
0.6130	2.1370	2.7360	1.0020	0.7870	127.50	108.40	105.80	10.17
-2.9180	-2.8690	-1.6230	-2.8660	-2.6710	67.44	93.91	74.42	13.68
-0.4600	-0.1040	1.0310	-1.3250	-0.9500	106.00	104.20	94.84	2.68
-2.3100	-0.9820	0.7820	-1.0950	-2.1990	85.28	102.80	82.29	26.66
-0.9030	-0.2640	0.7410	-0.8090	-0.9410	96.00	102.10	91.27	8.16
-2.0090	-0.8470	0.8530	-1.6190	-1.8770	86.43	102.30	84.10	14.87
0.9550	0.0340	-1.3020	1.0010	1.0490	96.50	95.10	109.50	6.82
-1.2810	-3.3700	-2.3170	-2.5650	-2.8460	69.89	92.55	85.82	2.84
-2.7200	-0.2530	3.5570	-2.8720	-2.9880	100.00	111.40	77.40	10.10
1.5910	1.0810	0.2060	1.3160	1.3330	125.30	101.30	120.80	2.15
-0.8230	1.5420	4.0710	-0.3440	-0.9010	117.50	112.30	93.44	13.95
-2.1820	-1.7120	-0.3090	-2.1550	-2.1680	76.82	98.55	79.81	10.40
0.6560	1.6410	1.9300	0.3560	0.8190	131.00	107.30	107.60	3.33
-2.4170	-1.8160	-0.2690	-2.3280	-2.3570	77.83	98.71	80.30	11.45
1.8770	2.7040	2.0190	2.2570	2.0350	129.60	105.80	117.20	12.63
-1.1750	-0.6830	0.3070	-1.0540	-1.1530	91.09	100.50	89.93	8.49
2.7670	2.9360	1.8740	2.6470	2.5780	145.70	106.20	125.30	8.89
1.8620	-2.2220	-4.7380	1.8640	0.9420	79.03	84.79	124.70	1.72
-0.2700	-1.2700	-1.6420	-0.3810	-0.4040	85.40	94.41	97.45	5.63
-1.2110	0.0250	1.1290	-0.6420	-0.8780	93.95	103.60	89.68	15.62
2.4600	-0.4760	-3.6340	2.1930	2.2830	89.69	87.07	128.10	4.19
-2.9190	-1.2260	2.7200	-4.1280	-3.5770	92.61	110.00	70.29	5.86
-1.0500	-0.5410	-0.2830	-0.5000	-0.5130	89.81	98.90	91.79	17.23
-1.3120	-0.4650	1.1240	-1.3940	-1.4950	95.72	103.60	87.19	7.67
-1.8170	0.2680	2.9610	-1.3540	-1.8710	99.30	109.70	84.34	14.80
-1.5770	-0.5530	1.8300	-2.9070	-2.1790	100.20	106.90	83.06	3.27
0.0540	-0.2530	-0.1850	-0.1420	-0.1940	100.00	99.38	100.60	4.78
-1.5060	-1.3670	-1.2630	-0.9880	-0.8470	82.25	95.74	88.23	17.23
-0.6030	1.6370	3.8870	-0.1320	-0.6470	118.70	111.70	95.19	13.59
-0.3690	-0.3720	-0.9730	0.0740	0.2930	91.05	96.58	97.04	12.80
1.5470	1.6410	0.8810	1.3810	1.6190	131.00	103.60	119.00	2.58
-0.5890	-1.3400	-2.5760	-0.0880	0.4330	82.63	91.30	95.33	14.41
-1.6260	-0.1360	2.1600	-1.9420	-1.8180	103.30	107.40	85.21	6.49
1.5520	0.5710	-0.5110	1.2710	1.1590	114.10	98.43	118.60	3.50
1.2300	0.1750	-2.8560	2.1790	2.7610	100.50	91.60	116.30	14.31
-0.3690	1.2120	2.6610	-0.8810	-0.2810	127.40	110.10	96.07	4.19
0.8500	-0.3950	-1.1550	0.5570	0.3790	100.30	96.56	110.10	3.04
-0.6780	-2.2200	-1.2190	-1.6880	-2.2560	80.91	96.41	91.63	1.33
-0.5400	0.4210	1.0130	-0.2740	-0.3170	102.60	105.30	95.23	27.88
2.3040	0.6380	-1.6790	2.1190	2.1410	105.40	93.83	124.40	5.44
-2.1810	-1.0310	1.2140	-2.1510	-2.3480	87.68	103.30	81.85	10.00
-0.3340	-1.3710	-2.9870	0.1760	0.7460	80.91	88.90	97.01	12.40
-0.6010	-1.4690	-1.2030	-0.9440	-1.0570	83.03	95.45	93.79	5.27
-1.4470	-2.1440	-2.1790	-1.4320	-1.1520	72.50	92.05	86.12	8.53
-0.7020	-0.9390	-0.6230	-0.7490	-0.8080	87.04	97.45	93.04	7.01

-0.8280	0.8070	2.5100	-0.3160	-0.7790	104.80	107.30	93.45	14.87
-2.1940	-1.1300	0.4450	-1.8600	-1.9930	81.69	101.20	80.87	14.01
-2.1500	0.7770	4.8480	-1.8060	-2.5360	103.90	116.30	83.06	22.42
0.7700	0.3690	-1.0070	1.1290	1.2820	99.39	96.42	107.30	9.18
-2.8090	-2.0230	-0.0870	-2.7860	-2.8380	73.52	99.34	75.05	12.83
-1.1360	-1.0700	0.5810	-2.0800	-1.8970	94.46	102.40	87.63	3.50
-1.4080	-0.7630	0.3530	-1.2330	-1.3470	87.62	100.80	86.78	9.64
-0.5700	-0.3430	-0.6990	-0.0680	0.0720	89.74	97.19	94.90	12.40
-1.8850	-0.6290	1.7090	-2.1490	-2.1550	95.93	105.40	83.26	7.24
0.4090	0.7500	0.5700	0.6690	0.5690	107.90	101.30	104.00	7.87
-0.1110	0.2940	0.0700	0.3390	0.3580	97.96	99.82	99.11	13.16
-0.5450	-0.2720	-0.1890	-0.2230	-0.2430	91.93	98.92	94.88	9.64
-0.7080	0.6650	1.8380	-0.1610	-0.4830	103.10	105.90	93.79	13.68
-1.1460	1.5290	4.9160	-0.6150	-1.4950	114.40	114.70	91.09	18.74
-1.5130	0.3050	2.7390	-1.9910	-1.6350	110.30	109.60	85.76	5.63
0.6310	-0.1070	-0.9960	0.7010	0.6400	98.34	96.43	106.50	5.63
-0.2160	0.2370	0.8940	-0.3180	-0.3580	106.70	103.00	97.82	6.16
-0.0420	-0.1110	-0.6640	0.2800	0.3800	94.32	97.49	99.65	9.77
0.2120	-0.3580	-0.3690	-0.1020	-0.2070	99.19	98.62	102.40	4.55
2.0410	1.2960	-0.1960	1.9500	1.9200	123.70	99.83	124.50	2.84
0.6280	1.6170	1.7510	1.0710	0.8480	115.50	104.90	105.80	12.14
-4.1250	-3.0980	-0.0370	-4.2110	-4.4160	66.13	99.67	66.65	19.89
-0.7560	-0.6090	-1.0370	-0.1700	0.0070	87.76	96.01	93.52	15.16
0.3610	1.3410	1.9740	-0.0930	0.3790	129.50	107.60	104.30	3.53
1.8810	-2.1010	-4.2780	2.1430	0.6890	82.33	86.35	126.80	1.07
0.7010	2.2510	2.9530	0.8610	0.9050	140.50	110.50	106.70	7.84
-0.3820	-0.5440	-0.6400	-0.2440	-0.2580	89.96	97.38	96.29	7.90
-0.2020	-0.4740	0.2480	-0.8750	-0.8410	101.90	101.30	97.77	3.20
-0.5950	-0.6380	-0.9590	-0.1900	-0.0650	91.55	98.37	94.64	19.99
0.8790	1.2120	1.1880	0.4710	0.7730	127.40	104.80	111.00	2.87
-2.5360	0.8530	5.9600	-2.1970	-3.1360	109.10	118.20	80.22	17.86
-2.0380	-1.6230	-0.3700	-1.0980	-2.1550	81.83	100.50	81.82	21.96
-0.6480	-0.7160	-1.0240	-0.2480	-0.1180	90.74	98.14	94.15	19.79
0.6550	1.1830	0.8410	1.0990	0.9820	109.30	102.50	105.50	12.73
0.2750	0.7220	0.2850	0.7580	0.7830	106.90	102.80	103.10	25.02
-1.2070	-0.1400	0.9940	-0.7460	-0.9600	93.59	102.70	90.40	14.05
-1.3830	-1.2200	-0.3500	-1.3720	-1.4210	82.95	98.37	86.69	8.39
-1.6330	-2.2580	-2.2380	-1.5990	-1.3020	73.95	92.57	86.04	8.66
-3.1220	-2.5230	1.3690	-4.9810	-4.4510	78.55	105.10	68.35	4.16
4.2580	1.3680	-2.9870	3.7540	4.4340	113.50	88.90	136.00	12.40
1.6840	2.5420	2.6300	1.9500	1.5730	132.00	108.60	114.00	14.11
-0.1390	1.1420	2.0950	0.1780	-0.0050	116.50	106.90	98.69	9.25
-1.3530	0.1640	1.7270	-1.1540	-1.2760	98.07	106.30	89.85	30.41
-2.9460	-0.8290	3.1840	-3.6360	-3.4060	95.88	111.30	71.54	8.16
-1.8790	-1.6890	0.8810	-3.6710	-3.1000	86.69	103.60	78.75	2.58
-2.5160	-1.4250	1.4730	-3.1350	-3.0830	87.71	104.70	77.41	6.88
0.2820	1.2250	1.6840	0.5080	0.3650	110.40	105.80	102.30	28.76

-0.6730	-0.7300	-1.1560	-0.1830	-0.0550	89.01	97.06	93.71	19.27
-0.8230	1.1870	3.3440	-0.3440	-0.8600	111.60	109.90	93.44	13.95
-0.4290	-0.1540	0.3090	-0.3560	-0.4610	97.30	100.60	95.80	7.47
0.5920	2.1540	2.6890	0.8250	0.8480	133.20	109.10	105.90	7.24
-1.4780	0.6150	3.0590	-1.4190	-1.7910	105.10	113.20	87.01	36.78
-0.5530	-0.9330	-1.3510	-0.3350	-0.1860	86.20	95.35	95.26	8.59
-1.2940	-0.2690	0.7890	-0.7790	-0.9990	92.00	102.10	89.81	15.79
-1.1250	-0.2720	0.6220	-0.7490	-0.8800	91.93	101.70	89.73	11.19
-1.7070	-1.3030	-0.9000	-1.1770	-1.0920	81.32	96.53	85.77	17.33
-3.9690	-0.9390	4.7450	-4.0670	-4.6420	87.04	115.80	66.92	17.33
3.3450	1.4230	-1.7390	3.0890	3.3700	115.10	93.45	127.20	17.33
-1.9530	-0.2350	2.4720	-2.1100	-2.1490	100.70	108.50	81.27	8.53
0.7640	0.8220	0.1460	1.0430	0.9940	106.30	100.10	106.90	9.25
-0.2040	0.8010	1.6150	-0.0940	-0.1520	112.40	105.10	98.18	7.08
-1.6780	-0.6480	1.7120	-2.5850	-2.2040	98.61	106.20	83.31	4.48
-0.9630	-0.7300	-0.6910	-0.5530	-0.4890	89.01	98.83	91.16	20.81
-0.5140	0.6470	1.5680	-0.0230	-0.3040	102.40	104.40	95.91	14.14
0.9120	-1.1860	-1.9190	0.9070	-0.1580	92.47	94.00	113.10	0.97
-1.4780	-0.3460	1.5210	-1.5060	-1.6280	97.11	105.00	85.76	8.33
1.9880	3.0100	3.4580	2.1800	1.6650	140.20	111.40	116.30	15.92
0.0410	0.8400	1.4390	-0.0220	0.0310	115.30	105.00	100.40	5.57
-0.2330	-0.2590	-0.7950	0.2110	0.3370	95.61	98.96	97.91	20.52
-1.6020	-1.5690	-1.4820	-1.0990	-0.9430	80.58	95.02	87.53	18.25
0.4150	0.7460	0.7710	0.5050	0.4160	111.00	102.40	104.10	7.15
1.5820	1.1240	-0.3780	1.7660	1.8650	109.90	98.44	112.80	19.79
0.2880	-0.3230	-1.5560	0.5690	0.7900	90.22	94.21	102.60	8.89
-1.1170	-0.9220	-0.7400	-0.7950	-0.7020	83.93	97.04	89.68	10.46
-2.3610	-1.3620	0.5240	-1.2270	-2.3630	80.07	101.40	78.78	12.07
0.2040	0.5420	0.5240	0.4320	0.3310	105.50	101.20	102.00	7.80
2.0940	2.5580	1.9740	2.1290	1.9600	127.90	106.70	117.10	28.50
-1.1510	-0.7230	-0.4680	-0.7250	-0.6510	85.78	98.01	89.64	11.98
-0.2760	-0.1290	-0.0100	-0.0830	-0.1510	96.78	99.48	97.60	8.00
-0.0990	0.6050	0.7550	0.3840	0.2520	102.00	102.30	99.24	20.09
-1.1600	0.2680	1.8020	-0.7110	-1.0130	99.30	105.80	89.63	12.40
-2.6310	-0.5560	3.0910	-3.2020	-2.9980	99.19	111.00	74.51	8.03
0.8020	1.0920	1.1040	0.5000	0.6550	124.10	104.30	109.30	3.99
1.0480	0.4750	0.0110	0.6790	0.6330	114.80	100.40	113.00	3.01
-1.8200	0.0490	2.3290	-1.3060	-1.7670	94.42	107.70	84.83	17.33
2.4660	1.2730	-1.9140	2.7640	3.2350	109.90	93.56	122.40	13.16
0.5420	-0.8290	-1.1580	0.0540	-0.2280	95.88	96.17	106.70	2.91
-0.2120	-0.4010	0.3810	-1.0190	-0.8770	102.10	101.90	97.55	2.25
-0.7570	-1.0850	-0.3750	-1.0940	-1.1920	88.79	98.43	92.22	5.50
0.7350	0.9350	0.8310	0.5130	0.6350	118.70	103.30	108.40	3.66
-2.6890	-0.7230	2.6850	-2.7380	-3.0330	90.81	108.80	75.63	11.58
2.2600	-0.3310	-2.2700	2.1910	1.3450	103.70	92.36	130.50	1.36
0.8440	1.0920	1.0560	0.5410	0.6900	124.10	104.10	109.80	3.96
-2.0230	-1.8090	-0.1150	-2.3400	-2.4170	78.46	99.19	80.31	7.84

-1.6140	-0.2640	1.9680	-1.9840	-1.8520	101.80	106.80	85.17	6.23
4.0730	-0.3950	-4.5180	4.7890	3.2450	100.30	85.57	158.80	0.94
-1.8950	-3.4630	-1.5870	-3.3070	-3.4690	67.63	94.41	79.22	3.30
0.6950	-1.8810	-2.4610	0.3760	-0.6260	85.20	91.69	109.40	1.23
-2.2780	-1.9310	-0.6900	-2.2160	-2.1680	76.85	97.39	81.23	10.66
-2.0840	-1.0600	0.2020	-1.6210	-1.7270	83.14	100.50	82.90	18.58
-1.6660	-1.4430	-0.3440	-1.6690	-1.7080	82.78	98.44	85.69	8.43
-1.3040	-1.8070	-1.3250	-1.4370	-1.4190	79.51	95.39	88.30	6.88
0.8940	2.3340	2.9230	1.1590	1.0370	138.30	110.00	108.10	9.25
0.9440	0.2940	-0.9620	1.1540	1.1420	101.30	96.53	109.50	6.55
-0.6830	-0.4010	0.9310	-1.6750	-1.2970	102.10	103.80	92.30	2.61
1.4220	0.1740	-0.9300	1.2370	0.9790	108.60	97.37	117.80	2.35
-2.7200	-0.0930	3.8150	-3.3960	-2.9410	105.30	113.20	75.69	7.01
-0.7130	0.1100	1.2360	-0.7560	-0.7920	104.20	103.90	93.52	6.55

Stunting	filter_$	Wasting	Underweig	Breastfeed	Interval	Interval2	Agemother	House
0.00	0	1.00	1.00	2.00	47.00	1.00	2.00	1.00
0.00	0	1.00	1.00	2.00	31.00	1.00	2.00	0.00
0.00	1	1.00	1.00	1.00	8.00	0.00	2.00	1.00
0.00	1	1.00	1.00	1.00	15.00	0.00	2.00	0.00
0.00	0	1.00	1.00	1.00	61.00	1.00	2.00	1.00
0.00	0	1.00	1.00	1.00	46.00	1.00	2.00	2.00
0.00	1	1.00	1.00	1.00	8.00	0.00	2.00	2.00
0.00	1	1.00	1.00	1.00	10.00	0.00	3.00	2.00
0.00	0	1.00	1.00	1.00	72.00	1.00	2.00	0.00
0.00	0	1.00	1.00	1.00	15.00	0.00	2.00	1.00
0.00	1	1.00	1.00	1.00	16.00	0.00	2.00	0.00
0.00	0	1.00	1.00	1.00	38.00	1.00	1.00	1.00
0.00	1	1.00	1.00	1.00	19.00	0.00	1.00	1.00
0.00	0	1.00	2.00	1.00	15.00	0.00	2.00	2.00
1.00	0	1.00	2.00	1.00	95.00	1.00	2.00	0.00
0.00	1	1.00	1.00	1.00	16.00	0.00	2.00	0.00
0.00	0	1.00	1.00	1.00	37.00	1.00	1.00	0.00
1.00	1	1.00	2.00	1.00	12.00	0.00	1.00	2.00
0.00	1	1.00	1.00	1.00	21.00	0.00	3.00	1.00
0.00	0	1.00	1.00	1.00	84.00	1.00	2.00	2.00
0.00	0	1.00	1.00	2.00	76.00	1.00	1.00	1.00
0.00	1	1.00	1.00	1.00	14.00	0.00	1.00	1.00
0.00	0	1.00	1.00	2.00	64.00	1.00	3.00	1.00
0.00	1	2.00	1.00	1.00	10.00	0.00	3.00	2.00
1.00	1	1.00	1.00	1.00	53.00	1.00	2.00	2.00
1.00	0	1.00	1.00	2.00	80.00	1.00	2.00	1.00
0.00	0	1.00	1.00	1.00	44.00	1.00	3.00	0.00
0.00	1	1.00	1.00	1.00	15.00	0.00	2.00	1.00
0.00	0	1.00	1.00	2.00	83.00	1.00	2.00	1.00
0.00	0	1.00	1.00	1.00	12.00	0.00	2.00	2.00
0.00	1	1.00	1.00	1.00	13.00	0.00	1.00	1.00
0.00	1	1.00	1.00	1.00	17.00	0.00	2.00	1.00
0.00	0	1.00	1.00	2.00	61.00	1.00	2.00	2.00
1.00	0	1.00	1.00	2.00	28.00	1.00	2.00	1.00
0.00	1	1.00	1.00	1.00	12.00	0.00	3.00	1.00
0.00	1	1.00	1.00	1.00	13.00	0.00	2.00	1.00
1.00	0	1.00	1.00	2.00	25.00	1.00	2.00	1.00
0.00	1	1.00	1.00	1.00	21.00	0.00	1.00	2.00
0.00	1	1.00	2.00	1.00	10.00	0.00	1.00	1.00
0.00	1	1.00	1.00	1.00	13.00	0.00	3.00	1.00
0.00	0	1.00	1.00	2.00	47.00	1.00	2.00	1.00
0.00	1	1.00	1.00	1.00	7.00	0.00	2.00	1.00
0.00	1	1.00	1.00	1.00	30.00	1.00	2.00	1.00
1.00	0	1.00	1.00	1.00	49.00	1.00	3.00	1.00
0.00	0	1.00	1.00	2.00	40.00	1.00	2.00	1.00
0.00	0	1.00	1.00	1.00	35.00	1.00	3.00	2.00

1.00	0	1.00	1.00	2.00	21.00	0.00	2.00	1.00
0.00	1	1.00	1.00	1.00	15.00	0.00	1.00	0.00
0.00	0	2.00	2.00	1.00	51.00	1.00	2.00	0.00
0.00	0	1.00	1.00	1.00	66.00	1.00	1.00	0.00
0.00	1	2.00	1.00	1.00	15.00	0.00	2.00	1.00
0.00	0	1.00	1.00	1.00	60.00	1.00	1.00	2.00
1.00	1	1.00	1.00	1.00	12.00	0.00	3.00	2.00
0.00	1	1.00	1.00	1.00	12.00	0.00	1.00	0.00
0.00	0	1.00	1.00	2.00	48.00	1.00	1.00	0.00
0.00	1	1.00	1.00	1.00	19.00	0.00	3.00	0.00
0.00	1	1.00	1.00	1.00	13.00	0.00	1.00	1.00
1.00	1	1.00	1.00	1.00	10.00	0.00	1.00	1.00
0.00	0	2.00	1.00	2.00	46.00	1.00	3.00	1.00
0.00	0	1.00	1.00	2.00	29.00	1.00	1.00	2.00
0.00	1	1.00	1.00	1.00	15.00	0.00	1.00	1.00
1.00	0	1.00	1.00	2.00	29.00	1.00	3.00	1.00
0.00	1	1.00	1.00	1.00	18.00	0.00	1.00	1.00
1.00	0	1.00	1.00	1.00	23.00	0.00	2.00	0.00
0.00	0	1.00	1.00	2.00	45.00	1.00	2.00	0.00
0.00	1	2.00	1.00	1.00	16.00	0.00	3.00	0.00
0.00	0	2.00	1.00	2.00	30.00	1.00	3.00	0.00
0.00	0	1.00	1.00	1.00	30.00	1.00	3.00	1.00
0.00	1	1.00	1.00	1.00	13.00	0.00	1.00	0.00
0.00	0	2.00	1.00	1.00	35.00	1.00	2.00	1.00
0.00	1	1.00	1.00	1.00	8.00	0.00	2.00	0.00
0.00	1	1.00	1.00	1.00	16.00	0.00	3.00	0.00
0.00	0	1.00	1.00	1.00	27.00	1.00	2.00	2.00
0.00	1	2.00	1.00	1.00	20.00	0.00	1.00	0.00
0.00	1	1.00	1.00	1.00	20.00	0.00	1.00	0.00
0.00	1	1.00	1.00	1.00	33.00	1.00	3.00	1.00
0.00	1	1.00	1.00	1.00	15.00	0.00	2.00	0.00
0.00	0	1.00	1.00	2.00	18.00	0.00	3.00	1.00
0.00	1	2.00	1.00	1.00	18.00	0.00	2.00	1.00
0.00	0	1.00	1.00	2.00	57.00	1.00	3.00	1.00
0.00	0	1.00	1.00	2.00	55.00	1.00	3.00	0.00
0.00	1	1.00	1.00	1.00	9.00	0.00	3.00	2.00
0.00	0	1.00	1.00	1.00	10.00	0.00	3.00	1.00
1.00	0	1.00	1.00	1.00	46.00	1.00	2.00	2.00
0.00	1	1.00	1.00	1.00	16.00	0.00	3.00	0.00
1.00	0	1.00	1.00	1.00	25.00	1.00	2.00	0.00
1.00	0	1.00	2.00	1.00	36.00	1.00	2.00	0.00
1.00	1	1.00	2.00	1.00	10.00	0.00	2.00	0.00
0.00	0	1.00	1.00	1.00	33.00	1.00	2.00	0.00
0.00	1	2.00	1.00	1.00	10.00	0.00	1.00	0.00
0.00	0	1.00	1.00	1.00	61.00	1.00	2.00	0.00
0.00	0	2.00	1.00	2.00	21.00	0.00	2.00	0.00
0.00	1	2.00	1.00	1.00	18.00	0.00	3.00	0.00

0.00	1	2.00	2.00	1.00	34.00	1.00	3.00	0.00
0.00	1	1.00	1.00	1.00	14.00	0.00	2.00	0.00
0.00	0	2.00	2.00	2.00	43.00	1.00	1.00	0.00
0.00	1	1.00	1.00	1.00	16.00	0.00	2.00	1.00
1.00	0	1.00	1.00	1.00	31.00	1.00	2.00	1.00
0.00	1	1.00	1.00	1.00	17.00	0.00	2.00	1.00
0.00	0	1.00	1.00	2.00	48.00	1.00	2.00	1.00
0.00	1	1.00	1.00	1.00	15.00	0.00	1.00	1.00
1.00	0	1.00	1.00	1.00	57.00	1.00	2.00	0.00
0.00	0	2.00	1.00	1.00	11.00	0.00	2.00	0.00
0.00	0	1.00	1.00	2.00	39.00	1.00	1.00	1.00
0.00	1	1.00	1.00	1.00	17.00	0.00	3.00	1.00
0.00	0	2.00	1.00	2.00	53.00	1.00	3.00	0.00
0.00	0	1.00	1.00	2.00	38.00	1.00	3.00	0.00
0.00	0	1.00	1.00	2.00	48.00	1.00	2.00	1.00
0.00	1	1.00	1.00	1.00	15.00	0.00	2.00	1.00
0.00	1	1.00	1.00	1.00	20.00	0.00	3.00	1.00
0.00	1	1.00	1.00	1.00	18.00	0.00	3.00	1.00
0.00	0	2.00	1.00	1.00	44.00	1.00	1.00	1.00
0.00	1	1.00	1.00	1.00	15.00	0.00	1.00	0.00
1.00	0	1.00	1.00	1.00	18.00	0.00	1.00	1.00
1.00	0	1.00	1.00	1.00	38.00	1.00	1.00	1.00
0.00	1	2.00	1.00	1.00	19.00	0.00	3.00	1.00
0.00	0	1.00	1.00	2.00	36.00	1.00	2.00	0.00
0.00	0	1.00	1.00	2.00	42.00	1.00	2.00	0.00
0.00	1	1.00	1.00	1.00	19.00	0.00	3.00	1.00
0.00	1	2.00	1.00	1.00	29.00	1.00	1.00	0.00
0.00	0	1.00	1.00	2.00	25.00	1.00	3.00	0.00
0.00	1	1.00	1.00	1.00	18.00	0.00	2.00	0.00
1.00	0	1.00	1.00	1.00	41.00	1.00	1.00	0.00
0.00	1	1.00	1.00	1.00	16.00	0.00	2.00	1.00
0.00	1	1.00	1.00	1.00	20.00	0.00	2.00	1.00
0.00	0	1.00	1.00	2.00	38.00	1.00	2.00	1.00
0.00	1	1.00	1.00	1.00	19.00	0.00	2.00	1.00
1.00	0	1.00	1.00	1.00	68.00	1.00	2.00	0.00
0.00	0	2.00	1.00	2.00	15.00	0.00	1.00	0.00
0.00	1	1.00	1.00	1.00	21.00	0.00	1.00	0.00
0.00	0	1.00	1.00	1.00	40.00	1.00	3.00	0.00
0.00	1	1.00	1.00	1.00	13.00	0.00	2.00	2.00
0.00	0	2.00	1.00	1.00	32.00	1.00	2.00	0.00
0.00	1	1.00	1.00	1.00	16.00	0.00	2.00	2.00
0.00	0	1.00	1.00	1.00	38.00	1.00	2.00	1.00
0.00	1	1.00	1.00	1.00	21.00	0.00	2.00	2.00
0.00	1	1.00	1.00	1.00	12.00	0.00	2.00	2.00
0.00	0	1.00	1.00	1.00	32.00	1.00	1.00	2.00
0.00	1	1.00	1.00	1.00	15.00	0.00	2.00	0.00
0.00	1	2.00	2.00	1.00	12.00	0.00	2.00	0.00

1.00	0	1.00	1.00	1.00	61.00	1.00	2.00	0.00
0.00	1	2.00	1.00	1.00	15.00	0.00	2.00	1.00
0.00	0	1.00	1.00	2.00	26.00	1.00	2.00	1.00
0.00	1	1.00	1.00	1.00	32.00	1.00	1.00	2.00
0.00	1	1.00	1.00	1.00	46.00	1.00	2.00	2.00
0.00	1	2.00	1.00	1.00	15.00	0.00	2.00	2.00
0.00	1	1.00	1.00	1.00	19.00	0.00	3.00	2.00
0.00	1	1.00	1.00	1.00	41.00	1.00	1.00	2.00
0.00	1	1.00	1.00	1.00	15.00	0.00	2.00	2.00
0.00	0	1.00	1.00	1.00	21.00	1.00	2.00	2.00
0.00	1	2.00	1.00	1.00	19.00	0.00	3.00	2.00
0.00	0	1.00	1.00	1.00	46.00	1.00	2.00	2.00
0.00	1	1.00	1.00	1.00	29.00	1.00	2.00	2.00
0.00	1	1.00	1.00	1.00	14.00	0.00	2.00	1.00
0.00	0	1.00	1.00	2.00	49.00	1.00	3.00	1.00
0.00	0	1.00	1.00	1.00	14.00	0.00	2.00	1.00
0.00	0	1.00	2.00	2.00	12.00	0.00	3.00	1.00
0.00	1	1.00	1.00	1.00	13.00	0.00	3.00	1.00
0.00	1	2.00	2.00	1.00	79.00	1.00	2.00	1.00
0.00	1	1.00	1.00	1.00	16.00	0.00	3.00	1.00
0.00	0	1.00	1.00	1.00	32.00	1.00	2.00	1.00
1.00	1	1.00	1.00	1.00	15.00	0.00	2.00	1.00
0.00	1	1.00	1.00	1.00	16.00	0.00	3.00	1.00
1.00	0	1.00	2.00	2.00	37.00	1.00	1.00	1.00
0.00	0	1.00	1.00	2.00	48.00	1.00	3.00	2.00
0.00	1	1.00	1.00	1.00	15.00	0.00	1.00	1.00
0.00	0	1.00	1.00	2.00	70.00	1.00	3.00	1.00
1.00	1	1.00	1.00	1.00	15.00	0.00	1.00	1.00
0.00	0	1.00	1.00	1.00	45.00	1.00	1.00	1.00
0.00	1	1.00	1.00	1.00	13.00	0.00	2.00	0.00
0.00	0	1.00	1.00	2.00	35.00	1.00	2.00	0.00
0.00	1	1.00	1.00	1.00	20.00	0.00	1.00	2.00
0.00	0	1.00	1.00	2.00	36.00	1.00	2.00	0.00
0.00	0	1.00	1.00	1.00	54.00	1.00	1.00	0.00
0.00	1	2.00	1.00	1.00	16.00	0.00	1.00	0.00
0.00	0	1.00	1.00	1.00	19.00	0.00	2.00	1.00
0.00	1	1.00	1.00	1.00	15.00	0.00	1.00	1.00
0.00	1	2.00	1.00	1.00	19.00	0.00	2.00	2.00
0.00	1	2.00	2.00	1.00	15.00	0.00	2.00	1.00
0.00	0	1.00	1.00	1.00	36.00	1.00	2.00	2.00
1.00	1	1.00	1.00	1.00	17.00	0.00	3.00	0.00
0.00	0	1.00	1.00	1.00	27.00	1.00	1.00	0.00
0.00	1	1.00	1.00	1.00	13.00	0.00	1.00	0.00
0.00	1	1.00	1.00	1.00	18.00	0.00	2.00	0.00
0.00	0	1.00	1.00	2.00	61.00	1.00	3.00	0.00
0.00	0	2.00	1.00	2.00	29.00	1.00	2.00	0.00
1.00	1	1.00	1.00	1.00	17.00	0.00	2.00	1.00

1.00	0	1.00	1.00	1.00	25.00	1.00	2.00	2.00
0.00	1	1.00	1.00	1.00	16.00	0.00	2.00	0.00
0.00	1	1.00	1.00	1.00	11.00	0.00	1.00	0.00
0.00	1	1.00	1.00	1.00	15.00	0.00	3.00	0.00
0.00	0	1.00	1.00	1.00	46.00	1.00	2.00	0.00
0.00	1	1.00	1.00	1.00	23.00	0.00	1.00	0.00
0.00	1	1.00	1.00	1.00	10.00	0.00	3.00	1.00
0.00	0	1.00	1.00	2.00	40.00	1.00	1.00	1.00
0.00	0	1.00	1.00	2.00	46.00	1.00	2.00	1.00
0.00	1	2.00	1.00	1.00	14.00	0.00	3.00	1.00
1.00	1	1.00	1.00	1.00	54.00	1.00	2.00	1.00
0.00	1	1.00	1.00	1.00	13.00	0.00	2.00	1.00
0.00	0	2.00	2.00	1.00	41.00	1.00	1.00	1.00
0.00	1	2.00	1.00	1.00	12.00	0.00	1.00	1.00
0.00	0	1.00	1.00	1.00	28.00	1.00	2.00	1.00
0.00	1	1.00	1.00	1.00	41.00	1.00	2.00	1.00
0.00	1	1.00	1.00	1.00	17.00	0.00	3.00	1.00
0.00	0	2.00	2.00	1.00	36.00	1.00	2.00	0.00
0.00	1	2.00	1.00	1.00	17.00	0.00	3.00	0.00
0.00	0	1.00	1.00	2.00	35.00	1.00	3.00	0.00
1.00	0	1.00	1.00	1.00	59.00	1.00	2.00	0.00
0.00	1	2.00	1.00	1.00	17.00	0.00	2.00	1.00
0.00	0	1.00	1.00	2.00	48.00	1.00	2.00	1.00
0.00	1	2.00	2.00	1.00	20.00	0.00	2.00	0.00
0.00	1	1.00	1.00	1.00	28.00	1.00	2.00	2.00
0.00	1	1.00	1.00	1.00	13.00	0.00	2.00	2.00
0.00	1	2.00	1.00	1.00	19.00	0.00	2.00	2.00
0.00	0	1.00	1.00	1.00	38.00	1.00	1.00	2.00
0.00	1	1.00	1.00	1.00	11.00	0.00	2.00	1.00
1.00	0	1.00	1.00	2.00	60.00	1.00	2.00	2.00
0.00	1	1.00	1.00	1.00	13.00	0.00	1.00	0.00
0.00	1	2.00	1.00	1.00	52.00	1.00	2.00	2.00
0.00	0	2.00	2.00	2.00	15.00	0.00	2.00	0.00
0.00	1	1.00	1.00	1.00	15.00	0.00	2.00	2.00
0.00	0	2.00	2.00	1.00	64.00	1.00	2.00	0.00
0.00	1	2.00	1.00	1.00	19.00	0.00	2.00	0.00
0.00	1	1.00	1.00	1.00	68.00	1.00	2.00	0.00
0.00	0	1.00	1.00	1.00	92.00	1.00	2.00	0.00
0.00	1	1.00	1.00	1.00	18.00	0.00	2.00	0.00
0.00	1	1.00	1.00	1.00	10.00	0.00	3.00	1.00
0.00	0	1.00	1.00	2.00	39.00	1.00	1.00	0.00
1.00	0	2.00	2.00	2.00	14.00	0.00	2.00	1.00
0.00	1	1.00	1.00	1.00	13.00	0.00	1.00	1.00
0.00	0	1.00	1.00	1.00	35.00	1.00	3.00	1.00
0.00	1	1.00	1.00	1.00	11.00	0.00	1.00	1.00
0.00	1	1.00	1.00	1.00	48.00	1.00	1.00	0.00
0.00	0	1.00	1.00	2.00	54.00	1.00	1.00	0.00

0.00	1	1.00	1.00	1.00	19.00	0.00	1.00	2.00
0.00	1	1.00	1.00	1.00	18.00	0.00	2.00	0.00
0.00	0	1.00	1.00	1.00	13.00	0.00	1.00	0.00
0.00	0	2.00	2.00	2.00	76.00	1.00	3.00	1.00
0.00	1	1.00	1.00	1.00	16.00	0.00	1.00	0.00
0.00	0	1.00	1.00	2.00	38.00	1.00	1.00	0.00
0.00	0	1.00	1.00	2.00	41.00	1.00	2.00	0.00
0.00	1	2.00	1.00	1.00	13.00	0.00	1.00	0.00
0.00	0	1.00	1.00	2.00	36.00	1.00	3.00	1.00
0.00	1	1.00	1.00	1.00	11.00	0.00	3.00	2.00
0.00	0	1.00	1.00	1.00	69.00	1.00	2.00	2.00
0.00	1	1.00	1.00	1.00	13.00	0.00	3.00	2.00
0.00	1	2.00	1.00	1.00	51.00	1.00	2.00	2.00
0.00	1	1.00	1.00	1.00	15.00	0.00	1.00	0.00
0.00	1	1.00	1.00	1.00	10.00	0.00	2.00	0.00
0.00	0	1.00	1.00	1.00	46.00	1.00	2.00	0.00
0.00	1	1.00	1.00	1.00	15.00	0.00	1.00	0.00
0.00	0	2.00	2.00	2.00	51.00	1.00	2.00	2.00
0.00	1	1.00	1.00	1.00	12.00	0.00	3.00	1.00
0.00	0	1.00	1.00	2.00	35.00	1.00	3.00	1.00
0.00	1	1.00	1.00	1.00	18.00	0.00	3.00	1.00
0.00	1	1.00	1.00	1.00	16.00	0.00	2.00	0.00
0.00	0	2.00	2.00	1.00	21.00	0.00	3.00	0.00
0.00	0	2.00	2.00	2.00	52.00	1.00	2.00	0.00
0.00	0	1.00	1.00	2.00	14.00	0.00	2.00	0.00
0.00	1	1.00	1.00	1.00	13.00	0.00	2.00	0.00
0.00	0	1.00	1.00	1.00	28.00	1.00	1.00	0.00
1.00	1	1.00	1.00	1.00	11.00	0.00	2.00	0.00
0.00	0	1.00	1.00	1.00	75.00	1.00	3.00	0.00
0.00	1	1.00	1.00	1.00	17.00	0.00	2.00	0.00
0.00	0	1.00	1.00	1.00	30.00	1.00	3.00	1.00
1.00	1	1.00	2.00	1.00	9.00	0.00	2.00	0.00
1.00	0	2.00	2.00	1.00	44.00	1.00	3.00	1.00
1.00	0	1.00	1.00	2.00	34.00	1.00	2.00	0.00
0.00	1	1.00	1.00	1.00	17.00	0.00	1.00	0.00
0.00	1	2.00	1.00	1.00	18.00	0.00	2.00	2.00
0.00	0	1.00	1.00	2.00	41.00	1.00	2.00	1.00
0.00	1	1.00	1.00	1.00	14.00	0.00	2.00	0.00
0.00	1	1.00	1.00	1.00	21.00	0.00	1.00	0.00
0.00	0	1.00	1.00	1.00	48.00	1.00	2.00	1.00
0.00	1	1.00	1.00	1.00	24.00	1.00	1.00	0.00
0.00	1	1.00	1.00	1.00	17.00	0.00	1.00	2.00
0.00	1	1.00	1.00	1.00	39.00	1.00	2.00	0.00
0.00	0	1.00	1.00	1.00	66.00	1.00	2.00	0.00
0.00	1	1.00	1.00	1.00	15.00	0.00	2.00	0.00
0.00	0	2.00	1.00	1.00	36.00	1.00	2.00	1.00
0.00	1	1.00	1.00	1.00	24.00	1.00	2.00	1.00

0.00	1	1.00	1.00	1.00	18.00	0.00	2.00	2.00
0.00	0	1.00	1.00	1.00	72.00	1.00	2.00	1.00
0.00	1	1.00	1.00	1.00	20.00	0.00	2.00	2.00
0.00	0	2.00	1.00	1.00	26.00	1.00	2.00	2.00
0.00	0	2.00	1.00	1.00	38.00	1.00	2.00	0.00
0.00	1	1.00	1.00	1.00	14.00	0.00	2.00	0.00
0.00	1	1.00	1.00	1.00	17.00	0.00	2.00	0.00
0.00	0	1.00	1.00	1.00	43.00	1.00	1.00	0.00
0.00	0	1.00	1.00	1.00	12.00	0.00	3.00	2.00
1.00	0	1.00	1.00	1.00	41.00	1.00	1.00	0.00
0.00	1	1.00	1.00	1.00	13.00	0.00	1.00	2.00
0.00	0	1.00	1.00	1.00	53.00	1.00	2.00	0.00
0.00	1	1.00	1.00	1.00	16.00	0.00	1.00	1.00
0.00	0	2.00	1.00	1.00	33.00	1.00	1.00	2.00
1.00	1	1.00	1.00	1.00	12.00	0.00	1.00	1.00
0.00	0	1.00	1.00	1.00	76.00	1.00	2.00	2.00
0.00	1	1.00	1.00	1.00	65.00	1.00	3.00	1.00
0.00	1	1.00	1.00	1.00	21.00	0.00	2.00	1.00
0.00	0	1.00	1.00	1.00	44.00	1.00	2.00	1.00
0.00	0	1.00	1.00	1.00	38.00	1.00	1.00	1.00
0.00	1	1.00	1.00	1.00	9.00	0.00	2.00	1.00
0.00	0	1.00	1.00	1.00	86.00	1.00	2.00	0.00
0.00	1	2.00	1.00	1.00	29.00	1.00	2.00	2.00
0.00	0	2.00	2.00	2.00	16.00	0.00	3.00	1.00
0.00	1	1.00	1.00	1.00	22.00	0.00	1.00	0.00
0.00	1	1.00	1.00	1.00	26.00	1.00	2.00	2.00
0.00	0	1.00	1.00	2.00	11.00	0.00	1.00	0.00
0.00	0	1.00	1.00	2.00	51.00	1.00	2.00	0.00
0.00	1	2.00	1.00	1.00	30.00	1.00	3.00	2.00
0.00	0	1.00	1.00	1.00	19.00	0.00	1.00	2.00
0.00	1	1.00	1.00	1.00	21.00	0.00	2.00	2.00
0.00	1	1.00	1.00	1.00	19.00	0.00	2.00	2.00
0.00	1	1.00	1.00	1.00	26.00	1.00	2.00	2.00
0.00	0	1.00	1.00	1.00	44.00	1.00	3.00	2.00
1.00	1	1.00	2.00	1.00	14.00	0.00	2.00	0.00
0.00	0	1.00	1.00	2.00	46.00	1.00	2.00	1.00
0.00	0	1.00	1.00	2.00	8.00	0.00	1.00	2.00
0.00	1	1.00	1.00	1.00	13.00	0.00	2.00	0.00
0.00	1	1.00	1.00	1.00	17.00	0.00	2.00	0.00
0.00	0	1.00	1.00	1.00	44.00	1.00	2.00	1.00
0.00	0	1.00	1.00	1.00	52.00	1.00	2.00	0.00
0.00	1	1.00	2.00	1.00	15.00	0.00	2.00	1.00
0.00	0	1.00	1.00	1.00	45.00	1.00	2.00	2.00
0.00	1	1.00	1.00	1.00	51.00	1.00	3.00	0.00
0.00	1	1.00	1.00	1.00	35.00	1.00	2.00	0.00
0.00	1	1.00	1.00	1.00	20.00	0.00	2.00	0.00
0.00	1	1.00	1.00	1.00	12.00	0.00	3.00	0.00

0.00	0	1.00	1.00	1.00	50.00	1.00	2.00	0.00
0.00	0	1.00	1.00	1.00	15.00	0.00	2.00	0.00
0.00	0	1.00	1.00	1.00	32.00	1.00	2.00	2.00
0.00	1	1.00	1.00	1.00	36.00	1.00	1.00	0.00
0.00	1	1.00	1.00	1.00	11.00	0.00	1.00	0.00
0.00	0	1.00	1.00	1.00	48.00	1.00	2.00	0.00
0.00	0	1.00	1.00	1.00	22.00	0.00	2.00	2.00
0.00	1	1.00	1.00	1.00	21.00	0.00	2.00	0.00
0.00	0	1.00	1.00	1.00	36.00	1.00	2.00	0.00
0.00	0	1.00	1.00	1.00	15.00	0.00	1.00	0.00
0.00	1	1.00	1.00	1.00	14.00	0.00	2.00	0.00
0.00	0	1.00	1.00	1.00	45.00	1.00	2.00	0.00
0.00	1	2.00	1.00	1.00	12.00	0.00	2.00	0.00
0.00	1	1.00	1.00	1.00	12.00	0.00	2.00	0.00
0.00	0	1.00	1.00	1.00	9.00	0.00	3.00	0.00
0.00	0	1.00	1.00	2.00	23.00	0.00	2.00	0.00
0.00	0	1.00	1.00	2.00	67.00	1.00	2.00	2.00
1.00	1	1.00	1.00	1.00	16.00	0.00	2.00	0.00
0.00	0	1.00	1.00	1.00	81.00	1.00	2.00	0.00
1.00	0	2.00	2.00	1.00	33.00	1.00	2.00	0.00
0.00	1	1.00	1.00	1.00	12.00	0.00	2.00	0.00
0.00	1	1.00	1.00	1.00	25.00	1.00	2.00	1.00
1.00	0	1.00	2.00	1.00	13.00	0.00	2.00	2.00
0.00	0	2.00	2.00	1.00	21.00	0.00	2.00	2.00
0.00	1	1.00	1.00	1.00	12.00	0.00	2.00	2.00
0.00	0	1.00	1.00	1.00	47.00	1.00	3.00	2.00
0.00	1	1.00	1.00	1.00	46.00	1.00	2.00	1.00
0.00	1	2.00	2.00	1.00	18.00	0.00	3.00	2.00
0.00	1	2.00	1.00	1.00	52.00	1.00	2.00	2.00
0.00	1	1.00	1.00	1.00	13.00	0.00	2.00	2.00
0.00	1	1.00	1.00	1.00	11.00	0.00	1.00	2.00
0.00	1	1.00	1.00	1.00	13.00	0.00	2.00	2.00
1.00	0	1.00	2.00	1.00	21.00	0.00	2.00	0.00
0.00	1	1.00	1.00	1.00	26.00	1.00	2.00	2.00
0.00	0	1.00	1.00	1.00	52.00	1.00	2.00	2.00
0.00	1	1.00	1.00	1.00	10.00	0.00	2.00	2.00
0.00	1	1.00	1.00	1.00	14.00	0.00	2.00	2.00
0.00	1	2.00	1.00	1.00	19.00	0.00	2.00	2.00
0.00	1	1.00	1.00	1.00	19.00	0.00	2.00	2.00
0.00	1	1.00	1.00	1.00	16.00	0.00	2.00	2.00
0.00	0	1.00	1.00	2.00	49.00	1.00	3.00	1.00
0.00	1	1.00	1.00	1.00	16.00	0.00	2.00	0.00
0.00	1	1.00	1.00	1.00	85.00	1.00	2.00	2.00
0.00	0	1.00	1.00	1.00	58.00	1.00	1.00	0.00
0.00	1	1.00	1.00	1.00	20.00	0.00	1.00	0.00
0.00	1	1.00	1.00	1.00	14.00	0.00	3.00	2.00
0.00	1	1.00	1.00	1.00	13.00	0.00	2.00	2.00

0.00	0	1.00	1.00	1.00	45.00	1.00	2.00	2.00
1.00	1	1.00	1.00	1.00	24.00	1.00	2.00	1.00
0.00	1	1.00	1.00	1.00	16.00	0.00	1.00	2.00
0.00	1	2.00	2.00	1.00	31.00	1.00	2.00	1.00
0.00	1	1.00	1.00	1.00	22.00	0.00	1.00	2.00
0.00	1	1.00	1.00	1.00	73.00	1.00	1.00	2.00
0.00	1	1.00	1.00	1.00	14.00	0.00	2.00	1.00
0.00	0	1.00	1.00	2.00	36.00	1.00	3.00	1.00
0.00	0	1.00	1.00	2.00	12.00	0.00	2.00	1.00
1.00	0	2.00	2.00	1.00	44.00	1.00	3.00	1.00
0.00	1	2.00	1.00	1.00	14.00	0.00	3.00	1.00
0.00	0	1.00	1.00	2.00	45.00	1.00	3.00	1.00
0.00	1	1.00	1.00	1.00	16.00	0.00	3.00	1.00
0.00	1	2.00	1.00	1.00	24.00	1.00	3.00	0.00
0.00	1	1.00	1.00	1.00	22.00	0.00	2.00	1.00
0.00	0	2.00	1.00	2.00	24.00	1.00	2.00	1.00
0.00	1	1.00	1.00	1.00	15.00	0.00	3.00	1.00
0.00	1	1.00	1.00	1.00	15.00	0.00	2.00	1.00
0.00	1	1.00	1.00	1.00	16.00	0.00	3.00	1.00
1.00	1	1.00	2.00	1.00	29.00	1.00	2.00	1.00
0.00	1	1.00	1.00	1.00	14.00	0.00	2.00	1.00
0.00	1	1.00	1.00	1.00	35.00	1.00	2.00	1.00
1.00	1	1.00	1.00	1.00	11.00	0.00	1.00	1.00
0.00	0	2.00	1.00	1.00	21.00	0.00	2.00	2.00
0.00	0	1.00	1.00	2.00	35.00	1.00	2.00	2.00
0.00	1	1.00	1.00	1.00	18.00	0.00	1.00	1.00
0.00	1	1.00	1.00	1.00	12.00	0.00	2.00	1.00
0.00	1	2.00	1.00	1.00	22.00	0.00	2.00	0.00
0.00	1	1.00	1.00	1.00	47.00	1.00	1.00	2.00
0.00	0	1.00	1.00	1.00	43.00	1.00	2.00	2.00
0.00	0	1.00	1.00	2.00	16.00	0.00	2.00	1.00
0.00	1	1.00	1.00	1.00	9.00	0.00	2.00	1.00
0.00	1	1.00	1.00	1.00	46.00	1.00	1.00	1.00
1.00	0	1.00	1.00	1.00	27.00	1.00	1.00	2.00
0.00	1	1.00	1.00	1.00	18.00	0.00	3.00	0.00
0.00	1	1.00	1.00	1.00	19.00	0.00	1.00	0.00
1.00	0	1.00	1.00	1.00	36.00	1.00	1.00	0.00
0.00	1	1.00	1.00	1.00	17.00	0.00	2.00	0.00
0.00	0	1.00	1.00	1.00	75.00	1.00	3.00	0.00
0.00	1	1.00	2.00	1.00	21.00	0.00	1.00	0.00
0.00	1	1.00	1.00	1.00	68.00	1.00	2.00	0.00
0.00	1	1.00	1.00	1.00	11.00	0.00	2.00	0.00
0.00	1	2.00	1.00	1.00	14.00	0.00	1.00	0.00
1.00	0	1.00	1.00	1.00	44.00	1.00	3.00	0.00
0.00	0	1.00	1.00	1.00	51.00	1.00	1.00	0.00
1.00	1	1.00	2.00	1.00	9.00	0.00	2.00	0.00
0.00	0	1.00	1.00	2.00	47.00	1.00	2.00	0.00

0.00	0	1.00	1.00	2.00	10.00	0.00	2.00	0.00
0.00	0	1.00	1.00	1.00	25.00	1.00	3.00	0.00
0.00	1	1.00	1.00	1.00	10.00	0.00	2.00	0.00
0.00	0	1.00	1.00	1.00	29.00	1.00	2.00	0.00
0.00	0	2.00	2.00	2.00	38.00	1.00	2.00	0.00
0.00	1	2.00	1.00	1.00	20.00	0.00	2.00	0.00
0.00	1	1.00	1.00	1.00	10.00	0.00	3.00	0.00
0.00	0	1.00	1.00	2.00	70.00	1.00	3.00	2.00
0.00	1	2.00	1.00	1.00	19.00	0.00	2.00	0.00
0.00	1	1.00	1.00	1.00	32.00	1.00	2.00	0.00
0.00	0	1.00	1.00	1.00	28.00	1.00	1.00	0.00
0.00	0	1.00	1.00	1.00	26.00	1.00	2.00	0.00
0.00	1	1.00	1.00	1.00	11.00	0.00	2.00	2.00
0.00	1	1.00	1.00	1.00	13.00	0.00	3.00	0.00
0.00	0	1.00	1.00	1.00	36.00	1.00	1.00	0.00
0.00	1	1.00	1.00	1.00	43.00	1.00	1.00	0.00
0.00	0	1.00	1.00	2.00	61.00	1.00	2.00	0.00
0.00	1	1.00	1.00	1.00	9.00	0.00	2.00	0.00
0.00	0	1.00	1.00	1.00	19.00	0.00	1.00	0.00
0.00	0	1.00	1.00	2.00	70.00	1.00	1.00	0.00
0.00	1	1.00	1.00	1.00	15.00	0.00	2.00	0.00
0.00	0	2.00	2.00	1.00	45.00	1.00	3.00	0.00
0.00	0	1.00	1.00	2.00	30.00	1.00	3.00	0.00
0.00	1	1.00	1.00	1.00	22.00	0.00	1.00	0.00
1.00	0	1.00	2.00	2.00	31.00	1.00	2.00	0.00
0.00	0	1.00	1.00	1.00	18.00	0.00	2.00	0.00
0.00	1	1.00	1.00	1.00	12.00	0.00	2.00	0.00
0.00	0	1.00	1.00	1.00	61.00	1.00	1.00	0.00
0.00	0	1.00	1.00	1.00	62.00	1.00	2.00	0.00
0.00	1	1.00	1.00	1.00	20.00	0.00	1.00	0.00
0.00	1	2.00	1.00	1.00	17.00	0.00	2.00	0.00
0.00	1	1.00	1.00	1.00	55.00	1.00	2.00	0.00
0.00	0	1.00	1.00	1.00	49.00	1.00	3.00	0.00
0.00	1	1.00	1.00	1.00	13.00	0.00	2.00	0.00
0.00	0	1.00	1.00	1.00	36.00	1.00	1.00	0.00
0.00	1	1.00	1.00	1.00	41.00	1.00	1.00	0.00
0.00	0	1.00	1.00	2.00	15.00	0.00	3.00	2.00
1.00	0	1.00	2.00	1.00	53.00	1.00	1.00	0.00
0.00	1	2.00	2.00	1.00	20.00	0.00	2.00	2.00
1.00	0	1.00	1.00	2.00	71.00	1.00	3.00	0.00
0.00	0	1.00	1.00	1.00	13.00	0.00	3.00	0.00
0.00	1	1.00	1.00	1.00	14.00	0.00	3.00	0.00
0.00	0	1.00	1.00	1.00	61.00	1.00	2.00	0.00
0.00	0	2.00	1.00	1.00	20.00	0.00	2.00	1.00
0.00	0	2.00	1.00	1.00	70.00	1.00	2.00	0.00
0.00	1	2.00	1.00	1.00	14.00	0.00	3.00	0.00
0.00	0	1.00	1.00	2.00	50.00	1.00	2.00	0.00

0.00	0	1.00	1.00	2.00	59.00	1.00	2.00	1.00
0.00	1	1.00	1.00	1.00	15.00	0.00	3.00	1.00
0.00	1	1.00	1.00	1.00	14.00	0.00	2.00	1.00
0.00	1	1.00	1.00	1.00	18.00	0.00	2.00	1.00
0.00	0	1.00	1.00	2.00	72.00	1.00	2.00	1.00
0.00	0	1.00	1.00	2.00	12.00	0.00	1.00	0.00
0.00	0	1.00	1.00	2.00	68.00	1.00	2.00	1.00
0.00	1	1.00	1.00	1.00	13.00	0.00	2.00	0.00
0.00	0	1.00	1.00	1.00	54.00	1.00	3.00	1.00
0.00	1	2.00	1.00	1.00	13.00	0.00	1.00	1.00
0.00	0	1.00	1.00	2.00	48.00	1.00	3.00	1.00
0.00	0	2.00	1.00	2.00	18.00	0.00	2.00	2.00
0.00	1	1.00	1.00	1.00	13.00	0.00	2.00	0.00
0.00	0	1.00	1.00	1.00	75.00	1.00	3.00	0.00
0.00	1	2.00	1.00	1.00	21.00	0.00	2.00	0.00
0.00	0	1.00	1.00	1.00	38.00	1.00	2.00	0.00
0.00	1	1.00	1.00	1.00	10.00	0.00	2.00	2.00
0.00	0	1.00	1.00	2.00	35.00	1.00	2.00	0.00
0.00	0	1.00	1.00	1.00	40.00	1.00	1.00	0.00
0.00	1	1.00	1.00	1.00	9.00	0.00	3.00	0.00
0.00	1	1.00	1.00	1.00	19.00	0.00	1.00	0.00
0.00	0	1.00	1.00	1.00	48.00	1.00	3.00	0.00
0.00	1	1.00	1.00	1.00	65.00	1.00	3.00	0.00
0.00	1	1.00	1.00	1.00	15.00	0.00	1.00	0.00
0.00	0	1.00	1.00	1.00	41.00	1.00	3.00	0.00
0.00	1	1.00	1.00	1.00	10.00	0.00	2.00	0.00
0.00	0	1.00	1.00	1.00	28.00	1.00	3.00	0.00
0.00	1	1.00	1.00	1.00	10.00	0.00	2.00	0.00
0.00	1	1.00	1.00	1.00	17.00	0.00	3.00	0.00
0.00	1	1.00	1.00	1.00	23.00	0.00	2.00	2.00
0.00	1	1.00	1.00	1.00	31.00	1.00	2.00	2.00
0.00	1	1.00	1.00	1.00	14.00	0.00	2.00	2.00
0.00	1	1.00	1.00	1.00	43.00	1.00	1.00	2.00
0.00	1	1.00	1.00	1.00	11.00	0.00	2.00	1.00
0.00	1	2.00	1.00	1.00	20.00	0.00	2.00	2.00
0.00	0	1.00	1.00	1.00	44.00	1.00	1.00	1.00
0.00	1	1.00	1.00	1.00	40.00	1.00	2.00	2.00
0.00	1	1.00	1.00	1.00	11.00	0.00	2.00	2.00
0.00	0	1.00	1.00	1.00	54.00	1.00	3.00	1.00
0.00	1	1.00	1.00	1.00	18.00	0.00	2.00	0.00
0.00	1	1.00	1.00	1.00	47.00	1.00	2.00	2.00
0.00	1	1.00	1.00	1.00	18.00	0.00	2.00	2.00
0.00	1	1.00	1.00	1.00	16.00	0.00	2.00	1.00
0.00	1	2.00	1.00	1.00	13.00	0.00	2.00	2.00
1.00	1	1.00	1.00	1.00	20.00	0.00	3.00	1.00
0.00	1	1.00	1.00	1.00	19.00	0.00	2.00	2.00
0.00	0	2.00	1.00	1.00	54.00	1.00	2.00	2.00

0.00	1	1.00	1.00	1.00	18.00	0.00	2.00	2.00
1.00	1	1.00	1.00	1.00	32.00	1.00	2.00	2.00
0.00	1	2.00	2.00	1.00	17.00	0.00	1.00	2.00
1.00	1	1.00	1.00	1.00	50.00	1.00	2.00	1.00
0.00	1	2.00	1.00	1.00	12.00	0.00	2.00	1.00
0.00	0	1.00	1.00	1.00	36.00	1.00	2.00	1.00
0.00	1	1.00	1.00	1.00	10.00	0.00	2.00	1.00
0.00	0	1.00	1.00	1.00	46.00	1.00	2.00	2.00
0.00	1	1.00	1.00	1.00	17.00	0.00	3.00	1.00
0.00	1	1.00	1.00	1.00	54.00	1.00	2.00	2.00
0.00	0	1.00	1.00	1.00	61.00	1.00	2.00	1.00
0.00	1	1.00	1.00	1.00	20.00	0.00	2.00	1.00
0.00	1	2.00	1.00	1.00	21.00	0.00	2.00	2.00
0.00	0	1.00	1.00	2.00	46.00	1.00	2.00	1.00

Toilet	Light	Fuel	Wealth	Wealth2	LBW	ANC	Timing	Adequacy
0.00	0.00	0.00	2.00	1.00	0.00	1.00	1.00	1.00
0.00	1.00	0.00	6.00	1.00	0.00	1.00	1.00	1.00
0.00	1.00	0.00	6.00	1.00	0.00	1.00	1.00	1.00
0.00	0.00	0.00	2.00	1.00	0.00	0.00	0.00	0.00
0.00	1.00	0.00	7.00	2.00	0.00	1.00	1.00	1.00
1.00	0.00	0.00	10.00	2.00	0.00	0.00	0.00	0.00
2.00	1.00	1.00	14.00	2.00	0.00	1.00	1.00	1.00
2.00	1.00	1.00	16.00	2.00	0.00	1.00	1.00	1.00
0.00	1.00	0.00	7.00	2.00	0.00	1.00	1.00	1.00
0.00	1.00	0.00	8.00	2.00	0.00	1.00	1.00	1.00
0.00	1.00	0.00	10.00	2.00	1.00	1.00	1.00	1.00
0.00	1.00	0.00	8.00	2.00	0.00	1.00	1.00	1.00
0.00	0.00	0.00	4.00	1.00	0.00	1.00	1.00	1.00
0.00	1.00	0.00	5.00	1.00	0.00	1.00	1.00	1.00
0.00	1.00	0.00	13.00	2.00	1.00	1.00	1.00	1.00
0.00	1.00	0.00	12.00	2.00	0.00	1.00	1.00	1.00
0.00	0.00	0.00	2.00	1.00	0.00	1.00	1.00	1.00
0.00	0.00	0.00	11.00	2.00	0.00	1.00	1.00	1.00
0.00	1.00	0.00	6.00	1.00	0.00	0.00	0.00	0.00
1.00	1.00	1.00	11.00	2.00	0.00	1.00	1.00	1.00
0.00	0.00	0.00	2.00	1.00	0.00	1.00	1.00	1.00
0.00	1.00	0.00	9.00	2.00	0.00	1.00	1.00	1.00
0.00	1.00	0.00	10.00	2.00	0.00	0.00	1.00	0.00
2.00	1.00	1.00	19.00	2.00	0.00	1.00	1.00	1.00
0.00	1.00	0.00	7.00	2.00	0.00	1.00	1.00	1.00
0.00	1.00	0.00	12.00	2.00	0.00	1.00	1.00	1.00
1.00	1.00	0.00	9.00	2.00	0.00	1.00	1.00	1.00
0.00	1.00	0.00	4.00	1.00	0.00	1.00	1.00	1.00
0.00	1.00	0.00	9.00	2.00	0.00	1.00	1.00	1.00
0.00	1.00	0.00	9.00	2.00	0.00	1.00	1.00	1.00
0.00	0.00	0.00	3.00	1.00	0.00	1.00	1.00	1.00
0.00	0.00	0.00	5.00	1.00	0.00	1.00	1.00	1.00
0.00	1.00	0.00	6.00	1.00	0.00	1.00	1.00	1.00
0.00	0.00	0.00	3.00	1.00	1.00	1.00	1.00	1.00
0.00	0.00	0.00	2.00	1.00	0.00	1.00	1.00	1.00
0.00	0.00	0.00	8.00	2.00	0.00	1.00	0.00	0.00
0.00	0.00	0.00	5.00	1.00	0.00	1.00	0.00	0.00
0.00	0.00	0.00	7.00	2.00	0.00	1.00	1.00	1.00
0.00	0.00	0.00	9.00	2.00	0.00	1.00	0.00	0.00
0.00	0.00	0.00	5.00	1.00	0.00	1.00	1.00	1.00
0.00	0.00	0.00	6.00	1.00	0.00	1.00	1.00	1.00
0.00	0.00	0.00	7.00	2.00	1.00	1.00	1.00	1.00
0.00	1.00	0.00	8.00	2.00	1.00	1.00	1.00	1.00
0.00	1.00	0.00	8.00	2.00	0.00	1.00	1.00	1.00
0.00	1.00	0.00	4.00	1.00	0.00	1.00	1.00	1.00
0.00	1.00	0.00	7.00	2.00	0.00	1.00	1.00	1.00

0.00	1.00	0.00	4.00	1.00	0.00	1.00	1.00	1.00
0.00	1.00	0.00	3.00	1.00	0.00	1.00	1.00	1.00
0.00	1.00	0.00	3.00	1.00	0.00	1.00	1.00	1.00
0.00	0.00	0.00	2.00	1.00	0.00	1.00	1.00	1.00
0.00	0.00	0.00	2.00	1.00	0.00	1.00	1.00	1.00
1.00	1.00	0.00	18.00	2.00	0.00	1.00	1.00	1.00
0.00	0.00	0.00	4.00	1.00	0.00	1.00	1.00	1.00
1.00	0.00	0.00	4.00	1.00	0.00	1.00	0.00	0.00
0.00	0.00	0.00	5.00	1.00	0.00	1.00	1.00	1.00
0.00	0.00	0.00	1.00	1.00	0.00	1.00	1.00	1.00
2.00	0.00	0.00	6.00	1.00	0.00	0.00	0.00	0.00
0.00	0.00	0.00	4.00	1.00	0.00	0.00	1.00	0.00
0.00	0.00	0.00	3.00	1.00	0.00	0.00	1.00	0.00
1.00	0.00	1.00	14.00	2.00	0.00	0.00	0.00	0.00
0.00	0.00	0.00	4.00	1.00	1.00	0.00	0.00	0.00
2.00	0.00	0.00	5.00	1.00	0.00	1.00	1.00	1.00
0.00	0.00	0.00	4.00	1.00	0.00	0.00	1.00	0.00
0.00	1.00	0.00	4.00	1.00	0.00	1.00	0.00	0.00
0.00	0.00	0.00	2.00	1.00	0.00	1.00	1.00	1.00
0.00	0.00	0.00	4.00	1.00	1.00	1.00	1.00	1.00
0.00	0.00	0.00	4.00	1.00	0.00	1.00	0.00	0.00
0.00	0.00	0.00	3.00	1.00	0.00	1.00	1.00	1.00
0.00	0.00	0.00	4.00	1.00	0.00	1.00	1.00	1.00
0.00	0.00	0.00	3.00	1.00	0.00	1.00	1.00	1.00
0.00	0.00	0.00	3.00	1.00	0.00	1.00	1.00	1.00
0.00	0.00	0.00	4.00	1.00	0.00	1.00	1.00	1.00
0.00	0.00	0.00	4.00	1.00	0.00	1.00	1.00	1.00
1.00	1.00	0.00	7.00	2.00	0.00	1.00	1.00	1.00
0.00	1.00	0.00	6.00	1.00	0.00	1.00	1.00	1.00
0.00	0.00	1.00	4.00	1.00	0.00	1.00	1.00	1.00
0.00	0.00	0.00	2.00	1.00	0.00	0.00	1.00	0.00
0.00	0.00	0.00	6.00	1.00	0.00	1.00	1.00	1.00
0.00	0.00	0.00	6.00	1.00	0.00	1.00	0.00	0.00
0.00	0.00	0.00	2.00	1.00	0.00	1.00	1.00	1.00
0.00	0.00	0.00	1.00	1.00	0.00	1.00	1.00	1.00
0.00	0.00	0.00	4.00	1.00	0.00	1.00	0.00	0.00
2.00	1.00	0.00	8.00	2.00	0.00	1.00	0.00	0.00
1.00	1.00	1.00	13.00	2.00	0.00	1.00	1.00	1.00
0.00	0.00	0.00	4.00	1.00	1.00	0.00	0.00	0.00
0.00	1.00	0.00	7.00	2.00	0.00	1.00	1.00	1.00
0.00	0.00	0.00	4.00	1.00	0.00	1.00	1.00	1.00
0.00	0.00	0.00	5.00	1.00	1.00	1.00	1.00	1.00
0.00	1.00	0.00	5.00	1.00	0.00	1.00	1.00	1.00
0.00	0.00	0.00	4.00	1.00	0.00	0.00	1.00	0.00
0.00	0.00	0.00	3.00	1.00	0.00	1.00	1.00	1.00
0.00	0.00	0.00	2.00	1.00	0.00	0.00	1.00	0.00
0.00	0.00	0.00	4.00	1.00	0.00	1.00	1.00	1.00

0.00	1.00	0.00	5.00	1.00	0.00	0.00	1.00	0.00
0.00	1.00	0.00	5.00	1.00	0.00	1.00	1.00	1.00
0.00	1.00	0.00	6.00	1.00	0.00	1.00	1.00	1.00
0.00	1.00	0.00	2.00	1.00	0.00	1.00	1.00	1.00
0.00	0.00	0.00	7.00	2.00	0.00	1.00	1.00	1.00
0.00	0.00	0.00	2.00	1.00	0.00	0.00	1.00	0.00
0.00	0.00	0.00	8.00	2.00	0.00	0.00	1.00	0.00
0.00	0.00	0.00	2.00	1.00	0.00	1.00	1.00	1.00
0.00	0.00	0.00	1.00	1.00	0.00	1.00	1.00	1.00
0.00	0.00	0.00	2.00	1.00	0.00	0.00	1.00	0.00
0.00	0.00	0.00	5.00	1.00	0.00	1.00	1.00	1.00
0.00	0.00	0.00	2.00	1.00	0.00	1.00	1.00	1.00
0.00	0.00	0.00	3.00	1.00	0.00	1.00	1.00	1.00
0.00	0.00	0.00	2.00	1.00	0.00	1.00	1.00	1.00
0.00	0.00	0.00	6.00	1.00	0.00	1.00	1.00	1.00
0.00	0.00	0.00	6.00	1.00	0.00	1.00	1.00	1.00
0.00	0.00	0.00	3.00	1.00	0.00	1.00	1.00	1.00
0.00	0.00	0.00	4.00	1.00	0.00	1.00	1.00	1.00
0.00	0.00	0.00	4.00	1.00	0.00	1.00	1.00	1.00
0.00	0.00	0.00	3.00	1.00	0.00	1.00	1.00	1.00
0.00	0.00	0.00	2.00	1.00	1.00	1.00	1.00	1.00
0.00	0.00	0.00	4.00	1.00	0.00	1.00	0.00	0.00
0.00	0.00	0.00	5.00	1.00	0.00	1.00	1.00	1.00
0.00	0.00	0.00	4.00	1.00	0.00	1.00	1.00	1.00
0.00	0.00	0.00	7.00	2.00	0.00	1.00	1.00	1.00
0.00	0.00	0.00	3.00	1.00	0.00	1.00	1.00	1.00
0.00	0.00	0.00	7.00	2.00	1.00	1.00	0.00	0.00
0.00	1.00	0.00	2.00	1.00	0.00	1.00	0.00	0.00
0.00	1.00	0.00	5.00	1.00	0.00	1.00	1.00	1.00
0.00	0.00	0.00	5.00	1.00	0.00	1.00	1.00	1.00
0.00	0.00	0.00	2.00	1.00	0.00	1.00	1.00	1.00
0.00	0.00	0.00	5.00	1.00	0.00	1.00	1.00	1.00
0.00	1.00	0.00	11.00	2.00	0.00	1.00	1.00	1.00
0.00	0.00	0.00	3.00	1.00	0.00	1.00	1.00	1.00
0.00	0.00	0.00	4.00	1.00	0.00	1.00	1.00	1.00
0.00	1.00	0.00	10.00	2.00	0.00	1.00	1.00	1.00
0.00	0.00	0.00	1.00	1.00	0.00	1.00	1.00	1.00
0.00	0.00	0.00	3.00	1.00	1.00	1.00	1.00	1.00
0.00	0.00	0.00	11.00	2.00	0.00	1.00	0.00	0.00
0.00	0.00	0.00	4.00	1.00	0.00	1.00	1.00	1.00
0.00	0.00	0.00	4.00	1.00	0.00	1.00	1.00	1.00
0.00	0.00	0.00	4.00	1.00	0.00	0.00	1.00	0.00
0.00	0.00	0.00	7.00	2.00	0.00	1.00	1.00	1.00
0.00	0.00	0.00	7.00	2.00	0.00	1.00	1.00	1.00
0.00	0.00	0.00	7.00	2.00	0.00	1.00	1.00	1.00
1.00	0.00	0.00	3.00	1.00	0.00	0.00	0.00	0.00
0.00	0.00	0.00	4.00	1.00	0.00	1.00	1.00	1.00

0.00	1.00	0.00	6.00	1.00	0.00	1.00	1.00	1.00
0.00	1.00	0.00	9.00	2.00	1.00	1.00	1.00	1.00
1.00	1.00	0.00	5.00	1.00	0.00	1.00	1.00	1.00
1.00	1.00	0.00	13.00	2.00	0.00	0.00	0.00	0.00
2.00	1.00	0.00	15.00	2.00	0.00	1.00	1.00	1.00
0.00	1.00	1.00	14.00	2.00	0.00	1.00	1.00	1.00
0.00	0.00	0.00	5.00	1.00	0.00	1.00	1.00	1.00
2.00	0.00	0.00	12.00	2.00	0.00	1.00	1.00	1.00
2.00	1.00	0.00	10.00	2.00	0.00	1.00	0.00	0.00
0.00	1.00	0.00	12.00	2.00	0.00	0.00	0.00	0.00
0.00	1.00	0.00	13.00	2.00	0.00	1.00	1.00	1.00
2.00	1.00	0.00	15.00	2.00	0.00	1.00	1.00	1.00
0.00	0.00	0.00	6.00	1.00	0.00	0.00	0.00	0.00
0.00	0.00	0.00	4.00	1.00	0.00	1.00	0.00	0.00
0.00	0.00	0.00	4.00	1.00	0.00	1.00	0.00	0.00
0.00	0.00	0.00	3.00	1.00	0.00	1.00	1.00	1.00
0.00	0.00	0.00	2.00	1.00	0.00	1.00	0.00	0.00
0.00	1.00	0.00	11.00	2.00	0.00	1.00	1.00	1.00
0.00	0.00	0.00	4.00	1.00	0.00	1.00	1.00	1.00
0.00	0.00	0.00	3.00	1.00	0.00	1.00	0.00	0.00
0.00	0.00	1.00	4.00	1.00	0.00	1.00	0.00	0.00
0.00	1.00	0.00	5.00	1.00	0.00	0.00	0.00	0.00
0.00	0.00	0.00	3.00	1.00	0.00	0.00	0.00	0.00
0.00	0.00	0.00	7.00	2.00	0.00	1.00	0.00	0.00
0.00	1.00	0.00	7.00	2.00	0.00	1.00	0.00	0.00
0.00	0.00	0.00	3.00	1.00	0.00	1.00	1.00	1.00
0.00	0.00	0.00	7.00	2.00	0.00	0.00	0.00	0.00
0.00	0.00	0.00	5.00	1.00	0.00	0.00	1.00	0.00
0.00	0.00	0.00	2.00	1.00	0.00	1.00	0.00	0.00
0.00	0.00	0.00	2.00	1.00	0.00	1.00	1.00	1.00
0.00	0.00	0.00	3.00	1.00	0.00	1.00	1.00	1.00
0.00	0.00	0.00	5.00	1.00	0.00	1.00	1.00	1.00
0.00	0.00	0.00	2.00	1.00	0.00	1.00	1.00	1.00
0.00	0.00	0.00	2.00	1.00	0.00	1.00	1.00	1.00
0.00	0.00	0.00	1.00	1.00	0.00	1.00	1.00	1.00
0.00	0.00	0.00	4.00	1.00	0.00	1.00	1.00	1.00
0.00	0.00	0.00	3.00	1.00	0.00	1.00	1.00	1.00
0.00	0.00	0.00	5.00	1.00	0.00	1.00	1.00	1.00
0.00	0.00	0.00	5.00	1.00	0.00	1.00	0.00	0.00
0.00	1.00	0.00	8.00	2.00	0.00	1.00	1.00	1.00
0.00	0.00	0.00	3.00	1.00	0.00	1.00	1.00	1.00
0.00	0.00	0.00	5.00	1.00	0.00	1.00	1.00	1.00
0.00	0.00	0.00	1.00	1.00	0.00	1.00	1.00	1.00
0.00	0.00	0.00	1.00	1.00	0.00	0.00	0.00	0.00
0.00	0.00	0.00	6.00	1.00	0.00	1.00	1.00	1.00
0.00	0.00	0.00	3.00	1.00	0.00	1.00	1.00	1.00
0.00	0.00	0.00	3.00	1.00	0.00	1.00	1.00	1.00

0.00	1.00	0.00	8.00	2.00	0.00	1.00	0.00	0.00
0.00	1.00	0.00	6.00	1.00	0.00	1.00	1.00	1.00
0.00	0.00	0.00	2.00	1.00	0.00	0.00	0.00	0.00
0.00	1.00	0.00	13.00	2.00	0.00	1.00	0.00	0.00
0.00	1.00	0.00	7.00	2.00	1.00	1.00	1.00	1.00
0.00	0.00	0.00	1.00	1.00	0.00	1.00	1.00	1.00
0.00	0.00	0.00	4.00	1.00	0.00	1.00	1.00	1.00
0.00	1.00	0.00	8.00	2.00	0.00	1.00	1.00	1.00
0.00	0.00	0.00	7.00	2.00	0.00	1.00	1.00	1.00
0.00	0.00	0.00	4.00	1.00	0.00	1.00	0.00	0.00
0.00	0.00	0.00	3.00	1.00	1.00	1.00	1.00	1.00
0.00	0.00	0.00	6.00	1.00	1.00	1.00	1.00	1.00
0.00	0.00	0.00	6.00	1.00	0.00	1.00	0.00	0.00
0.00	0.00	0.00	3.00	1.00	0.00	1.00	1.00	1.00
0.00	0.00	0.00	4.00	1.00	0.00	1.00	1.00	1.00
0.00	0.00	0.00	2.00	1.00	0.00	1.00	1.00	1.00
0.00	0.00	0.00	5.00	1.00	0.00	1.00	1.00	1.00
0.00	1.00	0.00	6.00	1.00	0.00	1.00	1.00	1.00
0.00	0.00	0.00	5.00	1.00	0.00	1.00	1.00	1.00
0.00	0.00	0.00	2.00	1.00	0.00	1.00	1.00	1.00
0.00	1.00	0.00	3.00	1.00	0.00	1.00	1.00	1.00
0.00	0.00	0.00	5.00	1.00	0.00	1.00	1.00	1.00
0.00	0.00	0.00	4.00	1.00	0.00	1.00	1.00	1.00
0.00	0.00	0.00	3.00	1.00	0.00	1.00	1.00	1.00
0.00	1.00	0.00	11.00	2.00	0.00	1.00	1.00	1.00
0.00	1.00	1.00	14.00	2.00	0.00	1.00	0.00	0.00
1.00	1.00	0.00	11.00	2.00	1.00	0.00	0.00	0.00
0.00	1.00	0.00	10.00	2.00	0.00	1.00	1.00	1.00
1.00	1.00	1.00	15.00	2.00	0.00	1.00	1.00	1.00
1.00	1.00	0.00	15.00	2.00	0.00	1.00	1.00	1.00
0.00	1.00	0.00	7.00	2.00	0.00	1.00	1.00	1.00
0.00	1.00	1.00	13.00	2.00	0.00	1.00	1.00	1.00
0.00	0.00	0.00	2.00	1.00	0.00	1.00	0.00	0.00
2.00	1.00	0.00	16.00	2.00	0.00	1.00	1.00	1.00
0.00	0.00	0.00	4.00	1.00	0.00	1.00	1.00	1.00
0.00	0.00	0.00	2.00	1.00	1.00	1.00	1.00	1.00
0.00	1.00	0.00	7.00	2.00	0.00	1.00	1.00	1.00
0.00	0.00	0.00	1.00	1.00	0.00	1.00	1.00	1.00
0.00	0.00	0.00	1.00	1.00	0.00	1.00	1.00	1.00
0.00	0.00	0.00	2.00	1.00	1.00	1.00	1.00	1.00
0.00	0.00	0.00	4.00	1.00	0.00	1.00	1.00	1.00
0.00	0.00	0.00	3.00	1.00	0.00	1.00	1.00	1.00
1.00	1.00	0.00	7.00	2.00	0.00	1.00	1.00	1.00
0.00	0.00	0.00	4.00	1.00	0.00	1.00	1.00	1.00
0.00	0.00	0.00	2.00	1.00	0.00	1.00	1.00	1.00
0.00	0.00	1.00	2.00	1.00	0.00	1.00	1.00	1.00
0.00	0.00	0.00	1.00	1.00	0.00	1.00	1.00	1.00

1.00	0.00	0.00	6.00	1.00	0.00	1.00	1.00	1.00
0.00	0.00	0.00	5.00	1.00	0.00	1.00	1.00	1.00
0.00	0.00	0.00	2.00	1.00	0.00	1.00	1.00	1.00
0.00	0.00	0.00	7.00	2.00	1.00	1.00	1.00	1.00
0.00	0.00	0.00	1.00	1.00	0.00	1.00	1.00	1.00
0.00	0.00	0.00	4.00	1.00	0.00	1.00	1.00	1.00
0.00	0.00	0.00	2.00	1.00	0.00	1.00	1.00	1.00
0.00	0.00	0.00	1.00	1.00	1.00	1.00	1.00	1.00
0.00	1.00	0.00	11.00	2.00	0.00	1.00	1.00	1.00
0.00	1.00	1.00	13.00	2.00	0.00	1.00	1.00	1.00
1.00	1.00	0.00	12.00	2.00	0.00	1.00	1.00	1.00
0.00	0.00	0.00	8.00	2.00	0.00	1.00	1.00	1.00
2.00	1.00	1.00	17.00	2.00	0.00	1.00	0.00	0.00
0.00	1.00	0.00	6.00	1.00	0.00	1.00	1.00	1.00
0.00	0.00	0.00	5.00	1.00	0.00	1.00	1.00	1.00
1.00	1.00	0.00	13.00	2.00	0.00	1.00	1.00	1.00
0.00	0.00	0.00	1.00	1.00	0.00	1.00	1.00	1.00
0.00	0.00	0.00	7.00	2.00	1.00	1.00	1.00	1.00
0.00	0.00	0.00	7.00	2.00	0.00	1.00	1.00	1.00
0.00	1.00	0.00	8.00	2.00	0.00	1.00	1.00	1.00
0.00	0.00	0.00	4.00	1.00	0.00	1.00	1.00	1.00
0.00	0.00	0.00	1.00	1.00	0.00	1.00	0.00	0.00
0.00	0.00	0.00	4.00	1.00	1.00	1.00	0.00	0.00
0.00	0.00	0.00	1.00	1.00	0.00	1.00	1.00	1.00
1.00	0.00	0.00	3.00	1.00	1.00	1.00	1.00	1.00
0.00	0.00	0.00	3.00	1.00	0.00	1.00	0.00	0.00
0.00	0.00	0.00	3.00	1.00	0.00	0.00	1.00	0.00
0.00	0.00	0.00	5.00	1.00	1.00	1.00	0.00	0.00
0.00	0.00	0.00	1.00	1.00	0.00	1.00	1.00	1.00
0.00	0.00	0.00	2.00	1.00	0.00	0.00	0.00	0.00
0.00	0.00	0.00	2.00	1.00	0.00	1.00	1.00	1.00
0.00	0.00	0.00	5.00	1.00	1.00	1.00	1.00	1.00
0.00	0.00	0.00	2.00	1.00	0.00	1.00	1.00	1.00
0.00	0.00	0.00	3.00	1.00	0.00	1.00	1.00	1.00
0.00	0.00	0.00	6.00	1.00	0.00	1.00	1.00	1.00
0.00	0.00	0.00	8.00	2.00	0.00	1.00	1.00	1.00
0.00	0.00	0.00	3.00	1.00	0.00	1.00	1.00	1.00
0.00	1.00	0.00	9.00	2.00	0.00	1.00	1.00	1.00
0.00	1.00	0.00	13.00	2.00	0.00	1.00	1.00	1.00
0.00	0.00	0.00	6.00	1.00	0.00	1.00	1.00	1.00
1.00	1.00	0.00	10.00	2.00	0.00	1.00	1.00	1.00
1.00	1.00	1.00	15.00	2.00	0.00	1.00	1.00	1.00
0.00	0.00	0.00	2.00	1.00	0.00	0.00	0.00	0.00
1.00	1.00	0.00	12.00	2.00	0.00	1.00	1.00	1.00
1.00	0.00	0.00	4.00	1.00	0.00	1.00	1.00	1.00
0.00	0.00	0.00	5.00	1.00	0.00	1.00	1.00	1.00
0.00	0.00	0.00	3.00	1.00	0.00	1.00	0.00	0.00

2.00	1.00	0.00	16.00	2.00	0.00	1.00	1.00	1.00
0.00	0.00	0.00	1.00	1.00	0.00	1.00	1.00	1.00
0.00	1.00	0.00	12.00	2.00	0.00	1.00	1.00	1.00
2.00	1.00	0.00	17.00	2.00	0.00	1.00	1.00	1.00
0.00	0.00	0.00	6.00	1.00	0.00	1.00	1.00	1.00
1.00	0.00	0.00	4.00	1.00	0.00	1.00	1.00	1.00
0.00	1.00	0.00	8.00	2.00	1.00	1.00	0.00	0.00
0.00	1.00	0.00	5.00	1.00	0.00	1.00	1.00	1.00
0.00	1.00	0.00	14.00	2.00	0.00	1.00	0.00	0.00
0.00	0.00	0.00	5.00	1.00	0.00	1.00	1.00	1.00
0.00	1.00	0.00	13.00	2.00	0.00	1.00	1.00	1.00
1.00	0.00	0.00	5.00	1.00	0.00	1.00	1.00	1.00
0.00	0.00	0.00	5.00	1.00	0.00	1.00	1.00	1.00
2.00	0.00	0.00	9.00	2.00	0.00	1.00	1.00	1.00
0.00	1.00	0.00	5.00	1.00	0.00	1.00	0.00	0.00
2.00	1.00	0.00	12.00	2.00	0.00	1.00	1.00	1.00
1.00	1.00	1.00	14.00	2.00	0.00	1.00	0.00	0.00
0.00	0.00	0.00	6.00	1.00	0.00	1.00	1.00	1.00
1.00	1.00	0.00	11.00	2.00	0.00	1.00	1.00	1.00
0.00	0.00	0.00	5.00	1.00	0.00	1.00	1.00	1.00
0.00	0.00	0.00	8.00	2.00	0.00	1.00	1.00	1.00
1.00	1.00	0.00	14.00	2.00	0.00	1.00	1.00	1.00
1.00	1.00	0.00	14.00	2.00	0.00	1.00	1.00	1.00
0.00	0.00	0.00	10.00	2.00	0.00	1.00	1.00	1.00
0.00	0.00	0.00	3.00	1.00	0.00	1.00	1.00	1.00
0.00	0.00	0.00	5.00	1.00	0.00	1.00	0.00	0.00
0.00	0.00	0.00	2.00	1.00	0.00	1.00	1.00	1.00
0.00	0.00	0.00	3.00	1.00	0.00	1.00	1.00	1.00
2.00	1.00	1.00	16.00	2.00	0.00	1.00	1.00	1.00
0.00	1.00	0.00	8.00	2.00	0.00	0.00	0.00	0.00
2.00	1.00	0.00	14.00	2.00	0.00	1.00	1.00	1.00
0.00	1.00	0.00	12.00	2.00	0.00	1.00	1.00	1.00
2.00	1.00	1.00	16.00	2.00	0.00	1.00	1.00	1.00
0.00	1.00	0.00	11.00	2.00	0.00	1.00	1.00	1.00
0.00	0.00	0.00	5.00	1.00	1.00	1.00	1.00	1.00
0.00	0.00	0.00	3.00	1.00	0.00	1.00	1.00	1.00
0.00	0.00	0.00	8.00	2.00	0.00	1.00	1.00	1.00
0.00	0.00	0.00	3.00	1.00	0.00	1.00	1.00	1.00
0.00	0.00	0.00	3.00	1.00	0.00	1.00	1.00	1.00
0.00	0.00	0.00	6.00	1.00	0.00	1.00	1.00	1.00
0.00	0.00	0.00	3.00	1.00	0.00	1.00	1.00	1.00
0.00	1.00	0.00	10.00	2.00	0.00	1.00	1.00	1.00
0.00	1.00	0.00	10.00	2.00	0.00	1.00	1.00	1.00
0.00	0.00	0.00	4.00	1.00	0.00	1.00	1.00	1.00
1.00	0.00	0.00	8.00	2.00	0.00	1.00	1.00	1.00
0.00	1.00	0.00	6.00	1.00	0.00	1.00	1.00	1.00
0.00	0.00	0.00	4.00	1.00	0.00	1.00	1.00	1.00

0.00	0.00	0.00	1.00	1.00	0.00	1.00	1.00	1.00
0.00	0.00	0.00	3.00	1.00	0.00	1.00	1.00	1.00
0.00	1.00	0.00	13.00	2.00	0.00	1.00	1.00	1.00
0.00	0.00	0.00	5.00	1.00	0.00	1.00	1.00	1.00
0.00	1.00	0.00	5.00	1.00	0.00	1.00	1.00	1.00
0.00	1.00	0.00	3.00	1.00	0.00	1.00	1.00	1.00
1.00	1.00	1.00	16.00	2.00	0.00	1.00	1.00	1.00
0.00	1.00	0.00	13.00	2.00	0.00	1.00	1.00	1.00
0.00	0.00	0.00	5.00	1.00	0.00	1.00	1.00	1.00
0.00	1.00	0.00	6.00	1.00	0.00	1.00	1.00	1.00
0.00	1.00	0.00	8.00	2.00	0.00	1.00	1.00	1.00
0.00	1.00	0.00	10.00	2.00	0.00	1.00	1.00	1.00
0.00	0.00	0.00	1.00	1.00	0.00	1.00	1.00	1.00
0.00	1.00	0.00	7.00	2.00	0.00	1.00	1.00	1.00
0.00	0.00	0.00	4.00	1.00	0.00	1.00	1.00	1.00
0.00	0.00	0.00	7.00	2.00	0.00	1.00	1.00	1.00
0.00	1.00	0.00	10.00	2.00	0.00	1.00	1.00	1.00
0.00	1.00	0.00	11.00	2.00	0.00	1.00	1.00	1.00
0.00	0.00	0.00	4.00	1.00	0.00	1.00	1.00	1.00
0.00	0.00	0.00	4.00	1.00	0.00	1.00	1.00	1.00
0.00	0.00	0.00	4.00	1.00	0.00	1.00	1.00	1.00
0.00	1.00	0.00	12.00	2.00	0.00	1.00	1.00	1.00
0.00	1.00	0.00	12.00	2.00	0.00	1.00	1.00	1.00
0.00	1.00	0.00	13.00	2.00	0.00	1.00	1.00	1.00
0.00	1.00	0.00	10.00	2.00	0.00	1.00	1.00	1.00
2.00	1.00	1.00	17.00	2.00	0.00	1.00	1.00	1.00
0.00	0.00	0.00	7.00	2.00	0.00	1.00	1.00	1.00
1.00	1.00	0.00	15.00	2.00	0.00	1.00	1.00	1.00
0.00	1.00	0.00	12.00	2.00	0.00	1.00	1.00	1.00
2.00	1.00	1.00	17.00	2.00	1.00	1.00	1.00	1.00
2.00	1.00	0.00	14.00	2.00	0.00	1.00	1.00	1.00
2.00	1.00	1.00	15.00	2.00	0.00	1.00	0.00	0.00
0.00	0.00	0.00	7.00	2.00	0.00	0.00	1.00	0.00
0.00	0.00	0.00	5.00	1.00	0.00	1.00	0.00	0.00
1.00	1.00	1.00	14.00	2.00	0.00	1.00	1.00	1.00
2.00	1.00	1.00	15.00	2.00	0.00	1.00	1.00	1.00
2.00	1.00	1.00	16.00	2.00	0.00	1.00	1.00	1.00
1.00	1.00	1.00	15.00	2.00	0.00	1.00	1.00	1.00
2.00	1.00	0.00	17.00	2.00	1.00	1.00	1.00	1.00
2.00	1.00	1.00	17.00	2.00	0.00	1.00	1.00	1.00
1.00	1.00	0.00	6.00	1.00	0.00	1.00	1.00	1.00
0.00	1.00	1.00	13.00	2.00	0.00	1.00	0.00	0.00
0.00	1.00	1.00	14.00	2.00	0.00	1.00	1.00	1.00
0.00	1.00	0.00	7.00	2.00	0.00	1.00	1.00	1.00
0.00	1.00	0.00	2.00	1.00	0.00	1.00	0.00	0.00
1.00	1.00	1.00	14.00	2.00	0.00	1.00	1.00	1.00
2.00	1.00	1.00	16.00	2.00	0.00	1.00	1.00	1.00

1.00	1.00	1.00	14.00	2.00	0.00	1.00	1.00	1.00
2.00	1.00	0.00	10.00	2.00	0.00	1.00	1.00	1.00
1.00	1.00	1.00	16.00	2.00	0.00	1.00	1.00	1.00
0.00	0.00	0.00	3.00	1.00	0.00	1.00	1.00	1.00
1.00	1.00	1.00	14.00	2.00	0.00	1.00	1.00	1.00
2.00	1.00	1.00	18.00	2.00	0.00	1.00	1.00	1.00
0.00	0.00	0.00	2.00	1.00	0.00	1.00	1.00	1.00
1.00	1.00	0.00	8.00	2.00	0.00	1.00	1.00	1.00
1.00	1.00	0.00	5.00	1.00	1.00	1.00	1.00	1.00
0.00	0.00	0.00	2.00	1.00	0.00	1.00	1.00	1.00
1.00	1.00	0.00	8.00	2.00	0.00	1.00	1.00	1.00
0.00	1.00	0.00	13.00	2.00	0.00	1.00	1.00	1.00
1.00	0.00	0.00	7.00	2.00	0.00	1.00	1.00	1.00
0.00	0.00	0.00	1.00	1.00	1.00	1.00	1.00	1.00
0.00	1.00	0.00	6.00	1.00	0.00	1.00	1.00	1.00
0.00	1.00	0.00	6.00	1.00	0.00	1.00	1.00	1.00
0.00	0.00	0.00	2.00	1.00	0.00	1.00	1.00	1.00
0.00	0.00	0.00	7.00	2.00	0.00	1.00	1.00	1.00
0.00	0.00	0.00	12.00	2.00	0.00	1.00	1.00	1.00
0.00	0.00	0.00	3.00	1.00	0.00	1.00	1.00	1.00
0.00	0.00	0.00	5.00	1.00	0.00	1.00	1.00	1.00
0.00	0.00	0.00	7.00	2.00	1.00	1.00	1.00	1.00
0.00	0.00	0.00	2.00	1.00	0.00	1.00	1.00	1.00
0.00	1.00	0.00	7.00	2.00	1.00	1.00	1.00	1.00
0.00	1.00	0.00	14.00	2.00	0.00	1.00	1.00	1.00
0.00	1.00	0.00	12.00	2.00	0.00	1.00	1.00	1.00
0.00	0.00	0.00	6.00	1.00	0.00	1.00	1.00	1.00
0.00	1.00	0.00	7.00	2.00	0.00	1.00	1.00	1.00
0.00	0.00	0.00	4.00	1.00	0.00	1.00	1.00	1.00
0.00	1.00	1.00	16.00	2.00	0.00	1.00	1.00	1.00
0.00	1.00	0.00	11.00	2.00	0.00	1.00	1.00	1.00
0.00	0.00	0.00	3.00	1.00	0.00	1.00	1.00	1.00
0.00	0.00	0.00	2.00	1.00	0.00	1.00	1.00	1.00
0.00	0.00	0.00	6.00	1.00	0.00	1.00	1.00	1.00
0.00	0.00	0.00	4.00	1.00	0.00	1.00	1.00	1.00
0.00	0.00	0.00	1.00	1.00	0.00	1.00	1.00	1.00
0.00	0.00	0.00	1.00	1.00	0.00	1.00	1.00	1.00
0.00	0.00	0.00	1.00	1.00	0.00	1.00	1.00	1.00
0.00	0.00	0.00	1.00	1.00	0.00	1.00	1.00	1.00
0.00	0.00	0.00	3.00	1.00	0.00	1.00	1.00	1.00
0.00	0.00	0.00	2.00	1.00	0.00	1.00	1.00	1.00
0.00	0.00	0.00	0.00	1.00	0.00	1.00	1.00	1.00
0.00	0.00	0.00	2.00	1.00	0.00	1.00	1.00	1.00
0.00	0.00	0.00	4.00	1.00	1.00	1.00	1.00	1.00
0.00	0.00	0.00	1.00	1.00	0.00	1.00	1.00	1.00
0.00	0.00	0.00	3.00	1.00	1.00	1.00	1.00	1.00
0.00	0.00	0.00	5.00	1.00	0.00	1.00	1.00	1.00

0.00	0.00	0.00	3.00	1.00	0.00	1.00	1.00	1.00
0.00	0.00	0.00	1.00	1.00	0.00	1.00	1.00	1.00
0.00	0.00	0.00	2.00	1.00	0.00	1.00	1.00	1.00
0.00	0.00	0.00	3.00	1.00	0.00	1.00	1.00	1.00
1.00	1.00	0.00	10.00	2.00	0.00	1.00	1.00	1.00
0.00	0.00	0.00	1.00	1.00	0.00	1.00	1.00	1.00
0.00	0.00	0.00	4.00	1.00	0.00	1.00	1.00	1.00
0.00	0.00	0.00	9.00	2.00	0.00	1.00	1.00	1.00
0.00	0.00	0.00	5.00	1.00	0.00	1.00	1.00	1.00
0.00	0.00	0.00	3.00	1.00	0.00	1.00	1.00	1.00
0.00	0.00	0.00	1.00	1.00	0.00	1.00	1.00	1.00
0.00	0.00	0.00	3.00	1.00	0.00	1.00	1.00	1.00
0.00	1.00	0.00	11.00	2.00	0.00	1.00	1.00	1.00
0.00	0.00	0.00	5.00	1.00	0.00	1.00	1.00	1.00
0.00	1.00	0.00	6.00	1.00	0.00	1.00	1.00	1.00
0.00	0.00	0.00	2.00	1.00	0.00	1.00	1.00	1.00
0.00	0.00	0.00	7.00	2.00	0.00	1.00	1.00	1.00
0.00	0.00	0.00	4.00	1.00	0.00	1.00	1.00	1.00
0.00	0.00	0.00	5.00	1.00	0.00	1.00	1.00	1.00
0.00	0.00	0.00	1.00	1.00	0.00	1.00	1.00	1.00
0.00	0.00	0.00	2.00	1.00	0.00	1.00	1.00	1.00
0.00	0.00	0.00	2.00	1.00	0.00	1.00	1.00	1.00
0.00	0.00	0.00	2.00	1.00	0.00	1.00	1.00	1.00
0.00	0.00	0.00	2.00	1.00	0.00	1.00	1.00	1.00
0.00	0.00	0.00	3.00	1.00	0.00	1.00	1.00	1.00
0.00	0.00	0.00	7.00	2.00	1.00	1.00	1.00	1.00
0.00	0.00	0.00	3.00	1.00	0.00	1.00	1.00	1.00
0.00	0.00	0.00	6.00	1.00	0.00	1.00	1.00	1.00
0.00	0.00	0.00	2.00	1.00	0.00	1.00	1.00	1.00
0.00	0.00	0.00	4.00	1.00	0.00	1.00	1.00	1.00
0.00	0.00	0.00	1.00	1.00	0.00	1.00	1.00	1.00
0.00	0.00	0.00	3.00	1.00	1.00	1.00	1.00	1.00
1.00	0.00	0.00	6.00	1.00	0.00	1.00	1.00	1.00
0.00	0.00	0.00	1.00	1.00	0.00	1.00	1.00	1.00
0.00	0.00	0.00	4.00	1.00	0.00	1.00	1.00	1.00
0.00	1.00	0.00	5.00	1.00	0.00	1.00	1.00	1.00
0.00	1.00	0.00	5.00	1.00	0.00	1.00	1.00	1.00
0.00	1.00	0.00	5.00	1.00	0.00	1.00	1.00	1.00
0.00	0.00	0.00	6.00	1.00	0.00	1.00	1.00	1.00
0.00	0.00	0.00	2.00	1.00	0.00	1.00	1.00	1.00
0.00	0.00	0.00	1.00	1.00	0.00	1.00	1.00	1.00
0.00	0.00	0.00	5.00	1.00	0.00	1.00	1.00	1.00
0.00	0.00	0.00	1.00	1.00	0.00	1.00	1.00	1.00
0.00	0.00	0.00	5.00	1.00	0.00	1.00	1.00	1.00
0.00	0.00	0.00	5.00	1.00	0.00	1.00	1.00	1.00
0.00	0.00	0.00	3.00	1.00	0.00	1.00	1.00	1.00
0.00	0.00	0.00	2.00	1.00	0.00	1.00	1.00	1.00

0.00	0.00	0.00	4.00	1.00	0.00	1.00	1.00	1.00
0.00	0.00	0.00	4.00	1.00	0.00	1.00	1.00	1.00
0.00	0.00	0.00	3.00	1.00	0.00	1.00	1.00	1.00
0.00	0.00	0.00	4.00	1.00	0.00	1.00	1.00	1.00
0.00	0.00	0.00	5.00	1.00	0.00	1.00	1.00	1.00
0.00	0.00	0.00	4.00	1.00	0.00	1.00	1.00	1.00
0.00	0.00	0.00	2.00	1.00	0.00	1.00	1.00	1.00
0.00	0.00	0.00	2.00	1.00	0.00	0.00	1.00	0.00
0.00	0.00	0.00	5.00	1.00	0.00	1.00	1.00	1.00
0.00	0.00	0.00	5.00	1.00	0.00	1.00	1.00	1.00
0.00	0.00	0.00	8.00	2.00	0.00	1.00	1.00	1.00
0.00	0.00	0.00	12.00	2.00	0.00	1.00	1.00	1.00
0.00	0.00	0.00	6.00	1.00	0.00	0.00	1.00	0.00
0.00	0.00	0.00	7.00	2.00	0.00	1.00	1.00	1.00
0.00	0.00	0.00	8.00	2.00	0.00	1.00	1.00	1.00
0.00	0.00	0.00	3.00	1.00	0.00	1.00	1.00	1.00
0.00	0.00	0.00	9.00	2.00	0.00	1.00	1.00	1.00
0.00	0.00	0.00	2.00	1.00	0.00	1.00	1.00	1.00
0.00	0.00	0.00	3.00	1.00	0.00	1.00	1.00	1.00
0.00	0.00	0.00	5.00	1.00	0.00	1.00	1.00	1.00
0.00	0.00	0.00	3.00	1.00	0.00	1.00	1.00	1.00
0.00	0.00	0.00	1.00	1.00	0.00	1.00	1.00	1.00
0.00	0.00	0.00	4.00	1.00	0.00	1.00	1.00	1.00
0.00	0.00	0.00	1.00	1.00	0.00	1.00	1.00	1.00
0.00	0.00	0.00	2.00	1.00	0.00	1.00	1.00	1.00
0.00	1.00	0.00	10.00	2.00	1.00	1.00	1.00	1.00
0.00	0.00	0.00	3.00	1.00	0.00	0.00	0.00	0.00
0.00	0.00	0.00	5.00	1.00	0.00	0.00	0.00	0.00
0.00	1.00	0.00	6.00	1.00	0.00	1.00	0.00	0.00
0.00	1.00	1.00	15.00	2.00	0.00	1.00	1.00	1.00
2.00	1.00	1.00	17.00	2.00	0.00	1.00	1.00	1.00
0.00	1.00	0.00	9.00	2.00	0.00	1.00	1.00	1.00
1.00	1.00	0.00	10.00	2.00	0.00	1.00	1.00	1.00
1.00	1.00	1.00	13.00	2.00	0.00	1.00	0.00	0.00
2.00	0.00	1.00	12.00	2.00	0.00	1.00	1.00	1.00
1.00	0.00	0.00	5.00	1.00	0.00	1.00	1.00	1.00
0.00	1.00	1.00	18.00	2.00	1.00	1.00	0.00	0.00
1.00	0.00	1.00	16.00	2.00	1.00	1.00	1.00	1.00
0.00	1.00	0.00	4.00	1.00	0.00	1.00	1.00	1.00
0.00	1.00	1.00	14.00	2.00	0.00	1.00	1.00	1.00
0.00	1.00	1.00	14.00	2.00	0.00	1.00	1.00	1.00
2.00	1.00	1.00	18.00	2.00	0.00	1.00	0.00	0.00
0.00	1.00	0.00	7.00	2.00	0.00	1.00	0.00	0.00
2.00	1.00	0.00	17.00	2.00	0.00	1.00	1.00	1.00
0.00	0.00	0.00	3.00	1.00	0.00	1.00	1.00	1.00
0.00	0.00	0.00	7.00	2.00	0.00	1.00	1.00	1.00
2.00	1.00	0.00	12.00	2.00	0.00	1.00	1.00	1.00

2.00	1.00	1.00	20.00	2.00	0.00	1.00	1.00	1.00
0.00	0.00	1.00	15.00	2.00	1.00	1.00	1.00	1.00
0.00	0.00	1.00	13.00	2.00	0.00	1.00	1.00	1.00
1.00	0.00	1.00	14.00	2.00	0.00	1.00	0.00	0.00
0.00	1.00	1.00	13.00	2.00	0.00	1.00	1.00	1.00
0.00	1.00	1.00	13.00	2.00	0.00	1.00	1.00	1.00
0.00	1.00	0.00	8.00	2.00	1.00	1.00	1.00	1.00
1.00	1.00	0.00	9.00	2.00	0.00	1.00	0.00	0.00
1.00	1.00	1.00	16.00	2.00	0.00	1.00	0.00	0.00
1.00	1.00	1.00	15.00	2.00	0.00	1.00	0.00	0.00
0.00	0.00	0.00	9.00	2.00	0.00	1.00	1.00	1.00
0.00	1.00	1.00	15.00	2.00	0.00	1.00	1.00	1.00
1.00	1.00	1.00	16.00	2.00	0.00	0.00	1.00	0.00
0.00	0.00	0.00	7.00	2.00	0.00	1.00	1.00	1.00

Education1	Occupatior	Postnatal	Postnatal2	Agechild2	DDS	Minimum	Parity	Interaction
1.00	2.00	9.40	0.45	3.00	3.00	0.00	3.00	3.00
1.00	1.00	6.20	0.56	2.00	3.00	0.00	2.00	2.00
2.00	1.00	6.80	0.52	3.00	3.00	0.00	2.00	0.00
1.00	1.00	4.60	0.58	2.00	1.00	0.00	3.00	0.00
1.00	3.00	2.00	1.00	1.00	0.00	0.00	3.00	1.00
1.00	2.00	5.90	0.98	2.00	4.00	1.00	2.00	2.00
2.00	3.00	5.40	0.45	3.00	2.00	0.00	2.00	0.00
2.00	2.00	4.90	0.38	3.00	1.00	0.00	1.00	0.00
2.00	1.00	3.90	0.98	1.00	0.00	0.00	2.00	1.00
2.00	2.00	3.80	0.54	2.00	4.00	1.00	2.00	0.00
0.00	1.00	4.70	1.18	1.00	0.00	0.00	3.00	0.00
1.00	2.00	4.70	1.18	1.00	0.00	0.00	2.00	1.00
0.00	1.00	4.80	0.96	1.00	0.00	0.00	2.00	0.00
1.00	1.00	4.40	0.55	2.00	1.00	0.00	2.00	0.00
1.00	1.00	2.00	0.67	1.00	0.00	0.00	2.00	1.00
1.00	1.00	3.70	0.74	1.00	0.00	0.00	2.00	0.00
1.00	1.00	5.60	0.93	2.00	4.00	1.00	2.00	2.00
1.00	1.00	2.60	0.24	2.00	3.00	0.00	3.00	0.00
2.00	3.00	4.00	1.33	1.00	0.00	0.00	3.00	0.00
1.00	2.00	3.70	1.23	1.00	0.00	0.00	3.00	1.00
1.00	1.00	4.60	0.92	1.00	0.00	0.00	2.00	1.00
1.00	1.00	4.70	0.59	2.00	5.00	1.00	2.00	0.00
1.00	1.00	6.90	0.86	2.00	5.00	1.00	3.00	2.00
2.00	1.00	6.00	0.50	3.00	5.00	1.00	2.00	0.00
1.00	3.00	6.80	0.36	3.00	2.00	0.00	3.00	3.00
0.00	2.00	4.30	0.61	2.00	4.00	1.00	3.00	2.00
1.00	2.00	4.00	1.00	1.00	0.00	0.00	3.00	1.00
1.00	1.00	5.80	0.64	2.00	4.00	1.00	2.00	0.00
0.00	2.00	7.00	0.54	3.00	4.00	1.00	3.00	3.00
1.00	2.00	6.60	0.83	2.00	6.00	1.00	3.00	0.00
0.00	2.00	4.80	0.60	2.00	4.00	1.00	2.00	0.00
0.00	1.00	5.50	0.79	2.00	3.00	0.00	2.00	0.00
0.00	1.00	6.70	0.61	2.00	3.00	0.00	2.00	2.00
0.00	2.00	5.60	0.51	2.00	4.00	1.00	3.00	2.00
0.00	2.00	6.40	0.58	2.00	4.00	1.00	3.00	0.00
2.00	1.00	6.40	1.07	2.00	4.00	1.00	2.00	0.00
2.00	1.00	10.90	0.55	3.00	3.00	0.00	2.00	3.00
1.00	1.00	8.40	0.47	3.00	3.00	0.00	2.00	0.00
1.00	3.00	4.50	0.41	2.00	5.00	1.00	2.00	0.00
0.00	1.00	5.40	0.54	2.00	4.00	1.00	3.00	0.00
1.00	3.00	9.10	0.38	3.00	4.00	1.00	3.00	3.00
1.00	1.00	7.20	0.60	3.00	4.00	1.00	3.00	0.00
1.00	1.00	7.60	0.63	3.00	4.00	1.00	3.00	3.00
1.00	2.00	6.70	0.29	3.00	6.00	1.00	3.00	3.00
0.00	2.00	9.20	0.38	3.00	3.00	0.00	3.00	3.00
0.00	2.00	8.90	0.68	3.00	5.00	1.00	3.00	3.00

1.00	1.00	10.30	0.43	3.00	3.00	0.00	3.00	0.00
1.00	1.00	4.00	0.57	2.00	1.00	0.00	2.00	0.00
1.00	1.00	2.90	0.58	1.00	0.00	0.00	2.00	1.00
1.00	3.00	4.00	0.67	2.00	1.00	0.00	1.00	2.00
0.00	1.00	3.40	0.57	2.00	3.00	0.00	2.00	0.00
1.00	3.00	3.80	1.27	1.00	0.00	0.00	2.00	1.00
0.00	2.00	4.40	0.40	2.00	4.00	1.00	3.00	0.00
1.00	3.00	7.30	0.38	3.00	5.00	1.00	1.00	0.00
0.00	2.00	6.85	0.29	3.00	3.00	0.00	3.00	3.00
0.00	2.00	6.10	1.22	1.00	4.00	1.00	3.00	0.00
1.00	1.00	6.80	0.68	2.00	3.00	0.00	2.00	0.00
1.00	1.00	4.60	0.33	3.00	2.00	0.00	2.00	0.00
1.00	3.00	1.90	0.95	1.00	0.00	0.00	3.00	1.00
0.00	1.00	7.20	0.36	3.00	5.00	1.00	2.00	3.00
1.00	3.00	4.20	1.05	1.00	4.00	1.00	2.00	0.00
0.00	2.00	9.20	0.38	3.00	4.00	1.00	3.00	3.00
1.00	3.00	2.60	1.30	1.00	0.00	0.00	2.00	0.00
0.00	2.00	6.30	0.48	3.00	2.00	0.00	3.00	0.00
0.00	2.00	7.20	0.34	3.00	3.00	0.00	3.00	3.00
0.00	1.00	4.10	0.68	2.00	4.00	1.00	3.00	0.00
0.00	2.00	2.30	0.46	1.00	0.00	0.00	3.00	1.00
0.00	2.00	7.90	0.36	3.00	4.00	1.00	3.00	3.00
0.00	1.00	7.70	0.77	2.00	1.00	0.00	2.00	0.00
0.00	2.00	3.60	0.72	1.00	2.00	0.00	3.00	1.00
1.00	2.00	5.80	0.48	3.00	5.00	1.00	3.00	0.00
1.00	2.00	4.30	0.61	2.00	1.00	0.00	3.00	0.00
1.00	2.00	3.70	0.46	2.00	1.00	0.00	3.00	2.00
2.00	1.00	4.80	0.44	2.00	4.00	1.00	3.00	0.00
1.00	2.00	4.10	1.03	1.00	0.00	0.00	2.00	0.00
0.00	2.00	8.10	0.54	3.00	3.00	0.00	3.00	3.00
1.00	2.00	7.10	1.18	2.00	2.00	0.00	2.00	0.00
0.00	2.00	5.30	0.29	3.00	5.00	1.00	3.00	0.00
0.00	2.00	3.30	1.10	1.00	0.00	0.00	3.00	0.00
1.00	2.00	5.70	0.52	2.00	3.00	0.00	3.00	2.00
0.00	2.00	6.80	0.40	3.00	4.00	1.00	3.00	3.00
0.00	2.00	5.50	0.50	2.00	3.00	0.00	3.00	0.00
1.00	2.00	6.30	0.63	2.00	5.00	1.00	3.00	0.00
2.00	3.00	5.50	0.37	3.00	3.00	0.00	3.00	3.00
0.00	1.00	5.60	0.80	2.00	4.00	1.00	3.00	0.00
2.00	3.00	9.90	0.41	3.00	2.00	0.00	2.00	3.00
1.00	2.00	4.80	0.25	3.00	5.00	1.00	3.00	3.00
1.00	3.00	4.90	0.35	3.00	2.00	0.00	2.00	0.00
1.00	2.00	4.30	0.86	1.00	0.00	0.00	3.00	1.00
1.00	2.00	5.10	0.43	3.00	2.00	0.00	2.00	0.00
0.00	2.00	5.60	0.51	2.00	1.00	0.00	3.00	2.00
1.00	3.00	2.00	0.67	1.00	6.00	1.00	2.00	0.00
2.00	3.00	3.50	0.70	1.00	7.00	1.00	3.00	0.00

0.00	2.00	6.10	0.27	3.00	5.00	1.00	3.00	3.00
0.00	1.00	4.40	0.55	2.00	7.00	1.00	2.00	0.00
2.00	1.00	3.00	0.60	1.00	0.00	0.00	1.00	1.00
1.00	1.00	4.20	1.40	1.00	0.00	0.00	3.00	0.00
1.00	1.00	6.20	0.36	3.00	2.00	0.00	3.00	3.00
0.00	1.00	3.20	0.53	2.00	2.00	0.00	3.00	0.00
0.00	1.00	5.50	0.46	3.00	4.00	1.00	3.00	3.00
1.00	3.00	5.70	1.43	1.00	3.00	0.00	2.00	0.00
0.00	2.00	4.20	0.42	2.00	2.00	0.00	3.00	2.00
1.00	1.00	5.60	0.47	3.00	5.00	1.00	3.00	0.00
0.00	3.00	4.80	0.53	2.00	1.00	0.00	2.00	2.00
0.00	2.00	4.80	1.60	1.00	4.00	1.00	3.00	0.00
0.00	1.00	6.30	0.33	3.00	3.00	0.00	1.00	3.00
0.00	2.00	5.40	0.68	2.00	3.00	0.00	3.00	2.00
0.00	2.00	10.90	0.45	3.00	4.00	1.00	2.00	3.00
1.00	2.00	4.00	1.00	1.00	1.00	0.00	3.00	0.00
0.00	2.00	2.60	0.65	1.00	0.00	0.00	3.00	0.00
0.00	2.00	3.50	0.88	1.00	1.00	0.00	3.00	0.00
0.00	2.00	6.30	0.39	3.00	2.00	0.00	3.00	3.00
1.00	1.00	2.60	0.87	1.00	2.00	0.00	3.00	0.00
1.00	2.00	9.50	0.48	3.00	4.00	1.00	3.00	0.00
0.00	2.00	6.70	0.28	3.00	3.00	0.00	2.00	3.00
0.00	2.00	2.00	0.67	1.00	4.00	1.00	3.00	0.00
0.00	1.00	9.20	0.40	3.00	4.00	1.00	3.00	3.00
1.00	2.00	7.40	0.32	3.00	2.00	0.00	3.00	3.00
0.00	2.00	3.30	0.66	1.00	0.00	0.00	2.00	0.00
1.00	1.00	3.80	0.76	1.00	0.00	0.00	2.00	1.00
0.00	1.00	6.00	0.55	2.00	3.00	0.00	3.00	2.00
1.00	2.00	2.80	0.70	1.00	1.00	0.00	2.00	0.00
1.00	1.00	7.00	0.37	3.00	4.00	1.00	3.00	3.00
1.00	1.00	6.40	0.91	2.00	1.00	0.00	3.00	0.00
0.00	3.00	3.00	1.00	1.00	0.00	0.00	2.00	0.00
1.00	1.00	1.80	0.90	1.00	0.00	0.00	3.00	1.00
0.00	1.00	4.50	0.90	1.00	0.00	0.00	3.00	0.00
2.00	1.00	6.80	0.36	3.00	6.00	1.00	2.00	3.00
0.00	1.00	3.80	0.63	2.00	6.00	1.00	2.00	0.00
1.00	1.00	2.80	1.40	1.00	0.00	0.00	2.00	0.00
0.00	1.00	7.00	0.41	3.00	4.00	1.00	3.00	3.00
1.00	1.00	3.90	0.49	2.00	6.00	1.00	3.00	0.00
2.00	1.00	5.30	0.33	3.00	5.00	1.00	2.00	3.00
0.00	2.00	4.00	0.67	2.00	3.00	0.00	3.00	0.00
0.00	2.00	7.00	0.70	2.00	4.00	1.00	3.00	2.00
0.00	2.00	4.50	1.50	1.00	0.00	0.00	3.00	0.00
1.00	2.00	4.60	0.42	2.00	4.00	1.00	3.00	0.00
1.00	3.00	4.20	0.60	2.00	1.00	0.00	2.00	2.00
0.00	2.00	3.80	0.48	2.00	5.00	1.00	3.00	0.00
0.00	2.00	2.70	0.45	2.00	2.00	0.00	3.00	0.00

1.00	3.00	8.40	0.38	3.00	5.00	1.00	3.00	3.00
1.00	2.00	5.00	0.83	2.00	4.00	1.00	2.00	0.00
1.00	1.00	7.00	0.37	3.00	3.00	0.00	3.00	3.00
1.00	1.00	3.00	0.75	1.00	0.00	0.00	2.00	1.00
0.00	1.00	2.30	1.15	1.00	0.00	0.00	3.00	1.00
1.00	1.00	5.40	0.68	2.00	4.00	1.00	2.00	0.00
1.00	1.00	3.50	0.70	1.00	0.00	0.00	3.00	0.00
2.00	1.00	5.70	0.81	2.00	2.00	0.00	2.00	2.00
1.00	3.00	6.10	0.68	2.00	4.00	1.00	2.00	0.00
2.00	1.00	6.10	0.44	3.00	3.00	0.00	2.00	3.00
2.00	1.00	5.00	1.00	1.00	3.00	0.00	3.00	0.00
2.00	1.00	1.20	0.60	1.00	0.00	0.00	3.00	1.00
2.00	1.00	7.60	0.38	3.00	6.00	1.00	3.00	3.00
0.00	2.00	6.40	0.64	2.00	2.00	0.00	3.00	0.00
0.00	1.00	6.50	0.38	3.00	5.00	1.00	3.00	3.00
1.00	2.00	6.00	0.67	2.00	3.00	0.00	2.00	0.00
0.00	2.00	6.30	0.26	3.00	5.00	1.00	3.00	0.00
1.00	1.00	6.60	0.73	2.00	4.00	1.00	3.00	0.00
0.00	1.00	5.70	0.34	3.00	4.00	1.00	2.00	3.00
0.00	2.00	5.00	0.71	2.00	1.00	0.00	3.00	0.00
2.00	1.00	5.90	0.37	3.00	4.00	1.00	2.00	3.00
1.00	2.00	5.10	1.28	1.00	5.00	1.00	3.00	0.00
0.00	2.00	3.00	0.43	2.00	2.00	0.00	3.00	0.00
1.00	2.00	6.30	0.27	3.00	5.00	1.00	2.00	3.00
0.00	1.00	6.50	0.27	3.00	4.00	1.00	3.00	3.00
1.00	2.00	3.80	0.42	2.00	1.00	0.00	2.00	0.00
1.00	2.00	11.70	1.06	2.00	4.00	1.00	3.00	2.00
1.00	2.00	5.60	0.70	2.00	3.00	0.00	2.00	0.00
0.00	2.00	4.60	0.77	2.00	4.00	1.00	2.00	2.00
1.00	1.00	5.00	0.71	2.00	3.00	0.00	3.00	0.00
0.00	2.00	6.00	0.32	3.00	4.00	1.00	3.00	3.00
1.00	2.00	3.50	0.88	1.00	5.00	1.00	2.00	0.00
1.00	2.00	5.90	0.45	3.00	4.00	1.00	3.00	3.00
1.00	2.00	7.60	0.33	3.00	3.00	0.00	2.00	3.00
1.00	2.00	4.80	0.69	2.00	4.00	1.00	2.00	0.00
0.00	2.00	2.00	1.00	1.00	0.00	0.00	2.00	0.00
1.00	2.00	6.00	2.00	1.00	5.00	1.00	3.00	0.00
0.00	2.00	6.10	0.36	3.00	4.00	1.00	3.00	0.00
0.00	2.00	3.70	0.41	2.00	7.00	1.00	2.00	0.00
0.00	2.00	5.40	0.60	2.00	1.00	0.00	3.00	2.00
0.00	1.00	4.90	0.82	2.00	2.00	0.00	3.00	0.00
0.00	1.00	5.80	0.45	3.00	3.00	0.00	2.00	3.00
0.00	2.00	6.00	0.75	2.00	4.00	1.00	3.00	0.00
0.00	1.00	4.20	0.70	2.00	3.00	0.00	3.00	0.00
0.00	1.00	8.10	0.35	3.00	5.00	1.00	3.00	3.00
0.00	2.00	5.80	0.64	2.00	4.00	1.00	2.00	2.00
0.00	2.00	5.10	0.73	2.00	5.00	1.00	3.00	0.00

1.00	1.00	6.10	0.27	3.00	2.00	0.00	2.00	3.00
1.00	1.00	4.90	0.82	2.00	2.00	0.00	3.00	0.00
1.00	1.00	8.10	0.74	2.00	5.00	1.00	2.00	0.00
1.00	3.00	5.50	0.61	2.00	4.00	1.00	3.00	0.00
1.00	3.00	5.40	0.60	2.00	2.00	0.00	3.00	2.00
1.00	2.00	4.60	0.58	2.00	5.00	1.00	2.00	0.00
0.00	2.00	5.00	0.45	2.00	3.00	0.00	3.00	0.00
1.00	2.00	5.00	0.63	2.00	4.00	1.00	2.00	2.00
1.00	1.00	4.20	0.84	1.00	2.00	0.00	3.00	1.00
0.00	2.00	3.76	0.47	2.00	5.00	1.00	3.00	0.00
0.00	2.00	6.00	0.40	3.00	5.00	1.00	2.00	3.00
1.00	2.00	4.71	0.59	2.00	4.00	1.00	2.00	0.00
1.00	3.00	4.90	0.41	3.00	5.00	1.00	3.00	3.00
1.00	2.00	4.90	0.61	2.00	4.00	1.00	2.00	0.00
1.00	1.00	6.00	0.60	2.00	3.00	0.00	3.00	2.00
1.00	2.00	7.30	0.66	2.00	3.00	0.00	3.00	2.00
0.00	2.00	7.40	1.23	2.00	5.00	1.00	3.00	0.00
1.00	3.00	1.10	0.28	1.00	0.00	0.00	3.00	1.00
0.00	2.00	2.75	0.55	1.00	0.00	0.00	2.00	0.00
0.00	1.00	9.80	0.41	3.00	7.00	1.00	3.00	3.00
0.00	2.00	7.30	0.30	3.00	4.00	1.00	3.00	3.00
1.00	2.00	4.80	0.69	2.00	4.00	1.00	3.00	0.00
1.00	1.00	9.10	0.38	3.00	5.00	1.00	2.00	3.00
0.00	2.00	1.88	0.63	1.00	3.00	0.00	3.00	0.00
2.00	1.00	5.80	0.83	2.00	2.00	0.00	2.00	2.00
2.00	1.00	3.80	0.63	2.00	2.00	0.00	2.00	0.00
2.00	1.00	3.40	0.85	1.00	0.00	0.00	1.00	0.00
0.00	1.00	2.40	0.80	1.00	0.00	0.00	3.00	1.00
2.00	3.00	5.00	0.63	2.00	7.00	1.00	3.00	0.00
1.00	1.00	6.80	0.28	3.00	6.00	1.00	3.00	3.00
2.00	1.00	5.70	0.63	2.00	4.00	1.00	2.00	0.00
1.00	1.00	1.30	0.65	1.00	0.00	0.00	3.00	1.00
1.00	3.00	2.60	0.65	1.00	0.00	0.00	3.00	0.00
2.00	1.00	6.10	1.53	1.00	3.00	0.00	3.00	0.00
1.00	2.00	5.80	0.29	3.00	7.00	1.00	3.00	3.00
1.00	2.00	3.40	0.85	1.00	0.00	0.00	3.00	0.00
1.00	3.00	3.50	1.75	1.00	0.00	0.00	2.00	1.00
2.00	1.00	4.70	0.67	2.00	4.00	1.00	2.00	2.00
1.00	1.00	4.20	0.70	2.00	2.00	0.00	2.00	0.00
1.00	2.00	6.10	0.51	3.00	5.00	1.00	3.00	0.00
1.00	2.00	8.10	0.58	3.00	3.00	0.00	2.00	3.00
1.00	2.00	2.40	0.34	2.00	6.00	1.00	2.00	0.00
2.00	2.00	6.80	0.68	2.00	4.00	1.00	2.00	0.00
0.00	2.00	9.50	0.40	3.00	4.00	1.00	2.00	3.00
1.00	2.00	5.50	0.42	3.00	4.00	1.00	2.00	0.00
2.00	2.00	4.10	1.37	1.00	0.00	0.00	2.00	1.00
1.00	2.00	3.80	0.76	1.00	2.00	0.00	2.00	1.00

1.00	1.00	4.20	0.84	1.00	0.00	0.00	2.00	0.00
1.00	2.00	3.80	0.76	1.00	0.00	0.00	2.00	0.00
1.00	2.00	3.90	0.65	2.00	1.00	0.00	3.00	0.00
1.00	2.00	6.40	0.32	3.00	5.00	1.00	3.00	3.00
1.00	2.00	4.00	0.80	1.00	0.00	0.00	3.00	0.00
1.00	1.00	4.01	1.00	1.00	0.00	0.00	2.00	1.00
1.00	2.00	6.20	0.69	2.00	1.00	0.00	2.00	2.00
0.00	2.00	5.30	0.53	2.00	3.00	0.00	2.00	0.00
1.00	1.00	4.60	0.38	3.00	4.00	1.00	3.00	3.00
2.00	1.00	6.50	0.54	3.00	7.00	1.00	3.00	0.00
2.00	1.00	5.90	0.39	3.00	4.00	1.00	3.00	3.00
1.00	1.00	6.10	0.61	2.00	3.00	0.00	2.00	0.00
2.00	1.00	2.40	0.80	1.00	0.00	0.00	2.00	1.00
1.00	1.00	5.80	0.64	2.00	3.00	0.00	2.00	0.00
1.00	2.00	5.90	0.45	3.00	2.00	0.00	3.00	0.00
1.00	1.00	2.80	1.40	1.00	0.00	0.00	3.00	1.00
1.00	2.00	7.60	0.84	2.00	4.00	1.00	2.00	0.00
0.00	2.00	6.50	0.31	3.00	3.00	0.00	3.00	3.00
0.00	2.00	4.60	0.42	2.00	5.00	1.00	3.00	0.00
0.00	2.00	7.50	0.58	3.00	4.00	1.00	3.00	3.00
1.00	2.00	5.30	0.88	2.00	4.00	1.00	3.00	0.00
0.00	2.00	6.60	0.94	2.00	2.00	0.00	3.00	0.00
0.00	2.00	5.50	0.37	3.00	4.00	1.00	2.00	0.00
1.00	2.00	3.70	0.46	2.00	6.00	1.00	3.00	2.00
0.00	2.00	5.31	0.66	2.00	1.00	0.00	2.00	0.00
1.00	2.00	4.50	0.41	2.00	3.00	0.00	3.00	0.00
0.00	2.00	5.10	0.64	2.00	1.00	0.00	2.00	2.00
1.00	2.00	5.80	0.53	2.00	3.00	0.00	3.00	0.00
0.00	2.00	4.20	1.05	1.00	0.00	0.00	3.00	1.00
1.00	1.00	4.30	0.72	2.00	1.00	0.00	3.00	0.00
0.00	1.00	7.60	0.35	3.00	4.00	1.00	3.00	3.00
1.00	2.00	4.90	0.35	3.00	2.00	0.00	2.00	0.00
0.00	2.00	2.50	0.50	1.00	0.00	0.00	3.00	1.00
1.00	2.00	6.70	0.28	3.00	5.00	1.00	2.00	3.00
1.00	2.00	6.29	0.90	2.00	3.00	0.00	2.00	0.00
1.00	1.00	4.00	0.80	1.00	4.00	1.00	3.00	0.00
0.00	2.00	8.32	0.35	3.00	4.00	1.00	3.00	3.00
1.00	3.00	7.30	0.81	2.00	7.00	1.00	3.00	0.00
1.00	1.00	3.70	1.23	1.00	0.00	0.00	2.00	0.00
0.00	1.00	8.30	0.35	3.00	5.00	1.00	3.00	3.00
2.00	1.00	7.00	0.29	3.00	4.00	1.00	3.00	3.00
2.00	1.00	5.80	1.16	1.00	0.00	0.00	2.00	0.00
1.00	2.00	4.30	0.72	2.00	1.00	0.00	3.00	2.00
1.00	1.00	4.90	0.82	2.00	3.00	0.00	3.00	2.00
1.00	1.00	5.40	0.60	2.00	3.00	0.00	2.00	0.00
1.00	1.00	5.90	0.45	3.00	5.00	1.00	3.00	3.00
1.00	2.00	6.40	0.27	3.00	2.00	0.00	2.00	3.00

0.00	1.00	2.60	0.87	1.00	0.00	0.00	2.00	0.00
1.00	2.00	4.60	0.92	1.00	1.00	0.00	3.00	1.00
1.00	1.00	1.70	0.57	1.00	0.00	0.00	3.00	0.00
2.00	1.00	2.70	0.45	2.00	4.00	1.00	2.00	2.00
2.00	1.00	5.80	0.41	3.00	4.00	1.00	2.00	3.00
1.00	1.00	6.10	0.68	2.00	3.00	0.00	2.00	0.00
1.00	2.00	7.20	1.20	2.00	3.00	0.00	3.00	0.00
0.00	1.00	4.00	0.80	1.00	0.00	0.00	2.00	1.00
1.00	1.00	5.40	0.45	3.00	5.00	1.00	2.00	0.00
1.00	1.00	4.00	1.33	1.00	0.00	0.00	2.00	1.00
1.00	2.00	5.90	0.54	2.00	3.00	0.00	2.00	0.00
1.00	1.00	5.90	0.33	3.00	4.00	1.00	2.00	3.00
1.00	1.00	5.30	0.76	2.00	4.00	1.00	2.00	0.00
1.00	1.00	5.70	0.29	3.00	6.00	1.00	2.00	3.00
1.00	2.00	5.20	0.43	3.00	2.00	0.00	3.00	0.00
0.00	1.00	11.00	0.48	3.00	4.00	1.00	3.00	3.00
2.00	3.00	5.30	0.48	2.00	4.00	1.00	3.00	2.00
1.00	1.00	5.20	1.73	1.00	0.00	0.00	2.00	0.00
1.00	3.00	3.50	1.75	1.00	0.00	0.00	3.00	1.00
1.00	1.00	5.90	0.54	2.00	4.00	1.00	2.00	2.00
1.00	1.00	6.40	0.64	2.00	2.00	0.00	2.00	0.00
1.00	1.00	5.50	0.42	3.00	4.00	1.00	3.00	3.00
2.00	3.00	3.80	0.48	2.00	6.00	1.00	2.00	2.00
1.00	1.00	2.70	0.68	1.00	4.00	1.00	3.00	0.00
1.00	1.00	3.10	1.55	1.00	0.00	0.00	3.00	0.00
1.00	1.00	4.30	0.86	1.00	3.00	0.00	3.00	1.00
1.00	2.00	5.80	0.53	2.00	6.00	1.00	2.00	0.00
1.00	1.00	4.40	0.40	2.00	6.00	1.00	3.00	2.00
2.00	1.00	3.40	0.68	1.00	0.00	0.00	3.00	1.00
1.00	1.00	6.90	0.30	3.00	6.00	1.00	3.00	0.00
1.00	2.00	2.60	1.30	1.00	0.00	0.00	3.00	0.00
2.00	2.00	4.30	0.86	1.00	6.00	1.00	2.00	0.00
2.00	2.00	3.80	0.38	2.00	4.00	1.00	2.00	2.00
0.00	1.00	5.00	0.31	3.00	3.00	0.00	3.00	3.00
1.00	1.00	4.50	0.56	2.00	4.00	1.00	3.00	0.00
1.00	1.00	6.40	0.71	2.00	5.00	1.00	3.00	2.00
1.00	1.00	6.70	0.52	3.00	5.00	1.00	3.00	0.00
1.00	1.00	6.40	0.58	2.00	4.00	1.00	2.00	0.00
0.00	2.00	5.10	0.85	2.00	1.00	0.00	3.00	0.00
1.00	1.00	7.90	0.44	3.00	4.00	1.00	2.00	3.00
1.00	1.00	4.70	0.43	2.00	3.00	0.00	3.00	2.00
1.00	2.00	3.50	0.58	2.00	0.00	0.00	2.00	0.00
1.00	2.00	4.90	0.45	2.00	3.00	0.00	3.00	2.00
0.00	2.00	6.90	0.63	2.00	4.00	1.00	3.00	2.00
2.00	1.00	3.50	0.44	2.00	3.00	0.00	3.00	2.00
1.00	2.00	2.90	0.73	1.00	0.00	0.00	3.00	0.00
0.00	2.00	5.90	0.74	2.00	3.00	0.00	3.00	0.00

2.00	1.00	6.00	0.38	3.00	4.00	1.00	2.00	3.00
1.00	1.00	5.80	1.16	1.00	3.00	0.00	3.00	0.00
1.00	2.00	5.10	0.85	2.00	1.00	0.00	3.00	2.00
1.00	1.00	2.50	0.42	2.00	3.00	0.00	2.00	2.00
0.00	1.00	4.90	0.38	3.00	4.00	1.00	2.00	0.00
1.00	1.00	7.60	0.40	3.00	6.00	1.00	2.00	3.00
2.00	3.00	5.70	0.44	3.00	2.00	0.00	2.00	0.00
1.00	1.00	4.20	1.40	1.00	3.00	0.00	2.00	0.00
1.00	3.00	6.50	0.41	3.00	5.00	1.00	2.00	3.00
2.00	1.00	4.40	0.55	2.00	4.00	1.00	2.00	0.00
1.00	3.00	5.90	0.84	2.00	6.00	1.00	3.00	0.00
1.00	1.00	4.80	0.96	1.00	0.00	0.00	3.00	1.00
1.00	1.00	4.40	0.44	2.00	7.00	1.00	2.00	0.00
1.00	3.00	6.30	0.90	2.00	7.00	1.00	3.00	0.00
1.00	3.00	6.00	0.46	3.00	7.00	1.00	3.00	0.00
1.00	3.00	6.60	0.39	3.00	7.00	1.00	2.00	0.00
2.00	3.00	3.60	0.72	1.00	0.00	0.00	3.00	1.00
1.00	3.00	2.40	0.80	1.00	7.00	1.00	3.00	0.00
1.00	3.00	5.10	0.57	2.00	7.00	1.00	3.00	2.00
0.00	2.00	4.80	0.27	3.00	7.00	1.00	3.00	3.00
2.00	2.00	7.40	0.67	2.00	6.00	1.00	2.00	0.00
2.00	3.00	7.50	0.31	3.00	4.00	1.00	2.00	3.00
1.00	3.00	6.90	0.29	3.00	5.00	1.00	2.00	0.00
1.00	1.00	0.60	0.30	1.00	0.00	0.00	3.00	0.00
1.00	1.00	5.80	0.64	2.00	3.00	0.00	2.00	0.00
1.00	1.00	5.00	0.63	2.00	5.00	1.00	3.00	2.00
1.00	1.00	2.50	1.25	1.00	0.00	0.00	3.00	1.00
0.00	1.00	1.30	0.33	1.00	0.00	0.00	3.00	0.00
1.00	1.00	1.60	0.80	1.00	0.00	0.00	2.00	1.00
1.00	1.00	5.20	1.30	1.00	3.00	0.00	2.00	0.00
0.00	1.00	6.50	0.54	3.00	3.00	0.00	2.00	0.00
2.00	3.00	8.60	1.23	2.00	3.00	0.00	1.00	0.00
0.00	1.00	2.40	0.14	3.00	6.00	1.00	3.00	0.00
1.00	2.00	1.90	0.48	1.00	0.00	0.00	3.00	1.00
1.00	2.00	3.90	0.78	1.00	0.00	0.00	3.00	1.00
2.00	1.00	6.50	0.46	3.00	4.00	1.00	3.00	0.00
2.00	2.00	5.10	1.02	1.00	0.00	0.00	1.00	0.00
2.00	3.00	3.00	0.60	1.00	0.00	0.00	2.00	0.00
2.00	3.00	4.50	1.13	1.00	3.00	0.00	3.00	0.00
1.00	1.00	2.60	0.65	1.00	0.00	0.00	1.00	0.00
0.00	2.00	9.30	0.40	3.00	4.00	1.00	3.00	3.00
2.00	3.00	3.90	0.49	2.00	5.00	1.00	2.00	0.00
2.00	2.00	5.60	0.51	2.00	3.00	0.00	2.00	2.00
2.00	1.00	5.70	0.63	2.00	4.00	1.00	2.00	2.00
1.00	1.00	2.50	0.83	1.00	0.00	0.00	2.00	0.00
0.00	1.00	7.50	0.83	2.00	5.00	1.00	2.00	0.00
2.00	3.00	5.20	0.65	2.00	2.00	0.00	2.00	0.00

2.00	3.00	5.40	0.36	3.00	6.00	1.00	2.00	3.00
2.00	3.00	5.00	0.42	3.00	6.00	1.00	2.00	3.00
2.00	3.00	6.70	1.12	2.00	5.00	1.00	2.00	0.00
0.00	1.00	4.60	0.26	3.00	5.00	1.00	3.00	3.00
1.00	2.00	2.70	1.35	1.00	0.00	0.00	1.00	0.00
2.00	3.00	7.20	0.31	3.00	6.00	1.00	3.00	3.00
1.00	1.00	4.70	0.67	2.00	1.00	0.00	3.00	0.00
0.00	1.00	5.20	0.40	3.00	4.00	1.00	3.00	3.00
1.00	2.00	5.86	0.65	2.00	4.00	1.00	3.00	0.00
0.00	2.00	1.20	0.24	1.00	0.00	0.00	3.00	1.00
0.00	1.00	4.60	0.92	1.00	4.00	1.00	3.00	0.00
0.00	1.00	2.80	1.40	1.00	0.00	0.00	3.00	1.00
0.00	2.00	6.50	0.93	2.00	4.00	1.00	3.00	0.00
0.00	2.00	4.90	0.45	2.00	6.00	1.00	3.00	2.00
0.00	2.00	3.80	1.90	1.00	0.00	0.00	3.00	0.00
1.00	2.00	4.70	0.39	3.00	5.00	1.00	1.00	3.00
1.00	2.00	8.10	0.90	2.00	5.00	1.00	3.00	0.00
2.00	3.00	5.30	0.66	2.00	5.00	1.00	2.00	0.00
1.00	2.00	7.60	1.27	2.00	5.00	1.00	3.00	0.00
1.00	2.00	3.70	0.62	2.00	0.00	0.00	2.00	2.00
2.00	2.00	4.40	0.55	2.00	4.00	1.00	3.00	0.00
2.00	1.00	6.80	0.52	3.00	6.00	1.00	3.00	3.00
1.00	1.00	4.60	0.46	2.00	6.00	1.00	2.00	0.00
1.00	1.00	2.60	0.87	1.00	0.00	0.00	2.00	0.00
2.00	1.00	6.90	0.38	3.00	4.00	1.00	3.00	3.00
1.00	1.00	4.00	0.67	2.00	5.00	1.00	2.00	0.00
1.00	2.00	5.80	0.64	2.00	4.00	1.00	3.00	0.00
1.00	1.00	1.80	0.90	1.00	0.00	0.00	2.00	0.00
1.00	1.00	3.90	0.78	1.00	0.00	0.00	2.00	1.00
2.00	1.00	6.80	0.32	3.00	6.00	1.00	3.00	3.00
1.00	2.00	7.20	1.03	2.00	4.00	1.00	2.00	0.00
2.00	2.00	6.90	0.46	3.00	4.00	1.00	2.00	0.00
1.00	2.00	3.50	1.75	1.00	0.00	0.00	2.00	1.00
1.00	2.00	6.90	0.31	3.00	4.00	1.00	3.00	3.00
0.00	2.00	4.20	1.05	1.00	3.00	0.00	3.00	0.00
1.00	2.00	3.90	0.98	1.00	0.00	0.00	2.00	0.00
1.00	1.00	9.30	0.39	3.00	4.00	1.00	2.00	3.00
2.00	2.00	3.30	1.65	1.00	0.00	0.00	2.00	0.00
0.00	2.00	4.20	1.05	1.00	0.00	0.00	3.00	1.00
1.00	2.00	1.30	0.65	1.00	0.00	0.00	2.00	0.00
1.00	2.00	9.10	0.38	3.00	5.00	1.00	3.00	3.00
0.00	2.00	5.80	0.73	2.00	5.00	1.00	3.00	0.00
1.00	2.00	4.30	0.54	2.00	4.00	1.00	2.00	0.00
0.00	2.00	7.10	0.34	3.00	4.00	1.00	3.00	3.00
0.00	1.00	3.80	0.54	2.00	7.00	1.00	2.00	2.00
0.00	2.00	5.10	0.39	3.00	4.00	1.00	3.00	0.00
1.00	1.00	4.60	0.58	2.00	6.00	1.00	3.00	2.00

0.00	2.00	6.80	0.68	2.00	2.00	0.00	2.00	0.00
1.00	2.00	5.30	0.41	3.00	4.00	1.00	2.00	3.00
0.00	2.00	6.40	0.58	2.00	3.00	0.00	3.00	0.00
1.00	2.00	6.30	0.57	2.00	4.00	1.00	3.00	2.00
2.00	2.00	4.30	0.33	3.00	1.00	0.00	2.00	3.00
0.00	2.00	2.60	0.87	1.00	0.00	0.00	3.00	0.00
0.00	2.00	4.70	0.52	2.00	6.00	1.00	3.00	0.00
1.00	2.00	5.70	0.41	3.00	7.00	1.00	3.00	3.00
2.00	2.00	4.40	0.88	1.00	7.00	1.00	2.00	0.00
0.00	2.00	5.70	0.81	2.00	6.00	1.00	3.00	2.00
0.00	2.00	7.00	0.54	3.00	7.00	1.00	3.00	3.00
1.00	2.00	5.00	0.50	2.00	7.00	1.00	3.00	2.00
1.00	2.00	6.10	0.61	2.00	7.00	1.00	3.00	0.00
0.00	1.00	8.00	0.89	2.00	7.00	1.00	3.00	0.00
0.00	1.00	4.10	1.37	1.00	0.00	0.00	3.00	1.00
1.00	2.00	4.90	0.70	2.00	7.00	1.00	2.00	2.00
0.00	2.00	3.80	0.76	1.00	0.00	0.00	3.00	1.00
0.00	2.00	6.10	0.55	2.00	7.00	1.00	3.00	0.00
1.00	2.00	3.60	0.72	1.00	0.00	0.00	2.00	0.00
1.00	2.00	4.00	1.33	1.00	0.00	0.00	2.00	1.00
0.00	2.00	7.70	0.86	2.00	7.00	1.00	2.00	0.00
1.00	3.00	4.40	0.22	3.00	7.00	1.00	3.00	3.00
1.00	2.00	6.20	0.34	3.00	3.00	0.00	3.00	3.00
1.00	2.00	3.00	1.50	1.00	0.00	0.00	2.00	0.00
0.00	2.00	2.20	0.55	1.00	0.00	0.00	3.00	1.00
0.00	2.00	6.50	1.63	1.00	0.00	0.00	3.00	0.00
1.00	2.00	4.30	0.48	2.00	4.00	1.00	2.00	0.00
1.00	2.00	2.30	0.77	1.00	0.00	0.00	3.00	1.00
1.00	2.00	8.30	0.35	3.00	4.00	1.00	2.00	3.00
1.00	2.00	3.00	1.50	1.00	0.00	0.00	3.00	0.00
0.00	2.00	6.30	0.90	2.00	4.00	1.00	1.00	0.00
1.00	2.00	7.80	0.33	3.00	5.00	1.00	2.00	3.00
2.00	1.00	8.20	0.34	3.00	4.00	1.00	3.00	3.00
0.00	1.00	7.30	0.66	2.00	5.00	1.00	3.00	0.00
1.00	1.00	10.20	0.43	3.00	5.00	1.00	3.00	3.00
1.00	2.00	6.40	0.53	3.00	1.00	0.00	3.00	3.00
0.00	1.00	4.50	0.50	2.00	6.00	1.00	3.00	0.00
1.00	1.00	4.60	0.35	3.00	4.00	1.00	2.00	3.00
0.00	1.00	1.80	0.60	1.00	0.00	0.00	3.00	0.00
0.00	1.00	10.00	0.48	3.00	6.00	1.00	3.00	3.00
0.00	2.00	8.50	0.94	2.00	2.00	0.00	3.00	0.00
0.00	2.00	5.60	0.93	2.00	2.00	0.00	3.00	0.00
0.00	2.00	8.10	0.35	3.00	5.00	1.00	3.00	3.00
2.00	2.00	3.30	0.83	1.00	0.00	0.00	2.00	0.00
0.00	2.00	1.30	0.65	1.00	0.00	0.00	3.00	1.00
0.00	2.00	3.90	0.78	1.00	5.00	1.00	3.00	0.00
1.00	2.00	10.70	0.49	3.00	5.00	1.00	3.00	3.00

1.00	2.00	7.30	0.30	3.00	5.00	1.00	3.00	3.00
0.00	1.00	7.10	0.89	2.00	5.00	1.00	3.00	0.00
0.00	3.00	4.40	0.63	2.00	5.00	1.00	3.00	0.00
0.00	2.00	6.10	1.53	1.00	5.00	1.00	3.00	0.00
1.00	2.00	9.90	0.41	3.00	2.00	0.00	2.00	3.00
1.00	3.00	5.80	0.53	2.00	2.00	0.00	3.00	0.00
0.00	1.00	7.10	0.51	3.00	3.00	0.00	3.00	3.00
1.00	2.00	5.50	0.55	2.00	4.00	1.00	3.00	0.00
1.00	2.00	6.10	0.31	3.00	5.00	1.00	3.00	3.00
1.00	2.00	4.10	0.51	2.00	5.00	1.00	3.00	0.00
0.00	2.00	10.50	0.46	3.00	4.00	1.00	3.00	3.00
1.00	1.00	3.90	0.78	1.00	5.00	1.00	3.00	0.00
0.00	1.00	5.60	0.62	2.00	5.00	1.00	3.00	0.00
0.00	3.00	5.60	1.12	1.00	0.00	0.00	3.00	1.00
1.00	3.00	2.50	0.83	1.00	0.00	0.00	3.00	0.00
1.00	2.00	7.40	0.31	3.00	5.00	1.00	3.00	3.00
1.00	1.00	7.60	0.69	2.00	6.00	1.00	3.00	0.00
0.00	2.00	1.80	0.90	1.00	0.00	0.00	3.00	1.00
0.00	1.00	3.80	0.63	2.00	3.00	0.00	3.00	2.00
0.00	1.00	9.00	1.00	2.00	5.00	1.00	3.00	0.00
1.00	2.00	4.50	1.13	1.00	0.00	0.00	3.00	0.00
0.00	2.00	7.80	0.33	3.00	5.00	1.00	3.00	3.00
0.00	2.00	7.40	0.32	3.00	4.00	1.00	3.00	3.00
1.00	2.00	5.50	0.92	2.00	3.00	0.00	2.00	0.00
0.00	2.00	9.70	0.46	3.00	2.00	0.00	3.00	3.00
1.00	1.00	6.30	0.53	3.00	4.00	1.00	2.00	0.00
1.00	2.00	5.10	0.43	3.00	7.00	1.00	3.00	3.00
0.00	1.00	4.90	0.45	2.00	7.00	1.00	3.00	0.00
0.00	1.00	5.30	0.76	2.00	3.00	0.00	3.00	0.00
0.00	1.00	12.40	0.59	3.00	0.00	0.00	3.00	0.00
2.00	3.00	5.70	0.44	3.00	4.00	1.00	3.00	3.00
2.00	1.00	5.50	0.69	2.00	5.00	1.00	3.00	0.00
0.00	1.00	7.70	0.43	3.00	3.00	0.00	2.00	3.00
2.00	3.00	5.30	0.59	2.00	6.00	1.00	3.00	0.00
1.00	3.00	3.20	0.80	1.00	0.00	0.00	2.00	0.00
0.00	2.00	3.80	1.27	1.00	0.00	0.00	3.00	1.00
2.00	3.00	3.80	1.27	1.00	0.00	0.00	3.00	1.00
0.00	1.00	6.80	0.57	3.00	2.00	0.00	2.00	0.00
1.00	2.00	9.60	0.53	3.00	4.00	1.00	3.00	3.00
1.00	3.00	3.00	0.75	1.00	0.00	0.00	2.00	0.00
1.00	3.00	1.80	0.90	1.00	0.00	0.00	3.00	1.00
2.00	3.00	3.70	0.62	2.00	0.00	0.00	2.00	0.00
1.00	1.00	4.30	1.43	1.00	0.00	0.00	3.00	0.00
1.00	1.00	4.00	0.57	2.00	4.00	1.00	2.00	0.00
1.00	3.00	2.90	0.97	1.00	0.00	0.00	3.00	0.00
1.00	1.00	3.70	1.23	1.00	0.00	0.00	3.00	0.00
1.00	1.00	3.20	0.40	2.00	3.00	0.00	3.00	2.00

1.00	1.00	3.50	0.88	1.00	0.00	0.00	2.00	0.00
1.00	3.00	4.30	1.08	1.00	0.00	0.00	2.00	1.00
0.00	3.00	1.40	0.28	1.00	0.00	0.00	2.00	0.00
0.00	3.00	1.30	0.43	1.00	0.00	0.00	2.00	1.00
1.00	1.00	4.50	0.38	3.00	4.00	1.00	3.00	0.00
2.00	3.00	6.00	0.33	3.00	3.00	0.00	3.00	3.00
1.00	2.00	5.50	0.61	2.00	1.00	0.00	3.00	0.00
1.00	2.00	4.60	0.51	2.00	1.00	0.00	3.00	2.00
1.00	1.00	6.20	1.24	1.00	0.00	0.00	3.00	0.00
2.00	3.00	6.40	0.80	2.00	3.00	0.00	2.00	2.00
2.00	3.00	2.10	1.05	1.00	0.00	0.00	3.00	1.00
2.00	3.00	3.70	1.23	1.00	0.00	0.00	3.00	0.00
2.00	1.00	3.30	1.10	1.00	0.00	0.00	3.00	0.00
1.00	1.00	4.20	0.84	1.00	0.00	0.00	3.00	1.00

Interval3	Interval4	Parity2	Birthinterv	Birthinterv	Agechild3	Interaction	Interaction	Zscore
3.00	0.00	2.00	47.00	4.00	2.00	94.00	94.00	16.50
2.00	0.00	1.00	31.00	3.00	1.00	31.00	62.00	0.70
1.00	1.00	1.00	8.00	1.00	2.00	16.00	16.00	15.00
1.00	1.00	2.00	15.00	1.00	1.00	15.00	15.00	-1.20
4.00	0.00	2.00	61.00	5.00	1.00	61.00	61.00	-11.90
3.00	0.00	1.00	46.00	4.00	1.00	46.00	46.00	-1.50
1.00	1.00	1.00	8.00	1.00	2.00	16.00	16.00	7.40
1.00	1.00	1.00	10.00	1.00	2.00	20.00	20.00	2.50
4.00	0.00	1.00	72.00	5.00	1.00	72.00	72.00	-6.00
1.00	1.00	1.00	15.00	1.00	1.00	15.00	15.00	-3.50
1.00	1.00	2.00	16.00	1.00	1.00	16.00	16.00	-10.00
3.00	0.00	1.00	38.00	4.00	1.00	38.00	38.00	-3.30
1.00	1.00	1.00	19.00	2.00	1.00	19.00	19.00	-7.30
1.00	1.00	1.00	15.00	1.00	1.00	15.00	15.00	-3.60
4.00	0.00	1.00	95.00	5.00	1.00	95.00	95.00	-16.60
1.00	1.00	1.00	16.00	1.00	1.00	16.00	32.00	-7.40
3.00	0.00	1.00	37.00	4.00	1.00	37.00	37.00	-6.90
1.00	1.00	2.00	12.00	1.00	1.00	12.00	24.00	-8.70
1.00	1.00	2.00	21.00	2.00	1.00	21.00	21.00	-12.30
4.00	0.00	2.00	84.00	5.00	1.00	84.00	84.00	-10.30
4.00	0.00	1.00	76.00	5.00	1.00	76.00	152.00	-5.50
1.00	1.00	1.00	14.00	1.00	1.00	14.00	28.00	-5.50
4.00	0.00	2.00	64.00	5.00	1.00	64.00	64.00	1.80
1.00	1.00	1.00	10.00	1.00	2.00	20.00	10.00	12.20
3.00	0.00	2.00	53.00	4.00	2.00	106.00	106.00	3.50
4.00	0.00	2.00	80.00	5.00	1.00	80.00	160.00	-9.30
3.00	0.00	2.00	44.00	4.00	1.00	44.00	88.00	-6.20
1.00	1.00	1.00	15.00	1.00	1.00	15.00	15.00	4.90
4.00	0.00	2.00	83.00	5.00	2.00	166.00	83.00	8.40
1.00	1.00	2.00	12.00	1.00	1.00	12.00	24.00	0.20
1.00	1.00	1.00	13.00	1.00	1.00	13.00	13.00	-2.10
1.00	1.00	1.00	17.00	1.00	1.00	17.00	17.00	-3.60
4.00	0.00	1.00	61.00	5.00	1.00	61.00	61.00	3.30
2.00	0.00	2.00	28.00	3.00	1.00	28.00	28.00	-6.10
1.00	1.00	2.00	12.00	1.00	1.00	12.00	24.00	-3.20
1.00	1.00	1.00	13.00	1.00	1.00	13.00	13.00	-1.10
2.00	0.00	1.00	25.00	3.00	2.00	50.00	25.00	6.50
1.00	1.00	1.00	21.00	2.00	2.00	42.00	42.00	6.50
1.00	1.00	1.00	10.00	1.00	1.00	10.00	10.00	1.50
1.00	1.00	2.00	13.00	1.00	1.00	13.00	13.00	4.50
3.00	0.00	2.00	47.00	4.00	2.00	94.00	47.00	15.50
1.00	1.00	2.00	7.00	1.00	2.00	14.00	7.00	2.70
2.00	0.00	2.00	30.00	3.00	2.00	60.00	30.00	3.50
3.00	0.00	2.00	49.00	4.00	2.00	98.00	98.00	4.40
3.00	0.00	2.00	40.00	4.00	2.00	80.00	40.00	12.80
2.00	0.00	2.00	35.00	3.00	2.00	70.00	35.00	12.80

1.00	1.00	2.00	21.00	2.00	2.00	42.00	21.00	4.10
1.00	1.00	1.00	15.00	1.00	1.00	15.00	15.00	-3.30
3.00	0.00	1.00	51.00	4.00	1.00	51.00	51.00	-2.20
4.00	0.00	1.00	66.00	5.00	1.00	66.00	132.00	-2.20
1.00	1.00	1.00	15.00	1.00	1.00	15.00	30.00	-5.10
4.00	0.00	1.00	60.00	5.00	1.00	60.00	120.00	-5.80
1.00	1.00	2.00	12.00	1.00	1.00	12.00	24.00	-4.00
1.00	1.00	1.00	12.00	1.00	2.00	24.00	12.00	9.50
3.00	0.00	2.00	48.00	4.00	2.00	96.00	48.00	14.50
1.00	1.00	2.00	19.00	2.00	1.00	19.00	38.00	-2.50
1.00	1.00	1.00	13.00	1.00	1.00	13.00	13.00	-2.50
1.00	1.00	1.00	10.00	1.00	2.00	20.00	10.00	-0.60
3.00	0.00	2.00	46.00	4.00	1.00	46.00	46.00	-11.50
2.00	0.00	1.00	29.00	3.00	2.00	58.00	58.00	7.50
1.00	1.00	1.00	15.00	1.00	1.00	15.00	30.00	-6.50
2.00	0.00	2.00	29.00	3.00	2.00	58.00	29.00	4.50
1.00	1.00	1.00	18.00	2.00	1.00	18.00	36.00	-11.90
1.00	1.00	2.00	23.00	2.00	2.00	46.00	46.00	-3.50
3.00	0.00	2.00	45.00	4.00	2.00	90.00	90.00	11.80
1.00	1.00	2.00	16.00	1.00	1.00	16.00	16.00	-1.30
2.00	0.00	2.00	30.00	3.00	1.00	30.00	30.00	-3.50
2.00	0.00	2.00	30.00	3.00	2.00	60.00	30.00	16.10
1.00	1.00	1.00	13.00	1.00	1.00	13.00	26.00	11.10
2.00	0.00	2.00	35.00	3.00	1.00	35.00	35.00	-0.60
1.00	1.00	2.00	8.00	1.00	2.00	16.00	8.00	9.80
1.00	1.00	2.00	16.00	1.00	1.00	16.00	16.00	-3.50
2.00	0.00	2.00	27.00	3.00	1.00	27.00	27.00	-1.60
1.00	1.00	2.00	20.00	2.00	1.00	20.00	40.00	2.80
1.00	1.00	1.00	20.00	2.00	1.00	20.00	40.00	-5.50
2.00	0.00	2.00	33.00	3.00	2.00	66.00	66.00	9.50
1.00	1.00	1.00	15.00	1.00	1.00	15.00	30.00	4.50
1.00	1.00	2.00	18.00	2.00	2.00	36.00	36.00	5.50
1.00	1.00	2.00	18.00	2.00	1.00	18.00	18.00	-4.30
3.00	0.00	2.00	57.00	4.00	1.00	57.00	114.00	2.50
3.00	0.00	2.00	55.00	4.00	2.00	110.00	110.00	6.50
1.00	1.00	2.00	9.00	1.00	1.00	9.00	9.00	1.50
1.00	1.00	2.00	10.00	1.00	1.00	10.00	10.00	3.50
3.00	0.00	2.00	46.00	4.00	2.00	92.00	46.00	-2.50
1.00	1.00	2.00	16.00	1.00	1.00	16.00	32.00	-0.80
2.00	0.00	1.00	25.00	3.00	2.00	50.00	25.00	6.50
3.00	0.00	2.00	36.00	4.00	2.00	72.00	72.00	-1.50
1.00	1.00	1.00	10.00	1.00	2.00	20.00	20.00	-1.40
2.00	0.00	2.00	33.00	3.00	1.00	33.00	33.00	-4.30
1.00	1.00	1.00	10.00	1.00	2.00	20.00	20.00	8.60
4.00	0.00	2.00	61.00	5.00	1.00	61.00	122.00	0.60
1.00	1.00	1.00	21.00	2.00	1.00	21.00	21.00	-7.90
1.00	1.00	2.00	18.00	2.00	1.00	18.00	18.00	-1.30

2.00	0.00	2.00	34.00	3.00	2.00	68.00	34.00	9.80
1.00	1.00	1.00	14.00	1.00	1.00	14.00	28.00	2.70
3.00	0.00	1.00	43.00	4.00	1.00	43.00	43.00	-2.50
1.00	1.00	2.00	16.00	1.00	1.00	16.00	16.00	-5.90
2.00	0.00	2.00	31.00	3.00	2.00	62.00	31.00	-1.90
1.00	1.00	2.00	17.00	1.00	1.00	17.00	34.00	-7.40
3.00	0.00	2.00	48.00	4.00	2.00	96.00	96.00	1.50
1.00	1.00	1.00	15.00	1.00	1.00	15.00	15.00	1.00
3.00	0.00	2.00	57.00	4.00	1.00	57.00	57.00	-5.30
1.00	1.00	2.00	11.00	1.00	2.00	22.00	22.00	7.40
3.00	0.00	1.00	39.00	4.00	1.00	39.00	39.00	-0.20
1.00	1.00	2.00	17.00	1.00	1.00	17.00	17.00	-0.30
3.00	0.00	1.00	53.00	4.00	2.00	106.00	53.00	14.00
3.00	0.00	2.00	38.00	4.00	1.00	38.00	76.00	1.50
3.00	0.00	1.00	48.00	4.00	2.00	96.00	96.00	18.80
1.00	1.00	2.00	15.00	1.00	1.00	15.00	30.00	-5.20
1.00	1.00	2.00	20.00	2.00	1.00	20.00	40.00	-11.50
1.00	1.00	2.00	18.00	2.00	1.00	18.00	36.00	-3.50
3.00	0.00	2.00	44.00	4.00	2.00	88.00	44.00	9.70
1.00	1.00	2.00	15.00	1.00	1.00	15.00	30.00	-15.60
1.00	1.00	2.00	18.00	2.00	2.00	36.00	18.00	5.30
3.00	0.00	1.00	38.00	4.00	2.00	76.00	76.00	-1.20
1.00	1.00	2.00	19.00	2.00	1.00	19.00	19.00	-8.10
3.00	0.00	2.00	36.00	4.00	2.00	72.00	72.00	17.00
3.00	0.00	2.00	42.00	4.00	2.00	84.00	84.00	13.60
1.00	1.00	1.00	19.00	2.00	1.00	19.00	38.00	-6.10
2.00	0.00	1.00	29.00	3.00	1.00	29.00	58.00	-5.40
2.00	0.00	2.00	25.00	3.00	1.00	25.00	50.00	6.10
1.00	1.00	1.00	18.00	2.00	1.00	18.00	18.00	-10.70
3.00	0.00	2.00	41.00	4.00	2.00	82.00	41.00	2.70
1.00	1.00	2.00	16.00	1.00	1.00	16.00	32.00	-1.50
1.00	1.00	1.00	20.00	2.00	1.00	20.00	40.00	-10.30
3.00	0.00	2.00	38.00	4.00	1.00	38.00	76.00	-12.30
1.00	1.00	2.00	19.00	2.00	1.00	19.00	19.00	1.50
4.00	0.00	1.00	68.00	5.00	2.00	136.00	68.00	4.60
1.00	1.00	1.00	15.00	1.00	1.00	15.00	15.00	-2.90
1.00	1.00	1.00	21.00	2.00	1.00	21.00	21.00	-10.00
3.00	0.00	2.00	40.00	4.00	2.00	80.00	40.00	8.50
1.00	1.00	2.00	13.00	1.00	1.00	13.00	26.00	-1.90
2.00	0.00	1.00	32.00	3.00	2.00	64.00	64.00	6.50
1.00	1.00	2.00	16.00	1.00	1.00	16.00	16.00	0.50
3.00	0.00	2.00	38.00	4.00	1.00	38.00	76.00	3.80
1.00	1.00	2.00	21.00	2.00	1.00	21.00	21.00	-12.70
1.00	1.00	2.00	12.00	1.00	1.00	12.00	24.00	0.60
2.00	0.00	1.00	32.00	3.00	1.00	32.00	64.00	-1.10
1.00	1.00	2.00	15.00	1.00	1.00	15.00	30.00	-6.50
1.00	1.00	2.00	12.00	1.00	1.00	12.00	12.00	-6.00

4.00	0.00	2.00	61.00	5.00	2.00	122.00	122.00	4.50
1.00	1.00	1.00	15.00	1.00	1.00	15.00	15.00	0.70
2.00	0.00	2.00	26.00	3.00	2.00	52.00	26.00	11.50
2.00	0.00	1.00	32.00	3.00	1.00	32.00	64.00	-6.50
3.00	0.00	2.00	46.00	4.00	1.00	46.00	46.00	-15.50
1.00	1.00	1.00	15.00	1.00	1.00	15.00	15.00	7.50
1.00	1.00	2.00	19.00	2.00	1.00	19.00	19.00	-3.50
3.00	0.00	1.00	41.00	4.00	1.00	41.00	41.00	-1.00
1.00	1.00	1.00	15.00	1.00	1.00	15.00	15.00	-2.70
1.00	1.00	1.00	21.00	2.00	2.00	42.00	21.00	3.90
1.00	1.00	2.00	19.00	2.00	1.00	19.00	19.00	3.50
3.00	0.00	2.00	46.00	4.00	1.00	46.00	92.00	-16.50
2.00	0.00	2.00	29.00	3.00	2.00	58.00	29.00	15.90
1.00	1.00	2.00	14.00	1.00	1.00	14.00	14.00	7.50
3.00	0.00	2.00	49.00	4.00	2.00	98.00	98.00	5.50
1.00	1.00	1.00	14.00	1.00	1.00	14.00	28.00	-3.50
1.00	1.00	2.00	12.00	1.00	2.00	24.00	24.00	9.50
1.00	1.00	2.00	13.00	1.00	1.00	13.00	13.00	3.50
4.00	0.00	1.00	79.00	5.00	2.00	158.00	79.00	5.50
1.00	1.00	2.00	16.00	1.00	1.00	16.00	16.00	2.50
2.00	0.00	1.00	32.00	3.00	2.00	64.00	64.00	8.50
1.00	1.00	2.00	15.00	1.00	1.00	15.00	30.00	-13.80
1.00	1.00	2.00	16.00	1.00	1.00	16.00	32.00	-2.50
3.00	0.00	1.00	37.00	4.00	2.00	74.00	37.00	9.00
3.00	0.00	2.00	48.00	4.00	2.00	96.00	48.00	13.50
1.00	1.00	1.00	15.00	1.00	1.00	15.00	15.00	-2.50
4.00	0.00	2.00	70.00	5.00	1.00	70.00	70.00	8.00
1.00	1.00	1.00	15.00	1.00	1.00	15.00	15.00	-11.50
3.00	0.00	1.00	45.00	4.00	1.00	45.00	90.00	-2.50
1.00	1.00	2.00	13.00	1.00	1.00	13.00	26.00	-1.80
2.00	0.00	2.00	35.00	3.00	2.00	70.00	35.00	6.70
1.00	1.00	1.00	20.00	2.00	1.00	20.00	40.00	-6.50
3.00	0.00	2.00	36.00	4.00	2.00	72.00	72.00	2.70
3.00	0.00	1.00	54.00	4.00	2.00	108.00	108.00	10.30
1.00	1.00	1.00	16.00	1.00	1.00	16.00	16.00	5.00
1.00	1.00	1.00	19.00	2.00	1.00	19.00	19.00	-14.30
1.00	1.00	2.00	15.00	1.00	1.00	15.00	30.00	-3.00
1.00	1.00	2.00	19.00	2.00	2.00	38.00	19.00	7.80
1.00	1.00	1.00	15.00	1.00	1.00	15.00	30.00	-4.20
3.00	0.00	2.00	36.00	4.00	1.00	36.00	72.00	0.80
1.00	1.00	2.00	17.00	1.00	1.00	17.00	17.00	-8.30
2.00	0.00	1.00	27.00	3.00	2.00	54.00	27.00	5.70
1.00	1.00	2.00	13.00	1.00	1.00	13.00	13.00	7.00
1.00	1.00	2.00	18.00	2.00	1.00	18.00	18.00	-7.20
4.00	0.00	2.00	61.00	5.00	2.00	122.00	61.00	12.00
2.00	0.00	1.00	29.00	3.00	1.00	29.00	29.00	9.00
1.00	1.00	2.00	17.00	1.00	1.00	17.00	34.00	-11.40

2.00	0.00	1.00	25.00	3.00	2.00	50.00	50.00	4.10
1.00	1.00	2.00	16.00	1.00	1.00	16.00	16.00	-4.90
1.00	1.00	1.00	11.00	1.00	1.00	11.00	11.00	5.30
1.00	1.00	2.00	15.00	1.00	1.00	15.00	30.00	-0.30
3.00	0.00	2.00	46.00	4.00	1.00	46.00	46.00	-0.50
1.00	1.00	1.00	23.00	2.00	1.00	23.00	46.00	-2.40
1.00	1.00	2.00	10.00	1.00	1.00	10.00	20.00	2.90
3.00	0.00	1.00	40.00	4.00	1.00	40.00	40.00	1.30
3.00	0.00	2.00	46.00	4.00	1.00	46.00	46.00	-4.60
1.00	1.00	2.00	14.00	1.00	1.00	14.00	28.00	-1.10
3.00	0.00	1.00	54.00	4.00	2.00	108.00	108.00	-1.50
1.00	1.00	1.00	13.00	1.00	1.00	13.00	26.00	-3.70
3.00	0.00	2.00	41.00	4.00	2.00	82.00	41.00	0.60
1.00	1.00	1.00	12.00	1.00	1.00	12.00	24.00	8.30
2.00	0.00	2.00	28.00	3.00	1.00	28.00	56.00	-4.50
3.00	0.00	2.00	41.00	4.00	1.00	41.00	41.00	5.30
1.00	1.00	2.00	17.00	1.00	1.00	17.00	34.00	-3.50
3.00	0.00	2.00	36.00	4.00	1.00	36.00	36.00	-11.50
1.00	1.00	1.00	17.00	1.00	1.00	17.00	17.00	-4.00
2.00	0.00	2.00	35.00	3.00	2.00	70.00	70.00	17.60
3.00	0.00	2.00	59.00	4.00	2.00	118.00	59.00	9.10
1.00	1.00	2.00	17.00	1.00	1.00	17.00	17.00	5.10
3.00	0.00	1.00	48.00	4.00	2.00	96.00	96.00	16.80
1.00	1.00	2.00	20.00	2.00	1.00	20.00	20.00	-11.50
2.00	0.00	1.00	28.00	3.00	1.00	28.00	28.00	2.30
1.00	1.00	1.00	13.00	1.00	1.00	13.00	26.00	-3.40
1.00	1.00	1.00	19.00	2.00	1.00	19.00	38.00	-8.50
3.00	0.00	2.00	38.00	4.00	1.00	38.00	38.00	-9.50
1.00	1.00	2.00	11.00	1.00	1.00	11.00	22.00	-4.40
4.00	0.00	2.00	60.00	5.00	2.00	120.00	60.00	9.00
1.00	1.00	1.00	13.00	1.00	1.00	13.00	26.00	1.20
3.00	0.00	2.00	52.00	4.00	1.00	52.00	104.00	-11.10
1.00	1.00	2.00	15.00	1.00	1.00	15.00	15.00	-6.50
1.00	1.00	2.00	15.00	1.00	1.00	15.00	30.00	-6.50
4.00	0.00	2.00	64.00	5.00	2.00	128.00	64.00	10.80
1.00	1.00	2.00	19.00	2.00	1.00	19.00	38.00	-4.40
4.00	0.00	1.00	68.00	5.00	1.00	68.00	68.00	-11.30
4.00	0.00	1.00	92.00	5.00	1.00	92.00	184.00	1.50
1.00	1.00	1.00	18.00	2.00	1.00	18.00	36.00	-1.50
1.00	1.00	2.00	10.00	1.00	2.00	20.00	10.00	5.70
3.00	0.00	1.00	39.00	4.00	2.00	78.00	39.00	7.00
1.00	1.00	1.00	14.00	1.00	1.00	14.00	14.00	-9.00
1.00	1.00	1.00	13.00	1.00	1.00	13.00	13.00	1.10
2.00	0.00	1.00	35.00	3.00	2.00	70.00	35.00	15.50
1.00	1.00	1.00	11.00	1.00	2.00	22.00	22.00	-0.50
3.00	0.00	1.00	48.00	4.00	1.00	48.00	96.00	-2.50
3.00	0.00	1.00	54.00	4.00	1.00	54.00	54.00	-5.60

1.00	1.00	1.00	19.00	2.00	1.00	19.00	38.00	-7.70
1.00	1.00	1.00	18.00	2.00	1.00	18.00	18.00	-4.70
1.00	1.00	2.00	13.00	1.00	1.00	13.00	26.00	-4.30
4.00	0.00	2.00	76.00	5.00	2.00	152.00	76.00	9.00
1.00	1.00	2.00	16.00	1.00	1.00	16.00	32.00	-6.50
3.00	0.00	1.00	38.00	4.00	1.00	38.00	76.00	-7.70
3.00	0.00	1.00	41.00	4.00	1.00	41.00	82.00	1.70
1.00	1.00	1.00	13.00	1.00	1.00	13.00	13.00	2.10
3.00	0.00	2.00	36.00	4.00	2.00	72.00	72.00	1.20
1.00	1.00	2.00	11.00	1.00	2.00	22.00	22.00	6.10
4.00	0.00	2.00	69.00	5.00	2.00	138.00	69.00	6.00
1.00	1.00	1.00	13.00	1.00	1.00	13.00	26.00	1.50
3.00	0.00	1.00	51.00	4.00	1.00	51.00	102.00	-9.50
1.00	1.00	1.00	15.00	1.00	1.00	15.00	15.00	5.90
1.00	1.00	2.00	10.00	1.00	2.00	20.00	10.00	7.10
3.00	0.00	2.00	46.00	4.00	1.00	46.00	46.00	-7.50
1.00	1.00	1.00	15.00	1.00	1.00	15.00	30.00	0.50
3.00	0.00	2.00	51.00	4.00	2.00	102.00	51.00	10.40
1.00	1.00	2.00	12.00	1.00	1.00	12.00	24.00	2.10
2.00	0.00	2.00	35.00	3.00	2.00	70.00	70.00	8.00
1.00	1.00	2.00	18.00	2.00	1.00	18.00	36.00	-1.50
1.00	1.00	2.00	16.00	1.00	1.00	16.00	32.00	-3.20
1.00	1.00	1.00	21.00	2.00	2.00	42.00	21.00	6.90
3.00	0.00	2.00	52.00	4.00	1.00	52.00	52.00	-3.70
1.00	1.00	1.00	14.00	1.00	1.00	14.00	28.00	0.10
1.00	1.00	2.00	13.00	1.00	1.00	13.00	26.00	-0.40
2.00	0.00	1.00	28.00	3.00	1.00	28.00	28.00	-0.80
1.00	1.00	2.00	11.00	1.00	1.00	11.00	11.00	-2.30
4.00	0.00	2.00	75.00	5.00	1.00	75.00	75.00	-10.00
1.00	1.00	2.00	17.00	1.00	1.00	17.00	34.00	-3.30
2.00	0.00	2.00	30.00	3.00	2.00	60.00	30.00	16.10
1.00	1.00	1.00	9.00	1.00	2.00	18.00	18.00	-1.40
3.00	0.00	2.00	44.00	4.00	1.00	44.00	44.00	-10.50
2.00	0.00	1.00	34.00	3.00	2.00	68.00	68.00	5.70
1.00	1.00	1.00	17.00	1.00	1.00	17.00	34.00	5.40
1.00	1.00	2.00	18.00	2.00	1.00	18.00	18.00	1.20
3.00	0.00	2.00	41.00	4.00	2.00	82.00	82.00	12.20
1.00	1.00	2.00	14.00	1.00	1.00	14.00	14.00	-1.40
1.00	1.00	1.00	21.00	2.00	1.00	21.00	42.00	-6.50
3.00	0.00	2.00	48.00	4.00	2.00	96.00	48.00	13.80
2.00	0.00	2.00	24.00	3.00	2.00	48.00	48.00	14.50
1.00	1.00	1.00	17.00	1.00	1.00	17.00	17.00	-5.40
3.00	0.00	2.00	39.00	4.00	1.00	39.00	78.00	-3.30
4.00	0.00	2.00	66.00	5.00	1.00	66.00	66.00	-3.10
1.00	1.00	1.00	15.00	1.00	1.00	15.00	30.00	-1.50
3.00	0.00	2.00	36.00	4.00	2.00	72.00	36.00	9.80
2.00	0.00	1.00	24.00	3.00	2.00	48.00	48.00	9.20

1.00	1.00	1.00	18.00	2.00	1.00	18.00	36.00	-9.30
4.00	0.00	2.00	72.00	5.00	1.00	72.00	72.00	-4.50
1.00	1.00	2.00	20.00	2.00	1.00	20.00	20.00	-12.00
2.00	0.00	1.00	26.00	3.00	1.00	26.00	52.00	-5.10
3.00	0.00	1.00	38.00	4.00	2.00	76.00	38.00	12.20
1.00	1.00	1.00	14.00	1.00	1.00	14.00	28.00	3.00
1.00	1.00	2.00	17.00	1.00	1.00	17.00	17.00	1.50
3.00	0.00	1.00	43.00	4.00	1.00	43.00	43.00	-6.50
1.00	1.00	1.00	12.00	1.00	2.00	24.00	24.00	2.50
3.00	0.00	1.00	41.00	4.00	1.00	41.00	41.00	-15.50
1.00	1.00	1.00	13.00	1.00	1.00	13.00	26.00	-1.50
3.00	0.00	1.00	53.00	4.00	2.00	106.00	106.00	8.20
1.00	1.00	1.00	16.00	1.00	1.00	16.00	32.00	0.50
2.00	0.00	1.00	33.00	3.00	2.00	66.00	66.00	12.50
1.00	1.00	2.00	12.00	1.00	2.00	24.00	12.00	-1.50
4.00	0.00	2.00	76.00	5.00	2.00	152.00	76.00	17.50
4.00	0.00	2.00	65.00	5.00	1.00	65.00	65.00	2.50
1.00	1.00	1.00	21.00	2.00	1.00	21.00	42.00	-7.50
3.00	0.00	2.00	44.00	4.00	1.00	44.00	44.00	-12.10
3.00	0.00	1.00	38.00	4.00	1.00	38.00	76.00	-1.50
1.00	1.00	1.00	9.00	1.00	1.00	9.00	9.00	5.00
4.00	0.00	2.00	86.00	5.00	2.00	172.00	86.00	5.50
2.00	0.00	1.00	29.00	3.00	1.00	29.00	29.00	1.80
1.00	1.00	2.00	16.00	1.00	1.00	16.00	16.00	-7.20
1.00	1.00	2.00	22.00	2.00	1.00	22.00	22.00	-11.40
2.00	0.00	2.00	26.00	3.00	1.00	26.00	52.00	-7.40
1.00	1.00	1.00	11.00	1.00	1.00	11.00	11.00	2.60
3.00	0.00	2.00	51.00	4.00	1.00	51.00	102.00	1.30
2.00	0.00	2.00	30.00	3.00	1.00	30.00	30.00	-3.50
1.00	1.00	2.00	19.00	2.00	2.00	38.00	38.00	9.60
1.00	1.00	2.00	21.00	2.00	1.00	21.00	42.00	-14.50
1.00	1.00	1.00	19.00	2.00	1.00	19.00	38.00	0.50
2.00	0.00	1.00	26.00	3.00	1.00	26.00	52.00	-3.20
3.00	0.00	2.00	44.00	4.00	2.00	88.00	88.00	7.60
1.00	1.00	2.00	14.00	1.00	1.00	14.00	14.00	-6.90
3.00	0.00	2.00	46.00	4.00	1.00	46.00	46.00	0.90
1.00	1.00	2.00	8.00	1.00	2.00	16.00	8.00	7.60
1.00	1.00	1.00	13.00	1.00	1.00	13.00	13.00	8.60
1.00	1.00	2.00	17.00	1.00	1.00	17.00	17.00	-5.60
3.00	0.00	1.00	44.00	4.00	2.00	88.00	88.00	7.50
3.00	0.00	2.00	52.00	4.00	1.00	52.00	104.00	-0.50
1.00	1.00	1.00	15.00	1.00	1.00	15.00	15.00	-6.50
3.00	0.00	2.00	45.00	4.00	1.00	45.00	45.00	-1.50
3.00	0.00	2.00	51.00	4.00	1.00	51.00	51.00	2.00
2.00	0.00	2.00	35.00	3.00	1.00	35.00	70.00	-0.50
1.00	1.00	2.00	20.00	2.00	1.00	20.00	20.00	-11.50
1.00	1.00	2.00	12.00	1.00	1.00	12.00	24.00	5.50

3.00	0.00	1.00	50.00	4.00	2.00	100.00	50.00	4.50
1.00	1.00	2.00	15.00	1.00	1.00	15.00	30.00	-4.50
2.00	0.00	2.00	32.00	3.00	1.00	32.00	64.00	-6.10
3.00	0.00	1.00	36.00	4.00	1.00	36.00	72.00	-5.50
1.00	1.00	1.00	11.00	1.00	2.00	22.00	22.00	3.50
3.00	0.00	1.00	48.00	4.00	2.00	96.00	48.00	8.50
1.00	1.00	1.00	22.00	2.00	2.00	44.00	22.00	3.40
1.00	1.00	1.00	21.00	2.00	1.00	21.00	21.00	-7.50
3.00	0.00	1.00	36.00	4.00	2.00	72.00	72.00	5.00
1.00	1.00	1.00	15.00	1.00	1.00	15.00	15.00	-3.30
1.00	1.00	2.00	14.00	1.00	1.00	14.00	14.00	6.50
3.00	0.00	2.00	45.00	4.00	1.00	45.00	90.00	-3.50
1.00	1.00	1.00	12.00	1.00	1.00	12.00	24.00	0.50
1.00	1.00	2.00	12.00	1.00	1.00	12.00	24.00	1.50
1.00	1.00	2.00	9.00	1.00	2.00	18.00	18.00	3.60
1.00	1.00	1.00	23.00	2.00	2.00	46.00	23.00	8.50
4.00	0.00	2.00	67.00	5.00	1.00	67.00	67.00	-3.50
1.00	1.00	2.00	16.00	1.00	1.00	16.00	16.00	-15.50
4.00	0.00	2.00	81.00	5.00	1.00	81.00	81.00	-0.60
2.00	0.00	2.00	33.00	3.00	2.00	66.00	66.00	2.70
1.00	1.00	1.00	12.00	1.00	1.00	12.00	12.00	8.50
2.00	0.00	1.00	25.00	3.00	2.00	50.00	25.00	14.50
1.00	1.00	1.00	13.00	1.00	2.00	26.00	13.00	4.50
1.00	1.00	2.00	21.00	2.00	1.00	21.00	21.00	-14.50
1.00	1.00	1.00	12.00	1.00	1.00	12.00	12.00	0.00
3.00	0.00	2.00	47.00	4.00	1.00	47.00	94.00	-1.50
3.00	0.00	2.00	46.00	4.00	1.00	46.00	46.00	-11.20
1.00	1.00	2.00	18.00	2.00	1.00	18.00	36.00	-13.50
3.00	0.00	1.00	52.00	4.00	1.00	52.00	52.00	-9.50
1.00	1.00	1.00	13.00	1.00	1.00	13.00	26.00	-3.50
1.00	1.00	1.00	11.00	1.00	2.00	22.00	22.00	6.60
1.00	1.00	1.00	13.00	1.00	1.00	13.00	13.00	-1.50
1.00	1.00	2.00	21.00	2.00	2.00	42.00	42.00	-6.30
2.00	0.00	2.00	26.00	3.00	1.00	26.00	52.00	-11.50
3.00	0.00	2.00	52.00	4.00	1.00	52.00	104.00	-2.30
1.00	1.00	2.00	10.00	1.00	2.00	20.00	20.00	7.50
1.00	1.00	1.00	14.00	1.00	1.00	14.00	14.00	-4.50
1.00	1.00	1.00	19.00	2.00	1.00	19.00	19.00	-2.50
1.00	1.00	2.00	19.00	2.00	1.00	19.00	19.00	-5.50
1.00	1.00	1.00	16.00	1.00	1.00	16.00	32.00	-7.50
3.00	0.00	2.00	49.00	4.00	2.00	98.00	49.00	14.70
1.00	1.00	1.00	16.00	1.00	1.00	16.00	32.00	-3.30
4.00	0.00	1.00	85.00	5.00	1.00	85.00	85.00	4.50
3.00	0.00	1.00	58.00	4.00	1.00	58.00	116.00	1.20
1.00	1.00	1.00	20.00	2.00	1.00	20.00	20.00	-11.10
1.00	1.00	1.00	14.00	1.00	1.00	14.00	28.00	6.20
1.00	1.00	1.00	13.00	1.00	1.00	13.00	26.00	-0.30

3.00	0.00	1.00	45.00	4.00	2.00	90.00	90.00	6.50
2.00	0.00	1.00	24.00	3.00	2.00	48.00	48.00	-9.50
1.00	1.00	1.00	16.00	1.00	1.00	16.00	16.00	2.00
2.00	0.00	2.00	31.00	3.00	2.00	62.00	62.00	4.50
1.00	1.00	1.00	22.00	2.00	1.00	22.00	22.00	-11.00
4.00	0.00	2.00	73.00	5.00	2.00	146.00	146.00	16.50
1.00	1.00	2.00	14.00	1.00	1.00	14.00	28.00	-2.50
3.00	0.00	2.00	36.00	4.00	2.00	72.00	36.00	7.50
1.00	1.00	2.00	12.00	1.00	1.00	12.00	24.00	-4.50
3.00	0.00	2.00	44.00	4.00	1.00	44.00	44.00	-10.50
1.00	1.00	2.00	14.00	1.00	1.00	14.00	14.00	1.90
3.00	0.00	2.00	45.00	4.00	1.00	45.00	90.00	-14.00
1.00	1.00	2.00	16.00	1.00	1.00	16.00	16.00	6.50
2.00	0.00	2.00	24.00	3.00	1.00	24.00	48.00	0.50
1.00	1.00	2.00	22.00	2.00	1.00	22.00	22.00	-9.20
2.00	0.00	1.00	24.00	3.00	2.00	48.00	24.00	3.60
1.00	1.00	2.00	15.00	1.00	1.00	15.00	15.00	5.00
1.00	1.00	1.00	15.00	1.00	1.00	15.00	15.00	-0.20
1.00	1.00	2.00	16.00	1.00	1.00	16.00	32.00	-1.50
2.00	0.00	1.00	29.00	3.00	1.00	29.00	29.00	-14.00
1.00	1.00	2.00	14.00	1.00	1.00	14.00	14.00	-4.50
2.00	0.00	2.00	35.00	3.00	2.00	70.00	70.00	6.70
1.00	1.00	1.00	11.00	1.00	1.00	11.00	22.00	-9.00
1.00	1.00	1.00	21.00	2.00	1.00	21.00	42.00	-6.00
2.00	0.00	2.00	35.00	3.00	2.00	70.00	35.00	10.00
1.00	1.00	1.00	18.00	2.00	1.00	18.00	36.00	-3.20
1.00	1.00	2.00	12.00	1.00	1.00	12.00	24.00	5.80
1.00	1.00	1.00	22.00	2.00	1.00	22.00	22.00	-9.40
3.00	0.00	1.00	47.00	4.00	1.00	47.00	47.00	-6.00
3.00	0.00	2.00	43.00	4.00	2.00	86.00	43.00	10.00
1.00	1.00	1.00	16.00	1.00	1.00	16.00	16.00	6.10
1.00	1.00	1.00	9.00	1.00	2.00	18.00	9.00	5.20
3.00	0.00	1.00	46.00	4.00	1.00	46.00	46.00	-11.30
2.00	0.00	2.00	27.00	3.00	2.00	54.00	27.00	7.00
1.00	1.00	2.00	18.00	2.00	1.00	18.00	18.00	-3.10
1.00	1.00	1.00	19.00	2.00	1.00	19.00	38.00	-10.50
3.00	0.00	1.00	36.00	4.00	2.00	72.00	36.00	6.90
1.00	1.00	1.00	17.00	1.00	1.00	17.00	34.00	-9.00
4.00	0.00	2.00	75.00	5.00	1.00	75.00	75.00	-10.00
1.00	1.00	1.00	21.00	2.00	1.00	21.00	21.00	-15.50
4.00	0.00	2.00	68.00	5.00	2.00	136.00	136.00	17.50
1.00	1.00	2.00	11.00	1.00	1.00	11.00	22.00	-6.70
1.00	1.00	1.00	14.00	1.00	1.00	14.00	14.00	1.80
3.00	0.00	2.00	44.00	4.00	2.00	88.00	88.00	3.00
3.00	0.00	1.00	51.00	4.00	1.00	51.00	102.00	-7.00
1.00	1.00	2.00	9.00	1.00	2.00	18.00	18.00	-2.00
3.00	0.00	2.00	47.00	4.00	1.00	47.00	94.00	-4.20

1.00	1.00	1.00	10.00	1.00	1.00	10.00	10.00	7.50
2.00	0.00	1.00	25.00	3.00	2.00	50.00	50.00	4.90
1.00	1.00	2.00	10.00	1.00	1.00	10.00	20.00	13.50
2.00	0.00	2.00	29.00	3.00	1.00	29.00	29.00	0.70
3.00	0.00	1.00	38.00	4.00	2.00	76.00	76.00	3.50
1.00	1.00	2.00	20.00	2.00	1.00	20.00	40.00	-10.50
1.00	1.00	2.00	10.00	1.00	1.00	10.00	20.00	-0.50
4.00	0.00	2.00	70.00	5.00	2.00	140.00	140.00	3.00
1.00	1.00	1.00	19.00	2.00	1.00	19.00	19.00	-2.00
2.00	0.00	2.00	32.00	3.00	1.00	32.00	32.00	-1.10
2.00	0.00	2.00	28.00	3.00	2.00	56.00	28.00	5.60
2.00	0.00	2.00	26.00	3.00	1.00	26.00	52.00	-0.50
1.00	1.00	2.00	11.00	1.00	1.00	11.00	22.00	4.50
1.00	1.00	2.00	13.00	1.00	1.00	13.00	13.00	11.50
3.00	0.00	2.00	36.00	4.00	1.00	36.00	36.00	-4.50
3.00	0.00	1.00	43.00	4.00	1.00	43.00	43.00	-4.50
4.00	0.00	2.00	61.00	5.00	1.00	61.00	122.00	-5.50
1.00	1.00	2.00	9.00	1.00	1.00	9.00	9.00	1.50
1.00	1.00	1.00	19.00	2.00	1.00	19.00	38.00	-8.30
4.00	0.00	1.00	70.00	5.00	1.00	70.00	70.00	-10.50
1.00	1.00	1.00	15.00	1.00	1.00	15.00	15.00	4.40
3.00	0.00	2.00	45.00	4.00	2.00	90.00	90.00	11.10
2.00	0.00	2.00	30.00	3.00	2.00	60.00	60.00	6.20
1.00	1.00	1.00	22.00	2.00	1.00	22.00	44.00	-10.40
2.00	0.00	2.00	31.00	3.00	1.00	31.00	31.00	-16.50
1.00	1.00	2.00	18.00	2.00	1.00	18.00	36.00	-3.00
1.00	1.00	1.00	12.00	1.00	1.00	12.00	24.00	-2.90
4.00	0.00	2.00	61.00	5.00	1.00	61.00	122.00	-11.20
4.00	0.00	1.00	62.00	5.00	2.00	124.00	62.00	12.70
1.00	1.00	2.00	20.00	2.00	1.00	20.00	40.00	-12.00
1.00	1.00	1.00	17.00	1.00	1.00	17.00	17.00	10.60
3.00	0.00	1.00	55.00	4.00	2.00	110.00	55.00	14.50
3.00	0.00	2.00	49.00	4.00	2.00	98.00	49.00	12.50
1.00	1.00	2.00	13.00	1.00	1.00	13.00	26.00	3.40
3.00	0.00	2.00	36.00	4.00	2.00	72.00	36.00	16.50
3.00	0.00	2.00	41.00	4.00	2.00	82.00	41.00	6.60
1.00	1.00	2.00	15.00	1.00	1.00	15.00	30.00	-2.20
3.00	0.00	1.00	53.00	4.00	2.00	106.00	53.00	0.00
1.00	1.00	2.00	20.00	2.00	1.00	20.00	20.00	-7.30
4.00	0.00	2.00	71.00	5.00	2.00	142.00	142.00	3.00
1.00	1.00	2.00	13.00	1.00	1.00	13.00	26.00	5.00
1.00	1.00	2.00	14.00	1.00	1.00	14.00	28.00	-1.00
4.00	0.00	2.00	61.00	5.00	2.00	122.00	122.00	19.50
1.00	1.00	1.00	20.00	2.00	1.00	20.00	40.00	-2.50
4.00	0.00	2.00	70.00	5.00	1.00	70.00	70.00	-11.30
1.00	1.00	2.00	14.00	1.00	1.00	14.00	14.00	-2.50
3.00	0.00	2.00	50.00	4.00	2.00	100.00	50.00	19.50

3.00	0.00	2.00	59.00	4.00	2.00	118.00	118.00	10.50
1.00	1.00	2.00	15.00	1.00	1.00	15.00	15.00	6.50
1.00	1.00	2.00	14.00	1.00	1.00	14.00	28.00	-3.50
1.00	1.00	2.00	18.00	2.00	1.00	18.00	18.00	-2.00
4.00	0.00	1.00	72.00	5.00	2.00	144.00	144.00	24.10
1.00	1.00	2.00	12.00	1.00	1.00	12.00	12.00	-0.10
4.00	0.00	2.00	68.00	5.00	2.00	136.00	68.00	8.50
1.00	1.00	2.00	13.00	1.00	1.00	13.00	26.00	1.50
3.00	0.00	2.00	54.00	4.00	2.00	108.00	108.00	8.50
1.00	1.00	2.00	13.00	1.00	1.00	13.00	26.00	8.50
3.00	0.00	2.00	48.00	4.00	2.00	96.00	96.00	8.50
1.00	1.00	2.00	18.00	2.00	1.00	18.00	36.00	-2.00
1.00	1.00	2.00	13.00	1.00	1.00	13.00	26.00	-1.00
4.00	0.00	2.00	75.00	5.00	1.00	75.00	75.00	-2.20
1.00	1.00	2.00	21.00	2.00	1.00	21.00	21.00	-6.60
3.00	0.00	2.00	38.00	4.00	2.00	76.00	76.00	12.00
1.00	1.00	2.00	10.00	1.00	1.00	10.00	10.00	6.70
2.00	0.00	2.00	35.00	3.00	1.00	35.00	35.00	-16.90
3.00	0.00	2.00	40.00	4.00	1.00	40.00	80.00	-2.30
1.00	1.00	2.00	9.00	1.00	1.00	9.00	18.00	7.00
1.00	1.00	2.00	19.00	2.00	1.00	19.00	19.00	-4.60
3.00	0.00	2.00	48.00	4.00	2.00	96.00	48.00	13.20
4.00	0.00	2.00	65.00	5.00	2.00	130.00	65.00	11.00
1.00	1.00	1.00	15.00	1.00	1.00	15.00	30.00	-4.00
3.00	0.00	2.00	41.00	4.00	2.00	82.00	82.00	11.00
1.00	1.00	1.00	10.00	1.00	2.00	20.00	20.00	-1.50
2.00	0.00	2.00	28.00	3.00	2.00	56.00	56.00	0.60
1.00	1.00	2.00	10.00	1.00	1.00	10.00	20.00	2.60
1.00	1.00	2.00	17.00	1.00	1.00	17.00	17.00	-1.20
1.00	1.00	2.00	23.00	2.00	2.00	46.00	23.00	19.30
2.00	0.00	2.00	31.00	3.00	2.00	62.00	62.00	2.50
1.00	1.00	2.00	14.00	1.00	1.00	14.00	14.00	-0.90
3.00	0.00	1.00	43.00	4.00	2.00	86.00	43.00	12.80
1.00	1.00	2.00	11.00	1.00	1.00	11.00	22.00	3.00
1.00	1.00	1.00	20.00	2.00	1.00	20.00	40.00	-2.70
3.00	0.00	2.00	44.00	4.00	1.00	44.00	88.00	-9.40
3.00	0.00	2.00	40.00	4.00	1.00	40.00	80.00	-11.70
1.00	1.00	1.00	11.00	1.00	2.00	22.00	22.00	8.50
3.00	0.00	2.00	54.00	4.00	2.00	108.00	54.00	5.60
1.00	1.00	1.00	18.00	2.00	1.00	18.00	36.00	-11.90
3.00	0.00	2.00	47.00	4.00	1.00	47.00	47.00	-12.30
1.00	1.00	1.00	18.00	2.00	1.00	18.00	36.00	-6.60
1.00	1.00	2.00	16.00	1.00	1.00	16.00	16.00	-8.40
1.00	1.00	1.00	13.00	1.00	1.00	13.00	26.00	2.00
1.00	1.00	2.00	20.00	2.00	1.00	20.00	40.00	-16.50
1.00	1.00	2.00	19.00	2.00	1.00	19.00	38.00	-9.50
3.00	0.00	2.00	54.00	4.00	1.00	54.00	108.00	-3.00

1.00	1.00	1.00	18.00	2.00	1.00	18.00	18.00	-3.50
2.00	0.00	1.00	32.00	3.00	1.00	32.00	32.00	-17.00
1.00	1.00	1.00	17.00	1.00	1.00	17.00	34.00	-11.00
3.00	0.00	1.00	50.00	4.00	1.00	50.00	100.00	-16.90
1.00	1.00	2.00	12.00	1.00	2.00	24.00	12.00	2.60
3.00	0.00	2.00	36.00	4.00	2.00	72.00	72.00	9.80
1.00	1.00	2.00	10.00	1.00	1.00	10.00	10.00	-0.30
3.00	0.00	2.00	46.00	4.00	1.00	46.00	46.00	-2.50
1.00	1.00	2.00	17.00	1.00	1.00	17.00	34.00	-1.00
3.00	0.00	1.00	54.00	4.00	1.00	54.00	54.00	-3.00
4.00	0.00	2.00	61.00	5.00	1.00	61.00	61.00	-11.20
1.00	1.00	2.00	20.00	2.00	1.00	20.00	20.00	-12.00
1.00	1.00	2.00	21.00	2.00	1.00	21.00	21.00	-2.30
3.00	0.00	2.00	46.00	4.00	1.00	46.00	46.00	-3.00

Zscore2	Birth	Postnatal3	Preceding	Intervalrev	ageindex	ageprecedi	Intervalrev	Preceding2
2.12	3500.00	447.62	38.00	47.00	21	68	1.00	1.00
0.09	2900.00	563.64	22.00	31.00	11	42	1.00	0.00
1.92	3600.00	523.08	20.00	29.00	13	42	1.00	0.00
-0.15	2900.00	575.00	23.00	32.00	8	40	1.00	0.00
-1.53	3200.00	1000.00	52.00	61.00	2	63	1.00	1.00
-0.19	3000.00	983.33	37.00	46.00	6	52	1.00	1.00
0.95	3500.00	450.00	22.00	13.00	12	25	0.00	0.00
0.32	3300.00	376.92	22.00	13.00	13	25	0.00	0.00
-0.77	3500.00	975.00	63.00	72.00	4	76	1.00	1.00
-0.45	3400.00	542.86	25.00	34.00	7	41	1.00	1.00
-1.28	2400.00	1175.00	14.00	23.00	4	27	0.00	0.00
-0.42	3600.00	1175.00	29.00	38.00	4	42	1.00	1.00
-0.94	2600.00	960.00	10.00	19.00	5	24	0.00	0.00
-0.46	2500.00	550.00	12.00	21.00	8	29	0.00	0.00
-2.13	2100.00	666.67	86.00	95.00	3	98	1.00	1.00
-0.95	2500.00	740.00	14.00	23.00	5	28	0.00	0.00
-0.88	2600.00	933.33	28.00	37.00	6	43	1.00	1.00
-1.12	4000.00	236.36	26.00	35.00	11	46	1.00	1.00
-1.58	2700.00	1333.33	12.00	21.00	3	24	0.00	0.00
-1.32	3000.00	1233.33	75.00	84.00	3	87	1.00	1.00
-0.71	2700.00	920.00	67.00	76.00	5	81	1.00	1.00
-0.71	2800.00	587.50	10.00	19.00	8	27	0.00	0.00
0.23	2800.00	862.50	55.00	64.00	8	72	1.00	1.00
1.56	2900.00	500.00	23.00	32.00	12	44	1.00	0.00
0.45	2500.00	357.89	44.00	53.00	19	72	1.00	1.00
-1.19	3000.00	614.29	71.00	80.00	7	87	1.00	1.00
-0.79	3300.00	1000.00	35.00	44.00	4	48	1.00	1.00
0.63	3200.00	644.44	26.00	35.00	9	44	1.00	1.00
1.08	3500.00	538.46	74.00	83.00	13	96	1.00	1.00
0.03	3100.00	825.00	25.00	34.00	8	42	1.00	1.00
-0.27	2900.00	600.00	23.00	32.00	8	40	1.00	0.00
-0.46	2700.00	785.71	16.00	25.00	7	32	1.00	0.00
0.42	2700.00	609.09	52.00	61.00	11	72	1.00	1.00
-0.78	2400.00	509.09	19.00	28.00	11	39	1.00	0.00
-0.41	2900.00	581.82	22.00	31.00	11	42	1.00	0.00
-0.14	2800.00	1066.67	23.00	32.00	6	38	1.00	0.00
0.83	2600.00	545.00	16.00	25.00	20	45	1.00	0.00
0.83	2500.00	466.67	12.00	21.00	18	39	0.00	0.00
0.19	3000.00	409.09	22.00	31.00	11	42	1.00	0.00
0.58	2600.00	540.00	12.00	21.00	10	31	0.00	0.00
1.99	2900.00	379.17	38.00	47.00	24	71	1.00	1.00
0.35	2300.00	600.00	21.00	30.00	12	42	1.00	0.00
0.45	1700.00	633.33	21.00	30.00	12	42	1.00	0.00
0.56	3200.00	291.30	40.00	49.00	23	72	1.00	1.00
1.64	2800.00	383.33	31.00	40.00	24	64	1.00	1.00
1.64	3100.00	684.62	26.00	35.00	13	48	1.00	1.00

0.53	2800.00	429.17	12.00	21.00	24	45	0.00	0.00
-0.42	3400.00	571.43	24.00	15.00	7	22	0.00	1.00
-0.28	2600.00	580.00	42.00	51.00	5	56	1.00	1.00
-0.28	2900.00	666.67	57.00	66.00	6	72	1.00	1.00
-0.65	2800.00	566.67	21.00	30.00	6	36	1.00	0.00
-0.74	3000.00	1266.67	51.00	60.00	3	63	1.00	1.00
-0.51	2600.00	400.00	21.00	30.00	11	41	1.00	0.00
1.22	2700.00	384.21	21.00	30.00	19	49	1.00	0.00
1.86	3150.00	285.42	39.00	48.00	24	72	1.00	1.00
-0.32	3400.00	1220.00	10.00	19.00	5	24	0.00	0.00
-0.32	2700.00	680.00	21.00	30.00	10	40	1.00	0.00
-0.08	3900.00	328.57	21.00	30.00	14	44	1.00	0.00
-1.47	2600.00	950.00	37.00	46.00	2	48	1.00	1.00
0.96	3100.00	360.00	20.00	29.00	20	49	1.00	0.00
-0.83	2300.00	1050.00	21.00	30.00	4	34	1.00	0.00
0.58	2800.00	383.33	20.00	29.00	24	53	1.00	0.00
-1.53	2900.00	1300.00	9.00	18.00	2	20	0.00	0.00
-0.45	2700.00	484.62	14.00	23.00	13	36	0.00	0.00
1.51	2800.00	342.86	36.00	45.00	21	66	1.00	1.00
-0.17	2400.00	683.33	14.00	23.00	6	29	0.00	0.00
-0.45	3700.00	460.00	21.00	30.00	5	35	1.00	0.00
2.06	2700.00	359.09	21.00	30.00	22	52	1.00	0.00
1.42	2500.00	770.00	22.00	31.00	10	41	1.00	0.00
-0.08	3000.00	720.00	26.00	35.00	5	40	1.00	1.00
1.26	3200.00	483.33	21.00	30.00	12	42	1.00	0.00
-0.45	3100.00	614.29	22.00	31.00	7	38	1.00	0.00
-0.21	3300.00	462.50	18.00	27.00	8	35	1.00	0.00
0.36	2600.00	436.36	11.00	20.00	11	31	0.00	0.00
-0.71	3000.00	1025.00	11.00	20.00	4	24	0.00	0.00
1.22	2600.00	540.00	24.00	33.00	15	48	1.00	1.00
0.58	3000.00	1183.33	22.00	31.00	6	37	1.00	0.00
0.71	3300.00	294.44	9.00	18.00	18	36	0.00	0.00
-0.55	2700.00	1100.00	9.00	18.00	3	21	0.00	0.00
0.32	3100.00	518.18	48.00	57.00	11	68	1.00	1.00
0.83	2700.00	400.00	46.00	55.00	17	72	1.00	1.00
0.19	2800.00	500.00	21.00	30.00	11	41	1.00	0.00
0.45	3000.00	630.00	22.00	31.00	10	41	1.00	0.00
-0.32	2900.00	366.67	37.00	46.00	15	61	1.00	1.00
-0.10	2400.00	800.00	22.00	31.00	7	38	1.00	0.00
0.83	3100.00	412.50	16.00	25.00	24	49	1.00	0.00
-0.19	3200.00	252.63	27.00	36.00	19	55	1.00	1.00
-0.18	2400.00	350.00	21.00	30.00	14	44	1.00	0.00
-0.55	3400.00	860.00	24.00	33.00	5	38	1.00	1.00
1.10	2900.00	425.00	21.00	30.00	12	42	1.00	0.00
0.08	2600.00	509.09	52.00	61.00	11	72	1.00	1.00
-1.01	3100.00	666.67	12.00	21.00	3	24	0.00	0.00
-0.17	2900.00	700.00	20.00	29.00	5	34	1.00	0.00

1.26	2900.00	265.22	25.00	34.00	23	57	1.00	1.00
0.35	3600.00	550.00	22.00	31.00	8	39	1.00	0.00
-0.32	2900.00	600.00	34.00	43.00	5	48	1.00	1.00
-0.76	2500.00	1400.00	22.00	31.00	3	34	1.00	0.00
-0.24	2500.00	364.71	22.00	31.00	17	48	1.00	0.00
-0.95	2800.00	533.33	22.00	31.00	6	37	1.00	0.00
0.19	2800.00	458.33	39.00	48.00	12	60	1.00	1.00
0.13	2600.00	1425.00	22.00	31.00	4	35	1.00	0.00
-0.68	3300.00	420.00	48.00	57.00	10	67	1.00	1.00
0.95	2600.00	466.67	22.00	31.00	12	43	1.00	0.00
-0.03	3000.00	533.33	30.00	39.00	9	48	1.00	1.00
-0.04	3000.00	1600.00	23.00	32.00	3	35	1.00	0.00
1.79	3300.00	331.58	44.00	53.00	19	72	1.00	1.00
0.19	3000.00	675.00	29.00	38.00	8	46	1.00	1.00
2.41	3100.00	454.17	39.00	48.00	24	72	1.00	1.00
-0.67	2900.00	1000.00	21.00	30.00	4	34	1.00	0.00
-1.47	2900.00	650.00	11.00	20.00	4	24	0.00	0.00
-0.45	3300.00	875.00	18.00	27.00	4	31	1.00	0.00
1.24	2700.00	393.75	35.00	44.00	16	60	1.00	1.00
-2.00	2700.00	866.67	12.00	31.00	3	34	1.00	0.00
0.68	1900.00	475.00	18.00	27.00	20	47	1.00	0.00
-0.15	2600.00	279.17	29.00	38.00	24	62	1.00	1.00
-1.04	3200.00	666.67	10.00	19.00	3	22	0.00	0.00
2.18	2900.00	400.00	27.00	36.00	23	59	1.00	1.00
1.74	2600.00	321.74	33.00	42.00	23	65	1.00	1.00
-0.78	3100.00	660.00	10.00	19.00	5	24	0.00	0.00
-0.69	2300.00	760.00	20.00	29.00	5	34	1.00	0.00
0.78	3700.00	545.45	16.00	25.00	11	36	1.00	0.00
-1.37	3300.00	700.00	9.00	18.00	4	22	0.00	0.00
0.35	2500.00	368.42	32.00	41.00	19	60	1.00	1.00
-0.19	2600.00	914.29	14.00	23.00	7	30	0.00	0.00
-1.32	3000.00	1000.00	11.00	20.00	3	23	0.00	0.00
-1.58	3000.00	900.00	29.00	38.00	2	40	1.00	1.00
0.19	3300.00	900.00	10.00	19.00	5	24	0.00	0.00
0.59	3300.00	357.89	59.00	68.00	19	87	1.00	1.00
-0.37	3000.00	633.33	12.00	21.00	6	27	0.00	0.00
-1.28	3200.00	1400.00	12.00	21.00	2	23	0.00	0.00
1.09	2400.00	411.76	31.00	40.00	17	57	1.00	1.00
-0.24	3000.00	487.50	21.00	20.00	8	28	0.00	0.00
0.83	2700.00	331.25	23.00	32.00	16	48	1.00	0.00
0.06	3200.00	666.67	14.00	23.00	6	29	0.00	0.00
0.49	3000.00	700.00	29.00	38.00	10	48	1.00	1.00
-1.63	3400.00	1500.00	12.00	21.00	3	24	0.00	0.00
0.08	2900.00	418.18	21.00	30.00	11	41	1.00	0.00
-0.14	2800.00	600.00	23.00	32.00	7	39	1.00	0.00
-0.83	2600.00	475.00	12.00	21.00	8	29	0.00	0.00
-0.77	2700.00	450.00	21.00	30.00	6	36	1.00	0.00

0.58	3200.00	381.82	52.00	61.00	22	83	1.00	1.00
0.09	2400.00	833.33	12.00	21.00	6	27	0.00	0.00
1.47	3300.00	368.42	17.00	26.00	19	45	1.00	0.00
-0.83	3000.00	750.00	23.00	32.00	4	36	1.00	0.00
-1.99	2900.00	1150.00	37.00	46.00	2	48	1.00	1.00
0.96	3000.00	675.00	12.00	21.00	8	29	0.00	0.00
-0.45	3500.00	700.00	10.00	19.00	5	24	0.00	0.00
-0.13	3000.00	814.29	32.00	41.00	7	48	1.00	1.00
-0.35	2900.00	677.78	10.00	19.00	9	28	0.00	0.00
0.50	2600.00	435.71	12.00	21.00	14	132	0.00	0.00
0.45	3000.00	1000.00	10.00	19.00	5	24	0.00	0.00
-2.12	3400.00	600.00	37.00	46.00	2	48	1.00	1.00
2.04	3300.00	380.00	20.00	29.00	20	49	1.00	0.00
0.96	3600.00	640.00	10.00	19.00	10	29	0.00	0.00
0.71	2800.00	382.35	40.00	49.00	17	66	1.00	1.00
-0.45	3000.00	666.67	10.00	19.00	9	28	0.00	0.00
1.22	2600.00	262.50	21.00	30.00	24	54	1.00	0.00
0.45	3000.00	733.33	10.00	19.00	9	28	0.00	0.00
0.71	2600.00	335.29	60.00	79.00	17	96	1.00	1.00
0.32	4000.00	714.29	12.00	21.00	7	28	0.00	0.00
1.09	3000.00	368.75	23.00	32.00	16	48	1.00	0.00
-1.77	2700.00	1275.00	12.00	21.00	4	25	0.00	0.00
-0.32	3400.00	428.57	14.00	23.00	7	30	0.00	0.00
1.15	2800.00	273.91	28.00	37.00	23	60	1.00	1.00
1.73	3500.00	270.83	39.00	48.00	24	72	1.00	1.00
-0.32	3400.00	422.22	12.00	21.00	9	30	0.00	0.00
1.03	2800.00	1063.64	61.00	70.00	11	81	1.00	1.00
-1.47	2900.00	700.00	11.00	20.00	8	28	0.00	0.00
-0.32	3000.00	766.67	36.00	45.00	6	51	1.00	1.00
-0.23	2900.00	714.29	10.00	19.00	7	26	0.00	0.00
0.86	4200.00	315.79	26.00	35.00	19	54	1.00	1.00
-0.83	2500.00	875.00	11.00	20.00	4	24	0.00	0.00
0.35	2500.00	453.85	27.00	36.00	13	49	1.00	1.00
1.32	2900.00	330.43	45.00	54.00	23	77	1.00	1.00
0.64	3200.00	685.71	10.00	19.00	7	26	0.00	0.00
-1.83	3000.00	1000.00	10.00	19.00	2	21	0.00	0.00
-0.38	2800.00	2000.00	12.00	31.00	3	24	1.00	0.00
1.00	2500.00	358.82	10.00	19.00	17	36	0.00	0.00
-0.54	2500.00	411.11	12.00	31.00	9	40	1.00	0.00
0.10	3000.00	600.00	27.00	36.00	9	45	1.00	1.00
-1.06	2600.00	816.67	16.00	25.00	6	31	1.00	0.00
0.73	3800.00	446.15	18.00	27.00	13	40	1.00	0.00
0.90	2900.00	750.00	21.00	30.00	8	38	1.00	0.00
-0.92	2800.00	700.00	9.00	18.00	6	24	0.00	0.00
1.54	3100.00	352.17	52.00	61.00	23	84	1.00	1.00
1.15	2700.00	644.44	20.00	29.00	9	38	1.00	0.00
-1.46	3400.00	728.57	10.00	19.00	7	26	0.00	0.00

0.53	2900.00	265.22	16.00	25.00	23	48	1.00	0.00
-0.63	3000.00	816.67	14.00	23.00	6	29	0.00	0.00
0.68	2500.00	736.36	21.00	30.00	11	41	1.00	0.00
-0.04	3100.00	611.11	12.00	31.00	9	40	1.00	0.00
-0.06	2100.00	600.00	37.00	46.00	9	55	1.00	1.00
-0.31	2900.00	575.00	14.00	23.00	8	31	0.00	0.00
0.37	2900.00	454.55	21.00	30.00	11	41	1.00	0.00
0.17	3400.00	625.00	31.00	40.00	8	48	1.00	1.00
-0.59	3400.00	840.00	37.00	46.00	5	51	1.00	1.00
-0.14	2540.00	470.00	10.00	19.00	8	27	0.00	0.00
-0.19	2300.00	400.00	45.00	54.00	15	69	1.00	1.00
-0.47	2300.00	588.75	9.00	18.00	8	26	0.00	0.00
0.08	2500.00	408.33	32.00	41.00	12	53	1.00	1.00
1.06	2900.00	612.50	10.00	19.00	8	27	0.00	0.00
-0.58	2500.00	600.00	19.00	28.00	10	38	1.00	0.00
0.68	3300.00	663.64	32.00	41.00	11	52	1.00	1.00
-0.45	3000.00	1233.33	24.00	33.00	6	39	1.00	1.00
-1.47	3900.00	275.00	27.00	36.00	4	40	1.00	1.00
-0.51	3250.00	550.00	27.00	36.00	5	41	1.00	1.00
2.26	2900.00	408.33	26.00	35.00	24	59	1.00	1.00
1.17	3200.00	304.17	50.00	59.00	24	83	1.00	1.00
0.65	3200.00	685.71	24.00	33.00	7	40	1.00	1.00
2.15	3400.00	379.17	39.00	48.00	24	72	1.00	1.00
-1.47	3120.00	626.67	11.00	20.00	3	23	0.00	0.00
0.29	2900.00	828.57	19.00	28.00	7	35	1.00	0.00
-0.44	3000.00	633.33	21.00	30.00	6	36	1.00	0.00
-1.09	2000.00	850.00	10.00	19.00	4	23	0.00	0.00
-1.22	3200.00	800.00	29.00	38.00	3	41	1.00	1.00
-0.56	2800.00	625.00	10.00	19.00	8	27	0.00	0.00
1.15	3400.00	283.33	51.00	60.00	24	84	1.00	1.00
0.15	3000.00	633.33	10.00	19.00	9	28	0.00	0.00
-1.42	3500.00	650.00	43.00	52.00	2	54	1.00	1.00
-0.83	2800.00	650.00	12.00	21.00	4	25	0.00	0.00
-0.83	2900.00	1525.00	12.00	21.00	4	25	0.00	0.00
1.38	3000.00	290.00	55.00	64.00	20	84	1.00	1.00
-0.56	2300.00	850.00	10.00	19.00	4	23	0.00	0.00
-1.45	2600.00	1750.00	59.00	68.00	2	70	1.00	1.00
0.19	3100.00	671.43	83.00	92.00	7	99	1.00	1.00
-0.19	3800.00	700.00	9.00	18.00	6	24	0.00	0.00
0.73	2400.00	508.33	21.00	30.00	12	42	1.00	0.00
0.90	2500.00	578.57	30.00	39.00	14	53	1.00	1.00
-1.15	3200.00	342.86	22.00	31.00	7	38	1.00	0.00
0.14	3200.00	680.00	22.00	31.00	10	41	1.00	0.00
1.99	2800.00	395.83	26.00	35.00	24	59	1.00	1.00
-0.06	2700.00	423.08	9.00	18.00	13	31	0.00	0.00
-0.32	2700.00	1366.67	39.00	48.00	3	51	1.00	1.00
-0.72	3200.00	760.00	45.00	54.00	5	59	1.00	1.00

-0.99	2700.00	840.00	10.00	19.00	5	24	0.00	0.00
-0.60	3100.00	760.00	9.00	18.00	5	23	0.00	0.00
-0.55	2900.00	650.00	10.00	19.00	6	25	0.00	0.00
1.15	2000.00	320.00	67.00	76.00	20	96	1.00	1.00
-0.83	2800.00	800.00	22.00	31.00	5	36	1.00	0.00
-0.99	3100.00	1002.50	29.00	38.00	4	42	1.00	1.00
0.22	2900.00	688.89	32.00	41.00	9	50	1.00	1.00
0.27	2300.00	530.00	18.00	27.00	10	37	1.00	0.00
0.15	2900.00	383.33	27.00	36.00	12	48	1.00	1.00
0.78	3000.00	541.67	12.00	21.00	12	33	0.00	0.00
0.77	2900.00	393.33	60.00	69.00	15	84	1.00	1.00
0.19	3300.00	610.00	22.00	31.00	10	41	1.00	0.00
-1.22	2600.00	800.00	42.00	51.00	3	54	1.00	1.00
0.76	3200.00	644.44	12.00	21.00	9	30	0.00	0.00
0.91	3300.00	453.85	21.00	30.00	13	43	1.00	0.00
-0.96	3400.00	1400.00	37.00	46.00	2	48	1.00	1.00
0.06	3000.00	844.44	12.00	21.00	9	30	0.00	0.00
1.33	2400.00	309.52	42.00	51.00	21	72	1.00	1.00
0.27	3100.00	418.18	10.00	19.00	11	30	0.00	0.00
1.03	3000.00	576.92	26.00	35.00	13	48	1.00	1.00
-0.19	2900.00	883.33	9.00	18.00	6	24	0.00	0.00
-0.41	3500.00	942.86	14.00	23.00	7	30	0.00	0.00
0.88	2400.00	366.67	12.00	21.00	15	36	0.00	0.00
-0.47	3000.00	462.50	43.00	52.00	8	60	1.00	1.00
0.01	2200.00	663.75	10.00	19.00	8	27	0.00	0.00
-0.05	3000.00	409.09	22.00	31.00	11	42	1.00	0.00
-0.10	2800.00	637.50	19.00	28.00	8	36	1.00	0.00
-0.29	2100.00	527.27	10.00	19.00	11	30	0.00	0.00
-1.28	2500.00	1050.00	66.00	75.00	4	79	1.00	1.00
-0.42	3400.00	716.67	22.00	31.00	6	37	1.00	0.00
2.06	3000.00	345.45	21.00	30.00	22	52	1.00	0.00
-0.18	2400.00	350.00	10.00	19.00	14	33	0.00	0.00
-1.35	2600.00	500.00	35.00	44.00	5	49	1.00	1.00
0.73	2600.00	279.17	25.00	34.00	24	58	1.00	1.00
0.69	2610.00	898.57	21.00	30.00	7	37	1.00	0.00
0.15	3000.00	800.00	22.00	31.00	5	36	1.00	0.00
1.56	2680.00	346.67	32.00	41.00	24	65	1.00	1.00
-0.18	3100.00	811.11	22.00	31.00	9	40	1.00	0.00
-0.83	2800.00	1233.33	12.00	21.00	3	24	0.00	0.00
1.77	2800.00	345.83	39.00	48.00	24	72	1.00	1.00
1.86	3000.00	291.67	15.00	24.00	24	48	1.00	0.00
-0.69	2700.00	1160.00	8.00	17.00	5	22	0.00	0.00
-0.42	3400.00	716.67	30.00	39.00	6	45	1.00	1.00
-0.40	3100.00	816.67	57.00	66.00	6	72	1.00	1.00
-0.19	2500.00	600.00	12.00	31.00	9	40	1.00	0.00
1.26	2700.00	453.85	27.00	36.00	13	49	1.00	1.00
1.18	3300.00	266.67	15.00	24.00	24	48	1.00	0.00

-1.19	3700.00	866.67	9.00	18.00	3	21	0.00	0.00
-0.58	3700.00	920.00	63.00	72.00	5	77	1.00	1.00
-1.54	3400.00	566.67	11.00	20.00	3	23	0.00	0.00
-0.65	3300.00	450.00	17.00	26.00	6	32	1.00	0.00
1.56	2500.00	414.29	29.00	38.00	14	52	1.00	1.00
0.38	2500.00	677.78	10.00	19.00	9	38	0.00	0.00
0.19	2000.00	1200.00	16.00	25.00	6	31	1.00	0.00
-0.83	2900.00	800.00	34.00	43.00	5	48	1.00	1.00
0.32	3100.00	450.00	22.00	31.00	12	43	1.00	0.00
-1.99	3000.00	1333.33	32.00	41.00	3	44	1.00	1.00
-0.19	2900.00	536.36	23.00	32.00	11	43	1.00	0.00
1.05	2600.00	327.78	44.00	53.00	18	71	1.00	1.00
0.06	3400.00	757.14	24.00	33.00	7	40	1.00	1.00
1.60	2900.00	285.00	24.00	33.00	20	53	1.00	1.00
-0.19	2700.00	433.33	23.00	32.00	12	44	1.00	0.00
2.24	2900.00	478.26	67.00	76.00	23	99	1.00	1.00
0.32	3500.00	481.82	56.00	65.00	11	76	1.00	1.00
-0.96	3000.00	1733.33	12.00	21.00	3	24	0.00	0.00
-1.55	3500.00	1750.00	35.00	44.00	2	46	1.00	1.00
-0.19	2800.00	536.36	29.00	38.00	11	49	1.00	1.00
0.64	3200.00	640.00	23.00	32.00	10	42	1.00	0.00
0.71	3100.00	423.08	77.00	86.00	13	99	1.00	1.00
0.23	3600.00	475.00	20.00	29.00	8	37	1.00	0.00
-0.92	2700.00	675.00	14.00	23.00	4	27	0.00	0.00
-1.46	2600.00	1550.00	13.00	22.00	2	24	0.00	0.00
-0.95	2700.00	860.00	17.00	26.00	5	31	1.00	0.00
0.33	3000.00	527.27	24.00	33.00	11	44	1.00	1.00
0.17	3300.00	400.00	42.00	51.00	11	62	1.00	1.00
-0.45	2800.00	680.00	21.00	30.00	5	35	1.00	0.00
1.23	2600.00	300.00	10.00	19.00	23	42	0.00	0.00
-1.86	2900.00	1300.00	12.00	21.00	2	23	0.00	0.00
0.06	3200.00	860.00	10.00	19.00	5	24	0.00	0.00
-0.41	3400.00	380.00	17.00	26.00	10	36	1.00	0.00
0.97	4500.00	312.50	35.00	44.00	16	60	1.00	1.00
-0.88	2000.00	562.50	10.00	19.00	8	27	0.00	0.00
0.12	3800.00	711.11	37.00	46.00	9	55	1.00	1.00
0.97	2900.00	515.38	24.00	33.00	13	46	1.00	1.00
1.10	4400.00	581.82	25.00	34.00	11	45	1.00	1.00
-0.72	2600.00	850.00	16.00	25.00	6	31	1.00	0.00
0.96	3200.00	438.89	35.00	44.00	18	62	1.00	1.00
-0.06	3400.00	427.27	43.00	52.00	11	63	1.00	1.00
-0.83	2600.00	583.33	12.00	21.00	6	27	0.00	0.00
-0.19	3000.00	445.45	36.00	45.00	11	56	1.00	1.00
0.26	2500.00	627.27	42.00	51.00	11	62	1.00	1.00
-0.06	3700.00	437.50	26.00	35.00	8	43	1.00	1.00
-1.47	3900.00	725.00	11.00	20.00	4	24	0.00	0.00
0.71	2900.00	737.50	12.00	21.00	8	29	0.00	0.00

0.58	3100.00	375.00	41.00	50.00	16	66	1.00	1.00
-0.58	3100.00	1160.00	13.00	22.00	5	27	0.00	0.00
-0.78	3100.00	850.00	23.00	32.00	6	38	1.00	0.00
-0.71	3700.00	416.67	27.00	36.00	6	42	1.00	1.00
0.45	3500.00	376.92	22.00	31.00	13	44	1.00	0.00
1.09	3000.00	400.00	39.00	48.00	19	67	1.00	1.00
0.44	2900.00	438.46	13.00	22.00	13	35	0.00	0.00
-0.96	3300.00	1400.00	12.00	21.00	3	24	0.00	0.00
0.64	2500.00	406.25	27.00	36.00	16	52	1.00	1.00
-0.42	3200.00	550.00	24.00	33.00	8	41	1.00	1.00
0.83	3500.00	842.86	23.00	32.00	7	39	1.00	0.00
-0.45	3100.00	960.00	36.00	45.00	5	50	1.00	1.00
0.06	2600.00	440.00	10.00	19.00	10	29	0.00	0.00
0.19	2700.00	900.00	10.00	19.00	7	26	0.00	0.00
0.46	2900.00	461.54	25.00	34.00	13	47	1.00	1.00
1.09	3100.00	388.24	14.00	23.00	17	40	0.00	0.00
-0.45	3500.00	720.00	58.00	67.00	5	72	1.00	1.00
-1.99	3100.00	800.00	25.00	34.00	3	37	1.00	1.00
-0.08	3200.00	566.67	72.00	81.00	9	90	1.00	1.00
0.35	2700.00	266.67	24.00	33.00	18	51	1.00	1.00
1.09	3400.00	672.73	26.00	35.00	11	46	1.00	1.00
1.86	2900.00	312.50	16.00	25.00	24	49	1.00	0.00
0.58	2700.00	287.50	24.00	33.00	24	57	1.00	1.00
-1.86	3600.00	300.00	12.00	21.00	2	23	0.00	0.00
0.00	3100.00	644.44	25.00	34.00	9	43	1.00	1.00
-0.19	3900.00	625.00	38.00	47.00	8	55	1.00	1.00
-1.44	3000.00	1250.00	37.00	46.00	2	48	1.00	1.00
-1.73	2800.00	325.00	9.00	18.00	4	22	0.00	0.00
-1.22	3400.00	800.00	43.00	52.00	2	54	1.00	1.00
-0.45	2300.00	1300.00	10.00	19.00	4	23	0.00	0.00
0.85	3000.00	541.67	25.00	34.00	12	46	1.00	1.00
-0.19	2900.00	1228.57	24.00	33.00	7	40	1.00	1.00
-0.81	4100.00	141.18	12.00	21.00	17	38	0.00	0.00
-1.47	3700.00	475.00	17.00	26.00	4	30	1.00	0.00
-0.29	3100.00	780.00	43.00	52.00	5	57	1.00	1.00
0.96	3000.00	464.29	25.00	34.00	14	48	1.00	1.00
-0.58	2600.00	1020.00	23.00	32.00	5	37	1.00	0.00
-0.32	3000.00	600.00	10.00	19.00	5	24	0.00	0.00
-0.71	2300.00	1125.00	10.00	19.00	4	23	0.00	0.00
-0.96	3400.00	650.00	25.00	34.00	4	38	1.00	1.00
1.88	3000.00	404.35	40.00	49.00	23	72	1.00	1.00
-0.42	3400.00	487.50	23.00	32.00	8	40	1.00	0.00
0.58	3600.00	509.09	76.00	85.00	11	96	1.00	1.00
0.15	3000.00	633.33	49.00	58.00	9	67	1.00	1.00
-1.42	3000.00	833.33	11.00	20.00	3	23	0.00	0.00
0.79	3200.00	833.33	25.00	34.00	9	43	1.00	1.00
-0.04	2800.00	650.00	24.00	33.00	8	41	1.00	1.00

0.83	3100.00	360.00	36.00	45.00	15	60	1.00	1.00
-1.22	3000.00	416.67	15.00	24.00	12	36	1.00	0.00
0.26	3300.00	1116.67	14.00	33.00	6	39	1.00	0.00
0.58	2700.00	255.56	22.00	31.00	18	49	1.00	0.00
-1.41	2800.00	1350.00	13.00	22.00	2	24	0.00	0.00
2.12	2800.00	313.04	64.00	73.00	23	96	1.00	1.00
-0.32	2700.00	671.43	23.00	32.00	7	39	1.00	0.00
0.96	3800.00	400.00	27.00	36.00	13	49	1.00	1.00
-0.58	2400.00	651.11	12.00	21.00	9	30	0.00	0.00
-1.35	3900.00	240.00	35.00	44.00	5	49	1.00	1.00
0.24	2700.00	920.00	23.00	32.00	5	37	1.00	0.00
-1.79	3100.00	1400.00	36.00	45.00	2	47	1.00	1.00
0.83	3300.00	928.57	25.00	34.00	7	41	1.00	1.00
0.06	2200.00	445.45	15.00	24.00	11	35	1.00	0.00
-1.18	3000.00	1900.00	13.00	22.00	2	24	0.00	0.00
0.46	3200.00	391.67	15.00	24.00	12	36	1.00	0.00
0.64	3800.00	900.00	26.00	35.00	9	44	1.00	1.00
-0.03	2700.00	662.50	26.00	35.00	8	43	1.00	1.00
-0.19	2900.00	1266.67	24.00	33.00	6	39	1.00	1.00
-1.79	2500.00	616.67	20.00	29.00	6	35	1.00	0.00
-0.58	3100.00	550.00	23.00	32.00	8	40	1.00	0.00
0.86	2400.00	523.08	26.00	35.00	13	48	1.00	1.00
-1.15	3400.00	460.00	24.00	33.00	10	43	1.00	1.00
-0.77	2400.00	866.67	12.00	21.00	3	24	0.00	0.00
1.28	3400.00	383.33	26.00	35.00	18	53	1.00	1.00
-0.41	2900.00	666.67	9.00	18.00	6	24	0.00	0.00
0.74	2700.00	644.44	23.00	32.00	9	41	1.00	0.00
-1.21	3400.00	900.00	13.00	22.00	2	24	0.00	0.00
-0.77	3400.00	780.00	38.00	47.00	5	52	1.00	1.00
1.28	3100.00	323.81	34.00	43.00	21	64	1.00	1.00
0.78	2700.00	1028.57	14.00	23.00	7	30	0.00	0.00
0.67	3000.00	460.00	24.00	33.00	15	48	1.00	1.00
-1.45	3300.00	1750.00	37.00	46.00	2	48	1.00	1.00
0.90	3200.00	313.64	18.00	27.00	22	49	1.00	0.00
-0.40	2700.00	1050.00	9.00	18.00	4	22	0.00	0.00
-1.35	3000.00	975.00	10.00	19.00	4	23	0.00	0.00
0.88	3100.00	387.50	27.00	36.00	24	60	1.00	1.00
-1.15	2700.00	1650.00	22.00	31.00	2	33	1.00	0.00
-1.28	2500.00	1050.00	66.00	75.00	4	79	1.00	1.00
-1.99	2900.00	650.00	12.00	21.00	2	23	0.00	0.00
2.24	3000.00	379.17	59.00	68.00	24	92	1.00	1.00
-0.86	2800.00	725.00	22.00	31.00	8	39	1.00	0.00
0.23	3400.00	537.50	24.00	33.00	8	41	1.00	1.00
0.38	2100.00	338.10	35.00	44.00	21	65	1.00	1.00
-0.90	2600.00	542.86	42.00	51.00	7	58	1.00	1.00
-0.26	2000.00	392.31	22.00	31.00	13	44	1.00	0.00
-0.54	2500.00	575.00	38.00	47.00	8	55	1.00	1.00

0.96	3200.00	680.00	24.00	33.00	10	43	1.00	1.00
0.63	2700.00	407.69	16.00	25.00	13	38	1.00	0.00
1.73	3200.00	581.82	24.00	33.00	11	44	1.00	1.00
0.09	3500.00	572.73	20.00	29.00	11	40	1.00	0.00
0.45	2900.00	330.77	29.00	38.00	13	51	1.00	1.00
-1.35	2500.00	866.67	11.00	20.00	3	23	0.00	0.00
-0.06	2800.00	522.22	22.00	31.00	9	40	1.00	0.00
0.38	3300.00	407.14	61.00	70.00	14	84	1.00	1.00
-0.26	2600.00	880.00	10.00	19.00	5	24	0.00	0.00
-0.14	3300.00	814.29	23.00	32.00	7	39	1.00	0.00
0.72	3200.00	538.46	19.00	28.00	13	41	1.00	0.00
-0.06	3200.00	500.00	17.00	26.00	10	36	1.00	0.00
0.58	3100.00	610.00	22.00	31.00	10	41	1.00	0.00
1.47	2500.00	888.89	12.00	31.00	9	40	1.00	0.00
-0.58	2500.00	1366.67	27.00	36.00	3	39	1.00	1.00
-0.58	3300.00	700.00	34.00	43.00	7	50	1.00	1.00
-0.71	3300.00	760.00	52.00	61.00	5	66	1.00	1.00
0.19	3200.00	554.55	25.00	34.00	11	45	1.00	1.00
-1.06	3000.00	720.00	10.00	19.00	5	24	0.00	0.00
-1.35	3400.00	1333.33	61.00	70.00	3	73	1.00	1.00
0.56	2900.00	855.56	12.00	21.00	9	30	0.00	0.00
1.42	3000.00	220.00	36.00	45.00	20	65	1.00	1.00
0.79	3300.00	344.44	21.00	30.00	18	48	1.00	0.00
-1.33	3100.00	1500.00	13.00	22.00	2	24	0.00	0.00
-2.12	3300.00	550.00	22.00	31.00	4	35	1.00	0.00
-0.38	2000.00	1625.00	9.00	18.00	4	22	0.00	0.00
-0.37	3400.00	477.78	24.00	33.00	9	42	1.00	1.00
-1.44	3200.00	766.67	52.00	61.00	3	64	1.00	1.00
1.63	3000.00	345.83	53.00	62.00	24	86	1.00	1.00
-1.54	3000.00	1500.00	11.00	20.00	2	22	0.00	0.00
1.36	2800.00	900.00	16.00	25.00	7	32	1.00	0.00
1.86	2300.00	325.00	46.00	55.00	24	79	1.00	1.00
1.60	3000.00	341.67	40.00	49.00	24	73	1.00	1.00
0.44	2800.00	663.64	22.00	31.00	11	42	1.00	0.00
2.12	3000.00	425.00	27.00	36.00	24	60	1.00	1.00
0.85	3100.00	533.33	32.00	41.00	12	53	1.00	1.00
-0.28	2600.00	500.00	12.00	21.00	9	30	0.00	0.00
0.00	3100.00	353.85	44.00	53.00	13	66	1.00	1.00
-0.94	2900.00	600.00	11.00	20.00	3	23	0.00	0.00
0.38	2900.00	476.19	62.00	71.00	21	92	1.00	1.00
0.64	2800.00	944.44	22.00	31.00	9	40	1.00	0.00
-0.13	2800.00	933.33	22.00	31.00	6	37	1.00	0.00
2.50	3400.00	352.17	52.00	61.00	23	84	1.00	1.00
-0.32	2500.00	825.00	11.00	20.00	4	24	0.00	0.00
-1.45	3200.00	650.00	61.00	70.00	2	72	1.00	1.00
-0.32	2500.00	780.00	10.00	19.00	5	24	0.00	0.00
2.50	2800.00	486.36	41.00	50.00	22	72	1.00	1.00

1.35	3200.00	304.17	50.00	59.00	24	83	1.00	1.00
0.83	2700.00	887.50	12.00	21.00	8	29	0.00	0.00
-0.45	3100.00	628.57	23.00	32.00	7	39	1.00	0.00
-0.26	2800.00	1525.00	9.00	18.00	4	42	0.00	0.00
3.09	2500.00	412.50	63.00	72.00	24	96	1.00	1.00
-0.01	2700.00	527.27	12.00	21.00	11	32	0.00	0.00
1.09	2700.00	507.14	59.00	68.00	14	82	1.00	1.00
0.19	2700.00	550.00	22.00	31.00	10	41	1.00	0.00
1.09	3000.00	305.00	45.00	54.00	20	74	1.00	1.00
1.09	3000.00	512.50	24.00	33.00	8	41	1.00	1.00
1.09	3000.00	456.52	39.00	48.00	23	71	1.00	1.00
-0.26	2800.00	780.00	9.00	18.00	5	23	0.00	0.00
-0.13	3500.00	622.22	24.00	33.00	9	42	1.00	1.00
-0.28	2600.00	1120.00	66.00	75.00	5	80	1.00	1.00
-0.85	3400.00	833.33	12.00	21.00	3	24	0.00	0.00
1.54	3100.00	308.33	29.00	38.00	24	62	1.00	1.00
0.86	2500.00	690.91	10.00	19.00	11	30	0.00	0.00
-2.17	3000.00	900.00	26.00	35.00	2	37	1.00	1.00
-0.29	3200.00	633.33	31.00	40.00	6	46	1.00	1.00
0.90	3000.00	1000.00	24.00	33.00	9	42	1.00	1.00
-0.59	3200.00	1125.00	10.00	19.00	4	23	0.00	0.00
1.69	4000.00	325.00	39.00	48.00	24	72	1.00	1.00
1.41	2600.00	321.74	56.00	65.00	23	88	1.00	1.00
-0.51	2500.00	916.67	12.00	21.00	6	27	0.00	0.00
1.41	2800.00	461.90	32.00	41.00	21	62	1.00	1.00
-0.19	2300.00	525.00	10.00	19.00	12	31	0.00	0.00
0.08	2900.00	425.00	19.00	28.00	12	40	1.00	0.00
0.33	2500.00	445.45	16.00	25.00	11	36	1.00	0.00
-0.15	3500.00	757.14	25.00	34.00	7	41	1.00	1.00
2.47	3000.00	590.48	14.00	23.00	21	44	0.00	0.00
0.32	2700.00	438.46	22.00	31.00	13	44	1.00	0.00
-0.12	3000.00	687.50	24.00	33.00	8	41	1.00	1.00
1.64	4000.00	427.78	34.00	43.00	18	61	1.00	1.00
0.38	3200.00	588.89	25.00	34.00	9	43	1.00	1.00
-0.35	2800.00	800.00	11.00	20.00	4	24	0.00	0.00
-1.21	2900.00	1266.67	35.00	44.00	3	47	1.00	1.00
-1.50	2400.00	1266.67	31.00	40.00	3	43	1.00	1.00
1.09	2200.00	566.67	12.00	21.00	12	33	0.00	0.00
0.72	3000.00	533.33	45.00	54.00	18	72	1.00	1.00
-1.53	2800.00	750.00	18.00	27.00	4	31	1.00	0.00
-1.58	3500.00	900.00	38.00	47.00	2	49	1.00	1.00
-0.85	2700.00	616.67	9.00	18.00	6	24	0.00	0.00
-1.08	2800.00	1433.33	14.00	23.00	3	26	0.00	0.00
0.26	3000.00	571.43	24.00	33.00	7	30	1.00	1.00
-2.12	2700.00	966.67	11.00	20.00	3	23	0.00	0.00
-1.22	3000.00	1233.33	10.00	19.00	3	22	0.00	0.00
-0.38	3200.00	400.00	45.00	54.00	8	62	1.00	1.00

-0.45	3300.00	875.00	9.00	18.00	4	22	0.00	0.00
-2.18	2400.00	1075.00	23.00	32.00	4	36	1.00	0.00
-1.41	3100.00	280.00	24.00	33.00	5	38	1.00	1.00
-2.17	3300.00	433.33	41.00	50.00	3	53	1.00	1.00
0.33	3300.00	375.00	24.00	33.00	12	45	1.00	1.00
1.26	3000.00	333.33	27.00	36.00	18	54	1.00	1.00
-0.04	2100.00	611.11	10.00	19.00	9	38	0.00	0.00
-0.32	2700.00	511.11	37.00	46.00	9	55	1.00	1.00
-0.13	3000.00	1240.00	24.00	33.00	5	38	1.00	1.00
-0.38	2500.00	800.00	45.00	54.00	8	62	1.00	1.00
-1.44	3200.00	1050.00	52.00	61.00	2	63	1.00	1.00
-1.54	2800.00	1233.33	11.00	20.00	3	23	0.00	0.00
-0.29	3000.00	1100.00	12.00	21.00	3	24	0.00	0.00
-0.38	3400.00	840.00	37.00	46.00	5	51	1.00	1.00

BTP	BTB	Agediff	BTB2	Wt	Wtdifferen	Growth	BTB3
3.00	3.00	47.00	1.00	12900.00	9400.00	447.62	3.00
1.00	2.00	31.00	0.00	9100.00	6200.00	563.64	2.00
1.00	2.00	29.00	0.00	10400.00	6800.00	523.08	2.00
1.00	2.00	32.00	0.00	7500.00	4600.00	575.00	2.00
3.00	4.00	61.00	1.00	5200.00	2000.00	1000.00	3.00
3.00	3.00	46.00	1.00	8900.00	5900.00	983.33	3.00
1.00	1.00	13.00	0.00	8900.00	5400.00	450.00	1.00
1.00	1.00	12.00	0.00	8200.00	4900.00	376.92	1.00
4.00	4.00	72.00	1.00	7400.00	3900.00	975.00	3.00
2.00	2.00	34.00	0.00	7200.00	3800.00	542.86	2.00
1.00	1.00	23.00	0.00	7100.00	4700.00	1175.00	1.00
2.00	3.00	38.00	1.00	8300.00	4700.00	1175.00	3.00
1.00	1.00	19.00	0.00	7400.00	4800.00	960.00	1.00
1.00	1.00	21.00	0.00	6900.00	4400.00	550.00	1.00
4.00	4.00	95.00	1.00	4100.00	2000.00	666.67	3.00
1.00	1.00	23.00	0.00	6200.00	3700.00	740.00	1.00
2.00	3.00	37.00	1.00	8200.00	5600.00	933.33	3.00
2.00	2.00	35.00	0.00	6600.00	2600.00	236.36	2.00
1.00	1.00	21.00	0.00	6700.00	4000.00	1333.33	1.00
4.00	4.00	84.00	1.00	6700.00	3700.00	1233.33	3.00
4.00	4.00	76.00	1.00	7300.00	4600.00	920.00	3.00
1.00	1.00	19.00	0.00	7500.00	4700.00	587.50	1.00
3.00	4.00	64.00	1.00	9700.00	6900.00	862.50	3.00
1.00	2.00	32.00	0.00	8900.00	6000.00	500.00	2.00
3.00	3.00	53.00	1.00	9300.00	6800.00	357.89	3.00
4.00	4.00	80.00	1.00	7300.00	4300.00	614.29	3.00
2.00	3.00	44.00	1.00	7300.00	4000.00	1000.00	3.00
2.00	2.00	35.00	0.00	9000.00	5800.00	644.44	2.00
4.00	4.00	83.00	1.00	10500.00	7000.00	538.46	3.00
2.00	2.00	34.00	0.00	9700.00	6600.00	825.00	2.00
1.00	2.00	32.00	0.00	7700.00	4800.00	600.00	2.00
1.00	2.00	25.00	0.00	8200.00	5500.00	785.71	2.00
3.00	4.00	61.00	1.00	9400.00	6700.00	609.09	3.00
1.00	2.00	28.00	0.00	8000.00	5600.00	509.09	2.00
1.00	2.00	31.00	0.00	9300.00	6400.00	581.82	2.00
1.00	2.00	32.00	0.00	9200.00	6400.00	1066.67	2.00
1.00	2.00	25.00	0.00	13500.00	10900.00	545.00	2.00
1.00	1.00	21.00	0.00	10900.00	8400.00	466.67	1.00
1.00	2.00	31.00	0.00	7500.00	4500.00	409.09	2.00
1.00	1.00	21.00	0.00	8000.00	5400.00	540.00	1.00
3.00	3.00	47.00	1.00	12000.00	9100.00	379.17	3.00
1.00	2.00	30.00	0.00	9500.00	7200.00	600.00	2.00
1.00	2.00	30.00	0.00	9300.00	7600.00	633.33	2.00
3.00	3.00	49.00	1.00	9900.00	6700.00	291.30	3.00
2.00	3.00	40.00	1.00	12000.00	9200.00	383.33	3.00
2.00	2.00	35.00	0.00	12000.00	8900.00	684.62	2.00

1.00	1.00	21.00	0.00	13100.00	10300.00	429.17	1.00
2.00	1.00	15.00	0.00	7400.00	4000.00	571.43	1.00
3.00	3.00	51.00	1.00	5500.00	2900.00	580.00	3.00
3.00	4.00	66.00	1.00	6900.00	4000.00	666.67	3.00
1.00	2.00	30.00	0.00	6200.00	3400.00	566.67	2.00
3.00	4.00	60.00	1.00	6800.00	3800.00	1266.67	3.00
1.00	2.00	30.00	0.00	7000.00	4400.00	400.00	2.00
1.00	2.00	30.00	0.00	10000.00	7300.00	384.21	2.00
3.00	3.00	48.00	1.00	10000.00	6850.00	285.42	3.00
1.00	1.00	19.00	0.00	9500.00	6100.00	1220.00	1.00
1.00	2.00	30.00	0.00	9500.00	6800.00	680.00	2.00
1.00	2.00	30.00	0.00	8500.00	4600.00	328.57	2.00
3.00	3.00	46.00	1.00	4500.00	1900.00	950.00	3.00
1.00	2.00	29.00	0.00	10300.00	7200.00	360.00	2.00
1.00	2.00	30.00	0.00	6500.00	4200.00	1050.00	2.00
1.00	2.00	29.00	0.00	12000.00	9200.00	383.33	2.00
1.00	1.00	18.00	0.00	5500.00	2600.00	1300.00	1.00
1.00	1.00	23.00	0.00	9000.00	6300.00	484.62	1.00
3.00	3.00	45.00	1.00	10000.00	7200.00	342.86	3.00
1.00	1.00	23.00	0.00	6500.00	4100.00	683.33	1.00
1.00	2.00	30.00	0.00	6000.00	2300.00	460.00	2.00
1.00	2.00	30.00	0.00	10600.00	7900.00	359.09	2.00
1.00	2.00	31.00	0.00	10200.00	7700.00	770.00	2.00
2.00	2.00	35.00	0.00	6600.00	3600.00	720.00	2.00
1.00	2.00	30.00	0.00	9000.00	5800.00	483.33	2.00
1.00	2.00	31.00	0.00	7400.00	4300.00	614.29	2.00
1.00	2.00	27.00	0.00	7000.00	3700.00	462.50	2.00
1.00	1.00	20.00	0.00	7400.00	4800.00	436.36	1.00
1.00	1.00	20.00	0.00	7100.00	4100.00	1025.00	1.00
2.00	2.00	33.00	0.00	10700.00	8100.00	540.00	2.00
1.00	2.00	31.00	0.00	10100.00	7100.00	1183.33	2.00
1.00	1.00	18.00	0.00	8600.00	5300.00	294.44	1.00
1.00	1.00	18.00	0.00	6000.00	3300.00	1100.00	1.00
3.00	3.00	57.00	1.00	8800.00	5700.00	518.18	3.00
3.00	3.00	55.00	1.00	9500.00	6800.00	400.00	3.00
1.00	2.00	30.00	0.00	8300.00	5500.00	500.00	2.00
1.00	2.00	31.00	0.00	9300.00	6300.00	630.00	2.00
3.00	3.00	46.00	1.00	8400.00	5500.00	366.67	3.00
1.00	2.00	31.00	0.00	8000.00	5600.00	800.00	2.00
1.00	2.00	25.00	0.00	13000.00	9900.00	412.50	2.00
2.00	3.00	36.00	1.00	8000.00	4800.00	252.63	3.00
1.00	2.00	30.00	0.00	7300.00	4900.00	350.00	2.00
2.00	2.00	33.00	0.00	7700.00	4300.00	860.00	2.00
1.00	2.00	30.00	0.00	8000.00	5100.00	425.00	2.00
3.00	4.00	61.00	1.00	8200.00	5600.00	509.09	3.00
1.00	1.00	21.00	0.00	5100.00	2000.00	666.67	1.00
1.00	2.00	29.00	0.00	6400.00	3500.00	700.00	2.00

2.00	2.00	34.00	0.00	9000.00	6100.00	265.22	2.00
1.00	2.00	31.00	0.00	8000.00	4400.00	550.00	2.00
2.00	3.00	43.00	1.00	5900.00	3000.00	600.00	3.00
1.00	2.00	31.00	0.00	6700.00	4200.00	1400.00	2.00
1.00	2.00	31.00	0.00	8700.00	6200.00	364.71	2.00
1.00	2.00	31.00	0.00	6000.00	3200.00	533.33	2.00
3.00	3.00	48.00	1.00	8300.00	5500.00	458.33	3.00
1.00	2.00	31.00	0.00	8300.00	5700.00	1425.00	2.00
3.00	3.00	57.00	1.00	7500.00	4200.00	420.00	3.00
1.00	2.00	31.00	0.00	8200.00	5600.00	466.67	2.00
2.00	3.00	39.00	1.00	7800.00	4800.00	533.33	3.00
1.00	2.00	32.00	0.00	7800.00	4800.00	1600.00	2.00
3.00	3.00	53.00	1.00	9600.00	6300.00	331.58	3.00
2.00	3.00	38.00	1.00	8400.00	5400.00	675.00	3.00
3.00	3.00	48.00	1.00	14000.00	10900.00	454.17	3.00
1.00	2.00	30.00	0.00	6900.00	4000.00	1000.00	2.00
1.00	1.00	20.00	0.00	5500.00	2600.00	650.00	1.00
1.00	2.00	27.00	0.00	6800.00	3500.00	875.00	2.00
2.00	3.00	44.00	1.00	9000.00	6300.00	393.75	3.00
1.00	2.00	31.00	0.00	5300.00	2600.00	866.67	2.00
1.00	2.00	27.00	0.00	11400.00	9500.00	475.00	2.00
2.00	3.00	38.00	1.00	9300.00	6700.00	279.17	3.00
1.00	1.00	19.00	0.00	5200.00	2000.00	666.67	1.00
2.00	3.00	36.00	1.00	12100.00	9200.00	400.00	3.00
2.00	3.00	42.00	1.00	10000.00	7400.00	321.74	3.00
1.00	1.00	19.00	0.00	6400.00	3300.00	660.00	1.00
1.00	2.00	29.00	0.00	6100.00	3800.00	760.00	2.00
1.00	2.00	25.00	0.00	9700.00	6000.00	545.45	2.00
1.00	1.00	18.00	0.00	6100.00	2800.00	700.00	1.00
2.00	3.00	41.00	1.00	9500.00	7000.00	368.42	3.00
1.00	1.00	23.00	0.00	9000.00	6400.00	914.29	1.00
1.00	1.00	20.00	0.00	6000.00	3000.00	1000.00	1.00
2.00	3.00	38.00	1.00	4800.00	1800.00	900.00	3.00
1.00	1.00	19.00	0.00	7800.00	4500.00	900.00	1.00
3.00	4.00	68.00	1.00	10100.00	6800.00	357.89	3.00
1.00	1.00	21.00	0.00	6800.00	3800.00	633.33	1.00
1.00	1.00	21.00	0.00	6000.00	2800.00	1400.00	1.00
2.00	3.00	40.00	1.00	9400.00	7000.00	411.76	3.00
1.00	1.00	20.00	0.00	6900.00	3900.00	487.50	1.00
1.00	2.00	32.00	0.00	8000.00	5300.00	331.25	2.00
1.00	1.00	23.00	0.00	7200.00	4000.00	666.67	1.00
2.00	3.00	38.00	1.00	10000.00	7000.00	700.00	3.00
1.00	1.00	21.00	0.00	7900.00	4500.00	1500.00	1.00
1.00	2.00	30.00	0.00	7500.00	4600.00	418.18	2.00
1.00	2.00	32.00	0.00	7000.00	4200.00	600.00	2.00
1.00	1.00	21.00	0.00	6400.00	3800.00	475.00	1.00
1.00	2.00	30.00	0.00	5400.00	2700.00	450.00	2.00

3.00	4.00	61.00	1.00	11600.00	8400.00	381.82	3.00
1.00	1.00	21.00	0.00	7400.00	5000.00	833.33	1.00
1.00	2.00	26.00	0.00	10300.00	7000.00	368.42	2.00
1.00	2.00	32.00	0.00	6000.00	3000.00	750.00	2.00
3.00	3.00	46.00	1.00	5200.00	2300.00	1150.00	3.00
1.00	1.00	21.00	0.00	8400.00	5400.00	675.00	1.00
1.00	1.00	19.00	0.00	7000.00	3500.00	700.00	1.00
2.00	3.00	41.00	1.00	8700.00	5700.00	814.29	3.00
1.00	1.00	19.00	0.00	9000.00	6100.00	677.78	1.00
1.00	1.00	118.00	0.00	8700.00	6100.00	435.71	1.00
1.00	1.00	19.00	0.00	8000.00	5000.00	1000.00	1.00
3.00	3.00	46.00	1.00	4600.00	1200.00	600.00	3.00
1.00	2.00	29.00	0.00	10900.00	7600.00	380.00	2.00
1.00	1.00	19.00	0.00	10000.00	6400.00	640.00	1.00
3.00	3.00	49.00	1.00	9300.00	6500.00	382.35	3.00
1.00	1.00	19.00	0.00	9000.00	6000.00	666.67	1.00
1.00	2.00	30.00	0.00	8900.00	6300.00	262.50	2.00
1.00	1.00	19.00	0.00	9600.00	6600.00	733.33	1.00
4.00	4.00	79.00	1.00	8300.00	5700.00	335.29	3.00
1.00	1.00	21.00	0.00	9000.00	5000.00	714.29	1.00
1.00	2.00	32.00	0.00	8900.00	5900.00	368.75	2.00
1.00	1.00	21.00	0.00	7800.00	5100.00	1275.00	1.00
1.00	1.00	23.00	0.00	6400.00	3000.00	428.57	1.00
2.00	3.00	37.00	1.00	9100.00	6300.00	273.91	3.00
3.00	3.00	48.00	1.00	10000.00	6500.00	270.83	3.00
1.00	1.00	21.00	0.00	7200.00	3800.00	422.22	1.00
4.00	4.00	70.00	1.00	14500.00	11700.00	1063.64	3.00
1.00	1.00	20.00	0.00	8500.00	5600.00	700.00	1.00
3.00	3.00	45.00	1.00	7600.00	4600.00	766.67	3.00
1.00	1.00	19.00	0.00	7900.00	5000.00	714.29	1.00
2.00	2.00	35.00	0.00	10200.00	6000.00	315.79	2.00
1.00	1.00	20.00	0.00	6000.00	3500.00	875.00	1.00
2.00	3.00	36.00	1.00	8400.00	5900.00	453.85	3.00
3.00	3.00	54.00	1.00	10500.00	7600.00	330.43	3.00
1.00	1.00	19.00	0.00	8000.00	4800.00	685.71	1.00
1.00	1.00	19.00	0.00	5000.00	2000.00	1000.00	1.00
1.00	2.00	21.00	0.00	8800.00	6000.00	2000.00	2.00
1.00	1.00	19.00	0.00	8600.00	6100.00	358.82	1.00
1.00	2.00	31.00	0.00	6200.00	3700.00	411.11	2.00
2.00	3.00	36.00	1.00	8400.00	5400.00	600.00	3.00
1.00	2.00	25.00	0.00	7500.00	4900.00	816.67	2.00
1.00	2.00	27.00	0.00	9600.00	5800.00	446.15	2.00
1.00	2.00	30.00	0.00	8900.00	6000.00	750.00	2.00
1.00	1.00	18.00	0.00	7000.00	4200.00	700.00	1.00
3.00	4.00	61.00	1.00	11200.00	8100.00	352.17	3.00
1.00	2.00	29.00	0.00	8500.00	5800.00	644.44	2.00
1.00	1.00	19.00	0.00	8500.00	5100.00	728.57	1.00

1.00	2.00	25.00	0.00	9000.00	6100.00	265.22	2.00
1.00	1.00	23.00	0.00	7900.00	4900.00	816.67	1.00
1.00	2.00	30.00	0.00	10600.00	8100.00	736.36	2.00
1.00	2.00	31.00	0.00	8600.00	5500.00	611.11	2.00
3.00	3.00	46.00	1.00	7500.00	5400.00	600.00	3.00
1.00	1.00	23.00	0.00	7500.00	4600.00	575.00	1.00
1.00	2.00	30.00	0.00	7900.00	5000.00	454.55	2.00
2.00	3.00	40.00	1.00	8400.00	5000.00	625.00	3.00
3.00	3.00	46.00	1.00	7600.00	4200.00	840.00	3.00
1.00	1.00	19.00	0.00	6300.00	3760.00	470.00	1.00
3.00	3.00	54.00	1.00	8300.00	6000.00	400.00	3.00
1.00	1.00	18.00	0.00	7010.00	4710.00	588.75	1.00
2.00	3.00	41.00	1.00	7400.00	4900.00	408.33	3.00
1.00	1.00	19.00	0.00	7800.00	4900.00	612.50	1.00
1.00	2.00	28.00	0.00	8500.00	6000.00	600.00	2.00
2.00	3.00	41.00	1.00	10600.00	7300.00	663.64	3.00
2.00	2.00	33.00	0.00	10400.00	7400.00	1233.33	2.00
2.00	3.00	36.00	1.00	5000.00	1100.00	275.00	3.00
2.00	3.00	36.00	1.00	6000.00	2750.00	550.00	3.00
2.00	2.00	35.00	0.00	12700.00	9800.00	408.33	2.00
3.00	3.00	59.00	1.00	10500.00	7300.00	304.17	3.00
2.00	2.00	33.00	0.00	8000.00	4800.00	685.71	2.00
3.00	3.00	48.00	1.00	12500.00	9100.00	379.17	3.00
1.00	1.00	20.00	0.00	5000.00	1880.00	626.67	1.00
1.00	2.00	28.00	0.00	8700.00	5800.00	828.57	2.00
1.00	2.00	30.00	0.00	6800.00	3800.00	633.33	2.00
1.00	1.00	19.00	0.00	5400.00	3400.00	850.00	1.00
2.00	3.00	38.00	1.00	5600.00	2400.00	800.00	3.00
1.00	1.00	19.00	0.00	7800.00	5000.00	625.00	1.00
3.00	4.00	60.00	1.00	10200.00	6800.00	283.33	3.00
1.00	1.00	19.00	0.00	8700.00	5700.00	633.33	1.00
3.00	3.00	52.00	1.00	4800.00	1300.00	650.00	3.00
1.00	1.00	21.00	0.00	5400.00	2600.00	650.00	1.00
1.00	1.00	21.00	0.00	9000.00	6100.00	1525.00	1.00
3.00	4.00	64.00	1.00	8800.00	5800.00	290.00	3.00
1.00	1.00	19.00	0.00	5700.00	3400.00	850.00	1.00
3.00	4.00	68.00	1.00	6100.00	3500.00	1750.00	3.00
4.00	4.00	92.00	1.00	7800.00	4700.00	671.43	3.00
1.00	1.00	18.00	0.00	8000.00	4200.00	700.00	1.00
1.00	2.00	30.00	0.00	8500.00	6100.00	508.33	2.00
2.00	3.00	39.00	1.00	10600.00	8100.00	578.57	3.00
1.00	2.00	31.00	0.00	5600.00	2400.00	342.86	2.00
1.00	2.00	31.00	0.00	10000.00	6800.00	680.00	2.00
2.00	2.00	35.00	0.00	12300.00	9500.00	395.83	2.00
1.00	1.00	18.00	0.00	8200.00	5500.00	423.08	1.00
3.00	3.00	48.00	1.00	6800.00	4100.00	1366.67	3.00
3.00	3.00	54.00	1.00	7000.00	3800.00	760.00	3.00

1.00	1.00	19.00	0.00	6900.00	4200.00	840.00	1.00
1.00	1.00	18.00	0.00	6900.00	3800.00	760.00	1.00
1.00	1.00	19.00	0.00	6800.00	3900.00	650.00	1.00
4.00	4.00	76.00	1.00	8400.00	6400.00	320.00	3.00
1.00	2.00	31.00	0.00	6800.00	4000.00	800.00	2.00
2.00	3.00	38.00	1.00	7110.00	4010.00	1002.50	3.00
2.00	3.00	41.00	1.00	9100.00	6200.00	688.89	3.00
1.00	2.00	27.00	0.00	7600.00	5300.00	530.00	2.00
2.00	3.00	36.00	1.00	7500.00	4600.00	383.33	3.00
1.00	1.00	21.00	0.00	9500.00	6500.00	541.67	1.00
4.00	4.00	69.00	1.00	8800.00	5900.00	393.33	3.00
1.00	2.00	31.00	0.00	9400.00	6100.00	610.00	2.00
3.00	3.00	51.00	1.00	5000.00	2400.00	800.00	3.00
1.00	1.00	21.00	0.00	9000.00	5800.00	644.44	1.00
1.00	2.00	30.00	0.00	9200.00	5900.00	453.85	2.00
3.00	3.00	46.00	1.00	6200.00	2800.00	1400.00	3.00
1.00	1.00	21.00	0.00	10600.00	7600.00	844.44	1.00
3.00	3.00	51.00	1.00	8900.00	6500.00	309.52	3.00
1.00	1.00	19.00	0.00	7700.00	4600.00	418.18	1.00
2.00	2.00	35.00	0.00	10500.00	7500.00	576.92	2.00
1.00	1.00	18.00	0.00	8200.00	5300.00	883.33	1.00
1.00	1.00	23.00	0.00	10100.00	6600.00	942.86	1.00
1.00	1.00	21.00	0.00	7900.00	5500.00	366.67	1.00
3.00	3.00	52.00	1.00	6700.00	3700.00	462.50	3.00
1.00	1.00	19.00	0.00	7510.00	5310.00	663.75	1.00
1.00	2.00	31.00	0.00	7500.00	4500.00	409.09	2.00
1.00	2.00	28.00	0.00	7900.00	5100.00	637.50	2.00
1.00	1.00	19.00	0.00	7900.00	5800.00	527.27	1.00
4.00	4.00	75.00	1.00	6700.00	4200.00	1050.00	3.00
1.00	2.00	31.00	0.00	7700.00	4300.00	716.67	2.00
1.00	2.00	30.00	0.00	10600.00	7600.00	345.45	2.00
1.00	1.00	19.00	0.00	7300.00	4900.00	350.00	1.00
2.00	3.00	44.00	1.00	5100.00	2500.00	500.00	3.00
2.00	2.00	34.00	0.00	9300.00	6700.00	279.17	2.00
1.00	2.00	30.00	0.00	8900.00	6290.00	898.57	2.00
1.00	2.00	31.00	0.00	7000.00	4000.00	800.00	2.00
2.00	3.00	41.00	1.00	11000.00	8320.00	346.67	3.00
1.00	2.00	31.00	0.00	10400.00	7300.00	811.11	2.00
1.00	1.00	21.00	0.00	6500.00	3700.00	1233.33	1.00
3.00	3.00	48.00	1.00	11100.00	8300.00	345.83	3.00
1.00	2.00	24.00	0.00	10000.00	7000.00	291.67	2.00
1.00	1.00	17.00	0.00	8500.00	5800.00	1160.00	1.00
2.00	3.00	39.00	1.00	7700.00	4300.00	716.67	3.00
3.00	4.00	66.00	1.00	8000.00	4900.00	816.67	3.00
1.00	2.00	31.00	0.00	7900.00	5400.00	600.00	2.00
2.00	3.00	36.00	1.00	8600.00	5900.00	453.85	3.00
1.00	2.00	24.00	0.00	9700.00	6400.00	266.67	2.00

1.00	1.00	18.00	0.00	6300.00	2600.00	866.67	1.00
4.00	4.00	72.00	1.00	8300.00	4600.00	920.00	3.00
1.00	1.00	20.00	0.00	5100.00	1700.00	566.67	1.00
1.00	2.00	26.00	0.00	6000.00	2700.00	450.00	2.00
2.00	3.00	38.00	1.00	8300.00	5800.00	414.29	3.00
1.00	1.00	29.00	0.00	8600.00	6100.00	677.78	1.00
1.00	2.00	25.00	0.00	9200.00	7200.00	1200.00	2.00
2.00	3.00	43.00	1.00	6900.00	4000.00	800.00	3.00
1.00	2.00	31.00	0.00	8500.00	5400.00	450.00	2.00
2.00	3.00	41.00	1.00	7000.00	4000.00	1333.33	3.00
1.00	2.00	32.00	0.00	8800.00	5900.00	536.36	2.00
3.00	3.00	53.00	1.00	8500.00	5900.00	327.78	3.00
2.00	2.00	33.00	0.00	8700.00	5300.00	757.14	2.00
2.00	2.00	33.00	0.00	8600.00	5700.00	285.00	2.00
1.00	2.00	32.00	0.00	7900.00	5200.00	433.33	2.00
4.00	4.00	76.00	1.00	13900.00	11000.00	478.26	3.00
3.00	4.00	65.00	1.00	8800.00	5300.00	481.82	3.00
1.00	1.00	21.00	0.00	8200.00	5200.00	1733.33	1.00
2.00	3.00	44.00	1.00	7000.00	3500.00	1750.00	3.00
2.00	3.00	38.00	1.00	8700.00	5900.00	536.36	3.00
1.00	2.00	32.00	0.00	9600.00	6400.00	640.00	2.00
4.00	4.00	86.00	1.00	8600.00	5500.00	423.08	3.00
1.00	2.00	29.00	0.00	7400.00	3800.00	475.00	2.00
1.00	1.00	23.00	0.00	5400.00	2700.00	675.00	1.00
1.00	1.00	22.00	0.00	5700.00	3100.00	1550.00	1.00
1.00	2.00	26.00	0.00	7000.00	4300.00	860.00	2.00
2.00	2.00	33.00	0.00	8800.00	5800.00	527.27	2.00
3.00	3.00	51.00	1.00	7700.00	4400.00	400.00	3.00
1.00	2.00	30.00	0.00	6200.00	3400.00	680.00	2.00
1.00	1.00	19.00	0.00	9500.00	6900.00	300.00	1.00
1.00	1.00	21.00	0.00	5500.00	2600.00	1300.00	1.00
1.00	1.00	19.00	0.00	7500.00	4300.00	860.00	1.00
1.00	2.00	26.00	0.00	7200.00	3800.00	380.00	2.00
2.00	3.00	44.00	1.00	9500.00	5000.00	312.50	3.00
1.00	1.00	19.00	0.00	6500.00	4500.00	562.50	1.00
3.00	3.00	46.00	1.00	10200.00	6400.00	711.11	3.00
2.00	2.00	33.00	0.00	9600.00	6700.00	515.38	2.00
2.00	2.00	34.00	0.00	10800.00	6400.00	581.82	2.00
1.00	2.00	25.00	0.00	7700.00	5100.00	850.00	2.00
2.00	3.00	44.00	1.00	11100.00	7900.00	438.89	3.00
3.00	3.00	52.00	1.00	8100.00	4700.00	427.27	3.00
1.00	1.00	21.00	0.00	6100.00	3500.00	583.33	1.00
3.00	3.00	45.00	1.00	7900.00	4900.00	445.45	3.00
3.00	3.00	51.00	1.00	9400.00	6900.00	627.27	3.00
2.00	2.00	35.00	0.00	7200.00	3500.00	437.50	2.00
1.00	1.00	20.00	0.00	6800.00	2900.00	725.00	1.00
1.00	1.00	21.00	0.00	8800.00	5900.00	737.50	1.00

3.00	3.00	50.00	1.00	9100.00	6000.00	375.00	3.00
1.00	1.00	22.00	0.00	8900.00	5800.00	1160.00	1.00
1.00	2.00	32.00	0.00	8200.00	5100.00	850.00	2.00
2.00	3.00	36.00	1.00	6200.00	2500.00	416.67	3.00
1.00	2.00	31.00	0.00	8400.00	4900.00	376.92	2.00
3.00	3.00	48.00	1.00	10600.00	7600.00	400.00	3.00
1.00	1.00	22.00	0.00	8600.00	5700.00	438.46	1.00
1.00	1.00	21.00	0.00	7500.00	4200.00	1400.00	1.00
2.00	3.00	36.00	1.00	9000.00	6500.00	406.25	3.00
2.00	2.00	33.00	0.00	7600.00	4400.00	550.00	2.00
1.00	2.00	32.00	0.00	9400.00	5900.00	842.86	2.00
3.00	3.00	45.00	1.00	7900.00	4800.00	960.00	3.00
1.00	1.00	19.00	0.00	7000.00	4400.00	440.00	1.00
1.00	1.00	19.00	0.00	9000.00	6300.00	900.00	1.00
2.00	2.00	34.00	0.00	8900.00	6000.00	461.54	2.00
1.00	1.00	23.00	0.00	9700.00	6600.00	388.24	1.00
3.00	4.00	67.00	1.00	7100.00	3600.00	720.00	3.00
2.00	2.00	34.00	0.00	5500.00	2400.00	800.00	2.00
4.00	4.00	81.00	1.00	8300.00	5100.00	566.67	3.00
2.00	2.00	33.00	0.00	7500.00	4800.00	266.67	2.00
2.00	2.00	35.00	0.00	10800.00	7400.00	672.73	2.00
1.00	2.00	25.00	0.00	10400.00	7500.00	312.50	2.00
2.00	2.00	33.00	0.00	9600.00	6900.00	287.50	2.00
1.00	1.00	21.00	0.00	4200.00	600.00	300.00	1.00
2.00	2.00	34.00	0.00	8900.00	5800.00	644.44	2.00
3.00	3.00	47.00	1.00	8900.00	5000.00	625.00	3.00
3.00	3.00	46.00	1.00	5500.00	2500.00	1250.00	3.00
1.00	1.00	18.00	0.00	4100.00	1300.00	325.00	1.00
3.00	3.00	52.00	1.00	5000.00	1600.00	800.00	3.00
1.00	1.00	19.00	0.00	7500.00	5200.00	1300.00	1.00
2.00	2.00	34.00	0.00	9500.00	6500.00	541.67	2.00
2.00	2.00	33.00	0.00	11500.00	8600.00	1228.57	2.00
1.00	1.00	21.00	0.00	6500.00	2400.00	141.18	1.00
1.00	2.00	26.00	0.00	5600.00	1900.00	475.00	2.00
3.00	3.00	52.00	1.00	7000.00	3900.00	780.00	3.00
2.00	2.00	34.00	0.00	9500.00	6500.00	464.29	2.00
1.00	2.00	32.00	0.00	7700.00	5100.00	1020.00	2.00
1.00	1.00	19.00	0.00	6000.00	3000.00	600.00	1.00
1.00	1.00	19.00	0.00	6800.00	4500.00	1125.00	1.00
2.00	2.00	34.00	0.00	6000.00	2600.00	650.00	2.00
3.00	3.00	49.00	1.00	12300.00	9300.00	404.35	3.00
1.00	2.00	32.00	0.00	7300.00	3900.00	487.50	2.00
4.00	4.00	85.00	1.00	9200.00	5600.00	509.09	3.00
3.00	3.00	58.00	1.00	8700.00	5700.00	633.33	3.00
1.00	1.00	20.00	0.00	5500.00	2500.00	833.33	1.00
2.00	2.00	34.00	0.00	10700.00	7500.00	833.33	2.00
2.00	2.00	33.00	0.00	8000.00	5200.00	650.00	2.00

3.00	3.00	45.00	1.00	8500.00	5400.00	360.00	3.00
1.00	2.00	24.00	0.00	8000.00	5000.00	416.67	2.00
1.00	2.00	33.00	0.00	10000.00	6700.00	1116.67	2.00
1.00	2.00	31.00	0.00	7300.00	4600.00	255.56	2.00
1.00	1.00	22.00	0.00	5500.00	2700.00	1350.00	1.00
4.00	4.00	73.00	1.00	10000.00	7200.00	313.04	3.00
1.00	2.00	32.00	0.00	7400.00	4700.00	671.43	2.00
2.00	3.00	36.00	1.00	9000.00	5200.00	400.00	3.00
1.00	1.00	21.00	0.00	8260.00	5860.00	651.11	1.00
2.00	3.00	44.00	1.00	5100.00	1200.00	240.00	3.00
1.00	2.00	32.00	0.00	7300.00	4600.00	920.00	2.00
3.00	3.00	45.00	1.00	5900.00	2800.00	1400.00	3.00
2.00	2.00	34.00	0.00	9800.00	6500.00	928.57	2.00
1.00	2.00	24.00	0.00	7100.00	4900.00	445.45	2.00
1.00	1.00	22.00	0.00	6800.00	3800.00	1900.00	1.00
1.00	2.00	24.00	0.00	7900.00	4700.00	391.67	2.00
2.00	2.00	35.00	0.00	11900.00	8100.00	900.00	2.00
2.00	2.00	35.00	0.00	8000.00	5300.00	662.50	2.00
2.00	2.00	33.00	0.00	10500.00	7600.00	1266.67	2.00
1.00	2.00	29.00	0.00	6200.00	3700.00	616.67	2.00
1.00	2.00	32.00	0.00	7500.00	4400.00	550.00	2.00
2.00	2.00	35.00	0.00	9200.00	6800.00	523.08	2.00
2.00	2.00	33.00	0.00	8000.00	4600.00	460.00	2.00
1.00	1.00	21.00	0.00	5000.00	2600.00	866.67	1.00
2.00	2.00	35.00	0.00	10300.00	6900.00	383.33	2.00
1.00	1.00	18.00	0.00	6900.00	4000.00	666.67	1.00
1.00	2.00	32.00	0.00	8500.00	5800.00	644.44	2.00
1.00	1.00	22.00	0.00	5200.00	1800.00	900.00	1.00
3.00	3.00	47.00	1.00	7300.00	3900.00	780.00	3.00
2.00	3.00	43.00	1.00	9900.00	6800.00	323.81	3.00
1.00	1.00	23.00	0.00	9900.00	7200.00	1028.57	1.00
2.00	2.00	33.00	0.00	9900.00	6900.00	460.00	2.00
3.00	3.00	46.00	1.00	6800.00	3500.00	1750.00	3.00
1.00	2.00	27.00	0.00	10100.00	6900.00	313.64	2.00
1.00	1.00	18.00	0.00	6900.00	4200.00	1050.00	1.00
1.00	1.00	19.00	0.00	6900.00	3900.00	975.00	1.00
2.00	3.00	36.00	1.00	12400.00	9300.00	387.50	3.00
1.00	2.00	31.00	0.00	6000.00	3300.00	1650.00	2.00
4.00	4.00	75.00	1.00	6700.00	4200.00	1050.00	3.00
1.00	1.00	21.00	0.00	4200.00	1300.00	650.00	1.00
3.00	4.00	68.00	1.00	12100.00	9100.00	379.17	3.00
1.00	2.00	31.00	0.00	8600.00	5800.00	725.00	2.00
2.00	2.00	33.00	0.00	7700.00	4300.00	537.50	2.00
2.00	3.00	44.00	1.00	9200.00	7100.00	338.10	3.00
3.00	3.00	51.00	1.00	6400.00	3800.00	542.86	3.00
1.00	2.00	31.00	0.00	7100.00	5100.00	392.31	2.00
3.00	3.00	47.00	1.00	7100.00	4600.00	575.00	3.00

2.00	2.00	33.00	0.00	10000.00	6800.00	680.00	2.00
1.00	2.00	25.00	0.00	8000.00	5300.00	407.69	2.00
2.00	2.00	33.00	0.00	9600.00	6400.00	581.82	2.00
1.00	2.00	29.00	0.00	9800.00	6300.00	572.73	2.00
2.00	3.00	38.00	1.00	7200.00	4300.00	330.77	3.00
1.00	1.00	20.00	0.00	5100.00	2600.00	866.67	1.00
1.00	2.00	31.00	0.00	7500.00	4700.00	522.22	2.00
4.00	4.00	70.00	1.00	9000.00	5700.00	407.14	3.00
1.00	1.00	19.00	0.00	7000.00	4400.00	880.00	1.00
1.00	2.00	32.00	0.00	9000.00	5700.00	814.29	2.00
1.00	2.00	28.00	0.00	10200.00	7000.00	538.46	2.00
1.00	2.00	26.00	0.00	8200.00	5000.00	500.00	2.00
1.00	2.00	31.00	0.00	9200.00	6100.00	610.00	2.00
1.00	2.00	31.00	0.00	10500.00	8000.00	888.89	2.00
2.00	3.00	36.00	1.00	6600.00	4100.00	1366.67	3.00
2.00	3.00	43.00	1.00	8200.00	4900.00	700.00	3.00
3.00	4.00	61.00	1.00	7100.00	3800.00	760.00	3.00
2.00	2.00	34.00	0.00	9300.00	6100.00	554.55	2.00
1.00	1.00	19.00	0.00	6600.00	3600.00	720.00	1.00
4.00	4.00	70.00	1.00	7400.00	4000.00	1333.33	3.00
1.00	1.00	21.00	0.00	10600.00	7700.00	855.56	1.00
3.00	3.00	45.00	1.00	7400.00	4400.00	220.00	3.00
1.00	2.00	30.00	0.00	9500.00	6200.00	344.44	2.00
1.00	1.00	22.00	0.00	6100.00	3000.00	1500.00	1.00
1.00	2.00	31.00	0.00	5500.00	2200.00	550.00	2.00
1.00	1.00	18.00	0.00	8500.00	6500.00	1625.00	1.00
2.00	2.00	33.00	0.00	7700.00	4300.00	477.78	2.00
3.00	4.00	61.00	1.00	5500.00	2300.00	766.67	3.00
3.00	4.00	62.00	1.00	11300.00	8300.00	345.83	3.00
1.00	1.00	20.00	0.00	6000.00	3000.00	1500.00	1.00
1.00	2.00	25.00	0.00	9100.00	6300.00	900.00	2.00
3.00	3.00	55.00	1.00	10100.00	7800.00	325.00	3.00
3.00	3.00	49.00	1.00	11200.00	8200.00	341.67	3.00
1.00	2.00	31.00	0.00	10100.00	7300.00	663.64	2.00
2.00	3.00	36.00	1.00	13200.00	10200.00	425.00	3.00
2.00	3.00	41.00	1.00	9500.00	6400.00	533.33	3.00
1.00	1.00	21.00	0.00	7100.00	4500.00	500.00	1.00
3.00	3.00	53.00	1.00	7700.00	4600.00	353.85	3.00
1.00	1.00	20.00	0.00	4700.00	1800.00	600.00	1.00
4.00	4.00	71.00	1.00	12900.00	10000.00	476.19	3.00
1.00	2.00	31.00	0.00	11300.00	8500.00	944.44	2.00
1.00	2.00	31.00	0.00	8400.00	5600.00	933.33	2.00
3.00	4.00	61.00	1.00	11500.00	8100.00	352.17	3.00
1.00	1.00	20.00	0.00	5800.00	3300.00	825.00	1.00
4.00	4.00	70.00	1.00	4500.00	1300.00	650.00	3.00
1.00	1.00	19.00	0.00	6400.00	3900.00	780.00	1.00
3.00	3.00	50.00	1.00	13500.00	10700.00	486.36	3.00

3.00	3.00	59.00	1.00	10500.00	7300.00	304.17	3.00
1.00	1.00	21.00	0.00	9800.00	7100.00	887.50	1.00
1.00	2.00	32.00	0.00	7500.00	4400.00	628.57	2.00
1.00	1.00	38.00	0.00	8900.00	6100.00	1525.00	1.00
4.00	4.00	72.00	1.00	12400.00	9900.00	412.50	3.00
1.00	1.00	21.00	0.00	8500.00	5800.00	527.27	1.00
3.00	4.00	68.00	1.00	9800.00	7100.00	507.14	3.00
1.00	2.00	31.00	0.00	8200.00	5500.00	550.00	2.00
3.00	3.00	54.00	1.00	9100.00	6100.00	305.00	3.00
2.00	2.00	33.00	0.00	7100.00	4100.00	512.50	2.00
3.00	3.00	48.00	1.00	13500.00	10500.00	456.52	3.00
1.00	1.00	18.00	0.00	6700.00	3900.00	780.00	1.00
2.00	2.00	33.00	0.00	9100.00	5600.00	622.22	2.00
4.00	4.00	75.00	1.00	8200.00	5600.00	1120.00	3.00
1.00	1.00	21.00	0.00	5900.00	2500.00	833.33	1.00
2.00	3.00	38.00	1.00	10500.00	7400.00	308.33	3.00
1.00	1.00	19.00	0.00	10100.00	7600.00	690.91	1.00
2.00	2.00	35.00	0.00	4800.00	1800.00	900.00	2.00
2.00	3.00	40.00	1.00	7000.00	3800.00	633.33	3.00
2.00	2.00	33.00	0.00	12000.00	9000.00	1000.00	2.00
1.00	1.00	19.00	0.00	7700.00	4500.00	1125.00	1.00
3.00	3.00	48.00	1.00	11800.00	7800.00	325.00	3.00
3.00	4.00	65.00	1.00	10000.00	7400.00	321.74	3.00
1.00	1.00	21.00	0.00	8000.00	5500.00	916.67	1.00
2.00	3.00	41.00	1.00	12500.00	9700.00	461.90	3.00
1.00	1.00	19.00	0.00	8600.00	6300.00	525.00	1.00
1.00	2.00	28.00	0.00	8000.00	5100.00	425.00	2.00
1.00	2.00	25.00	0.00	7400.00	4900.00	445.45	2.00
2.00	2.00	34.00	0.00	8800.00	5300.00	757.14	2.00
1.00	1.00	23.00	0.00	15400.00	12400.00	590.48	1.00
1.00	2.00	31.00	0.00	8400.00	5700.00	438.46	2.00
2.00	2.00	33.00	0.00	8500.00	5500.00	687.50	2.00
2.00	3.00	43.00	1.00	11700.00	7700.00	427.78	3.00
2.00	2.00	34.00	0.00	8500.00	5300.00	588.89	2.00
1.00	1.00	20.00	0.00	6000.00	3200.00	800.00	1.00
2.00	3.00	44.00	1.00	6700.00	3800.00	1266.67	3.00
2.00	3.00	40.00	1.00	6200.00	3800.00	1266.67	3.00
1.00	1.00	21.00	0.00	9000.00	6800.00	566.67	1.00
3.00	3.00	54.00	1.00	12600.00	9600.00	533.33	3.00
1.00	2.00	27.00	0.00	5800.00	3000.00	750.00	2.00
3.00	3.00	47.00	1.00	5300.00	1800.00	900.00	3.00
1.00	1.00	18.00	0.00	6400.00	3700.00	616.67	1.00
1.00	1.00	23.00	0.00	7100.00	4300.00	1433.33	1.00
2.00	2.00	23.00	0.00	7000.00	4000.00	571.43	2.00
1.00	1.00	20.00	0.00	5600.00	2900.00	966.67	1.00
1.00	1.00	19.00	0.00	6700.00	3700.00	1233.33	1.00
3.00	3.00	54.00	1.00	6400.00	3200.00	400.00	3.00

1.00	1.00	18.00	0.00	6800.00	3500.00	875.00	1.00
1.00	2.00	32.00	0.00	6700.00	4300.00	1075.00	2.00
2.00	2.00	33.00	0.00	4500.00	1400.00	280.00	2.00
3.00	3.00	50.00	1.00	4600.00	1300.00	433.33	3.00
2.00	2.00	33.00	0.00	7800.00	4500.00	375.00	2.00
2.00	3.00	36.00	1.00	9000.00	6000.00	333.33	3.00
1.00	1.00	29.00	0.00	7600.00	5500.00	611.11	1.00
3.00	3.00	46.00	1.00	7300.00	4600.00	511.11	3.00
2.00	2.00	33.00	0.00	9200.00	6200.00	1240.00	2.00
3.00	3.00	54.00	1.00	8900.00	6400.00	800.00	3.00
3.00	4.00	61.00	1.00	5300.00	2100.00	1050.00	3.00
1.00	1.00	20.00	0.00	6500.00	3700.00	1233.33	1.00
1.00	1.00	21.00	0.00	6300.00	3300.00	1100.00	1.00
3.00	3.00	46.00	1.00	7600.00	4200.00	840.00	3.00
